# A computational investigation of kinetoplastid *trans*-splicing

**DOI:** 10.1186/gb-2005-6-11-r95

**Published:** 2005-10-17

**Authors:** Shuba Gopal, Saria Awadalla, Terry Gaasterland, George AM Cross

**Affiliations:** 1 Laboratory of Computational Genomics, Rockefeller University, 1230 York Avenue, New York, NY 10021, USA; 2 Department of Biological Sciences, Rochester Institute of Technology, 85 Lomb Memorial Drive, Rochester, NY 14623, USA; 3 Laboratory of Molecular Parasitology, Rockefeller University, 1230 York Avenue, New York, NY 10021, USA

## Abstract

*Trans*-splicing is an unusual process in which two separate RNA strands are spliced together to yield a mature mRNA. We present a novel computational approach which has an overall accuracy of 82% and can predict 92% of known *trans*-splicing sites. We have applied our method to chromosomes 1 and 3 of *Leishmania major*, with high-confidence predictions for 85% and 88% of annotated genes respectively. We suggest some extensions of our method to other systems.

## Abstract 2

A novel computational approach is presented and applied to predicting *trans*-splicing sites in 2 chromosomes of *Leishmania major*.

## Background

RNA splicing is a key process in the transformation of genomic instructions into functional proteins and may play a critical role in regulating gene expression in a variety of eukaryotes. Two forms of splicing have been documented in eukaryotes. Many eukaryotes use *cis*-splicing, the process of removing introns from precursor RNAs, to generate mature mRNAs. A related and less understood process, *trans*-splicing, appears most commonly in a family of protozoa known as the Kinetoplastida, although recent evidence suggests it might be quite widespread as well [1].

While much effort has been focused on identifying the sites of *cis*-splicing [2-4], a rigorous and thorough analysis of the likely sites for *trans*-splicing has been slower to appear. Yet the two processes may have common mechanisms, because many of the spliceosomal components are shared [5,6]. Indeed, in *Caenorhabditis elegans *it appears that both *trans*-splicing and *cis*-splicing occur in a coordinated fashion [7]. It is therefore possible that through consideration of the signals involved in *trans*-splicing, new insights can be gained regarding RNA splicing processes in all eukaryotes. As a first step in this direction, we present a computational analysis of *trans*-splicing signals from *Leishmania major*, a member of the Kinetoplastida family.

The Kinetoplastidae diverged approximately 800 million years ago from other eukaryotic lineages [8]. Perhaps as a consequence of this long divergence time, the various species of kinetoplastids exhibit features rarely seen in other eukaryotes. Many genes in kinetoplastids are co-transcribed as polycistronic pre-mRNAs [9-11]. A striking feature of these polycistronic transcripts is their sheer size; in *L. major*, polycistronic units have been identified that extend nearly half the length of a chromosome [12,13]. Cleavage to monocistronic transcripts is accomplished by the addition of a short spliced leader (SL, or mini-exon) sequence to the 5' untranslated region (UTR) of each transcript through a process known as *trans*-splicing. As in other eukaryotes, polyadenylation occurs at the 3' end of each mRNA.

In this paper, we use statistical methods to identify those regions most likely to be involved in *trans*-splicing and predict the most likely splice site(s). Specifically, we have observed that the AG dinucleotide that is most often used as the splice acceptor site is isolated from other AG dinucleotides by long stretches of non-AG dinucleotides. We applied this observation to develop a novel computational approach to predicting *trans*-splicing acceptor sites in the genus *Leishmania*. The method and results are presented here and some potential applications for the approach are discussed.

## Results

The canonical *trans*-splicing signal is believed to be composed of four elements: the branch-point adenine (A), a polypyrimidine (C, T-rich) tract, a short variable spacer region, and a downstream 3' splice acceptor site (AG) [9,14]. Of these signals, the easiest to detect computationally are the polypyrimidine tracts. However, these tracts can be highly variable in length (from 5 to well over 100 nucleotides in our datasets) and in composition (entirely pyrimidine or interspersed with purines).

In the analysis we describe here, we used two forms of sequence data. We use the term '*trans*-splicing region' to describe data that contain the upstream sequence region with the signals for *trans*-splicing. This region would include all four of the known signals for *trans*-splicing and possibly additional sequence information. In our datasets, approximately 400 nucleotides of upstream sequence are designated as the *trans*-splicing region for each sequence considered. Kinetoplastid 5' UTRs are fairly short, ranging in length from 40 to 200 nucleotides (based on a survey of GenBank entries). By utilizing 400 nucleotides of sequence, we could be reasonably confident that we had included all of the signals associated with *trans*-splicing. When we wish to refer to the *trans*-splicing splice site, we will use the term splice junction. This refers to the specific AG dinucleotide that will serve as the 3' splice acceptor site.

### Problem statement

In developing a computational method for predicting *trans*-splice junctions, we can take one of two approaches. We can identify putative coding regions within a genomic sequence, and then search upstream of these genes to locate putative splice junctions. This is not an ideal approach, because it is predicated on accurate gene prediction. No gene-prediction method to date is 100% accurate [15,16], and therefore we will inevitably miss some genes and their associated splice junctions. A second issue is that many gene-finding programs will find the longest possible open reading frame, even if the actual coding start of the gene is internal to the predicted start [15]. The longest open-reading-frame approach was used in the gene-prediction phase of genome annotation in *L. major *[17], and there are already some instances where the annotated start is known to be upstream of the functional start (AC Ivens, personal communication). When using predicted coding regions as an anchor for searching for splice junctions, we cannot account for such errors in predicting the start of the coding region. Clearly there are some disadvantages to relying on gene prediction as a means for anchoring the search for splice junctions within a genomic sequence.

Alternatively, we can develop a method that attempts to identify splice junctions independently of the presence or absence of genes. In essence, we would take the reverse approach from the one outlined above: we begin by finding all putative splice junctions, and then search downstream for regions that are likely coding regions. The first advantage of this approach is that we can potentially identify genes that were missed by a gene-prediction method. More importantly, we can refine the starts of predicted coding regions based on splice-site predictions. The coding start of a gene must per force be downstream of the *trans*-splicing junction, so this approach can both predict splice sites and refine gene predictions at the same time.

The disadvantage, of course, is that we must search long stretches of genomic sequence without a clear means for anchoring the search to likely regions of the genome. For this approach to yield reliable predictors of splice junctions, we must first find regions of the genome that are likely to contain splice junctions, and then attempt to identify the putative splice junction itself.

The approach we describe here follows the latter plan of action. In essence, the problem is twofold. There is a classification problem, namely classifying a sequence region as containing *trans*-splicing signals (what we term *trans*-splicing regions) or containing other signals. We can use any of a number of well known methods for sequence classification in this phase of the analysis.

Once we have identified sequence regions that are likely to contain a *trans*-splicing signal, we turn to the second half of the problem. We must now specifically identify the most likely splice junction or junctions within this putative *trans*-splicing region. This is a separate problem from classification of sequences, and we use a simple metric to identify the most likely splice junction(s) in a given sequence region.

### Dinucleotide composition is a reliable indicator of *trans*-splicing regions

The nucleotide composition of known *trans*-splicing regions is heavily skewed in favor of pyrimidines in general, even outside of the polypyrimidine tract believed to be part of the *trans*-splicing signal [18]. In previous work, we used the dramatic shifts in nucleotide composition between known *trans*-splicing regions and known coding regions as the basis for identifying likely coding regions. This was done in the related organism, *Trypanosoma brucei*. At the time, we were able to correctly identify 90% of known *trans*-splicing regions based on dinucleotide composition, for an overall accuracy of 93% in *T. brucei *[18]. We used linear discriminant analysis (LDA) to classify sequences based on dinucleotide composition.

We have now extended this analysis to *L. major*. We used 214 expressed-sequence-tag (EST)-mapped *trans*-splicing regions and 198 known, experimentally verified coding regions as described in Materials and methods. Because these datasets are relatively small, we used tenfold cross-validation to evaluate our models. As described in Materials and methods, tenfold cross-validation involves training on 90% of the data and testing on 10%. This is done over ten iterations, with each iteration involving a random split of both known positives (*trans*-splicing regions in this instance) and known negatives (coding regions) into the relevant training and testing datasets. Performance of the model is then averaged across the ten test datasets and reported [19]. We also note the range of values across the ten test sets to enable a more fine-grained evaluation of performance.

After tenfold cross-validation, we obtained the results shown in Table 1. Our LDA model has, on average, an accuracy of 96% (range of 90% to 100%). The sensitivity, or ability to identify known *trans*-splicing regions, is 97% (range of 91% to 100%), and the specificity, or ability to identify known coding regions, is 96% (range of 88% to 100%).

The high accuracy of the LDA model allowed us to reliably classify regions of genomic sequence that were likely to contain *trans*-splicing signals. To further improve our accuracy at this phase of the analysis, we considered only those predictions that had an individual confidence level of 95% or better. In other words, we only selected those sequence regions where the likelihood that the region was a *trans*-splicing region was 95% or better. Such predictions have an overall accuracy of 99% (data not shown).

### Identifying putative splice junctions within a *trans*-splicing region requires other metrics

Our LDA model is useful for locating the regions of the genome that are likely to contain a splice junction. However, the LDA model cannot on its own identify the actual splice junction. This is where the second phase of the problem must be addressed. We need a way to identify the most likely splice junction, specifically the AG dinucleotide that will serve as the 3' splice acceptor site. Below, we describe two such metrics and outline why we have selected the use of inter-AG distance as the primary metric for identifying splice junctions.

#### Polypyrimidine tracts are not reliable indicators of splice junctions

Our first hypothesis was that identifying the longest polypyrimidine tract would enable us to reliably identify the known splice junction. We used pattern matching to identify pyrimidine tracts and allowed variable numbers of purines to be interspersed. After extensive empirical testing, we determined that the fewest false positives were generated when up to two purines were allowed for every six pyrimidines identified. We defined a false positive as a polypyrimidine tract that could be found with equal probability in known *trans*-splicing regions and known coding regions. Thus the sequence YYRYYYYRYY (Y = pyrimidine, R = purine) would be accepted, but YYRRRRRYYY would not be accepted as a pyrimidine tract.

We applied this approach to 214 known *trans*-splicing sites derived from EST mappings (see Materials and methods). Unfortunately, it appears that the longest polypyrimidine tract does not directly correlate with the true splice site. In fact, only 51% of known splice sites in the test dataset were correctly predicted using this approach (see Additional data file 1).

We highlight this finding because many previous efforts at identifying the splice junction have focused exclusively on this signal [9,14,20]. Our data would suggest that while the polypyrimidine tracts are certainly necessary for *trans*-splicing, they do not appear to be sufficient to computationally pinpoint the specific splice junction with a high degree of accuracy.

#### The distance between AG dinucleotides seems to be a good indicator of splice junctions

**Figure 1:**
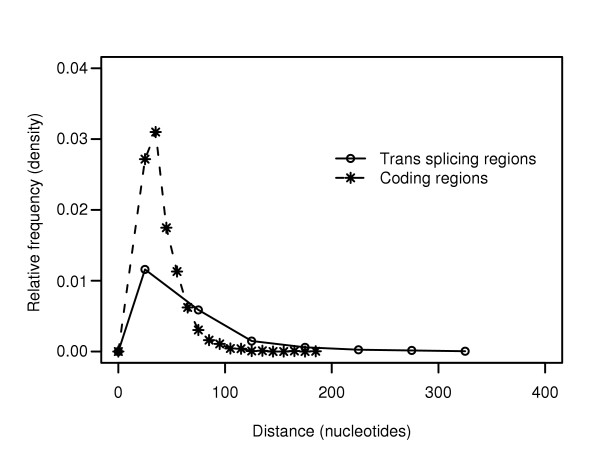
Inter-AG lengths in known splicing and coding regions. Inter-AG distances in known coding and *trans*-splicing regions show different distributions. The distance between AG dinucleotides is significantly greater in known *trans*-splicing regions than in known coding regions. Distances are shown for 214 known *trans*-splicing regions and 198 coding regions. The mean inter-AG distance for the coding region data is 42 nucleotides, compared with a mean inter-AG distance of 81 nucleotides for known *trans*-splicing regions.

While analyzing our training data, we observed that AG dinucleotides show an unusual distribution in known *trans*-splicing regions (Figure 1). When compared with known coding regions, the inter-AG distances are significantly greater in known *trans*-splicing regions than in known coding regions. In addition, the longest inter-AG segments seem to correlate well with the known splice sites.

We therefore proposed as a second hypothesis that the inter-AG distance may be a good indicator of splice sites. Surprisingly, up to 60% of known splice sites can be exactly identified simply by selecting the longest inter-AG segment in known *trans*-splicing regions (data not shown). This is strikingly effective, given the simplicity of the measure.

### Overview of method and performance

As described below, we have been able to identify splice sites by combining the evaluation of dinucleotide composition with the inter-AG distance. The method has a mean accuracy of 82%, with a range of 74% to 93% in tenfold cross-validation (Table 2). The sensitivity, or ability to identify splice junctions in known *trans*-splicing regions, has a mean of 80% (71% to 93%), and a specificity, or ability to eliminate coding regions from predictions, of 85% (75% to 100%). Within *trans*-splicing regions, on average 92% (15.7 out of 17 predicted) of known splice junctions were correctly predicted (Table 3). Of these, 81% (12.6 out of 15.7) were exact predictions.

#### Approach to identifying splice junctions

These results were obtained by first extracting all possible inter-AG segments from the training datasets. Each training dataset had on average 192 *trans*-splicing regions (five sequences), which yielded on average 3,468 inter-AG segments (± 100 segments).

To evaluate the performance of the method, we extracted inter-AG segments from coding regions as well. We chose these sequences because we can reasonably expect that *trans*-splicing signals will not exist within a functional protein-coding region. However, there is a small probability that these coding regions will contain signals for *cis*-splicing, as there is some evidence of *cis*-splicing in kinetoplastids [21]. Therefore, some predictions within coding regions might be functional *cis*-splicing sites. Nevertheless, in the absence of a better set of negative controls, we have relied on coding regions as our best representative of sequences that do not contain splicing signals.

For each inter-AG segment, regardless of whether it was from a *trans*-splicing or a coding region, we then used LDA to classify the sequence based on dinucleotide composition. We retained any inter-AG segment that had a 95% confidence or better likelihood of being a *trans*-splicing region (see Materials and methods).

**Figure 2:**
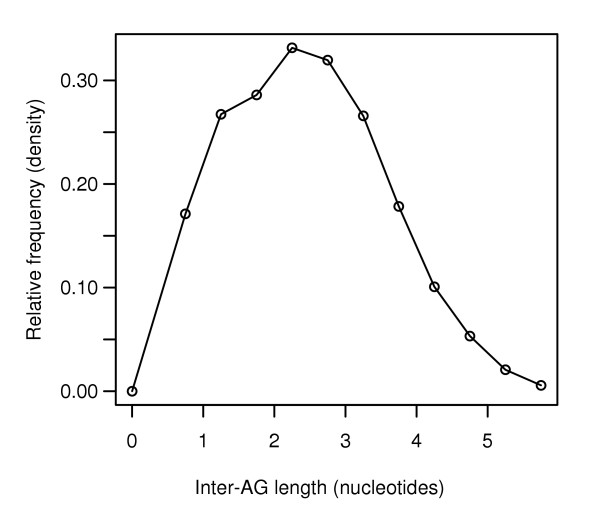
Log-normal transform of inter-AG lengths. Inter-AG distances after log-normal transform show a roughly normal curve. To better evaluate the inter-AG distances in known *trans*-splicing regions, we transformed the long-tailed distribution seen in Figure 1 using a log-transform. The result is a good approximation to a normal curve, allowing us to use the full panoply of statistical analyses available for manipulations of normally distributed data.

With the set of inter-AG segments that had the best dinucleotide composition for each *trans*-splicing region, we next evaluated the inter-AG length. The distribution of inter-AG lengths seen in Figure 1 is quite long-tailed, so we log-transformed the data to approximate a normal curve (Figure 2). We then used the *z *score as a measure of how a given inter-AG length compares to the mean of the distribution. The larger the *z *score value, the more standard deviations lie between that inter-AG length and the mean of the distribution [19,22].

**Figure 3:**
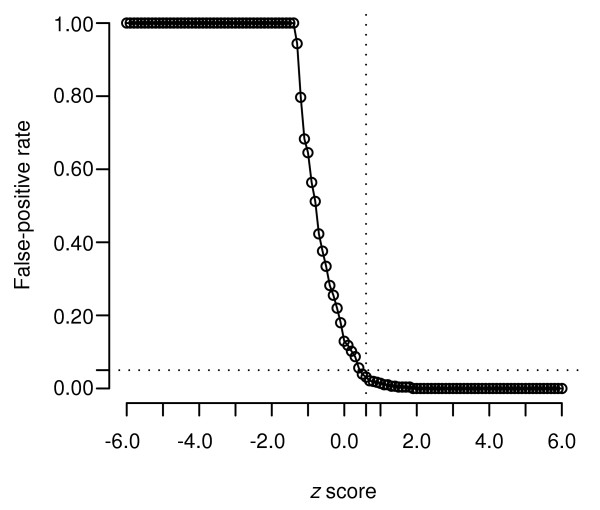
False-positive rate as a function of *z *score. False-positive rate as a function of *z *score can be used to measure the confidence of an individual prediction. The rate of false positives predicted by the method is shown as a function of the *z *scores used to evaluate inter-AG distances. False-positive rates were estimated for a range of *z *scores from -6 to +6 based on known splice sites in the training data. The dotted lines indicate that a z score of 0.6 or greater will yield a false positive rate of just 5%. In other words, inter-AG segments with a z score of 0.6 or greater will have a 95% confidence of being *trans*-splicing regions.

We could also assign a confidence value to each individual prediction of a splice site by considering the number of false positives likely to occur at a given *z *score. We used the training data and results from both *trans*-splicing regions and known coding regions to determine confidence levels. This would allow us to estimate the likelihood that a given inter-AG length was indicative of a known splice site. We wished to identify an optimal *z *score such that the false-positive rate could be as low as possible. In Figure 3, a *z *score of +0.6 yields a false-positive rate of just 5%. Therefore, any inter-AG segment with a *z *score of 0.6 or greater would have a 95% confidence in the prediction. In all of our analyses, we consider only these high-confidence predictions in assessing the validity of our method.

#### Splice junction identification in sequences

On average, the model predicts 1.25 high-confidence splice junctions per *trans*-splicing region. In other words, most *trans*-splicing segments have one splice junction prediction, with a few having two predictions per sequence. We only have evidence for one splice junction per sequence in this dataset, so at first glance, this might suggest that the false-positive rate is higher than our estimates from coding regions would suggest. However, there is experimental evidence that *trans*-splicing may occur at multiple sites upstream of some coding regions, and it may be specific to certain stages within the life cycle [23-29]. Thus, it is possible that multiple, valid *trans*-splicing sites exist for any given transcript. Any computational method for identifying splice sites may therefore identify sites that are functional in some limited context, but for which we do not currently have experimental validation. Predictions that might currently be construed as false positives may prove to be functional as experimental evidence accrues.

#### Similar performance on data with multiple, known splice junctions

To test the ability of our method to identify multiple known *trans*-splicing sites, we assembled a small dataset of 21 genes from *Leishmania *species. These genes each contain experimental evidence for more than one *trans*-splicing site within their upstream regions. A total of 36 splice sites have been experimentally confirmed within the upstream regions of these genes, and 27 (75%) were identified with high confidence by the method. Of these predictions, 19 (70%) mapped exactly to known splice sites. Allowing for a small window of error of ten nucleotides (error of 0.002 given the length of the sequences analyzed), the method had near exact predictions for 85% (23 out of 27) of the known sites that were identified (full details in Additional data file 2). These findings are very similar to the results obtained from our EST-mapped set of *trans*-splicing signals.

### Application to genomic sequence

We have applied our method to the entire genomic sequence of chromosomes 1 and 3 of *L. major*. We would emphasize that this represents an example of how our method might be applied and is not presented as a mechanism for evaluating the performance of our method.

There are four challenges to analyzing these data. First, the chromosomal sequences are much longer than any of our other datasets, on the order of several hundred kilobase pairs (kbp). This dramatically raises the statistical noise present in the data. Second, both strands of the genomic sequence must be analyzed for accurate predictions. In all previous datasets, only the strand known to contain the *trans*-splicing site was evaluated. Third, few of the genes annotated on these chromosomes have been experimentally evaluated for *trans*-splicing. Therefore, we must compare our predictions with the approximate region in which a prediction would yield a reasonable transcript, as described below. Finally, not all genes annotated on these chromosomes have a clear biological function assigned. Some of the genes will have annotations such as 'hypothetical' or 'conserved hypothetical.' For our purposes, we decided to accept each annotation in the publicly released data, but in Tables 3 and 4 we break down performance based on the category of annotation.

For both chromosomes, we evaluated the entire genomic sequence in both the forward and reverse complement directions. After predicting splice sites regardless of the location of annotated coding regions, we considered the number that were within a reasonable distance upstream of an annotated gene. The key constraint here is the length of the 5' UTR that might result if *trans*-splicing occurred at the predicted site. Kinetoplastid 5' UTRs are fairly short, ranging in length from 40 to 200 nucleotides (based on a survey of GenBank entries). We therefore considered a prediction as being a reasonable prediction if it was within 400 nucleotides of the annotated start of the coding region. This would yield a UTR that would be within the observed range of lengths.

On chromosome 1, 71 of the 84 (85%) genes have a high-confidence prediction within 400 nucleotides of the annotated start of the coding region (Table 4). Of the remainder, ten genes had low-confidence predictions, and only three genes were missed entirely by the method (i.e. no prediction within 400 nucleotides of the annotated start). Of the missed genes, one was annotated as 'hypothetical' and the other two were annotated as 'conserved hypothetical'.

The results for chromosome 3 are similar: of 98 annotated genes, 86 (88%) had high-confidence predictions (Table 5). The other 12 genes had low-confidence predictions. No genes were missed for this chromosome.

These results are very consistent with the method's performance on other datasets, suggesting that the method is robust and can be applied to long genomic sequences. The few instances where we have missed genes may be instances in which the annotated start of the open reading frame varies from the functional start of the coding region. We explore this issue in more detail in the Discussion. A more detailed version of these results is included (see Additional data file 4), and a graphical representation of predicted splice site locations along each chromosome is also available [30].

## Discussion

The ability to identify a small signal in much longer sequences is a critical issue in the computational identification of both *trans*-splicing and *cis*-splicing sites. The results seen here mirror similar results from methods attempting to identify the 3' acceptor site in *cis*-splicing [16]. While intronic sequences are typically longer than the upstream *trans*-splicing regions used here, a comparison in performance is still valid. This is because our method attempts to identify *trans*-splicing sites without prior knowledge of coding region location. As a result, our method scanned the entire length of each chromosome or genomic region available for analysis. These lengths are more than equivalent to the intron-length scans used by many other gene-prediction methods [16]. Given the nature of the signal, our method performs as well as most existing tools that identify 3' acceptor sites in *cis*-splicing.

In contrast to other methods for identifying splicing signals, however, our method takes advantage of two relatively simple statistical measures. Nucleotide composition and inter-AG distance seem to be almost too simple, and it would appear that a more powerful method would yield better results. Indeed, most tools for *cis*-splicing use complex probabilistic models such as hidden Markov models to identify splice sites effectively [2,31]. Such methods could indeed further our ability to identify *trans*-splicing sites, if sufficient data were available to correctly train such methods. As the complexity of a statistical model increases, so does the quantity of data required for accurate and reliable modeling. In our case, the paucity of known *trans*-splicing sites limits the applicability of complex statistical models. Our method represents, to our knowledge, the best available option given the need for predicting these regions and the limited data available for modeling the features of the *trans*-splicing junction.

A key advantage of our method is that it can identify splice sites without *a priori* knowledge of the location of coding regions. One of the challenges with gene prediction in the kinetoplastids is that the longest open reading frame present in a genomic segment does not necessarily correlate with the true open reading frame in the mRNA. Currently, the most popular tool for gene prediction in the kinetoplastids will always predict the longest possible open reading frame [15].

Our method could be used to refine the identification of the true open reading frame. If a genomic segment predicted to contain a gene also contained a high-confidence splice site within the putative open reading frame, that would be strong evidence for an internal start rather than the furthest upstream start codon. Conversely, if the high-confidence splice site were upstream of the furthest upstream start codon, then it would argue in favor of retaining that start codon as the functional start of the gene. In this manner, true coding regions might be more effectively identified. This could in turn assist in subsequent experimental studies of gene function.

A second advantage of our method is that it can predict more than one splice junction in a given sequence region. Given the possibility of multiple splice sites for *trans*-splicing in kinetoplastid genes, any method for splice-site prediction must be able to account for this phenomenon. Since our method provides a confidence estimate with each prediction, it is possible to evaluate multiple splice-site predictions for the likelihood that they are functional. This should provide others with the means to evaluate these predictions in an experimental context.

Our method also suggests interesting avenues for further research into the phenomenon of *trans*-splicing. Two models have been proposed for the mechanism by which splicing might occur. The first model would argue for a set of highly conserved motifs that direct the spliceosome to a specific location and a target AG that will serve as the 3Â' splice acceptor site. For example, *cis*-splicing sites in *Saccharomyces cerevisiae *include highly conserved sequences immediately upstream of the 3Â' splice acceptor site that allow for precise splicing at the correct location [32]. In the second model, the spliceosome would employ a scanning technique, evaluating each AG as a potential splice site [33-36]. In this model, strongly conserved signals are not required, but consistent nucleotide context, including a polypyrimidine bias, would be critical for the spliceosome to identify candidate splice acceptor sites.

The success of our method, which does not rely on strict consensus sequence features, favors a scanning model, where the *trans*-splicing process can occur at any AG dinucleotide that satisfies the general requirements for a splice site. The experimentally observed utilization of multiple *trans*-splicing sites in a given upstream region is consistent with this view of the *trans*-splicing mechanism. Indeed, splicing probably occurs sequentially at multiple upstream sites, with differing efficiencies, until no further site is recognized and a stable mRNA is generated almost by default. In effect, the final mRNA is formed by lack of further recognizable splice sites as the spliceosome, perhaps linked to the transcription machine, passes along the nascent RNA. We would encourage the use of the findings presented here not only in their predictive capacity, but as an impetus for further study of this intriguing process.

The method presented here is easily extensible to other members of the kinetoplastid family, and perhaps to other organisms that exhibit *trans*-splicing. It may also be possible to generalize this approach to improve on the prediction of 3' splice acceptor sites in *cis*-splicing. Preliminary results with human introns and exons indicate a distribution of inter-AG lengths that is almost identical to the distribution shown in Figure 1 (based on human intron and exon sequence data from [37]; data not shown). Thus, it is possible that the study of signals underlying *cis*-splicing might benefit from cross-fertilization with the methods we have developed for *trans*-splicing.

## Conclusions

We present a method for identifying the 3' splice acceptor site during *trans*-splicing, an unusual process in which two RNAs are spliced together to yield a mature mRNA. Our method is able to predict 92% of known splice sites with high confidence, and 81% of these predictions map exactly to the known splice site. Based on the statistical measures we use, it appears that our method would support the scanning model of *trans*-splicing rather than the model of site-specific binding by the spliceosomal apparatus.

We propose that our method might be applied to refine gene prediction in kinetoplastid genomes by assisting in the identification of the true starts of coding regions. In addition, our method could possibly be extended to other organisms and may even be relevant to the study of 3' acceptor site selection in *cis*-splicing.

## Materials and methods

### Data

Our data consisted of upstream sequences known to be involved in *trans*-splicing and experimentally verified coding regions from *L. major*. We used tenfold cross-validation to train and test our method. In addition, we had an independent test dataset of experimentally verified splice junctions derived from other members of the *Leishmania *genus. The latter data are described in more detail in Additional data file 2.

#### Primary dataset

For our primary dataset, we started with 527 5' ESTs from *L. major*. These were mapped to the completed genome sequence of this protozoan, based on the genome sequence release of July 2005 [17]. Each of these ESTs was mapped to the genomic sequence using BLAST [38], with the requirement that the EST had to match the genomic sequence at 95% or greater sequence identity. In addition, the EST had to match no other genomic sequence with greater than 50% identity. By setting these very stringent levels on the EST mappings, we were able to obtain a set of 266 unique, nonredundant, highly accurate mappings of ESTs to the genomic sequence of *L. major*. Of these, 214 had a clear AG splice acceptor site immediately upstream of the mapped region, and these sequences were used for all aspects of the analysis described here.

While ESTs do tend to have higher rates of sequencing error, our stringent mapping to the genomic sequence allowed us to use the latter for all analyses. That is, we primarily used the genomic sequence that the EST mapped to, rather than the EST sequence itself. Therefore, we can be reasonably confident that the sequences do not contain the high error rates of the original EST sequence.

For each EST mapping, we obtained 400 nucleotides of sequence upstream of the 5' end of the EST mapping. This would be expected to contain the actual AG dinucleotide used as the splice acceptor site, as well as signals upstream of this AG dinucleotide.

In addition to these *trans*-splicing regions, we identified 198 coding regions from GenBank that had experimental evidence of function. The full set of GenBank accession numbers for these sequences is provided in Additional data file 3. We noted that four of these coding regions were present on chromosome 1 or 3 of *L. major *by using BLAST to map coding sequences to the genome.

With our dataset of 214 uniquely mapped *trans*-splicing regions and 198 coding regions, we generated ten sets of training and testing data for tenfold cross-validation. Using an *ad hoc* Perl script, we split the *trans*-splicing regions such that 90% were used for training in each set and 10% were used for testing. Similarly, we split the coding regions to generate ten sets of training and testing data. By training on each of the ten sets and then evaluating performance on the associated testing data, we were able to obtain reliable estimates of the method's performance.

#### Other data

We also used the publicly available genomic sequences for chromosomes 1 and 3 of *L. major *[12,13]. These were downloaded from GenBank ([GenBank:AE001274, GenBank:AC125735]), and the analysis was run on the entire genomic sequence. After the prediction of splice sites for the entire forward and reverse strands, any splice site that was within 400 nucleotides of the annotated start of a predicted gene was retained as a splice-site prediction. Those predictions were then evaluated for their confidence level as reported in Results.

### Algorithm development

The algorithm described here has three main stages of analysis. In the first stage, all inter-AG segments are extracted from a FASTA formatted file [39] of sequences using an *ad hoc* Perl script [40]. In the second phase of the analysis, the nucleotide content of each inter-AG segment is evaluated using linear discriminant analysis (LDA). Finally, the inter-AG length is assessed using the *z *score. For the LDA analysis, we needed a means of comparing sequences of differing lengths and composition. We compared the *trans*-splicing regions to coding regions at the dinucleotide level using transition probabilities. We applied maximum likelihood estimation (MLE) to estimate these probabilities, since this method allows for estimation from a relatively small sample size. MLE-based transition probabilities are calculated by the formula:



where a_kl _is the transition probability that the nucleotide l follows the nucleotide k, and c_kl _is the number of times the dinucleotide combination _kl _occurs. In the denominator, we calculate the sum of the transition probabilities of all nucleotides that could follow k, represented by l' as any of the four nucleotides [41].

We used these transition probabilities to train LDA, which, as implemented in the statistical package R, was used for this analysis [42]. We then tested performance of the LDA approach on the test datasets. The training and testing occurred over ten iterations.

Each prediction by LDA is accompanied by a posterior probability value, a measure of the likelihood that a given individual prediction is correct [42]. We used these posterior probabilities to select those inter-AG segments that scored at the 95% confidence level or higher. We have previously shown that selecting sequences with a 95% confidence in the individual predictions leads to an overall classification accuracy of 98% [18]. Therefore, selecting inter-AG segments that had such high posterior probability values ensured that true *trans*-splicing regions were selected with a high degree of confidence.

The selected inter-AG segments were then assessed based on the inter-AG segment length. For each inter-AG segment, a *z *score was calculated based on the mean and standard deviation of the log-transformed distribution from the training data.

We calculated *z *scores as follows:



where *z *is the *z *score, x is the log-transformed inter-AG distance, μ is the mean of the log-transformed distribution, and σ is the standard deviation of the log-transformed distribution [19]. For the *L. major *training dataset, μ was 3.351 and σ was 0.881 averaged across the ten cross-validation datasets.

To estimate the likelihood that a given prediction was a true assignment, we used the false-positive rate, determined from the training dataset, as a function of *z *scores. That is, for each *z *score from -6 to +6, we determined the proportion of predictions that were false positives (known nonsplice sites predicted to be functional splice sites) assigned at each *z *score. This led to the plot shown in Figure 3. From this, we determined that a *z *score of 0.6 or higher would be likely to represent a false-positive rate of just 5%. In other words, we could be 95% confident that a prediction made at this *z *score represented a true positive. Thus, any prediction with a *z *score of 0.6 or greater is considered a high-confidence prediction. High-confidence predictions for the various datasets are reported in Results.

## Additional data files

The following additional data are available with the online version of this paper. Additional data file 1 is a PDF file describing the method, data, and detailed results for the identification of polypyrimidine tracts within known *trans*-splicing regions. Additional data file 2 is a PDF file containing the data and detailed results for our dataset of multiple splice sites with experimental confirmation. Additional data file 3 is a PDF file containing all the data used in this analysis; the sequence data are presented in the FASTA format. Additional data file 4 is a PDF file containing the full set of predictions for chromosomes 1 and 3 of *L. major*.

## Supplementary Material

Additional data File 1 PDF file describing the method, data, and detailed results for the identification of polypyrimidine tracts within known *trans*-splicing regions Click here for file

Additional data File 2 PDF file containing the data and detailed results for our dataset of multiple splice sites with experimental confirmation Click here for file

Additional data File 3 PDF file containing all the data used in this analysis; the sequence data are presented in the FASTA format Click here for file

Additional data File 4 PDF file containing the full set of predictions for chromosomes 1 and 3 of *Leishmania major* Click here for file

## Figures and Tables

**Table 1:** Classification of sequences by linear discriminant analysis (LDA)

	Known *trans*-splicing regions	Known coding regions
Predicted *trans*-splicing region	True positive	False positive
	20	0.9
	(14-24)	(0-3)
		
Predicted coding region	False negative	True negative
	0.7	18.5
	(0-2)	(10-31)
		
	Sensitivity: 0.97	Specificity: 0.96
	(0.91-1.00)	(0.88-1.00)
		
	Accuracy: 0.96	
	(0.90-1.00)	

The overall performance of the LDA method after tenfold cross-validation using 214 known *trans*-splicing regions and 198 coding regions is shown here. The average across all ten testing sets is reported, with the range of values indicated in parentheses for each class of sequence. Each test dataset had on average 20.7 known *trans*-splicing regions and 19.4 known coding regions.

**Table 2:** Identification of splice junctions

	Known *trans*-splicing regions	Known coding regions
Predicted splice sites	True positive	False positive
	17	2.5
	(10-22)	(0-4)
		
Predicted nonsplice sites	False negative	True negative
	4.5	13.9
	(1-8)	(11-16)
		
	Sensitivity: 0.80	Specificity: 0.85
	(0.71-0.93)	(0.75-1.00)
		
	Accuracy: 0.82	
	(0.74-0.93)	

The overall performance of the method in identifying splice junctions was determined by comparing the number of known splice junctions that were identified by the method in known *trans*-splicing regions versus those in known coding regions. These results are from tenfold cross-validation, and each test dataset had on average 21.5 known *trans*-splicing regions and 16.4 known coding regions.

**Table 3:** High-confidence predictions for known *trans*-splicing regions

Distance from known site (nucleotides)	Number of regions with sites predicted (*n *= 17 regions)
Exact matches	12.6 (7-16)
10	1.38 (1-2)
25	1.5 (1-3)
50	1.38 (1-2)
Missing predictions	4.5 (1-8)

Overall performance of the method on a set of known *trans*-splicing regions (tenfold cross-validation of 214 EST mapped *trans*-splicing sites). Each test dataset had on average 21.5 known *trans*-splicing regions, of which on average 17 had predictions. Missing predictions indicate those sequences for which no high-confidence prediction was available or where the nearest prediction was more than 50 nucleotides away. The mean of all ten datasets is reported with the range of values in parentheses.

**Table 4:** Predictions for chromosome 1

Public annotation	Totals	High confidence	Low confidence	No prediction
Forward strand				
Protein function assigned	22	20	2	0
Conserved hypothetical	18	16	0	2
Hypothetical	13	9	3	1
Total	53	45	5	3
Reverse strand				
Protein function assigned	9	8	1	0
Conserved hypothetical	18	14	4	0
Hypothetical	4	4	0	0
Total	31	26	5	0

Comparison of predicted splice sites with annotation of chromosome 1 of *Leishmania major*. A total of 84 genes have been annotated on chromosome 1 of *L. major *[12]. Of these, the method finds a splice site with a high-confidence score in all but 13 instances (85%). Only three genes were missed entirely by the method, with no prediction within the 400 nucleotide window upstream of the annotated start of the gene.

**Table 5:** Predictions for chromosome 3

Public annotation	Totals	High confidence	Low confidence	No prediction
Forward strand				
Protein function assigned	18	14	4	0
Conserved hypothetical	5	5	0	0
Hypothetical	44	40	4	0
Total	67	59	8	0
Reverse strand				
Protein function assigned	7	6	1	0
Conserved hypothetical	2	2	0	0
Hypothetical	22	19	3	0
Total	31	27	4	0

Comparison of predicted splice sites with annotation of chromosome 3 of *Leishmania major*. A total of 98 genes have been annotated on chromosome 3 of *L. major *[13]. Of these, the method finds a splice site with a high confidence score in all but 12 instances (88%). A splice site was predicted for every gene annotated on this chromosome.
