# Supplementary material for: A computational investigation of kinetoplastid trans-splicing
Source: Genome Biol. 2005 Oct 17;6(11):R95. doi: 10.1186/gb-2005-6-11-r95 (PMC1297651; doi:10.1186/gb-2005-6-11-r95)
Supplement: Additional data File 1 — PDF file describing the method, data, and detailed results for the identification of polypyrimidine tracts within known trans-splicing regions [file gb-2005-6-11-r95-S1.pdf]

Utilizing Poly-pyrimidine Tracts to Identify *trans*-splicing Sites

Results

The *trans*-splicing site is characterized by three sequence-based elements. The best characterized is the poly-pyrimidine tract. These long runs of cytidine (C) and thymidine (T) nucleotides are the predominant feature of known *trans*-splicing regions. As already discussed, however, these regions tend to be highly variable in terms of composition and length from one *trans*-splicing region to the next. We began our analysis by attempting to identify the longest pyrimidine tract present in known *trans*-splicing regions.

It is apparent even from the small sample in Table 1 that simply identifying the longest consecutive string of pyrimidines would be insufficient to capture the pyrimidine tracts. We had to accept some purines interspersed in runs of pyrimidines. After testing multiple combinations of pyrimidines interspersed with purines, the fewest false positives could be ensured by allowing up to two purines between pyrimidines. We added a constraint that at least six pyrimidines should precede or follow such purines. That is, the sequence YYRRYYYYYYY (Y=pyrimidine, R=purine) would be accepted, but the sequence YRRYYYYRRR would not be accepted as a pyrimidine tract. In addition, up to two more single purines were accepted as long as they were preceded or followed by six pyrimidines. For example, the sequence YRYYYYYYRYYRRYYYYYY would be accepted.

These criteria, while determined empirically from the training data set (see Methods), are also supported by experimental analyses. In *Trypanosoma brucei*, experiments have demonstrated that the efficiency of *trans*-splicing is impacted by variations in pyrimidine tracts directly upstream of the known splice acceptor site. Pyrimidine tracts may contain up to two consecutive purines without a loss of splicing efficiency. However, when three or more consecutive purines are present, the efficiency of splicing drops one hundred fold [43].

We used our criteria to identify the longest such instance for each sequence within an independent test data set of 107 *L. major trans*-splicing regions. These sequences were obtained by mapping $5'$ expressed sequence tags (ESTs) to the *L. major* genome (see Methods for details). We then selected the first AG downstream of this poly-pyrimidine tract as the putative splice site. Table 2 shows the results of this approach when compared to the known splice site for each *trans*-splicing region in the data set.

Table 2 suggests that about half of all the tested sequences conform to the proposed model of *trans*-splicing regions where the AG downstream of the longest poly-pyrimidine tract is utilized as the likely splice site. Each sequence examined was 400 nucleotides in length, so a window of 10 nucleotides around the known splice site represents an error rate of 0.025 or 2.5%. This seems an acceptable range of error given that multiple splice sites are possible within a given sequence.

However, as shown in Table 2, many of the predictions are more than 10 nucleotides from the known splice site. Some are as far away as 100 nucleotides from the known splice site. In these instances, using the longest poly-pyrimidine tract to identify the splice site would lead to vastly erroneous predictions. Therefore, we consider any prediction that is more than 25 nucleotides from the known splice site to be an inaccurate prediction.

The results shown in Table 2 are not particularly surprising. Given the high variability in sequence composition of these poly-pyrimidine tracts, it would be extremely difficult to develop a set of rules that would allow us to capture the complete range of possible variation. For example, at least one of the sample sequences (GI:2695653) shown in Table 1 contains pyrimidine tracts that would not satisfy our criteria for demarcating a pyrimidine tract. Loosening the criteria for selecting pyrimidine tracts leads to a large number of false positives, with sequences in known coding regions being as likely to be selected as a

poly-pyrimidine tract as those in known *trans*-splicing regions (data not shown).

Materials and Methods

Data

The data used for this analysis are drawn from the EST-mapped data sets described earlier. The method was trained on the 107 EST-mapped sequences and tested on the remaining 107 EST-mapped sequences. In addition to these data, 198 known coding regions were extracted from GenBank for *L. major.* These were used to compare poly-pyrimidine tracts in known coding regions and known *trans*-splicing regions. For each of these genes, only the coding sequence (cds) was used.

Algorithm

Pyrimidine tracts were evaluated using a Java program. The program applied a greedy approach to identifying the longest pyrimidine tract in a given sequence. As the sequence was scanned, the occurrence of a pyrimidine would initiate a "tract-builder" that would keep track of additional pyrimidines and purines. The tract would be extended as long as there were at least six consecutive pyrimidines preceding or following every purine encountered. For each six pyrimidine segment, up to two purines were allowed. Tracts were terminated when additional purines were encountered or if the six pyrimidine segments ended. The position of each such pyrimidine tract was noted, and the longest tract for each sequence retained. The six pyrimidine constraint was determined by testing a range of pyrimidine combinations from two to ten. The number and length of putative pyrimidine tracts obtained were compared between sequences in the training data and sequences among known coding regions. The six pyrimidine length provided the best combination of sensitivity and specificity given the training data available.
To determine whether the longest pyrimidine tract could identify the true splice site, the first AG dinucleotide downstream of the longest pyrimidine tract was noted. These AG positions were compared to the known splice sites in the EST testing set. The distance between the selected AG and the known splice acceptor AG was noted and tabulated as shown in Results.

Tables

Table 1 Examples of Known trans-splicing Sites and Upstream Sequences

Ten instances of sequences from immediately upstream of the known splice junction in various *Leishmania* species. Pyrimidines are capitalized for ease of viewing; the AG dinucleotide that terminates each sequence is the experimentally determined splice junction. GenBank identification numbers are provided for each sequence as a reference to the original work detailing the identified splice junction.

GenBank ID	Sequence
GI:15488541	CTCCTCCggCaTgCgaCTgCTTCgTTgTCggTgCaTaaTgCaCTCgCgCgTCgTgg**ag**
GI:159405	gCagagCCCTgCCTCCCCgCCTCTCTgCCgTTggCaggTgaagCgaaaaCgaagCg**ag**
GI:2695653	aTTTCgCTgTgCTCTgCaTTTggTgCTgCTTgTCTgTCTCCgTgTgCgCaTgCgCC**ag**
GI:28625247	CCTTCaCCCCCCCCCTCCCCCTCCCCCTTCgTgTgCTTTCgaCaaCgTCTgTgT**ag**
GI:28804194	TCCTCTTTTTTCTCTgTCTTCTCTCCCTCgTgTgCCgTCgaCaaCgTCTgCgC**ag**
GI:293058	ggTaCCCCTTCTCCgCCaCgTCTCCTCCTCCCTCTCCTaTCCgCCaaaCCaCaCgC**ag**
GI:30248030	gTgTgCCCgaCTgTgTgTaCTgCTCTCTgCCTaTTTCTgCgTCaCTCggaTgagga**ag**
GI:312487	CaCaCaCaCgCaNNgTgCaCCgTgTgTCTaaCTCTCTTaCTgTgTCCCaCCgTCTT**ag**
GI:312490	CTCgCTTgCgTCTgaCTaaaTCTCCCCCCCCCTCCCCTCCCCCaCCgCaTgCCCgC**ag**
GI:3192902	aTaTaTaTaTTaTTCTTgTTTTCgTTTTgTgTTgCTCCCTCTaTTTTgCTTga**ag**
GI:3192904	CTCTTCCCCCTCTTCCCTCTCaTCgCCCTgTTCTCTgTgCCgTCaCTgggCgC**ag**

Table 2 Poly-pyrimidine tract based trans-splicing site predictions

Analysis using the longest poly-pyrimidine tract to identify the likely splice acceptor AG. Using this approach, 69 (51%) of the known splice sites were exactly identified. Allowing for some inaccuracies in prediction, 54% (73) of the known splice acceptor sites were identified within ten nucleotides of the known splice site.

Known Splice Sites

Total: 89 out of 136 sequences

Distance from known site (nucleotides)	Number of genes with sites predicted
Exact matches	69
+/− 10	4
+/− 25	8
+/− 50	8
Missing	47

43. Siegel TN, Tan KS, Cross GA: **A systematic study of sequence motifs for RNA *trans*-splicing in *Trypanosoma brucei*.** *Mol. Cell Biol.* 2005, **in press**.
