# Supplementary material for: A computational investigation of kinetoplastid trans-splicing
Source: Genome Biol. 2005 Oct 17;6(11):R95. doi: 10.1186/gb-2005-6-11-r95 (PMC1297651; doi:10.1186/gb-2005-6-11-r95)
Supplement: Additional data File 2 — PDF file containing the data and detailed results for our dataset of multiple splice sites with experimental confirmation [file gb-2005-6-11-r95-S2.pdf]

Performance on Multiple Splice Site Data

Results

A method for predicting *trans*-splicing sites must be able to identify more than one likely splice site in a given *trans*-splicing region if the conditions warrant such a prediction. Otherwise, the predictive ability of the method would be limited to identifying one of several possible splice sites. Therefore, we wished to assess our method's predictive ability on a set of known *trans*-splicing regions where more than one documented splice site exists. We created an independent data set with 21 genes and 36 experimentally verified splice sites. We note that these sites, while verified experimentally, lack data on the frequency or probability that alternative sites will be used. It is possible, in addition, that not all alternative splice sites have been identified, even in this data set. Nevertheless, this small data set provides the best opportunity to assess the method's ability to identify multiple *trans*-splicing sites in a given genomic region. The results are shown in Table 1.

A total of 36 splice sites have been experimentally confirmed within the upstream regions of these genes, and 27 (75%) were identified with high confidence by the method. Of these predictions, 19 (70%) mapped exactly to known splice sites. Allowing for a small window of error of $^+/-$ 10 nucleotides (error of 0.002 given the length of the sequences analyzed), the method had near exact predictions for 85% (23 out of 27) of the known sites that were identified. These findings are very similar to the results obtained from our EST mapped set of *trans*-splicing signals.
Given the lengths of the sequences searched, a window of 10 nucleotides represents an error rate of just 0.002. Furthermore, of those predictions that are within 10 nucleotides of the true site, the average distance from the true site is just 4 nucleotides. This suggests that the method is surprisingly robust to noise in the sequence data and is able to make predictions that are well within the range of reasonable error. Finally, this data set, albeit small, does provide some measure of confidence that the method is capable of identifying multiple *trans*-splicing sites in a given sequence.

Methods

Data

We developed the multiple splice site data from 21 genes of various*Leishmania* species, including *L. donovani*, *L. infantum*, *L.tarentolae*, *L. amazonensis* and *L. major.* Each of these genes was selected from GenBank because it had an experimentally verified *trans*-splicing site. In ten instances, more than one *trans*-splicing site had been experimentally identified for a given genomic region. Thus, a total of 36 known splice sites were present in this data set.

In addition, the entire sequence as reported in the GenBank entry for each gene was utilized, so that the sequence data included *trans*-splicing regions, coding regions and other inter-genic regions. As a result, this data set provided a preliminary indication of how the method might perform on longer stretches of genomic sequence. On average, each sequence in this data set had 4,500 nucleotides of sequence, ranging from 700 to 15,103 nucleotides. The GenBank accession numbers for these sequences were AY034610, AF109296, AF083881, AF067496, AF067495, AJ548878, AF031902, AY035390, AF406767, AF038409, AJ237587, L14605, AF058760, X97072, AF060886, X73120, X73119, X60102, L16952, AF016403 and AY273788.

Approach

The same approach detailed earlier, using inter-AG nucleotide composition and distance, was applied to this data set to obtain the results reported here.

Tables

Table 1 Independent Test Data with Multiple Known Splice Sites

Results of analyzing 21 genes from a variety of *Leishmania* species. This data set includes 36 known splice sites.

Known Sites

Total: 21 sequences, 36 splice sites

Distance from known site (nucleotides)	Number of sites predicted
Exact matches	19
$^{+}/-$ 10	4
$^{+}/-$ 25	4
$^{+}/-$ 50	0
Missing	1
