# Supplementary material for: A computational investigation of kinetoplastid trans-splicing
Source: Genome Biol. 2005 Oct 17;6(11):R95. doi: 10.1186/gb-2005-6-11-r95 (PMC1297651; doi:10.1186/gb-2005-6-11-r95)
Supplement: Additional data File 3 — PDF file containing all the data used in this analysis; the sequence data are presented in the FASTA format [file gb-2005-6-11-r95-S3.pdf]

Complete Data Sets

There were a total of three data sets used in this analysis. The first data set, consisting of sequences for training and testing the models, is included here. The sequence and splice site data for the *Leishmania* genes data set were derived from the GenBank entries for selected genes. The GenBank accession numbers for these genes are reported in Supplemental Data 2. The sequence and annotation data for chromosomes 1 and 3 of *Leishmania major* are publicly available from the Sanger Institute website [44].

EST mapped data

The training data consist of 214 EST-mapped sequences wwith a splice site determined as described in Methods. For each of the sequences here, 400 nucleotides of upstream sequence are reported. In each instance, the splice site is at position 400 (end of sequence) in the reported sequence. As described in Methods, ten data sets of training and testing data were generated from these data by randomly partitioning 90% of the sequences into a training set and reserving 10% for testing.

The sequence identifiers indicate the chromosome from which the sequence derives and list the dbEST identifier for the EST which was used in that mapping. For example, the id

```
>lmaj_01_2 with EST W88316 10020, 10420
```

indicates that the sequence derives from Chromosome 1 of *L. major*, is the second such EST mapping on Chromosome 1 and that the mapping derives from EST id W88316. The two coordinates 10020 and 10420 indicate the beginning and end of the extracted sequence region with regard to the chromosomal sequence.

>lmaj_04_1_up with W88315 10020, 10420
GTCGGTCCCCGTTGCTGCTGGGGACAAGGTGCAGCGGGCGAATGAGCTTTCATGGTGCTCTGTCGAACAG
CGTGTAGGTTAGGAGATGTTTCGGCCGTTTGTTTCGTTCGCATCCCGCTAGTCGAGCGCCGCGGCGTCGC
TGTTGACAAGACGTGAAGGGTGTGAACAGATACGCAGTGACATCCGCGGATGTTTTACGAGCGGAGATGC
TGTTGCCCTCTTCTTGCGTGCCGCCTTCACAGCCCTACTTTTTCATGTTTTCCCTTGTTGTAGGGTGGCG
TGTTCCCTGCATCGACGTCTCATCCCGCTTCTCTCATTGCGCTCTCCCTTTTTGCTTGAGCGTGTGCAAT
CTCTGCTGTGCTATCATCACCTCTCCCGTACCCACTTCACCCTTTGACAG
>lmaj_05_1_up with AA060765 413050, 413451
AGGGAGGCTTTCCTGCCCGACGACTGCACAGCCTTCTCTGCGTGACTACAACAACTCGATCTCTCCTGCT
CTTCCCGCCTCCCCCTCCGCCCATCGCCTCACGCGCACCTGCACGTGTACACACAGTGGTACACGCTGCG
CACAAACTTCGCGCACGCACACCGGTGGTTGCACTATTCTGTGTGGCGTTCATGTCTTCTCTGCCCGGCG
GTGATGCGTTATCACTGCGGCTTTACCGCTCCTGTACTGCTTCCCGTCTTCTTGGCCGCAGGTGGTGCGC
ACGCGCGGGACGGCAGCGACGGTGTCATGGAGAACTTTTCCATCTTTCTCTTCCTGTTCATCGGCGGCAT
CGCCCTGGGGGCGGTCATCATGCTGCCCATCGCGTATCTCCGCTGGCAAG
>lmaj_05_2_up with T93356 146697, 147098
CACCCCGGATTCCAGAAACGTTTCCGCCGGGACACGGAAGCGTTTCCGCCCCAGGGGCGCTTACGCGTGA
GTCCCACTGCGTGTGCAAAGCGTTTTTTTGTACCGTTTTCATCCTTACTCCTATTTTTCGCTGAAAAAGG
GTGTCGGTGGCGAATTCTTGCAAAAACCCGCTCTCTGCTACGCGACTCGAATTATGACAAGCGAAACACA
TCGCCTCTTGAGAGCGTCTTGGCTAGTGGAGGAGCACACAAGTTCCCTCCACTGCCTTTGTTGTCATGAC
TTTCTCACGCATCAGGCGTCGCAGACATTTGCTTCTCCACTCCCACTCACTTGCCCTCTTACTTCCTGAC
CTCTTGGCTTTCTTTCGATCTGCACTGTGTTACCAATTGTACACGAAAAG
>lmaj_05_3_up with W88316 82981, 83381
GGTGTGTCGCCCCGCGCTGTAACCCTCACAGGATTTCAATCTGGTGTGCCTTGTGCTCCTCTGGCTCAAA
GACGAACACGTGGCCTGAAACTATTTCGTTTCTGTTGTTGTCACCCAGTGAGGCACCATTCGTGCAACAC
ATTCGCTTGCGGCGTACCTGTAACTTTATCGGTTGCTTTCCCTGAAAAAGCGCTGTTACACTTTGCGATG
AATAGGGCTAAGCATAAATTCACGTATGTGATGTCGGCTTATTCCTCACAGTGGTTTCATTTCCACACAA
AAGATAACACCGTGGCATCCCGATTACTGCTCTCTTCTCTAGCATTCTCCCATTCCTCTCCCCTTTCTTT
GCCATACTGCTTCTTTCACTAACTCCGCTGATTTCCAAACAAAGGTGTAG
>lmaj_06_1_up with T67346 256151, 256551
TCCGTAACACGTCCCACCTGTTCACATCCGCACTCTTCGCCGGTGCGCCCCGACACACACACACACACTT
AGTGATGGCTCAAGCAGGACGTGACGGGTGGTACACAAGCAGAGAAGGGCGCTTGCGCACTCGCGCGGGT
GTGCATCTGCCGACATGTGCACTATCATACGCTCTTGCTCTCCTGCTACGACGCACACCAGGCGCGAGCT
GCCCTCTCTTACCCCATCTTCCCCCCTCCGCCGACCTTTGCCTCTCACGCGCGCACGCCCTCACCATCTC
CTCTCAACCGCTCCTCGTGCTTGCTTCTTTGGGCCCCTCCATCTCCCTCCCTCCCTCCATCTCCCTCCCT
CCCTCCATCTCCCTCCCTCCCCTCCCCACTGGCTCGCATGTGTGCGTGAG
>lmaj_06_3_up with W88361 131478, 131878
ATTATGAAATATCAATGGAGTCTCCACCGGCGGCAGACGTTACTTCCACCCAACCTCCGGTTTCTTCATG
GTGGGCGATGAGTGTAACCGTCGTGTGTTGCTGCGTGATAATAGTTGCAGCTTTGTAAACGAGCAGACCG
CTTGTCACAAAGAACAATACTATTCATTATAGCAGTTGCTCCCTTTCTCCTGCGACCACTTTCAAACAGA
TGTGACGGAAAGTAAAAGTGTCACACAGTGCTGTTGTCATCGCAGCTCTACAAAGAGCAATCCTTTTTTG
TGTTGGAATGCACACACTTCTCAACTCCCCACTTGCAAGCCTTTCTCAATTCCCATTTACGTCTTTTTTT
TTTCTTGGCCTCTCTTTCCACACTACTCTAAATCTCCACATCGAATGTAG
>lmaj_06_4_up with T67367 132057, 132457
TGTGACCATTGGCTTACTCGTAGCCGTTTCGGCTCGAGTATGAAACTGGAGACTGTTTTGTGCAAGCACC
CCACCTTTTTGGGGAAGGCTGCCTGCACTCACGCACTAGCATCCTTACTCTCTCCCTAGTGAAGTGTGCG

TGCAGAAGCTGTTGGTGACACGTTCTACTTTTTTTTGTTCTCCAATGGTCGTTAGAGGGAACGGACAACA
TTAGCGAGATGTCGAATGGCGGTTTGCTCTTCACAGATGCCGATTCTACCTTTTCGTTGACTAGATGAGC
CTTCTTTCCGCGCTGCGTAAGGGCGCCTATTTGTTGACTTTGCCGACTGGATTTCAGTCCATGCATGTAT
CACGACAACTTTCTTTCTCCTTTGCATTTCTTTGTTTGCCCCTCTCGCAG
>lmaj_08_1_up with W88346 265958, 266359
CCGCCGGTCAGAGCGCGGCACAGCGCGGGTGGGTGCGCCTGGGAGCGCTATCGGGGGCATCTCCCGTGTG
TGCGTGTGTGTGTGTGCCACCATCTACCCAACCTGAGCAGGGAAACGAACTAATGGCGGCAGCGTGGGGG
ATGACCAAGCATGCTGCTTTTTCACTGCTTCGGTTTTCTTCATCTCTGTCTGGGCGTCTCAGGAGGGCAC
GATCGGTTCGTCCACAGCCACGGTGAACAATTCAAGCGCGTACAAGCAGCGCGAATGGCCATGCCGACCA
GAAGCAGAGCCGCCTGGTCACACTCGCTTCTTCCCTCATCTCTCTCCCTCTCTCTCCCACCGTCTCTGCT
CTCTCTGCGCACTCCACCACTCCACCTCACCCCACCCTCCGCATCGTCAG
>lmaj_08_3_up with AA125621 137107, 137507
ACACACACACACACACACACACACACATACACGTACGCACGTACGCACGCGGACACCGATGCCTTGGGTGTG
GGTGGGACAGACATCCGGAGCCGAGGCAACACAAACCCTGCACCAGAGGGATGGGGCGTGCGGCAGGCTG
GTATGCAGAGTGGCGTCGGATTCATGCTCCATGGCGGAGAGTGGGCGCGTTGAAAGTGAAGGTTTTCGCC
GCTGTGCATGCAACGGCAGAGGAGCACACCGTGTGTGCACGCCACTCAGAGACCGATGATGCAGCTTCAC
CGATCATGCGCAGGGATGCCATAGCCTGTTGAGCTTTCTCTCTGCCGATATAGCATCCTCATCCTCCTCC
CTCTCTCTGCTTCCTTGCTCCTTGTCTCAAACATGTGCACACATCCACAG
>lmaj_09_1_up with H64195 95171, 95572
GACTTCAAGAGTTACAGAGCCCATTAAAGGGGCGCACGTGAGAGGCACAGCGCCCGCCCTCCCCTGCGCG
GACAGGAGCCGCAGCGACCCTTTAATCCTCTCTGTACACACATACACACAGACGTGCGCTTGCGTCCTTT
TCTTTCGCTTCACGTACATTGTGTCGCTCGTGGCTTCTCTGGTGGAGCAGGCGTTCATCCTGTCCGCAAG
AGCCCTCTCCATCTTCCCTGCACCATCGCCACTACCTCTTCTTCTCTCGCTGCTCTCGCCCCTCCGCCCT
GCGTCTCCACCCATACACACACATACACACATACACACACACACACATATATATATATATGTGTGTGTAC
GAGCACGTGTGTGTGTGCACGCGACGCCTTTACTCTCGGCCTGGGAAAAG
>lmaj_09_2_up with AA060775 167231, 167632
CTCTCTCTCTTCTGGTGGTTGCGCGTGTGCGTGTACGCCTTCGGGTGTTTCTTGTTTTTGTTTTTGTTCT
TTCGCTGGGGGGGGGGGGGGGGTGCTGACCGACCTCGGCCACACGATCGCACCACATCACTTCACCCCAC
CGCCGCGCCACGCGCACCCCAGCACGCACACAAGTGCACGTTCGCGACAGCCCTTCAGTGTGTGTGTGTG
TGTGTGTGTGTGTGTGGACGGGTGTGCCTACGCACAGACGCGCACCAAAGAAGCAGCTCCCGTCACATTT
TAGATGAAGCTGAGGCGCCCCACCCCCACGCGCCTTCACTGTTACAGGTTTTCTTGCGGACGTGCTCACC
GGCCCACACATCTACGCGCACGCACTGTCGTTGATGCTTCGCTGCGTAAG
>lmaj_09_3_up with W88340 22731, 23131
TTTCGGAGGGTCACATGTTGACGTAGCCGATGTCAACGGATATGCAAGAGCGATTCCCGTGAGAAGAAAG
CATGCAAGGCATTTGACTGCGCGGTGCCATTCATGGTGCTGCACCCTGACGCACCGAGGCGCGAAAGGAG
ACGTTGGCGCATCCGCGGGTAAACGTAGCCATCCCCCCAATCTCTAGGTCGCTCATCGGCTGCAACGCTG
CCTCGCTTGCGGCCCTGCTGGTGCCGATGAGTGCGGCTCCCTGGTGGGTTGCGGCCGATACATGGTGGAA
AACACGCCGCACTTGTCGTCTCTTGCTCTGCTCGGTGTGCTGCACCCCTCATCACTTCTTCGCTACGCCT
CTCTCTCTCTGCTTGTTATTCATTTCGTGCTTCCATTGTGCGTGCGCTAG
>lmaj_09_4_up with W88313 233327, 233728
CCGCCCTTCGCCTCACCCGTTAGCTGCCTTGGGCAGGTTGGCGGTCACGGCAGAGACGGAAGTGATGGGG
GAGGGGAAGGTCGGGATCTGACGGCGAAGTCGCTGCGCCGACATCGTTGTGGCAGCCCCTCAGCCCTGCG
CACGTCTTCTCCCCTACTCGCCCCATCCTCTAGAGTTCACAGGCCCAAAAAGGAAACACAATCGCTGTGG
ACAACTCGCGCCTCGTGCGCTGACCGAAGTTGGAAGAGACCCACGCGGTTCGCCTGGCGCTCTCTCTCTG
TACATGCACATGCACGTACTCTGCCCTCTCGTCATCGATAGTTCGAATCCGTCCTGTTGTCCCCTACCCC

```
TCTCCCTCTCACCTGACGCGCGCACGTGTCCATGCCGCCTCATTCCTTAG
>lmaj_09_5_up with T67301 30777, 31177
AGGTGCCTCAGGATACCGCCGGCGGGTGGCTGGTGGGAAGAGAGACCGACGAGGCATTGCTCTCGCTTTC
CTCTCACCCACGCTTCTCGCTCCGTCTGCAGTGAACCCTCGATGCCGAGCGAAGTGTGCTTCTTACTGTT
CCTGATCAGCAACCGCGTTTGCAGGTGCTGTCGAGACCCTTGCAGGCGCACCTGCAGGCACTGCCTGTCA
CCTCCTGACGACCTGAACCTCGCACTTGCCTTTATCGCTTGCGTTCTCCCGCGTGGGGTCTGCGCTGCAA
CAGAATGGAGTCGCTGCCCATTGTGCAACGCGCCCGTCTCGCCCCATCTCACCCACCCACTCTTCTCTCT
CTGTCCTTCCTCTCTCCATGTTGTGCGTGTGTGCTGCCTTCCTTCTTTAG
>lmaj_10_2_up with W88356 37897, 38297
GCATCACAGGGGATCTGCAAGGAGATGAATCGTATGGCTATGCTTTTTTCTGCATTCGAATTCGCCGAAT
ATCTGTTTCACACTCGCGATAATGTCCATGATCTGGGTAACTCCATCTAGTCTTGCTTACCTTGCTGTTT
TGATTTCGTTGAGTTTGCGGTGCATACTTCAGTTGCGAATGAACAAGGCAAGAAGAAGTCCGAAACACCC
GTTGAAGTTTGACCACGTGGTGTGTCGATCGCCTTTGTGTTCTGGGAGAACAGAAAGCCTGCTCTGTGGC
TCCACGCCACGGCAAATGCCAAAATACGGAGTCTGTGCATGTGTGCCTTTGTTGTGTTCATTCATTTCTA
TCATTTTATCCTGCGTTTTTCGGTTGTTTGCTTTCATACCTCCCTTTAAG
>lmaj_10_3_up with W88366 534400, 534801
AGAAAATCAGATTCATGAATCTTCTCAGTATGCGACCATCGGGCTCCTAGTGTTAAAAATTGGTAAACCT
ATCGAAATGTCGACGAAGAGCCGATCCTTTCGGCGAAGAAAAAGAACTCTGTTGTTCAGCACCTGGAATG
CACTCAAGCTACTATACTGAAAGAAAGGCTCCATGCTGCCTTCCAGCAGCCCTGCTTCCGCGAATCTAGC
CGTTTTCACACCCAGGGAGAGTTTTTGGTGAAAAATTTTCATTTCTGGTCTTCTCTGAGAGTTTTCCTTC
ATTCCGAGCTCTGGTCCTTTCTCTCTTTTTTCTTTTGCTTCGCTTTACTCCTCTGTTCTTCTGCATACAA
CGGACCTACACATCTACCATTAATTCAAAATACTCCCGCATTCATGCAAG
>lmaj_10_4_up with AA125640 76645, 77046
AGTTCTTCCTCCACACACACAGACACACACACTTTTTGCCTGTCTGCCACGACTATGGATCACCTGTGAC
TGACGGAAAAAGGGAGGGGGTGCGCGCCTCTACCTACTCGGGTGTAGTGCTGTTCGAGGTAGCAAGTGTA
GGGATGTCCGATCGGTATGCGCGTCCACGGAAATGGAAAACTCGCCAGCCTTGATGCGTTGGAATGATCT
CTTTCGACCACGTCAGGCACATATTTTGCGACATCACACACACTCTGCGTCTCCCCTGCTTCTATTATTT
TCCCTTTTGTTGTCGCACACGATGTGCGGGCTTGTGAGTGTGGCCTGCGCACGTGGGCCGTGTCTCGGTT
CTCCTCTCTCTTCGCCTGTCTGTGTATCGGTCTGCCATGCCGCTGCTCAG
>lmaj_10_6_up with W88345 551867, 552267
TCGCGCATTTCAACAGAAGCGAGTGACCCTGATGCGCCAGATCCATTCACTGGAGAGCCGAGAAGACTTA
CAAGACCTCTCTGATCAAAGCATGCCAGCTTAGATATGACGTGTTGTGCAACGGCACACCTCAAGCCGTG
CAGTTTGCCGAGAAACAAGGTGAGCCCATTTGCATTCAGCACTGTCCGCCACCAACTCCGAACAACTCGC
GCTCCTTACTGGCAACCCTTGTACTTTCTCCTGCCACCCCAGTCTCAACGTACGCTAGCAACTCCTCAGA
AAGACGAGAAGTCGGTACTGTGATAGTCCTGTCTAGGCGCTTCGAACAGTATCAGCGCTGGGTGTTCCTC
GTAGATGCCCCCTAGTCCATATAATCTTGTTTCTTTTTTTTTTGCTTCAG
>lmaj_10_7_up with T93381 113359, 113759
CTGGGCCCTGAGGATGCGACGCGCGGAGGGTTGAGTGTCTGCCCCCTTCACAGGAACACTGAGCGTTGAA
CACAGAAAGACATGTTGCCTGCGATAGCAGACGGCACCCGTGAAGCGCAGCGACAATGTTTCACGGGTGG
TGGAGCTGCGCGCGCGTGCGGGGAGGGTGGCCCGATGCAGGCGGCGATGACGTCACGTGAAAAGCACGGG
GACTGCGCAGCCACCCTTTTGCGGAGTCCTCCCTGAGCCGCTGGCGGTGGCGCCCCCTCCCCCCTCCCCC
TCTCCCTCCCTCACGCATCCGTGTGATGGTGCTGTTATGTAAGCAGAAAAAGGGCGCCACGCTCGTTCTT
CTGCCGCCGTGTGCAAATCCCCCACCGCCTCCCTCTCTACTCCTTCCAAG
>lmaj_10_8_up with AA060734 39114, 39514
CATAGGCACTCTGTGGCCAGACGACCTTGTCTACTATTTTATGACTCCTTCTTCGTTGCTTTGGAGATGA
```

TGCGAGCTCAACAAAGACAATTACGTGCTTTCACTCTATTGAAGAGCGTCGCGCCGTGGGGCGTATATGG
TTTTGCGTCATCGCCAAGAACTCTCAGCAGACGAAGAAAAAAAAAGGCACCTTCTGTGATGGCAAAGATGC
AGTGCGTTAGGCTCTATCATCCTCTGTATTTTCTTCATGACAGTTTTTTGTATATCCCTGTATTGAAGCT
CGAACGGGAGCCGCAGGCATCACTGGGATTACCTGCTTAGGAAAAGGTTGCATCTCTGGTGTTTTTTTTT
ACATCCAATCGAGTAGCGCTCTCATCTGTCCCGCTTTTCTCTCTCTCCAG
>lmaj_12_2_up with AA125638 378230, 378630
GTGTGTGTGCGTGTGTATGTGTGTGTGTGTGTGCGTGTGCGTGTGTGTGTGTGTGTGCGTGTGTAT
GTGTGTGTGTGCGTGTGTGTGTGTGTGCGTGTGTGTGTGTGTGTGTGCCGTGTTTCTGCATCCCCGAT
GACGTGCAGACACGCGCCTTCTTGATTTTTGTTGTCCTCGTCAAACAAAAGGCCGGCGCATGGCAGTCTA
CACTTCCAACTCTACGCAAGTCTGCTTCTTCCTGACCCTCCCTCTCTATCCACACATACTTCCATACGCA
CTCGGCATCTCGGCTCCCCACTACGCCGCTCGCTCGGTCTCCTCTGCGGCTGCGGCGCCTGATGTGATGC
TCGCTGGTTTCTTTGTTCTTGACCATCCGATCGGACTTTTTCCTCCGAAG
>lmaj_13_1_up with W88319 183240, 183641
GAGAAGGAGGGGCGGCCGGGCCCGCGGACACAAATGTGCAGGCGTGTGTGCGGCGTGCGCTCCTCTTCCG
TCCCCTCCCGCTCTGCTTCTCCTGCCCCTGTCGCCCCCTTCTACTTCCTCCGCACGGGCCTGCATTTCAC
TTCGACGTCCCAGCACACCGACACCAGCACCGACGCCCGCGGGTGCGCAGCTGTGCAGTCCTCTTTGTTC
CCTTCGCCGCTAATACCAACGTGCGTGTTTTCTGAATCCGTCTCCGCTGCTAATACTACTGCTACCTCGC
TCCTCTCCCCACCGCTACGTCCTCCTCTTCTCTTTTCCATATGCTGGGCGCATGTCTACTCTTGCCACAC
ACACACACACACACACACACACACACACATGCACGTCTTAACATGTACAG
>lmaj_13_4_up with W88394 176336, 176737
ATAAGACAACCGCTTGTAAGGCGGCAACATACCTGCCTGCCTGTGTGTGCGTGTGTGCGGGCTGTATCAG
CTGCGTATCTTTCTCTCTCGCTGCGAATAGAAGGCGCGCCAAACCAAGTGAAGTGAAGGCCGTGAGGCGT
CGCTCTGCTCGAGAGTGGCTGACCATTTTGTAACGCAGTCGTCGCGGTTGCTCGCCAAACACACTGTGTG
TGCTCACTCTCCCTTCCTCCACCCCCCCCCCCCCGTCGCTCCGTCACGGTCGAATGCGCGTGGACTTGT
GCGGGGTTGACCTCTGATCTACACGCTCTCATCCCTCTCCTTGCTGGCCTCCTTTCTCTCTCACGTGCAC
ACGCGCCCGTGTATACACACAAACCTGCACGGCTCAACGGCTCGGCGCAG
>lmaj_13_5_up with T67341 147624, 148025
GGGAAATGCCTGGTTATATATGTATATATGTATATATATATCTATATATGTGGCATGGTATTGTCAGAAT
AAGCAATTTTGAGGACTTTTTGGACGTGGAGCGCTAATTTGGTCACGTCTGAGTAGCAAAACAACAAAAG
TCACGATTTCTGTAAGGCGAAGAAATACTTTTAGTTGGAAAAGTCTTCCAACGGTCGAAATAAAAGTCTT
ACTTGAGCTGCGCGAGTCGCATTCGCCCTTTCTCATAAGATTTCCTCCTGAAACTGATTTTTCAGGAAAA
ATATGGATGGATGCTTGGCCTACTAAGCCTTTGAGTTTCATCATAATCATTGTTTCTCCCCATAGACCTT
CGAAACCCCCTTTTCCCCACTACCTCAACTCTTAGCTTTTTTTTTTACAG
>lmaj_13_6_up with T67348 511787, 512187
CGCGTCGTGTCACGATGCAACTCTATGTGGTGCTACACTGTGAGTACGGCTGAAAACTGAAAGCAGTACT
CTTTTCGCAAGTATCTGGGTATTGTGACACAGCGCTGAATACTATGCTGTTCCTTTTCCCGGAGTCAAAG
CGAAGTGTCAAAACAGGGTGACAACAGGCCCTTTGGACAGGGTTTTCACGGTCGGCTGGTGTCCACATCT
TCAACGTCCCCTATCGAATGCGTTGTGCCGCTGTTACAGGAATTGCTGCTTCGAAAACAACTCCGGAAAT
ATTTCATCCTCTTCGCGGTATCGTGTATGGGTAATGCGGAGATGAAGACTCGCATTGACTATTTACTGCT
CTCTTTATCATCTCTTTTCTCACTCCTCTCATTTCCCTTTTCCCACACAG
>lmaj_14_1_up with T93389 201804, 202204
ATACAACCACCCACAAACTTGCACACGCTCGCGGGAAGGGAGGGAAAAGAAAGGGGAAGGGAAGCGTCCC
GGATGCGCGAAGCTGTGGCGCAACGATACCTGAGCACCATGTGCGCAGCACGGGAGGAGGAGGAGGAGAT
CAGTTGTTACCGTGGGCGGTATGAGCGAGAACCCCAACAAACCACGGCAGCCAAAAGCGCACAAGAGTGA
CGACACAAATCGAAAGCCGTAGGCATGGGGCCACACACCCTTACTGCTGTTGCTGTTGTTGCTTCCGCGT

TGTCTGCGGTGCCTTCTCTCTGTTCCCTTCCCCTCTCCCTCCCTCCCTCCCTCTCTCTCTCTCTCTCT
TTCGCAAAACCCTTTTTGCTTCGCTTCTGTTGTAACGCGTAACCGTGAAG
>lmaj_14_2_up with T93358 450929, 451330
GTGTGTGTGGCACCTTCAGTGACGGCACGTCCCCCAAGAACGAAAAAAAGACGACAAGGGAGAGGCGACG
CACTTTCCCGACAATCGATGCCCAAACACCAACACCACGGGAGAGATGCGCTGACTGCTTGACGCCACCG
CTCTCCACTGCCACCGGGGTGTAGGCGTAGGCGTCGATACGGGGCGGGGCAGGGCAGGAGAGAGAAAGAG
GGGGAGGAGTAGTGGGCTGCACTTTCCCTTCATGTGATCTCCTATCATCACTTCTCGGTTTCGTTTTGTG
CCGTATGCACATGCGTATGGCGACTGGTGTGCGTGCCTGCCTGCGTGTGTATGTGTGCTGCACGGCCAGC
GCGAACCGCTATTTTTTCCCTACGCCGCCTGTGAACTCGTCTTGGTCAAG
>lmaj_14_3_up with AA060757 196160, 196560
GAGCAGGAGCAGACTGGATTTTGACCTCCTGTTTTGCTGCAGGAAATCGAAACGTTCAAAAGCACCGAAA
AAGAAAACCTTGCTCTTCTTCTACTACTTGAGAGGAGTGCTCGCCGGGTTCAGCTCGAATGAACTGAGGA
AGCTGAGCGTGCGGCCGGAAAGACTGACGCAAAAGACAAGGTGACCTAGGTTTCCAAAGGGCATTTCTGG
TTGACAGCAGCTACTTGCAAAGTTGTGCTTACGGTCGCGCTCCAGCGCAACAGCTGGTACCGCTGATGGA
GTCATAAGCGACAGGAGGACAACATGACAGTGTCGTACGCATGCCCTCAACAAAGCGTTCCCGTCTGCTT
CTGTTTTTGTGCTGTTGCTTTTGGCACCTCTACCTTTCTTCCCCTCTCAG
>lmaj_14_4_up with T93447 382955, 383355
AGGGCAAACACAGAAAGGGAAACAAAGAAGAGAAAAAGAAAAGCGTGACTGATATCCTTCGTGTGTGTGT
GTGTGTCTGTGTGTGTACGTGTGTGTGTGTGTGTACGTGTGTGTACGTGTGTGTGTGTCTGTGTGTGTAC
GTGTACGAGGAGATGGGCCCTTCATGGCCACCGCCTTTTCTCTCACCACCCATTACCTCTGCCGCCACCA
CCCCTCTTATCGACATATCTAACAGACACCTGTGCAGGCTAACGCACACCTTTCGCACCTTCTCTCTCTG
GTGTTGAGCGATCCTTCACCCGCTTTCAACGCTGTCACGCCGCCATCCTACTCCCTTCTGTTCTCTCTCT
CTCTCTTGTTGTTGCTTTTTGTTGGGGGGAGCGTCACTGCCCCTGTTTAG
>lmaj_14_5_up with T67309 353698, 354098
ACTGAGGAAAACGAAAAGAAGGCGAAGACGAACTCGCCCGGCTGTGAACAGCGCTGCTGCTGTCGCTACG
TGTTCTTTTCGCGTGAGTGTGAGCTGATCCACACATCATATGTGCGCACGGGCACACATGCGCAAGCAAT
CCGGCGAAATCGCGCCCCGAACTCAGCGGCGGTCATGGCTTTCGCGGGAGAATACGCGTTGCAAGGTGTC
AAAGAAGACGCCACCACGCTTCTGCTTCGCCTTCCTCGTCTCTACCCGCCTATCCCCCACTCCTCCACCC
ACCTTTCTGCTCGCACTCGCCTCTCCTTCTGGGTCTCCTTTTTTTCACGACGACGCTGCGTCACCGCTGC
CACGGCGTGTCTCGTTCTCCGACACACACGCACACATCCTATGCTGTAAG
>lmaj_15_1_up with T93474 64063, 64463
ACTCAACGGCACACGGGCATAGACATACGCGTACACGCATGACGTCACGCATGCCCCTCCCCGACGGCGT
CGTATCGTTTCGGTGGCACCGCCTGCACAACCCCCGCTCCCTTGCCCACCAGCTCCATGAGTAGAAGCGG
GTCTTCATGCTTTCCAGGATTACTCCAGCCGGCGCACGTTTTGGTGCCCCTGTCTTGTGTTAGTTTGTTT
TTTTTTAAGGGAGAGGGTGATGCTGTCAAGCCACGCTCATCTGCTTGTGGCTGCCCCCCCCCCTCCTCCT
CCTCCCCAAACACACACACACACACTCTCTCTGTGTTGAACCCAAATCTCCTCTTCCCATCCCTCCTTCC
TTTGTTTTCTTCTTTCTCTGCCTGCCTGCCTTCCTGCGGGCGTGCGTAAG
>lmaj_15_2_up with W88367 19986, 20386
CAAAACGATCCATTATGGAATTTCAGGAAGCGAGGAAGAGGGAGGCGTATCGTGGCAAGCGTGCTGTGCT
GACGCGGTATCTCTGCCCTTGCCACAGTGCGGCCAAGCGGACGCTGCTCTCTCGGACGAGTACACCGTCG
CTCGTGCCCACGGGCCTCGCAAGCATCACTATTGTGGGCGTTGTATCCTCACGTACACTTCACCTTGGCT
TCTTCATCCCACCTAAATGTGAGACATGGACACAATTTCCGACTCTGCATCCTCTCGTCGTGAGATAATC
TATCCATCGTCTGGTTCTCCCTCTATTGATGTCTGTCATCTCCCACTGCTTTCATACGCTCTCTCTATAC
GTGTGCGTACCGACTCCTCTTGTCACGAGTCCTCGTCCGATTATCTATAG
>lmaj_16_1_up with W88277 697815, 698215

CCGTGTACTGTAGCCGCGAGAGGACTTCTGCGACGACTTGCGCTCTGCAAGCGCTCTAGTGCGGCCATGT
TGGTGCGAGTGCATCGCCTTTCTCATAGTGGATGTCGTTACATCGTTGGTCACTCAGGGCATGTGCGGCT
GAGAAGGGTGAAGCCTGTGTGCGACAGCGCCGTTCCGGTCAACGTGCAGCGTGAGGGGAGTCAGACACGT
GCCGCCCGCAGCTCTGTTGGTGGTCCGTGTCGGCCTGCCTCCTCTCGCGAGGGCATTTCAACCCACGCCG
CCGGGTCTTTCTGCTCCACTGCCATTTTTCTTGTCGCCGTATCTCTGTTTTACGACGGTTCACGCTCTCT
GCCGACCACACGCCTACTACTCCACCTCGTTGGGTGCGTTCTTACACTAG
>lmaj_16_2_up with AA125650 345011, 345412
GTTGGAGCAATCTGCTAGTACACAGAAGGCCGACCAGGAACTCCCCAATCTTACTGTGCCTACCCATACC
CTTCCTCAAAGGGAATCGAAATTCAAGGGGAAAGAGGAGGTGAACGAGCAATACGAAGATGCTGTACCTG
CTCGTCAGTATGCTTAGTAAGCTACCCAGCTCTTGTTTTCAAGAAGCTACTCTGGATTAATATTGCGTGT
AGATGCAACAGGGAAACAAAACAAAGTCAAAGCTCGCTGCTCACCATGGGAAAGCCGTGGTATGAATTCA
ATTCCGCTCTCGGAAAGGTGAGAGTCATGGTTCTCAGCGCTACCGATTCGCCGTATGTGCACCTCTTGAT
TTTCTTCTATCTTTCCTTTCCCGCTTCCTTCTTTCTACTCTGCAACGAAG
>lmaj_16_3_up with W88267 129430, 129830
GTGGTTAACGGGCGCCGGGCACCCGCTCGAGAGAGCGCATGGCAAGGGCTGCGTTGCGCGCCTTCCTGTT
TCTCTCCCCGCCCTACGCCACGCTGCACCTCTCCCCTCCGCCCTCCTTCCGCGTCCGCCCCTTTCACAGA
GCCCGGGACTTGATCATAGTGTGCAGTGCATCAGAAGGGCAGCCCCCATCTGACTAGGGACAACAGCGGT
ACGCGGCTCTCGCCCCTCCGCCCCGCGGTACTTCCGATCTCCCAACCCCCACACCCCCTAACCGGCATTC
ACCGGAGCGCATGCGCGTCTTTGCTTCATCACACACACAGCGGCGCCGACCACTCCCCTTTGCCTCTCCT
CTCTTCCCTCCCTCCCTCCCTCATGCGCTGCTGCACTGCCCCACACGCAG
>lmaj_16_4_up with T93442 344051, 344452
TTTCTGCAGAGGGTGGTCGCTGCGAAGTAGGTCGCTTGTTGTATGTTCCACGCTGAAGGGGGAGGGTGAG
CGGGTGCCGGGGATGGCGTCAGGTATGAATCGTGGCGGGCTTGACTCTCAGAGTCCCATCTCGCTGGGTG
GAGGGTGTATGCAACCAGCGTCGCTGGAGGGTCATGCCTGAGAACTTTTAGAAGTCGCAACACGAAAGTG
CTAGTGGAGCCCAAAGACAGGCTCAGCTAGGACTGCTTTACAACCGGTACTGGTATTTGCAGGACATTGC
AGCAGCTGATAAAGCTCGCACTTCGTCTTGCGTGGAAAACTGGCCACCTACGTTTGCAGCTCCGCTTGAC
GATCGTTATGCTTTTCTCCTCCGTCTGTTTAATCTTCTCTCTCTTTGCAG
>lmaj_16_5_up with W88299 345653, 346054
TTGGACCTTCCTGGCATTGCGTTATTGGAGAGGGATTCGGATTTGAGATCACCTATGAGGTGAAGCACCT
GATGTACATGTTCCACAGGGGTTTCATTGCTATTGTTGTGTTCAAGGGCCTCTAAGGTGGTGTTTATAGC
ACTTTGACTTCTCATGTATACAGCTTTACGGAGGAAACGCAAGAAATTGAGCTGCTATCTCAGCGAGCCA
CGGCTACAATGACGTGGTTTATCTGCGCAGGTCGGAATCCTCAGCGCAAAAGGTGTCAGAGATATCATGG
ATGACAACTTGTCCTCCTTTCGATTTGCCAGTGCTTTCTCTATGTGTGTGTGTGTGCGAGCGGAGTATTC
GTGAGGCTATGCCGTCATTGTGCCCCCCACTCTCTCTTGTTGAGCGAAAG
>lmaj_16_6_up with T67305 421595, 421996
CATCTTCGCGCGCGCGTGCGTGCGTCTTCGTTTCTGTATCTGTATGCCGGTATTTGCCTAGAAGCAAAGA
ACGCGAGCGACACGGAGCACCAACACACATGCTCCATCATCTACAGTATGGGCAAGAGGAGAGGCGTGCA
TGAGTCCCTCCCCCCTCGTAGCGACGCACACAGGTGTACGCTTCTCTGACTCTCTCTCTCTCTCTCTCTC
CGTGCCTGTGTATGTGTCCGTGTGCGCGCACCATCGGACTCTGCGAAGGGGCAGCGTAATCGACTATCGT
GAGCCCCACCCCGCACTTCACTCACATCATGCACGGTTGCCCCACTCTGTCTTCCCTTTTCCCTATGCTC
TTCTCTGCGCCTCTTGCTGGATCTGTGCGTATTCCGACTACCTCCATAAG
>lmaj_17_1_up with W88350 665430, 665830
TCGTACTCAAGCGCAGCCACACATTGATGTGACAATGACAAACAGTTGAATAAAGTTAAAGTCCATACGA
GGGGTCGTGCATGTGGACATGCTCGTGCTTACCGCACCGCAGGGTCTCTCATGCAGGTCTTGTGAGAGAT
GGAAACTTGTATCTTTTTTGACTACGGCGCGCTTATGTGAGCGCCTGCGGTTTTACGGTGTCACTGTCAC

CGCACAGGGACCTCGCCAAGAGTACACCCGTCACCGTACTCCACCTGTGTCTAATGCGATTAGGCTTTCA
TGCCCTCGCTATCTGTCCCCTCTTTGTCACTACTAACCTTCTCCCATGCGTCACTCTCCGCTTCGTGGCT
TGCCTGTGTTTTCGAGGTCGTCTCCGAATTTGGCGGTTGACACGGCCCAG
>lmaj_18_1_up with T97244 376204, 376605
CGCTCAACTAAAAGCGCGTTGTGTGCGTGTGCGTGTGTGATCACGTCAGTATTGCCTGAGTCGATATGCG
ATGCTGCTGGCCGCGCGTGTGTCCGACGACGCCGTGTGCGCGTCTCGGCTGTCGCACACAGGCGAATGAC
TGTAACACATGCGGGGGCACCTCTCACGCAAAAAGAAGTCAACCCACGAAGAGGGCAGCGGCCACCGACA
CAATCAGACGTTGCGGTGCCGTCCTCACGCAGCCTCCGCTTCCACCTCATGAATGCGCGAGAGCGGCGCT
GCTCGTCTTGCCTCTGTTGCTGTTGCGTCGCTTCATGCTGTGATGTTCTCCCCGCTGTACCGGATCTCCG
TCTCACCCACACCCTCCCCTCTGTGTGCTTGCATTGACCCCGCGTTGCAG
>lmaj_18_2_up with W88272 691016, 691417
TCAGTACGCCTCGTAGCAGCGGTTGCACCACTCATTAGCGACAGGGAAGTGATAAGGGCGAGGGGTGTGA
AACGTGACTTTGTTTGTGGCTTTACCCCACACGCTGAATGCTGGGGTGAGCGAGAGAGGTAGCGCGACCT
ACGCGCTCTGTGTTAGCCTTTCCTATTGCGTCTGCTCTTGTCTGCAACTCGCCATGACCACAACGAACGC
TTCACCAGAAGGGCAGAGCGGAGGAAGGATGCTGCAGGATGGCGAAGAAGTAATCATCGCAGGGGCCGCA
GCCCTGCTCCAACAGGTCATCTTTAGGTGCCTCATGTGGGCACGTACACACATAACGCATCGCTCTTCCA
CGAGCCCTCCCTCTCTTCCTCCCTCTTCGGCTTGTTCCTCGATCGCCTAG
>lmaj_18_5_up with AA125622 326670, 327071
CCTCTCTCTCTTTGTGTGTGTGTGTGTGTGTGTGTCTCACAGTCGGTCGAGTGGCCGGCACCGGTTCTCGTC
ACGGCAGCTGCCTGCCCTTAAGCGCTCTTCCGCATCGCTCACCTCACCTCCTAGCTCCGCACTCGTCGTC
TTACACGCGTGAGGAAGAAGAGAGGAGCCACCACCGACATACCAACTGAGGTCCTTGCATGCGTCTCTCC
CGTGTTTGCACGCTGTTGTTGTTGCCGCTCACCTAAATTGTGCCTCATCCCTCCCTCTCCTGTGCGACCG
CCTTGTCGGTGGGCTGCCATCGACGCCGTGGGTTTTCACTCGCCCCCCCCCCTCCCGACCCTTCGCTCAC
GCACGCACACTTAAACGCATGCGGGCAAGCGCTGACAACCACGAGCGCAG
>lmaj_18_6_up with T67390 672428, 672829
CGCGAAAGGGCAATGCAGGCGTCGTGTTTGTGGTGCAGAGGCGAGGACCCAGTTCCACCGGGCACGCTCG
CGTCTGCCTACCTGGAGACGTGGCCCTGCGTATCGTGCATCCTCGGATGGCTTGATGGAGCACGAATTCC
GTAGCCGGAACGATGAAGGTGGGTGCACGACCTACTGAACGATTGCTTCCTCATGTGCGCTGACGAGGGA
GACGCCAACACACGCGCTCTGCCTGTGTTGGCGTCGCAGACACAGTTTCATGAAGACTGTGTTGCGGCAC
CAAATATTTTGCTGAGGCCCCTGCCCTATCCGGTGGTGAGTGGCGTGACCTCGCTCTGCTCCTCTTCGCC
TTCTCATCTGCGTTGTATACCCCCTGTCCTCCTCTCTTTTCTGCACGAAG
>lmaj_18_7_up with T93360 424682, 425083
ACACAGGCCCACGCGTGCCCCAGTGCAAGGCGGTCAAGCGTGCCATCCAGCCGCTGATGCAGCCTGGACG
GAAGTTGCGGCATATACCACGCTGGTCAAGAGGAAACCAAGCAGATCCATCTGCGCCTGTCCGTACACGC
GGTGTCGCACTTGGCGCGGCTGCTAAGGTGTGTCTTGGGTCCTGGCGCGCGCGGGGGAGGGGGGCAAGAA
AGGAAAGGGCGATGAAGTGCTGAGCGAAGCCGAGTGCACATCTCTCTTCCGCCTCTCCCTCTCACTCTAT
ATCCGTCTTTCCTCAACATCTGTCACGCATGGCATATGCGCCTCTCGCTGGACACCACCTCCAACCTTCT
CCTCCTTCGCTGGGACTTCTGTTGCATGTGATCGTCATGCATCTGTTGAG
>lmaj_18_8_up with T93417 705875, 706276
GCTCCCTTGATACCCTCACACCCGGGAGCTTCGTACGCCTCTCTCCCTTACACACTCCAGACGGCAGTAG
ACGCGCCAGTGACGCCCGCTGAATGACCGAAGCGCGTGCTTGTCCGTGGTGTCCTCGCACCGTCGCACTG
GGTGCTGTGCGTGGCGCCCCTCTCCAGTGTGTGAAGTGCCTCTCGCACGCGCTAAAACCTCTGTAGCTGC
GCGTTTCATTAGCCAAGACGGTGGCGGTGCTCGCCACAATCATGCCAAGGAGAGCGCTTGCGTTATTATG
TCGCCCCTAACCTCTTTTCATCCCCGTATCCGCTTCTCGACGGCCGCCCCTCTCCCCTCCCCTCCCCCCT
GCGTTGTCTGTTTCTGCGCCGATGTGCTTATGGTGTTTGGTCGCGGGAAG

>lmaj_18_9_up with AA060779 724632, 725033
AGCGAAAAGGAGAGAGCCCTCGGATGTACAGTTTTCTCCACGCCATGCCCTTGCAATCTCGACTGTAGA
TACCCCTTTGTGCTATAACATGCTGTGGCACTCATGGGCTCGAGTGAGTCCCTGCGCCGTGGGTTGCCGA
CACCGGTACTCAACACTCACCCACTCGCTCTTCACGAGTGCTGCTCCCACCCCTCCCCATCGCTCATCAT
GACCCTGCATCCATTATATCGACTCTCGCGTCACTCTGTTGTGGCCACATGCGCTGCACTCCACTCTCTG
CTATCCCTTTTACCTGCTCGCTATTTGCTCATCTTCACTGCTCACCACCCGCGCGGCCGCACACATGCAC
ATGCTCATTCACGCATACACGAGACCTCCTGCAACGCGTTCACTATCAAG
>lmaj_19_2_up with T67351 528513, 528913
GCGCACTGCAACTCGCGGGGTCGTCTCGTCAAGAGTTACGGCTCTTTCGCTTCCTTCTTTGGTTGCCAAC
ACCCCGCTCATGAAGAACGCTGAAGCGCCCCTTTGGGTGGAGAGGAGAGGAGAGGAGGAGGACTATACCG
ACTCCTGCGCGCTACAGCCGAAGCCCCTTTTCGCTCCTGTGCCCGCACGCGCACGCACGCGTCTGCGGCG
ATGATGTGGCGGACCCTGATTTTTCTCGCGTTTCTGCCGTCGCCTGCTTCTCCATCGATCACCACCACCA
CCACCGCCACCGCCACCGCCCTTCCTCCCTGCCTTTCATTCTGCCACCATTGGCTCTCTCGCTCGATCAC
ACTTTTCTCCTCACGACTACCGCAACCCTTCACCGCCCGTCGCCCCGCAG
>lmaj_19_3_up with W88352 333552, 333952
CAAAAATGGGAGTCGACTGTTGGCGTTGCAACGCCAAGGACCAACTCTCACATTTGAGGCGTGTCTTCTT
CCCTCAGAGGCCAGAAGAGGAGATGCGGAGAGCACGTTGTGTCAGTCTAGATGTATTCACGGCCTCTGCC
ATGAAGGCTTTCAGGAGGAAGGAGCATGCTCGCAGCAGCTTTGCATCCACGCACGCACACGCAGAGAGAC
CTTCTCTCCTCCTCGAGTTGTATTCGTTTCTGCCCTTGACCCGCTCCTCTTCCCTCTTCACTTCTCCTAT
TCTGTTCGCTTCCTTGCCACACACACACACACACACGCACACGCACACGCACACGCACGCTCCCACCCGG
CCCTCTCTTTCCTTGCATGCGTGCGCTCGTTACTTTGGTCAACGCGCAAG
>lmaj_19_4_up with T93399 395172, 395572
ATAAAACGAATCCCTATTTGCCATTTCCGCAAGACGCAGCAGCGACTGACCTGAACTGTGTGGCATGGAT
ATTACAAGCAATAGTGAAGCTTTTTTTATTTTGCGCAGTACAGAACAGAAATCGGCACTAATGTTTGTCA
CTTACTGCGCATAAGCAGAAGCTTACTTATTCGTAGACTGCTCACTACCAGACTGCTAGTGCATAATCCT
CCGAGAGTTTGAACTCCGCTACTTCTAGCCTCCAAGACCACGCGCAGATCTAGAGTCGCGGAGTGGGTTT
ACTACGCAGCTGTTGCTGAACCAACCCAGGGCTCTTCTCCCTCACGAGGCTTTGACTTTCTCATATTTTC
GCTTCTCCTCACTTCCCGCTCCATGCTTACCATTGTGCCAAACAAAAAAG
>lmaj_19_5_up with AA125613 640070, 640471
TGAAGTGAAAGCACCGATGAGTACATGTATGTATACATGTTCTGTCGCGTGCTAAAGTAGAGAAATAGAC
AAGCGAAAACGCCATTGTATAACCTTCATGGCTTTTAATCCCTGCCCTCTGCGCGTGTCATCACAAGGGT
TGCAAAGCCCCTTTTTTGCTTATAAGCTGATGTGGCCAAAAAAAAAATCAAGCGATTGCTTGTGTCATGT
TCCAGAGCACTATGTTGAGTACTAAAACAAGCTACTATGCGTTTGTCACCAACATCTTTCGCTGTTTCTG
TTCGCTATGTACAGGACAAGCGATACGCTGATGTATTCATCGTGTTCAATTCCTCTCATAACAGTATGAT
ACTCTCCGCCCTCACCTATCCTCGTGTCTTTTCATTTATTATGGGTCAAG
>lmaj_19_6_up with AA060759 646367, 646768
CGTGCCACTGTATCTGCTACAGTGTTCATAGATGTGGTGTGGAGCCGGGAAGATGCGAGCAACGCGCAGC
CACTCGGTCTCTTGATTATGGGGGAGGTGTGTCCATGCCGTCAGCTGAGTCCGCATGGCGTGCCTACGCC
CGCTGAACTGGTGAAAAGCGCTCTGCCCTCCAGTTGCAGTACCCCCCTTGCAACCCTGAACTTTACCATC
GCCGTCTCTCGTCGTTGGCTGGAACGCACGGAAAATGGAGTAGGCCATGAGATATTCTCATCCCCTCCCC
CTCTTCGATTGCGAAATGTGCGCATGGCATGATGCGTTGAACACTTCCAAGGCTTCCTCGTCTTGCCCAC
ACTCTTCTCTTCGTGCTTTTTTTGTCATGCCGAAATCGTGTGCATCAAAG
>lmaj_19_7_up with T93377 531794, 532194
ATGGCGTCTTGTGCATCCACGTCGCCCGCCCACCGACCCACCCCCACCAGAATATCGTGCGGCCGACGTG
GGATAGCAGTAGCCTTGAGGGCGCGTTGGTAGTGGATGATCGAAACCGTTGCAGCAGCGCGCCATCATCC

```
ACGGATAAGGGTGAGGGGAAGTAAATCACAGGGGTCGCGGGAGAGACAACAGCCGTTCTTTCATCTCTCG
TCCTTCCAACCCCGCCAAACACCCACGATCACGCTCGCATGCTGTCCTGAGTTGTCTTTCTGCTTCGCTT
GTTGTGCCTCTTACCGTCTCTTCCTGGTTTTCGGCCCTCGCACCCACTCACTTCCGTCCACCCTGGGCAC
ACGCCCGCGCGATCGCGCTTGAAATGCATCGCCAACGACACCACCGCTAG
>lmaj_19_8_up with W88292 408705, 409105
GTATATCTGTGTGTCTTCGTCAGTGGGAAGCTCCCCCCCCTCTCATGCATGCCGACATGCCCCGGCATCC
TTCACTCCCCCCTCCCCTCCTACTCCTTTCCACACCTGCCCTTCTCACTTTCACCTGTTTTGTGTTTCTG
TTTCTCCTGTCGTGTGACTATCCGACTCTCTATCTCCCCCCCCCAAACACACACACACACATACGCACGC
ACACGCAACTCTCTCTCTCTCTCTCTCGTTGCTCTCCGTATGTCACGTGTGGCTGTCCCTTTGGTGGGGT
GTGCGCCTTCATTGGTTTCTGTACTCTTTTTTTCTGCTCGGGTGTGTTCGTGTGGTCCTTCTCTCACACC
CTCACATCCTTTCGAGTCAAACACGTCCCTCTTCCCCCTCCTGTCGCAAG
>lmaj_20_1_up with W88320 29676, 30076
ACTGACGTACACTACAAGTCGTTCCGTTTCGCTTACTGTCTCTCCCCTCGGGTGTATATGCCTGTGTGCC
AGTCTCTCGTCCCCCACTCCACCCCTCCTCCCCTGTCTTTGCCCTGCTCACGTAAGTGGGACCACACGAA
TGCGCGTATTTGTGCGTGTGCTGGTGTTTTGTTGACGCGCATCAACGACATGAAGAGAGAGATGGGCCGA
TCGAAACGCTACGAACCTCTCGTCGATGCAGCCGTGTGGGAGTTCGTGAGGAAGCGCGGCGGATCAGCTT
TGTGTCTGTGTGACCCTCTAACACCCCGTCACTGCCCCCTCATCGCCATGATCGGCTGATGCGCCTCAGA
GCCTTCCATTCGTCCCTCCCTCTTCGGCCTCCTCAATCCTGTCTTGTCAG
>lmaj_20_3_up with T93364 112531, 112932
AGAGATGCTCACATCAAGGGCGGCGCCTCGCCGCCAGTGCTGGTGTGTTCCATCTGTGTGTAGGGCTTTC
GCGCCGTCGCGCATGTCTTAGGCACCTTGTGCGCTTCGCGTTACTCCCTGTCGAGGGAAACGCTGGGGCG
AGTGCGAGGAGCGAGGACGGATGCATCGGCGAAGTCTCACCGATGTCCAAAATCGAAGCACTTGTGGATG
TCGAGCTCTCTCCTCCTCCGTGCCCCCCTTCATCTCTTTGCGTCTTTCTTAGAACAGCAGCCACGCTGCA
CTCCCGCTCCTCACCCACCCGACCATCCCGCGGAAATCGTCCGCTGTCTGTGCAACTCTTACGACGCTCC
TTTTTCCTTTGCCCTATTTCTCTCTACTCACGTCCGTTCATCTACACGAG
>lmaj_20_4_up with T67338 615966, 616367
CCCACCCTTCTCCTCGTAGCATCCTTTACCGATAGGTGTGCGTGTTGCGGCGGCGGAAGTGGCGACTGAT
CAGGCGCCGAAAAAACAGAGATACCCGTGTGCGGTGCAGCTCCGTTCCTCGCGTGATGTTCACGAATGGT
CGTTGCTGCCGCCTCCGCTGTTCCACGTTGCCCATTGGCTTCGATACCTGCGCCCAATCGCTTCACTTCT
CCGTCTCTCTCTGTCTCCTGTATGGCAACTGATGCGCGTGACACTGCCCCCACCCCCCTTCCCGTGTTCC
CTTCCCTCGCATCTGAACTGTCCTTGTACTCGTGGCGCCTTGTCTTCTCCTCTTCTACTGCTCTGCTTTT
CTCTCGCTGCCCTCACCACACGTGTGTGCGTGTGTGGGTGTCTGATGAAG
>lmaj_20_5_up with T67395 281584, 281985
GAGCACGTCATGCGATGAGGTGTGGAGGGAGGGTACTGGTGGCGGTGGAAGAGGGGCAGTGAAACAAAAA
AACATGTCGCGCGCCGCCATTGGCGCGCACCCACATGCGCACCGCACCAAGAACGAATGCCCCGCTTCAC
CAGCCCTCCTTCATGGCGACAAGGACGGGGAACTTCTCCTCTTTCCACCTCCTTTGCCCTCCGCCCGTGA
ATGCCTTGCTGCCGTTGTCCCCATCCGCTGTTCAGCTGTCTCCAATCCACATGCGCTCTTATTGATCAAC
CCTCTCGCCTGCCCTCTCACCCACCCTTCGGCCACGACACACACACACACACACACACACCACCACAACA
ACAACAAAAACATGATCAACTACAACACCACATGAAAAAACGCCCCAAAG
>lmaj_21_10_up with W88399 433674, 434075
CCTTCGACCGGAGGTTGCACGGGGTGGGGGGGGGCTGTGCTTGGTTGGTTAGGCATCGCCTCATCTATTT
CCAGCGCACGGCGTGTGTGTGCGTGTGTGTGTGTGTGGGGGGGGTGCTAGCGCCATGCAGGGAGTTCTGC
CGGTGAGACTTGCCCACCTTCTGCCTCTCCTTGTCTGCTGCTATCTTTAACAACGCACTTACTCGTCGCC
TCTCTTTCCTCTGCCCTTTGCCTCCCGTGCGTCTTCTCTCGCTCGTCTGCACGCGCGTGATGTCTGCGGC
CTGGCCTCCCCAACTTGCGCCGCTCTCCGCATGCACGCCTTCTCATGTTCACACGCACATTATTCTGAAA
```

CTCCACCGGTAAACGCCCACACGACCCCCTTCCCTCCTCCAACGCGACAG
>lmaj_21_11_up with AA125611 70804, 71205
CTCGCACAGGACCCATTACCGAGAGCCGAGGCACGTCCCCTCGCCTTAGCATGTATGGGCGGGGCATCTC
AGCCCCACAATCATCGTGACGGCTCTTCAAGAGCGCCATATGCGCGGCGGTCTCGCGTTACTGCGGCATG
CCTCGCACGCGTGCAGAGATGGAGGTAAGCGCACGCACACGCACACGCACACAGAAGGCACCTACGCACA
TGCCATCGTACTACGGCGCCGTAGCGGTGGGGAGGACGCGCGCCGAGCCATGCGCGCGCACATGTTTTCA
CTCCACCGGCGGATTCTGTTTCTTGCTTAGGCGCCTCACCCCGCCCCCTGGCGGCATGCCCGCACTCGCC
TAACTCGATCCGCCTTTTCTCTGCCACATGCTCCGTCAACAATGTCACAG
>lmaj_21_12_up with T93400 10062, 10463
TCATGGACTCTGCCCCCCCCACCCCACCCCACCCCCTGTTTTGTATTTCGTGTCGACGCTGTTGTTTCTC
ACCGCAAAAGGTTTCTACAGGGGCTTTTCAGCGGCATTGAGGAATACTGCTGAAGTGCAGCTGCATGGTT
ATAGAAAGCTAAGAAAAGTGAAGGTTGCCTCTGTAACTCTGCGAGCATTTGCAAAAAGTGCATGGATGTG
CGAATGAAGGATTTTCCTTTCCGATGTAGCTCTTTTGAAGAAAAGCAGCTGCAAAGCCACAAGACAGTGG
TCCGAGCTGAGAAATCAACATGATGGTCCCCTCTGCATCCACAAATGATCTTCTTCTTCGATGCGCCTTT
CTGCATTCATCGCACCCGCCTGGTGTTCATCACTGAATTGATGGGGAAAG
>lmaj_21_13_up with T93452 234026, 234427
GGGAAGACACTCTCGCGGTCCTTGTAGACTAGCGACGATGTAACGCGGAAGTGGATCAGATGGCGAAAGA
GAAGGGGGAGAGCACTTCCCGACCATTGAGGCGAAATAACAGCCTTGATCTCACATGGAATGAAAAGTGG
TCGTTTGGGATAAGTGCGCAGTATCCCAATTTGTTTTCTCCTCCGCCTTTTTCAAATCCTTTTCATCTCG
TTACTGCCTCCACACGCTTTGCTTTCTCTGCGACGCGCTGCACATAATGCGACTCCCACACACGCCCACC
CGCATTCCCACTCACACACAAACATAACCGTATACGCGTCAACACCAATTCTGTCGGTGAAACGACGACT
GCCTGTACTTCTCGTCTCCCCTTCCGTGATATTCGCCTCGACACGCGCAG
>lmaj_21_1_up with T93405 343933, 344334
TCTTCGATGAGTTGTTCATCGCTCTGTCTGCGTCTGTGGACGTATGCGTGCTTGTGTGCGAGTCGGTGAG
GCTGTGGGAGCTTCACCGTCTGCCACACACGTTTTCCATGCATCTCTTTCTCGCACACCTCCGGGGCACG
GAGGTGGTGTTGGCGGAGCAGGGGAACTGGTCGCTTGCCACCGGCATCTTCCTCCCTACCACTCATGTGC
TGTTTTTCCCCGCCCCCACCCCACTCAACACGGCGGCTGAACTTTCCACGCATCTGACGTTCATCTCCTT
GTTTGTGCTCTCTGAAGCACTTCCTTGCGCATGCGTTCACTTGTTTTGTCTTCATGCACGCTTCCCCCTT
GACCGCGTGTGCCTCGCCTGCGGTGACATCCTGCCTCCATCGCCACCCAG
>lmaj_21_2_up with T93427 725925, 726326
CATTACAGTGAGTTGCAAACTGGCACCTCTTCCAAAGTAATGAGCAGAGGATGGGGAGGGGGGGGGGAAG
AACTGGTCATAACCTCATGCGTGGCAGTCGGACTCCTTTCCAGTTGTGTCACAGCAAAGTCCAGAGGAGG
GGGCAGGAACGCCTACAATCAGGTAATCTTTCCTCTGTGCATCTCGTTCCTCGCAGTTCTTGAGTCACTT
AACAATCAAAGCCTTCGATCTCGATTTCCCAGCATGCGGTGAGACGAGGCGCCTCGGCTGTGCAAGTACA
GAAACTCGAGACCCAGTGTCAAGCTCCTTTTTTCAACCTTCATGGCACCATCTCGACCTCCTGCTTACAT
GCGCACAATCTTCTTTCTCTCCGCTACATACCTGCGGTGGCAACTCAGAG
>lmaj_21_4_up with T67334 365551, 365952
CCTTCTGTTTTTTGTTTTTGACTGTGTTTCGGCTTCTCGCTTCCTGCTGCGCCACCGCTGCTTCCATGAA
CCCCTCCCCCCACTTGCCGTTGCTCGGGTGACGGTCATCTATGCCTGAGAGACATCATCCCCTGACAGTG
ACCCCACCCTCCCCCCGTCTTGGCCTCGCCGGATGCCGCTCCACACAGGCACCCACGCGCAGTCGCCTCG
ACCCTCCCCTTTCCTGCTGGTCGTATGGGAGAGACAGGCGCAAAAGGGAGAGGGGAGCGGCGTACAGCTG
TGCATGCCTCAAGGCAGCGAGGGAGGGCGACAGCCGTGCCGCCGCCGCCTCCTCCCTGCTCACCGGAGGT
GTGCACTCGTTCATCTCCCGCCTCCCTCCCCTTCGTCTTCGTCTTTGTAG
>lmaj_21_5_up with T93426 35820, 36221
TCCTTACTAAAAGAGGATGGAATGCACAAGTGGCAATATACCCACGCTTGTTAACCTCCCCATCTCACTC

TGCCACTGAAGACGCGCTGCTGAGATGCGCAAAGGTGGGAAAGAGAAAGGTGGCGGCGATGTGCGTGTGC
GCATCTGCGGTATTGCCGACTGTCGGCTGCAAGAGCTACAGTGCGCTCAAGCCGGTGGCTATACCTCTGT
TCTCGCTCCTCTGCTTCTCCATACACATGCTGTCACGCCATGTCCGGCGGCTCCCCTCCCCTTCTCCCCC
TTCAGCATCTTGCATTTTTCCCTCTTACACCTTCACCTGTCTCTCTCTCTCTCTCTCTCGAAACCCCCCT
CCTCGCTCTCTCGATTTATTTTCCCTTCTTCGATGCGCACGACGCGGCAG
>lmaj_21_7_up with T93425 224548, 224949
CGTAGGGCGATGAGTGAACAATCGTGGTTGAAACACGAACTCTTCCCTCGTTCGCTGCTGCTGAGACTCT
TTCAGTTGCAAAACCGATTCACAAAGAAATCGACAAGCAGATGCGCAGACATCGCACCCTTGAAGCGTTC
CCCGCGCCGAGTGCGACTTCTTCCTTTACAACATCAAACATGAGTTCGCGGACGGTGTCAGCTCACCGCC
ATGGACAGCCGGAGGCGTAATCAAAAGGACCCGAATGAATCTAGCAGTCAACTGTGAGCTCCTTGTACCT
TGTACTCACCACTTTCCTCGATAAGCTATTCGTAGCAATTACATGACACCCACCTTGTGTTCATGCACGC
CACCTACTTGCAAAGGGTCACTCGACATTTTTGCGAGGATAAAGGGAAAG
>lmaj_21_8_up with W88261 41082, 41482
ACTGGAAAGGAGTCCGACTGCCACGCATGAGGTTATGACCAGTTCTTCCCCCCCCCCTCCCCATCCTCTG
CTCATTACTTTGGAAGAGGTGCCAGTTTGCAACTCACTGTAATGAGCTGCGGCCCTCATGTGGACTCATT
GCGAGACTTACTTATTCCATTTCTGCGTCTCACATGACCAACCAAGATGTTTGCCGTCCTCCTGAAGTAC
TTCGCACAGAACCTCAAGAAATCGGGAGGAAGCTCGTGCGCATGCTCCAGATGCTGCGAATTTATCCTTG
CGAAAGCCACGATGTGGTCACGCGCGCGTCATACTAGCTATTGGTGGTGCCCCTTCCGAAGACATTGATC
CCTAGAGCCAATAAACCTTTCGCCTTTCTCATCCCTGCATCTCTTTGTAG
>lmaj_21_9_up with AA125634 226660, 227061
GTGTCGGCAAGGAGAAGCGTGCCTTGAAGTACTGCAAGAAGCGTCTTGGCGATTTCACTGCGGCCAAGAA
GAAGCGCTCGAAGATGGAGGAGGCGCTGCGTCACGCGACCAAGAAGCACCATTAAAGTATTCGGCAGCTG
TCATCACTTTTTTTTCTACTTTGGCAAAACATCAAGCGAGAACATAAGAAAAAAAAATAACCAAACACCAA
TACAAGGATTAAAAAGTTGTGCTATCACAGCGACGAACTGCACCTTTTCCGCGCGAGCTACTCCTGTGCT
CTGTTGCCTTCTCTTTTTTTGACGATGCCTGTGATATCGATTGATGCTCTACTATTCCTTCTGCACTATC
ATCTCTGTTTTCTCTTGAACCGCTGCACTACTCATCTGGGTGACATGAAG
>lmaj_22_1_up with AA125626 123150, 123550
GACGCGTGGTCCGATTGAATAGCAGGATGGTTTCTCCAAGAAGAGCACGATTTAGTTATATTCCAAGAGA
GGTCGGTAAGGTGTGTTTGTGCGTGTTGGCTTGTCGACTTGTGTATTGGAAAATAATGCGTTAACAAAGA
AAGATTTCTCGCTCCTCGGCGTTTGATCGAGGTGCAGATGATGGCAAAGTAGCTTAGTCTCTAGCCTCTC
TTGCCCCCTTCACAGCCGGCCAATCATGTGTGCGCGTGCATGCGCTGTATGAGCACGACCCCCCCCCCCC
CCCCCCAAAAAAAAAACAAAATGTACGCTATGGTCCACTTCTTCTTTCCCTTTTCCCTTTATTTTCTAAA
CCTGTTGCCGTGTCTCCATTCGCTCTGATCACGACCGTCGCCGAACACAG
>lmaj_22_2_up with W88317 448939, 449340
CTCAGCGCTCCAATCCACCGCCGCACAAAACTCACGATCTTGAACGCTGCTCGCCACGCCAATAATTTGC
AATCCTTTTCCTTGCTGTGTATTTCGTTGCTGCCGTGGTGTTCACGCATGCGCGACGAGGTTGTTGCTGT
TATTATTACTGTTGTGAGCACCCCCATGCACTTGTACTCGAGACGCGCACGCGCGCGAAAGCCTTGTCCC
CGCCCCGCCAACATGGACCCATCTTTGGTTGTCTTTGGCGGTGTCTATTATCGCCCTCTCCACTGCTGCC
CTTCGTCCCCTTTCTCTCTCTCTCTCTCTCTGTGTCTCTGTCTGTCCTCATGATCTTATCGAAATTGCTTGC
CTGCTCGCACGTGTGGTTGCACCTGGGTCTGTGTGTCTCTTGGTGAGTAG
>lmaj_22_4_up with T67353 161708, 162108
AGACGGAAGCGACAACCGTACGAGGAAAGACAAGGTGCGAAACGCAGCAGCCTTCACATGGCAGGACGAC
CGCACAGCTGCTCTGCACACGCCAAGGCTGGATATGAACGAAACAAAGCCACACAGAGGCAACGACAGCA
ACGCAGCTGCACCACATCGATGGGTGTCTCCGTCTCTCCACCGCTTCCCCTCCTGCTCCCCACACTCGCT
GCGTGCACGGCGCGGCCGCCCGCGGTGCGTCATAGTGGAGTCGAGCTACACATGCCCTAGCCTTGTTTCA

CTGCTTCTTCTCGTCATCTGCCGCACAGCCACGCCACTTGCGATCCCCCTCCCCCTCCCGCTGCTGCTTC
TCCCCACCACGACTCCTGTGTACTTCTCTTCATGCCTTTTCTCGACCAAG
>lmaj_22_5_up with T67365 672810, 673211
TTACACCCCATCCCACTGCTTATACATCGCAGCAGGGATCTTTCGGGTGGCGGAAAAGGTAAGGAGGGGA
GGGGGGAAGCTGCTACGTAAGTCTGCCTATCCGTGCGTAGGGTTAGCCGATGGGGACTTGAAAACAACAG
AATTCTCAAGGATGGCTTTGCCAGCGCCAGGAGGTATCGAGAGCGCAAGTGGAGGGATACCAGGTTGGTA
TACTCACCCACAAGAATAGGAAACTCGCCAGCCAGGATGGGTTTGGGCACATGTGTTGGAATGCATCATG
CTCATCGTTTTTGACATCATTCTTTGTCTCTCCTTGTCTTTCTGCCTTTTGGGTGTATGCCTTCGTTGTT
GCCGATGCTCTCCTCGTGCTCCCTGCTTGCTCTCCATTCCCGTGGCACAG
>lmaj_24_2_up with W88372 29600, 30000
TAAACGAAAGACGCCCCCCTCACCTCACTGTTTTGTTGGTGCCGCTTATGTTTTGGCGCGTCTCCTAGAA
CGCACACGATTTGTGAGGGGTTCAGGAGTGTGGCGTTCATGACGGAGGGAGCTGAGCATCCACACGAAAA
GAAAACAAACAGGAGAGTCTGCTACTGTGTGGAGCAAGGACTGGGCAGGCTCTAAGTACCTTGCTGCGAT
CCCCTTCTGCCTCCCAGACGGCCCTGTCTGAGGCTCCTCGACCGTTATGGCGATGAACTAATTCTGCGAT
GCAAACTTTTCTGTCTGGTGCACTCCTCGCTGTACTTCTTGTACTTCGGTGAACGCTACAATCTACACGG
GTGTTGTGCGAGGCGGTCGCTGCTCTCTCTCGCATACGAGGCGGTGGAAG
>lmaj_24_4_up with AA125647 59385, 59785
CGTGTGGCCGTCGCCAGCGGCACGTACACCACGCGAAGGCCCTCCGGAGGCGCAAGGGCGGTAAACAACG
AAAGCTGTGCGATCCCTCTCTTGGAGTGGCCTGTCTGCGTGCCTGCGGGGCTCGAGGGCGCAATGAGCAT
CGCGTTTTCGCGCTACCGCATTCGCTCTCGCTCGCTCGCTCTCTCCGTCCTGTGTACGGCGTGCGACATC
TCGCCGGTTACGCGTCCCCGCCGCTGGCTTGCGATGTCGCCTACATCACTCTCTAGATGGTCAAGAGCAT
CCTAAGGCCCTCTTTCTCCTTTGTGCAGCTCTGTCCATGCCGGTGATACACCCTCACGCCTTCTTTCTTT
TCGCCCACCCGCCCTCGCCCTCTCCCTTCCCTACCTTCCACGCCTATCAG
>lmaj_24_5_up with W88296 335766, 336167
GCACGCGTGTCACAGGGGAGGGAAGGGAGCGTGGCAGCGAGCTCGTGCACACGGGTGCGCATGGAGGTAA
GCCACGGCTGTCCTCCCAGCGTGGTACTCATGGTTGTAAGCTATGCAAGACCCTTTACGAGCGCGGAAGC
GCTGACCGCCTTCACGCCACGCCTTTGGCGTGTTGCGCGTGCATGGGTGTATACGTGCACATTTCGATGC
GCCGTGACCGCAGGACGTGCGAAGCCAACGCATCCCCTCACGCGTCATGCAACGCCGCTGCCCCTCCCAC
CCACCCACCCCCCCCCTCCTCTGTCCCGACACACACGACCTTCAACTGCCCCGATGCCGGCTTTTATCGT
TTCTCTCTCCTCTTCTTCACGCCACGCTCGCCTTGGTTACATCCGCGTAG
>lmaj_25_10_up with AA060769 863601, 864002
TTTCTCGAAATTCTACTCGTCGGCGATGTGCTGCATGGACTCGCACCGCGGCAGGCAGAGGCCGAACGCC
GATTGCATCTGCTGGTTCGTGTCTTCATCGCATCTGGGTTTGTGGTGATGCACAGCGGCCTTGAGAGCCA
CTGGCGGTCGGTGCATCCACTCTGCCTCTCTCCCACTCTCCGCCCACTCCTTTGCACCATTCTTCTCTTC
CTCCTTTCATCCCTCTGTTTCCCTCTCTCGCCTTCCTTCACGCGCCTTCTTGGTCTCGCTCTACCTCTGC
GGCTGCTGCTTCTGCGACTTCCCAATGTGGCCCGTGGCGGACGCGGCTTCTACTCCTGCTGCGCCACACA
CTCTCTCTGCCCGTGCACGGTAACTTTGCGTGTGCACGTGGCCACTAAAG
>lmaj_25_1_up with T93382 831175, 831576
CTTTATAGGCACCACAGACATCATAAGCGGAAATCGCATGATTCGATCGTTGCTGCCGTTTGAACCGGCT
CGGTGAAGGCGTTCTCTCTCTCTCTCTCTCTCTGCGCTCCGCTTCCCGTTTGTTTGCTCCCTTTTCCTCC
TGGCGTGTGTCAGCAACAGCTTCGCGATAGCAGGCAGAACAACAGAACTTTGCTTTGTCGTCTCACATGC
TATCTGAAAGCGCCAGCCTCGCTTATTAGGCAAATCTGGTGAGATAAGCAGCGTGTCACACCTACGCCCT
GCGCCTGCGACGATGCTCCCATGCTTGATTTTGATGCAGTGGTCAACTGTGCCTGTTTTCGTCTTCTTTC
CCACTCCCTCTTCGTCTTTCTCACGTATAATGCGCATTTTTTGCTTGCAG
>lmaj_25_2_up with T93470 104826, 105226

TCCTTTTTCGAACGGAGTCGCCTTTCATGTATAGCGTGCGTGTGTGCTCTGTGCCTGATGCCGCCGTCTG
CAGTGACGCACAGCCAACTACACCCACCGCTGTGCGCATCTCGCTCCCTTGCCTACAGACAGTGGCAGAC
GTGGGTTGTTGTGCCGCGCCACTTTCCCCTCCTCCTCTCCTCCCACCCCTTGCACCTCTCCTCCCTTTCT
CCCATGGTGTCTGTATGGGCACTCTCCCGCCGCCGCTGCTTGTGCTGCCGCTGGCGCCCTTGTGCAACAC
ATCCCCCATTCACACACACACACGCACTGCCTTCTCCTCTCAACACACCAACGCGCGCACACGCTGGCGC
CCTCTCCTCCTTCCCAAAACACGTCTCTCTCCCATACCGACGCCTCACAG
>lmaj_25_5_up with W88406 891003, 891404
TTCTCTTGTTCTCTTTCACTCCTTCACGGTCGCTCCCCCCACCTGGCGCCCGCCTTCCACGCCTTTTCTC
CTCTCCAAAAAAAAAACGCAGGAGAGGGGATGTGTGTTCCACAGATGATCTCCCTCTTTCTGTATTGCTG
ATGATGGCCCTGCTGCTGGTGCTGCCCTTCTCTATTCCTCTCTTCCATTGTTACACCACTCACGGCATCG
CGCTGCCGTGGGGTAGCGACGCGGCAGCACTGCTGGTGCTTGTACGTGCTGCCATCGTTCTTTGGCAGAC
GAGAGCTTCGCCGATACACCAGCCTCCACTTCCGCTATGTCTATCCTATTTTCTCGCTTCCACTTTTTCT
GACTCTATACGTCGGCGCTACACCGCACATTATCACCCACCACGCTGCAG
>lmaj_25_8_up with T93473 829026, 829427
TTGCTTGTCGGGACTGTCCGGTGGATTACCAGCACGTAAGAAGGAAATGTAGGTATCGAGTCGAATTCAC
ATCCCACGTTGAGAGCGACAAGCGGACGCAACATAGCAATTACAGCTCGTGATCACACACACATGCACAG
CGTATGTGAGCATCGCTTTTTCGTCTTTCTCTTGAAGGTTAGGCATGCTGCACCAGGTATCCGCTATGCA
CAGCTGTGGCATCGTTGAGGGCGCCACGTGTCCTGCGTGCCGGCTCTTGTTGCTCTTTTTCCCCCTCTTG
AGCTGCGTGGGCTCTCTTCAATGGTGCCTTCAGCTAGCGTCTTATCTCCCATCATTTCTCTCTCTGTAAC
AATGTCATTCGTTCTGTCTGAGCTGGAATTGATTCTTTCTGGCACGCCAG
>lmaj_25_9_up with T93448 837957, 838357
TGCAGCAGCGAGAACTGAACCACAGCAGTGCTTCATGTGGCGCACAAGGGCTGAGCAGCAGCAGCTCTTG
CTCTGCCACGCAAGAAGTGCGAAGAAGAAAGCTAGAGGAAGCGGTGCGCGGCGGGGCTGAGTCGGGAGCG
TGTGCCTCTCCGCTCTCACCTCGTACCGCTTCCTCTCTCTCCAAGCGCTCCACATGTATCTGCGCTCCGC
GCTCCTTCTCTCCAGGATTGTATCGACTCCTGCCTCCGCGTCCGTGTTTATGCCTCCTGCTAGCACAACC
CTCCCTTCCCCCTCCACACACACACACACACACACACACACTGCTGCTTGTCATCTTTCGATTGTTTCGT
CGTTGTCTCTCACCTTGTGCTGAGCGCATCATTACTGGAGGTCACATCAG
>lmaj_26_1_up with W88337 953017, 953417
TCCGGCTTGTATCCTTTGTCCATTATACATGCGCACGCGCGCGTGTCTCCTTGTCTGGATGGGTATTTGT
TCTCTGGCTTATTCCTATGTTTCAGCGTGCGCCTCTGCTGTCTTTCCCCAAACAAACTGTTGGGACGGTG
CACTCGAGACACTCGTAAGCACCACAGGCCAACCAACCCCCCTCCCTCCCTCCCATTCCCCCGTGATGGT
GCGGATTCCGCTAAAGCGACCGCTCATCCGCACGTCATCTCAATGCACCTTCTGCTGCAACGCATCGCGT
GGTCAGCAGCTCGAGCACCTACGAGAACAGCTTGAGCGACGTCACTCCTTGTCGCTTGTCTTTGCGCCCC
GCCTCACTCCTACCCCTCTCTCTACCTCCGACTGCCCCCCTCCCGCTCAG
>lmaj_26_2_up with W88411 630026, 630427
CTCCTCACTTCTCCCTCCTCCTTTCCCCCTCGTTCTTCTCTTCTTTCTGTTCGCTGTATGTGTGTTTGTG
TCCGGGGCCGTCTGGCGCCGATCAAGCAGCATGTATCCGAAAAGGGCTCGAGGAAGGACAAGAAGCTCCT
GTTTTATGCGTGGCCCAATTCAGCTTCAGGCGGTTCGTCCAAGGCGACTAGGATTTCGCCGTGGTGTAAC
AGACGTATGTGAACTGTGGCTGTGAGCTTCTATTTGCTGACGTGTTCGTGGCCAAGTGCCACATCGCGAG
AAGTATGGGTGTGTCTGGTATTCTAGATGCCGGGACATTGTGTTTTGGAGTGTGTTTCCTCTGTCTATAT
GTCCTGTCCCTGTGTTTCCGCTCCTTCTCCTTGCTCTTTCTTCCGCCAAG
>lmaj_26_3_up with W88398 902494, 902894
CTGTTTCGCATGTGGGTTAACGCATCCAGCCTAACGCGGCAAGTCACAAGGCATACGGATGCACAGCTAA
TAATGGTGGGCCTCACGGACATGCCGCCTCGCTAAGTGCAGTTGCGAAATGCAGGCTGCATGTTTCTCTT
CCCCTCCGGATTTCTCGACGCACCCGGCTGTGTCGCATCTCCAATGGAGGGCGTCTCGCCCGCTGCTCTC

ATGCGCGTGCACCCTTTTCGATTGCTCCTCGCCCTTCTCCTACGAACATTTCCTCTTCTTCGTGTGCTCT
CTCCCTGGTCGCACGAGTACGCACGCACGCACACCTGAAATCAAGCTGCCCTGCTCCCCCTTCTTCCTTC
TCTTTTCCTCTGCTGTCGATTCGAGTGCCGGGGAAGATTCACCGTGACAG
>lmaj_26_4_up with H64198 934489, 934890
TGGGCTAGTGCGCGTTACCGCTCTCGCCGCCTTCACCGCTAGGGAATGGACTCGTCTCCCCACCGTGCAC
GCTCTCCCGCCCTGTATCTGTACGCGTGTATGCGTGTGCGCGTGGTGCAACGATCATGTGGGTGACTCCA
TCACTATCCCCATGCGATCTCTCTGTTTTTAGTGTACTGAATGTCTTCTCTCTGCCTCTTTCTCTATCCT
AACGTGCGCCATCTCACCCGCCATTCTCCTCCGCCCCCTTACCCCCTACGGCCCTACCCACCACCTTCTT
CTGATACATCACATCCATACATGTGCGTCAACGTGTGTACGCGCATCGGTGTCGTCTGCTCGTGCATATA
TGTGCTTCCCGCCACCGCCACCACCTCTTTCTCCTCCTGCGACGTTACAG
>lmaj_26_5_up with T93469 786062, 786462
CGGTAACGAAGACTGCGTGTTCTCTGGGTGAGGGTCAAGGAAACTCAGGGTCTACTTCTGACCTGTGTTA
TTTTCTTCCAATTACTTCATTTCACTCATTGTATTTGGTCGCATACAAGAAGCAAAAATCCTCTATAGGA
TGACTTCCGCGCGTGCGGCGGGTACCGGTATGTCTCAGTGCGCTACAAAAACTAGGCTAGCTGTCTTACC
ACCGTTATATGTGCGCTAGCAGAACATTAGCGTTTTAGCCTCAGCTTGGTCGCTGCCCCAAATGGTTGCG
AGCAGTCTTTTTCCAGTGTCGTGGTGCAGCAGGTGCTTTCCATTCTCATTTTCCATTCTCTTTTTCTTCT
CTCTGCTTATCGTGGTGCCTTTATGCGGGGGCAAACCTACACGTGTAAAG
>lmaj_26_6_up with T93410 1028040, 1028441
CCCTTCCCCACCCTCCTTCTGCCTCTCGCATGCTGGTCCTTTCACACGTGTACGAGGAGCTGAAGGTGAA
GACCACGCGCAACGATTCGGCAGCAGACTGAAGAAGACGCGAAGCCGCCCCTCGCGGCCCACGGGCGCAG
AGGTCGAGAAGGGAGGTTGGTGGTGCGTCCTCGTACCAGGGCGCAAGGGAGGAACAGCAGGTGCTGGGCA
AAGGCGTAACGTTGCCAATCTAGTCAAGGCGCTTCCGTCTGTTTTTGACGAGAGCGTCGTAGCCTCCTGT
CATTCGCGATGAGGTTGTTGCACCCGCCAATGCCCGGCTGCACGTGGTCCATTGCTGCCGCGGAAGCGAT
CTCACGATGATCACTCAGTCCTCACCCGATCTCGATCCCATTTTCGGCAG
>lmaj_27_1_up with T67350 294481, 294881
ACATACACAGCGCATTCCATTGGCAATGACAGCACGGCGTGTGATGCCACAGCGGGGGGAGGGGGGGGT
GGTAGACGGCGGCACGAGAGGACGAGAGTGAGAGGTACGAAAAAAAACAAAGGATGCATCAACGACAGCC
GTGGCGGATGACTTCTGAAGAGGGAACGGCTGCTCCTCCCCCTCCCCTTCTTATCTCACCCCTTTGGCGA
TCTGAACCCGCTGTCTCCACACTGTCGCCTCCAGCCCTTCTCTTCCTTCCACCGATGTGCGTTGTGATTC
CGTCTTCTGTTCTTCTGACCATCCTTTCATCAGCTCCAATGCTCTCTAGTCTCTCAAACCCCTTGTCTTT
TCCCCGACCCAACCCCCTCCAAACACACATATACACACACACACACACAG
>lmaj_27_2_up with W88354 1070549, 1070950
TGTTTCTCTTCGGTGACGTCCACGTCGTGGATATGTCTGCGTGTCTGTCGGCGGCTGCCAGTGCGTGTGT
GTGTGTGTCGGTTGGGGTATCACTCCCGCTCGCATTCGTCATCGCCGTTTACTCCTCTATTGGTTCTCAT
TATTTTCACCCTCAGGCGACGCTCCGAGGCGTCGCAACGGCTGGTAGCACGGCGCGCAATGCCTCTAATG
GCCCACCATGCGTGGGATGCCTATGCGTGTGTCGGAAGAGCACGCCTCACCGATGCTCTTTCCCTGTCGC
CTACATCTCTCTCTCTCTCACACACACACACACACACGCACACACACACATCCTTCCCTTGTTTCTCTCG
CTCTTTTTACCTTACACACACACGCACACGCACACATGTGCACACGGAAG
>lmaj_27_3_up with AA060756 150362, 150762
CATCAGCGGGCAGCGCTGCGGATGCTCGTGCGCTCACACCCAGCACCACGACCCTCAGCCGATTGGCGCT
CCCAGAAATGAAACAAGAATCATTCTGTGATTGCCGTGTACTTCAACTTCGAGCGGCTACTACTATTCTT
GATCAAATCGCGGCTTGGCTCTCTGCTGCGTCTCAAAGCTAACACCGGATGAGCTGCAAACACACGATCT
TTTGGTACTGCATAGCGGCACTTGTTCGCAGCTGGTAACAAGGGGAGGCTGTCACTATATTCTTGATTAC
CCTTCTCGGCGGCATCTTTTTGTTCATGCGTCTTTGCCTAATCTGCCTCCTCTGCACCTTTTACCTAATA
CCCGCTTCTCCATCCACTCCTTGAAACGGTGCCTCTCTTTTTCCTGCTAG

>lmaj_27_4_up with T93414 535785, 536186
TGCTGCTTTCATACGATTCAGAGGTCGTGGCTTCACTTTATTGGAGGTTATAGGCTTGTAAACCGAGTCA
TCGCTCTTTGCATCTTAGCGACAGTGGGGCGACAGTGACTAGTTGGGCACACACACATCCATACACACAC
AGGCGAAGAAACGTTTTTTTTTGTTTGTGGTTATCTAGAAGCGGGTCGCGTATCTTAGTGTTGATGAGGA
ATGTGTCAGAACAAGCTTCACAATACGTGCAGCGTGTGGTGTTCTGTTGACCACATCTCTTTTACTGTGT
AGCTGCGAGATGCGCCTCGGTGGCGAGTTGCCGGTGTCTTTGGGGCACCTTACACTTTTCCCCTTCTTCT
CCTTTTTCACTTCTTGTCACTGTATCAGGAGCACGCGAAAACGAGTTCAG
>lmaj_27_5_up with W88278 298867, 299268
CTGCAGTAGCATGCAGTGTGTTTTTTTTTTCCGGAAACTGAAACGAACAAAGATGTGACGCATCTCAGCA
CTCATGACTCCCTGCGTGCACCAGACGAATGTGTGCATCTGCATCTGTGTGCTTGCGTGTGTCTCAAAGG
CGTGTGCCGCCCCACCGTCATCAAGCATGGCTTACAAGCTATTTCCATCGCATGTGCGTGCGGTCATAGA
CGTCCGCCCCCTCACGGCCGCTGCTAGTACATTAGCCTACTTGGGCGCCGCGTGTGCATCGCTTTGTGCC
CGTGCCATCTCGTTTGGTTCCTTCGCCACCTCATCTCCTTGCGTTGTGCTGTCAGCTCGACGGTTCCATC
TCCTTCTCCTCACATCGTCTGGGTTTCTCTGTCTTGCGCCCTCCGTGGAG
>lmaj_27_6_up with W88298 1059002, 1059402
CACCGCCTCGGCGTCCGATGATCTCTGTTCGTGCGCCGCGTGTCGACGCAGGCTCACTGCGATCACTGTT
CATTCGATTCTCTGTTTCGCGAGTATTTCGCTTGCGCGTGGTTTGCATCCCACTTTCTAAAGCAGAAATA
GCATACGCTCCTCAGCTTCTTTCTCCAGAGCTTGTACTGTTGAACCCTACTTGCAGTGTGGTGCTGTTTT
CCAGTAGCTGCTGTAAGGGGTGCTGAGACACTTCTCATGGCAATGTTGTGGAATAGTGAATCCGATGCAC
CTGTTTCGTATTCCTGCCAAGCTTGCTCTTCATCTCCTCATCTTCACTCTCCATTCCTCACCTTCCTTCC
GCTTACTTTGCGTCTGCCCTTGGTTAAAACACTGCTCTATAAACGACTAG
>lmaj_27_7_up with W88302 394794, 395194
CCGTCCCTATGCGTCGGACTCGTGACCTCTCGAGTGCTCTCCCTGTCTCTCTCTCTCTCCCCCTGCATCT
GCTTCTTGTTATTGTTCGCCTTGCACAGTCGCCGCCACATCAAGAAAAGTGGGCGAGAGAGAGAGAGGAA
AGCAGGGGTAACGAAAAACGAAAGGCAACGAAAGGAAGAGAGAAAATACCACAAGCGTCATGTGAGCGAT
CGCATGAAGGTGCGCAGGCGGGTGGGGATGCGACACCAGCGGTGATGCTGTTTTTTTTTTAGTGATGGAG
ACATTGTGCAGTGAACCCGAACCAAACAAAAGACGGCCGCTTGTGTTTTGTTCTTCCCTTGTGCTCCATC
ACACTGATGTCTCTGCGTTAGCTGGCCCTCCCTCTTCCTTTCCTCGTAAG
>lmaj_27_8_up with AA125624 83444, 83845
CTTGTCATTTTTCTCCTCTGGCCCGCGGCCCCGCGGCAAGGGCCGATATGCAGGCGCGCCGTCACCGAGA
CGTTCAGAGACATACTTCGAAGTTTTACTGGCGACTGCCAGTGGGCAGAAGCCATTCGCAATTTATACTT
GGGGTGCGTATCGTGTTCTTGGAGTATTACTAGGCTCCCTGCTGTTCCTGTTATTCCAACTCGGATGTCG
CCTGCCTTCAAGCACTGTCGAAGAGGTAGCTGACTTGTGAGATCACTAACCGCATGCAAAGACGGTCGAT
CCACATGATCCCGCCACACTCTTCTTTCCGAACCTCTTCATCCTCTTGTGTTTTCCGATTTCCTCATGTG
TGACTGCCGCTGATCACCACGTCGGCTTGACTCGCACTGAAAAAAAAAAG
>lmaj_27_9_up with T67304 1079532, 1079933
GACGACGCTGAATGACGAGGTCTCTGCAGGGGCGGCGGTGGCGGTGATGACAGCCGTCGTCAGTGCGGCC
GGTTGCACCCTGCGCTTCTTTTTGTTATCAACATGCTTGTGTGCTATGTTTGCCTTAGCGACGCCTGCCA
CTCGTTGACGAGCGCCCAACCCGTCTTACCCCCCTCCCCTCCGGAGGCTGTGACGCGGCGCTCTGCGCCT
CAGCCTTCCCCCAAACCATCTCGCATACGCAACACGCACACACACATATATATATATATATATATATATA
TATATACACACACACACGTGTGTGTGTGTGTGTGTGTGTATGCGTGCCCCTAACGCACACCCACATTCCC
CTTGCGCTCTCTACTCGCTCCCTCTTCTCCTAACCGCGTCTTTTCTGCAG
>lmaj_28_1_up with T67377 824435, 824836
CAGCTAAGATTTTGGCTTGGATCGGCGTTTGTTCCGCTGATTTCGTCTGTACAAAAAAAAAAAATATCGCA
TGGTTCAGTCATTGGAAGAAGGAAGTGGTTGGTAATGAGTACGAGCGTCTTTAAACTTTACAGCTTATTC

TTCCCGATATGTTTCCTCGAGTCTGAGTAAACTAATGTACATATATATATATATATATATACACGTATAC
ATGTATGTGTATATAAGGAGCACCACTTCATACCGCGAAACGGAGAAGTAGGCCCCTGCTCTAGAGCGAT
TGGTGTGATCATGGCTCTTTTTGCGTGGAAAAAAAAATCACGAACCCTTCATTTTCCACCGCTGTGTGCTT
TGCTCTCTCTATTTCATTCAATTCGATGCACGCTTTCATTTTCGATGCAG
>lmaj_28_2_up with W88360 36420, 36820
ATTACTCTCGCCATTCTTGTGTATCATGGCGCACTTCTTCGACTTCCTCCGCGGTTGTCATTGACGTGGG
TCTGGTGGTGGTGTGTGTCTCTGCGGGAACTTCTTGAAGCGCACGCTTGCCGCATGCAGCCCGGGGCGTG
GCGCCATCGAATGCAAACCCACTCCGCATGACCTAGGCACCTTACTTTCTCAGTCGATATTCGATGGATT
TGTCCTCCCCTCTCCTCTGCCCTGTCAAGTACACTCTCTTTCTGCTTTCTCTCCTGGTGTTCCTGCTCCG
CACACTCCTCTGCTCTCCACATTTTGTTTTCTTGGCGACTCTTGTTTCACCTCACTTCTTGGCTTGCGCG
CGCCACGCCCCCCACCCCCCTCACACACACATGCACACATATGCACGCAG
>lmaj_28_3_up with AA060771 938332, 938733
GCGATCGGTGGCGCCGCGCGCTTCTTTGCGTGTTTCTCTCCGCGCCAGTGTCAAAGGTATCCAGGGCGAA
AAGGAGCGGGTGAGTGGGATAGGGGCAGGACGGAGGAGGTTATCTCTGTGGCACGCCTGCTGCCTCCATC
CCAGTGACGAACATGATATCCTGTGTGCCTCGCCCTCCCCCCTCTTCACGGCTCTGCGCGCACCGCGTGT
GCACCGCATCCTTTGCTGCTCTTGCCCCCCCCCCCCTCCTACCCCTCCACCTCGTATGCCGCGGATTCCT
CTCTCGTGTATCCTTTTCTACTGCTGCTGCCGCTGCCATCCCCATCCCCGTCGATGGATGTGTGCACCCG
CTTACACATGTCTGCGGTGATTCTTGTCCTCATGGTCGATTTGAAATTAG
>lmaj_28_4_up with T67340 585726, 586127
GTGTCCTTCGCGCTGGTCGTCCCGCGTGCTTCCCTTCCGGGTAGATGGAGCCTCGAGCAAGCATTTTGCC
GGAGCAACAACAGCAACGACAACAACAAAAAAATGAACACGCAGACGCAGGCGCGCCTTCTCTCCCTTCTC
TGCATGTGTTCGGTCGTTTTTTTTGTTGTTGTTTTCCTTTGGTTTCTTCTCCTGCCGCGTGATCGTAGTT
AAGCCTTCCTCACGCAGCGTTGGGCACGTGCTACCCTACGGCACTGCGCAGCGCGAGGTCCGCACAAGTA
CGCCGATTACTTTTCGCCTCTCGCTCTCAAGGCTCGGACGCGATCTGCAGGGACTCAGCTCTACCATCCT
TTTCCTCCTTCCGTCCCCTCCCCCCGCCCTCTCCCTCGTCGCAAAACGAG
>lmaj_28_5_up with AA125618 22446, 22846
TGATGCGTGGTGGTGATGGCAGAAGTGTTAGCGGCGATCTCGACACTAGGCGGTTATAGTTTCTGTTTTT
CAGCCTATTTGAAGGCTAAGGTGCTCCTAGTTGTTGTTTCGGGAAATTCCTTTATTGCGTACAATTCAAA
GAACTGCAACCACTTGCGTTTGGGATTTTTGCTCGCCAAGTCTGGCCGCACATTCTTCAATGCGGTGCGG
CAGCGTTGGTACGTCGAGGTCTCTTTTGTTTTCTTTGGAGAGGAAAAAGAAACGGTGTCCTCGTGCTCTG
CAGTCTATGTCCTTGACAATCTCTACAGCTGCGTACTTTGAACTATCCTTTCGTGCAAGCACTGCCCCTC
GTTGATATCCGCTTGACTTTTTTCTCGTCTCCCTCTGCCTCTCGATGAAG
>lmaj_28_6_up with W88253 30221, 30622
GTATGGCAGTGAGTGGACATGTTGAACAAACGACAATGAATCATTTTATATGTTATGTAGCTCCTTACTC
CGCACTGGAAAACAAGAAAAGAACAACCAAAGCGACACCGCGTTGTGTTTGAGGGCAGCCGGTGAAGTAC
TCTCTGTGGCAGGTCCTACATGGAGTGGTGTGAGGGCCGCACACGCACAGCGGGGGAGTCTGCCGCGCCA
TTTGCACTGAGGGCGTCCTCTCTTCGCATCTCCTATCTCCATTTACTGCTAGCTGGCAGAGTATGCACAC
ATTTTACACCCACGTGCTTATCGGCCTATCAAGTCCTAATTCATTCTGTGTGCTTCTCCTTTTCTTGTCC
GATCGTTCTCCTTGCACATATTGTATCGAGGGATCCTGTGCAAACGCGAG
>lmaj_28_7_up with T93412 113826, 114226
AAGCGTGAGTCATGGCTGCATGCAAAAACCACCGCTCCCCCCCCCTTCTCCCTGTCAAGGCCGTTGGAGG
CACGGAAAAGTGCCAGCCCCCGTCCTGGTCTGCTCCCTTCTGTCTTCTCTCGTAGGGTGATGATGGCGAG
CCTCGCCTGCTTGAGCTGACACGTCCACGCCGCACTGCCACCTCTCTGACACACCGTTATCATCCGCCGT
GACTTTTGGGTTGGCGTGCACCTCACCCCTTTCCGTCCACTCCCCTCTGCTGTCTGCTCTTTCTGCTCCT
TCGTCTTCGACGTCTGGCGCCACATCTCGACGCCGTCTCGCCGCACCCCCCCCTGCCTGTGTGTTGGGTG

GAATGGCGAACTCACTCGCCGATGGTGCACCACAACTGCCCCATCTGCAG
>lmaj_28_8_up with T93459 114299, 114699
TCCTGAGTTATTTGATTGCCGCTTCTCTCGAGTCGGCACCCGCAGACGCCTCTGTGGCTGTCCGTCTTTC
TTTGTCTTGGGAAAGTGAAGAAGGGTGTGTTCTCCACGGGGCGGCAGTAGCAGCGGCAGCAGGATCTGCA
GCCACCGTTGCGAGCAGAGCGCTGCTTGCACCTGTACTCTCCACCCTCGACTCTCTGCATATATATCCTG
CGCGTTGGGCAGTGACGAGCCGTAGCGCAGGCTTCTCTCGCGCGTCTGCACATCCGCGTCGCTATTGTGC
GTGCTGTTGCCTCGCTGGACCACGTCGTGGGGCCATCCGCAACCATCTCCTTCTTTTCCTTTCTTCTCTC
TGTTTCCGTCTCGCGTGCGCATCTGTTTACTGCCCTTGCGTAATCATCAG
>lmaj_29_3_up with AA060754 1166726, 1167127
TCTCTGCACTGCTCTTTCTGGACGCACTAGGGTGCGAACGAGGGAATGGACTGCGCGGGTGCGAAGCTTA
CAAGCAAGCAAAGCAGACGAAGCCTCTGTGCACACCCGCTGCATGCGCTGTAAAACGAGGTGGCTTGTGG
CTCGCTGAAACCGGTGCGTTGCCGTCTCCGTCTTCTGCGCGGCACGTCGATCACGCGACACTGCACCCTG
CGGGGCCGTGGGCATGTGCTACTGCAAGTGAAGCGCTCCTCCGGCCTCTTCACTAGTGAACGACCCACCT
AGTCGCTTGCCTCCGCTCTCGTCTGTTCTTCGTGGTGTTACTGCCGGCCTTCATTGACATACATCCGGAC
ACGCGTGCGATGTGGCGCGCTCTCGTTTTTCTTCGACCGGGTCGCACGAG
>lmaj_29_4_up with T67333 1155435, 1155836
CGACGTTCCCCCATCTCCTAGTCGTTCACCCTCTCAGCGAAGGTGTTACAGAGATGGAGAGAGACGAGAG
GGTTGCGACGCTTGTTGCGCCACCACCATCACCAGCCACTGATCCCCTGGCAACAGAGAAGGTTGACACC
GGCGGTGTCGAAGCGGGTGCAGGGAAGAGTGGGTGGGGTGGGGTGGCGGCATTGTATGCGTGCTCTCTGC
TACTCTTACCATCACCCCCGTTACTCCTCCCTCTTCTCGATCTCTAACTTGTCTCCTGCCGACGCCGCGG
CATCTCTGTGACACGCGGTCGTCCTTTCGTTCGCGGCTCTCTCTGGATTTCCATACACCATTCTGTCCAC
CGCGTGTTTGCTCTTGGGTGTTTCGTGCCGCGTATTGCTTTGCCACTCAG
>lmaj_29_5_up with T93344 537532, 537933
TAGAGTTTGGCGTGCCACTGTTGCACGTATGTGTATAGAGCAGCCGCTCCTTTATCGGGGTGAATCCTCC
AGTAATTTTGCCCACACAATGGCGCATGAGAACGAAAAGAAATGGCGACACACGGCTTTACTGTGAGACT
TACCCTGTACAGCACATGGAAAAGAGGAGGAAAGAGATGAGCCAGCACAGGACAGCTTCTTTCCCACATC
CCCGCCTCTCTCCGCTCGCGTTGCGCGTGATTTTTTTCCACCTGACGCAACCCACTGCGACGCTCTCCGA
GTCACCTGCTTTACAGAGCCGGTGGCTGATCCCGTTGCCGCGAAAAATCGTCTCTCCTCCACTCCCACGT
CTCTCTCTCTCTCTCGCCCAATCTCTCAAACCGACCCCCTTCACCTAAAG
>lmaj_30_12_up with T93404 234389, 234790
GCAAGCATCGAGGATACGCCCCGGCCTATGTGGAACTAGTGAACTTGTCACTTTTCCTTTTGATCCTGAT
TCTCTTGCTAGTCACATATTTTATTAGCACTTGTGATCTTCACTGCGGTGTATCTGAAGACCCGAAACAG
GAACCTGAGTAACTGCGGCAGCAAAGGCGAACGAATGCAGTTTCGAAAAACGTACTTGAATGCAGGTATT
GAGTAGCAACGCAAGAAATACGAAGCCGGGTCTTTACTTGAGTCGGTTTTTGTCTGTGTTTTCACTATGG
GAGCGAGATTCGACCACAGGGTACTGATTCATGACATCCTGGTGCAGTACTCGCTTGCTTCCATTTGCAC
TCCCACTTCCACTTCCCTCGTGTGTGGCTTCCTCTTTTTTTTTGCTTCAG
>lmaj_30_13_up with W88286 1192477, 1192877
GAGAGTTTTTCCTTTGTTTTTGCTTTACCTGCTAGGCAGAAGTTCTCTAGTACATTTTCTTTGTCATTGT
TGCTGCCATTAAGCTTCGAGAAGTCCATGAGTCACCCTGTAGCAAAAAAAAAGCAAAAGTAAGCAAGCAC
ACTTTGTCAAACACTGCTCTGCTAAGCGGAGGGACCCCATGTCCCTCCGCTAGTTTCCCATGCGCAGACG
CATTACGTGGGTGAAATACCTCTTGTCTCCTGGTTTTTGTGTGGCTTGGAGACAGGTTTTGCAAGCTTTA
TCTGCTCTGACCAGCCTTACTATCGATCTGCCTGAAAAGAAGCAGAATGATGCGCTATCGGCGCTTGCGT
GAGAACGATATTCGCTTGCACGTGTTTCTTTGCCTCTCCTTTCTTTGAAG
>lmaj_30_15_up with T67384 1248273, 1248674
TTGCGACTTCACAAGAAACAAAAAAAGACATGAACAACAGCAACAACGAGATGCGTGACGACTAAAGCTG

TTTGGACGTGCTGCCTGTCTCGACGGCTATGTTTTCGAGGTGCTTCGCCTCTTTTTGTGTTCACGCCCAG
CTTGATCTTGTAACCTCCTCGCCGACCTCATCGCATCATGTGGCGTCAAGAGCTAAACGAGACGGGACCT
CTTCAAGAAAAGAGGGGGGGGGGGGCTGGATCCGTGGCGCGACGTGCGTGAGCTCTGACTATCGAGCCAC
GTAGAGGCATGCTATGGAGCGCCTTTTTTTCCCCATCTCTCTCTGGTCCCGTGAGTGTATCCTCTCTACT
TCACTCACCCACTCTCACTTGCGCTCTGCGACACTACTCAACGAGGTAAG
>lmaj_30_16_up with T67381 605049, 605449
TTTCTTCTCCCCACCACCACCACCACCACCACCACCACCATCACCACCAAACAAAAAAAAACGCAGATAC
GAACCCTATCGATCCCCTTGTGCTTTGGACGCACAGCGTGGACGCTGTACACCGACAGTGACGCTTCTCA
TCTCACCGCGAGAAAAAAACTCTGCAGAAAACGGGACGGCGCAGACAGGTGCTGTTGCACCCTTTGCCAG
TCTACATGTCAGGAGTGGTATGCGAATGGCGCAGTGTACGCTGCCACACGTCCTAGTCAATACCCGAGCC
ACGAATACGCGCCGGGATGGTGTGTGCCTTTTCGCTCTACCACTTGACTCTCTGGCATGCACTTTGGCCA
TCGGCTCTACCACCCCCACCCCTTACCCATCTATGCCCACATACGCATAG
>lmaj_30_19_up with AA125633 1259432, 1259832
CTCTCTCCTTGTACGAGCGCTGTCGCTGCCGTGGCACCGTCAGCCCCGTCCCCTCTCTTTGTGCAGTCGC
CGCTCCATACTTTGTTCTCTTTATTCCTTTAAAACGAAAAGTGCACGCTAAGAAAACGAAAAGATTCCAC
CGGCACGTAATCCCTCGCCCTCCCCATCCCCCTCGCCGACTCAGTGTGACCGCCACTGCTGCTGCTGGTG
TGGGCGTGCGGATATGTGTTGCACCCGCGTGCTGTCATGCGCGCACACCCTCGAGCTTTCTCTCGCTCGC
TCGCTCTTTCCACTCTTTGCTGATCGAACAACGGCTGCGCTGCGCGCTCCCCGCACATTCCCCATGCGAT
TATCTCACATGCTCACTCCCTTCTGGTATGCTCTCTCTTCCCCTCCACAG
>lmaj_30_20_up with W88369 181295, 181695
TACTACACACTGGCTTGGTGGAAACTCGGCGAGGGCATTTTCGACGCTGACGACGAGGATCGCTAAGCAT
TAGTCGCAAGGGTCTTCCTGAGTAAACGTTCACAGTGTTGTCGATGGGTTTTGCTTGAGTTGAAGAAACA
TAAGCAAAGGTCTTCAGAAGCGCGGGACTTGAATATATGTGTGATTGTACTCTGGGCACCAGCAGATCGC
TCTTTTGCTACACCGACGTCACACAAGCTGTTGCCTCAGCCAACACGACCTCGTGCAGCGTTCTGCTCAT
TCTTTTTCAACACGCTCCTGTGTGAATGACTATGAGCATTAATGAATACCTCAATCTCCAGCTTTCCTGA
TCTCCTCTGTGTATCTTGTCTCCTCGTGCCCTCTTCACTCCCTGTCACAG
>lmaj_30_21_up with T93435 323548, 323948
AAGCTTAATTGACATTCAAGCTTCGCACAAGTATTTTTCCCCCACTCAAGGGTGTGTTATTGTTTTCTAC
TTGATTGATGTACGCTCACAACAGCGCACCTCGTCTGAAAAAGCACTTGATACTGTTCCTTCTTTCGCAT
ATGTTCATCTATGAAAGAGCAACATTATTAGGTCACCTGTTTCGCGTGGGCGCACGCACACTAGGGCTAT
GAAGTTTCGCTTCATTGGCAATTCGTGCTCTGCTGAGGCAGCCGCTGTTTTGCTTGGTGCTCTTTTTCCC
TTTTGTTCGACGCAGTGCATTCCGCTTCTCGCAGCAACCATTCTCCCACTCTGTTTCCTTTCTCAACTGT
CGTCGCTTCTATTCCCTCGATCTTTTCTCTGGATGCATGACAATTCGCAG
>lmaj_30_2_up with W88408 1193484, 1193884
CAGACGGGCTCAGCAAAACTGTGCATGGAAACTCCATTCACTGCTGTCGTCACCGCGGCTGCATACGCAA
AGGCGATCAAAGCACCTACAAAGAAAAGTCTCTTTGTTTTTTGTGGTGGAGTGTCTCCGCGCACACCCAT
TCAGGTCGGCACCTACGCTTTCGAATGGGGCCGCTTTGGTCGGCTTAGGTATTCGGTCCTCCCTTGAGAG
TACTTGTATTGGTCACACAGAAAAGTGAAAAAGAGCTCATTGGCGGACAGCGCTATCACTGCCTTCTGCA
GCCGCAAAAAAGCTACCGTGCGTTTGCTTGCGTACGTGACACTAACGGATTACCGAGCTGTGCGTGTGTG
TGGCGACGTCGCTCCGCTACTACATCACGCGGGGTTGTTTGTTTCGAAAG
>lmaj_30_4_up with T93408 644391, 644792
TGGCTCATTGCTGCTAATCTGACTTTCTGATTCTAACCCTTCATCTGAAACACGCAAGAAAAGCCTGTAG
GGCAAAGCGTATTACTACAGTTTTGCTGTCCTTCTACATTGCATGTACATGGACATTCATGTTTCTTTAC
TGAAGCGAACCAAGAGCAGTTGTTTCGATAATTATGGGCTCGTTGGTGCCTCTGGGGCTCCGCCTGAGCC
TGATCGTATCCGCTGCTCGCATGGTTGTGTGACTACGTCGCTTCCATTTGAATCCCTGCATTTGCGGGTG

CCCACAGCTTGGGATCTAGCGTCTTCTCATCTCACGCATGCTCTAACTCTTTTCCCTCGCTGTCTTCCCT
GCGCGCTCTCTCCTCCGCTGTGAGTCCTTCTTCGTGCCTACTTCCCTTAG
>lmaj_30_5_up with W88349 347303, 347703
CAGGCCAGCGCACGTGACGTGGCGCTGTTCATGCACACCGTGCTGGTGACGCCGTAGTCTCGATGCCGGG
GCAGAGGCACGAGCGCCTCAACTGATGCGGCGCATCGACCTTCTGATGACGCCTTTGGGGAGAGATTTGC
GGCTCTCACGATCAGTCGAGTGTGGGGTGTGTATGCGCCTGCTGCTGCTTTTGAAGCGGGCTGCAGTGTC
GAGCGGAGACACACGCCTCATACTGCTGATCGCCCTTGCACGCTTCAAGCAGCTGCCGTCGCCCACGTCT
CTCCTTAACAGCTGATGTCCTTCTCTCTCTATCGCCTTCTCTCTTCTCCTCCATGCAACAACGGAAACGC
CGCTGTTGGAATCTACGACCCTGCGTGCGTACACGAGCACGCACCCATAG
>lmaj_30_6_up with T93433 322637, 323037
AACAACCGCGTTGTGTCAGCAAGCGAATTTGAAGGTAACACAATATCGCCTTTTCTTCTCACATGACATC
GTTTGGTTATCAGAATTCGCTTTATGGTGGCACTGCGTTTGCCATGCGAACAGCACCAGCGACATGCAGT
TCATTTTCAAACCTCGAAGTGCCGTTTGATGGAGCGAAGGTGTAGGGCGCCTATTCTTCGGCACTTTTGA
AAAAGAACCTATCTAGGTGTTGCAGCGAAGCGTCGCTAAAGCACTAGCGTTTCTGTCTAGATATGCGCTC
TAGAGATCACACTATCGGAGGCAGATGTCTCTTGTTCTGCACTTGCGCACTGCTGTTTCTCAATCACTCT
TTCCCTTACCCCCTGCCTTTCTATTTCATCTCGCACTATCCTTCCCGAAG
>lmaj_30_7_up with W88335 180642, 181042
CCTCATGCTTTTCATGCACCCCTCCTTACTGACGGCAAGAACAGCTTCGGCGTTCTGAACATCTCTGGCA
ATATGGATCTGATTTCGATTCTCTCAGAAACGTCCGCGCATTTTTCACAGATTCTTTCAACTTCTACAGT
GCGCGAAAAGAAGATGTGCTTGTTTTTTACACTCCAGTCACAACCGGTTAAGCATTTCAAGGCTTGCAAA
TACAGCCCAGTCCAGGGAGTGGCGCCGTCGTTTTGGTGCAAAGCTGTACTGATTGTCCGATAAGAAAGGC
AATCGTGCCGCATTCATCACCGAGTCGTCAGCCGACTTTTCTCATGCTGGATCGTGTTGAAATCTTTTTC
CCCACTTGTTCGCCTCCTTTTCACTTTCTCATCTCTCTCATTCACAACAG
>lmaj_30_9_up with W88385 638889, 639290
TATAGCACACACACAGACGCACGCATACAGTAGTCAAAAACGAAACGCCCTAAAGCCAATCATTCCGTCG
CCCTTCCCTGTGCTGCGGTCGCGTGCCTTTCATCTGCCGCTTTATATTGCTGCCTTCGTGCGCCTCCTGT
GTGCTGAAAGCGGCCGTAGTGGTGCGAAGGACGAGAGGCGGCGCGAGGGCGATGCGATTTGCCGCCCCCC
CCCCCCCCAAACACCCCTTTCCTTCTTTGTTGTTAGAAGGATGAGGAGCGTAGCACGTCCCTCGATTCGC
CCGGTGATGTGTGTGGGGGTGGGGTGGGGGGTCAGCGCAACAGTTGCACGACTATTCTCTCCTTCCCGTT
GTCGTCTGCGCTTTCCTGTCTACGTTCTTTTGTTGTTTTTTCCTTTCAAG
>lmaj_31_10_up with AA125628 1475189, 1475589
GGCTTGGGCTTCCGCTGCGTGGTGCCAGCGGCTTCGCCAAGGGACGGCGCGCCGCACCCAAGTCCGACAC
CCCCACGAGCGAAGAGAACAATCTGCACGCGCACCAGCCCCAAGGAACGCCTCTCGCCACCGCGGTCGTG
CACCGCGCCCGCAACAAACGCCAGCGTCGACAAAACATGTGCAGCGCTTCCCTGCCCACCATTTGTCCGA
CGTCATTGGAGAATTCACTTCTCAATCCAGCGGGCCGAGGGCAGAAAACGCTCTCTGCCCTTCTCACTTC
ATCTTCTCGATATTCTTCACCACGGCGGCAACCTCCTGCCGCTCGCTTGTGCCCACAAATGTCTCTCCCT
GACCGAAACTCTTCTCATCGTTATTCTTTCTCTTTCTTGCTCCGAAACAG
>lmaj_31_11_up with AA060778 1150687, 1151087
TAAATGAGATGGGGTCGCTGTAAGGAGTGCGCTGCATATATATATATATATGCGTACGTGTGCGTGTGTG
CGCTATTCGCGCAGTCGTGCTTCCGCCTTGCACGGAACCCATGTGCTCTAGAAGTGAAACAGTTTGTCGT
CGTAGTCACAGCTCCCTTCCTCCGTTTCAGATTGTGAAGTACAGTCTCTCAGCACAGCCGCTACACTGCA
AGATCTGCTGTGTGAGGCCTTGGAAAACGAGCATAGGCACAAAGGAAAGGCAAAGGAATCCGCAGACCCT
CTGATTGGCTGCTGAAGACGTTGTTTGCTGAAGATTTTCCTTCTTCATCGTCATCTTTCTCGCATCGCTT
CTTTCCTCTCTACACTCTCGCATACCGCTCCACGTCCACCAACGCCGCAG
>lmaj_31_12_up with W88300 1042048, 1042448

TCTTTGGGTGTCTTAGTCGCAGAACACACAGCGATAACAGGTGGTCTCGTCTGCACGAACTCGGGGAAGG
ACTACGATGGTTGGCGGGTGGGCAGTCTGCATCGCTGGCCTCTTCCTGGTTTGTGTGTCGCGTCTACACG
CTCTCAAGGATCGTACGCAACGCGTGTGCTGACTGAACCTCAACAGCAGCAAGAGATGCTTTTAGGCCAC
GCTGTTCTGCCACTCGGAGATGTCCCCCTTGGAACCGAGCGACAGCAACGCTGCGCACGCTGTCGTGTAT
GACGAATGACGCGGCGCTCTGACTGATCTTGCGGTTTGCAGGGCAGACAGTGGCGTTGTCATTGCAGCAG
TGCATCTGGCATTCGTTGTTGTTACCCACTTGTCGTCTCCTTTTTCTTAG
>lmaj_31_13_up with T67355 711379, 711779
GGAGGTGATTGACAACCTGACTGGTGAAGAGGACGTGCGCTCCGGCCTTGGTGCAATGGGCGTGCTGACG
GCTGGCCGCCTTGGCCGCGCAGCAGCTTATGAGCACGGGCTGGTGTTCGCGCTAGCTCCGTTTCTCTCTG
ACAGCAAGTATTGGTGAGTGCTATCGATACATGTTTGAACTTACGGCTGCAATAGTAAACAACGCCGAGT
GCTTAAGCGGCAGACCCTCAGCGCCGCCATCACGTGAGCATAAAAGCAACTTTGTGTGCGAGAGAAGACT
TGATGTTCCTTGCCCTGCTATTGGGTGTTTGAGATCCGTGTGTCTGCGGTCTTGAGAGCCTACTTTTAAT
TCTACTCTTTCACTGTCCCACGTCAATTGAAATGAGGCTTGCACTCGCAG
>lmaj_31_14_up with AA060755 966678, 967078
GGAGTTTTCTGGAGAGCTCGGTTGTCTCATCGTTTGCATTGAGCGATGGACCCCTGTCTCACGTGCGCGG
GGGAACGCACCGTGCTCGCATGGGCGCGTGTGCGGTGCAGAAGCTACGTTGCTGGAGTGCAGCACGGGAG
GTCTCGCTACCGCATCGATGTGAAAGGAAACGTATGCGATGTCGGACTGCGTACTTTCCGGAGGGTTCCG
ACTCGACCCCGATTGCCCTGCACGCCCGCAGTGTGGCTTCTCCTCCACTCTAACGAGCACCATCGCCCCT
TTCACATCCTCTTTTCTTGCGTCCTGCATCCCCATGGCGAACAACTCTCTGTTTCGGTGAATGATGGCGC
TTGCCTACTCTCCTTTCGAGAACACCAACCATGACAACATGGAGGGCAAG
>lmaj_31_15_up with T93365 538244, 538644
ACGGCGCACTCCACCTGCCCAGCGACTCCAGTGTCTCATCGTTAGCAGAAAGGGGGTCTCTCGGTTTTGT
TTGCTGTGTCGTCCCCAGTGAGCTAACGTGACTATGGGGAGGGGGGCTCTACATGTCAAAACGCAACCTT
TGCTGGGTCTTCCCCCCCCCCCTCGTGCTTCTTTGCTCGAGCTCACGCTCTCATGCGTTGAGACTCTTGC
CGTCTCCCTTCCCACTCTCGCCCTCGCCCCCGTCGTGTCTTTCTATGTGTGCAACCTCGATGGGGTGTTC
CCTCCCACATACATACGCGGCATCAATGCGGACTCCTCCTCCTCCCCTACACAAACGAATTACACGCATA
TATATATATACATATCTGTGTATATCCACAATCATGCGTACATGCCAAAG
>lmaj_31_1_up with W88344 118738, 119138
GAGTGGCGCGTTGTCTGCGTCGGTGGGCCGTGCGCGTAGCGCGTCGGGGATGCACGCATTCCTGTGCCGT
TGCTCCGAACATTCAAAAAAAAAGAGAGAGAGAGCTACAAGCATGCATTCAGCAGCCCTCACTTGGAGGGA
TACGGCGCTGGCGGCCCATCATGGGTGCCGCTTAGCTGTTGTCTTTCAACTCTGCAAGGCCGCTGGGTTT
GCGCTTGCACACACGGCTGCCGCTGCGTGAGGTGAACACACGAATATCCGTTGTGCGGGAACTCTGTCCC
GTTTCGAGTGCGCTCACTGCCGGCTCTCTCCTTCATCTTCCTTCGCTGCCCACAATTTGCTTATGCTGCG
CTTCTCATCCCCACCTTCTCTGCCGTTGTGTCTGAGGTGCTGGTCGTCAG
>lmaj_31_2_up with H64193 687808, 688208
ACTGATCCCCACTGCAGCGCTCCCTGCCTGCGCAACCACACATCTATCGGTGAACGAGGGATATGACCCT
TCTTGGTGTCCACCTGTTTGCTGTCTTCTTTTGTGTTACTGATCGTATTGCACCAAACTGTGAATCCTCT
GCCGATGAGTTACGCCGTCACTCTTTCTTTGGGTTATTTGAGGGGAGGGGTTTGAGCGCTGCGTCTTTCT
TCCTAAACTCGGTGCATGTGCTTGTGCCAGTGTTTTCTGATCAAGAAATCACCTGCATGTTCTCAGAAAA
CGGTATCGCGAGTGTGTCTCTCAATGCCCCCTCTGCCCCTCACTTCCCGCTCTTGGCCCCGTTCCCACCC
CTCACCCTCGACCCCCCTCCTCCTCCTCCCCCTCCACCTTGGCTCATTAG
>lmaj_31_3_up with T67363 12395, 12795
ACTAGCGGCAGCAGACGTTTTTATTACCACAGGCAGGTGTCGGGGATGGTGCTGCGGCTGTTGTTGAGA
ACACGGTTCGTCAGTGATGCTGGTTCACCTCTTTCAAGGCGCTGCTTGTCCTCCTCCGAGGCGCGTTTTT
ACGGGTAAGGATGAAGGTATCCGGCGACATCCTCTTAACAGCTCCACGGTTGAAGTCAATCGACACGGAC

GCTGAGTTGCAATGCTTCTTGTAGCTGAAATGAAAGCCTTCTCCACTTACTGCTTACTGCCGCTCTCTTC
TCTGTTGTGTCTTCCCACATGGCAGGATTTCAGCTCATCATTGTGACCCACACCATTTTGGCAACTCCCT
GTATTTTGTGACCGAAATGAGCGCCTTCTCGTCTGTGCTCGGTGTGCCAG
>lmaj_31_4_up with T93387 147920, 148320
AGAACACGGCGAAAGGTCAGCTGCGCAATACCGCGGCCATGGCGGGTCCCACGACCCGCCGACGGCGCAC
TTCCCAAGGTCCTGCTTGTGATGAGCAGCGCGGGCTGCATTAGCTGAACAGAATAGAGGTGATGTGTTTT
CGTCGCTGCCCTGCTCAAGCGCTTGAATTCCTTCACGTACACGCACCGGCGCTCTATACACTGCAGTTAT
CTTCGAATGCTCACTATATATATATACATATATGTGTGTGTGTTTAGGAGGGGGCATGCTCTTATCCACT
CGTCGCTCTCACCCTCCCTCTCTTCTCTTTCTCTTCGGCGTGTCCGTCGCCCACGCGCGTCACAATTACC
GTCCCCGGTATTGTGTTGTCTTTCTCCGACCGCACTTTTCGTACTCCAAG
>lmaj_31_5_up with T93347 20074, 20474
CGCGAACAAAGAGTACACGATGGTGGTCGTTCAAACTGTCAAAGGGCGGTGCGCCTGAGGTGACACATGA
CGATGTTGGCACGCTCACCGTCCATTTCATGTACATAAAGCCCGCGGCAGGAGTCAAACGACGCTTTGAA
TTGTGCGTGGAAGAGCAGCTGACCATCCTCGAGCGCCTTCCATGGTGTTTTCCGTAAGTGCGACGTCCAC
ACACGCTGCTGTCCTTGTGCGGGGTGTCGGTGACTTCCTTCTTTCTTGTCCGCCTTGCTCACTACCGCCC
TGATGGGCAAGCCGCCGCCTACTAGCGCCACTAGGGCCTCCTCGTCCCTCTTGTGGAGAACAACAAGCCA
CATGGTAACTCTCTCCTTCTCTGCTCTCTCCCTCCCTCTTGGTAGCTCAG
>lmaj_31_6_up with W88388 129442, 129842
GCATGTCATGCACCTGTCCTCTCTGTCCGAGGCATCTTTCACCCTAAACCACAACGAAAGCGACTGCCAC
GCATTAGCGGTTCGCTTCGCAAGCTACGATCGGCTCAGGCAGTGTCTTGCTCTGCAGGCCATAACCGTCA
GTGCCCCCTCCCCCCTCCCATACAATGCTCGCAGTGCATTTGCATGCACGAATAGCCGATTCTCGCCCAT
CGCCGTGCTGCCTTCCTCTTGTACCGCCGCTCTCTCTTTCTCGGCGTTAGACATTGTGCATGTGGGCATT
CATCCACACCAGTGCCTGCTCCGACTCCTTCAGATACGGCCGCCTTTCCTCGCCTCCCGTGCTACCTCCC
CTTCCTCCTCCCCCGCTTCTCGCTTCTCGCCTGCTTCCCCGTGTGGACAG
>lmaj_31_7_up with AA060744 1273197, 1273597
ACGCCAATCATGAAGGCGCTCCTCGCTCTTTCCCTTGTCAGGCGACGATGAAGAATCGAAGGACGCCTGC
GCCGCCGCCTGTTGTGAATCCTCCTCCACAAGTGGGGAGAGAGACACCGGCAGTGTCCGTGCCACTGCCT
CCCTGGCTGCACATCTCCTCCCGCGCCGCCTCTTCTCCGTCCCAGTCCTTTTCTCCCCCCTCCTGCATCC
ACCCTCTGTTCATCGTGCACCAGAGAGGTGTTCCCCGTTGAGGAAAAAGGAAAGTGCGGCAAAAATTGTC
GCTCGCGTTACGCAACAGCCAACCTCTCTCTCTCTCTCTCTCTCTCTCTCTCTCTGCGTGTGCGTGTGCC
TCTCGAGGACGCTTCTCACTTACACTCCCCTCCTCCCTCTCCTTCGGTAG
>lmaj_31_8_up with T93420 861780, 862180
CTTACGTACTGTGTCGTGTATTGAACTCGAAAAGGGAAGGGCTATAGGCAGTGCGCGAGTGGCCTTGAAG
GAGGCGGACATGCACGCGTTGCTTGCCCACGAAGCCGAGACGTGTGCGGTATGGTCTCTTTGAAGAGGTG
CTATCCGTGCGCCTCAGATGGACTCATGGATGAAGCTGCGTCGGTGCCTCTCAGTCGGCAGAGAGCAACC
TCTGGCTCGTGTGCCTTTTCCCGTCTTGCTGTGTAGCGTGCTGGAAGGTGGATCGGACGTCGATGTCTTG
GTGTGCTTTTCTGTGTATGCTCTACAGCAGGGCTGCGCCTCTCTCGTCCTTTAATATCCTCGCCTTGCGT
CCCCCTTTCTTTATGGTTGGGTTGCTTTCTCGATCGCATTGCCATGGTAG
>lmaj_31_9_up with T97254 1061123, 1061523
AAGTGTATAGAAAGACGAAGGGCAGCAGCAGCCAAAGCGAAGCGTGATTAAGCGCCGCGGTATTCGCGAC
GGCACTCCCTCACTCATCGGTTCACGTCCTTCGCAGCAGCCACCGCCTCAGCAGCACACCACCACCACTA
CCACCACTGTTGGGGAGTCATAGCAGCAAAACAAGCATACACTGCTTGGTTATCGGTATATTCCCTTATA
CCGTTGATCTCCAACCAAACCGTACACGGAGTTACAGGCACTCGAGATGGATACCCCCTTGTACTACACG
CTTCATCAGCTCTTCTCCCCCCCCCCTACGCACACACGTTTCCCTCTCTTAACATGCGAGCAACTGCCTT
CACCACTTGTTTTCCTTCTCTCTCTCACTCGCTCGCTCCCCTCTGGAAAG

>lmaj_32_10_up with W88305 909439, 909839
AGAGCGTGTTTGTGTGTGTGCGTGTGTGTGGAGGGGGACGACGGCGCAATCTCATGGAGCTGCACAGGCA
ACGTGGTGTCGTGTGCCATTCACTGAGCTTCTCACCCGGCATTTCCCTCAAGGCAGAGACACACACGCAC
ACACTGGTGCTGTCCGGTGGCAGTCGCGCATGGGAACTGCGCCTTCACCGTTTCCCTTCGCGAGGCTCCG
CACCTGGCCCCACGGCCACGGACTTCAACGTCCGCTTCCATGTAATTTCCCGCGACGCCTTCCTTGTGCC
TGTTCACTACTCCCCTTCTGCTCTTTTCTTTTTACGGCGCATCTGTCATGCGGGATTCGCACACCCATCA
CCCGCCCTCTCTTTCAATCGACCCGCGCGTCTCCGATGCGACCAACTCAG
>lmaj_32_11_up with AA125627 1383073, 1383473
TGTGGTGATCTATTCCTCAACTGCTGTTATCTCCGTTTGTTCTTTCTGTAGAGAGACTCAAAGATAGTTT
TTATTCGCCTTTGTTGTCCCTCTCTCTCCTCGCCTTGTGTTAGTCATGGAGCCGAAAAAAAAAAAACACA
TACTTTCAATGTTAAGCACTTCTCTTCGTTTCTCAGGTGTGGGATTTTTCGTCATCTCATCTGCTTGGCT
GGGGGTTATTGTAAGGCCACCCGTAGCTCTTCTCTGAGTTTTGCAAGGGTACTCATCGTACCAAAAAAAA
ATACGATGCGCTCCAGGTCGGTTTCTGAAGTCACTAATCGTATTACTTCATCCTATGCATCATTATCTTC
CTTGGCCAATATTCATGCGTTCTAGAGTTTCTAAGCGCTGAAACTTCAAG
>lmaj_32_16_up with AA125645 194753, 195154
CAGCAAGGGAAGGGGAAAGGATGTTGGATGTGGTGTGTTCCTCCTCCAGTCAGCACCAGAGTGCCTGACA
ACAGCCTCCCACTCCTTTCTTTGTGATGAGAAGTACGGTCCTGAGCGTCGCCTGTCGGTTGCACAGGAGA
CATAGCCCCGCATATTATTTGCCCATCTCCGTGCGTGCGGGTGCGCACTGGAAAACCGAGGCAGACTGGA
ATACTTCGCAGCAAATGACAGTTATTATTTCTCGTTGTGGGGGCAGTTGATACCGAATGACCGCTATTTT
CGACGCCTTCATATCTTTTCCATTTTTCTCATGCTCTTCTCCGTGCTTTCCCTTGCCTCTTCCCGTGTTC
ACGGCGTTGTGCTTTTCCTGTTCAATGATCTCCCATCTGAACCGTTGCAG
>lmaj_32_19_up with W88382 190160, 190561
CGTTTTCACTTTCTTTTTTTCCCCATCGAGCGATGGAGAGCGGTATCAGCGGAGGCGTGATCTACCTCAC
CCTCTGCCTCACGGATCGCCCATCTACCTCCGTACACGATCAAAATGAAAAGCAGCAGCACCGTTTTCCA
TCTTCCCCACTCCCAGGCAAAAGTGCGCAACAGTATTACACCTTTCGGTGGTGCTGATCATTGAAAGCAA
AGAGCGAACCTTGTGTACATGCAAAAGCACATGAAGAGGACCGCATCCTTTGCTTAGCCTTGAAGTGCCG
AACCCTGTCATGAACTTTTGCGAACTCAGTGCCCCTCTACGCGCCCTCTTTTCACTTTCCCTCGCGTCCC
ATTCTTGTATTTGCGAACCATGCCGTTCTCTCTCCCGCGTAGCATAACAG
>lmaj_32_1_up with H64197 1283528, 1283929
ATCGCGTCCTCGCAACAACGAAAAAAAGGAGTGGGATTCGAGATCGAAAAAGAGGGCTGTGCGCTGCGAT
CGCTGCTGGGCAGTCCCTCTGCTGGCTCTGCAGTTCTTCCGTCCCTATCCACCCCCATACCCCTCTCTCT
CTCCATCGAGGAAGCTGCGGAGGCAAAACCACCTCACAAAACGTTAAGGCTCGGCGAACGTGTAATGGCA
ACGCATCAGTGCAGCCGAGTCCCCTGCATGAATCGACTGCGCTTCTCTTTGATTATCGTGGCCAGGTGCG
CTAGCGCGTTGATGTCGTATCCATCTCTCTGCCTTTCTCTCACTCTTCTCCGTGCTCTCCATCTTTACAC
ACGTACGACGACACATCTGCATTTCTTGCTTTCTTTGTTTCGAATCGCAG
>lmaj_32_20_up with W88403 404291, 404691
TGTGAAGTCGGTGTCTGGTCCGTTTCCGGGACAATGCTGGAGTTTGCCGAAGACTAGAAAAAGAGCATTT
CCATGGATACATTTCTTTTCCCTCGTCTTGCTACCAAAGGCCGCGTGCTTTCCCTTAGAAGAGACTTTAA
CGACAGTAAAATGGCAAGCGTGCGGCAGAAGCCACGGCGCAAGGTTTTCGTCGTCTCTGTGTCTCCTTCG
CAGTGCCACAGGATCGGGGTGACAGAGAGATGACATCCTCGGCAACGCTTCAGCCCTGGCGGCCGCTTCA
AATGAAAAGAGAAAGAATCGTTGTGAGCCTCGTATTGCCCTCCTTCCCTTGCTCCTCGCGCAACTCCTTT
GCTTTTCATTCTTCACTGCCTCTCTTCAATGTTGACTTTGCCACTGCCAG
>lmaj_32_21_up with W88378 474844, 475244
TCTTCGCATCTGTGCGTGCGTGCGGCTGAACACCTTTAACCTCCCTCTTTCATGAGTCTTTCTCTTTGCC
TTTTTTGCGCGTTCCCCCCTCTTGGGCAGCGTCTCACCTCACTCTGCCGCCCCGATGCCTCTCCGTTTCT

CCGCGTCTGTTCCCTGCTTCGTCCCACCTCTCGCTTCCTTCTACCCGCATGGTACGCGTCTCGCCGTTCG
TTGGGTCATTTCTCCGGTGCGTCGTTGCAGTACGCCCTTGTAGAGAAGGGTTTCCTCTTACGTTGTTGGT
CTCTCATTCCCTGTGCTGCACCCACGACTCCTCCTTGCCCCACTACTGCTCCTTCTCTTTGCTCTACCGT
GCCTCCTGCCAACGCCCACTTCCTCCCCCCTCCCCCACGCTCACGCGCAG
>lmaj_32_22_up with T93446 1383048, 1383448
ATGTCTCGCCTCGAAAATATGTGACTGTGGTGATCTATTCCTCAACTGCTGTTATCTCCGTTTGTTCTTT
CTGTAGAGAGACTCAAAGATAGTTTTTATTCGCCTTTGTTGTCCCTCTCTCTCCTCGCCTTGTGTTAGTC
ATGGAGCCGAAAAAAAAAAAAACACATACTTTCAATGTTAAGCACTTCTCTTCGTTTCTCAGGTGTGGGAT
TTTTCGTCATCTCATCTGCTTGGCTGGGGGTTATTGTAAGGCCACCCGTAGCTCTTCTCTGAGTTTTGCA
AGGGTACTCATCGTACCAAAAAAAAAATACGATGCGCTCCAGGTCGGTTTCTGAAGTCACTAATCGTATTA
CTTCATCCTATGCATCATTATCTTCCTTGGCCAATATTCATGCGTTCTAG
>lmaj_32_23_up with AA125646 1554104, 1554505
AAAATTCTAACCGTGGTGCTCGCGGGTTTCATCCTTCTGGAAGGGACGCTATGTAGTATCGCAGTTGAGC
GAATTCGCTTTCTCCTTCGGCGGAGCGGTGACGCTACCGATGGCTACCAAGGTCAGATGGACGGAGAGAA
GGCAAGCACTGGATGTCCAATGTGTGCTCTGAGCATGTGACATCTCCACTTCTGTCATGGCCCACAAAGA
AACTGGGGCAGAAGTCCTAGTCGACAGCAGGGGGCTTGTATTCCTCTGTCGTTGAAGCTCCCCCTCCCCC
CGTGTCGCGGCTTCTTTTTGATGTCCCTTCACCTCGTACACACAACCGACTCTGGTTGCTCATCGTTTCC
CCTCACGAGGTTCCTGCAAACCGTGGACCTTCTGCTATATCTCCTCTCAG
>lmaj_32_24_up with AA060780 499421, 499822
TTGGCTGCCGTCGCGGGTGCGTGAGTATTTGCGTGAAAAGGTAAGTCCTGTTGTAGAGAGAGACCCGCCG
AGCACAAAACTCCAGAGATGCAGCACCAATGGCTGAGAACGCAGTCTAGTATGCATGTGTAGGGAGTGCG
TACCTCTGCCCATCCGTGTACAATTACAACGGCTCGCATGCCTGGTGTAGAGTTGAGGGATGTTCCCCGT
TTCTCCTGCGTGCACGAGGGCGATGTTGCCACTCATGCGAACACCCGCACGCTCTCGCGATTGTCGCGTT
TTCATTCTCACTGGCCCCTTCCTCCCCCCTCTCCCTCTAAACATGTTTTTTGCCACTTGACGCAAGTCAA
AGGACTGTTTCGGTTCTGGCGCACACGCGATGTCATCAGACATATCGCAG
>lmaj_32_25_up with W88280 1383058, 1383458
TCGAAAATATGTGACTGTGGTGATCTATTCCTCAACTGCTGTTATCTCCGTTTGTTCTTTCTGTAGAGAG
ACTCAAAGATAGTTTTTATTCGCCTTTGTTGTCCCTCTCTCTCCTCGCCTTGTGTTAGTCATGGAGCCGA
AAAAAAAAAAACACATACTTTCAATGTTAAGCACTTCTCTTCGTTTCTCAGGTGTGGGATTTTTCGTCAT
CTCATCTGCTTGGCTGGGGGTTATTGTAAGGCCACCCGTAGCTCTTCTCTGAGTTTTGCAAGGGTACTCA
TCGTACCAAAAAAAAAATACGATGCGCTCCAGGTCGGTTTCTGAAGTCACTAATCGTATTACTTCATCCTA
TGCATCATTATCTTCCTTGGCCAATATTCATGCGTTCTAGAGTTTCTAAG
>lmaj_32_2_up with T93413 425735, 426135
TGTGCATGGTCGTTCTTTGAAATCACCACTTGCTGCCTTTGTTGCATTTAAGGTCAAGAGCTCCTGTTGT
TAATGACCCGGGTTTTTGTTTGCGTCTACAGATCGACTCTAGATACCTCTTCCCTCTTTTTCTTCTTCGT
TGATGTATGCATACGCGCGCTAAATCTGCTCTGGACATTCGCGGCAAACTGTGCCTTGCGGTGCGCCGTG
GTCACATGGTTATCGGCCGTCTCGGTCTCTCTGTGTTTGACACGCTACCCCTAACGATGCGTCTGCGTTG
TTTATAATTGGCATGTATGTGGGACATTCTGTGACGCACACTGGATTCGCACATACCCACGTTCGACCAC
ACGCACAAACACACACACATACGACACTCTTTGCAACTTGCCGGGCATAG
>lmaj_32_5_up with T67379 811705, 812105
TGTGTGTGAGCGACTAGCGTTGACTTGGTGACCTGCGCCACGCCCTATCCTCTTTCACGAGTGGTTGCCC
CCTCCCCCCTTAGCAACAGTTCAGAGTCTGGGGTGTGGTGTTGACAGTTTCCCGTGTCGCTGAGGATGAT
CCGGCTTCTCGGTTGCCGGTCTCTGTTGTGTATCTCTGTGCGTCTTTGCCACTGCTCTTGCTGATCACGG
ATGGCAAGTGACAGTCGACGAAGGCAGGAGCTGGAAAACAAAGCCTTTCCTTTTGTTCTCTGCTCGTCCA
GCACCGCGCGCACGCACATTTTTCTGACGTACATAACGTCGCTCTCCACTACCCCTCCAGCTGGCACCAC

TTTCCTTCTCCGGATCGCTTTTTCTCATGCCTGTCGTGAGTCGCTGGCAG
>lmaj_32_6_up with AA060760 1386218, 1386618
GTCTGGCGCCGATCGCGCAGCACGTATCCGAGGAAGGACGAGAGGTGCCCAGTAGAAGCATGGCTCAATG
CAGCGACAGGACGGCTTGTCCTAGACGAGCAGGGCTGAGCCGTGGCGCAGCAGAGGTAGGCGAACTGCGA
TTGTGTGTGCGCATAGTTCTTGACTTGCTCGTGGCAGAATGGTCACGTGGACGAAAGAAAATTATTATTT
GGTGCAGCAGTGGTGCTGTGACTGTGGTTATGCAAAGGGGATGGCGTGCTCGCTTGGAGCTCATGCCTTA
GCAGTTATGTGCCGGTCGGAGGATGCTTTTATGCACGTCACTGTCGTCTCGTAACTATCCCCCGTTTTTT
TCTGTCGACGACTGACGTGGTTCACATGCGCCACTGATATGCTTTTGTAG
>lmaj_32_7_up with W88327 1507852, 1508252
CTTACTCTAAAGAAAGCGATAAAATGAAGAACAGAACGTATCTGGCGCCTGTGGAGATGTGCCATCCTCT
TCACGTTGCGCCACTGTGACATCCTGCCCCCCCCCCCCGCACACACACACGGACAAGTGAGTGTGCTTGT
GTGTACAGATGCTCTGCCGCCTTTGGTGGCTCCCGCTCTTGTTGTCCTTTTGCTCTGCCGCGTTCACGGG
CGGCACAAAACGCGACGCCTGCGTTCCGATGCCTTCCTGCCAACTTTGCTTCTTTTTTATTTCTGCACTT
CCGATCCTCTGTGCCCATTCGCGTTCTCGACTTGCCCGTTTCTTCCGCCCTCTTTTCATCCCCTCTCACA
CGCTCTCCTCTACGCGGTTTGGTGTTGCGGGCGGGGACCGGCTGCCGCAG
>lmaj_33_10_up with W88365 265496, 265897
CTACGGTGGCACAGCGCACCAAGAGAGAGAGAGAGATCGCTGCTCAGGGGAGACTGATGGCAAAACCGTA
GTCAATAAAGACACCTGCTAATGTCTTATAGTGACTATAAGATCGCGCATCGTTTCGGCGCCGCTTGCCA
AATCCGCATTACTCAAGTCGATCGGGAGACATGTATCGGTTCGCATTGCGCTCCATGAAACCCCCCCCTG
AAGTTCTCAACGTTCCTCCTCTGCGCGCCATGGCGAAAGGCGCATGTGCGCTAGCTAGACCCTCGCCGCG
GTACACGCAAATACATGTGAAGCAGCCCCCACAGCTCTGATGGGCTGCATTGAACTTTTCCCTATTATCC
TTTCTCCCTTCTTTCTTTTTCATGATCGGTTGCATTCTTCCACCGAAAAG
>lmaj_33_11_up with AA060742 1561256, 1561657
GCTTATGCACCACCACTGTTGTTGTTTGCGCTCCTTCCTGTCGCATAGCACTTTTCACACACAGATCGCG
TCATAGACACACCAGCATAAGGCGTACATGTATAGGCGCATTCGCAGCGGGCCTTTGGCACGCCATGCAG
TCACGCGCACGCTCACGGATACGTTGCTGCAAAGAGGACACTCGCGGCACAAACACACACACACACTTTC
AGCGTGTGCTCTCTTAGGCTCAGCGTGCAAGCGCCTCGCATGGTTTCTGCACACTCTGCCTCTCCAGATG
GAAAGATGAGCGTGACAGCGACCAAGGCGGTGCCGTCGCGCACTTGAACATCCTGGACTCTCTTCGATCT
GCTACTGCCCATCTCTCTCGCCCTTCGCGCCTTTGTTTTGCCCGTTTCAG
>lmaj_33_12_up with T93463 1353502, 1353903
CCCTCGACGTATCTAAGTACACACGTATGGCGCCCATACGCATTCTGTGCGATCTTACGTGTGCCCGGGG
TGCACCCAAAGGATGATGAGGTTCATCGTCAGCGTTGAACACGGCCAGAACGACTCACTGCGTCAGGAAC
CACGAGCTCACACGTAGTGGAGGAGGTCCGCTGGATTGAGCGCGCTCAAGCATCGCAGCGTTTCCGGAGT
ACAATGCCTTACATCTTTCTGCCGACTTGTAGCTCTCATCTTCGCCGCTTTTTTTTTCTTTCGTGCCGC
GTGGCTCTTTCTCCGTCTCTCGCTTTCGCCTTCTTACCCAACGCACATCCGACATGCCGCATGCTCATCG
TTCCTTTTGAGCGTTCGGCGACGTTTACGTCGTATCCTTCGACCGAGCAG
>lmaj_33_13_up with AA060741 818059, 818460
ACATCCTTATACGCAGGCACGGCACGGATCATCTCCGGATGAACAACGTTATTGAAAATGCCCTGCGGGA
GACACCCATCACACTTGTGCTCTGCGGGCTGATGGGGATGCTAGGCGTTATGTCGTCGTTGGAGGTGATT
CATCCTCTCTACTTACTGCTAAGTCCGTCGTTAGTGTTCAAAGAGCATCAGTACTGGCGCCTGGTCACCA
ACTTCCTCTACTTTGGTCCAATCAGCGCTCACTGCATCATGGAAATTCAGTGGATCTACCTCGTGAGCTC
TTACTTGGAAGCGCAGTATTACCACAGGCGACCGCTTGACTACATCTTCCTACTCTTGATCATCGGCTGC
TCGCTTTTGGGCCTGCGGTTTTCGTCGATTGTGAATGTGCCATACCTAAG
>lmaj_33_14_up with H64182 1470778, 1471178
TGCACTACTGACATCGGTGGGCTAGCGATGCGCGGGAGCAGCGTGAATCATGCAGACGAGGGCAGTCGAT

AGCAAGCTCAGATGCACAGGGCTCCTGTGAGTAAGTCCCCGACTTACTCACACACCTTTGTTTTGTGCCC
TCTTCCCCACGCTGCAAGCGTACTTACAGATAAGCCATGGAGGAAGCATATAGCGATGCGATATCGAAGT
CCCTAAGATACCGGAAAACGAAAAGCAAAACGTCATGCGTCGTGTGAACTGGCGCAGGGGTGCACAACGG
CGGCAGGGGAGGGAGGGGAAGTTGAAGATGTGGTTTATCCTTGCTTGAGGCAGCTGCTCGTGTCAATCAT
CCGCCTACGGATCTCTCCTCTTTATCTTTCGTGGCTGTGCTGCCCTACAG
>lmaj_33_15_up with AA125636 624851, 625252
GTGGCCTCCACCTCCACGGCAGTAGTGACCGGAACCTACGTGGGTTGTTCTAACGACGTGTGTTCGTGCT
CCTCCCTCTCTCTCTCGCGGCCCTTCGTGGGCTGTTGATCGCAGCCTAGAGTGAGGTCTGCTCCATGCTA
GGGGCTGCCACACGTTGATGAGGTGGGGCTTGTGCACATGCCAGCGTGTGTGTGTGTGTGTGTGTGTGTG
TCTCCCTCTCTGCACCTCGAAGGCGAATGCGCTCGCGCATCCTCAAAAGCCATTGCTTACACACGCACAC
AACCCTACTGTATTCTCCTTGAACTCTAACTCCTTCTCTCCCTGCATTCTCTTTCCTCCTTTGCGGTGTC
TCGCATATTTTGTTGTCGCTCGCTTTGTGTTGTTGTTTGAAACCTGTGAG
>lmaj_33_16_up with T93384 1545680, 1546081
CACAGACTCGGATGCTGTCAGGTGTGGCGACCTTCCAACCACTTCTCGCTCTACCAGCTCCATTCGAAGA
TAACGTAATGTGTGTACCGAATAAGCCGAAGGCTGCTTGAGGTTGGAAGAGGAAACACACGCGAAGACGT
GCTCAAGCGAGAGCCTTCGTTGCCGGTAGTCATTCAGAGTCGTCTTTGCAATCAAAGGAGACGAAATGGC
GACGGGCAGGCACTGGGGTGAAGGTGTGTGCGCAGGATGCAGCTGTTGACGTGGCCCACTTCTCTCTCGC
TCTTTCTGTGCTGGTGAATGCAGCAAGGCCAACACGGCAGCAGCACCTCGAAGGTTATTCCTCTCCCTCC
CGCTTATTTCAATCCCTCTCCTTCCCGCCTTCTCTTTGTGTGCATGCAG
>lmaj_33_17_up with W88363 605210, 605611
GGACAATGCTGGACAGGATCGATTACAATGTAGACAAAGCATTGGTGAGCGCATCGAAAGCACAGATCGA
ACTGGAAAGAGCTCGCGAGAATCAGAGCAGCTGCATTCTTATGTGACTTCCGCCCGTCCTACTTTTGCCT
ACCCCTGCTCAGCAAACTGCACCACCATTTTGTTCACTATCCGTGCTTTTTCCTCTCGTCGCACACACAA
AGAAAACAATACAAAGCAGGTCACTCCGTGCCATCGGTGCAACTCGATCATGCTCTCATCTGTGCCGTAT
CGTCGAACGAGTTTCTAGTTAGTTATTCTTCTTCGTGCTCCCCACTTTTCATTGCCCCTTTCTCTTGGCA
ACGTCATTGTCTTTGCATAACCTACAATTTGCTCTCCGCTACCGTCAAAG
>lmaj_33_2_up with T67349 872058, 872459
GCCGGGCCTTCTGAGCACTCGCTACAGCTTGCATGGACGGCACAGTATCCTCGGAGGCCCAGAGAATGCG
GCGACGGCGAGGTCGCTCCCATTCGCCCCTCACCCCCTCCCACGTCCGCACCCCTTCTCTCCCATCTCTA
CCTCCGACCTTGCCGGTGAGGGCTGGGATGGGGCGAGGTGGGAGGCCTGCTGCGTGCCTTTTCATGTTAG
TGAGAATCTATCTCCTCTCTCTTGTTTACTCCCCCTCGAACACACCCACACGTACACCTACACGAACAAA
TCTGCGCGTCATCACTTCCACACGTTGCTTGCGCCCGTCTCACCGTCGCGCACAACACAACTTCACCATG
CTCTCTGCTCGACAAACACCAACCTCCTGAACCTGTCACTTCTTTCGCAG
>lmaj_33_3_up with T93351 850610, 851011
CTCCAATAAGGACGAATGAAGCAGCAGACGGTATGCTTGAGGAGAGAGATAGAGGACACATCGTTGGCAG
AGCACTGGAGCAGGGGTGGGCTTCAAAGGCGGGCGATAAGGTCCCCCACAGCGCAACGGCGATGTTTTTG
TCGCTTCTTTGCACCGCCCCTTCCCTCTCCTCCTCCTCCTCCTGCTTTCTCTCTCCCAGACACAAGG
GTGTGAACTAAGGGCGCGCCTGGTCGTACGAGCGGACGGCACTGCCGCACTTCCTCTTCACCGTTGTCCA
CTCCTTTCGCGCACGGCCGCGGCGGCTTCTCCGTTGCGTTTCTATACTTCTTCCTTCCTCTCCTCGCCTT
TCCTTTCTGCCTGTCTCCTTGCCCACTGCTTCGCCTGAAATCGGCAAAAG
>lmaj_33_5_up with AA060782 1320269, 1320669
TCTATGTCAAGCAAGCATCAATGGCAAATATACGTGAAGAGCTGAATTTTGTGCGTGGTCTGAGGCTGCT
GCAGTCGTGGACTGAAATCATTGTTGAGTGCTCACCTGATAGTTGTCAGTGGGGAGTGAGTTGTTTTTGA
AATAGGACAGTGTAAGAGGCCACCAGAGCGTGGATGTTCTCTTCGGTTACCTGGCGCGCGGACATGCGAA
ACGGCAACGTAAACCTGTGTGTCGGTTTTCGTGTCTCTGCTTTCCAAAGCAGCTCACAGCTTTTAGTCTG

TATAGCCGCTTGTGGCTTCCATCTGGAGTCATTCTGAACGAAGACGATTTCTTTTTCGGTATTCACTCTC
TCTCTGTCTTGCTTTCTTGTCGCTTTATTCACTCTGTCGTCTCTTCACAG
>lmaj_33_9_up with T97247 267148, 267549
TGCAAAGAGCGATCATGAGCGCATCATCGTCGACATGCTGCGTGGAAGTTGCTCATCGTGGGTGAAAACG
TTGCAGTTTTTTAGATCATAAGCAATCACCGCGCAACATATTGGAAACCACCCTTATACACAGCGAGCAA
CTGATTGCTTTAGCGAAGCACAATTCACCAGCTGTCGTGTTTCTTATAAATGCCAAGAACTGTACTAGAA
GAAAATTGAAAAGGTAATGGCAGCAGCATTGGCGCTGTCGCTTTTTCTACCAGTCATGTTCTACGACGCC
TTGCTGACAACCACCAACAGAGCACTTGACCATGGCGTTCGTGAGTGTTCATGAGATGGCTCTTTCCCAC
TTTTCTCCCGCTTACGTTTCCCGCCTTTTTACTGATGTGATTGTGTCTAG
>lmaj_34_10_up with W88383 47532, 47933
TGAGTTGTCGTTCGTTCATCCGTTGGGGGAGAGCCGGAGTGCAACGCAAAGTGTCTGCCACACACACACA
CACACACACGCACGCGCAACGCTTTGGAAAGACTCGCGCACAGATGTCGCCACGACGCTGATGCGAAGGA
GTCGGTGTAGACGTGTGACAGGCCGGCGGCGGAAAACGGATGAAGATAAATGCTTGCTCCACACCTCATG
AGCACTGTGCATAGCCATCCAAGAGAGCGCTAAGAAGATGCTTTCCTTCGGAAGCACACAGGTGTTTGCC
GCTGCCTCCGTCTATCGGTGTAAGCGCCTGTGCTTCAGCATGTTTTATGCGGAAGCACTTGTGATGCGGG
CATGATCTCTTCCCCCCCCCTCTTTCTTTGTTTTGATCATCCCTCCATAG
>lmaj_34_11_up with W88257 1506991, 1507392
ACTGAGTAAAACCCGAAGAGACAAAACGACAAGGCGGCCTACTCTGCATGGCATGTCGGCTGTTTCTCTC
CGCCTTCCCCCTCTCTTCAGCCATCGGCCCGCACGCATGCGCACGGGAGCCGGTTTTGCCGCTGTAGAGG
TGCTTTAGTTGCTTTTTTGCTCTGCCGCAGTCGTTTTCAACTAGGGCCGAGGCGTGCCCGCTCGTGCCTG
AAGCCTTTTCTTTGCTCCCCATCTTGTGTACGTTTCCCATGCCACAAGACTTTTCTGGGCGTTGCTGTGG
CCCTGCCACACACACACACACACACACACCGGTAACGCGACCTGACTTTCCCGACTTCTCCCTCTCGCTTTT
TCCTGCTCCTCGTCTCTATATGAGCGGCCTCTCGGCCACCACACCAACAG
>lmaj_34_12_up with W88351 759971, 760371
GGTACGACACACCGCTACGGCGGCTGGTGCTCCTCCTCTTTTGTTGCGCGACCGTTTCCGGCACCCTTTT
ACGACTCCGCTAAGCGGTGTGCTTTTTTGTTGATATTTCCCCTCCCTGGCTGCCAGGACGCCATGCTGGT
TGTGGAGCGAGTCGTTCTCCCAATCCTGCGCCTCTTCTCATCCGCCTTATCCCTCCTCCCTCCATCATCG
TTATCGCCTCTTTCACGTTCTCGGTGTGGGCATACATGCGTCCGGCTGGCCTCTTCCTCTTTCTCATCCG
CTTGCGCACATGCGGCTCCCCACATTCGTGATGACCTCTCTCTCCCCCCACACCCCTCACACGCGATCAC
CAACTCGCGACCCTTGCTTACATTCGCCACCATCGACCACGCGACGAAAG
>lmaj_34_1_up with W88295 194008, 194409
TAGGGGGTCCATCTCACTGCGGTACTCCATGTGAAATAAACAAAGGCATGAATGCGCACGTGGCTTAGAG
TTGTCTGCTGTGTTGTTGCTTAGGTAGCCTTGGCGGACATCGTCATGGCATCGGTGCTCGCTCATAAACA
GGAGAGACAGACCGGCACTGTGTGTACACACTTGTGGTTGGCCCACCTCTCCATCCATCTGCCTCTCTCG
TTGAGAAATACCGCTACCTTATGCAGAGCCCCTTTTGGTGCTTCACGGCACGTTGGTGCACGGCTCTGCT
CCCCTGAGCGCCCAACGCGTCGCACCTTCTCCACTCTCTCGCAAAGGCAGCCATGGATTCTGTCTCTCTC
TCTCTCTCTCGTTACTTCCGCCTTCGTCCGCAATCATCGCCCTCTGTCAG
>lmaj_34_2_up with T93357 867561, 867961
CCATGCGTAGTGTAGGACGAGCAACCAGAGCCAAACAAAGTCGCGGACCGGTGAGAGCTATTGCTGTTCC
GTGGACCGACCTGGTGTGCGTGAAAAGCCGGCCTGCGTGGCCTCTGTGGTCCTCTTTCCAGCCCCGATGG
ACTGCTGGGGGGGGGACAGCTGTGAGCGCCTGCGTGGCCCTGCCTTTTTTCGTTTTTGCGATGCTTCTTT
TCCTCCGTCTCTGCCCCACACCCTTCTCCCGCACCCTCGTATTCTCCCTCTGTTCCTCTGTCTCTCGGCA
TTGTTGCGGTGTTCACGAGCGTGCTTTCGTGTGGCGGTCTTTGTGGGGTTGCCCACGCACCTCCTCTTGC
CTGCTCGTGGTGGCACGACCACCGCCCTTTCCCGCTATCCTGCTTCTTAG
>lmaj_34_3_up with W88347 49052, 49453

GGTTCGCGTGCGCGGCTATATTTTTATTTTCTTGTTTTCTTGTGTTGAGGCTGTATACCAAACACACACA
CACAAAAAAAAACAACGCACCGCGAAGAGAAGTAAAAGGGGATCGACAATGACCTGTGCGACAGACGACA
CACAGACTGCGTCGTGTGCTCTTCTGCTTGCTGCGTGGCCGCCCTTCCCCCTTGTCGTCTTCTCAGCCGC
CACCGCTCCCTGTTCTCACTATCTGTCTGTCTCGCTCTCTGCGAGCTATCCGTCGGCGTATTTCGATCAC
TCTTTCCTATTATGTTCTTGCACATTAGTACACAAGCGCTCTGTGCTTTGTAGTTATCATCTTCGCGTCT
GGCGTGTTGAACATCTCCTGTCCTCGCCGATGACCTCCACCACCGCACAG
>lmaj_34_4_up with W88281 738051, 738451
ACTGCGCAGGTGAGTGCATGTGTGCGTGTCTGTGCCACTCCCTCGCGTTCACCGCGAGGGAAGGCGAGCT
GGATGGGCACTGCAATGGAACTGTGGTGGCGGTGTAGGTGACTCGAAAAACTGTGAGAGGCGTGACAAAA
TGCATCCTTTTTATCCAAGTGCTCGGTGAAACGATCGAGCCAAAACCGCTTGTACGCTCATCATCCCTCC
CTTTCTCCTGCAGGGAGAAGCACCTTTGGGTTGCTGTGCCGTCTCCATGCGGGAACGATGTTGTCTGTCG
CTTCATCGTCCGCCACCTCCACCATGTCCCTTGCCTCTGTTTCCCACACGAGTGTCGCGTTCTGATTTTC
TGCTTCTGTAATCTTTCTCCCGCCCTCTGTCGAGTTGTGACACGGTGAAG
>lmaj_34_5_up with T93475 425889, 426290
TTGCAATCCTAGTAGATTGTGTCCCTCTCTTCTTCCTTCGCACCCCTCCTTTGACCTGCACCCGCGAATC
CAACCACTGAGGAGCGAGTGGCCAGCTCATGCATCCATGTTCCTTAGCTTCCGCCACCTCGAGCTGACTA
CTTATCCAGCTTCTCATGCCGATACACAGCCAAGCGCTAGCTTCTCGCTTTTATTTTCTGCGCCCTCTCT
CCGCTCCGCTCCTCCGATGACCTGCATTACCCTGGCTATCAAAACTCAACCATGAAATGTAACACTTATA
CTTGTATGCACGCCTTTTTATTTTGACATCGTTTTCGTTGCTGCTCCGAACGCGCTTTGGGATTACGGAA
ACATGCACGCACACATGCATGCATGCATGCCTATACACACGCCCACAAAG
>lmaj_34_6_up with T67400 1635313, 1635714
TCATGGCGATCGCGATGCGCGACACTGCGTCAGCGGCGCTGTTTGTTGGAGGATTTCTTGCGGTCTTTGG
CAGGGCTTGCCTCCTTCTTGCCTTTCGTGCGCCGACCACCTCTCTCTCTGCTCCCTCGCATAGGGGGAAT
GACGTGCATACAATGCTGCCTTCGTCGTCTACTTCGCTCCGCTCTCTTCGCATCCATTTGTGGTTGCTGC
GTGCTTCTCCGCGTTTGCAAGAGCGAAAAGGACCTTCCCCCTAGAACAGCTGCACTGAGTTGGATCAGCA
ATCGCGAGAAAAGTGGCACGACGATAACCGTACCAGTGGGCGTCCTTGTGTTTCTGTGCAGAGGTCGGCC
TGCTCCACCCGCTTCACTGCTCTTTTTTGTTCCCTCCTCATCTATTTCAG
>lmaj_34_7_up with AA060747 1557685, 1558086
ACTGCACTGTCCCCGTGTCCTCGACCTCTTGCGTTCTTATCAAAAGACGAGAAACACGCTATCGCCACCG
ATGCAGGTGTGCTACCACACTGGAGAGTGAGTGAAGCACGAGAGTGGGAAGGTGCAGCGCAACCGTTGCC
CCGATTTCTCCTTCTAATGCTTACACAGTTTGCTTTTCTTTCCCGAGCCGTTTGTCGGTGCCCGTTGAGG
AGCCACTTGCGCGGCTATGCATGCTGCTATCCAGTCGCCACATCATCGTTCGGCCATACCGTGCCCTGTC
TTTTTGTTGTTCTCCGACTATGCGCACATCAACACTCCGTACCTGCCCTTCAACACTGCTACTGAAAACA
ACACAAACGCCCGTTCTCTGCCCCCTTTTCGCCCACGCACGTACGCGCAG
>lmaj_34_8_up with AA125623 938279, 938679
TGCGAGTTATCGCTGCACCTGCCCAGCCCACCGCGCGTTCTTCTTCGGCTTTTCCACAAACACGCTCTCT
CTATTTGTCGCTGTGTTTTGCACTGCGGCCTTGATCCCGGGTACTCGCGTTTTCGTTGCTGCTGTCGTCG
CTGCGTTCCGCTTCCTTCGACGTGGGGATGGGCGCCTGCTCCTATACGCTGACCTGACGATGTGCGTACA
AGCACATCTCCCTGGGTGCTGTCGCTGCTAGGAAAGTGGTTGTAGGACTGCGTATTTGTCCCCATAGCTT
TACCCTGTGTAGCTCCACCTCTTCTCACCTAGCTGCGCTCTCGCACCTGTGCATGTTGAGTTTCTGTCAT
CGCCCACCCACCCCTCCCCCCCCTCCTGCCCCGCTTTTTTCTCTATTCAG
>lmaj_34_9_up with T93388 1213993, 1214393
ACAGGAGAGAGGTGCGTGCTCATATGGCTGACCAGCAACGAGCACACGAGACGATGCTGCGAGGTCGAGT
CTTGTGTATTCGAGGACCACGTGCTTTCTCTGGTTCGTTTCTTTCTCGTTAGGTTTGTGCTCATCAGTGG
TCTGGTGAAATGCGCTTCTCTTCAACGAGTGATGCATCATTTTCCTTCACGCCAAGGCCACAAAGCAAGG

CGGTTGATACACGGACTTGTACTTGCCTCCTCCGACGCACGCGCACGCGCTGCAGGCGAAGCTGACGGAT
AAGATGCTGATTCCGCGGACCAGGGTATCAGTCCTCGCGTCTCTCTCCATTTTCCCTCTACCTCACGGCA
ACTCTTTTTTTCTTGCTGGTGCTCTCATACCTCTCGCCTGTCACCCCAAG
>lmaj_35_11_up with AA060750 880634, 881035
CAACTTGATTGCAACCACAGAAATTACCGCCATTACAAATTTTTACAATAAAGACCAATGGGACCGCAGA
TGAACGCTTGTTTGAAGACAGCGAAGAGTATTGAAATGCGTGTCTGTCAATCTGATGTTGCTGTGTGCGG
AACACTTTTGCGTGCCATGGAGGACTGTGCGGCTTCGCGGAGGCAGCATGCATAGTTCTGTTTCCTTTTC
TGCGCAGCATCCAGTAACTGGGGGCTACACAAGGACGAACTCGACCTTGCAAACGCTTCTAAGCGACAGT
CATATTCTCCCTTCGTTTTTCTTTCCTTTGTGTCCTGTGCTGCACTTGATGCTCTCGTATCAACCGAATA
CACACGCACAAATCTTTTTTGTTCTGTGAGTCGTCTTCGACGCGCCTCAG
>lmaj_35_15_up with AA125648 752374, 752774
AGGAGGGGGAGGAACACTTGCGCATGTGCAAGGCTATTCATAGGTGGTGTCGACTACCGAAACAGGAAAA
GCAAAGGAAGGAAGATCGAGGGAGGTGTAGATCGCACCGTTGCTCTTGCGGTACTCGCGCTGTTTTTCTG
TGCACCAGCTACTCTGGTCTGCGCAACTGGTCAATGTGGATACTTGCATAGCTAGGACCCCAGAGGAGGG
TCATGGGGATGTGGGCTGTAGCTAGCAGGCACGTCGGCCATGCCCCGGTGAAAATCTCTCCGCATCCTGT
ATTTGCGTATTGTGACTTCCTCATCCTGCACCAAGCACTTCCGCATGCCACTCTTCGGCTGTTTCTCTCC
GCCTGTTGCCCTCTGCTTCGTTGCGGTCTCCTCGTTTTTTTTTTTGGTAG
>lmaj_35_17_up with W88414 572268, 572669
CCTCCCCACACAGAGTGGTTGTGCTGGACCCTCAGATGCCATACTGAGGTGTCCCCCGTCATGAGGAGCT
GATGAAAAACACAAAAAAGATGAAGGCGGGGGAGGGGAAAGGCAGAGGAGGCGCGTCGGAGAGGGTGACA
TGGACTGACGTCGCCGTATGCTCCGGTATATTGCTTTCCCAGAGCCCCCCCAGCTCCCAACCTTTTCCTT
CCTCGTCTTTCCTAGATCCTCCAAACTTCGTCCGCCCTTCTCCATGTTTCCCTCTCCCTTCGCCTCTTTC
GTGCTTATTTTTTTACTCCCTGCGCGCGTTTCTCACCCATCGATCACCACCACGCGTTATGCTTGCTCTT
ACGTCGCCACGCCCCTCCTCCGACACCACCCTACTTGTTTTGTTGGCCAG
>lmaj_35_18_up with T93457 780958, 781359
CGGGGGAGGCGTGCCCGTTTCGGCTCCTCTTCTCTGCTTCTGCCCCTGTGCACCACTTGTACGCTGGCAC
GCGAGTGCAGGCGGTTCGGTGTTCTCGTGATTTGTCACCGCTGTTCCTCCTCCTTTAACTCTTTCCTTAC
CCTCATTCTCTCTATCGCTGCATCGTGATGCTTTGTTTCCCCTCCACTCCTCGCCGTCGCTATCACTGCA
CTCTACGCGCACCTACACACATGCGTATCTTGTACGCTCACCCACCTGGCGTTACCGGCGTGCCGCCTCA
TCGCCGCAACCTCCGCTGCGTCCGTGCGCTCCGCTGGACTGGCTCTGCAACGAACTGTCATCATCGACCC
CCCCCCCCCCCCAAAAAAAAAAAAAAACGAACTACAACACTTCGCCAAG
>lmaj_35_19_up with T67394 1853567, 1853968
AAAGGCAAAGCGGAGCTGGTCGCAGGGAAAGGCAACCAAGGCCCAGGATCAGCGCCTCGCCGGGGCGGGG
GGCGCACATCCGCCAGCCGACCGAAACCCGTCGCCGAAAATGCGCCGCACACAAAAGCACGCGCATGTTT
TTTTTTTTTGCCTGAAGCTAGGTGTAACCGGAACGATCTGCCGAGGACCGATCTCATTCACACAGCAGCT
AAGAGTCCTAAAAACAAACGTAAAAGCGCCTCCAAGCATCAGCCAAACACCATGATGCACAGCTTTTCGC
ATATTGGCAAGTGTCAGTCGCACGCAGAAACTGTGTTTCGAACTGCCGAAGTTTTCTCTATTCTCTGCTC
CATTTTCCCTTCTCTCCACACACGCTCTTCTGCAAACGTCCTGCTTACAG
>lmaj_35_1_up with AA125620 1744610, 1745011
TCCCTTGTCGTTCAAAGTTTGCCATCTTGCTGTCTATGATGTACAGACAATAGTGGCGTGCAACATCCTC
TCACCACCGCTGAGAAAGTCGAAAGGCATGCATGCGTGCCTCGCAGCCAGACATGCGGAGACGTCAAGCT
CTGCACACATGGGGGCGCTCGCCTCGCCTTGGAAGTGCGTAGCAGAGAGATACCGAACGTGCGTGCCAGA
TCCGCGACGGAGATTGTCAGAGCACTGCGCTGCGCACTGCGCTCCGAAAGGCAAGAAGACGGTTTCCGTC
TGCCACAAGTGTTGCATGGGCACGCAAAGAAAAAGAAAGCAAATGGCAGCGCAACCGCCGACACGCATGC
GAACTTCTTTCCCCTCCACTTTCTCTTGTCGTCTTGCCTCATTGCGTCAG

>lmaj_35_20_up with AA125630 594130, 594531
CCCACAGCTGCTCTCGCTTGTTGGCTCGACCCCACGCGGTGACACATGAGACCTTTGGTCAAATCTGCAA
AGTATGGCCAGCGCGAAGGTAAAGCGGTGGTGTGGTTGTTGTCTTGGTTGCCGGATGGCCGGTAGTACTG
CTACCCGAGCCATTCGGGGAGCAGTACTACGCAACTCCCGTGCTTCTGCGTCTCCTCCCTCTGTGCACCC
CCTCGAACTCACGCTCGTCGGCTTCACCTGGAACGTCACTCGCCGCCACCTCCTCAGCTGTGATGGTCCC
TGTGCACTGCTCATATGATACCCTCCTCTCCTCCGCTTCCTTTTTCTCGCGTGTGCGCCTCTCTCCCTTC
TCCCCCAAAATAATATACACTCGCTTGCGCACACGCACAAACCATTGCAG
>lmaj_35_21_up with AA060753 1638732, 1639133
ATCACCGAAAGGACCCTCCAGCGAAAAGGATCCGTATAGGTGCGGTTCGCCTCTGTGAACGCGAAGTCGG
GTGTGCTATTCATGCTCCACCCCTCTCACCCCACTGCAACCATGCCGCTTTCTCCACTCCCTCAAAGCTG
AATCCAACTGTCGTTGGCTCGCTGCAGCTGTCAGAGGACGCGTGGGAATGGGGAGGGAGGGAGGGAGGGG
GGAAGGGGGGCGGCCATCGGCTGCAGCACGGCGCACCGCCTCACCATTCGATGATCGTCTCGGCTCTTTT
CCTCCTCAAACCCTCTCTCTTTCCCATATTCGTTTTCGCTTGCTTCTCTACACGACTTGTTGGTCCTCTC
GAGCATGAGGTACCCCTTCCATCCATCTGGGCACACTCTGCTAACACCAG
>lmaj_35_22_up with T93458 1714365, 1714766
ATACGACCGCGTGTGTGCGGTAGAAAATAGCCACACCTCGAACCGGAAAACGAAAAGACGAACAGAGTAC
TAGAATGCTGAAGGGGCTTCTTGGTTACCTGACGAAGGACTTGTGCGCGCAAGTGCTCGCGGGTCGTTTT
TCTCTTAATCGCGAGACAATGACAAGAGATACGCGCGCTGGAGGAGTCGGAGAGGGCAACCACGTACTAA
CACCGGCGCTTGCCATTTATACCGTGGCCACCGTGCACGCTGTTGCGACAGCGGTCTACACGCTCTTTGA
TGTTTTTGGAAACAGGGCAGCTTTGTCGCCCCCTCCCCCCCTTTCGGACGGTTTGACTACTCCGCTTCTT
CTACAGACACTCGCTCAATGCTTACTCTTCGTCTCTCCTCGCATGCCAAG
>lmaj_35_23_up with T67337 561306, 561706
TGTTTGATCAAAGCTTACCATGGCAGGGTCGTCGACGGGCGCAACTGCTTCTCTGCCCGTCCTTGGTGAG
CGCGCATTACGCGTGAATCGCACGCCTTGCGCTGGGTTCATCTTTCGACCACGCCGCTACCTACAAGCTT
GTTTTGGCCTGGACGAAAACAGCTGGGCTCTGAGTCTGCACATGATGCTCACAACTCTTTCCCACTCTCT
CCTGCACCTGACGCATCACGTCGTTTGCCGCCTCATCTTTTGCGTGATGTGCCTTGTGGTTCTCACGCTC
TCGGCCTGTTGCTGCACTGTTTACTCAGTATCTATTCTACCTCACACGTCCAGACACCTCAACCTACCCC
ATCTCTCTCCCCTCGCCGCGTACACTCCACTTCTCTCTCACACACTCAAG
>lmaj_35_25_up with T97246 850355, 850756
TGTAAGAACAGCAGCAGTGGCAACAATACTGCGCCTCCTCGCTCCTCTAACAAGACCTTTCGACCTGAAA
ACGTGCGCGCAGCCGCGTTAGTGAGCGACTGTGCTGTGGTGGTGCCACCATTTGTATGCGTACCGGGGAG
GAAAAGACGTGTGATGCGTTACCGCCCACATCATTGACCCCGAGCTGCCATGAACCTGTCCAGAGCTGTG
GCACACGAATGTGTTGCGGTGCGACCGGATAGCAGCGAGAGAAGGCGAAGACCAGCACAACCACAAAAGC
AGCTGCTCCTATGATTGCCCACTCTTCTGTTTTTCTTTCTTTTTGCTCAACCGTTTCACGTTTCCTTCTC
TTAATGGTGTCGATGACATCCCTCCACGCTCCACACGCACATGCACACAG
>lmaj_35_26_up with W88407 1355308, 1355708
GGCTTTCTGTTTTCTTTGAGTCTCCATTGCTACCAGCTTCCTTTGAAGCGGCAGGTGGTGAGGGAGGGGT
ATAACAGAGCGAACTTGCTGCAACGCATGCTGTTTTCTGTACAAGGTTATTGACAGCTGCTGTGAGGTGG
CTCGCTGAGCTACTTTTTCGTCCCGTCTGTATCGTCGGTTTCCCCCATTGAGAGAAAAACATACTGATGT
GTGCCTGCTGGAAGTGCTGAAAGAGCTTGACAGTGTAACTGCTCTTCCTTGGAGTGTCTGCCTCGCTTTA
TACAAGCGTCTCGATGTGCTCTCTGCTTGCATCCCCATCCTTTTTGTACTCTTTTCTCTCTATAACATCT
TTGCCTACGTGTTCCTGCGCCATTGCGTTTCCTTTCATGGATCTTTTCAG
>lmaj_35_27_up with H64188 1854096, 1854497
GCTCGGGGCGAGGGCGGGTGAGTTTCAGCACATCTTGAACATGTCCAAAATTCCAGCCCAAGTGGAGCAA
AAGCTGCGTCCGGCGATGGATCCTGGTGAGCTCTTCACCTATCAAGATTCGCGCATTTTTGTGCGCGCTC

GCCCGATCGTCGCACATGATATGGAAGACCCAAACAATCAGAGCATTGTGCAGAAAGTCGAAGAAAACAG
AGACCTCGTCGTGATGACGCCAAAGATATCACTTTCTGGCGAGTGCACAATTGATCCGTGCGTTGTCAAT
CTTGACGGCGTGTTTGTGGGCGCCCGTGACGATACAGAGCGAGTTTACCTAGAGTCATGTGCCCCATTGG
TCTCTCTCTCTGTTAACGGCGCATCTACGTGCGTGCTCTGTTACGGGCAG
>lmaj_35_2_up with W88329 892958, 893359
AGAGCAGGCACGCGAATTTCCTTCGCGGAGGAGAACCCTGCAGACAGAAGAACAAGCAGGGCCTGTTTCA
CATAAGAGAAAGACATATGGGCGATGCGGCACCAACGGCGCCGTATGATATGCCGTGAAGAAGAAAACTA
GTGGAGATGAACACGGTTGTGGCACACCACTGGCAGGTTGTGCCTGCACCTCTGCTTCCTCTCCCGTCGC
TGGAGAGCTCTTCGTGGAAGGTGGTCACGTTTTAGTGGGGACTGCAGCTCCTCGGCCGCCCTTCCTTTTC
CCTCCTTCGTCTTGTTCGCGGACTCGACCTCTCCACCTTCTATCTCCTCCCTTGTGCTTCATGTGCCGTA
CACAACAAACAAAACCCATAAACAACACAAATGACGAAAACGTGGAGCAG
>lmaj_35_30_up with T67347 1409610, 1410010
CCACCCTCCCCCTCTGCCGCGCGCTGGCTGGGTAGAGGGAAAGATCGGGACGGGTCCTACGCGCTAGGCG
TCTGCATGTAACACGGCAGAAGACACTGAAAGGCTCGCGAGTGCCCATCTTTCAAGCAGCGAGCACAAGC
GTGTGTTCAAGCTCTTCGCAGCGGACGTGGCCGATGCGACCCTTTTTTCGTCGGAATCGTCAAGACGGCA
CATTTTCACGCTTATGTGCGGGCCTGGCCAACATCGCCTTCAGCTCGTGTCTACTCGCTCCTCCCCCCCC
CCTCCTACTCGAGCCCTCTCGCTTTCTCCTCTCTCTGACGTCCTTCTGAAACCCGCTCGCCCTACCTCGC
GCGTTGCCCCGCGCCCTGTGCAATGTGTCTCTCACACACGCACGCACAAG
>lmaj_35_3_up with W88381 64300, 64701
AAAAAAAATAATGCACCAGGATGCCCCCTCTCCCCCGTCGTACTCCGATGCTGCCGCGCATGACGCTCTT
TACTTAGGTGGTTGCCGCACCTGGCGTGACCGTGTTCTCCCACTTCATGAGAACAAAGAAAAACTAGCAG
AATACAGTTTTTCTGCTTGCGCATCACCGACGCGTTACCGACTAGTCGTCGAACTCACGCTCTCTCTCCA
ATCACGGAATAACACGCTGGCTCTATCACTCCGTATGTGCTCACTACCCGTCAACAATACGTCCCCCTTC
TCCATCTTGTATTCCATTGTTCATCCTTTTCCGGCACACATTTGCACTGTACCCTCCCTCACCGCTTTCA
TCCCCACCACCTTGAACACTTCTTCCCCCTCAAATGCACCCGTGCCGCAG
>lmaj_35_4_up with W88308 738502, 738903
AGGGAAATACTTGTACTCATAGAGGAGAGTGAGAAGCAAAGTATTCTAAGTCGCTGCATTTGACGAGTTT
TTTGTCACCAAGCTTGGCTCAGTGCTTCGGAATTCAGTCCCTGAATTACAGGAAAAAAACTAGCACCGAT
CCCGAGCACCAGCGTACAAAGTGTTGCGGTGGTATCTGAAACAAAAAAAAGCGTTGGAACGACTGCTCTG
CGTGTAGAAGTGGACCTTGGATTCCCGTGCTGACCTGCGTCCTCTCTCTTGTTCCCCCGTAGCGTTCCCT
GCGCGCGACGGGAGAAGAAATGCATTTATGTAACTGGCTTGATACTCATTCTCAGTCGAATAATAGCCTC
CTTTCTCGTTCTCCCCTGGCCTCACTTTTTGTTGCTCGGCTACACTTTAG
>lmaj_35_5_up with T67319 687613, 688013
CACCTGTGAAAAAGAAAAGACATGCTTTTTCCCGCGCTTTCTTTGTAATGATTCTGCTACGGTGGCCTTT
TTGTGTTGCCAATGCTTGAGGCTTTCACTGAAGTGCACGCGATCAAAGAGACGAAATCCCCCGAAAAAGG
TACAGAACGAAACAGCAACGCTTGACTGCTGCTCTCAGCAATCTGCTTCTCTGTGGGGGACTTCGCACTC
CGCTCAAAATGTTATCAAAAGGCTTTCTCATTTTCTCTTTCTGCTGTCTGTCTTCTTTCTACTCGTTATA
TTCCTGTGATACCTGATTTCACTGGGGAAATATATATATATATCGACACCATATCCTTCACAACTAACCA
CATTTTATTTCCTTTGTCCTCTGCTTCTTATTGGAAACGGTTTGCATCAG
>lmaj_35_6_up with T67312 2034078, 2034479
GGCCGCGTAACGTTCGGAAGGCTTTGAATATTCTTTGTGTGTATCCTACTAGCATATCCCCCAGCGTCGT
GTGCAGTGACGTGCCGGCGACTCTGCAGGAAAGGATGTGTATTTTTCTTCTGCCTCATTGCGTGCCACTG
AGTTCAGTCTCTCCCACCAACCAAAATAGTCCGTGCTTCGCCACGTAAAGCTCTGCAACAAAAAGTGGCT
GTTGCTTCAAAGAGCGCCAAAGATGTATGCGCGCACACCGCCGGCAAAAATCCCGGGTGAGAACCCCTCC
CTCTGTTCTCCCCTCCCCCTCGTTGGGCAGAACAACCGCCGCCCGCGTACTTTCTCTCTCTCTGTATGCC

GAGCCATGAGTGTGGCCAACTCTTTCCGTGATTCTCCTCTTGTGTTCAAG
>lmaj_35_8_up with T93462 47477, 47878
TTCGTCGAATTGCGTCATGCCTGTCGCCCACGGTAAATGCGTCAACAGGGGTAACATATGCCACTGTACA
CGCTGAATTGAAAACGAGATTTATTGCCCAAAGATGGCCAACTATGCGATGATAGGCGTTTTATGCACCG
CATAAAAAGAGTACTATCTTGCCAAGCGCTTATTGCTTTTTTTCCGACAGGGTTCGACGGCTGCCAAGAG
CAAACATCATATCTGGCATTTATCTTTTCCACTCATTTTTGCTGCGCTCTGGCTATGTTTTTATAGCAAA
GAGCCAAATTTTGGCCCGATTATCCCTCCACGAATCTTATCGAATAAGCTGTGGTTGTTTGAGCTCAAAT
TGATACCTTCCTCACTCCTTTCGCTTTTGAGTTCTCTTTGTTCAGCAAAG
>lmaj_35_9_up with W88404 1787009, 1787410
CCGCACGAAACGAGGGGCAGCACCAGCAGCCTAGTCTGCCTCGTTCCCACGGTCACCACTAACAACTCGC
CTCAGCTGCCGATGCTCATTCAGTCGCTGGGCTCTAGCACCATTAGCTTTCATGCCGACCCGGCCTTGCG
TACTCAGGAAACGCACGCTACGCCCCAGGCGATGCAGCAGGTGCTGACAACAGACTACATGCAGATCTCG
ATGGAGAAGCCCGTGCCTCCGTCGAAGTCCGCTACCGACCCAGCCGCCAAGACAAACGTGATGAGCTCTT
CTGAGGTGTGCCGTCACTACATCAATGGCCGCTGCAACCGCCGCAAGTGTCGTTTTCTTCACCCTGACCT
GCGTAGCCCGCTCATGCCCTCCCATGTGACCTATACGCCTTGCGATTCAG
>lmaj_36_10_up with AA125625 1728696, 1729097
CGCTGAACCCCAGAAATGACGACGGCGTAGGAAAGGAAGAGCTCTTCTTCGATTAAAAACAAACAGCAAA
TATTGACACGACAGCGTACTAGTCATGCGGATCATTGATACGGGGAAGTAGACTCGCCCATCGTAAGAGA
AAGTCAGAGCCTCCGCAGCTCTCGAAGTGTTCGCTGACGACTGCCGGTCGAGGATGGATAGCGTTGCACG
ATCTGTCTATTAGGGCCAAATCTTACGCTGCGTCTCTGATCATCGCCGCCTGCATTTCCGACGCGTGCCA
CTCCGTCCAAACGGTGGGGTGGTGGTGGTGGTGATGGAGCCAGCTTCCGGGCCACCATGTCGTGGCCTAT
CGGTGAGAACTCCACTCTTCGTCTCATCTTACCAATCGAGCATCCTACAG
>lmaj_36_11_up with T93468 345688, 346088
AGGTAAGAAGCGAAAGCACCGCACATCTTTTCTGTTGTTCTTTATGCTCTTTTCAACTGACCACGCGCCG
CTGGACGCCAAACAAGCAGACAGGGGCAGAGCCGCAGCGGAGAGCAAGATCGCTTCCATCGCAGGTCCTC
CGGCGTAAAGGACGACAGATGACGCCTCTCTCGCATGCGGTTCCGATCGACGTGTCGTCCGCCCCCCCCC
CCTCCAGTTTGGTGATCCGGGACTCACTGCGGGTTTCACGAGATTTTTTTCGTTTTCAGCGGCGACGATG
AGAGGATCGCCAGGTAAAAAAAAAAATGAGGGAAATGAAATGGGGACCATAAGCGGACTTGTCTGGGCTTT
CCCGAATGCGATTTCCGCGCCGCCTCCGTCTTTTTTTTTCTTCGCTCTAG
>lmaj_36_12_up with T93461 460724, 461125
AGGACGCTTTCAGAAATGACTGTGATACGTACAAGACACCATAATGGGAATTATGAAGCACGACTAAAAG
GGATACAGGAGTCCGCTGTTGCCTCTTGCAGTCTGCAGGCAGAGTTATCTGCTCGCTCAGCATACCGTCA
CAAAGAAACAAACACCCTGCGTACTTTGCTAACTAGAGGGCGATGGCACCGGAAGAGCAGCTCCTTTTTT
TTTGTTCCTCGCTCGACTGCCTCGCGCCGACAGGGTCAGAGAGGGCACATGCTGCCTTGAGTTGGCTCTT
TGTGCTCCACCGTCACCTACTCTCGCTGCTTCAACCTCGTCTTCTCGTGCGGGTGTATATATCGACCCTC
CCTGCTTCCGCCCCTCTCCTCCGCCTCATCCTCTCACATGAACCCGATAG
>lmaj_36_14_up with W88343 234160, 234560
TTTTCGCTCGTACTCGCCCCCCTCTCCGTGTCCCTCACACTCTCTGTGCCCATTGCTCTTTGTCGCTCTT
TTTGGGTGCTTCCCTTCGAGGAAGCGCACTGCCGCGCTGGCGTGAAGATCTCGTGGTCGCTCGCTTGGCG
CATGTGCTGTGATGTAGACTAGCACCGGCGGACAGGGCCTTTGAATGCGCTCATGCGGGCTAGGCGTTTT
CGAAACTTCCAGTACGGCCACCACTCACCGTTGCCGATGCGCTGTCACCTCGTCTCTCGGAGTGTCAGTG
GGTTGAAAATTCGGTGTTTTCCGCGTCTTCTTTGATTCCGTTCTCGTCAATTTCTTTCGGTGTTGCTGTG
TTCTCGATGGGATCATACGCCGACGCGCCGTGGGCCGCCCCGCTGGCAAG
>lmaj_36_15_up with T93378 469262, 469662
TACAGCCGAATGAGGCTGTGGAACGAGGCCAGGTGAAGGGCTGCGGCTATGCTGCGGCACCCTAATGTGC

ACGGGCGATGGCGTTTGCTGGCTTCTGTGGGAGCCCGACTCCCTCTTGTGGGGCCACCTTTGCGGTTTGC
GCCAGAGGTGTTGCGGCCTTTCTTTTCCTGCCCTCTGCCATTCTTTGCGGCCGCTCAAACGCATTGGGAG
GTGCACATCAGCCGGCTGTTTCACTCACGGGCGGCCCGCGCCCAGCAGTGCCCTGACTTTTCACTCTTCC
TTTCTCTCGACATGGATGAACGCCCCACGGGGTCCCTGCAGCACTCGGCCTCTGCTCAACACCGCTCGTG
ACTCTCTCTCTCTCTCTCTGTTTCCATTGTTTCCACTGTTTCTTCTGCAG
>lmaj_36_16_up with W88390 1443629, 1444030
TTGCCAGTCGGCACGCAGCCACATTCCAGCACCCCCCTCGAGTTTGTGTATCCGCGCATGAGTTGCGGCG
GTCGGGTGAGTGTTGGGTGGGTAGGCACACATTCACTGGAGGGCTCGGGAGGGGAAAGTGTGCACACAAG
ACAGACTGTGCCAAGCGCGTGTGTCGGCGCCTGCTTTTCACATTTTATCCTTCTCATCCCACTTTTGCGC
CTCCGCCCCTTGCTATTGCACCCTCACACGCCCCATGCCAAACACTCACGCACATCGGCGCCACACTCGC
TTCTCCTGCACCAATGGTACTCGCCGCTCTCTTATGCCTTCATTCTTTGGCTGGCTATTCCTCACCCCCT
TTCTCCATCATCACCATCACGCACGTCTCTCCGCTACCGAAACGAGGAAG
>lmaj_36_17_up with T67373 2655550, 2655950
CGGAGATGTGGTCGTACGAGGCTTGTTGCGTGAGGTGCTGCTGCGTTGCAGGATGCTTTTTTTGTGCGCG
CCAAGTGCGCTGGGCTGCGCTTTTTGGCTGTTCCACGAACGGTGCACTCTCCCGACGTTTCTTCTCTGTC
TGTCTTGCCCGGATGCTTTTCATATGCGATGTAGGCTCCCTCGGCGTCGCCGCATGTACGGTGTGATGTG
CGTGCGTACTCCTGGCCATCTCACCACTGCTATCTCCACCTGGTCACCCTTCTTCGCTTTTTCGTCGCTG
CGCACAAACCTGTTCCTTGGTGCTACCTCGCTCTTTGTGTTTTTTTTGCGTTCTGGACGGCCTCCCACTC
CGACGTGTGCTGTTACTTGTTTCACACTTGTCGCTGCGGTTGTGGGGTAG
>lmaj_36_18_up with W88255 1802885, 1803286
TTGCTGCCGAAGAAGTGTCTTTGCTATGAGCCCCTGTGCACCTGCGATCGGTGAAACCCTTGTTTCTGCT
TTTTGTGTGTATCTCGCGGGGGAGGGGGGAGGGAGATGCCCTTGGCGCTGCACCCTGTGTAGCGTACGCC
CCCCCCCCCTCTTTTTTTGTATCCGTGACGGCTGTGCAGCGAACCACGCGGTGCGAGATGAAAAAAGGCA
TCGATCACCATTCGTCGCCTTTTTGTTTGTGGCAAAGCGGTTTCGTCAGCATACCGCTCTCCCCCAGAAG
AACCAAAAGATGAACATGACTATGTGTGTTGCTCCCCCTCCATGACCGCTCGTCGCTCGGCACGGCAATT
TCATTCGTCGCTTCCTGCTCGACTCCCTCTTCTGTTTGCTCTTCTCCAAG
>lmaj_36_19_up with T67364 462157, 462558
GCCCCTAAGTTTGCTTGCGTGGTTGACTTTCAACGGCAAACGGCGACAGTAGCGTGACAGGCGCGGCGGA
AACGCAGACGCAGATATCGACACTAGGGATGCGGAGTCGTTGCCATCCCCAGGGGCGGCAGCATTTCTCT
TCGCGGCTTGTCATCCAGAAGAGCGTCCCTCCCTCAACAAGGTGTCGCTCTGTAAGTGTTTGTATGCTCC
GCGCGACCTCAAAGGACTGTAATCCGAGCGACCCTATCACCCACCGCACATGGGCCGCTCTGCGCCTCGG
CGTTTTATGTGCGCTTCCACTCTCCGCTTCACTGTTTCACCCACACGCCCCTCACCCCCCTCACCCCCCA
CACACACACACGCACCACGGCAACGTCCTACACCACGCGCGACTCCACAG
>lmaj_36_1_up with W88392 1415100, 1415501
CTCGATTGCACACACAGCGTCAGCGAGCAGAGGCGGAACTTTACGGCTTCGAGGATGAATATGATGATGA
GGCTCCTGCTGAAATTCAGCGAAAGTGAGCACAATGGGCTTATCCTTTTCTCCTAAGTCAATATGTATTT
TCTTTTGCTTCATGCAAGGAAGGAAGTAAAAAAAAAAAAGGAACGTTTATCGGGAAACAGGCACCGCGGCC
TGTGATGCTGCGCGCCCTCATGAGGTGATAAAAAGACCCAGTAATAACGCTTTTTTCGCCTGATCTAGTT
CAGCTGTTGCACATGCCGAGGTGGCAGGTAAAAGCTCGGCCCTCTTTACAGTCTCAAAAAAATGCAGCGT
CCCTGTCTCTTCTCCTTCCTCTTGCGGTTGATCGCGCTTTTTATGTGAAG
>lmaj_36_20_up with T67316 155814, 156215
GGGTGTGATACCGATTGCACTGAGAAAAGAGATAAGACCCCAGTGAGCTTGCAGCGAAGTCTTCATTGTC
CTTACAGAGCGCGGGCTTCGCCTTTCTGCTGTTGGGCAGCCCATCCCTTGCACATTCTCGCACCATCACT
AGCGAGAAGGGGTACTGCTTTTGTGATAAACAACGAAACCCTACGCACAAGAGGCTTCCCCTCTGAAGCG
CTCAAAAGCATCAGAATGACTGATCTTACTTCCACAGCGGACTGCACGTTGATTTTCAACGGAGCAAGAG

TCGTTGCAGTGCATACCGCTTCCGATTACCTGTTTCCCAGTGACGTTTCAACTCCCGTGCTTTTCTCTCG
TCTTACCTTGGTGTCTTTGGGTGAACTGTACTTGCGCACAACACATCTAG
>lmaj_36_21_up with AA125616 177654, 178055
CCCCGCGCAAAGCAAGAAGGTCAGAGAGCTGAGAGGCGGAGAGAGAGTGGGAGGCGTGTTCGGCGCACGC
ACTCCAGCAGACTCTCTCCTCTCCTAGGTGATGTAATGCTGCTTGGTGCGTCTGTCTTTAGCCAAGAAAA
GCGGAGCTGCCGGTGCACTACGAACTTGAGCTTGCTCTCATGATGTATGGTCTTCTCAGCCAGACGCGCC
CTGGCGCAACTGCTATTCGGCGGCGCAATGGCTTTGATGCATCTTTTAGCGATGGGAGCAGGCGCACACA
TGCATGCATCTCTCTCCGTGTCTCGTCTCGCCTCTCCCCTCCAATGTACCTTCCCCTCTCCACTGTGCCT
GATACGGTTTTGTCTCCCCCCCCCCACCCTTCCTCGGCCGCAACACACAG
>lmaj_36_2_up with AA060758 334261, 334661
CGGGTCATCAGCCGCGACCGAGGGAAAGCAGGCTATACTCGCCCCTGTTTTGAATGAATGCGCATGAACT
TGCGGTGGTGCCGAGGCGATGGGCTGGCGGTTCCTGTGTCGCCAGTTTGGTTGGCTGACAATCGTGTTTG
CGCACCCCTCCCCTGCGGCGCCCCCGCTTTCCCTTCCCTTCACTTACAGAGCCCTCGGTGAAGAAAGAAC
GGGCCTGCTCGGTCACGCGGCGATTCTGGTGTGACGTGCACGTGCTCTATCACTACTGTGGCAAGTAAGT
GCTTCTCCTGTGCTCCTCTTTTGCCTTATTCCCTCTTCGCAATGCCTTTTCGTTGTTCGTCACAACCACC
TTTTGGGTTTGGCCTGCTGCGTGCTGCGCTGTGTATACGTATCCCACTAG
>lmaj_36_3_up with T67372 708167, 708567
CGCGCGTGAACTAGCAGCTGAGCAGTTTGCCCACCCCCTCCTTTTGCGTTGCAAGGGCGTATTATCATCA
GTCGTCGATGAAGGATGTCCATCAGCTTCGACCGCAGCTGCAGCCCACTCTCATGACGAATGTGTGGGGA
GGGAGGCGCTGTGGCTGTGACGGAAGCCTCCTCATGCACACGAAGAGGGGCGGCTGGCATCATGTGCTCG
TCTCCTCTCTTGCAGGAAGGGCAGTACGCTCTGTCACGTCTCCGATTCTGACCTCGCAAGTACCAGCCTC
ATCACTTTCCCTTTGACTTGGTCTTCCACTCTACCTTACCTGCTCGTTTTGTGTGTATACCTCGCCCGTC
TCTCGCCCCTGAAACGGTGCCTGCGAGCTTGTTTCTCCTTGCTGAAACAG
>lmaj_36_4_up with W88336 1589803, 1590204
ATGGGCACGGGTGCCTGAAAAAAGCACAAGACAAGGTCAATGCACACGCGTTTGGAGAAAGAAGCAGCTG
GGGTAGGTGGAACAGGTGTGCTGCTTCTCTTTACACGGTTAGGTAGCCGTATTAACACCGAGGGTGTGCT
CCTGGGGTGAGCGAAATGTGTCCTTTCGAGCACCGCAGGCAGGGAGAAGGCTGCAATGCGTTCGCGCGAT
GGTGCCTTTTTGGCCGCCGCTTTCGCTTTCGCCTCCTCTCTCCCTCACTGCCGCTCTGACAGCACCCGAG
CACCAATCGCACGACCGTAGGCGTTACTTTAGCGGTCTTTTCGGTACACTGCATCACCCTACCCCCTTCT
GCCCTTTCTTTTCGTCTGTTGTCTCGCCGCACTTTTGCCTCCCTGCACAG
>lmaj_36_5_up with T93379 1413772, 1414173
ATTCCACACTGTGCAGTCGCTTCTCGAATCGCCCCTGTCCACTTTGGAAACACTTAATGAGGCAATTTCT
CTGAATGCATCAGAAGCGCATTCGCTGTAATAAATTGAGGTCGGGGTGAAAATGATTTGTTTCCCCCAGT
ACCGTAAAGAAGACTTGCACATGTGCCATCGAGTTGATCGTTGCAAAGGAACCGACACGCTCAAGCCCTG
TCAAAGGAGATATTCGCCTCCTTTTTTCTCATCCGTGTGTTCTGGCACTCATGTTTTCAGCGGCGGAGAG
GAAATCCGTTTTGCACATTGCCTGACTTTTGCATCCCTCTGAAAGAACTCAGTCGTGCTTCTTTGCGCTC
AAAGGCAATTTCCGCCCTTTGTTTGTCTCACCTCTTCTCCGGCCTAACAG
>lmaj_36_7_up with W88373 462175, 462576
GTGGTTGACTTTCAACGGCAAACGGCGACAGTAGCGTGACAGGCGCGGCGGAAACGCAGACGCAGATATC
GACACTAGGGATGCGGAGTCGTTGCCATCCCCAGGGGCGGCAGCATTTCTCTTCGCGGCTTGTCATCCAG
AAGAGCGTCCCTCCCTCAACAAGGTGTCGCTCTGTAAGTGTTTGTATGCTCCGCGCGACCTCAAAGGACT
GTAATCCGAGCGACCCTATCACCCACCGCACATGGGCCGCTCTGCGCCTCGGCGTTTTATGTGCGCTTCC
ACTCTCCGCTTCACTGTTTCACCCACACGCCCCTCACCCCCCTCACCCCCCACACACACACACGCACCAC
GGCAACGTCCTACACCACGCGCGACTCCACAGTAAACCAGGTTCTTCAAG
>lmaj_36_8_up with W88256 483140, 483541

TTTGGCTTGCGTGCGCTTGCATGTCGTCCGAGGCGTGGAGTGCGGTGGGATGCCACACTGCTGCTGGCAA
CTGGGGCGCCTGGGCATACTCACACTCATGCACTCGCGCACCCAGTTGCAAACTAGTTTGTGATTCGATT
TTCCTTTTCTACAGGTGTACCGGTGCGTAGATGTCAATGTGGTTTTGCTTGAGAAACATGTACACACGCC
CTCGCAAAGGGGCAGCGCTTGACTTCAATGTAACGAGTGAGGTCCCGCATACTTGCGTAAGTTGCTGTGT
CCGTGTGCCCGTCGTCGATTTGTCGTTCACTGGTTTCTGACCAGAAGCCGCGTATTCGCGCGCTTGGGTG
ATGCTGGTGCATCCTCCACTCCCCCCTCCTCCTCCCCCTCTGCATGAAAG
>lmaj_36_9_up with W88279 1582721, 1583121
CCCTACAGGCAGTGTTTGGCTTGTTTTGTGAGGCGCTTTCGACTCTTTTGTAGTGTTCCTCCTTAAAGCA
GATGCCGCTTCATCACCATCTTAAGGCCTCGTGCTTCGCAGCTATCGTTTCAGTTCAAGATCCAAGACAA
AAGAACACAAGAAGATCGGATTCCGCAGATGTCGACTGGATTTACCGCCGTACAAGAGAGCGCAACCAGG
ACCTGCCAGACGCTGGAGATGCAGACAAACGGTGCACAGTTTTTTCCGTTTTTTTTTCCCTTCGTGACCT
GAGGCAGGTAGGCGGGGAGCTCACCTTCCACTTCTCGCCCTTCTCTTCATCCTCTTTCTCTTCGATGTTT
GCGTGGGTGCGCACATTCTGCCTGCATTCTCACTGAGCCACCTTACGCAG

Coding data

As a negative control, we utilized 198 coding regions that had been experimentally verified. These sequences were retrieved from GenBank and are reported below. For each gene, the coordinates for the protein-coding region are listed as well as the GenBank annotation of function.

>AF009148 1, 921 DNA polymerase alpha
CGATACCAGGAGCTGAACATCTCGTCGTGGTCCACCATTGGCCGCCTGGACCTGAAACGACTTCCGCGCT
TCCAGGGAACCGCCACTAACGTGAATCAGGAAAAAGAGACGTGCATCGGGCGCCTTGTTGTGGACTCCTA
CTCACTCTCGCGCGAGCACTACAAGACAGTGAATTACAGGCTGCTGTCGCTTGCAGACCAGATGCAGCTG
CAGGGGATTACAAAGGGCAGCAACAACTTCGAGCCTGGCACCTCGGTGCTCACACCTGCGATGCTGTCGG
CATCGCGCGACATCTACGATGTGCTGCTGCAGGTGTGCAACTGCGCTGTGCTGTCCACAGCGGTCGTCTC
ACATCTCGATGTGATTCGACTGACCAAGCGCCTCACCACGATTGCAGGAAACCTGTGGAGCCGTACTCTC
TTCGGTGCCCGCTCCGAGCGCATCGAGTATCTCCTGCTGCACACGTTCCACGACCTCAAGTTCATTACCC
CTGATCGCTATGTGCAGGACTTCAAGCGGGGCCACGACGACGAGGAGGAGGATGGCAAACGGAAGGCCAA
GTACCAAGGCGGCATGGTGCTCGACCCCAAGTGCGGCCTTTACTCGGATTACATTCTCCTCCTCGACTTC
AACTCCCTGTACCCGTCGCTGATTCAGGAGTTCAACATTTGCTTCACCACCGTCGATCGTGAAAATCGGA
GCGAGATCGATGTGCCACCACCGGAGAACCTCATCTGTGCCTCGTGCGCCGCAGCAGGCCTCTCCGCTCC
GTGCTTGCACAAGTGTGTGCTGCCGAAGGTGATCAAGAGTCTCGTAGACAGCCGTCGCGAGGTGAAGCGG
TTGATGAAGACAGAGAAGGACGCGAACAACCTGGCGCTGCTGGAGATTCGCCAGAAGGCGCTGAAGCTGA
CGGCAAACAGC
>AF009163_0 325, 7236 RPOIILS
ATGTCGGGTGGTGCGGCTCTCCCGCCTTCGCAGATGCCGCTGCAGAAGGTGCATGAAGTTCAGTTTGAGG
TGTTCAAGGAGGCGCAGATCAAGGCCTATGCCAAGTGCATCATTGAGCATGCCAAGTCCTATGCGAGCAC
GGCCAGCCGGTGCGGGGCGGCATCAACGACCTGCGCATGGGCACGACAGACTTCGAGTATAGCATGCGAG
ACCTGCGGGCTGAGGCACCCGGAATGCCCCGGCCACTTTGGCTACGTGGAGCTGGCCGAGCCGATCTTCA
ACATCAACGTGTTTGACGTCGTGCTGATCGCGCTTAAGTGTGTCTGCAAGTACTGCGGCGCGCTGCTGAT
GGACACAAACGACCCGTCAGAGATGAAGAAGATCGCGCATCTGCAGGGCCTCAACCGCCTGCGCATGGTC
GCCAAGCTCTGCGGCAGCGTGTGCAAGCGGAGCAAAGACATCCAGGGCTGTGAGGGCNAAGGGCCGCCAG
CCGCGCATCGGCCCTTCGTTGGCATCTACCCAGGTCTGCAGATCAAGGTTACTCAGGAGGAGCAGGATTA
CGTGTGGCACGCCGAGAACGCACGGCAGGTGCTGGACCGCGTCTCGGACAGCGATGCCCTCATCATGGGC
TTCGACCGGCGCTTCTGCCACCCACGCGACCTTGATCTCACGGTGCTGCCGATCCCCCCGCCGCAAGTCC
GGAGCTCCATATACCTGGGCGGTCTAATGCAAGTCGACGACCTCACCCACCAAATCATGTCCATTGTGAA
GCGCAACATTCAACTACGCAAGGACAAGGAGTCCGGCGTCAAAGCCGCCGTGGACCGCTCGCGCGCACTT
CTGCAGGAGCACGTTGCCACTTTCTTCAACAATGCCTCCACCTACTACAAGCCCGCCAAGGTCGGCGACA
CCAAGAAGCTTAAGTCACTGACGGAGCGGCTGAAGGGGAAGTACGGCCGGCTGCGCGGCAACTTGATGGG
CAAGCGCGTAGACTTTTCGGCCCGCACGGTCATCACCGGCGACCCAAACATCGACGTCGATGAAGTCGGC
GTACCGTTCTCCGTTGCCATGACGCTGACCTTTCCGGAGCGGGTGAACGTGATTAACAAGAAGCGTCTGA
CGGAGTTTGTGCAGCGCACCACGTACCCTTCGGCCAACTACATCATCCGGCCGAACGGCAACGTAACGAA
ACTGGCCCTTGTGAAGGATCGCAGCGCCGTCCACCTCGACGTTGGCGACGTGGTCGAGCGCCACGTCATC
GACGGCGACGTTGTGCTCTTCAACAGGCAGCCGACGCTGCACCGCATGAGTATGATGGGGCACCGCGTCC
GCGTGCTCAACTACAACACATTTCGCCTGAACCTGTCCTGCACGACGCCGTACAACGCCGATTTCGACGG
CGATGAGATGAACCTGCATGTTCCGCAGTCGCTGCTCACTAAGGCGGAGCTGATTGAGATGATGATGGTA
CCCAAGAACTTCGTGTCGCCGAACAAGTCGGCGCCCTGCATGGGCATCGTGCAGGACAGCGTGCTCGGCA
GTTACAAGCTGACAGAGAAGGACACCTTCCTGGATAAGTACTTTATTGCAGAGCGTCCCCTTTGGCTGGA
TCTGTGGCAGCTGCCGATCCCAACCATTCTGAAGCCGCGTCCACTCTGGACCGGAAAGCAGATCTTCTCG
CTTATCCTGCCGGAGGTCAACCACCCCGCCTCCCCGCACGACAAGCCGCCCTTTCCGCACAACGACAGTA
AGATCATGATCCAGCGTGGCCAGCTGCTGGGGGGCGCCATCACAAAGGGCGTCGTCGGCGCCGCCCCGGG
GTCGCTGATCCACGTCATATTCAACGAGCGCGGCTCCGATGAGGTGGCCAAGTTCATCAACGGCGTGCAG
CGGATCACAACGTACTTCAACTACTGCTTTGCCTTCTCGGTCGGCGTGCAGGACACCGTGGCCGATGCGA
CGACGCTCAAGGAGATGAACAACGTGCTGCACAAGACGCGCCAATCCGTCGAGAAGATCGGCGCCGCTGC

GAACAACGGCAAGCTGACGCGCAAGGCTGGCATGTCGCTGCTGCAGTCCTTCGAGGCAGATGTGAACAGT
GCCCTGAACAAGTGCCGTGAGGAGGCGGCGAAGAAGGCGCTGAGTAACGTCCGCCGCACAAACAGCTTCA
AGGTCATGATCGAGGCCGGAAGCAAGGGCAGTGACCTGAATATTTGCCAGATCGCCGTGTTCGTCGGGCA
GCAAAACGTCGCCGGCAGCCGCATTCCCTTCGGCTTCCGTCGCCGCACGCTGCCGCACTTCATGTTGGAC
GACTACGGTGAGACGTCTCGTGGCATGGCGACGCGCGGGTACGTGGAGGGGCTGCAGCCGCACGAGTTTT
ATTTCCACACCATGGCAGGGCGCGAAGGGCTCATCGACACAGCCGTCAAGACGTCCGACACCGGATACCT
GCAGCGCAAGCTCGTGAAGGCGCTGGAGGATGTGCACGCCTCCTACGACGGCACGGTGCGGAACGCGAAC
CAGGAGCTCATCCAGCTGGCGTACGGCGAAGACGGTCTGGACGGTGCTCGTATTGAGGGCAACCAGGCCT
TCCCGATCCCGCACATGACGAACAGTGAGATGGCTGACAAGTACCGCTACGAGTACAACGACGAAGGCAG
CTTCTCGGAGAACATGGGCGGCCATTACATGGATCCGTTTGTGCGCGACTCGCTGCTGCGCGACCCGCAG
AGCGTGTTGAAGCTGCAGGAGGAGTTTGAGCAGCTGATGAAGGATCGCGCGATGTCGCGGCTCGTGATCG
ACATGGAGGACAAGAACAAGCTCAAGATGAACCTGCCCGTGAACGTCGCGCGGCTCATCCAGAACGCGCG
CACGACGATGGGCAAGCGCAGCCAGGTGTCCAATCTGAACCCGATCACTGTGATCAACCGCGTGCGGGAG
CTGCAGGAGGACCTCGTGCAGCTGTTCCCGTCCTACCACAAGGACTACAACGGCCGCTTCGTTAATGTGC
TGAGCCAGCAGCGCGTGGAGCGGGCCCTGACGCTCTTTGGCATCCACTTGCGCCAGATCCTCGGCTCGAA
GCGGGTGCTGAAGGAGTACAAGCTGAACGACAAGGCGTTCGAGTACCTGCTGAAGGAGATCCGCACCAAG
TACCAGCAGTCCCTCATCACCCCTGGTGAGATCATCGGCGCCATTGCCGCGCAGTCGTGCGGTGAGCCCG
CCACCCAGATGACCCTGAATACCTTCCACAACGCCGGCATTTCGTCCAAGAACGTGACCCTCGGCGTGCC
GCGGCTGTTGGAGCTGCTGAATGTGAGCAAGAACCAGCGCAACGCCAGCGTGGCCGTCTGCCTGATCCGC
GAGTACCAGAAGCGCAACAAGGCGCAGGAGGCGCAGCAGTTTATCGAGTACTGCACGCTGGCGAATATAA
CGACAACGGTGCAGATCATATACGACCCCGACCCGCGCAACACCGTCGTGGCCGAGGACGAGGAGATGAT
CCGGTGGGAGCAGGCGGTGATGAATGCAGAGGATGAGGAGCCAGATGCGGAGCAGCCGCCGTCACCTTTC
ATTGCACGCCTCATCCTCGACAACGACCTCTTCAACGACAAGCGACTTAACATGAAAGACGTGAAAAGTG
CGATCCGGCAGGTGGACGACACTTACATGGTGCAGGCAAACATGGAGAACGACGGCCAGCGCATTGTGCG
GCTGCGTCCGCGCAAGTGCACCGGGGCGGACTCGNTGCCGGCGCTCACGAAGGCGGTCGCGCAGCTGCTG
CAGAGCGTGCACCTACGCGGTATCCCTGGCATCAAGAAGACGCTGCTAAAGGAGGGCAACACGTTCCGCG
TCGACCCCGAGGCGGGCGGCATCAAGAACGAATCGAGCTGGATGGTGGACACAGAGGGCACGGCACTGCA
GCGCATCTTCGTCGGCGTCGTGAACAGCGAGGGCAAGAACATCATCGACTTCTCCAAGACCAGTAGCAAC
AAGATCCCTGAAGTGGTGACGGTGCTAGGCATCGAGGCAGCGCGGCGCAAGATGCTCTCCGAGCTGCGCG
AGGCTTACCTCGCCTACGGGCTAAACATCAACTACCGCCACTACACCATCCTCGTCGACACCATGTGCCA
GCGCGGTTACCTGATGGCCGTCAGCCGCACCGGCATCAACCGCAGCGAGACATCCGGGCCGTTGATGCGG
TGTTCCTTCGAGGAAACGGTGAAGGTGCTCATGACAGCCGCCGCGTTCGGCGAGAAGGATCCGGTGCGTG
GGGTGTCTGCGAGCCTCGTGCTCGGAAATCAGGCCCGCATCGGCACCGGCCTGTTTGACCTTCTGCTGGA
CATGAGCAAGCTGCAGCACGTGGTCCCGCTCGATAAGGCTACGGAGGCACGAACCTCAAACGTGTACCAC
ACCGACGCATCCGTCGCACCGGGCTCGTCGACGCTGCAGGGGTCGCACGGCGAGCTGCCGCCGTCGACTG
TGCACGAGAACAGCTCTCTTGCCGCCGGCGCCTCCTCCGTGTACCCGCGACATGAGAGAGCTGGCATGTA
CGGCGGTATCCCGATCGAGGCAAGCGAGGTGCAGTTCAGCAGCGCTCTGCCGACGGTGATGAGAACGGCC
TTGGCTTGCCTCGAACACGACCGAGTACCACAGCAGCCACCACCTCTNTGGTGCTTCCACGTACTTGGNG
TCGTCGGCGCTGCCGAGCGCGAGTCGCTTGATACCGAGCTGTCCTCCTACCACCTACAGTCAGTCGCCCC
TTCTACAGCGTACGGTTACGCACCCATGGCGGCCTCTGGAATGCCTATGCCCGGTGCTACTCAGCTATCC
GTCGGTGAGGGCGGCAGCCTGCCATACCCGTACGAGGCGTCGAACGGCGAGCGCTTCGGCTCTCTCGGCG
GCGCGGCCAGCTCGCGGGCGAGTGCGCCCTACGACCCCACCCAGCAGCCGTCTCAGGAATTCTCGCAGAC
CGAGGAGCAGGAGGAGCCGTGA
>AF009163_1 6364, 7236 CLT
ATGTTGGCCGCCATCGTTGGCGTGCTGGTTCTCTGCGTGTACGTTGTCCAGGCGGAGATCTTCTTCCACG

AAGAGTTCAACACCATGGATGGGTGGGTGCAGTCAGAACACACGAGCGACTACGGTAAGGTCGCGCTCTC
GGTGGGTGCGATTCACGTGGATGCTGAGAAGGAGCAGGGCCTGAAGCTCATGGAGGACGCCAAGTTCTAC
GCCGTCTCGAAGAAGCTGCCGAAAGCAGTTTCGAACGACGGCAAGCCGATCGTTGTCTCCTTCTCCGTCA
AGAACGAGCAGAAGCTTACGTGCGGCGGCACCTACCTCAAGTTCTTCTCCGAGCTGGACCAGAAGGACTT
ACACGGCGAGTCTGCGTACTGGCTGATGTTCGGGCCCGACATCTGTGGCTCCAACAAGCGTCTGCAGTTC
ATCTTGAGCTACAACGGCACGAACCATCTCTGGAAGAAGCTGTCGACGCCGAAGACCGACACAGCCACTC
ACGTCTACACGGTCGAGATCGCGCCAAACAACACGTACCAGCTGTACGTCGATGGCATGCACATCCAGGA
AGGGTCGTTGGAGGAGGAGTGGGACATGCTGCCGCCAAAGACCATCTCTGACCCGACGGATAAGAAGCCG
GCGGACTGGGTGGATGACATGATGATGGATGATCCGTCTGACACGAAGCCGGAGGACTGGGAGGACGAGC
CGGCCACCATCACTGACTCCGAGGCTGTCAAGCCGGTGGACTGGGACGATTCGGAAGACGGTGTGTGGGA
GGCCCCGAAGGTTCCGAACCCGAACTACCCGGGTGCGTGGAAGCCGCGCCGCATCCATAACCCCGACTAC
AAGGGTGAGTGGGCAGCGAGGCAGATCGAGCTC
>AF031902_0 5881, 13997 LORIEN
ATGCATGCTCTTTCGGAGGCGCTGCCCGCTGCACGTAGTGCTGCGAAGCGCACTTCGGGCAGGTCTTCCT
CGCCTCTGTCTGCCGGCGACGGCGAGGGGGGTGATGCGAGCGAGGAGGACGAGGACGCCTTACCATTTGT
GCGGGCTGCAAGGCGCCACGTGCTTGAGGTGCTCCGCCACGAAGTCTTCGAGAACCCGTCGAGGTTTGTC
TTGTCGCCCGTGTACCACTTCGTGGACATGCTCGGGGCGGTGGTGGTTGAAAACAGCAATCTGACCACGA
CGCTGCCTGCACCTCACACGCTGCACGAGAGACCGGCGGAGGGCTTCTCGCCGCAGCGGCGCGTGCTGCT
CGTGCCTTTGACAGAGGCCTACGAGCACACCACGGGGTGGATGGTGCCAACGGAGATGGAAGGCGCGCAG
CTTGCCATGTCGCTGTACGTCAACGACACACCCGTCTCTCTACCGCGAAACTGGCAGCTTTCCCCCTCCA
AGGAGGCGGCCGCAGTTAAAACCGCAATGACGGTGGATATCACGCCCCTTGTGCTCCCGATTGAACGGGA
TCTCTTCTCCTTACAGGTGATCTTCTCTGGCGATGTGGCCGAGATGGAGATGTGGCGCGGCGTCATCGCT
TGTGTGCTTGTGGAGGAAGTAGAGTTGGCCCACCTCGGTGAGCGCATCGTGTCCACGTATCACAAGCGTA
GAGCGCCCGTCATCCGGCGATCGCCGTTGCCATCCTGCGGCGAGCGCGGCGTCGTTGCAATCACGGAGGC
GTCGGTGAAGATTCAGTGCCCCATCACCACGCTCACGATGGAGATCCCTGTGCGGGGCATGTACTGCGAG
CATCTGCAGTGCATGGAGTTGGCGGCGGTGCTCATTCAATGTGCTCGGCAAAACGTGTGGAACTGTCCAC
TATGCTCGGCTGCCATGAAGCCGGAAGACATCATCGTCAACTACCGCCTCAAGGACTGGATCGCCTCGCA
TTCGCAGGAGGTAGTGGCGCAGGTCGAATATGTGGTGGAGACGGAGCTTGGCCGACCGCTGAAGATTGTG
TACAAGGCCGCAAGACCAGAGCAGCACTCCACAATCGAGGTGGTGGATGACGAGGAGGAATAA
>AF031902_1 7687, 13997 metK
ATGTCTGTCCACAGCATTCTCTTCTCCTCCGAGCACGTGACGGAGGGCCATCCAGACAAGCTGTGCGATC
AGGTATCCGACGCTGTGCTTGACGCGTGCCTCGCCGGCGACCCGTTCTCGAAGGTTGCGTGCGAGTCGTG
CGCGAAGACGGGCATGGTGATGGTGTTCGGCGAGATCACGACAAAGGCAGTGCTAGACTACCAGAAGATC
GTCCGCAACACGATCAAGGACATTGGCTTCGATTCCGCGGACAAGGGTCTGGACTACGAGTCGTGCAATG
TGCTGGTTGCGATTGAGCAGCAGTCGCCGGACATCTGCCAGGGTCTGGGCAACTTCGATAGCGAGGATCT
CGGCGCTGGCGACCAGGGCATGATGTTCGGCTACGCGACGGACGAGACGGAGACGCTGATGCCGCTGACG
TACGAGCTGGCCCGCGGCCTTGCGAAGAAGTACAGCGAGCTTCGCCGCTCCGGCAGCCTGGAGTGGGCTC
GCCCGGACGCGAAGACGCAGGTGACGGTGGAGTACGACTACGACACGCGCGAGGGCAAGCAGGTGCTGAC
GCCGAAGCGCGTGGCGGTGGTGCTGATCTCTGCGCAGCACGACGAGCACGTGACCAACGATAAGATCAGC
GTGGATCTGATGGAGAAGGTGATCAAGGCTGTGATCCCTGCGAACATGCTGGACGCGGAGACGAAGTACT
GGCTGAACCCATCTGGCCGCTTCGTGCGCGGTGGGCCGCATGGCGACGCCGGTCTGACTGGCCGAAAGAT
CATCGTAGACACGTACGGCGGCTGGGGCGCGCACGGCGGTGGCGCCTTCTCCGGTAAGGACCCTTCGAAG
GTGGACCGCTCCGCCGCGTACGCCGCGCGATGGATCGCGAAGTCGATCGTTGCAGGTGGCCTGGCGCGCC
GCTGCCTTGTGCAGCTCGCGTACGCCATCGGTGTAGCGGAGCCGCTGAGCATGCACGTGGAGACGTACGG
CACCGGCAAGTACGATGACGCGAAGCTGCTGGAGATCGTGAAGCAGAACTTCAAGCTGCGCCCGTACGAC

ATCATCCAGGAGCTGAACCTGCGCCGCCCCATCTACTACGATACGTCGCGCTTCGGCCACTTCGGCCGTA
AGGACGAGTTGGGCACGGGCGGGTTCACTTGGGAGGTGCCAAAGAAGATGGTCGAGTAA
>AF034357 6, 557 Leishmania major Abdou surface protein
amastin gene, complete cds
ATGCTGTGCTCGTGCATCGTGTTCATGTTCCTCGTGACGTCTGCGCCGATCTCGCAGTTCCGCGGCAGGG
GCATCAACGCTAGCGCCACTGGCGGCGCGAGCAAGTTGTCGTGCGTGACTGCGTGGGGGCTGAAGAACGA
CTGCAACTCCAACAACTACGACTACCGCCCGACGAGCATAGGGTGCGCACGCGCGAAGCAGCTGTTCCAG
GTGTCGGAGGCGTTCTACATTGTCGCCGTTATTGTGTCGTTCCTGTCGTGCCTGATGAGCGGCCTGTACT
TCATCGGCATGAAGGCAAAGGTGCTGCTAGTCCTGCTTGCCGTCCTTGAGGTTGTGGTCGCGCTGATCCC
ATGGGTGTGCATGACGGCGGTGTGGCACGGCAACTACTGCGGCGGGTCGACCGTGAAGATCAACACGTCG
AACGGCAAGGCGGACGGCGTGCCGTACGGCTCTGTGCTGCGTGAGTCGTTCAAGGCCAGCGCTGGCTACG
GTCTGACGGTTGCGGCGTGGTGCACCCAGGTGATTGGCCTGGTGCTGCTGATTATAATGTAG
>AF034804 22, 960 LACK
ATGAACTACGAGGGTCACCTGAAGGGCCACCGCGGATGGGTCACCTCCCTGGCCTGCCCGCAGCAGGCGG
GGTCGTACATCAAGGTGGTGTTGACGTCGCGCGACGGCACAGCCATCTCGTGGAAGGCCAACCCCGACCG
CCACAGCGTGGACAGCGACTACGGTCTGCCGAGCCACCGCCTGGAGGGCCACACCGGCTTCGTGTCGTGC
GTGTCGCTGGCCCACGCCACCGACTACGCGCTGACCGCGTCCTGGGACCGCTCCATCCGCATGTGGGACC
TGCGCAATGGTCAGTGCCAGCGCAAGTTCCTGAAGCACACCAAGGACGTGCTCGCCGTCGCCTTCTCGCC
GGACGACCGCCTGATCGTGTCCGCGGGCCGCGACAACGTGATCCGCGTGTGGAACGTGGCCGGCGAGTGC
ATGCACGAGTTCCTGCGCGACAGCCACGAGGACTGGGTGAGCAGCATCTGCTTCTCGCCGTCGCTGGAGC
ATCCGATCGTGGTGTCCGGCAGCTGGGACAACACCATCAAGGTATGGAATGTGAACGGGGGCAAGTGTGA
GCGCACGCTCAAGGGCCACAGCAACTACGTGTCCACGGTGACCGTGTCGCCAGACGGGTCGCTGTGCGCG
TCCGGCGGCAAGGACGGCGCGGCGCTGCTGTGGGACCTGAGCACCGGCGAGCAGCTGTTCAAGATCAACG
TGGAGTCGCCCATCAACCAGATCGCCTTCTCGCCCAACCGCTTCTGGATGTGCGTCGCGACGGAGAGGTC
TCTGTCCGTGTACGACCTCGAGAGCAAGGCCGTGATTGCGGAGCTGACGCCGGACGGCGCGAAGCCGTCC
GAGTGCATCTCCATCGCCTGGTCCGCCGACGGCAACACCCTGTACTCCGGCCACAAGGACAACCTGATCC
GCGTGTGGTCCATCTCCGACGCCGAGTAA
>AF034925 196, 3564 MRK1
ATGCGCAGGGAAGGTGGGGCATTTTCGCGGACATCGTCACCGCGGTCGACCACCTCGCCCATCAGCCCTG
CCGACGAGGAGGATGAGTACATGGGAAGTAGCACGTCCACCGCTGGGCCGCCACCCGAGTCCAACACCAT
GACCGGTCGCTTCACTCGACCGCAGTGGTCGCTGGCAACAGAGCGACACTATGCAAGCAGTGGGCACTCA
GCCGCCAGCTCGTGCAGCACTCACACGCTGAAGGTGAACCAAACGGAGAGCTACCGGCCGGGTCCCGATG
ACAGCGGCACATACAGCGCGAACCTGCGTGACTCGCCCAGCCGCACCATGAGCAACACCAGCGTTTCGGT
CTCCCCCATGGATAGCAGCAGCCCGCAGGAATGGGGCCGTTCCTTCCGCCTTTTCAACTCCTGTCGCTTC
CTCACCAGCGCCTCAGGCTCACGGCAGCGTGACGGCAGCATTGGGCAGCTGTATCGCAGCACTTTCCTGC
ATGGTCGGCAGGGCCGCGGCAGCAGCATTGGCCCGGAGGCCGAAAGGCCACTCCTCGGCAGCTCCATGGT
CCATCAACCACAGAACAGCTTCTTTGGAACCTTTAGCGGGCAAGGTGGACGCTATTCCTTCTGGCCCCCT
GATGTGAACGCCAGCGGAGTACTGACGGCCGCCTCGCCGTACGTCGCATCCAATCCGTCCGCGCCTGTGT
CGACAGCGAGTCCCCTATTAAGCTCTTCTCGCTTCCTCGGGACGGGGATCTCGCCTCGCGGGCTGGCGGA
CGGGCACGCTGTGCATCGTGTTGTCGCGTCGTGCGCAATGGGCTCGGTGGTGTGTACTGTCTCACCAGGT
CAGAACGAACTGCCTTTGGCGATGAATGCCTTACGCACGCACATTGCGCTGCGCGGCAAGACGATCATTC
AAGGCGATGCCGCGCACCCGCTGGACATTCGCAAGGGTCCCCTTATCGGCGCCGGCGGCTTCGCGAAGGT
CTTCGCGGGTGTGGACACGATACGCGGAGAGCTTGTCGCCATCAAAGAGATCGACATCTCCCGCGTGGAC
GACGTGAAGGCGCTCAACGCGATCGAGGCGGAATTCGCCCTCCTCAAGTCGCTTCATCACCCCAACATTG
TCAGCTACTCCCTCTTCGAGCACAGCAAGTCGCAGAAGGTCTGCCGCATTGCGATGGAGCTGCTTGCTGG

TGACTCCACTCTCCACTTGCTGCAGAAGTTTGGACCACTGACGGAAGCGGTGCTGCGCATCGTGGCGCGG
AGCGTCCTGCGCGCCATTCGCTTTATCCATAAGGAGGGCATTTTTCACCGAGACATCAAACCCGCGAATA
TCCTCGTCAGCCACCGCGGCGAGGTGAAGCTGTGCGACTTTGGCTGCAGCAAACGCGTGTCGGAGCTGAA
CAAAGCTGCCAGCTGCATCATTGGAACACCAGTGTACATGGCCCCGGAATTCATCAAGGGTGAGGCCAAT
CACAAGGCGGATATATGGTCGATGGCATGCGCGCTTTTCGAGCTGAGCACCGGGCTGCTGCCTTGGTACC
ACTCCGGCGCCAAGGACAATCTCCCTCTCATGTTCTACCTCACGACAACGTCGGAGTCTCCCATGGTGCT
GCCCTCGCCAGAAGCCAAGAGCGAGTTCTCCGCGGAGTTCTTCAGCTTCATGGAACTGTGCTTCACACGC
AACGTGGCTAATCGACCAGAAGCTGACGATTTGCTGAAGCATCCGTGGATCACGGGGTCGCGGCTCGCGC
CGGTACCAAACCTGCCCAGCGCTGTCTTCTCTCTCCACCAGGAAAGTTCCGCCGTGCTTCGGGCCTTCGG
CTCACCCGGGAAGTCTTCCACATTCTCCAGCCCGCGAGACTCGGCGGCGGACCGCGACGACATGAGCTTC
AACGGCAGCGACGGCTTGCAGAAGTCCGTCTCCACCACCCCGACTCTCAACCCCGCGGAGGAGGAGATGG
CGTGCCAGCAGGAGTTGGAAACCGTGGCGGCGGCGACGGCGCTGGAACTCTGCTCCCTGCTCGTGTGCTC
ACTGGAGATCCCCCCTCGTACCGCGCAGGAGACTGTGACAGGGGAAGTGGGCTCGGGGACCGGCGACACG
AGTCCCTCGCTCACGCGCACCTTCTCGATCCACTCACCCGCCCTCAGTCATCGTGGCGGCGGTGTCAGCG
CGAGCGTGGCCGAATTTCTATCCCCTGTGTATACCCCAGAGCATTCCATGGTGCACGAAAACTTTTACTA
CAGCCAAGTGCCACTTGGGGCGTCTGCAGGCAACTTTGTCTTGCCGCCCGGACTGGACTTGGATGGGGCA
CCGCCGCAGCAGCAGTACTTGCGCATCAACGAGGAAGGCAACCTTGATATGGTTCACCTCCCCGGTGACG
AGATGGAGGACTCGGTGCACGGCAGGTCCGCCTTCGGCGACAGCGCCCACGGCTCCTTCCACGCTGGGCG
TATTGTGTCCCCAGCGCGTAACAGCTCGTTCAGCAGAGGTGCCCTCAGCCCGCGCGTCGCGACGGGGCCG
AACGCCATGGGCGTAGCCGACGGCCCTTTCTCCCCGCTTCTCGGCATCAGTGGCGTCTTCTCTCGCAGGG
AGTCGCCGTCGCGCGCGGGCCCGTCAGCTCCGTCGTCCCCGCAGGGGATTCCCGCGCCGCCGCTGCTGTG
CCGGCCCGGCCCGCCGACGACATTTGTAAGTCAGCCATGCAGCCACACCGGCTCTGCGCATACGTCGCAC
GTCTCTCCCACGTCCGCATCCAAGACCGGCAGCAACCTCGACGCCGTGACGCCTCGCACCCCGCGGTCTA
ACTCGCCCACTTCCACGCAGCGCAGTGAGAGCTTCCGCGGGTTGCCGGAAAAACTGAAGGCGCACGCTGA
TGGAAAACTGCACATGTCTTTCTCTGTCAACACCGCCCCAGGATGCGCCGTGAACGTGGAGCTGAACGTC
GATGTGGCTGATGTGCAGTGCAAAGTGGTGGATAACCAGCCGAACTTTGTGGTGGCCTTCACGGATGACG
TGCGAAGCCAAATCGCAAACAAGATTAAGGAGGTGGCTGAACACTCTTCGTCGGGCATGCCTGCCCAGAC
TGCAGGAGCTCATGCCTCGTCCCTGAGCCCGTCGTGCGGGATGGAAGCGCACCGGTCGGTCTCTGCAGGT
GGCGGCCACTTTTACAACGCACCGTTATTCGCGTCCGCCTCGAAGGCCACGCCGCGTCCGCTTTCACACG
TCTCCACCGGCAGCGCGCAGCGACTGGTGGCCGTCCCGCGCGAGTCGCTTGCCGGCTATTCGCCCTACTC
TGGGGAGGAGGGCTCCGACATGGACTCCCACACGTCGTCGCCAATGGCAGCGGGCTGGAATGAACGCATG
GACAAGTAG
>AF038409 912, 1901 arginase
ATGGAGCACGTGCAGCAGTACAAGTTCTACAAGGAGAAGAAGATGAGCATTGTGCTTGCCCCTTTCTCTG
GCGGCCAACCGCACAGTGGGGTAGAGCTGGGTCCTGACTACCTCCTCAAGCAGGGACTGCAGCAGGACAT
GGAGAAGCTTGGATGGGATACAAGGCTCGAGAGGGTGTTCGACGGCAAGGTTGTTGAGGCTCGCAAGGCG
AGCGATAATGGCGACAGGATCGGTCGTGTCAAGCGCCCGAGGCTGACGGCGGAGTGCACGGAGAAGATCT
ACAAGTGTGTGCGCAGGGTGGCCGAGCAGGGTCGCTTTCCTCTCACCATCGGTGGCGATCACTCCATCGC
CCTCGGCACGGTGGCCGGTGTGTTGTCCGTGCACCCGGATGCCGGGGTGATTTGGGTGGACGCCCACGCG
GACATCAACACTATGTCTGGCACGGTCTCCGGCAACTTGCACGGCTGCCCCTTATCGATCCTGTTGGGGC
TTGATCGAGAGAACATTCCTGAGTGCTTTTCGTGGGTACCGCAGGTGCTGAAGCCGAACAAGATTGCCTA
CATTGGTCTGCGTGCTGTGGACGACGAGGAGAAGAAGATCCTGCACGACCTGAACATCGCCGCCTTCAGC
ATGCATCACGTGGACCGCTACGGCATAGACAAAGTGGTGTCCATGGCGATCGAAGCCGTCTCGCCGAAGG
GTACGGAGCCGGTGATGGTGTCATACGACGTGGACACGATCGACCCCCTCTACGTCCCGGCGACGGGCAC
TCCCGTGCGTGGCGGCCTCTCTTTCCGCGAGGCGTTGTTCTTGTGCGAGCGTATCGCCGAGTGCGGTCGT

CTTGTCGCTCTGGACGTGGTGGAGTGCAACCCGCTCCTCGCCGCTACGGAATCGCACGTGAACGACACCA
TCTCCGACGGGCGCGCCATCGCGCGCTGCATGATGGGAGAGACACTTCTTTACACTCCGCATACGAGCTC
CAAGCTGTAG
>AF039721 301, 2235 gp63-6
ATGTCCGTGGACAGCAGCAGCACGCACCGGCGCCGCTGCGTCGCCGCGCGCCTGGTGCGCCTCGCGGCTG
CCGGCGCCGCAGTCACCGTTGCTGTCGGCACCGCGGCCGCGTGGGCACACGCCGGTGCGCTGCAGCACCG
CTGCGTCCACGACGCGATGCAGGCACGCGTGCGGCAGTCGGTGGCGGACCACCACAAGGCCCCCGGCGCG
GTGTCCGCGGTGGGTCTGCCGTACGTTACTCTCGACGCCGCGCACACCGCGGCCGCCGCCGATCCCAGGC
CGGGCAGCGCGCGCAGCGTCGTGCGCGACGTGAACTGGGGCGCGCTGCGCATCGCCGTCTCCACCGAGGA
CCTCACCGACCCCGCCTACCACTGCGCTCGCGTCGGGCAGCGTGTCAACAACCACGCCGGCGCCATCGTC
ACCTGCACCGCCGAGGACATCCTCACCAACGAGAAGCGCGACATCCTGGTCAAGCACCTCATCCCGCAGG
CGGTGCAGCTGCACAAGGAGCGGCTGAAGGTGCAGCAGGTGCAGGGCAAGTGGAAGGTGACGGACATGGT
CGGCGAGATCTGTGGCGACTTCAAGGTGCCGCAGGCGCACATCACCGAGGGCTTCAGCAACACCGACTTC
GTGATGTACGTCGCCTCCGTGCCGAGTGAGGAGGGTGTGCTGGCGTGGGCCACGACCTGCCAGACGTTCT
CTGACGGCCATCCAGCCGTGGGCGTCATCAACATCCCCGCGGCGAACATTGCGTCGCGGTACGACCAGCT
CGTCACGCGTGTCGTCACGCACGAGATGGCGCACGCGCTCGGCTTCAGCGGCCCATTCTTCGAGGACGCC
CGCATCGTGGCGAACGTTTCGAACGTTCGAGGCAAGAACTTCGATGTTCCCGTGATCAACAGCAGCACGG
CAGTGGCGAAGGCGCGCGAGCAGTACGGCTGCGACACCTTGGAGTATCTGGAGGTGGAGGACCAGGGCGG
TGCGGGCTCCGCCGGGTCGCACATCAAGATGCGCAACGCGCAGGACGAGCTCATGGCGCCTGCTGCAGCT
GCCGGGTACTACACCGCCCTGACCATGGCCATCCTCCAGGACCTCGGCTTCTACCAGGCGGACTTCAGCA
AGGCCGAGGTGATGCCGTGGGGCCAGAACGCCGGCTGCGCCTTCCTCACCAACAAGTGCATGGAGCAGAG
CGTCACGCAGTGGCCGGCGATGTTCTGCAATGAGAGCGAGGACGCCATCCGCTGCCCCACCAGTCGTCTC
CTCCTCGGAACGTGTGGTATAAGGGAATACGAACCGCCGTTGCCGCGATACTGGCAGTACTTCACCAACG
CGTCTCTTGGGGGCTACTCGCCATTCCTGGACTACTGCCCGTTTGTTATCGGCTACGCTGATGGCTCGTG
CAATCAGGACGCATCGTCGGCAGAGGAGTTCTTCACTGCATTCAACGTCTTCTCTGACGCGGCGCGCTGC
ATCGATGGCGCCTTCAGACCGAAGGCAACTAACGGCATAGTCAAGTCGTACGCCGGCCTGTGCGCCAACG
TGCAGTGTGACACGGCCACACGCACGTACAGCGTGCAGGTGCACGGCAGTAACGACTACACCAACTGCAC
GCCGGGCCTCAGAGTTGAGCTGAGCACCGTGAGCAAAACCTTCGAGGAAGGCGGCTACATCACGTGCCCG
CCGTACGTGGAGGTGTGCCAGGGCAACGTGCAGGCTGCCAAGGACTTTGATGGCGACTCCGACAGCTCCA
GCAGCTCCAGTGACGCTGCCGACAAGGCGGCGATTGAGCGGTGGAATGAGAGGATGGCCGGCCTGGCTAC
TGCGGCGACGGTGCTGCTAGGAGTAGTTCTCTCTCTCATGGCACTCGTCGTGGTGTGGCTACTGCTTGTC
AGCTGCCCCAGGTGGTGTTGCAAAGTTGGGGGGCTCCCGACGTGA
>AF044679 27, 626 TSA
ATGTCCTGCGGTAACGCCAAGATCAACTCTCCCGCGCCGTCCTTCGAGGAGGTGGCGCTCATGCCCAACG
GCAGCTTCAAGAAGATCAGCCTCTCCTCCTACAAGGGCAAGTGGGTCGTGCTCTTCTTCTACCCGCTCGA
CTTTAGCTTCGTGTGCCCGACAGAGGTCATCGCGTTCTCCGACAGCGTGAGTCGCTTCAACGAGCTCAAC
TGCGAGGTCCTCGCGTGCTCGATAGACAGCGAGTACGCGCACCTGCAGTGGACGCTGCAGGACCGCAAGA
AGGGCGGCCTCGGGACCATGGCGATCCCAATGCTAGCCGACAAGACCAAGAGCATCGCTCGTTCCTACGG
CGTGCTGGAGGAGAGCCAGGGCGTGGCCTACCGCGGTCTCTTCATCATCGACCCCCATGGCATGCTGCGT
CAGATCACCGTCAATGACATGCCGGTGGGCCGCAGCGTGGAGGAGGTTCTACGCCTGCTGGAGGCTTTTC
AGTTCGTGGAGAAGCACGGCGAGGTGTGCCCCGCGAACTGGAAGAAGGGCGCCCCCACGATGAAGCCGGA
ACCGAATGCGTCTGTCGAAGGATACTTCAGCAAGCAGTAA
>AF047497 1, 582 GAPDH
GACGTCGTCGCTGTCGTGGACATGAGCACGAATGCTGAATACTTCGCGTACCAGATGAAGTACGACACGG
TGCACGGTCGCCCGGAGTACACGGTGGAGGCTGTGAAGAGCACCCCGTCCGTGAAGACGCCAGATGTGCT

TGTGGTGAACGGCCACCGCATCAAGTGCGTGAAGGCGCAGCGCAACCCCGCGGATCTGCCGTGGGGCAAG
CTCGGCGTGGATTACGTGATCGAGTCTACTGGCCTGTTCACGGACAAGTTGCAGGCCGAGGGTCACATCA
AGGGTGGTGCGAAGAAGGTCGTGATCAGCGCCCCGGCGTCTGGCGGCGCCAAGACGATCGTGATGGGCGT
GAACCAGCACGAGTACTCGCCGGCGTCGCACCACGTGGTGTCGAATGCGTCGTGCACGACCAACTGCCTG
GCCCCCATTGTGCACGTGCTGACGAAGGAGAACTTCGGCATCGAGACCGGTCTGATGACCACCATTCACT
CCTACACGGCAACGCAGAAGACGGTGGACGGCGTGTCGCTCAAGGACTGGCGTGGTGGCCGCGCGGCGGC
CGTAAACATCATCCCGAGCACA
>AF058760 631, 1515 mag
ATGCGTGACGGAGTGCACCGCTTCGGCCCTCGCGCGCTCGCTCGCTGCGTTGATTGCGTTGAAGGGCGGG
GGGGGCGACAGTGCAGTGCGCAGACACGCACACAATCCAAGGCACGCTCTCATTCACGGCTCGAGCGCCG
TCCCAGCCGCCATCCCTCTGCCGTCGCTGTCCGCGTCGCTGTGGGTCCGCTTGCTCCCCGGTGCGCCGCT
CACGCCATCACGCCAGCCGCCGTTCAGACCCCGCTCAACCCCCCCACGGCCACGGCCACCACCACCACCC
CACACACGCACGGACACACACAGCGTCGTCCTCGGCGACGTCCCTTGTCGTGCCCCCTCTGCTGTGCAGC
ACCCCTGCGCGTCGTTGTGTGGCTCACAGCAGTGTCGGTGCGACGCCGCACTGGGCGGGCGAAACGACTC
GCACGGGGGAGGGCGGCCGCTGCTGCTGCTCCCCTGCGCCTTGCCTGTCGCATTGTTTGGGGTGGCCCCC
TCTCCACGGGGCCCTCGTGGGGACGCTGCGGCCTCTCGTGGAAGGACGGGCGTGCACGCGGGCTCCGATG
GGCCCCCTTGTCTCTGCGCCTGGCTTGCTGGCCTCGCTCAGTGTCCCGCGGCCAGCCGTGCTGCGTCAGC
GCACCCCTGCCCAGCTCATGCGAACTGCGCACCCGTCAAGAGGCGACGGTGGCGAAAGGCGCGCGTGTGG
CACGAGCACACACGCCGTCACTGCTGCCGTGGTGCCCACTCGCTTTCCTTGGTCCCTTGCATGCAGCGTG
CCTGCCTCGCTCTCAGCGAGGCGAAGTACTTTGCGAGGGGCGGACGGCAGGAGCGCCAACCAAAACTGCA
CAGAAGACGCGCTGCTGTGCGCGGTGGCGGTGCCGCTGGTGGTAG
>AF060886 385, 1092 aprt
ATGTCCCTCAAGGAAATCGGACCCAACTCTTTACTGCTCGAGGATTCGCACTCCTTGTCGCAACTGCTAA
AGAAGAACTACCGCTGGTACTCCCCAATCTTCTCCCCACGCAACGTCCCTCGCTTTGCCGATGTCAGCAG
CATCACCGAATCTCCAGAGACGTTGAAAGCTATTCGTGACTTCCTTGTCGAGCGGTACCGCACCATGTCG
CCAGCCCCAACGCATATCCTAGGCTTCGATGCTCGCGGCTTTCTCTTTGGCCCCATGATCGCCGTCGAGC
TCGGAATCCCGTTCGTTCTGATGCGCAAGGCGGACAAGAATGCTGGTCTCCTTATCCGAAGTGAACCGTA
CGAAAAGGAGTACAAGGAGGCCGCGCCGGAGGTCATGACGATCCGCCACGGCAGCATCGGCAAGAATTCG
CGTGTGGTGCTAATCGATGACGTTCTGGCAACGGGTGGAACGGCGCTGTCGGGGTTGCAGCTGGTGGAGG
CAAGCGGTGCAGAGGTTGTCGAGATGGTCTCGATCTTGACCATTCCTTTCCTGAAGGCGGCGGAGAGGAT
TCACTCTACCGCTGGCGGTCGATACAAGAACGTTAGGTTTATCGGTCTTCTCTCTGAAGACGTGCTGACA
GAGGCGAACTGTGGAGACTTGAACGACTACACTGGTCCCCGTGTGCTGAGCTGCAGCGATCTACTGGTGA
ACCAGTAG
>AF062379 1, 1134 RAD51
ATGCAGACCCGTTCCAAGGCCAAAGGTCGCCGTGGTCGTCCGTCGGCGCGGCCCTCTGAAGAGGTTGAGG
TTGTGGAGAGCCAGCCGCAGGAGGTCCTACAGAACGAGGAGCAGGAGCCTCAGCAGCAGCAGCAGCAGCA
GAGCACCGACATGGCTGAACCGAACGCAAGTGGCTTTCGCGTTATCCAGATCTTGGAGAACTACGGCGTG
GCCAGCTCAGATATCAAGAAGCTCATGGAGTGCGGCTTTTACACGGTGGAGTCGGCGGCCTACGCACCGA
AGAAGGCCATCCTGGCAGTGAAGGGGATCAGCGAGAACAAGGCCGAGAAAATTATGGCGGAGTGCGCCAA
GCTGGTGCCGATGGGGTTCACTTCCGCGGTTGCCTACCACGAGGCGCGCAAGGAGATCATTATGGTCACT
ACGGGCAGCCGCGAGGTGGACAAGCTACTCGGCGGCGGCATCGAAACTGGGAGCATCACGGAGCTCTTCG
GAGAGTTCCGTACGGGCAAGACACAGCTCTGCCATACGCTGTGCGTGACGTGCCAGCTGCCCATCTCACA
GGGTGGCGCGGAGGGCATGGCGCTCTATATCGACACCGAAGGCACCTTCCGCCCGGAGCGCCTCGTTGCC
GTTGCGGAGCGGTACAAGCTGGACCCGGAGGATGTGCTCGCTAACGTGGCGTGTGCGCGTGCCTTCAACA
CGGATCACCAGCAGCAGCTGCTGCTGCAGGCGTCTGCCATGATGGCCGAGAACCGCTTCGCGCTCATCAT

TGTAGACTCTGCAACCGCTCTCTACCGCACAGACTACAGCGGCCGCAACGAGCTCGCGGCGCGGCAGATG
CACCTCGGCAAATTCTTGCGCTCGCTGCACAACCTCGCCGAGGAGTACGGAGTGGCGGTGGTTGTGACAA
ACCAGGTAGTGGCCAACGTGGACGGCTCCGCGCAGATGTTCCAGGCGGACTCCAAGAAGCCGATTGGAGG
CCACATTATGGCCCACGCCTCGACGACGCGGCTTAGCCTGCGCAAGGGTCGTGGCGAGCAGCGCATCATA
AAGGTGTACGACTCTCCATGCCTGGCCGAGGCCGAGGCGATCTTTGGCATCTACGATGATGGCGTTGGTG
ATGCTCGGGATTGA
>AF062380 1, 1095 DMC1
ATGCAGCAGCAGCAGCAGCAGCAGCGGCAGCACAGTTCGCACTTCGCGGAGGAGCGCGTCGGTGACCGCG
GCGCCGCCTTCGCAGAGCCTCAGCCACTCCACAACTCGGTCACCGGCGAAGCGGCGGGACAATCTCTGCT
CGAGGTGGAGCGTCTTGCGGAGCACGGCATCGGGGCCGCAGACATTACAAAGCTGAAGCAGGCGGGGATC
TTCACGGTGCCTGGTGTGCAGATGCAGTGTAGGAAAGACCTCATTCAGATCAAGGGCCTCTCCGAGGCGA
AGGTCGACAAGATCATCGAAGCTGCGCGGCGCGTGAGCGAGGTGGGCTTCATCACCGGCTCCAGCTGCCT
GCAGCAGCGCAGCACTCTTCTGCGCATCTCCACCGGGAGTACGGCGCTCGATCAGCTGCTCGGTGGCGGC
GGTATCGAGAGCCGCTCCATCACGGAGGCGTTCGGGGAGTTCCGCACCGGCAAGACGCAGATCGGGCACA
CCTTGTGCGTGACCTGTCAGCTGCCGCTGGAGATGGGCGGCGGCAACGGAAAGGCGGTGTACGTGGACAC
CGAGGGAACGTTTCGCCCGGAGCGGATTCGGCCCATTGCAGAGCGCTTCGGGATGGACTCAAACTCCGTG
CTGGACAACATCCTTGTCGCCCGCGCCTACACGCATGAGCATCAAGCTCACCTGCTATCGATGGTGGCGG
CCAAGATGGCGGAGGATCAGTTTAGCCTGCTCGTCGTAGACAGCATCACGGCTCTCTTTCGCGTTGACTT
CTCCGGCCGCGGCGAGCTCGCCGAGCGGCAGCAGAAGCTGGCAAAGATGCTGAGCCAACTGATAAAAATC
GCGGAGGAGTTCAACATTGCCGTGTACATCACCAATCAAGTAGTCTCCGACCCCGGTGGCGCCTCCATGT
TTGTGGCGGACCCAAAGAAGCCCGTCGGCGGCCACATCCTCGCCCATGCGTCGACGACGCGCCTGTCGCT
GCGCAAGGGCCGCGGCGATCAGCGCGTGTGCAAAATATTTGATAGCCCATCGTTGCCGGAGCTCGAATGC
GTGTACAGCATCTCTGAGCAGGGGATCATTGACGCGGTCGAATGA
>AF067495 1303, 4446 CA1
ATGAACTTTGGAGACGAACAAGGCGACGAGCCAACTCCCGATGCGTACGACGCCAAAGACAAGTTCTGGG
CGTTGGCGCTGGAGAACGTCTTCAACCTCGTGCAGTTGGAGGACCCCCTCGCTGGTATCGATGCCGCCGA
CGCACCGCGCCATGCCAAGGAACTCGGCGACAACGTCATCCCAATCAAGGGCGGCCCGAGTTGGATTGTT
ATCTTGGCGAGCCAGTTCAAAAACGCCATCACGATTGTTCTGCTGATCGTTATTATCATCAGCGGTGTGT
TTGGGGACTGGGCTGAGTTCGGCGTCGTTCTCTTCATCCTCTTCTTTAACGCCTTGCTTGGCTTCTACCA
GGAGTACGGCGCCGAGAGGTCGCTGGCGAGTCTCAAGCAAATGACGGCAGGTGTGGCGAAGGTGATACGT
AATGGCATTCCCGAGATCATCTTCATTGACGAGGTCGTCGTCGGTGACGTCATCGTGCTCGAGCAGGGCG
CCTCCGTGCCGGCTGACTGTCGCATTTTCGAGTCGAACGGGCTAGAGGTGGATGAGGCTCTGCTGACGGG
CGAGGCGCTACCGGTGGTGAAGCACGCGAACGTCATTCGCGACCCGGAAAACCGCCTCGCTCTTGGCGAC
CGCAAGAACATGGTGTACCGCAACACGCAGGTGACGCAGGGCCGCGGCAAGGCGGTGGTCGTCGCCGGTG
GTCTGAACACCGAAATGGGCAAACTTGCAAAGCGGCTGAACGACAGCAAGGGCAGTGGCAAGACGGCGCT
GATGAAGAAGCTCGACTACATGATGTACTTCCTGTTCCTTTGCTGTGCCGTTGCCGCCGTGATCGTGTTC
GCGGCCAACAAGATGCGCTACACGCCGGCAACCCTCTCGTACGCCACGGCGGTGGCCATTGCCATTCTCC
CCGAGTCGCTTTGCGCCGTCATCACGGTTGCCATGACCTTCTCTGTGAAGCGCATGGCACAGCAGAAGTG
TATTGTGCGCAAGCTGCCGGTGCTGGAGGTGCTGGGCAACGTCACAGACATCTGCTCCGACAAGACCGGG
ACGCTGACGGAGAATAAGATGGTGGTCAAGAAGGCGGTGATTGGCGTTGACGACGTGTACAGCGTTGGCG
GCGCGCCGTACGACACTCACGGCGACTTCTTCCCCGCCATGCGAAACGGCCAGGAACAACAGCCCGTGAA
CATGGCCCATCAGCAGGAGGTGCGCTACATCTACCAGTTTCTCAAGTGTGCCGCCCTCTGCAGCACAACG
GTCCTGCATGTCTCTGAAGAGGACATGGACTCCCTCGCTGGCAACGGCAACCCGACCGAGATTGCCATCC
AGGTAATGACCTGGAAGGCCGGCCTAAACCGCGACAAGCTGGAGGAGGAGGGCTGGGAGTGCATCACGGA
GTACTCTTTCGACTCTAAGATCAAGCGTATGTCCACGGCATGGGAAAACAAGGCGAAGCGCGAGCTCTAC

CTCTGCACCAAGGGCGCCCCCGAGCGGGTCGTTGAGCTGTGCACGTACAAGATCAGCGAGGATGGCAAGC
TTGTCAGCCTGACGCAGCAGGACCGCGAGCAGGTCGACCAGCACATCCGTGACTTGGCCGCGCAGGGTCT
ACGCACCATCTGCTTTGCATACCGCGAGGACGCCACGAAGATCTTCCCGATCCCGGCTGAAGAGCCGTTC
ATCGACGCTTACGACCGCGACCAGGTCGAGCGCGACCTCGTCTTCCTGGGTATTGTCGGCATCTACGACC
CGCCCCGCCCCGAGTCTCGACCGTCCGTCGTCGCCTGCCAGCACGCCGGCATCTGCGTGCGCATGCTCAC
GGGTGACCACACGTCCACCGCCGGCAGCATTGCCTCGATGCTGAACATCATTCGCCGCCGCGACCTCGAC
GACCCGGTGAAGCTGCAGGCGGGGCCTGACTTCGACAAGGTCGACCCTGAGACGATCGACGGCTGGGCCG
ACCTCCCCGTCGTCGTTGGCCGCTGCTCTCCCGAGTCGAAGGTGAAAATGATCGAGTCGCTGCACCGCCG
CAAGAAGGTGGTGGCCATGACGGGTGACGGCTTCAACGACTCCCCCAGTATCAAGATTGCCGACATTGGC
TGCGCTATGGGCTCCGGCGTCGACGTGACGAAGGGCGTCGCAGATCTCGTCATCACTGATGACAACTTTG
CCACCATCGTGAAAGCTGTCGCCGAGGGACGCCGCATCTCGCAGTGTATCCGCAAGTTTGTTGTACATCT
GCTGTCGAGTAACGTGGCTGAGGTGATTGCGCTGATCTGCGGCCTGCCTATCAGCCACGGCGGCGAGTCG
GTGTTCATCCTATCCCCCATCGAGATTCTGTGGCTGAACATGTTCACCTCGGCCCCACCGGCGACGGGTC
TGTCGCTCGACAGGGCGACGGACGACATTCTGCAGGTGCCGCCGAACACGCAGGGATTCTTCACGATTGA
GCTGATTGCCGACACAGTCGTTTACGGGTTCTGGCTCGGCGCCATCACTCTGTGTGGCTTCGCGGTAGTG
CTTTACGGCTTCAAGAGCGGCCCGGAGGGGACAGACTGCAACAGGCACAACGGCGTCGGCTGCGACAACA
TCTGGACCGCCCGCAGCACCGCCTTTGGAATTCTGTACTTTGGTTTGCTGATCCACTCCTACACGGTGCG
CCACCCGCGCGTCTCCATCTTCAGAATGCGCTGGCTCGACAACAAGTGGATCTTCGGTTCCTGCGTCCTG
GGCGGTGCGCTGTTTGTGCCGATTGTATACATCAACGCGATCGCCCATGGCCTCTTTGTACACAGCATGA
TCACTTGGGAGTGGGGCGTCATTGCGGTCGGCGTCATGACCTTTCTCGCCATCTGCGAAACCTACAAGGT
GATCAAGAACCTGATCTTTCCGATCCGCAAGGTGCTGGTGGAGGTGGACGAGGAGGAGGCCGAGGATGTC
GAGGAGCAGCGCCAGCGCGAGTACAACACCTTTACCCGCACCATGCCGGACGATCGCAACGTGGAGAGGA
TCGCAAACGAGAACCTGCGCATGTCGTTTGCCTCTCTCGCCGGCAGCGTGGCCACCGCCAACACAGGCTC
CTTCCGTGTGCCCGCGCAGCGCCAGAAGAAGAAACGTATTGGGCTCTTCCGCAAGCGCGAGTAA
>AF067496 714, 4184 APLT1
ATGCGACAGATACGCTACCGGGCGCTGCACAACGGTGCTGATGAAGACGCGCGAAGTCGCAACAGCAGCA
CCCCCAATATCTCCACGAGCAGCGCACATGGTCCTCAGCTGCCACAGCGGCACTGCTGTCGGCCACGTGA
TTTCGTGTTTGACGACTCCCTCAGCACTGGCGCGGCGGTGTCAGTGTTGGCCATTCCACAGCAGAAGAAA
CACAGTGTGCGACGGTGGCTGCAGGAAATGTTACATCTGCCGATCGCGCCGTCTCTCGACAACCGCCAAA
TCCCGTTTGGCTACTTCCCTGGCGAATGGTCGCCGGTCGGCTATCCGAGCAACGCGGTGAGCAACCGTCG
CTACACGCTCTCCACGTTTCTGCCGCTGGCGCTGCTCCGTCAGTTTCAGAACTTCTTTAACCTCTTCTAC
CTCTTTCTGGCCTTCTCGCAGGTCATTCCAGTGCTGAAAGTTGGCTTCATCTTCACTTACTTTTCACCGC
TAGTAATGGTGGTGTGCCTCTCGCTGATGAAGGACGCGCTCGATGAGTGGAAGCGATTCCGGCGCGATCG
CATCGCCAACGTCGAGGTGTTCGAGAAACTCACAGGCGGCGGCGACGTGGTCACGGTGCGGTCGTCACAC
ATTCGGGTAGGAGATCTGCTCATCCTGCATCAAGATCAACGGGTGCCGGCCGACTGCGTCGTGTTGCACA
CCTCCGAGAAGTCCGGCACGACGTTCATTCGTACCGACCAACTTGACGGCGAAACGGATTGGAAGCTGCG
CTACCCGTTGAACTCTACCAAGGGTCTAGGATACGCCGAACTTGGTGCCTGCGCTTCAACGTGCACTGTG
GCGCGCCTCACAAAGACATCTACAGCTTCGCCGGCACCCTCGAACTGGCCAACGGACAGAGCGAGTCGAT
CACGATGGAGAATGCCGTCTGGTCGTCGTGTGTGGTGGCGACGGGGACGCTGGTGGTTGCCGTCCTATAC
ACGGGTAGGGAGACGCGCAGCCCTCAACTCGACAAGCCCGCGTAACAAACTTGGCCTCATCGAGCGCGAG
CTCAACCTAATCGCGGTTCTGTGCTTCGCGCTGCTTCTGATCCTTTCCTTTCTTCTCGTAGCGCAACAGC
AGTTCCGTGGCGACTTTGCCGTCATGTTCGTGCGCTTCTTCATCCTCCTCTCGTCCATGATTCCCATCTC
GATGCGCGTGAACGTAGACGTCGGCCGCCTCTGGTACTCGTACGATATGTCGCATGACCCCAACATCCCT
GGCGCCGTGGCTCGCAACGCAAACCTTCCGGAGGAATTGGGTCGGCTGCGCTATCTCTTCTCCGACAAGA
CCGGCACACTCACAAAGAACAAGATGAAGTTTCGCGTGATGCAGGTGGGACCGGACACGGTGCTAACGGA

CCGCTTCACTGATCAGCTGGCGGGCGCGCTGCAGGAGTACTTTGGCACAGGCAACTGGGGAGCCGGGGCT
CGGCTCATGCTCAGCAACGTCTCCGCGCGAGTCAGCGTGAGAAATTCGAAGCAATGGAGCCGTGATGTGC
AGCGCGTTGGCGACCTTATCAGAGCCATTGTCCTGTGCCACAATGTGAGCCCAGTGGTGATGGAGGAGCC
GTTGGCCCTTGGTACCGACGACGAAACAACGGCACTGGGTCGCGGTGCGGCTGCGGCTGTGCCGGCCACA
ACCGATATGAGGAGTCAGCCGACTGACTACCAAGCCTCCTCGCCAGACGAGGTTGCGCTGGTCAAGTTCT
GCGGCTCCCTCGGTATTGTCTTGACCTATCGCGATCTCACAACGATCGCCTTCACCGTTGGCGGCGGTGC
GCAGCTGCACACGTGCACAATCGTCAAAACGTTCCCGTTCTCGTCGGAGCGGAAGTGCATGGGCATCATC
CTGCGCGAGCGCACTGGGAGCTGCAGTGGCGGCAGCTCACAGGAGACCGTCAAGTTCTACATGAAAGGCG
CAGACGTGAAGATGGCGTCGGTGGTGCGCCAATCGGAGTGGCTGGAGGAGTGTTGCCAGGAGCTGGCGCA
GATGGGCCTGCGAACGCTCGTGTTTGCCAGTCGAACGCTCAGCGAGGAGATGCTCGGCGCCTTCCTGCAG
CAGTACGAAGCCGCCATTTCAATCCTTGGTGAAGGTCGCGCAGCAGCAATCGAGGCGGCGATGAAGCTAT
TGGAGACGGACATGACGCTGACAGGGGTGACGGGGGTGGAGGACGAGCTGCAAGACGACGTTGTCGTCTG
TCTCGAGACGCTTGGCATGTGTGGCATCAAGGTGTGGATGCTCACGGGTGACAAGGTCGAGACTGCCATC
ACCATTGGCCGCTCGACTCGGCTCATTCCCCGCCACTGCCGCATCGAGTACCTGTGTGGGATGGCCCCGG
CAGACATTGGTCACCGCCTGCACGAGCTGCAGGAGGAGTTTTGCTGGGGCGTGACGTGCGGCAGCATAGT
GCCGCCATGGACGCTGGTTCTGGATGGAAACGCCCTGGCGTACTGCCTCTCCCACGCCTCCTTCTTCGCG
GACGTGGCCAGAACCGCCTACTCGGTTATAGTGGCGCGATGCTCCCCGACGCAGAAGGCTGCGGTGGTTA
CTGTAATGCGTGATAGCTGTCGTGACGCGCGCGTCGCGGCGATCGGTGACGGAGGCAACGATGTGTCGAT
GATTCAGGCCGCCAACGTGGGGATCGGTATTGAGGGGTTGGAGGGGAAGCAAGCAAGCATGGCCGCTGAT
TTCTCCATTACGAAGTTCTCTCACTGTCTACGACTGATCATGTGGCATGGGCGGAACTCGTACCAGCGCA
CGTGCCGACTCAGCCAGTTCATCATGCACCGCGGCATCGTCTACTCGGTTGTGCAGACGGTCTTCTCGGT
GCTGTTTGCCGGGACGACCATGTCTGTCTTCAACGGTTATCTACTAATGGGGTACTCCACCGTATTTACC
ATGGCGCCAGCCTTCGCATTGGTGCTCGACGAAGACTTCAACGAGTCGGACGTGCACGAGTACCCGTTTC
TCTACAAAGATTTGCTCAAGGCCCGCTCCATGAACACCCGCACCTTTCTGCAGTGGGTGTGGGCATCGTT
CTTCCAAGGAGGGGTCATGATGCTGATGGCGCTACAGCTCTTCAAGTCGGACATGTTCCAGATTGTCACG
ATCGCCTACACATCGCTCCTCATCACCGAGCTGGCCATCGTTGGCGCCACCGCGCATCTTCGCATTCTCT
GGCGAGAGCGCCGTGCTCGCTTCTTTCTCTTCTTGTGCTCTGAGGTGGTGTCTCTGCTGATGTTTTTCCT
CGCCGTCGTCGTGTTGCCCGACACGTTCGATAGAGACTTTTTCTTTTCGTGGAGCTGCTGGTGGCGCGTC
GCGGTAATCTGCCTAGCCAGCATTGCACCACTCATGATATTTCAAATGGTTGGCAAGTACATATTGTTCA
ACCCGCGCATCGCCGTTTCCTGCATGGGATGTCGGACGTGA
>AF069386 135, 734 peroxidoxin
ATGTCCTGCGGTAACGCCAAGATCAACTCTCCCGCGCCGTCCTTCGAGGAGGTGGCGCTCATGCCCAACG
GCAGCTTCAAGAAGATCAGCCTCTCCTCCTACAAGGGCAAGTGGGTCGTGCTCTTCTTCTACCCGCTCGA
CTTCACCTTCGTGTGCCCGACAGAGGTCATCGCGTTCTCCGACAGCGTGAGTCGCTTCAACGAGCTCAAC
TGCGAGGTCCTCGCGTGCTCGATAGACAGCGAGTACGCGCACCTGCAGTGGACGCTGCAGGACCGCAAGA
AGGGCGGCCTCGGGACCATGGCGATCCCAATGCTAGCCGACAAGACCAAGAGCATCGCTCGTTCCTACGG
CGTGCTGGAGGAGAGCCGGGGCGTGGCCTACCGCGGTCTCTTCATCATCGACCCCCATGGCATGCTGCGT
CAGATCACCGTCAATGACATGCCGGTGGGCCGCAGCGTGGAGGAGGTTCTACGCCTGCTGGAGGCTTTTC
AGTTCGTGGAGAAGCACGGCGAGGTGTGCCCCGCGAACTGGAAGAAGGGCGCCCCCACGATGAAGCCGGA
ACCGAAGGCGTCTGTCGAGGGATACTTCAGCAAGCAGTAA
>AF073381 421, 1356 CRK3
ATGTCTTCGTTTGGCCGTGTTACCGCCCGTAGCGGTGACGCTGGGACGCGTGACAGTCTTGACCGGTACA
ATCGCTTGGATGTTTTGGGAGAGGGAACGTATGGTGTTGTGTATCGCGCGGTCGACAAAATCACTGGGCA
GTACGTTGCTCTCAAGAAAGTGCGACTCGATCGCACTGAGGAGGGCATTCCGCAGACTGCGCTGCGCGAG
GTGTCGATTCTACAAGAGTTCGACCACCCCAACATTGTGAACTTGCTTGATGTCATTTGCTCGGACGGGA

AGCTCTACCTTGTCTTCGAGTATGTGGAGGCGGACCTGAAAAAGGCGATTGAAAAGCAAGAGGGTGGATA
CTCTGGAATGGATCTGAAGCGGCTTATTTATCAGCTTTTAGACGGCCTTTACTTTTGCCACCGCCATCGC
ATCATCCACCGTGATCTGAAGCCAGCCAACATCCTCCTGACATCAGGGAACGTCCTTAAATTGGCTGATT
TCGGTCTCGCCCGTGCGTTCCAAGTGCCCATGCACACCTACACGCACGAGGTGGTTACACTGTGGTACCG
TGCCCCTGAGATCCTCCTCGGTGAGAAGCACTACACTCCTGCCGTGGATATGTGGAGTGTCGGCTGTATT
TTCGCTGAGCTAACGCGCCGAAAGGTTCTTTTCCGCGGCGATAGCGAAATCGGGCAGTTGTTCGAGATTT
TTCAAGTGCTGGGGACGCCGACGGACACCGAGGGGTCCTGGCCTGGTGTGTCGCGGCTGCCCGACTACCG
CGACGTATTTCCCAAGTGGACTGCAAAGCGCCTGGGGCAGGTACTACCAGAACTTCATCCGGACGCTATT
GATCTGCTCTCCAAGATGCTCAAGTACGATCCGCGGGAGCGCATATCAGCCAAGGAGGCCCTTCAGCACC
CGTGGTTCAGTGACCTTCGCTGGTAG
>AF083881 917, 1495 mar1
ATGCCTCGCTTGATGCCGCATTATTCTACGAGCAAGACAGCGTTTCTGTGCGTAGATCTCCAAGAAGCGT
TCAGTAAACGCATCGCCAATTTTCCGAATTGCGTGTTCGTTGCCAACCGGCTCGCACGCCTGCATGAGGT
GGTTCCCGAAAACACCAAGTACATTGTCACGGAGCAGTATCCGAAGGGCCTTGGCCACATCGTGCCGGAG
ATTACTTTGCCCCCAAGTGCCCACGTGATCGAGAAGACCCGCTTCTCGTGTGTTGTGCCACAGATGGAGG
AGCTGCTGAAGGACGTGGACAATGTTGTCGTCTTCGGTATTGAGGGGCACGTCTGCGTCCTGCAAACAGT
GGCCGACCTCTTGGACATGAATAAGCGTGTGTTCCTTCCAAAGGACGGCCTGGGCAGTCAAAAGACGACA
GACTTTAAGGCCGCGATGAAGCTGATGGGTTCATGGGGACCCAACTGCGAGATCACCACCTCTGAGTCAA
TTCTTCTTCAGATGACAAAGGATGCCATGGACCCTAACTTCAAGAAGATTTCGAAGCTACTGAGGGAGGA
GCCTCCCATCCCGCTCTAA
>AF093062 263, 1945 PAB1
ATGGCTGCTGCTGTCCAGGAAGCTGCTGCCCCGGTGGCGCATCAGCCGCAGATGGACAAGCCGATCGAGA
TCGCTTCCATCTACGTTGGCGACCTTGACGCCACCATCAACGAGCCGCAGCTGGTGGAGCTCTTCAAGCC
GTTCGGCACCATCCTGAACGTGCGTGTTTGCCGCGACATCATCACGCAGCGCTCGCTGGGCTATGGCTAC
GTCAACTTCGACAACCACGACAGCGCGGAGAAGGCGATCGAGTCCATGAACTTCAAGCGTGTCGGTGACA
AATGCGTACGCCTTATGTGGCAGCAGCGCGACCCCGCCCTCCGCTACAGCGGCAACGGCAACGTTTTCGT
GAAGAACCTGGAGAAGGATGTGGACAGCAAGAGCCTGCACGACATCTTCACCAAGTTCGGCTCGATTCTC
TCCTGCAAGGTAATGCAGGACGAGGAGGGCAAGAGCCGCGGCTACGGCTTCGTTCACTTCAAAGACGAGA
CGTCCGCCAAGGACGCTATTGTGAAGATGAACGGCGCTGCCGATCACGCCTCAGAGGACAAGAAGGCGCT
CTACGTGGCCAACTTCATTCGCCGCAATGCACGCCTTGCCGCGCTCGTGGCCAACTTCACGAACGTGTAT
ATCAAGCAGGTGCTGCCGACCGTGAACAAGGACGTCATCGAGAAGTTCTTCGCAAAGTTTGGCGGCATCA
CGTCCGCTGCAGCCTGCAAGGACAAGAGCGGCCGCGTGTTCGCCTTCTGCAACTTCGAAAAGCACGACGA
CGCCGTCAAGGCGGTTGAAGCGATGCACGACCATCACATCGACGGCATCACGGCACCTGGTGAGAAGCTG
TACGTACAGCGCGCCCAGCCGCGCAGCGAGCGCCTCATTGCCCTTCGACAGAAGTACATGCAGCACCAAG
CCCTTGGTAACAACCTGTACGTGCGCAACTTCGACCCTGAGTTCACGGGCGCGGACCTGCTCGAGCTCTT
CAAGGAGTATGGCGAGGTCAAGAGCTGCCGTGTGATGGTGAGCGAGAGTGGGGTGAGCCGCGGCTTCGGT
TTCGTGAGTTTTTCGAACGCCGACGAGGCCAACGCCGCTCTGCGCGAGATGAATGGTCGCATGCTGAACG
GCAAGCCCCTCATCGTGAACATTGCCCAACGCCGCGATCAGCGCTACACAATCGTGCGCCTGCAGTTCCA
GCAGCGCCTGCAGATGATGATGCGTCAGATGCACCAGCCGATGCCGTTTGTCGGCAGCCAGGGCCGTCCG
ATGCGTGGCCGCGGTGGTCGCCAGCAGCTGGGTGGTCGCGCGCAGGGCCATCCGATGCCGATGCCATCAC
CACAGCAGCCGCAGGGTGCCGCGCAGCCACAGGGTTTCGCCACGCCGTCGGCAGTCGGTTTCGTGCAAGC
GACGCCGAAGCACTCCCCTGGCGATGTCCCCGAGACGCCGCCGCTGCCGCCGATCACACCACAGGAACTC
GAAAGCATGTCGCCCCAGGAGCAGCGCGCCGCCTTGGGTGACCGTCTCTTCCTAAAGGTGTACGAGATTC
CGCCAGACGTGGCGCCGAAGATCACGGGTATGTTCCTCGAGATGAAACCCAAGGAGGCGTACGAGTTGCT
CAACGACCAGAAGCGACTCGAGGAGCGCGTGACAGAGGCGCTATGCGTGCTCAAGGCGCATCAGACGGCG

TAA
>AF099106 1, 1086 p45
GATGAAGAAGAGGAGGACACCACCATCAACAACTCCGACGTGGTGGTGCGCTACAAGAAGGCCGCAACGT
GGTGCAATGAAACGTTGCGCGTGCTTATCGATGCCACAAAACCTGGCGCCAAGGTGTGCGACCTGTGCCG
CCTCGGTGATGACACCATCACCGCCAAGGTCAAGACAATGTTCAAAGGCACGGAAAAAGGCATCGCTTTC
CCGACCTGCATCTCGGTCAACAACTGCGTATGCCACAACAGCCCTGGCGTGTCGGACGAGACGACGCAGC
AAGAGATCGCGATGGGTGACGTCGTGCACTACGACCTGGGCATCCACGTGGACGGCTACTGCGCCGTCGT
CGCGCACACCATTCAGGTGACAGAGGACAATGAGCTTGGCAAGGACGAGAAGGCGGCGCGCGTCATTACA
GCGGCGTACAACATCCTGAACACGGCGCTGCGCCAGATGCGTCCCGGTACGACCATCTACCAGGTGACAG
ACGTAGTTGAGAAGGCTGCGGAGCACTACAAGGTGACTCCGGTAGACGGCGTCCTCTCGCATATGATGAA
GCGCTACATCATAGACGGATACCGCTGTATCCCGCAGCGCAGGGTCGCGGAGCACATGGTGCACGACTAC
GATCTCGAGAAAGCGCAGGTGTGGACGCTAGACATTGTCATGACCTCCGGCAAGGGCAAGCTGAAGGAGC
GCGATGCGCGGCCGTGCGTGTTCAAGGTGGCTCTGGACTCCAACTACTCTGTGAAAATGGAAAGCGCGAA
GGAGGTTCAGAAGGAAATCGACTCCAAGTATGCCACCTTCCCCTTTGCCATCCGCAACCTGGAGGCCAAG
AAGGCCCGCCTCGGTCTCAACGAGATGGCGAAGCACGGTGCTGTCATCCCGTACCCTATTCTCTTCGAAA
AGGAAGGCGAGGTCGTCGCCCATTTCAAGATTACGGTGCTCATCAGCAACAAGAAGATTGAGCCGATTAC
CGGCCTGAAGCCGCAGAAGGCCCCGGCGCTCGAGCCATACACGGACGAGATGCTGCTTGCGACGAACAAG
CTCTCGCTGTCGCTAGAGAAGAAGGCGGCGAAGTAG
>AF099107 70, 645 p21
ATGAGCATTATCAAGGAGGACGACGCCGTGGGCTGCTACATGACGGTGACCCTCGTGGACGACACCAAGG
TGGAGGGTACCATCTTCACCTACAATTCCAAGGAGGGCATCATAGTACTCCTGTCCCTCCGCGACGATCA
GACGAACATGAAGCTAATCCGCACTCCGTACATCAAAGACTTCAGCCTTTCACACGCTGAGGAGGGAGCG
CACCTGCCCCCGGCACTGGACTCCTTCAACGAGCTTCCGTCCATGCACGCCGGCCGCGACAAGTCCATCT
TCAAGCACGCCAGCACGCAGCTCAAGAACGCCGAGGCGAACCGCGAAAAGCACTTCAACTCTGTCACGAC
CGACACACCGATTGCCACACTTGATGCGTACCTCAAGCTCCTGCGGCTATACCCCTTAATTGAGTGGAAC
AGCGACGAGGGTGTCATCCAGGTCTCGGACACCGTCATTGTCGTAGGAGACCCCGACTGGCGGACGCCCA
AGGCAATGCTGGTGGACGGCGCCCCTGAGAAGGACAGACCGCTTGTAGATCGCCTGCAGGTTGCGCTCGG
AAACGGCAAGAAGTGA
>AF099108 72, 395 H2B
ATGGCTTCTTCCCGCAAGGCTTCCAACCCGCACAAGTCGCACCGCAAGCCGAAGCGCTCGTGGAACGTGT
ACGTGGGCCGCTCGCTGAAGGCGATCAACGCCCAGATGTCGATGTCGCACCGCACGATGAAGATCGTGAA
CTCGTACGTGAACGACGTGATGGAGCGCATCTGCACTGAGGCCGCGTCGATTGTTCGCGCGAACAAGAAG
CGCACGTTGGGTGCGCGCGAGGTGCAGACGGCGGTGCGCATTGTGCTGCCGGCGGAGCTCGCGAAGCATG
CCATGGCTGAGGGCACGAAGGCCGTGTCGAGCGCGTCCCGCTAA
>AF109296_0 2971, 10930 H1A
ATGAGCTCCAAAAAGTACGAGCTGGACGCCGCCGCGTTCGAGGACAAGCCCGAGAGCCATTCCGATGCGG
AGATGACCCCACAGAAGCCTCAGCGCCGCCAGTCTGTGCTGTCGAAGGCCGTGAGCGAGCATGATGAACG
CGCGACGGGGCCTGCGACAGACCTGCTACCGCCGTCGAAAGGCCTGACGACAGAGGAGGCGGAGGAGCTG
CTGAAGAAGTATGGGCGCAACGAGCTGCCGGAGAAGAAGACGCCGAGCTGGCTGATCTATGTGCGCGGTC
TGTGGGGCCCGATGCCTGCCGCGCTGTGGATTGCAATCATCATCGAGTTTGCGCTGGAGAACTGGCCCGA
CGGCGCCATCCTGTTCGCCATCCAGATCGCGAACGCGACGATTGGGTGGTACGAGACGATCAAGGCCGGC
GACGCCGTTGCTGCACTGAAGAACTCACTGAAGCCGACCGCGACTGTGTACCGCGACTCGAAGTGGCAGC
AGATCGACGCAGCGGTGCTGGTGCCTGGCGACCTTGTCAAGCTTGCTTCCGGCTCCGCGGTGCCTGCGGA
CTGCAGCATCAACGAGGGCGTGATCGACGTGGACGAGGCTGCGCTAACGGGCGAGTCGCTGCCCGTGACG
ATGGGCCCGGAACACATGCCGAAGATGGGGTCGAACGTTGTACGCGGCGAGGTGGAGGGCACGGTGCAGT

ACACCGGCTCGCTGACGTTCTTCGGGAAAACCGCTGCGCTTCTGCAGTCTGTGGAGTCGGATCTGGGCAA
CATCCACGTGATTCTCAGGCGTGTGATGTTCTCGCTGTGCGCGATCTCGTTCATGCTGTGCATGTGCTGC
TTCATCTACCTGCTGGCAAGGTTCTACGAGACGTTCCGCCACGCGCTGCAGTTCGCGGTGGTGGTGCTGG
TCGTGTCGATCCCGATTGCCCTGGAGATTGTGGTGACGACGACGCTGGCGGTGGGGTCGAAGCACCTGTC
GAAGCACAAGATCATCGTGACGAAGCTGTCGGCGATCGAGATGATGTCCGGCGTGAACATGCTGTGCTCG
GACAAGACGGGGACGTTGACGCTGAACAAGATGGAGATCCAGGAGCAGTGCTTCACGTTCGAGGAGGGCA
ACGATCTGAAATCGACGCTGGTGCTGGCGGCGCTTGCTGCGAAGTGGCGTGAGCCGCCGCGCGATGCGCT
GGACACGATGGTGCTGGGCGCGGCGGACCTGGACGAGTGCGACAACTACCAGCAGCTGAACTTCGTGCCG
TTCGACCCGACGACGAAGCGCACTGCGGCGACGCTTGTGGACCGGCGCAGCGGCGAGAAATTCGACGTGA
CGAAGGGCGCGCCGCACGTGATCCTGCAGATGGTGTACAACCAGGACGAGATCAACGACGAGGTGGTGGA
CATCATCGACAGCCTGGCTGCGCGCGGTGTCCGCTGCCTGTCTGTGGCGAAGACGGATCAGCAGGGCCGC
TGGCACATGGCCGGCATTCTGACGTTCCTGGACCCGCCGCGCCCGGACACGAAGGATACGATCCGTCGCA
GCAAGGAGTACGGCGTGGACGTGAAGATGATCACTGGCGACCACTTACTGATCGCAAAGGAGATGTGCCG
CATGCTGGACCTCGACCCGAACATCCTGACGGCGGACAAGCTGCCGCAGATCAAGGACGCGAACGACCTG
CCGGAAGACCTCGGTGAGAAGTACGGCGACATGATGCTGTCTGTCGGCGGGTTTGCGCAGGTGTTCCCGG
AGCACAAGTTCATGATTGTGGAGACGCTGCGCCAGCGCGGGTACACGTGTGCGATGACGGGCGACGGCGT
GAACGACGCGCCTGCGCTGAAGCGCGCCGACGTGGGCATTGCGGTGCACGGTGCGACGGACGCTGCGCGC
GCTGCGGCGGACATGGTGCTGACGGAGCCCGGTCTGAGCGTGGTGGTGGAGGCCATGCTCGTCTCGCGTG
AGGTGTTCCAGCGCATGCTGTCGTTCCTGACGTACCGTATCTCTGCGACGCTACAGCTTGTGTGCTTCTT
CTTCATTGCGTGCTTCAGCCTGACGCCGAAAGCCTACGGCAGCGTGGATCCACACTTCCAGTTCTTCCAC
CTGCCCGTGCTGATGTTCATGCTGATCACGCTGCTGAACGACGGCTGCCTGATGACGATCGGCTACGACC
ACGTGATCCCGTCTGAGCGGCCGCAGAAGTGGAATTTGCCGGTGGTGTTCGTGAGCGCGTCCATTCTGGC
GGCGGTCGCGTGTGGGTCGTCGCTGATGCTGCTGTGGATCGGCCTGGAGGGCTACAGCTCGCAGTACTAC
GAGAACTCGTGGTTCCACCGCCTTGGCCTCGCGCAGCTGCCGCAGGGCAAGCTGGTGACGATGATGTACC
TGAAGATCTCGATTTCGGACTTCCTGACGCTGTTCTCGTCACGCACGGGTGGCCACTTCTTCTTCTACAT
GCCGCCAAGCCCGATCCTGTTCTGCGGCGCGATCATCTCACTACTGGTATCAACGATGGCTGCGTCGTTC
TGGCACAAGTCGAGGCCGGACAATGTGCTGACGGAGGGTCTCGCGTGGGGCCAGACGAACGCGGAGAAGC
TGCTGCCACTGTGGGTGTGGATCTACTGCATTGTGTGGTGGTTCGTGCAGGATGTCGTGAAGGTCTTGGC
GCACATCTGCATGGACGCCGTGGACCTGTTCGGGTGCGTGTCGGACGCTTCGGGCTCCGGGCCGATCAAG
CCATACAGCGACGACATGAAGGTTAACGGGTTCGAGCCGGTAAAGAAGCCTGCTGAGAAGTCGACGGAGA
AGGCTCTGAACTCGAGCGTTTCCAGTGCGTCGCACAAGGCACTGGAGGGCCTGCGGGAGGATACGCACTC
GCCGATAGAGGAGGCCAGCCCCGTGAACGTGTACGTGTCCCGCGATCAGAAGTAG
>AF109296_1 8006, 10930 H1B
ATGAGCTCCAAAAAGTACGAGCTGGACGCCGCCGCGTTCGAGGACAAGCCCGAGAGCCATTCCGATGCGG
AGATGACCCCACAGAAGCCTCAGCGCCGCCAGTCTGTGCTGTCGAAGGCCGTGAGCGAGCATGATGAACG
CGCGACGGGGCCTGCGACAGACCTGCTACCGCCGTCGAAAGGCCTGACGACAGAGGAGGCGGAGGAGCTG
CTGAAGAAGTATGGGCGCAACGAGCTGCCGGAGAAGAAGACGCCGAGCTGGCTGATCTATGTGCGCGGTC
TGTGGGGCCCGATGCCTGCCGCGCTGTGGATTGCAATCATCATCGAGTTTGCGCTGGAGAACTGGCCCGA
CGGCGCCATCCTGTTCGCCATCCAGATCGCGAACGCGACGATTGGGTGGTACGAGACGATCAAGGCCGGC
GACGCCGTTGCTGCACTGAAGAACTCACTGAAGCCGACCGCGACTGTGTACCGCGACTCGAAGTGGCAGC
AGATCGACGCAGCGGTGCTGGTGCCTGGCGACCTTGTCAAGCTTGCTTCCGGCTCCGCGGTGCCTGCGGA
CTGCAGCATCAACGAGGGCGTGATCGACGTGGACGAGGCTGCGCTAACGGGCGAGTCGCTGCCCGTGACG
ATGGGCCCGGAACACATGCCGAAGATGGGGTCGAACGTTGTACGCGGCGAGGTGGAGGGCACGGTGCAGT
ACACCGGCTCGCTGACGTTCTTCGGGAAAACCGCTGCGCTTCTGCAGTCTGTGGAGTCGGATCTGGGCAA
CATCCACGTGATTCTCAGGCGTGTGATGCTTGCGCTGTGCGCGATCTCGTTCATACTGTGCATGTGCTGC

TTCATCTACCTGCTGGCAAGGTTCTACGAGACGTTCCGCCACGCGCTGCAGTTCGCGGTGGTGGTGCTGG
TCGTGTCGATCCCGATTGCCCTGGAGATTGTGGTGACGACGACGCTGGCGGTGGGGTCGAAGCACCTGTC
GAAGCACAAGATCATCGTGACGAAGCTGTCCGCGATCGAGATGATGTCCGGCGTGAACATGCTGTGCTCG
GACAAGACGGGGACGTTGACGCTGAACAAGATGGAGATCCAGGAGCAGTGCTTCACGTTCGAGGAGGGCA
ACGATCTGAAATCGACGCTGGTGCTGGCGGCGCTTGCTGCGAAGTGGCGTGAGCCGCCGCGCGATGCGCT
GGACACGATGGTGCTGGGCGCGGCGGACCTGGACGAGTGCGACAACTACCAGCAGCTGAACTTCGTGCCG
TTCGACCCGACGACGAAGCGCACTGCGGCGACGCTTGTGGACCGGCGCAGCGGCGAGAAATTCGACGTGA
CGAAGGGCGCGCCGCACGTGATCCTGCAGATGGTGTACAACCAGGACGAGATCAACGACGAGGTGGTGGA
CATCATCGACAGCCTGGCTGCGCGCGGTGTCCGCTGCCTGTCTGTGGCGAAGACGGATCAGCAGGGCCGC
TGGCACATGGCCGGCATTCTGACGTTCCTGGACCCGCCGCGCCCGGACACGAAGGATACGATCCGTCGCA
GCAAGGAGTACGGCGTGGACGTGAAGATGATCACTGGCGACCACTTACTGATCGCAAAGGAGATGTGCCG
CATGCTGGACCTCGACCCGAACATCCTGACGGCGGACAAGCTGCCGCAGATCAAGGACGCGAACGACCTG
CCGGAAGACCTCGGTGAGAAGTACGGCGACATGATGCTGTCTGTCGGCGGGTTTGCGCAGGTGTTCCCGG
AGCACAAGTTCATGATTGTGGAGACGCTGCGCCAGCGCGGGTACACGTGTGCGATGACGGGCGACGGCGT
GAACGACGCGCCTGCGCTGAAGCGCGCCGACGTGGGCATTGCGGTGCACGGTGCGACGGACGCTGCGCGC
GCTGCGGCGGACATGGTGCTGACGGAGCCCGGTCTGAGCGTGGTGGTGGAGGCCATGCTCGTCTCGCGTG
AGGTGTTCCAGCGCATGCTGTCGTTCCTGACGTACCGTATCTCTGCGACGCTACAGCTTGTGTGCTTCTT
CTTCATTGCGTGCTTCAGCCTGACGCCGAAAGCCTACGGCAGCGTGGATCCGAACTTCCAGTTCTTCCAC
CTGCCCGTGCTGATGTTCATGCTGATCACGCTGCTGAACGACGGCTGCCTGATGACGATCGGCTACGACC
ACGTGATCCCGTCTGAGCGGCCGCAGAAGTGGAATTTGCCGGTGGTGTTCGTGAGCGCGTCCATTCTGGC
GGCGGTCGCGTGTGGGTCGTCGCTGATGCTGCTGTGGATCGGCCTGGAGGGCTACAGCTCGCAGTACTAC
GAGAACTCGTGGTTCCACCGCCTTGGCCTCGCGCAGCTGCCGCAGGGCAAGCTGGTGACGATGATGTACC
TGAAGATCTCGATTTCGGACTTCCTGACGCTGTTCTCGTCACGCACGGGTGGCCACTTCTTCTTCTACGT
GCCGCCAAGCCCGATCCTGTTCTGCGGCGCGATCATCTCACTACTGGTATCAACGATGGCTGCGTCGTTC
TGGCACAAGTCGAGGCCGGACAATGTGCTGACGGAGGGTCTCGCGTGGGGCCAGACGAACGCGGAGAAGC
TGCTGCCACTGTGGGTGTGGATCTACTGCATTGTGTGGTGGTTCGTGCAGGATGTCGTGAAGGTCTTGGC
GCACATCTGCATGGACGCCGTGGACCTGTTCGGGTGCGTGTCGGACGCTTCGGGCTCCGGGCCGATCAAG
CCATACAGCGACGACATGAAGGTTAACGGGTTCGAGCCGGTAAAGAAGCCTGCTGAGAAGTCGACGGAGA
AGGCTCTGAACTTGAGCGTTTCCAGTGGGCCGCACAAGGCACTGGAGGGCCTGCGGGAGGATACGCATGT
GCTGAATGAGAGTACGAGCCCCGTGAACGCGTTCTCGCCGAAGGTTAAGAAGTAG
>AF109875 1333, 3528 opb
ATGTCGTCGGACAGCTCCGTCGCGGCCTCTGCGCAGCCGCCGATCGCCGCCAAGAAGCCGCACCGCGTCA
AGTTCGGGTACGTGGAGGGTGAGGACCGCGGCCCGAACCCGATGAACCCGCCGCGCTACCGCGAGGACCC
GTATTTTTGGATGCGCGATGACGATCGTAAGGATCCGGCCGTGATTGAGCACCTCAACAAGGAGAAGGTC
TACTTCCAGGCGCGCAGCGCCGACATTGCGCAGCTGCGCGACGACATTTACGCAGAGCACATATCGCACA
TCAATGAGGACGACATGTCCGCGCCGTACGTGTACGGCAAGTACCGGTACTACACCCGCGAGGTGAAGGG
TAAGCCGTACAAGATTTACTGCCGCGTGTTCACGGACAAGGAGCCGGGCGACGTCGCGGCGGAAGAGGTC
ATCATCGATGTCAACCAGGTCGCCGAGGGCAAAGCGTTCTGTGACGTGATGGAGGTGAAGCCGGCACCGC
CGGAGCATGACCTTGTAGCCTTTTCTGTGGACATGAGCGGTAACGAGGTGTACACGATCGAATTTAAAAG
GATTTCGGACCCGTCCCAAACCATCGCCGACAAGGTGAGTGGCACCAATGGCGAGATCGTGTGGGGCCCG
GACCACACCTCCTTATTCTACGTGACCAAAGACGAGACGCTGCGCGAAAACAAGGTGTGGCGCCACGTGA
TGGGCAAGCTGCAGTCCGAGGACGTCTGCCTCTACGAGGAGCACAACCCGCTGTTCAGTGCCTTCATGTA
CAAGGCCGCGGACACAAACACACTTTGCATCGGCTCGCAGTCACCGGAGACGGCGGAAGTGCACCTGCTT
GATCTGCGCAAGGGCAACGCCCACAATACGCTGGAAATTGTGCGGCCGCGCGAGAAGGGTGTGCGCTACG
ACGTGCAGATGCACGGCACCAGCCATCTTGTGATCCTCACCAACGAAGGTGGGGCGGTGAACCACAAGCT

TCTCATAGCGCCGCGTGGGCAGCCGAGCGACTGGTCGCATGTGCTGGTGGATCACAGCGAGGATGTGTTT
ATGGAGAGCATCGCGGTGCGCTCGAACTACCTCGTCGTGGCAGGCCGCCGCGCCGGGCTGACGCGCATCT
GGACGATGATGGCGGACTCGCAGGATGGTGTCTTCCAGGCTGGCACCGGGCTGCGCGAGGTGGTGATGGA
GGAGCCGATCTTCACGGTGCACCTCGTGGAGTCCCAGATGTTGGAGTACGAAGAGCCGACGTTCCGCATG
GAGTACTCCTCCCTTGCCACGCCGAACACCTGGTTCGACGTCAGCCCGCAGGACACTCTCGCACCGCTGG
TGAAAGTGCGCGAGGTCGGCGGTGGCTTCGACGCCGCCAACTACAAGGTGGAGCGCCGCTTCGCTACCGC
ACCGGACCAGACCAAGATCCCGCTCTCAGTTGTGTGCCACAAAGACCTCGACATGAGCCAGCCGCAGCCG
TGCATGCTGTACGGGTACGGCAGCTACGGCCTTAGCATGGACCCGCAGTTCAGTATTCAGCACCTGCCAT
ACTGTGACCGCGGCATGATCTTCGCCATAGCCCACATCCGCGGCGGCAGTGAGCTGGGCCGTGCATGGTA
CGAGATCGGCGCCAAGTACCTCACGAAGCGCAACACCTTTTCCGACTTCATCGCGGCAGCCGAATTCCTG
GTGAACGCGAAATTGACGACGCCGTCGCAGCTGGCCTGCGAGGGGCGTAGCGCCGGTGGCCTGCTGATGG
GCGCCGTGCTGAACATGCGACCCGATCTCTTCAAGGTGGCGCTCGCCGGCGTACCGTTCGTGGATGTCAT
GACGACCATGTGCGACCCCAGCATTCCCTTGACGACGGGCGAGTGGGAGGAGTGGGGCAACCCGAACGAG
TACAAGTACTACGACTACATGCTGAGCTACAGCCCCATGGACAACGTCCGCGCGCAGGAGTACCCGAACA
TCATGGTCCAGTGCGGCCTGCACGACCCCCGCGTCGCCTATTGGGAGCCGGCCAAGTGGGTGAGCAAGCT
GCGTGAGTGCAAGACGGACAACAACGAAATTCTGCTGAACATAGACATGGAGAGCGGGCACTTCTCCGCC
AAGGATCGCTACAAGTTCTGGAAGGAGTCGGCTATCCAGCAAGCGTTCGTGTGCAAGCACCTGAAGAGCA
CCGTGCGCCTGCTGGTTCGCAGGTAA
>AF110372 1, 274 putative glycoprotein
GATCGAGGCAGAGAGGCAGAAGCTGAAGGAGCTGCAGGAGGAGCACGAGGGGGAGCAGGAGGAGGCGCGC
CAGCGTCGCGTCGCGGAGGAGAAGGAGGCACAGAAGAGGGCCGAGAAGAAGGCCGAGGAGGCCGAGGGCG
AGCTTGCTGCGACGCGCCGGCAGAGGAAGGGGGAGTTGGAGGAGCTGCAGCGCCACGCGGAGGAGGAGGA
GAAGCAGCGCATCGAGATGGTGCGCAAGCAGCGTGAGGAGACCCAAAAGAAGCGCGAGGAGATC
>AF155593 1, 1305 hmgr
ATGCGCCGCTCTCTGCTGCTTGCCTGCTCCGCCGCCAAGGGTGAGAGCTGGGCATCCATGTCCGATACAG
AGATCATGAAGCAGGTGGAGAATAAGAAGATTGCATTTCACGGCCTTGAGCAGGCTCTTGCACCCGACTA
TGACCGCGCCATCGCCATTCGTCGTGAGATTGTGAAGAAAAAGATATGTCCATCTCCAGCTGCCACACAT
CCGCTGGAGAGGGTTCCCTACAAGAACTACGACTGGAGCAGTGTTGTGGGACAGAGCTGCGAGAACATCC
TCGGCTACGTTCCGGTACCGGTAGGGTTGGCTGGTCCGCTGCTGTTGGATGGAAAGGAAGTGGCACTGCC
GATGGCGACGACGGAGGGTGCGCTGGTCGCGAGTGCGCACCGCGGTGCTCGCGCCATCAACCTGAGCGGC
GGATGCCGGACAGCGGTGCTGAAGGAAGGCATGACGCGCGCCCCTGTAGTGGAGGTGAACTCCTTCGACG
AGGCCATCACCGTGATCAAGTTCTGTGAGGAGCGGTTCGACGTTCTAAGGGAGGCCTTCGAGTCGACGAC
CCGCTTCGGGAAGTTGCTGAGCATCAAGTGTGCCATGGCGGGTCGCCAGGTGCATCTGCGCTTCTCTGCC
TTTACCGGAGATGCGATGGGCATGAATATGATCACGAAGGGCTGCGACAAGGCGCTGCAGGTGCTGCAGC
AGCACATTCCCTCTGTGCGAGTGCTGACCTTGTCAGGCAACTTCTGCACTGACAAGAAACCGTCCGCACT
GAACTGGGTGGAGGGACGCGGGAAGTCGGTCGTAGCGGAGGCCGTCATCAAGCGTGATGTGGTCGAGAGT
GTTCTCAAGTGCACGGTGGACAGTGTTGTGTCGCTGAATGTAACTAAGAACCTGCGCGGATCTGCGCTTG
CCGGTTCCATCGGCGGCTTCAACGCACACGCTGCCAACATCGTGGCCGCGCTCTACATCGCCACTGGCCA
GGACCCCGCACAGGTAGTGGAGTCCGCAACCTGCATGACGACGGTTGACAAGGCTGGTGAGGATCTCGTG
ATCAGCCTCATGATGCCGAGCATTGAGGTCGGCGCCGTAGGCGGTGGTACAGGTCTGTCGTCGCAGCGCG
CCATGCTGGAGCTGATGGGGTGCGCGGGCTCTAACAAGGAGGACCCAGGTGCCCACTCCCGCCAGATCGC
GCGTGTCGTTGCTGGCGCAGTCATTTGCGGTGAGCTGTCCCTCTTGTCCGGCCTTGCAGCTGGCCACTTA
CTAAGCGCCCACATGAAGCTGAACCGCAAGCCTCCGACTCCGTAA
>AF199016 1, 1932 EF2-1
GAGAAGGGCACTGTGGCGATCGGCTCTGGCCTGCAGGCGTGGGCGTTCTCGCTGACCCGCTTTGCGAACA

TGTATGCGGCGAAGTTCGGCGTGGACGAGCTGAAGATGCGCGAGCGCCTGTGGGGCGACGACTTCTTTGA
CGCGAAGAACAAGAAGTGGATCAAGCAGGAGACGAACGCCGATGGCGAGCGCGTGCGCCGCGCGTTCTGC
CAGTTCTGCCTAGACCCCATCTACCAGATCTTCGACGCTGTGATGAACGAGAAGAAGGACAAGGTGGACA
AGATGCTCAAGTCGCTGCACGTGACGCTGACGGCTGAGGAGCGCGAGCAGGTGCCGAAGAAGCTTCTGAA
GACGGTGATGATGAAGTTCCTGCCGGCTGCTGAGACGCTGCTACAGATGATCGTGGCGCACCTGCCGTCG
CCCAAGAAGGCGCAGGCGTACCGTGCGGAGATGCTGTACTCTGGCGAGGCGTCGCCGGAGGACAAGTACT
TCATGGGTATCAAGAACTGCGACCCCGCTGCGCCGCTCATGCTGTACATCAGCAAGATGGTGCCGACGGC
CGACCGCGGCCGCTTCTTCGCCTTTGGCCGCATCTTCTCCGGTAAGGTGCGCAGCGGCCAGAAGGTGCGC
ATCATGGGTAACAACTACGTCTACGGCAAGAAGCAGGACCTGTACGAGGACAAGCCTGTGCAGCGCTCCG
TGCTGATGATGGGCCGCTACCAGGAGGCCGTGGAGGACATGCCGTGCGGTAACGTGGTGGGCCTTGTGGG
CGTGGACAAGTACATCGTGAAGTCCGCGACGATCACGGACGATGGCGAGAGCCCGCACCCGCTGCGCGAC
ATGAAGTACTCTGTGTCGCCCGTCGTGCGTGTGGCCGTGGAGGCGAAGAACCCGTCCGACCTGCCGAAGC
TTGTGGAGGGCCTGAAGCGCCTTGCCAAGTCCGACCCGCTGGTGGTGTGCAGCATTGAGGAGTCTGGCGA
GCACATTGTTGCCGGCGCTGGCGAGCTTCACCTTGAGATTTGCCTGAAGGATCTCCAGGAGGACTTCATG
AACGGCGCGCCGCTGAAGATCTCCGAGCCGGTGGTGTCGTTCCGCGAGACGGTGACGGATGTGTCGTCGC
AGCAGTGCCTGTCGAAGTCTGCGAACAAGCACAACCGTCTCTTCTGCCGCGGTGCGCCGCTGACAGAGGA
GCTGGCGCTGGCGATGGAGGAGGGCACCGCTGGTCCCGAGGCGGATCCGAAGGTGCGCGCGCGCTTCCTT
GCCGACAACTACGAGTGGGACGTGCAGGAGGCCCGCAAGATCTGGTGCTACGGCCCGGACAACCGCGGCC
CGAACGTGGTCGTGGATGTGACGAAGGGTGTCCAGAACATGGCTGAGATGAAGGACTCCTTTGTTGCGGC
GTGGCAGTGGGCGACCCGCGAGGGTGTGCTGTGCGACGAGAACATGCGCGGCGTGCGCGTGAACGTGGAG
GATGTGACGATGCACGCGGATGCCATTCACCGTGGTGGTGTCCAGATCATCCCGACGGCGCGCCGCGTGT
TCTACGCGTGCTGCCTGACGGCGTCGCCGCGCCTGATGGAGCCGATGTTCGTGGTGGACATCCAGACCGT
GGAGCACGCCATGGGCGGCATCTACGGTGTGCTGACCCGCCGCCGTGGCGTGATCATTGGCGAGGAGAAC
CGCCCCGGCACGCCCATCTACAACGTGCGCGCGTACCTGCCGGTTGCGGAGTCGTTCGGCTTCACTGCCG
ACCTGCGCGCTGGAACTGGCGGCCAGGCCTTCCCGCAGTGTGTGTTCGACCACTGGCAGGAGTACCCCGG
TGACCCGCTGGAGCCGAAGTCGCTGGCCAACACGACGACGCTTGGCATCCGCACGCGCAAGGGCCTGAAG
CCGGATATCCCCGGCCTGGACCAGTTCATGGATAAGTTGTAA
>AF199018 48, 869 RS4
ATGGCCAAGAAGCACCTCAAGCGCTTGTATGCGCCCAAGGACTGGATGCTGAGCAAGCTGACCGGCGTGT
TCGCGCCGCGTCCGCGTCCGGGTCCGCACAAGCTGCGCGAGTGCCTGCCGCTGCTGGTGATCATCCGCAA
CCGGCTGAAGTACGCGCTGAACGCGCGCGAGGGTGAGATGATCCTGCGCCAGGGTCTGGTGCACGTGGAC
AACCACCCGCGCCGCGACGGCAAGTATCCCGCCGGTTTCATGGACGTGGTCGAGATCCCGAAGACGGGCG
ACCGCTTCCGCCTGATGTACGACGTCAAGGGCCGCTTCGCGTTGGTGAACCTGTCCGAGGCGGAGGCGCA
GATCAAGCTGATGAAGGTTGTGAACCTGTACACGGCCACCGGCCGCGTGCCGGTCGCTGTGACGCACGAC
GGCCACCGCATCCGCTACCCGGACCCGCACACCTCCATTGGTGACACCATCGTGTACAACGTCAAGGAGA
AGAAGTGCGTGGACCTGATCAAGAACCGCCAGGGCAAGGCCGTGATCGTGACCGGTGGCGCCAACCGCGG
CCGCATCGGCGAGATCGTGAAGGTGGAGTGCCACCCCGGTGCGTTCAACATTGCGCACCTGAAGGACGCG
TCCGGCGCCGAGTTCGCCACCCGCGCCGCGAACATCTTCGTGATCGGCAAGGACCTGAACAACCTGCAGG
TAACGGTGCCGAAGCAGCAGGGCCTGCGCATGAACGTGATCCAGGAGCGCGAGGAGCGCCTGATCGCGGC
GGAGGCCCGCAAGAACGCGCCGGCTCGTGGTGCCCGCAGGGCCCGCAAGTGA
>AF199019 1, 603 alpha tubulin
CTGACGGAGTTCCAGACGAACCTTGTGCCGTACCCGCGCATCCACTTCGTGCTGACAAGCTACGCTCCGG
TGGTGTCTGCCGAGAAGGCGTACCACGAGCAGCTGTCCGTCGCGGACATCACGAACTCGGTGTTTGAGCC
TGCTGGCATGCTGACGAAGTGCGATCCTCGCCACGGCAAGTACATGTCGTGCTGCCTCATGTACCGCGGT
GATGTCGTGCCGAAGGATGTCAACGCCGCGATTGCGACGATCAAGACGAAGCGCACAATTCAGTTCGTGG

ACTGGTGCCCGACCGGCTTCAAGTGCGGCATCAACTACCAGCCGCCGACCGTTGTGCCCGGCGGTGACCT
CGCGAAGGTGCAGCGCGCCGTGTGCATGATTGCCAACTCGACCGCGATCGCTGAGGTGTTTGCCCGCATC
GACCACAAGTTCGACCTGATGTACAGCAAGCGCGCGTTTGTGCACTGGTACGTGGGTGAGGGCATGGAGG
AGGGCGAGTTCTCCGAGGCGCGCGAGGATCTCGCTGCGCTGGAGAAGGACTACGAGGAGGTTGGCGCCGA
GTCCGCCGACGACATGGGCGAGGAGGACGTCGAGGAGTACTAA
>AF202062 1, 225 hexokinase
GATTTGGGAGGAACTAATTTCCGCGTGTTGCGTGTGAGCCTGCGCGGCGGCAAGGTGGACGACCGCACCG
ACTCGAAGTTCGTCATCCCGAAGAGTGCCCTGGTTGGCGATGCCACCGACCTGTTCGACTTCATTGCGCA
GAGCGTGAAGAAGATGATGTCGGGAAAACGCCCCCGACGACCTGGAGAAGCCGTGCCGCTGGGATTCACG
TTCTCCTTCCCCTTG
>AF205930 1, 1352 N-acetylglucosamine-1-phosphate transferase
AACGCGGCCTTCGCGGTCGCTGTGCACGCGCCGGTGCTGGGGCTCATTCTGCTGGGCAGCATCGTGGCAT
ATGTTGGTACCATGCGCTACATCCCAAATGTGGCGAGGACGCTCTTGGAGCGTAACATCTTCGGCATCGA
CATTAACAAGAACACGGAGGAGCAGCGCCAGAAGTTTGCTGTGAAGCGCCGGGCCGGTCAGACAGAGGAA
AAGGAATTCCAGAAGCAGGCGATCCCAGAGTCTCTCGGCATCCTCGTAGGCGCCGTGTACCTTTCTGTGG
TGATGGTGCTCACCGTGTGTCTCCGGTTTCTCGGCACCGCTGGTGAAGGGTTAGACAACCCTTACGCATC
ACTTCCGGGTCCCTTGATGACCATCACCCTCATGCTTCTCTTGGGGTTCACGGATGACGTGCTGGATGTG
AAATGGCGCCACAAGATCATCCTCACAACGCTCGGCTCGCTGCCTCTCATCATGACGTACGACGGAAGTC
TATCCGTGCTGATGCCGTGCGTGTTCGGTCGCTTCGGTCTGCCCACCATGAACGTAACGAAGACGTGGCT
TCTTGGCCTCGCTGCCGCACAAGGCGAACCGACAACCACCTTTCGCGTCACGGCTCCCTCGACATGGTTC
TCCTACGTTGTCAGTCACCGTTCCTACGTCAAGGTCTCCGAAAGTGGCACGGTGTTGATCTACCTCGGTC
CCATCTACCTCGTTTACTTGTCCATGCTGTGCATCTTCTGCACCAACAGCATCAACATTCTCGCCGGCGT
CAATGGTGTGGAGGTGGGGCAGAGCATTGTGATCGCAGTCGCGTCTGTTGTGTACAACCTATTCCAGATG
CGCCTCGAAAGACAGGCGACACCGGCCTTGAGCAGCGTCGACGCTGTGGCGGCAGATGCACGCGACATGC
GGAGCGATCATCGACTGCGCGCGCTGCTCTTGCTGGGGCCTTTCATCGGTGTGAGCCTGGCCCTCTGGCG
CTACAACCGCTACCCCGCCCGCGTCTTCGTGGGAGACAGCTATACCTACTTTGCCGGCACCGTGCTGGCG
GTGTCCAGCATCACCGGCGTGTACAGCAAGACACTGCTGCTCTTTTTTGCGCCGCAGGTGTTCAATTTTA
TCATATCACTGCCGCAGCTCTTCAACATTGTGCCGTGCCCGCGCCACCGCGTGCCAACGTGGAACCCGCG
GACGAACTTGTTGTCAAACAGCCACAACTATACCATCCTCAACGTTGTCCTCTACCTCTTCGGCGATATG
CACGAGGAGAAGCTGACGTGGGCGATCCTCAAGTGTCAGGTCATCGCCTGTGTTTTGGGGTTCGTCGTGC
GGTACGTGTTGAGCTCCTTTCT
>AF208490 1, 1243 selenophosphate synthetase
TTGCTGTGCGAAGCGAGGAGAAGCCGCAAGCACACCCACCCCCTTCATGTCGCACAAGCGACCGCAGTCC
TCCGCAGGAGAGTCGAACGGGGCGGTCGATCTCAAAACGCCGCGTTTCGACCCCGTCTCACTGGGCCTCC
CTGCTGAGTTCCAGCTCACCGACTACACACGCCTGAAGGGGTGCAGCTGTAAACTGCCACAGCCCAAGCT
ACTAGCCCTGCTGCAGGAACTCTCGGCAACGCCTGGGCAGAAGGACGTCGGCATGGACTGCAGCATTGTG
CCGCTGCACCACACCAACAGCAAAGGTGAGGCTCTGTTCCTCGTCTCCACGACGGACTTCTTCTTCCCCA
GTGTGTCGGACCCGTTCCTGCAGGGTCAAATCGGGGCTGCAAACGTCTTGTCCGACCTGTATAGCATGGG
CATCCCGGATTGTGATACAATGCTGATGTTGCTCGCGGCCAGCACCGAGATGGACGAGCATGAACGCCTG
ATTACGACGCGCGAAATCATGAAAGGCTTTGCAGAGCGGGCGCGGTTGGCGACGACAACGGTGACAGGAG
GGCAGACGGTCATGAACCCGTGGCCGCTGATTGGCGGGGTGGCCATGGCCGTCGTGTCCGAGGCGGAAAT
GGTGCGGCCAACAGGGCTCTTGTGCGCCGGGGACATATTGGTGCTGACGAAGCCGCTGGGGTGCCAGGTT
GCCGTGAATCTCAAGCAGTGGCTGCTGCGGCCCTCGCCGCTGTACGAGGAGGCCATTGCAGGGCACATCT
CCCCCGAAGAGATCGAGGAACTGTACAATATGGCCACGGATAGCATGCGACGTCTGAACCGCGAAGGGGC
TCGGCTGATGCGGAAGCACGGCGCGCACGGGGCCACCGACGTGACCGGCTTTGGCATTCTGGGGCACGCG

AACAACTTTGGGGCAGCGCAAGCCGTCGGTGATGCGCCGCGCTCGCTGTGTCTCGTACTGGAGCGGCTGC
CGATGTTCAAGACCGCTGTGGCCGCCTCGAAGCAGATGAATGACAAGTACCGCCTTCTCGAAGGGTACTC
GGCCGAAACAAGCGGCGGGCTGCTGGTGGCGTTCCCGAGCACCACCGCAGCGGCGGCGTTCTGCGCGGAG
CTCACGGCGGTCGATGGCGGGTGTCCGTCGTGGATTGTCGGGCATGTGGAGGATCGCGCCACCAACGCCG
TCGATGGTGTTTACGCCCGCCTAAAGGACGGGTATGAAATTGTGGAGGTGTGA
>AF224339 344, 1318 casein kinase 1 isoform 1
ATGTCAAGCGCACTAGAGAACTGCCTGGTGCAGGGCGGCCGCTTCCGCCTTGGCCGCCGCATAGGCGGCG
GCTCCTTCGGCGAGATCTTCCTGGGAGTCAACACCCAAACCGGTGAAGTGGTGGCCATGAAGGTGGAGCG
CACAAGGACGGCCCACCCGCAGCTGTTGTCAGAATCGCGGTACTACACCCTTCTGAGCCAAGGCCGTGGG
GCGGCTTACATGCCAACCATCTTCGGATACTCCTCCGAGGGCGAGTTCAACGTGATGGTGATGGAGCTGA
TGGGCCTGTCACTGGAAGATCTGCATGAAAAGTGCGGCAACCGCTTTTCGCTCAAGACGACGCTGATGCT
GGCGGACCAGATTCTGTGGCTCATCGAGCTTGTGCACTCTCACAGCGTTCTGCACCGCGACATCAAGCCC
GACAATTTCCTCATGGGCACCGGAAAGAAGGGCCACCACGTGTACATCATCGACTTTGGGCTGGCGAAGA
AGTATCGTGACCCCCGCACGCACGCCCACATCCCATACAAGGAGGGCAAGAGCCTGACAGGCACGGCCCG
CTACTGTAGTATCAACACGCACCTCGGCGCCGAGCAGAGTCGCCGTGACGACATGGAGGGCATCGCCTAC
CTCCTCATCTACTTCCTTCGTGGATCGCTGCCATGGCAGGGCTTGAGGGCGTCAACGAAGGAACGAAAGT
ACAGCTTAATCGCGCACGCGAAGATGAGCACCTCCGTCGAGACGCTCTGCAAAGGGCTGCCCATCGAGCT
CGCATCATTCCTGAATTACTCGCGTGCGCTGCGCTTCGAGGACCGCCCTGACTACGGCTATCTCCGTTCT
ATGTTTCGCCGACTCTTCATGCGAGAGGGCTACCAGGAAGACTATGTCTATGATTGGACGGTGCGCAGCA
TGCATGAGACGCTCACAGCGCGGCAGCGCAAGAATGCAGCGAAGCGCGGAAAGAAACAGAAGTAG
>AF224340 57, 1118 casein kinase 1 isoform 2
ATGAACGTGGAACTGCGCGTCGGCAACCGCTACCGGATCGGTCAGAAGATCGGCTCCGGCTCCTTCGGTG
AGATCTTCCGCGGCACGAACATTCAGACGGGAGACCCTGTCGCCATCAAGCTCGAGCAGGTGAAGACGCG
CCACCCGCAGCTCACGTATGAGTCGCGCTTCTATCGCATCCTCGGCAGCGGCGGCGGCGCCGTCGGCATC
CCCATGATGTTCTATCACGGCGTCGAGGGCGAGTTCAACGTGATGGTGATTGAGCTGCTCGGCCCCTCGC
TGGAAGACCTCTTTTCCTTTTGCGGTCGCCGCCTCTCGCTGAAGACGACGCTGATGCTGGCGGACCAGAT
GATCAGCCGCATCGAGTTTGTGCATAGCAAGAGCGTTCTGCACCGCGACATCAAGCCCGACAATTTCCTC
ATGGGCACCGGAAAGAAGGGCCACCACGTGTACATCATCGACTTTGGGCTGGCGAAGAAGTATCGTGACC
CCCGCACGCACGCCCACATCCCATACAAGGAGGGCAAGAGCCTGACAGGCACGGCCCGCTACTGTAGTAT
CAACACGCACATGGGCGTGGAGCAGGGCCGCCGTGACGACATGGAGGGCATCGGCTACATCCTCATGTAC
TTCCTTCGTGGATCGCTGCCATGGCAGGGCTTGAAGGCGCACACGAAGCAGGAGAAGTACAACCGCATTT
CCGAGCGAAAGCAGACGACACCGGTGGAGCTGCTGTGCAAGGGCTTCCCCAGCGAGTTCGCCGCCTACAT
GAACTACGTGCGTGCGCTGCGCTTCGAGGACAAGCCCGACTACTCGTACCTGAAGCGCATGTTCCGCGAC
CTATTTGTGCGCGAGGGATACCACGTCGACTACGTCTTCGACTGGACGCTGAAGCGCATTCATGAGAGTC
TGCAGGAGCAGCAGTCGTTCCCGGGCGGCAGCAACGGTGGCGGTGCTGCGGGCAATGGCTCCCCGGTGAA
CCAAAGCCCGGCACAGGGCGGCAACGGTGGTGCCCCAAACAGCGCTAACAACCAGGAATCGGGTGCGCCA
GAGCAGCAGTAG
>AF229369 1, 328 20S proteasome alpha 5 subunit
TGCTGTGGAGGCCATCAAACTGGGTAGTACCAGCCTCGGAATCCGCACGCCTAAGGGCGTCGTTCTTGCC
GCGGAGAAACGCGTGCCATCGACCTTGGTCGTTCCGTCCAGCATGAGTAAGATTATGGAGATCGACAGCC
ACATTGCGGCTGTCATGAGCGGCATGGTTGCAGATGCGCGCATCCTTGTCGAGCACGCCCGCGTGGAGTC
GCAGAACCACCGCTTCACCTACAACGAGCCCATGTCTGTGGAGTCCTGCACGCTAGCGACGTGCGACCTG
TCGATCCAGTTTGGTGAGAGCGGTGGAAAGCGCAAGCTCATGTCGCGC
>AF234766 2222, 3526 LPG1
ATGGCGCCTCGTCGCTGGCATCACAACTGCCGGCGCATGGCGTCATTTGTGCGTATTGGTCTTTACACAC

TTCTCTTCCTCATGGGCTACATTGTCCCGCTTATCATTTTCTATAATCGCTCGGGCACAGAGACCTTCGA
AGATACCCCTCGTCCAGGTGAGAGGTTTATCAGTGACGAGAATTTTTTCCACTGCATCGCGGAGCGTCTC
TCATATAAGGACCAACACCCAGCACGAATCCCGTACGTGCTAATACCGGTGACGATGGATTACCAGGACA
TCAAGCAACTCTTCTGCAATATTACAGTCCCCATGACGTACATCATGTTTATTAACAATGGCATGTTCCG
CCCACTGCGCTCGCTTCTCGACCGTCTCGCCGTGGAGCTGAGGGACTACGTCGACCAGAATCTCTTTATC
ATTCACCACCCAGAGAACATCGGCTACGCGTCTGCCGTGAACGAGGGTTTGCGGCACGCTCTCAACTTCT
CCGTTGCCCAGGTGCCGTGGATCTTCATCACCAATGCCGACGTGCGCTTTGCGCCGGGGCTCATGGACGA
GTTTGTCTCGCAGGCACACATAAGGACACAAGGCCAGCTCGAGCGCATTCGCCGCCTCGACGAAGAGATC
GTTGCCGAAATCAAGACGCTGAAAAACGTGCCAGACCCGCGGTTTGCTTTCCGCAGCAGCCGGCACCCCA
TCGTCACGGCATCCTCGCTGCCGTATCGTATTCGGACCATGCCCCCAGAGCAGATGAAGAAGCAGTTCGC
CGACGCATACGGCATCTTTTTCACAGACCACAAGGAGTTCATGGCTACATTTGCGCTGTCCCGCCTCGCC
ATTGCGACCTTAGGGTTCTTCGACGAGAACTTCTACCCCGCCTACGGCGAAGATCACGACTACATGTGGC
GCATGAATGCTCTGGGCTACCGAAAGTACTTCTCCGAGCGGGGAAAGTATGTGCACTTTCAAAACGCGAA
CCTCAACGTCGGTGGGTCGGCCAAGAACCGGGGCGTCTTCAAGGACACTGCCTACTTCGTCCAGAACGTC
AAGTTTTGGCGTATGAACAATGAGCTGTTTCGCCTGCAATATCGCCATGCGAAGTGGTTCCCTGATGATG
TCACCATCTATCGAGGTGTCAGACGCCGGCCACTACCGTTTAACGGAACCATTCCTGTCGACATGTGGGT
ACTCGACGCTGAAAGGCAAAGGTCCATCTGGCAAATCGGCGAAAGCATGCGCTGTCACCGCGACTACAAG
CCATACAGCACAAAGCTACTGGACTTTGCTGTTGATCCTAGCTAA
>AF242436 1, 1440 gnd
ATGTCGAACGACCTCGGCATTATCGGTCTCGGCGTCATGGGCGCGAATCTCGCCCTGAACATCGCCGAGA
AGGGATTTAAAGTTGCCGTCTTCAACCGCACCTACGCGAAGACGACGTCGTTTCTCAAGGAGCATGAGAG
CGAGAAATTTGCCGCCAACCTGAATGGATACGGGACCATGAAGGAGTTCGCTGCGTCCCTCAAGAAGCCG
CGCCGCGCGTTCATTCTCGTCCAGGCCGGCGCCGCTACTGACTCTACAATCGAGCAGCTCAAGGAAGTGT
TCGAGAACGGCGACATCATAATCGACACTGGTAATGCGAACTTCAAGGACCAGGACAAGCGCGCGGCTCA
GTTGGAGAGCCAGGGTCTCCGCTTCCTCGGCATGGGCATCTCCGGTGGTGAGGAGGGTGCGCGCAAGGGG
CCGGCCTTCTTCCCGGGTGGCACACCAAGCGTGTGGGAGGAGGTGCGCCCGATCGTGGAGGCGGCTGCGG
CCAAGGCTGAGGACGGACGCCCGTGCGTGACTTTCAACGGCAAGGGCGGCGCTGGCTCCTGCGTGAAGAT
GTATCACAACGCTGGGGAGTACGCCGTGCTGCAGATCTGGGGTGAGGCGTACAGCGCCCTGCTTGCCTTC
GGCTTCGACAACGATCAGATCGCCGACGTGTTCGAGTCGTGGAAGGCGGATGGCTTCCTTAAATCCTACA
TGCTCGACATCTCCGTTGCCGCTTGCCGAGCGAGGGAGGCAACAGGCAACTATTTGTCAGAGAAGGTCAA
GGACCGCATCGGCTCCAAGGGCACCGGCCTCTGGTCTGCCCAGGAGGCTCTGGAGATTGGTGTGCCAGCG
CCCTCGCTCAACATGGCCGTCATCTCACGCCAGATGACCATGTACAAAGGGGAGCGCATCGCCAACTGCA
AGGCGTTTCCTAACTTCCCTCGTGGCCCTTCTGAGGAAGCCACGGACAAGTCCCCGAACTCCCCCGAAGC
GAAGAAGCTGTACCACGCCGTCAGCCTTTGTATCATCGCGAGCTACGCGCAGATGTTCCAGTGCTTGCGC
GAGCTGGACAAGGTGTATGGATTCGGCCTGAACCTGCCCGCCACGATCGCCACCTTCCGCGCCGGCTGCA
TTCTGCAGGGCTACCTGCTAGGGCCTATGACCAAGGCCTTCGAGGAGAACCCGAACCTGCCCAACCTGAT
GGACGCCTTCACCAAGGAGATAGCGGCGGGCCTTGATGACTGCCGTCAGATTCTCGCCAAGCTCACAGAG
AACACGGCAGTGTCACTGCCGGTCATGATGGCCTCACTGTCCTACATCAACGCCATGTACACGGAGACTC
TTCCGTACGGACAGCTGGTGTCGTTGCAGCGCGACGTCTTTGGCCGCCACGGCTACGAGCGCACGGACAA
GGACGGTCGTGAGTCGTTCGAATGGCCCGCACTGCAGTAG
>AF244919 493, 2388 BT1
ATGCCAGGTCGTTACCGCGTGCCGTCCGCGCCGGAATCGCCGCCGCCGCAGGGGGCCAAGTACGTTCATC
CTGTTTCCGCTCACGTGCTGCGCGCTGCGCCGTTCCTTGGCTACATCCCGGTCTTCAGCATTGTGATCAG
GTCATTCCACCCCAAAGTTGTGCTCGCCATTTGCATACAGCGCTTATTCGAGAAGGGCCTCGCAGATGGG
CTGATGCGACTGTCGATCCAACCGATGCTGACGGGACGCTACGGACTCACCGGCGCCATGTACCAGCGCC

TGTCCACCCTCTACACCCTGGGCTGGGCCCTCAACGCATTCATCACAGTGATGGCGGACACGTTTGCGCT
GTTCGGGTACACGAAACGGTGGTACTGCGTGATGTCGGCGGTGGGCGGCAGTGTGTTCGCGCTGCTGTAC
GGGCTGCTGCCGGCGAAGGAGTCGAGCGCGAAGCCTGCTGCGGCCCTCATGTTCCTGACGGCACTGTTCA
TGAGCAATATCGACGTCTTCGCTGTTGCCTTGTACAGCGAGCAGATCCGCCGGCGTCCAGCTGCCGGGCC
AGCGCTGGTGAGCTGGATGTGGGGCACGGCGCTCATCGGTATTATGATTTCCAGCGTCATTCTGGGTCCT
CTCTCTGACAATGGACTGGCGCACTTTGGTGTGTACATCACAGCCGCCATCCTGCTGCTGTCGGGTCTAC
TGTTCGTGTTCAACCTCTTCGAGGAGCGCCGCAACCGCGCGGCACGCCTGGAGGACGCCATGATCGAGTT
CCTTCAGAAAACCAAGAGCGCAAGCAGCGAGAGCACAGCCGGCTCCCCGCCGTCGCCACATAAGCTGGAT
GGGCATCTCACCATTGATAACCGCGTGGAGGAGGAGGACGACAACGTCGACGAGCAGGAGGCAGCCGCGA
TGGATGCGCAGGGCTTTGTGCGGCCGCGCATGGACACGTACCTATGCGGTGCCGTGGAGATGAACCGGGA
CGTCATTCTGCGAAACTGGCGGATGGCGTTATTCTGCCTGATTCTCACGCTCGGCGTGATCGCTAATTCA
CTCGTGAGCATTCTTGGCACGTGGTGGGACATCATGTATGTCTGCATCGTCTTGGCGGCGGTGTTGTGCG
TCAGCTCCTTCTTCACGTTGCCGCTGGCCATTGCCAAGGCGGTTGTGTTCATGTATTTCAACGCAATCCT
CTACCTGAGCCTGCCCGGCGTGCTGAATACTTTCTACGTCGCGAAGCCGTCATGCCTCCCCGATGGACCG
CACTTCTCGTACACGTTCTACAACGCCATGAATGGCTTTCTGGGCAACATCGCGGGCATCGGTGGTACCA
TCGTGTTCACGCATCTGTTCCCTCACCACAGCTATCGGTTTGTCATGGGCCTCTCCGCTGTTCTGCTGCC
GGCAGCCTCTATGTTTGACGTCCTCATCTTGAAGCGCTGGAACCTGGCCATCGGCATCCCGGACCACGCC
ATGTACATTTTTGGGGACACCATCATCTACGAAGTGTGCAATATGCTCCTGAACATGCCCATGATGATGC
TCATGTGCCGTATCGCGCCGCGCGGGTCGGAGTCGATGGTGTTTGCGCTACTCGCGAGCATTTACCACCT
CGGCACCTCGACCTCGTCAGCGATAGGCTACCTGCTGATGGAAACGATTTGGCCGGTTGTCACGCAGGGC
CAGTGTGACTACAGCAACGCGCTGTGGTTGGTGATCACTGGGCACATTGTTACACCCGTTTTCATCTTAC
CATTGGCTTACATCCTCCTCCCATCAGCGCGCATCAGTGACCACATTGACCACACGGGTCGGAAGGTGAT
GGAGGTCGCACTCCCGGAGACGCGCACTGAGGAGGCAGAGGAGCCGATCGCTATGACGACGAGGCGGGAC
AGCTAG
>AF305956 1, 1266 NMT
ATGTCTCGCAATCCATCGAACTCCGACGCTGCGCATGCGTTCTGGAGCACACAGCCCGTACCGCAGACGG
AAGATGAGACGGAGAAAATTGTGTTCGCTGGTCCGATGGACGAGCCAAAGACGGTAGCCGATATTCCTGA
GGAGCCGTACCCGATCGCCAGCACATTTGAGTGGTGGACGCCGAACATGGAGGCGGCCGACGACATTCAC
GCAATTTACGAGCTTCTTCGGGATAACTACGTCGAAGACGACGACAGCATGTTTCGTTTCAACTACTCCG
AGGAGTTTCTTCAGTGGGCACTATGCCCACCGAACTACATCCCGGACTGGCACGTTGCAGTTCGCCGAAA
GGCGGATAAGAAGCTGCTGGCCTTCATTGCCGGCGTTCCCGTGACGTTGCGCATGGGCACTCCCAAGTAC
ATGAAGGTGAAAGCACAGGAAAAGGGCGAAGGGGAGGAGGCGGCCAAGTATGATGAACCCCGTCACATCT
GCGAAATCAACTTTCTCTGTGTCCACAAGCAACTCCGGGAGAAGCGGCTTGCCCCGATTTTGATCAAAGA
GGCGACGCGCCGCGTGAACCGCACCAACGTGTGGCAGGCGGTGTACACGGCTGGGGTGCTGCTACCCACT
CCGTATGCATCAGGGCAGTACTTCCACCGCAGCCTGAACCCCGAGAAGCTTGTGGAGATCCGCTTCAGCG
GCATTCCAGCACAGTACCAAAAGTTTCAGAACCCAATGGCGATGCTGAAGCGCAACTACCAGCTGCCAAG
CGCGCCGAAGAACTCTGGTCTTCGTGAAATGAAGCCGTCTGACGTTCCGCAGGTGCGGCGGATTCTCATG
AACTACCTGGACAGCTTCGATGTAGGTCCCGTCTTTAGCGATGCCGAGATCAGCCACTACCTCCTTCCAC
GCGACGGTGTGGTCTTCACCTACGTGGTGGAAAACGTCAAGAAGGTGACCGACTTCTTTTCCTTCTATCG
AATTCCGTCGACTGTGATTGGGAACAGCAATTACAACCTTCTGAACGCAGCTTACGTTCACTACTATGCC
GCGACGAGTATACCTTTGCATCAACTCATTCTCGACCTTTTGATCGTGGCGCATTCACGCGGCTTCGACG
TGTGCAATATGGTAGAGATCCTCGACAACCGGTCTTTCGTTGAGCAGCTCAAGTTTGGCCCCGGCGACGG
TCATCTTCGATATTACTTCTACAACTGGGCGTATCCAAAGATCAAGCCTTCTCAGGTTGCCTTGGTGATG
CTGTAG
>AF312583 1, 627 FeSODB2

ATGCCGTTCGCTGTTCAGCCGCTGCCGTACCCCCACGACGCGCTCGCGTCGAAGGGCATGTCGAAGGAGC
AGGTCACCTTCCACCACGAGAAGCATCATAAGGGGTACGCCGTGAAGCTGACCGCTGCCGCGGAGTCGAA
CTCGGCTCTTGCGTCGAAGTCGCTGGTGGACATCATCAAGTCTGAGAAGGGCCCCGCCTTCAACTGTGCG
GCGCAGATTTACAACCACGACTTCTTCTGGCGCTGCCTCTCGCCACGCGGCGGGTCAAAACCGCACGGAG
AGATCGCGAGCGCGATCATCGACAGTTTCGGCAGCTTCTCGAATTTCAAGAAGGAATTCACGGATGCGGC
CAACGGCCACTTTGGCTCGGGCTGGGCGTGGCTCGTGAAGGACAAGTCGAGTGGCAAGCTGAAGGTGTTC
CAAACGCACGACGCGGGCTGCCCGCTCACGGAGCCCGACCTCGTACCGATCTTGGCGTGTGATGTATGGG
AGCACGCGTACTATATCGACTACAGGAACGACCGCGCGTCCTACGTGAGCGCGTTTTGGAACATGGTGAA
TTGGTCGCACGCCAACCACTGCTACAGGGCCGCTGGTGGCTCCCACTACGTCAACAGTGATCTGTAA
>AF312586 1, 588 FeSODB1
ATGCCGTTCGCTGTTCAGCCGCTGCCGTACCCCCACGACGCGCTCGCGTCGAAGGGCATGTCGAAGGAGC
AGGTCACCTTCCACCACGAGAAGCATCATAAGGGGTACGCCGTGAAGCTGACCGCTGCCGCGGAGTCGAA
CTCGGCTCTTGCGTCGAAGTCGCTGGTGGACATCATCAAGTCTGAGAAGGGCCCCGCCTTCAACTGTGCG
GCGCAGATTTACAACCACGACTTCTTCTGGCGCTGCCTCTCGCCATGCGGCGGTGGCGAGCCATCGGGCA
ATTTGGCCAGTGCGATCATCGACAGTTTCGGCAGCTTCTCGAATTTCAAGGAGGAATTCACGGCTGCGGC
CAACGGCCACTTTGGCTCGGGCTGGGCGTGGCTCGTGAAGGACAAGTCGAGTGGCAAGCTGAAGGTGTTC
CAAACGCACGACGCGGGCTGCCCGCTCACGGAGCCCGACCTCGTACCGATCTTGACGTGTGATGTATGGG
AGCACGCGTACTATATCGACTACAAGAACGACCGCGCGTCCTACGTGAGCGCGTTTTGGAACATGGTGGA
CTGGGATTTCGCCTCTAGCCAGCTTTAA
>AF312588 1, 693 FeSODA
ATGTTCCGCCGCGTCTCGATGAAAGCCGCCACGGCCACCGCTCCCGTCGGGTTTTCGTTCCTGTGCTACC
ATACCCTTCCTCATCTTCGGTACCCGGCCGAGCTGCCGACGCTCGGCTTCAACTACAAGGACGGCATCCA
GCCCGTCATGAGCTCCCGTCAGTTGGAGCTGCACTACAAGAAGCACCACAGCGCGTACGTGGACAAGTTG
AACACGCTCGGCAAGGGCTGCGAGGGGAAGACGATTGAGGAGATCATCTTGGCGACCAGCGGCAGCACTG
AGAGCAAGGTCATGTTCAACCAGGCCGCTCAGCACTTCAACCATTCCTTCTTCTGGAAGTGCCTGTCGCC
TGGTGGCAAGCCGATGCCGAAGACGCTCGAGAATGCCATCGCGAAGCAGTTCGGAAGCGTCGACGACTTC
ACGGTTTCCTTCCAGCAGGCCGGCGTGAACAACTTTGGCTCTGGCTGGACGTGGCTCTGCGTCGATCCCC
GGACGAAGGAGCTTCGCATCGACAACACGAGCAACGCGGGCTGCCCGCTGACCTCTGGCTTGCGCCCCAT
CTTCACCGCTGATGTGTGGGAGCACGCCTACTACAAAGACTTTGAGAACCGCCGCGCGGACTACCTGAAA
GAGCTCTGGCAGATCGTCGACTGGGAGTTTGTCTGCCAAATGTATGAAAAGGCCACAAAGTAA
>AF336276 1, 336 H2B
ATGGCCTCTTCTCGCTCTGCTTCCCGCAAGGCTTCCAACCCGCACAAGTCGCACCGCAAGCCGAAGCGCT
CGTGGAACGTGTACGTGGGCCGCTCGCTGAAGGCGATCAACGCCCAGATGTCGATGTCGCACCGCACGAT
GAAGATCGTGAACTCGTACGTGAACGACGTGATGGAGCGCATCTGCACCGAGGCCGCGTCGATTGTTCGC
GCGAACAAGAAGCGCACGTTGGGTGCGCGCGAGGTGCAGACGGCGGTGCGCATTGTGCTGCCGGCGGAGC
TCGCGAAGCACGCCATGGCTGAGGGCACGAAGGCCGTGTCGAGCGCGTCGGCTTGA
>AF350492 782, 1807 lipophosphoglycan biosynthetic protein
ATGAACCACACTCGCGCTGTCATGGAGGCGATCCTCGCCGTCATCACGTATAGCTTCTGCAGCGTGAGCA
TGATTCTCGTGAACAAGCTAATCATGAACACGTACGACATGAACTTCCCATTCGGAATCCTGGTGCTGCA
GACTGGTGGTGCACTAGTGATTGTGGCGCTGGCGAAGGCAGCACGCTTCGTTGAGTACCCGGCCTTTTCA
TTCGACGTGGCGAAAAAGTGGCTTCCTCTTACTCTGCTCTTCGTGGCAATGCTATTCACATCCATGAAGA
GCTTGGCCACGATGTCCGTCGCGGCTCAGACGATTCTCAAGAATCTGGCCGTTATCTTGATTGCACTCGG
CGACAAATTCCTCTACGGCAAGGCGCAAACCCCGATGGTCTACTTTTCGTTTGCGTTGATGATCCTCGGC
AGCTTTCTTGGCGCCAAGGGCGATAAGTGGGTAACAGCGTGGGGTCTCATCTGGACGCTTCTTAACATTG
TCTCCACCGTCTCGTACACGCTGTACATGAAGGCTGTACTGGGCTCTGTCAGTAACTCGATCGGCCGTTA

CGGTCCTGTCTTCTACAACAACCTGCTGTCCCTACCCTTTTTCCTAATAATGGGTGTCGGGGAGATCATG
CCGTTCTCGGCCGCAATCGGTGAGACAACCACGCTCGGTAAGCTGGTGCTGACGTTCTCGGTGCTGGTGA
GCTCCGTGATGACGTTCTCTGTCTTTTGGTGCATGTCTATAACCTCGCCCACCACAATGAGCGTCGTGGG
CTCACTGAACAAGATACCCCTCACTTTCCTGGGCATGCTGGCGTTCCACCAGTTCCCCACCGCAACGGGT
TATCTGGGTATCATGGTCGCGCTATCCGCAGGATTCCTATACACGCACCTCAACATCAGGGCGAACCGCG
CCAAGGCTTCTTCCGATTTGGAGCACCAGATGCAGCAGACCGGCAAGACGACAACGGAGTCGATCGTACT
GGTGCGCACGGACGAGAACAGCAGTGACAACTTCAAATCTGAGTAG
>AF363975 1, 939 p36 LACK protein
ATGAACTACGAGGGTCACCTGAAGGGCCACCGCGGATGGGTCACCTCCCTGGCCTGCCCGCAGCAGGCGG
GGTCGTACATCAAGGTGGTGTTGACGTCGCGCGACGGCACAGCCATCTCGTGGAAGGCCAACCCCGACCG
CCACAGCGTGGACAGCGACTACGGTCTGCCGAGCCACCGCCTGGAGGGCCACACCGGCTTCGTGTCGTGC
GTGTCGCTGGCCCACGCCACCGACTACGCGCTGACCGCGTCCTGGGACCGCTCCATCCGCATGTGGGACC
TGCGCAATGGTCAGTGCCAGCGCAAGTTCCTGAAGCACACCAAGGACGTGCTCGCCGTCGCCTTCTCGCC
GGACGACCGCCTGATCGTGTCCGCGGGCCGCGACAACGTGATCCGCGTGTGGAACGTGGCCGGCGAGTGC
ATGCACGAGTTCCTGCGCGACAGCCACGAGGACTGGGTGAGCAGCATCTGCTTCTCGCCGTCGCTGGAGC
ATCCGATCGTGGTGTCCGGCAGCTGGGACAACACCATCAAGGTATGGAATGTGAACGGGGGCAAGTGTGA
GCGCACGCTCAAGGGCCACAGCAACTACGTGTCCACGGTGACCGTGTCGCCAGACGGGTCGCTGTGCGCG
TCCGGCGGCAAGGACGGCGCGGCGCTGCTGTGGGACCTGAGCACCGGCGAGCAGCTGTTCAAGATCAACG
TGGAGTCGCCCATCAACCAGATCGCCTTCTCGCCCAACCGCTTCTGGATGTGCGTCGCGACGGAGAGGTC
TCTGTCCGTGTACGACCTCGAGAGCAAGGCCGTGATTGCGGAGCTGACGCCGGACGGCGCGAAGCCGTCC
GAGTGCATCTCCATCGCCTGGTCCGCCGACGGCAACACCCTGTACTCCGGCCACAAGGACAACCTGATCC
GCGTGTGGTCCATCTCCGACGCCGAGTAA
>AF406767 1235, 1684 centrin
ATGGCTGCGCTGACGGATGAACAGATTCGCGAGGCCTTCAACCTCTTCGACGCCGACGGCTCTGGCGCTA
TCGACGCGGAGGAGATGGCGCTAGCGATGAAGGGTCTCGGGTTCGGTGACCTGTCGCGCGACGAGGTGGA
GCGCATTATCCGCTCTATGCACACAGACTCGAACGGTCTGGTGGCGTACGGCGAGTTTGAGGCCATGGTC
AAGTCGCGCATGGCGCAGAAGGACTCGCCGGAGGAGATCCTAAAGGCCTTTCAGCTCTTCGACCTCGATA
AGAAAGGCAAAATCTCCTTTGCGAACTTGAAGGAGGTTGCGAAACTGCTGGGTGAGAACCCCGGCGACGA
TGTGCTGAAGGAGATGATCGCCGAGGCCGATGAGGACGGTGATGGCGAGGTGTCCTTCGAGGAGTTCAAG
AGCGTGATGCTGCACATGCGTGGAAAGTAG
>AF424693 1, 1698 lap
ATGCTTCGTCGCGTGCTGTCTCGTGGCCCACTCCCACAGTCGCTGTCGGTGGGCCCCACGTCGAAGCTGA
CGGTGAACAAGGGCAAGCTCGCCAAGAGCCGTGGAGTTCATGTGCACTTTCTCACCATCGCCGAGTGTGC
AGCGGCGGCCGGTGATGCTAGCGCCCCTATTCCACCAGGCTTTACCGGCAAGGCAGGGGAGGTCGACTTC
CAGACCCTCTGGACGACCGCGCAGGATCCGGCCACAGCGGAGAGCGTTCTGAGAAAGAGTGGCCGAAAGG
CAGCCGGGGCCAGGCGCGCTGCGGAAAAGCCTGCCGAGCTCGTGGCTGGCATGGGGGCCCACTCCACGGT
GCGGGATTACCGCCTCGCGGTCACGGCCGCCGTGCGCAAAGCCAAGATGTGCAACGCCGCGTGGGTGTCA
CTGCACCCGCCGAAGCAAGATGTCTTTACCATGACAGACCCGTACCACCCGCCACAGCGGCTGAAGAGCC
ACGAGATGGTGGAAAAAACCGCCATCTTCGCGGTGACGGGGGCGTACCAGTACGACCGCCTGAAATCGGG
CACCCTCGCCTCCAAGGATTCTGCACAGACGGGCAGAACCCGCGCGAGGCGGGCGGAATCGCGGGCTAAG
CCACCCGCTGCTCCACAGCCGCCGATGGAGCTTGTCGTGGCGTCAAGCAATGCGCAGGCGGTGCGCGCGG
GTGAGGTGATCGGGCACTGCGTCAACGAAGCGCGGAACCTCGGCAACCTGCGCGAGGACGAAGGTGTGCC
GGAGTTCTATGCGGAGTGGGCGAAGAAATACATGATACCGGAGGGCATCAAGGTGCGCAAGGTGCTGCGC
GGACAGCAGCTCGAGGAGGCGGGCTTGCACCTCATGTACAACGTCGGCCGCGGCTCCCGCTATGAGCCGT
ACTTGGTGGTGCTGGAGTACATCGGCAATCCCCGCTCGAGCGCGACGACGGCCATCGTGGGCAAGGGCGT

GACCTTCGACTGCGGCGGCCTGAACATCAAGCCCTATACCTCGATGGAGACGATGCACAGCGACATGATG
GGGGCGGCCACCGCGTTCTGCACTCTCAAGGCGATCGCGAAACTGCAGCTGCCCATCAACGTTGTGGCCG
CGGTCGGACTCGTGGAGAACGCCATCGGCCCCGAAAGCTACCACCCCTCCTCCATCATTACTAGCCTCAA
GGGGCTCACGGTGGAGGTGCTCAACACTGATGCTGAGGGCCGCCTGGTACTGGCAGATGTGCTCACATAC
GTGCAGAAGTACGCACCACTCGATAAGAAGCCTACCCGCATCATCGACCTTGCCACGCTCACAGGTGCCA
TTATTGTCGGTCTCGGCTCGCGCCGCGCCGGGTTGTTTTCGCCATCTGCGATGCTGGCGAATTCGCTAAT
GGCTGCCGGCACTCAGTGCGGGGAGGAGCTGTGGCCGATGCCGATCGGTGACGAGCACAAGGACATGATG
AAGGGCGGCATTGCCGATCTGATTAACGCTGCTCCCGGTCGGCAGGCCGGCTCCTGCACCGCCGCCGCGT
TCCTCTCCAACTTTGTAGAGCCGGAGGTGCAGTGGGCGCACCTCGATATCGCCGGCGTTGCAGATGTCGG
AGACAAGCCCAAGGGCTACGAGCCGGCTGGTGTGACGGGCTTTGGTGTGCAGCTTCTGGTCGACTTCCTG
CGCCACAACAAGCCTTAA
>AF441859 1, 1425 RNHII
ATGCTCCACGCATGGCGCTTCGTTCCTCTTGCACGTAGTACGTCCGCCCTTGGCAGTGGGCTGCTCCGGC
CCAGTCGCGTTCTCCGGCAGAAGCGACTCGCCTATTCCCCTTTCAGGCCACCGCCGACATGGAACGCGCT
GCAGAAGGTGGAGGTGGGACATCGCCAGACGGACACGGGCGAGCCTACACTCGATGGCACGCTGGACCTG
GTCGGGTACCAGCTCTCTCGCGACGGTCTGCTGCCTCAGACGAACATCTTAGGCCGCACGCTGCCGGTTC
CCGATACCAGTGGCATCTCTGCGGCGACTCGTCGTAAGCTGGAGCGAGAGAGAGATGTGGCGGTGGCGCA
CATGAAGAGGCACGGCGTGCGGCTGAGGCGCAGCATCAAGGAAACAGCCGTGACGATCGGATGCGATGAG
GCGGGCAGAGGCCCACTTGCCGGCCCTGTCGTCGGCGCCGCCGTCTCGCGCATTCCCGTCTCCAGCTTCA
ACAACGAATTTGACCAGCTGTACGAGGCACCCGAGCAGTTTCAGATCTTCGACAGTAAGTCCGTTTCGGA
GCGGCAGCGCGACCTCGTGTTTGCCATGATCACCGGACACGTCGATTTCTTCGACATTGCCAGTTGCAAG
AAGTTTGTCGTGCACCACTGCGCCGGTGATGAGGCGACATCGTTGACGCTGAGCAGTAAGAAGCTCGACT
CCCACGTGAAGCTATCGAAGCTGCCCTTCAAGAAGCTGCTCTCCATGCAAACACCGTATCTGATCACCTA
TCACGGGTACAACAGCGCAGGCAACTACGTGTACTTCTGGTCCATCGGCATTGCCAACCACACCTACATT
GATGAGTACAACATCTACAACGCCTCCATGAACACGATGCACCGCTCCGCTCAGTCGATTTGGCACATGC
TGAGCGACGCGCGCTTCTCGCACGAGGCGGCGCCGCGGCCGCGCTCATCCAGCATCGCGCAGTACCTGTT
CAGCCGCTTCTGCACCGCCGCCAACCACGACAACGAGAAGCGCTATCAGGTGCCGCATCACCTGGAGCTC
CTGAAAGGGGCCACCGAGTACTTCGACTTTGAACCAATACAGCCTCCGCTAGTTCTCATCGACGGCCATG
CCGTGCCGGGCCCGAGCTACGACTACTTCACCAGCGTTCGTATTGGTGGCGATGTGCAGCCCATCATCGA
GGGCGACAAGCGCAGCCTCTCCATCGCCGCTGCGTCGTGTCTTGCCAAGGTCACCCGTGACGAGCTCATG
AACTACATCGACGCCCTCTACCCGGGCTACGGCTTTCGGGACAACAAAGGATATCCGGTCGAGCAGCACA
TGAAGTACGTGGCCAAGAACGGACTGTGTCCGATACACCGCAAAACATATCGCCCATGCCGCACGGTGCT
GGAGAAGGGGCTGCAGAGGAAGTGA
>AF461507 1, 2295 farnesyltransferase alpha subunit
ATGCGTCTTCGCATGGGCACCGCAGGCGCGATGCAGCGTTCGAGCAGCACATCCACCCCGTCCTCGGTAT
CGTCCTGGACGGCTGCCTCCTCCGCTTGCTCGCAGAATTCCGAGGATGGAGAGTGGCTTTACGAGCTCGC
CTGCGTCGAGACCTTCATGCCGCTTGCTGCGGACGTGACACCAGCAGACGAGAGTCCCGGTGCATCTCCA
GAGCACCCCGTCGCGAAAATTAACTACCCGCCGAACTTCAGCTGCATCTACGGCGTATACCGCTCACTTC
GCGGTCAACTGGAGTACACCCCGGCGCTCCCGGCCCGCGGCGAGCGATGTGGGGGTGGTGAGTTGCCGCC
TGTGATTCATGCCATCCGCACCTCAGCGGCACGCTGGTTTCTTCTGCTGGCGTTTGCACTTCGTCAGTGC
ACCTCCAACTACACCGTCTGGAAAGACCGACGCGATGTACTCATGTCCCCTGCGGTGTTGGAGCAAGCGA
CCCGTGACGCGCTGCCGGAACTTCCGGTACCGGCGGCCATCCTTTCCTGCACCGCCTCGGAGGCAGACAA
GCAAGAGGCGCTGCGGGAGCTGGACGAAAAGCAGACGAAGCTGAGGCTGACGTCACAGCACTGGCTCCCT
GCGCGCGCCGACATTGCATGGGAAGGTCGACTGAGCCCGTGGCGTGCGGTTCGCTGGGAGCTGACGGCGG
TCGGTTGCTTCACACGGCTGTATCATAAGAACTTTCAGGTGTGGCATCACCGAAAGGAGCTGCTGACGTA

CGCTCTCCAGCAGAGCCCCTCTCACGTCCGATGCGGTGTGGACGCCGCGGCCACGGATGAAAAGCTGGAG
CAGCAGGTGGAGGACAGCAGTCCGTTTGTGCCCCTCTTGGCGAGTGAGGCAACATTTAGCGATTACCTGC
GCCGCCATGACGGCCTCGACTTTGCCGCGGTCGACGAACGGCCGACGTTGCGCGCGGTGCTGTGCAACGA
GGATGGAAAAAACTATCATGCCTGGCTGCACCTGTCTTGGTACTTGCATGTTTTCTCCTTTCTGCTGACG
CCGCCGTCGCGGAAGGCCCTGGATGAGCACGCGACGGTGGCTACAGCTGCCGCAGCTGATCAAGGTCTGC
GCTTTACGCCGCAACCCGACTGGATCACTACCCGGGAGAACGCAGCGGTGGCGGCGCTGCGCCCCACCCT
ACCACCAAGTCCACTCACCGAGGAGATGCAGTTCACGGCGCAGCTTATCTATCAAGACTGCCGCAACAAC
TCTGCGTGGTGCCACCGCTTCGCCCTCTTTAGGGAAGCCCTGCTTCGTCCTCTCTGGCAGCAGCTCTGCT
CCGAGGCTTGCACCAGTTCGGTCGGCGCACCCACAAACAGCGAGAACTGGCCGGGTACCCTGCGAGGCAT
TTGTGCAGTGGAGCTGAATTACTCGCTGCAGTGGTTGTATGTGGACCCAACGAACGAGGCCGCCTACACC
CACGCCCGCTCCGTTGCCCTCCTCTTCCACACACTCATCACGCGACGGCACGTGTGGGCGGCAGAGCACG
CCAGCACGGACGGTAGTGAGGAGCTTCAGCGATACCTCACCAACGCCCCATTGCTCCCCCCGGTCGCACT
GGCGAACTCTGAAATGTGCGCCGACGCCGACGCCGGCACCGTCAGCCCCTCCACGCCACTTGCGCGTCTC
CTCCGTGGCCGCCACCGCTGTGTCCCGTGGGTCACCTACGTGGAGAGCTTCGCACTGCTGCGGCACATGC
AGCGGGTGCTGCACACTGTCATCCGGCTCCGGGTTACCGAGCTGGAGGCGCAGGCGGCGCGCGTCTTGCG
CTTGGCGGCGGCGAGCAACACGAGAAGCACATCGGGGCAAGCACCCAAGTCGGAGGCGATTGCCGAGCTA
AAGACTCTCTACGAGCGCAGCTCTCAGTACATGCTGGATAGCTTCCACCAGGTCGACACAGCGCAGTACT
TGGCCTGTCACGCAATCCTGGAGGAGATGTGGCTGTCGTACATGGACGCGGCACAGCGACGCAGGGTGCA
GCAGCTTCGCCCGTCGGAGGCGTACCGCGAGGGTCTCCAGCCGGAGTGGCTGAAACCCTGTCCGTGGGAT
GAGCCGAAGACGAGCACAATGGACGCCGGCAAGCAGGAAGACGGTCAGGATGCTGTCGTCCTCGACTTCC
TGGCCTACGAGGCCGCTGCGTTGAGCAAGGCAAAACAGCTGACGGTGGCGGACCCGATCCGTCTTAAGTA
TTGGAAGAACGAGGTTCTTAACGTTATCTACCGCGGCTATGGCATGTCTGTCTGA
>AF461508 1, 2178 farnesyltransferase beta subunit
ATGATGAACTTAGCACCGGACACCATCACAATAGGGGCGCAGCGCAACACGGAGCTGCTGCTGCTCGAGT
ACCTGCGCAGCACGAACCCTCACATTCACGCCCTGTGGCAGCGTCACCCGAACAAAGACAACGACGCCTC
CTCTTCATCCGCCGCGTCGTCGCCGTCGAAGGACACTCGTGGCGCCAGTGGTAGCGATGTCGGAGACGCC
AACGCCTCCTCCTCCTCAGCAGCGTCGGCATTGAGGGAGTCGTTCGACTACACGGAGCCGCGCCTCTACC
GTGCCGCACACGTCCATTTTCTCATGGAAAACCTCAGCGTGGCTCCACAGGGCTTCTCCAGTCTCTATCC
GTCGCGACCGTGGATCGTATACTGGGCCTTGCAAGCTGCCGACGTGCTGAATTCCATGGAGAGCGAAGTG
CTGCACCGTACCCCGCCGAGCGCCATCGTGGCGTTCCTGCACAGCTGCCTCAGCGTCGACCACACATGCC
AAGCGGAGGTGAACAACCGGCTCGCGGCTGCCGCCACGTCTGCCCGTGGAGCCCCGGAAGGGAAAGACCC
CCACGTCACCGGGACACCTCTGGAGAGCAGCACAACATCCGTGACACTCGAGAGGGACAGCGCTGACGCG
ACGAGTGCCACCTTGATGCCGCCAGTGGAGGACGCCGCAGAGGTGGCTGAAGGTCCCTCACGGCCGGTGA
TGGGTTTTGCAGGAGGCGCCACGCACCAAGAGCCACACATCGCCTCCTCCTACGCAGCCTGCTGCGCTCT
CGCGATGCTGAGCTGGTATGGCGACGGGGCGCCGCTGCGGCAGCTCCCGCGCGCCGCTATCAAGCGGTGG
CTCCTCACGCTGCGCAACGAGGACGGTAGCTTTCGCGTGCATGGCGGAGGCGAGTCGGATATTCGCGCCT
CGTACTGTGCCGCCGTCATAACAACTCTCCTCGGGCTCGATGATCCGACAACCTTTGACGGCGAGGCCGG
CCGCTGCGAGTTTGTGGACGACGTGCGCGACGTTCCGGTGCTAACCCTGCAGACGGCGCGCTTCGTCGCC
GCGTGTCAGACCCACGAAGGCGGCTTCACATGCTCGCCCACCGCGTCGGAGGCGCACGGGGCATACACCC
AGTGCGGGCTGGCCGCCCTACTGCTCATGAAGCAGCCGCACATGGTGCATCAGGCGTCACTGCGTCGCTG
GCTGGCCGCGCGTCAACTGAACTATGAGGGCGGCTTCAATGGTCGCACAAACAAGCTGGTCGACTCTTGC
TACTCGCATTGGATCGGCGCATCGCACGTGCTGCTGCGCACGGTCGAGGCATACACAAAATGCTTCACAG
AAGTGCCTACCACACATTCGCGCAACGGAGACGGTACAGGAGGTTGCGAAGATGGTTATAGGGAGGATGT
AGATTGCACCCCGTCCGCTCGTTGTCTTCGGGCCCGGGAGGTAGTGCTGCTGGATCATGCGCAGCTACTC
GACGCAAAAATGATTCACGCTAGCGCCGATGACGCCTGGGACCGTGCCGAGAGTGCACATCTTACGCGAC

TGGGTGTAGTGGATCAGTTTCTCGGCGCCGATGATGCGGTGCTGCGGTCGGCGGAAGAAAAAAGAACCGC
CGTCACAGCCTTGTACGAGCGCCTACTGGAGAGCCGGCTCGCTGCGGTGCGCAGCTGTGACGGCAACGGT
GACGCGGCCGCGGGAGACGTGCAGGGCAGCCTGGAGGCATGGCGAGCGCGCCAAGCGTTCCTCTATGCTG
ACGTCGGCGACTTCTACTTTAACCAGCGCAAATTGCAGGACTACGTCCTGCGCTGTTGCCAGGACTCCGA
GATTGGCGGCTTGATGGACAAGCCTCAAACAGCGCACGACGGGTACCACACCTGCTACTCGCTCTCGGGG
TTGAGTGCAGCGCAGAACTTGCAGTACCGGACTCATGTCACCGCCGCCGATGCCGCCTACCCGTCCACCT
ACCTTGAACGCGCCTTTGCCCGCTCGTACCTCCCAGGGCGCGAGAGTGGCAGCACAAGCGGCAATGAGGA
AGGTAGCTGTGGCGTAGTGTTGGGCTTACCAAACACCTCCGCCGCTGCGGAGTCGATGCTTCTTCGGACG
ACGAGCCCGATCTTTAACATACATCAATCGCGCGTGCTCTCTGCCCTGCGTGCATGGAGCGTGAAAGCCT
TCGTGTAA
>AF467951 1, 798 ribosomal protein S2
ATGGCAGATACTCAGCCTGCTCAGGAGGCCCCCGCCGCCGACGCCCCCCGCGCGGAACGCAACTTCGGCC
GAGGCCGTGGCGGACGCGGTGGCCGTGGCCGTGGCCGCGGTGGCCCGGGTGAGGAGAAAGAGTGGGTCCC
GTGCACCAAGCTGGGCCGCCTCGTGAAGGCGCAGAAGGTCACCTCTCTGGAAGAGATCTTCCTCTTCTCG
ATGCCCATCAAGGAGCACCAGATCGTCGACACCCTCATCGCTGAGGGTAAGCTGCACGATGAGATGATGA
AGATTTACCCCGTGCAGAAGGCCACATCGGCCGGCCAGCGCACCCGCTTCAAGGCATTCAACGTCGTCGG
CGACTGTGACGGCCACATCGGCATCGGTGCCCGCGTCGGCAAGGAGGTGTCGCTGGCGATTCGCGCCTCG
ATGATTGCGGCCAAGCTCAACATCGTGCCGGTGCGCCGCGGCTACTGGGGCAACAAGATCGGTGAGCCAC
ACACGATCCCGATGAAGGTGACCGGCAAGTGCGGTTCCGTCGCGGTCCGTCTCGTGCCCGCGCCCCGCGG
TACCGGCATCGTCGCCGCCCCCGTGCCCAAGAAGATCCTCGAGTTTGCCGGTGTCGAGGACGTGTACACG
AGCTCCCGTGGCAAAACCCGCACGCACGGCAACCTAATCATGGCCACTTTCTACGCCCTGCGCAAGACCT
ACGGCTTCCTCACGCCCGACCTCTGGGCGGACACGGAGCCCAGCCGCGATCCGACCGACGAGCATGGTGA
GCTGCTTGCCGAGATGACCACCGCCTAA
>AF507947 1, 1149 ICAM-L
ATGGCGCAGCAGCGGGGGAAGAGGGCAGTGCCGTCAAAGGCAGCCGTGGCGAATGCGTCTGCAAGTGCGC
CTGACCCGCCGCCGTTCTTTTCCATGGGGCCCGCCACCTTCACCGACGCCATCGTCATCACCAAGGTCAT
TTCGGTCGTGCTGCCATTGCGCCACTTGCGTGACCGCAACATCCTGCATACGACGCTTCTGAAGAAGTTG
CTCCATCGTCCCTACGAGGCATCTGCAGAGGACCTCGCCCAACAGCAGCTCTCGCAACAGGCGACCGACG
ATGGCGGCAAGCATGGCCGCGGTGCACACAGCGCGCTCGACAACCTTGCGGCACCCACGTCGTCATCTTC
GTCGACACCCTACGTGATTGTCCACATCAAGTCCGCTGAGATTCACGCCGACACGGCTCGCCAGGTAAAC
CCGCGCGGCGATGTGCAGGTGAACGTGATGGTGACCCTCGTCGCAGCTCGCCTCCAGCACGGGATCTGCG
CCGGTGTCTCGGAGGCGTCGCCGTTCACGTCGTGCATGCAAGTGCGGGTACCGACAGTGGCCGTGTCCGC
CGCGGTGGCAGCCAGGGGCAGTACGGACGATGCGGCAACACTGTTACTCAGGGATGCTTCGGAGGCAGGC
ACCGATAATGTGTCGCAGGAAGGGGTCAAAGACGCGAAGGCAGAGCCGAGCGGTGGCGATGCAGCGCTCG
ATGCCTCCGTGTCAGCCACCACGACGGCTGCTGCCGCGAAGCGCAGCTGCTGCACTGGCGTCCCTGAGGA
TCAACAGCTGCGCGTGACAGCGCGGTGCCTAGAGGACGAGCCGATCGTGCCAGGTCAAGCAGTCTTGCTC
ATCTCCGAGCCGGCCGCTATGCGCTGCATGGGCATCCGGCCGCCGCGTGCAGCACCAGGCCCGTTTTACC
TAACAGAAACGACGCAACCGTCGGCTCAGTTCTCCAGCGGGGAGGCAGAGGGCGGGCCTGAGGCGAGGCC
GAAGAAGCGCGCACGCAAGTCACGCGCGTCCGCCGGCGGCACAGGTGGTGCTACACCAGCGCGTCGTATC
GTGTCCATCGAGTGCGCCATCCCGAAAAGAGAGGAGGATGGGGAGGGCGGCGCAGCGGCAGGCACCCGCA
AGGGCCCGCGTAAGCGACCACACACTTAA
>AF523363_0 12, 2826 ORF-q
ATGACGCGGTGGAACCAGCGGATGGCCAAGATGGCCTTCCGGCGCTCCAAATTCCGTCGCTTAGCCTGGG
GCGACGGTGACCCGTACCCCTACACCCCGCGCGCCACCTTCCGCTATCAGTGGGACGACTGGCCGATGTG
GGAAAAGATGTGGCACCTCGCTGTCGGCGTGCTGGGCGTCGAGTTTGCGGTGTGGGCCTACTACTCGAAA

CTGAACAGCCGCATGGACTGGGCGCGCGAGGAGGCGCTGCGTCGCATGCGTCTGCGCCGTGAGGCCCAGG
AGCTGATGATCCTCGAGGGCGAGACCTTCCCGAGCGACATCCAGCCGGACCGCCACTGA
>AF523363_1 935, 2826 QDPR
ATGAAAAATGTACTCCTCATCGGTGCTCGCGGCGCCCTTGGTCGTGCCGTCGCGAACGCCTTTGCCAACG
GCAAATGGTCCATCATCAGTGTAGATCAGGCAGCTGCTGTCCAGCAGGGCGATGAGTGCTGCGCTGTCAA
CCCGGCAAGCTCCATCGAGGAGCTGCAGCAGGCCTACAAGTCTGCGGTCACCGGACTCAAGGTTGACGCA
GTGATCAACGTGGCTGGCGGTTGGGCCGGCGGCTCTGTCGCGGACGCCAGCACCGCTGCCTCGACAGAGC
TGATGCTGAGACAATCGCTCTTCTCCTCCGTAGCGGCGGCACATGTGTTCAGTACGCAGGGTGAAAAAAA
TGGACTGCTTCTTCTCACCGGCGCAGCGGCTGCTGTGAGCCCCACACCCGGTATGATTGGATACGGCACA
GCAAAGTCTGCAGTGCACTTTCTCTGCCAGTCTATCGCGGAAGACCCATCCGTGTTGCCGACAGATGCGA
GTGTCCTGGCTATCCTCCCCACTATCCTTGACACTCCCGGAAATAGAAGTGCGATGCCTCATGCTGACCG
CTCCACGTGGACGTCGCTCGAGGACGTGGCGCAGCAAATCGTGGAGTGGAGCAATGGAAGTCGTCGTCCT
GCTAGCGGATCACTCGTTAAGATCGTTACAGAAAACTCGAAGACGCGTTTTATTGTGTAG
>AF523363_2 2164, 2826 20S proteasome beta 7 subunit
ATGGCGTCTGGCGCATCTGTCATCGCGATCAAGTACAACGGCGGCGTACTGATGGCGGCGGACACGCTGC
TGTCCTACGGGTCTCTCGCCAAGTGGCCCAACATCCCGCGCATCAAGCTGCTCGGCAGCCACTCCGCCGT
GTGCGCCACGGGCAGCTACGCCGACTTCCAGATGATGGCGAAGCAGGTGGAGGACAACATTGAGCGGCAG
AGGATGTACTACAACGTGGACGAGTTGAACCCCAGTGAGGTGTTCAGCTACCTGCACCGCTCCATCTACC
AGAAGCGCTGCGACTTCGAGCCGTGCCTGTGTCAGATGGTCTTCATTGGCGTGCGCGACAGCGAGACGTT
TCTGGCGGCCGTGGACGACGTCGGCACGCGCTGGGAGGACGACTGCGTCGCGACCGGCTACGGCGCGTAC
ATCGCACTGCCGCTGCTGCGCCAGGCGCTGGAGAAGAATCCGGGCGGCCTGTCGCGGGCCCAGGCGGTGC
AGATCCTCACCGACTGCCTGCGTGTGCTCTTCTACCGCGAGTGCCGCACCATCAACAAGTTTCAGATGGC
CGACGCCGCCGGCGACGGTGTGCGCATCAGCGAGCCCTTCGACGTGGAGACGAACTGGGAGTACGAGGGC
TTCTGCTTCGAGAAGACGGCCATCATCCGCTGA
>AF532102 29, 184 A2
ATGAAGATCTGCAGCGTGCGTCCGCTTGTCGTGTTGCTGGTGTGCGTCGCCGCGATGCTCGCACTCAGCG
CCTCCGCTGAGCCGCACAAGGCAGCCGTTGACGTCGGAACGCTCTCTGTGGGCCTGCAGGCCGTTGACGT
TGGCCCGGTGTCGTAA
>AF542055 208, 657 ribonuclease HI
ATGCAAGAAGTCACGATCTATTCCGATGGCGCCTGCAAAGGCAATCCCGGCCCCGGCGGCTGGGGTGCCG
TGCTGGTCGCCAATGGCCACGTCAAGGAGCTGTTCGGCGGCGAGGCCAATACCACCAACAACCGGATGGA
GCTGACCGCCGTCATCGAGGCGCTGCGCGCGCTGAAGCGGCCTTGCAAGGTGCAGGTCTGGACCGACTCC
CAGTACGTGCAGAAAGGCATCTCCGAATGGATCCGCGGCTGGAAGGCGCGCGGCTGGAAGACCGCGGAGA
AGAAGCCCGTCAAGAACGCCGACCTGTGGCAGGCGCTCGACGCCGCCGCGGCCGAGCACGACATCTCGTG
GCACTGGGTACGCGGCCACAACGGCCATCCGGGCAACGAACGGGCCGATGCGCTGGCCAATCGCGGTGTC
GCCTCGCTCACCGGCGCCCAGGTTGAGTAG
>AF544253 1, 906 ribonuclease H(35)
ATGACGGATACGCCATCGGCTCTCAGTGCGCGGCTGTCGACGCGACTCAGCGGCACGAAGCGCCCGCGCG
AGTCGCTTATCGAGTTCCGTGTGAAGGACATCATGGAGTCGCTTGGCGTGAGCGAGGATTACTTGGTATT
GGGCATCGATGAGGCAGGTCGAGGTCCCGTCATCGGCCCCATGGTCTACACCGGCGCTGTCATTTCGCTG
GGTGAGCACGACGACCTTGTGCGGTTGTGCCATGTGGCAGACAGTAAGATGCTTAATGAGCGACGCCGAC
TCGCCTCCCTGCAGCAGCTGCGCCAGCTCAAGACGTTCCGCTCCTTCACTGTGTGCGTCTCTCCCGAGGA
AATCTCGAAGACCATGACAGGCCGTAGCGGCCGTAACTCGAATACGCTGAGCCACGAGACAGCCATTCAA
ATCATCTCCGAGGCAACGCTGGCCTCCGCTGGCAAGCTCTGCGCCGCATACGTAGACACTGTCGGTCCAC
CTGAGACGTACCAGGCGCGCCTGGCTGGCCGTTTTCCTCACCTGCGTGTAACCGTTGCAAAGAAGGCCGA

CTCCATATTCCCAATTGTGTCCGCGGCGTCTATTGTTGCCAAGACGGTGCGAGACACCGCCATCGAGGCG
CTCGGCGAGAACATAGGCAGCGGGTACCCCAGTGATCCTCGCACGATGGAGTGGCTGCGGTCGCACGTGC
ATCGCTTTTTCAGTTTCCCGCACGCCTATGACTTTGCGCGGCACTCGTGGGGGCCTCTCGTGCAGCTTGC
CAACGACCCGGCGGTGCGCGTGCCCGTGGTGTGTGAGCAGGATCTCGAGGGGGCGAGACAGCAAGGGCGC
GGCGGCGACAGCCGGCAGCAGAAGCTCAGCTTCGCGAAGCCCTCGCCGAAGCGGCACATGGTGTAG
>AJ223860 211, 528 sw3.0
ATGTCCTCTAATTCCGCCGCTGCTGCCGTTTCCGCCGCCACGACCTCGCCGCAGAAGTCTTCTCGCTCGT
CGCCGAAGAGGGCGGCCGTGGGCAAGAAGACCGGCGCGAAGAAGGTTGCGAAGAAGACCGGCGCGAAGAA
GGTTGCGAAGAAGCCAGCGAAGAAGGTTGTGAAGAAGCCAGCGAAGAAGGTTGTGAAGAAGCCAGCGAAG
AAGGTTGTGAAGAAGGCTGTGAAGGCTGTGAAGAAGGCTGTGAAGAAGGTTGTGAAGGCTGTGAAGACCG
CGAAGAAGTCGTCGAAGAAATCCTCGGCGAAGAAGTAA
>AJ237587_0 1674, 9556 haspa1
ATGGGAAGCTCTTGCACGAAGGACTCCGCAAAGGTGCCCCAGAAAAGTGCGGGTAACATCGATACGACCA
CTCGAAGCGATGAGAAGGACGGCGTCCTTGTGCAGCAGAACGACGGGGATGTGCAGAAGAAGAGCGAAGA
TGGAGACAACGTGGGAGAGGGAGGCAAGGGCAATGAGGATGGTAACGATGATCAGCCGAAGGAGCACGCT
GCCGGCAACTAG
>AJ237587_1 3498, 9556 sherp1
ATGGACCAGGAGACAAGGGACCAGATGAAAAACGCCGCGGCGGAGGCGAAGGACAATGTGCACGACAAGA
TCCAGGAGCTGAAGGACGATGTGGGGAACAAGGCTGCAGAGGTCCGTGACGCAGTGAGCAGCACGGTTGA
GAGCATCAAGGACAAGCTAAGCGGTGGCTCGTAA
>AJ237587_3 7183, 9556 haspb
ATGGGAAGCTCTTGCACGAAGGACTCCGCAAAGGAGCCCCAGAAAAGTGCGGATAAGATCAAGAGTACCA
ATGAGACCAACCAGGGAGGGAATGCGAGTGGCTCCCGGAAGAGCGCCGGCGGTCGGGCCAACGAATATGA
CCCGAAGGATGATGGCTTCACGCCGAACAACGAAGACCGTTGCCCGAAGGAGGATGGCCATGCGCCGAAG
AACGATGACCATGCCCCGAAGGAGGATGGCCATGCGCCGAAGAACGATGACCATGCCCCGAAGGAGGATG
GCCATGCGCCGAAGAACGATGACCATGCCCCGAAGGAGGATGGCCATGCGCCGAAGAACGATGACCATGC
CCCGAAGGAGGATGGCCATGCGCCGAAGAACGATGACCATGCCCCGAAGGAGGATGGCCATGCGCCGAAG
AACGACGGGGATGTGCAGAAGAAGAGCGAAGATGGAGACAACGTGGGAGAGGGAGGCAAGGGCAATGAGG
ATGGTAACGATGATCAGCCGAAGGAGCACGCTGCCGGCAACTAG
>AJ237814 6, 302 lnp18
ATGACCTCGCCGCAGAAGTCTCCTCGCTCGTCGCCGAAGAAGACGGCTGCGAAGAAGGCCGCGGCGAAGA
AGGCCGCGGCGAAGAAGGCCGGGGCGAAGAAGGCCGGGGCGAAGAAGGCGGTGAGGAAGGTGGCTACGCC
GAAGAAGCCGGCGAAGAAGGCTGCGAAGAAGGCCGCGAAGAAGCCGGCGAAGAAGGTCGCGAAGAAGCCG
GCGAAGAAGGCTGCGAAGAAGCCGGCGAAGAAGCCGGCGAAGAAGGCTGCGAAGAAGGCCGCGAAGAAGG
CCGCGGCGAAGAAGTAA
>AJ243188 1, 1119 mkk
ATGAAGAATCGACCCGCTCTCGCGCTGGATGCGGCAGTTGTGGAGAAGTTCCAGCAGGACTCGAAGTCTT
ATCAAATCAACAGCAACAACTCTCTGCAGTTCCGTGCGTTCCTCTTCAATGAGGCCGGTATGTACACAAA
AGACGGCCGCGAGCTGCCGAGTACTGTGAAGAAGGATGACATTGACTACTCATCGAAGCGCAACGTCGGC
GCTGGAGCGAGCGGCGACGTTTTTTTCGCGCGCCTCAAAAAGGGCACATCGATTGCCTTGAAGCGTATTC
CCATCTCGTCCAAGGCGCATAGAGACGAGGTGGACCGTGAGCTGCAGGTTTTCATGGCCCGCGGCGATTC
CCCGTATGTGATGAATAACTACGGCGCCTTTTGGGATGCCGAGGACGATGCGATTGTGATCCCGATGGAG
TGGATGCCGTACACAGTGAAGGACCTCGGACTGTTTTGGGGCGGACTCAACGAGGCACTCTTGAAGGCAG
TCTTCTTTCAGGTGGTGTCTGGCCTGGTTTACCTGCATGATGTGAAGCGTGTGCTGCACCGTGACCTGAA
GCCGAGCAACCTGCTCATCAGCGAAACTGGCCACGTAAAGATTGCTGACTTTGGTGTGTCGAAGCTGATT

CAAACGCTTGCTGTGTCGTCAACGTACGTTGGCACCATGTGCTTCATGGCCCCAGAGAGGTTGGAGCAAG
GCATGTACGGTTTTAGCAGCGATGTCTGGTCACTGGGACTCACTATGATCGGTGCTGTCACCGGCAAGAA
CCCCTGGGCGCCGCCGGAGGAGATGAATCTCTACCAGCTGCTCGGTAAGATGGCTAACGGAAGCACACCG
ACGCTTCCTGAGTCCGGCGCGTTTTCCGACGACGTGAAAGACTTTGTGAAGCAGTGCCTCGAGCGTGACC
CGGACACCCGCCCAACGTGCGCAGAGCTGCTTCAGCACCAATTCTTTGATGGGGTAACCACCGAAAGTGC
TGTGGCGATGGTCAAGATGGCAGTGGAGCAGATGACTCGCCTCATCAATAACAACGCACAGAAGGAGACG
GAAGTGGCTCGATCACAGGAGTCTCTGGAGAAGGATGTAAAGGCACAGCTTGATAAGATGGTGCTCTAG
>AJ297564 440, 1585 PKAC2a
ATGCTAGTGAGTGTGAAGCCGAAACCTGAGGTGGCTGGCACAGCTCACGTACCCCTTGGGCCCACGGGGA
CGAATGTGCGCGGTGCGTTTCCCATCACCGCTGCGTCCAGATCGGCGCATGCTATGTTTCCTAGCACGTG
GTACTCCGCTTTAAAACCCAAGGTCGCGTGCAACTTCGCAAAGCCAGACACCTCCAGCTGGAAACTCTCA
GATTTCGAACTGAAGAACACGCTCGGCACCGGTTCCTTCGGCCGCGTGCGCATCGCCCACCGCAAGGGCA
CGGAGGAGTACTACGCGATCAAGTGCCTGAGAAAGCGCGAGATCATCAAGATGAAGCAGCAGCAGCACGT
TGCGCAGGAAAAGGGGATCCTAATGGAGCTGTGTCACCCGTTCATCGTGAACATGATGTGCTCCTTCCAG
GACGAGAAGAAGGTGTACTTTCTGCTGGAGTTTGTCATGGGCGGCGAGATGTTCACGCACCTGCGCACTG
CCGGGCGGTTCCCGAATGACGTTGCGAAGTTCTACCACGCGGAGCTGGTGCTTGCGTTCGAGTATCTGCA
CTCGCTGGACGTGATCTACCGCGATCTGAAGCCGGAGAATCTGCTGCTGGACAACAAGGGGCATGTGAAG
ATGACGGACTTTGGGTTTGCGAAGAAGGTGCCGGACCGGACGTTCACGCTGTGCGGGACACCGGAGTACC
TTGCGCCGGAGGTGATCCAGAGCAAGGGCCACGGGAAGGCGGTGGACTGGTGGACGATGGGCGTGCTGCT
GTACGAATTCATTGCCGGATACCCGCCGTTCTACGACGACACGCCGTTCCGGATTTACGAGAAGATCCTT
GCTGGGCGGCTGAAGTTCCCGAACTGGTTTGACGGCCGCGCGCGCGACCTCGTGAAGGGGCTGCTGCAGA
CGGACCATACGAAGCGCCTGGGGACGCTGAAAGGCGGGCCGGCGGACGTGAAGAACCATCCGTACTTCCA
CGGTGCGAACTGGGACAAGCTGTACGCACGTTACTACCCCGCGCCGATCCCTGTGCGGGTGAAGAGCCCT
GGCGACACGAGCAACTTCGAGAAGTACCCGGACAGCCCGGTCGACCGCACGCCTGCGCTGACGTCGGCGC
AGCAGGCCGAATTGAAGGGGTTTTAA
>AJ297954 64, 838 PKAC2a
ATGCTAGTGAGTGTGAAGCCGAAACCTGAGGTGGCTGGCACAGCTCACGTACCCCTTGGGCCCACGGGGA
CGAATGTGCGCGGTGCGTTTCCCATCACCGCTGCGTCCAGATCGGCGCATGCTATGTTTCCTAGCACGTG
GTACTCCGCTTTAAAACCCAAGGCCGCTTATTACTTCGCAAAGCCAGACACCTCCAGCTGGAAACTCTCA
GATTTCGAACTGAAGAACACGCTCGGCACCGGTTCCTTCGGCCGCGTGCGCATCGCCCACCGCAAGGGCA
CGGAGGAGTACTACGCGATCAAGTGCCTGAGAAAGCGCGAGATCATCAAGATGAAGCAGCAGCAGCACGT
TGCGCAGGAAAAGGGGATCCTAATGGAGCTGTGTCACCCGTTCATCGTGAACATGATGTGCTCCTTCCAG
GACGAGAAGAAGGTGTACTTTCTGCTGGAGTTTGTCATGGGCGGCGAGATGTTCACGCACCTGCGCACTG
CCGGGCGGTTCCCGAATGACGTTGCGAAGTTCTACCACGCGAAGCTGGTGCTTGCGTTCGAGTATCTGCA
CTCGCTGGACGTGATCTACCGCGATCTGAAGCCGGAGAATCTGCTGCTGGACAACAAGGGGCATGTGAAG
ATGACGGACTTTGGGTTTGCGAAGAAGGTGCCGGACCGGACGTTCACGCTGTGCGGGACACCGGAGTACC
TTGCGCCGGAGGTGATCCAGAGCAAGGGCCACGGGAAGGCGGTGGACTGGTGGACGATGGGCGTGCTGCT
GTACG
>AJ298026 1, 705 mpk1
GGCAGCGGCGCCTACGGAATCGTCTGGTGTGCCCTCGACCGCCGCACGGGCAAGTGCGTTGCCCTCAAAA
AGGTCTACGATGCCTTTGGCAACGTTCAAGATGCGCAGCGCACCTATCGGGAAGTGATGCTTTTGCAGCG
ACTGCGGCACAACCCCTTCATCGTCGGCATCCTCGACGTGATTCGTGCGGCCAACGACATTGACCTGTAC
CTCGTTTTTGAGTTGATAGAGGCCGATCTCACAGCCATTATCCGTAAAAACCTTCTGCAGCGCGATCACA
AGCGCTTCCTCACCTATCAGTTACTTCGCACAGTGGCGCAGCTTCACGCACAGAACATCATTCACCGGGA
TTTGAAGCCGGCCAACGTATTTGTGAGCAGCGACTGCTCGATCAAGCTAGGGGACTTTGGTCTGGCTCGC

ACGTTTCGCAGCGGCTTCGACAACGAGCAGGAGTTTCTCGACCTGACCGACTACATCGCAACTCGGTGGT
ACCGGTCGCCGGAGATTTTGGTCAAGTCGCGCGCGTACTCCACAGCGATGGACATGTGGGCCGTCGGGTG
TGTGATTGGGGAGATGCTGCTGGGCCACCCACTCTTCGAGGGCCGCAACACGCTGGATCAACTCCGCCTG
ATCGTCGAGGCTATCGGCGTGCCGAGCGACGCAGACGTGCGCAGCCTTCACTCGCCCGAGCTCGAGACTC
TCATT
>AJ298055 1, 855 prp8
GAATTCTACATCTACGACGACTGGCAGCAGAGTTTGCAGTTCCAACCGATCACGTGCTTCAACCTACTGA
ACCTGGTTCTGCGTGGCTACCACATAAATCTGCAGCGCACGCGGCAAATTCTCGTTCCTGACCTGCACGT
CGAGGTGAGCGCGTCTCACTTCTGGCCCACGTACGCGACCCGTCAGGAATGGGAGCAGGTGTCCATCCGT
CTGCAGGAGATGATCATCGCCGACGCCGCGCGCCGCATGGACGTGAGCCCGAAGGACTTCACGGAAAAGG
AGAAGGAGGGCATTCTTCTGGGCAAGAGAATGACCAATGTGGAGATTCAGCAGGAGGAGATGAAAGAGAT
CGAGGCCATGCAGCGCACAAAGCTGGCAGAGACGCAGACGGTCAACGTTGTCACGAGCAGCGGCGACGTC
GTGAAGCGCAAGGTGAAGGCCGCCTTCGACTTTGGAGCGACGGCGTTGAACAACAACTGGCGTCCCCGCT
CCTTGGCCGATGCCGCCTTTCTTGACGAGAACACTCCCATCACCTTCGACGCCGCCGGCGCCACCGGGGC
AAGCGACCAGCTCATCTTCTCTGAGGACGCGATTCAGAAGTTGCTGGCGTGCTGCGACGTCAAGGTTCAG
TGCTGCGCGTACATGCTTGGCCATGCGCTGCCCGACTCGCCAAACATCAAGGAGGTGCTGTGTGTCATGA
TCCCGCCGCAGTTCGGGACGGCTGTCGAGGCCCGCACGCCACCACGCATTCCGTTTGACGCGGCAGCACT
GCAGGAGGCGAACCTGTCCTTCTTGGGACTCATGAGGATTGGCGAGAGCGAGGCGCAGCTGACCTCGCAC
GACCTCGCCCTCCAG
>AJ304943 1, 859 dnap
CGATACCAGGAGCTGAACATCTCGTCGTGGTCCACCATTGGCCGCCTGGACCTGAAACGACTTCCGCGCT
TCCAGGGAACCGCGACTAACGTGAATCAGGAAAAAGAGACGTGCATCGGGCGCCTTGTTGTGGACTCCTA
CTCACTCTCGCGCGAGCACTACAAGACAGTGAATTACAGGCTGCTGTCGCTTGCAGACCAGATGCAGCTG
CAGGGGATTACAAAGGGCAGCAACAACTTCGAGCCTGGCACCTCGGTGCTCACACCTGCGATGCTGTCGG
CATCGCGCGACATCTACGATGTGCTGCTGCAGGTGTGCAACTGCGCTGTGCTGTCCACAGCGGTCGTCTC
ACATCTCGATGTGATTCGACTGACCAAGCGCCTCACCACGATTGCAGGAAACCTGTGGAGCCGTACCCTC
TTCGGTGCTCGCTCCGAGCGCATCGAGTATCTCCTGCTGCACACGTTCCACGACCTCAAGTTCATTACCC
CTGATCGCTATGTGCAGGACTTCAAGCGGGGCCGCGACGACGACGAGGAGGATGGCAAACGGAAGGCCAA
GTACCAAGGCGGCATGGTACTCGACCCCAAGTGCGGCCTTTACTCGGATTACATTCTCCTCCTCGACTTC
AACTCCCTGTACCCGTCGCTGATTCAGGAGTTCAGCATTTGCTTCACCACCGTCGATCGTGAAAATCGGA
GCGAGATCGATGTGCCACCACCGGAGAACCTCATCTGTGCCTCGTGCGCCGCAGCAGGCCTCTCCGCTCC
GTGCTTGCACAAGTGTGTGCTGCCGAAGGTGATCAAGAGTCTCGTAGACAGCCGTCGCGAGGTGAAGCGG
TTGATGAAGACAGAGAAGA
>AJ304948 1, 1209 rnap
TCGTGAAGGCGCTGGAGGATGTGCACGCCTCCTACGACGGCACGGTGCGGAACAACCAGGAGCTCATCCA
GCTGGCGTACGGCGAAGATCTGGACGGTGCTCGCATTGAGGGCAACCAGGCCTTCCCGATCCCGCACATG
ACGAACAGTGAGATGGCTGACAAGTACCGCTACGAGTACAACGACGAAGGCAGCTTCTCGGAGAACATGG
GCGGCCATTACATGGATCCGTTTGTGCGCGACTCGCTGCTGGCCGACCCGCAGAGGCTGTTGAAGCTGCA
GGAGGAGTTTGAGCAGCTGATGAGGGATCGCGCGATGTCGNNNCTCGTGATCGACATGGAGGACAAGAAC
AAGCTCAAGATGAACCTGCCCGTGAACGTCGCGCGGCTCATCCAGAACGCCGGCACGACGATGGGCAAGC
GCAGCCAGGTGTCCAATCTAAACCCGATCACTGTGATCAACCGCGTGGAGCTGCAGGAGGACCTCGTGCA
GCTGTTCCCGTCCTACCACAAGGACTACAACGGCCGCTTCGTTAATGTGCTGAGCCAGCAGCGCGTGGAG
CGGGCCCTGACGCTCTTTGGCATCCACTTGCGCCAGATCCTCGGCTCGAAGCGGGTGCTGAAGGAGTACA
AGCTGAACGACAAGGCGTTCGAGTACCTGCTGAAGGAGATCCGCACCAAGTACCAGCAGTCCCTCATCAC
CCCTGGTGAGATCATCGGCGCCATTGCCGCGCAGTCGTCGGGTGAGCCCGCCACCCAGATGACCCTGAAT

ACCTTCCACAACGCCGGCATTTCGTCCAAGAACGTGACCCTCGCGGTGCCGCGGCTGTTGGAGCTGCTGA
ATGTGAGCAGGAACCAGCGCAACGCCAGCGTGGCCGTCTGCCTGATCCGCGAGTACCAGAAGCGCAACAA
GGCTCAGGAGGCGCAGCAGTTTATCGAGTACTGCACGCTGGCGAATATAACGACAACGGTGCAGATCATA
TACGACCCCGACCCGCGCAACACCGTCGTGGCCGAGGACGAGGAGATGATCCGGTGGGAGCAGGCGGTGA
TGAATGCAGAGGATGAGGAGCCAGATCGGGAGCAGCCGCCGTCACCTTTCATTGCACGCCTCATCCTCGA
CAACGACCTCTTCAACGACAAGCGACTTAACATGAAAGACGTGAAAAGTGCGATCCGGCAGGTGGACGAC
ACTTACATGGTGCAGGCAA
>AJ311570 1, 1959 tryS
ATGTCATCTCTGCAGCGCGCGTCTGTGAGCTTTAATAAGCCGGGCCACATTCCGTTTGGTGCTGTGCAGG
GATACGCCCCGGGTGGCGTCCCGGCGTACAGCAACAAGCACGATCACTACTTCTCCGGCGAGCGCAACAT
TGAGGACAACATCTTCTTCGGCTTCAAGTACCAGTGCGTCGAATTTGCGCGCCGCTGGCTACTGGTGCGT
AAGGGACTGTTGCTGCCCGACGTGAACTGGGCCTGCCACATTTTCCAATTGAAGGAGGTGCGGGACGCCG
CCACGACGGAGAGCTTCGCGGTATTGCAAGTGCGCAACGGTACTACGACGAAGCCAGAGGCCGACGCGCT
TCTTGTCTACCCCTCCACCGATGCAAATCCAGTTGGTCATGTCGGCACCATCACCGAGGTGGGTGACGAC
TACGTATGTGTCGCGGATCAGAACTACCGCTTCCACAAGTGGGAGTCCTCCTGCGCGTACAAGCTGAAGC
TTGATCACAGGGACGGCATCTGGACCATCATCGATGACATTGACGCGGACGAAATCGAGATCCCACTTGG
GTGGTTGACGTTTCCTGGCCGCGCAAATCGACCGGAAGGGGCGCCGCCGGTGGCGCTGCATCCGTCACTG
CACTTCAAGGAGCCGCCGAAGCCGTATCTGCTGCGCCGTAACTTTCTGCCGACGGAGTCCAAGGCGAACT
GGCTCGACATGAACAACCCGGCGGAGCGGCTATTTGTGGAGGAGTTTGGCATGGACGTCAGCCGTACCCG
CCTGGAGGAGAAGGTCGTCAGCTACTACGAGTCGAATCACGAGTTTCACCTGCGGTGCGTCGCGTACGGA
ACGCAGCTGCACGCCATCTTCATGGAGGCAACCGCGCAGGTGATCGAGAGCGATGAGAAGCTCCGGCTCT
TTGCCATTCCTGAGGAGTTCTGGCCCCGCATCCGCCACTCGTGGAAGTACCAGCAGACGTACATTTCTGG
TCGCTTCGACTTCGCCTTCAACAACGAGACGGGCGAGGTCAAGTGCTTCGAGTACAATGCTGACAGCGCG
TCGACGCTGCTAGAGTGCGGCCTCATTCAGCAAAAGTGGGCCGAGTCGGTGGGGCTGGACAAGCAGGACA
CGCGCGGCTCCGGCTTCGCGGTGGAGCGCAACCTCAAGATGGCGTGGGCAAACAGCGGGGCGACTGGCCG
CGTTCACTTCTGCGTTGATGAGGAAAGGGAAGAGCAGTACACGGCCCTCTACTGCATGCAGGCCGCTGAG
GCAGTCGGGTTGGAGGGCAAGCTGTGCATCTTGTTCGATGAGTTCCGCTTTGATGATAATGGTCACGTTG
TCGATAGCGACGGCGTGCGTGTGCGCAATGTGTGGAAGACGTGGATGTGGGAGTCGGCCATCACGGACTA
TTACGCCGCCCGGGAGGAGCGAGGCGAGAACTGGAAGCCGTCCCCGAAGGACAAGGTGCGATTGTGTGAC
TTGCTCCTCGGCGATGACTGGGAAATTCTCTACTTCGAGCCGATGTGGAAAGTCATCCCGAGCAACAAGG
CCATCCTGCCCATGATCTACCACAACCACCCCGAACACCCAGCCATCTTGAAGGCGGAGTACGAGTTGAC
GGACGAGCTGCGCAAGCACGGGTACGCCAAGAAGCCGATTGTCGGCCGCGTGGGCAGCAACGTTATCATC
ACCTCCGGCGACGGCGTGGTGCACGCTGAGTCTGGTGGCAAATATGGCAAGCGCAACATGATTTACCAAC
AGCTGTTTGAGCTGAAGAAGCAGGATGACTACTACGCCATCATCGGCGGCTGGATGATCGGCGACGCCTT
CAGCGGCACCGGCATCCGTGAGGACAAGTCAGTCATCACCGGCGTGGACAGCCCCTTCGCTGCCGTTCGC
ATCAAGACCGACAAACTTCCGCACCCCGTGACGCTCAAGGATATCGACAAGATGGCCGAGGACGAGTAA
>AJ313201 1, 1113 MST
ATGTCTGCTCCTGCTGCTGCGCCGAAACACCCGGGCAAGGTGTTCCTGGACCCGAGCGAGGTAAAGGACC
ACCTTGCTGAGTACCGCATCGTGGACTGCCGGTACAGCTTGAAGATAAAGGACCACGGCAGCATCCAGTA
CGCGAAGGAGCACGTGAAGAGCGCCATCCGCGCCGATGTGGATACGAACCTCTCTAAGTTGGTGCCCACC
AGCACCGCCCGGCATCCGCTACCGCCCTGTGCTGAGTTTATCGACTGGTGCATGGCGAACGGGATGGCGG
GAGAGCTGCCAGTGCTCTGCTACGATGACGAGTGCGGCGCCATGGGTGGATGCCGCCTGTGGTGGATGCT
GAACTCTCTTGGCGCCGACGCGTACGTGATCAACGGCGGCTTTCAGGCCTGCAAGGCTGCGGGGCTGGAG
ATGGAGTCCGGCGAGCCCTCGTCGCTGCCAAGACCCGCAACGCACTGGCCCTTCAAGACGGCCTTTCAGC
ATCACTACCTTGTGGATGAAATCCCGCCCAACGCAATCATCACCGACGCGCGCTCCGCCGACCGCTTCGC

CTCGACAGTACGACCTTACGCCGCAGACAAGATGCCAGGCCACATCGAAGGTGCGCGTAACCTCCCCTAC
ACGTCGCACCTCGTGACACGCGGTGACGGCAAGGTGCTGCGCAGTGAGGAAGAGATCCGCCACAACATCA
TGACCGTCGTGCAAGGCGCGGGTGACGCGGCTGATCTATCGAGCTTCGTCTTCTCCTGCGGCAGCGGCGT
CACCGCCTGCATCAATATCGCCCTGGTGCACCACCTCGGCCTGGGCCATCCGTACCTCTACTGTGGCTCC
TGGTCTGAGTACAGCGGCCTCTTCCGCCCCCCCATAATGCGCAGCATCATCGACGACTACGGCATGTGCA
TGCAAATGCAGACCCCTAGCCTCGGCGACAACCCGAAGGCAAACCTCGACACCATGACGCTGAAGGTCGA
CGGCGCGCCCTGCGAGAGACCCGATGCGGAGGTGCAGAGCGCCGCAACCCACCTCCACGCTGGCGAGGCC
GCTACCGTGTACTTCAAGAGCGGCCGCGTCGTCACGATCGAGGTGCCGGCAGTGCCCAACTAA
>AJ548776 728, 1069 icp
ATGATCGCCCCGCTCAGTGTGAAGGACAACGACAAGTGGGTGGACACGCATGTCGGCAAAACGACCGAGA
TCCACCTGAAGGGCAACCCGACCACTGGATACATGTGGACGCGCGTTGGTTTTGTGGGCAAAGATGTGCT
CAGCGATGAGATCCTGGAAGTTGTCTGCAAGTACACGCCGACACCATCCTCGACTCCTATGGTGGGAGTT
GGTGGCATCTACGTTGTGCTCGTGAAACCGCGGAAAAGGGGCCATCACACGCTGGAGCTGGTCTACACGC
GCCCCTTCGAGGGCATCAAGCCAGAAAACGAGCGGTACACGTTGCACCTCAACGTGAAGTAG
>AJ548878 749, 1102 icp
ATGCAACCAAAAATGACCGCCCCGCTCACTATGAAGGACAACAACAAGTGTTTGAGCGTGCGCGTCGGCA
GCACGCTCGAGATCCACCTGGAGGGCAACCCTACCACTGGCTACACGTGGACGCGCGTCGGCTTTGTGGG
CAAGGAGATGCTCAGCGATGAGCACCTGGAGGTCACCTCCAAGTACACGCCGAAACCAGTCTCGGGTTCT
ATGGTGGGGGCTGGTGGCTCCTACACGGTGTTCGTGAAACCGCTGAGAAAGGGCCAGCACGCGGTACAGC
TGGTCTACGCGCGCCCTTTCGAGGGCCCTAAGCCAGATAACGAGAGGTACACGTTGCACCTCAACGTTGA
GTAG
>AJ578436 34, 633 top1a
ATGAAGGTGGAGAATAGCAAGATGGAGGTGAAGCGAGTGCAGAGCTACTCCAATGAAGATGAGGAGATCA
ACGAGGAGGACCTGAACTGGTGGGAGCAGGAAAACCTCCGTATCGCCATGAAGGGCGAGCGTCGCTGGGA
GACGCTGGCCCACAATGGCGTCCTTTTTCCACCCGAGTACGAGCCCCACGGCATCCCCATCTTCTACGAT
GGACGCGAGTTTAAGATGACGCCGGAGGAAGAAGAGGTGGCGACAATGTTCGCCGTGATGAAGGAGCACG
ACTACTACCGCATGGAGGTGTTCTGGCGCAACTTCTTCGAAAGCTGGCGCGAGATCCTGGGCAAGCGCCA
GCACCCCATCCGTCGCCTGGAGCTCTGCGACTTCGAGCCCATTTATCAGTGGTACCTCGTCCAGCGGGAA
AAGAAGCTCAGCCGGACGAAAGAAGAGAAGGAGGCCATCAAGGAGAAGCAGGACGCGGAGGCAGAGCCGT
ACCGCTACTGCGTGTGGGACGGACGTCGCGAGCAGGTGGCGAATTTCCGCGTGGAGCCGCCGGGCCTATT
CCGCGGACGCGGCAAGCACCCCCTAATGGGCAAGCTGAA
>AJ582069 1, 1353 tdr1
ATGGCCGCGCGCGCGCTAAAGCTGTACGTGTCGGCGACGTGCCCGTTCTGTCACCGTGTGGAGATCGTTG
CGCGGGAGAAGCAGGTCTCCTACGATCGGGTTGCTGTTGGGCTTCGCGAGGAGATGCCGCAATGGTACAA
GCAGATCAACCCGCGTGAGACAGTGCCGACGCTGGAGGTCGGCAATGCGGAGAAGCGGTTTGTGTTCGAG
TCGATGCTGATCGCGCAGTACCTGGACAACAGTGGCGCGCCCGCGGGTGCGCTGATGGGTGCCTCGTCGG
CGCAACGACACCAGATTGAGTTCTTCCTCGCCCAGGTCGGCGATTTCATTGCTGCTGCGCACGGGCTACT
CCGCGACCCGCTGAGTGGTGAGAAGCGCAAGGCCGTGGATGACAACGCGGCGTACGTGGACGGGCTGCTC
GCGGCGAACCAGACGACGGGGCCATACTACTGCGACGGCGAGTTCACGATGGCGGACGTTGCGCTTGTGC
CGTTCCTGGTGCGACTGAAACCTGCTCTGATGTACTACGCCGGGTACGACGTGTTCTGCAAGGCGCCACG
AATGAAGGTGCTGTGGGCCGCTGCTGCTCAACGCACATCTGTGCGTGAGACGTCGCCGACGGCGGCACAG
TGCATCGAGAACTACCGCCACCTGGTTCCGGAGAGCGCGCCAATGATGGGCGCCAATGGCGGGCACGTGC
TGTACAGCAATCTTTTCTGTCCTTTTGTGGACCGCGCACGCCTCGCGTGTGAGCTGCGCAAGTTCCAGGT
GCACACGGTGGAGGTGCCGCTGCATCCAGAGCCGGAGTGGTACAAATATATCAATCCCCGCGATACGGTG
CCTGCGCTATTTACGCCGAGCGGCGAGGCTGTACACGAGTCGCAGCTGATTGTTCAGTACATCGACTGCG

TGGCGACGGAGGGTACTGCGCTGGTGCCGCGTGGGGACGCGGAGAAGGAGTACGAGGTGGGTTTCTTCGT
GGAAAACGCTGGGTATTTCGTTGGAGGACTGATGTCGTGGATCATCCGTGGTGGCGAGGATGCGAAGGCT
GAGCTTCAGTGGGCTGCTGGCGAGCTCGAGCAGCAGCTGGCGAAACACCCGTTTGGTGAGGGCCCCTTCT
TCGGCGGCAAGAGGATGAACGCTGGCGATGTGGCCATTCTACCTTTCCTGGTCCGTGCAAAGGCGTTTAT
GCCGGAGTTTAGTGGCGGGTACGATCTCTTCGCCCACTTTCCGCTGCTGAATGTGCTGGCAGAGGCTGGC
ATGGCGGCACCGGAGGCGAAGGCGGTGTTTCGTACACTCGAGGAGTACAAGGAGCACATCCGTCAGCGTC
AACGGAGGGCCCAGGGCGGGTAA
>AY026067 1, 528 dihydrolipoamide dehydrogenase-like protein
GAGAGCCTCGATCGCTACCTCGAGATTGACGAGGTCAAGATGCGGCAGTCAATGGAACAAGTGAGCCGCA
TCCGCGAGGAGCAGATCCAGGCCGCACTCAAAGCCTCACCGAACGTAGAACTCGTGCTTGGCAGAGCGAC
CTTTAGCAGCAACCACGAGATCCAGTGCCATAGCAAGCGGACGAAGGAGTTCCGCAGCATCACGGCGGAC
TATTTCATCGTCGCCACCGGATCGAAGCCACGCAAGCACCCTTACGTGGCAGCAGACGGGCGGTTGGTGA
TGACCTCCGACCACATCATGCGAGCCCCGCTGCCGAAGAGCTTGGTGATCGTGGGCGCTGGCGTGATCGG
CTGCGAGTTTGCCAGCATCATCGGCCGCCTCGGCAAAACGAAAGTCTCCATCATCGACAAGGCGCCGCAC
ATTCTGCCACGGGAGGACCCCGACATTGTTCGCATGATCGAGAGTGGAATGGACCGCGCTGGCATCGTTG
TCCACCACAACTCCGACCTCTATGATATGCAGCCGTG
>AY034610_1 2090, 3538 P9
ATGGAAGAGGGCGCGCCCGCTAATGCGACAGCGTCACTACCGCCGTCCTATGAGTCTGTCCTCGTTTTTC
TTACAACGCTGGCGCAGTGGCTCAAGCATGCGCGAGAGCGCCTGAAATGCCGAGACGAAGTCGGCCGACA
CGCAATTGCGGTGGAGGTCCTCACCCGCCACCGCGAACGCGCCTATGAGCGGCTGGCAGAATCTGTATGG
CGTCATACAGCTGCTGCCGGTGACATAAGCACATCACTTTTTCCCTCTGCTTCAGTCGAGCTGGCCGCGT
GGGTGCTGCTGCGGCTTCTCGTCCTCCTCCTTACCTGGTGGTTGCTGATTCGGCTGGTGCGCGCGACCAC
CCTACCGCGACTGCGCGCTCGCCAAATTGCGGTGCACCCTGACGTGCATCTGTCAACGAGCGTCGGAAAT
GCACAGGCGGTAGAGGCGTTTATTCATCCCGACGCTGCTCAGCGCGGCATCAACGCCACGCTGCTGTTGA
CTGTCAGCGCTTGCGTGTCGGTGGCGTGCCTGCTCATTCTCTGCCTGCTTACAATGCATGTATGGGTGGA
CGTAGTGCTGTTGCGCCTGCTCCAACATTGGACCGCCCCACTACGGCAGGCACAGGCGTGGGTGGCGTCG
CATTGGTTGTGGCCCTCCAACAGCCCGCAGCCGGGCTCACTGCGAGCGCCTGCCGCCCCTGTGGAGCAGT
ACGCAACTTCTTTCATTAGTGTTGTTTCTGGACTAAATTCCAGCTTGTTCAGCAAGCTCGCGTGGCTGCG
TCGAGTGACACAGCAGGTGAAGCTGATGTACCTGTGGGGCATGGTCGGAGGGACCATGTTGGGGGTGGCG
CTCTGGCTACTTGGCTACTCCTCTAGGCTGCTGCGGACCTACAACACGAGTCTGCCGTACTTTGAAGAGA
ATGACCCTCTACTGCGCTGGCTGGGCCAGCAGGAGGCGGCGGCCGCTCAGCAACGTACCAACGCGGCGTT
TGCCGCTCTGCTTGAAAGCCAGCATAGGCAAGAGCGGGTAATGGAGAGGCTGGCAAGCTCATTGGCGCCC
GCGACTCCTCGACAGCGGCAGCGCTATCTGCAAATGACGGAAGAAGGAGTAGAATCGCATTGCTTCGTGG
ATGATGCTGCGCTAGCGGCAGCGACAAGCGACGCCACAGAAGACGGGCGGTGCAATGGTGAGACAGCCGC
AGGCGGAACTGACGCGGTGATCATCGGCAAAGGCTGCAGCGGCGGTGGCGATCCAGGAAAGTTGCGTGTG
GAATCTGGTCCTTCACCCGATAAAGCTTTGGCCGCTGCCTTCGAAGAAGCAGCGGCGGCAGCGTCGGCGA
CGCGAGATGAAGGAGCCGCTGAGGCTAGCTGCGGCACAACACCGCAGGCGGAGGAGAAGGAGGGGGCAGC
AACCACAGCTTCGCGGATGGCAAAAGAAGGAGAGAGTCCTAGGAGCTAG
>AY035390 609, 3317 A14
ATGATGCAGGACACTGCTTCACCCTTTCCCCACCACCGTACGCCGGCGACGTACCAGGCCGGCTTGCATT
ATCAGGCCAACGGACGCGAAAGCGCGGCGCCGCTGCTTACCTCGTCGGCTGGAAATGCTGCCAACGGTAA
GCGGCTCAGTCACTGGGGCCGCAACGTTTTGCGTGGCTTTTACCATGTCAAATGGCATCAGCGCACGCAG
TGTGCCGTTGTAGCGTGTTTGCTGTGGTTCTTCATCCTTTTTGTGCTCCTGCACACTTTTCTTCAGGTGT
CGGGTCTGACGCAGTCGATTTATGCGGTTTTCTCGAGGCCGACTTCCGGGCTGCAAACACTCCTGGAGGA
CGAGCTCGTTATACCTAGCCCTGTTGTGCCACCGAATGGAGCATTTTGTGGTCTGTGTACAGAGGGCCTC

GTTGTCTTCCGTCCAGCTAGTGCCGCCACATCCCTGGCTACGACGCCTGCCCAGACCGAGATGGTAGTGT
GCGTGGAAACGATGGAGGAAGCCTGGTTGTCAGCGAGCGAGTCGGAGCCTCTCAGCACTGTACGAGACTG
TGTAGCAAGGCTCTATAACCTGGGTCTCCTGCAGCCTCCTCGTACTACCATAACCGCGGTGAATGACACG
TGTGCAGCAGTGCGCGTGACAGTGCAGGTGCTCTCAGGCGAGTCAGAAGAGGCAGATCGGAACAAGTGCA
GCAGCGGGTTCGTGGCGGAGCTGCAGATTTCATCGACAGAACGGGAGAAGGTGGAGGTGCACACTCAGCG
GTTCCCGTCGTGGCCGCTCTGCCCTGTCGCCTCACCGGAGGCTATTGATGCTAAGGATGGACACCGAGGT
GGCAGTGACGGTAATCATTACATCGTGCCCGCGTACTTTGCGACAGCAGCTGTTCGAGACACAGACGCAG
ACTGCGATGTCCCACCGCCGTGGAGAATTGACACTATCGAGGACCTTGATGAGGTGGTACTGCGTTTCGC
CGGGGACGCGGCTCAGACCACAGAGACATCGTCGCTGCTGGCGAGTGACCTGCGCTGCCCGACCTACTTC
CTCACGGCAAACCTTGGCCGCTTTGGCCGCCATCACAATCAGCTGCAGGAGATCTTGAACGGCATCTCGC
TGGCCAGGAGATCGAACCGCACGTTTATTTTGCCACCCTTTGTGCCGGCGCTGTACATGTCGTACTTGAG
GCTAAATCCAGAGCTTCTGTACGGATGGAATGCGCTACGCGGAAACGGCCACTACTGTGTTCTGTCGTAC
GCCGAGGCGCGGCCAATACTGCGGCGTCTGCGTGAGCGCGATGGGGTAGTGAGTATGGAACGGGTCAGTT
TTGCAGCCACTGCAGACCTGCACGTGCTGTTCAACACCACAGAAGAGCACGACAGTTATGCATGGGGGTC
CATGCCTCGCGTGCCGGGCACGGATGGCGCGTTTGTGTACGATGCGGATGAATGGTTCTCGATGGGGGTG
CGGCGAAGGGAGGAGATGACAGCGCACACCGCAAGCAATACCTCATCAGTGCCATCGCCGCTAACGTTCC
ACGAGGCAAGGCCGCCTAGTGCGGAGGAGTGTAGCGGCTTCGCCGGCCCCGCTCTGAACAAGAACTCAAC
GCAGTGGGAAAGGATTCGGAGGTGCACCACTGCGTTTCTGCGCACCTACGGCGATGGTGACGGGAGACGC
GAGTCGTCGTTAGCCTCACGGCGACAGCCACGCATTGTGGTGATTTCGAGCGTCACAGCGTTTCATCTGC
GGCCGACGCTGACGGAAATGACCAGGCTGCTTGGCCTCCTGCGGCCGTCACCGACACTCACGGTGGAGAT
TGGCCGGTACTACCGACTCTATGCGACGCAGCTCCAGTGGCCAGCCAACACCGATCCGAAGCGCTCTTTT
TCAAACGTGCTGCAGCCATTTCGTTACAAGAGCACCATTGGTGTGCACGTACGCCGTCGCGAGTTTACGT
GTCGCGAGGAGGCCGAGCACCCCAGCGCCACCATCATCGCTATGTCGGAGGCGCGCTACCAGTTTGACGG
CACAGGTAAAAACGATAGCGCCGCTGCTGGGCGCCCCATCACCACCGTAGCGCGACTGGCGAGCGACTGC
GGATGGAATTCGCAGTCGCTACTGCACATCTACGACACCTATGCGAGCTGGATGCGATCGCGACAGGCGC
AGGGGCTGAGGCGGTCGCGTTGCTTCGCGCATGTGCAGACTTCAAACAAACAGGTGCTGCGGAGCTACGT
CGCCTTTGACGAGCAAGTCGGCCCCATTGGCTCTCAGCTGCAAGCTGCACTGCAGCAGCGCTACAACCCA
CTAAAGAAGAAGCGCGCCGCCAATGGATATCCACCCATCTATGCCGCATTCTACGACCACCGGCCCCGCA
AAGACCTTTTCCAGACTTACCAAGAAATCCTGGCAAGATGGGAGCATTCATCCGCAGACACGCAACGCGC
CGACGGGGAAGCCGTCTTCGACGTCGCTTCTGTACCGCGGACAACGGACAGTCTTGTTGAGATGTTGTAC
CCCATCGCGGAGCAGGAGGCGCTGGCCCTGGGGATCGACTTCTTTGTGCTCGGTAACACAGGCGTGTTCC
GCGGCAATATTATTTCGTCCGTGTCGATCAACGTGTGCTTGCGCCGCTTGGGCAGAGGGCTGCCGTGTCA
CGGCGTCCTCGCTGGGTACTACGAAATGCTGTACAAGGGTTACATGTGA
>AY064402 293, 1754 SRP54-like protein
ATGGTCTTGGCAGAGCTGGGTCAGAAAATCGGGCAGGCGATTCATCGCATGTCCGCCAAGTCGATGCTGG
GTGAGGACGATGTGAAGGAGCTGATGAACGAGATTGCGCGTGCGCTGCTGCAGGCAGACGTGAACGTCAC
CATTGTGAAGAAGCTTCAGGTGAGCATCAGAACGGAGCTGGCACTAGCGGAGGAGGGCGCTGGACTGAAC
AAGCGCAAGATCCTTCAGAACGCTGTCTTCAATGGCCTCAAGCGCATACTCGACCCCGGGGTGAAGCCGT
TCCTACCGGTGAAGGGGAGGCACAACGTCGTGATGTTTGTGGGTCTGCAAGGGTCCGGCAAGACGACATC
GTGCACGAAGTACGCCGCCTACTTTCAGCGCAAGGGCTTCAAGACGGCGCTGGTGTGCGCCGACACGTTC
CGCGCTGGCGCGTACGATCAGCTGCGTCAGAACGCGACCAAGGCCAAGGTACGCTTCTACGGATCACTGA
CGGAGGCAGACCCGGTAGCGATCGCCAAGGAAGGTGTGGCGGAACTCAAGAAGGAGAAGTACGACCTCAT
CATCGTAGACACGAGTGGCCGCCATAAGCAGGAGTCGGCGCTCTTCGAGGAGATGAAGCAGGTCGAGGAA
GCCGTGAAGCCGAACGACATAGTCTTCGTCATGTCCGCGACAGACGGCCAGGCGGTAGAGGAGCAAGCAC
GCAACTTCAAGGAAATGGTTGCTGTCGGCTCGGTCATTGTCACGAAGCTAGACTGCCAGACCAAGGGCGG

CGGTGCGCTCTCCGCCGTCGCGGCGACGCGCAGCCCCATCGTCTTCATCGGCACCGGCGAGCACTTCGAG
GACTTCGACCTCTTTAATCCGGAGCGGTTTGTGCAGAAGATGTTGGGCATGGGCGACATAGGCGGGCTGA
TGGACACGATGCGCGACGCCAACATCGACGGCAACGAGGAAGTCTACAAGCGCCTGCAGGACGGCCTGTT
CACAATGCGTGACATGTACGAACATCTGCAGAACGTGCTCAAGATGGGCTCCGTTGGTAAAATCATGGAA
ATGCTGCCCGGCATGTCGGGCCACGCCGCTACGGCGGGCCAGCAGGGCGACATTGCGCTGAAGGGGTTTA
TCCACATGCTAGACAGCATGACGGTCGCCGAGCTGGATGAAGTCAGGGTGAAGAAGATGATGACACCATC
CCGCATGCACCGCATCGCGCGCGGCAGCGGCCACTCCGTCGTCGAGGTGCAGAACCTGATTATTAGCTAT
ACGAAGTTTGAGGAGATCGTGAAGAAGATGGGCAAGATGAACTTCAAGGCCATGATGCAGGACTCTTCCG
GGCCCGCCGCGGGGCACATGGGGCAGCAGCAGATGGGTCAGCTGGCGAAGCTGCTCAACCCGAACATGCT
ACGGCAGATCGGCGGCATGGGTGGACTGCAGGGGATGATGAAGCAGCTGCAGCAGAGCATGG
>AY079097 1, 951 class I nuclease
ATGCCTGCGTTTGTCGGCCTCCGTCTGCCTCTCACAGTGCTGTGCCTCCTGGTACTCAGCTCTGCGCTGT
GCGTGACTGAGGTTCTCGGATGGGGCTGCGTGGGCCACATGCTCCTCGCCGAGATCGCGCACCGTCAGCT
GAACGATGAAAACAAGGAAAAGCTTGACGCGATGGCGCACGCATTTGCGCAGAGCGGCCCGTTCGAGTCG
TCCCCGGACATGGTGCAGGCCGCATGCTGGGCGGATGACGTGAAGCGTTGGGGTCAGTATGCCATGGCCA
CGTGGCACTTTTTCGCTACGCCGTACAACCCCGAAGACATCAATATCACCGATCCGGTCGCTACAGTCAA
TGCCGTCACGGTGTCCCGCAATATGGTAACATCGCTTCGACGTACGAATGCGCCGCTGCACCTGCTAAAC
TTCGCTTGGGTGAACCTGGTGCACATCCTTGGCGATCTGCACCAGCCCCTGCACACCATCTCCAGGTACT
CCTCTGAGTACCCGCACGGAGACAGAGGAGGCAATAAGGTTGAGGTTCGCGTACGCAAAAGAAAGGTGAA
CCTGCATGCCGCCTGGGATAATATTTGCAGTGGCACGCCTCCACGCTACAAGCGGCCCCTCTCTTACACC
GACCTCTTCGCCCTCGCTGCGACAGCGGACCGGCTGCTGGAAACGTACACCTTCCCAGAAGCGTTGCGGA
CACTCGTGGACGTGGTGGCGATTCACGAGGAGAGCCACATGTTTGCGGTAAACACCAGCTACCCCGGCGT
AACACCAGGTGCGACTCTCAGCGATGATTATCTTGCTCGATGCAAGCGTGTCGCTGAGGCTCGACTGACG
CTTGGTGGCTACCGCCTCGGCTACCTTCTGAATGAGCTGCTGTCGAGTATCCCTGTGGACGAGGCCACAC
TGGAGGCGTACCGCGCTGCACGTTGGAAGTGGAGCGCGTAA
>AY129954 190, 732 signal peptidase type I
ATGCGGGAACACATCAATACCCTACTCTCCCTCCGCGTCCGCGACGTCATCCAGCAGGTCGTCACCGTTG
GTCTTTTTCTCAGCATCGTCCTCGTTGGCTGGCGCGCCGTCGCCGTCGGCACGAACTGCGAGGCCAGCAT
CGTCGTCGTGCTGAGCGGCAGCATGGAGCCCGGTTACTACCGCGGCGACGTGCTGCTGCTGCACCACCGG
CCAGAGTACCCTGTGACGGTGGGCGACATCATCGTCTACACGCTGCCTGGACAAGAAATCCCAATCGTGC
ATCGCGTCCACCGCATCCACCAGCGCAGCGAGGACGGCAAAAAGTTTTACTTGACCAAGGGTGACAACAA
CGTGAACGACGACCGCTTTCTCTTTCGAAATGGACGGGAGTGGGTGGAGGAGGGGATGATTATTGGCAAG
ACCTACGCCTATGTGCCGCGCATTGGGTATCTGACAATTATGTTCAATGAGTCCAAGATCATCAAGTACC
TAGCGCTGGGGCTGATTGGCTTTTTTCTGCTGACCACCACAGATGAGATGTAG
>AY147013 1, 2796 POLIA
ATGCAAAACCAGAGTGCATCCAGCAGCGGCGCTCGAAGCAAGCCAACACCACTGAGGCGCGATTGTGCCG
GCACGAAAGCCGCTGCTGACGCCTCCGGTGCGAAACGAAAGCCGAAGACGAGGAAGCGGGGTGGGGGCGA
CGCAATCCTCCCCGGTGCCTTGGGCCCCTCTGTGTGTGCAACGCCGGCTCCTCGCACGCTTTTCTCTCTG
GCATCTTCCGAAGCTGGCGGGGGTGCGATGGCGGAGCTGCAGGGTGGACCCACCGCGGCGGCCACGGTCT
CTGTCCCCTACATCTATCTGCAGGAATCGGCCGAGGAGCTGTTCAGCGACGTGCACCGTGCCCTCAACGA
CCCGGATCGCATCCAAGCACCACCCCTGTTTGTCGGCGTTTCGGCCCTCAGTAGCAATGTCAACGCGGCG
GCGGCGCTCGCGAGTGTGGAGTCTCGCACGAACTTTACCTACTACAGCAGCGGCGCCAAGACGGCGAAGC
GCCGCGTGTCGTGCTGTTTCTCCTCCCTCGACCCCGTGCGTAGCCACCAGCACGTCGAGCTTGTTCTGAT
ACGGTGGAAAGACGACATGTACGCGCTGCCGGCACAGACCGTTGGCTTGGCGCTTCTGTGCCGCGTCCTC
ACAGAGCTACCCGGGGTGGAGCTCATTACCTTCAATGCGCAGGTGCTCCTGGTGGCGCTACTCGCTTACT

ACAAGGGCACATTGTGGTCGAACTGTGTCAGCGACGTGCGCGTGATGGCGTGGATGGCTCAGCTGCACGT
GGCAAGTACTCTGTCGAAAGCTCCGTCCGCCGTGCGCGCTTGGGCAGGGGGAGGCACCGCCGCTGCCGCG
GACGGGGCGAGTGACGCGGACACGAGTGTGCTCTACGACTACAATCAGCTGCTCTTGCACGTTTGTGGCG
GGAAGCTGCCGCCGACAGTGCAGCTGGAAAGCATGAGCAACGCAGGGTGCTTCTCGCACTCAACGATCAT
TTTTGCCGAAGGCATCGGGACTGCCGCCGCCGCCGCAGCAGCAGCAGCCCCGGCGCCGCCCCTGACGTCC
GCACAGTGCCAGCTAGCTCATCAAGTGTACTACTTGGCCACTGTGTACCGCAGTCTTTACGGTCTGCTGG
GCAGCAAAGGGCTCCTGCAAGCGTTCCTGCGGCAGGAGAAGCGGATTGCGCTGCTGCTGGCGCTTCTCAA
GTACAATGGCATGCGCGTGGATCTGCATGAGGTTCACCGCTACCAGAAGAGCTGCGAGGCGGAGATGGCA
CGGCAGCGCCAACTTGCCAACAAGCTCGTCCCGGAGCTTGGCGAGGACTTCAATATTCAGAGCCACGATC
AGTGTCGCAAGGCACTCTACGAGGTGCTTCAGCTGGGCAAATACTTGCTTAAATCCGGAAATGGCGAAGA
CGTCGGTGCCACCGGGCTCACAATCACAAAAGGGGGGCGGCTGTCGACGGCGGAGGACACACTGCGTGCC
CTCGCCCGGCATCACAAGTTTCCTGCGTGCTTGTTGCGCTATCGCAAGGTGTCGAAGCTGATGCAGACAT
ACATCGAAGGGATGATGAGCTACGCCGTGGTGCGCTCTAGCGCACAAGCAAGCCCAGCTCCTTGTCTCGG
CAGCGTTAACGCTGAGACGGGAACTAGTGAGAGCACGAAAGGGCGGCAGCAGGCGCACGGCGATGATGAG
GGCGCCAAGGCCGTGTCGCTGCTGTTTACAAGCGAAGCGGTGGACATGAACGACGACAGCCCGTTTGTGG
ATCGCCGGCTTCACACGAGCAGCAGCGGATACAGCGCTAGCGGCGCCGAGCCAATGCCAGCACGAAATCA
ACGGAGCAGCCGCGAGGAGGCAAGTGCGACGCCGGTGGGCGAGGTCATTCTTCCTGCAAGGGGGTACGCC
ACGCTGCACCCAAACTTTCTTCAGGAGGGCACCGACACTGGACGGCTATCCTGCGTTGAGCCGAATCTTC
AGAATCTTCCACGTAATGGCATCACAGCCGCCGTTGCAGGCAACGACGCCGAGGAGGACGGCGAGGAAGA
CCTCTTGGGCTTTCGGCGCTGCTTCGTCGCCACTGACGGATGCGTGCTCTTGTCCATTGACTACGAACAG
ATCGAGCTACGCGTACTAGCGCACCTGTGCGGCGACGCCGCGCTCGTAGAAGCGCTGACGACATCGGCAG
ATATTCACCGGGCGATCGCCGAGGTGGTCTTCAAGAAGAAGCCCGTAAGCACTGAGGAGCGCCGTCTGGC
CAAGCGCGTCGTATTTGGTTTGCTCTACGGCGCCGGCCCCAAGACGCTCGCGACGCACATGGGTGTTACG
GTTGATCGGGCGTTGCACATCACCTCGTTGCTGGCAAACGCCTTCCCCGGTATCGACGCGTATCAGCGCC
GCGTCATCGAGGAAGCCCGCACCAATGGGTTCGTGCGCACTCTCAGCGGTCGCCTGAGGTACCTGCCGGA
CATCCGCAGCACAGTGCTCTCGCGACGGTCGTACGCGGAGCGACAGGCCTTCAACAGCGTCGTGCAGGGC
AGCGCCGCAGACGTGATGAAGATGGCGATGCTTGCCGTGTCAAAGGAGGTGCTGCAGCGCTACGATCGCT
CCGACGTGTCCCTGCTTTCGCAGATCCACGACGAGATGGTCTTCATGGTGCGCAAGGAGCTGCTCCCGAT
GGTGGTGCCGCTGGTATCCTCAGCGATGTCGCACGCCATGCAGTTGCTCGTACCGCTGAGTGTAACGGCC
AAGTTTGGCGATTCGCTGGGTAACTTGCAGGAGTGGTCCGTGGAGCACGACCTAGGCGTGGTGTAG
>AY155573 241, 1674 pdi
ATGCAGCGCTCATTCCTTGTTTTTGTTCTGTGCGCCCTTCTCTTCTGCGTCGCGTCCGCAGAGGTGCAGG
TGGCCACTAAGGACAACTTTGACAAGGTCGTAATCGGGGATCTCACGTTGGTCAAGTTTTATGCTCCGTG
GTGCGGCCACTGCAAGACACTCGCCCCGGAGTTTGTAAAGGCCGCTGACATGCTGGCCGGCATCGCGACC
CTTGCAGAGGTCGATTGCACCAAAGAAGAGAGCCTTGCTGAGAAGTACGAAATCAAGGGGTTCCCCACGC
TGTACATCTTCCGTAACGGTGAGAAAGTGAAGATCTACGATGGTCCCCGCACTGCCGCCGGCATCGCGTC
GTACATGAAGGCGCATGTCGGTCCATCGATGAAGGCCATCTCAACGGCTGAAGAGCTGGAGGAGCTCAAG
AAGGAGACTTTCCCGGTGTGCGTGGTGAAGACAGCGAGCACCGACTCGGAGATGGCGTCGATGATAACCA
AGGTGGCGGACTCTCTCCGCTCGCAGATGAACTTTGTGCTCGTGACGGATGCGGCCATCTCTCCGAATGA
TGCCATGGAGTCGGTTACGGTGTATCGCAAGAATGCGGAGCGCGAGGCGTACACCGGCGCTACACCAATG
ACGGCAGAGTCGGTGAAGAGCTTTCTCACGAGTGCTGTGTTGGACTACTTTGGCGAGCTCGGCCAGGAGA
GCTTTCAGAAGTACATGGAAGCGAACAAGGATAAACCTCTTGGGTGGGTGTTCATCGACAAGAACACGGA
TTCTGCGTTGAAGGGGTCACTTGTGGCGGTGGCGGAGAAGTACCGCTCGCAGGTGTTGCTAACCTACATT
GACGGCGATCAGTACCGCCCCGTCTCGCGCCAGCTGGGCATTCCTGAGGATGCGAAGTTCCCGGCGTTTG
TGGTCGATTTCGAGCGCCGCCATCACGTGATGGGGACGGACACCCCAGTCACCTCCGAGTCTGTCGCTGC

GTTTGTGGAGAAGTATGTCAAGGGCGAGACGAAGCAGACCGTGATGTCCGACGCGATTCCCGCTAAGGAG
ACGGTGAACGGCCTCACAACGGTGGTGGGTCAGACTTTTGCGAAGTACACGGACGGCACACAAAACGTGA
TGCTGCTCTTCTACGCGCCGTGGTGCGGACACTGCAAGAAGCTGCACCCCGTCTACGATAAAGTAGCCAA
GAGCTTCGAGTCTGAGAATGTGATCATTGCGAAGATGGATGCCACGACGAACGACTTTGACCGCGAGAAG
TTTGAGGTGTCTGGATTTCCAACGATTTACTTCATCCCAGCCGGCAAGCCGCCAATCGTGTACGAGGGTG
GCCGCACCGCAGACGAAATCCAGGTGTTTGTGAAGTCTCACCTGACCGCCTCCGCCGCTCCATCTGGCGG
CCCTTCCGGCAACAGCGAAGAGGAAGATTTGTAG
>AY157268 1, 750 GPI12
ATGCACAGTATCACAGTTGTGTGCATCAGCGTGGTGCTTGCGCTGCTGCTGCTTCTGTGGATGAAGATAT
CAAATCTTGCCGGGACGTCCGTTCGCAGTGACGTTCTGTTTGTGTTTGCGCACCCGGATGACGAGGCAAT
GTTTTTCACGCCACTCCTGCATTCGCTGCGGACACAGCGGGTCACGGTGCATTTTCTGTGCCTGTCCAAC
GGCAACTACGCCGGGATGGGAAAGGAGCGTGAGAAGGAGCTGTACGCCAGTGGCGCTTTCTTTGGGGTGC
AGCGGCGTAACATCCGAGTCGTCGACCACGCCGACTTGCAAGACGGCATGTGTAATATCTGGAGCCCGTT
GCTTATTCGCAGGGAGATCGAGTCGTACATGCAGAAAGCCGGCAACATCAGCACCATTGTTACATTTGAC
AAGTACGGTGTCTCTGGTCATCCTAATCACATCGCCGTTCACAATGGCGTGCGGGAGCTCAAGGAGAACA
TGCCTCCAGGGATCCTCCACCTGCAGCTCAGAACACGGAGTCTGCTGTGGAAGTACGTTGGACTGCTGGC
TGTTCTCCCTTATGCCCTGTGGTCATCCACGTGCGTTAGTCGCGCCAGCTTTGTCGCCGTCATTCCACCC
GCATCAGTCTGGGAGAGCATGGCCGCAATGCGCAAGCACGCCGGTCAGCTTGTCTGGTTTCGGTATTTGT
TTGTCATTTTCTCTTCGTACACGTACGTGAATGAGATCGAAGAGCTCTAG
>AY168561 1, 822 6-phosphogluconate dehydrogenase
GACATCATAATCGACACTGGTAATGCGAACTTCAAGGACCAGGACAAGCGCGCGGCTCAGTTGGAGAGCC
AGGGTCTCCGCTTCCTCGGCATGGGCATCTCTGGTGGTGAGGAGGGTGCGCGCAAGGGGCCGGCCTTCTT
CCCGGGTGGCACACCAAGCGTGTGGGAGGAGGTGCGCCCGATCGTGGAGGCGGCTGCGGCCAAGGCTGAG
GACGGACGCCCGTGCGTGACTTTCAACGGCAAGGGCGGCGCTGGCTCCTGCGTGAAGATGTATCACAACG
CTGGGGAGTACGCCGTGCTGCAGATCTGGGGTGAGGCGTACAGCGCCCTGCTTGCCTTCGGCTTCGACAA
CGATCAGATCGCCGACGTATTCGAGTCGTGGAAGGCGGATGGCTTCCTTAAATCCTACATGCTCGACATC
TCCATTGCCGCTTGCCGAGCGAGGGAGGCAACAGGCAACTATTTGTCAGAGAAGGTCAAGGACCGCATCG
GCTCCAAGGGCACCGGCCTCTGGTCTGCCCAGGAGGCTCTGGGGATTGGTGTGCCAGCGCCCTCGCTCAA
CATGGCCGTCATCTCACGCCAGATGGCCATGTACAAAGGGGAGCGCATCGCCAACTGCAAGGCGTTTCCT
AACTTCCCTCGTGGCCCTTCTGAGGAAGCCACGGACAAGTCCCCGAACTCCCCCGGAGCGAAGAAGCTGT
ACCACGCCGTCAGCCTTTGTATCATCGCGAGCTACGCGCAGATGTTCCAGTGCTTGCGCGAGCTGGACAA
GGTGTATGGATTCGGCCTGAACCTGCCCGCCACGATCGCCACCTTCCGCGCC
>AY185122 27, 182 A2
ATGAAGATCTGCAGCGTGCGTCCGCTTGTGGTGTTGCTGGTGTGCGTCGCCGCGATGCTCGCACTCAGCG
CCTCCGCTGAGCCGCACAAGGCAGCCGTTGACGTCGGAACGCTCTCTGTGGGCCTGCAGGCCGTTGACGT
TGGCCCGGTGTCGTAA
>AY196150_0 969, 3409 40S ribosomal protein S5A
ATGAGCAGCAAGACTCCCAAGCTCTTCAACAAGTGGTCGTTCGAGGGCCTCGAGACGTCGGAGCTGGCCC
TCCGCGACCACATCTCGACGACGGCCGCGTATGTGCCGCACACGTCCGGCCGCTGGCAGAAGCGCCGCTT
CTACAAGGCCCGCATGCCGATTGTCGAGCGTCTGGCCAACGGCCTGATGTTCAAGGGCCGCGGCAACGGT
CGCAAGCTGCAGGCTGCGCGTCTGCTGAAGCACACACTGGAAATCATCCACCTGCTGACGGACGAGAACC
CCCTGCAGGTTGTTGTGGACGCCGTGTCGAAGGGGGCGCCGCGTGAGGACTCGACCCGCGTCGGCTCCGG
TGGTGTTGTGCGCCGCCAGGCTGTGGATGTGTCACCGATGCGCCGCGTGAATGAGGCGATCTACCAGATG
TGCAAGGGCGCGCGTGAGGCTGCCTTCCGCAACCTGAAGTCGATGCCGGAGTGCCTCGCGGACGAGATCG
TGAACGCGTCCAAGGGTAGCTCGAACTCCTACGCCATCAAGAAGAAGGATGAGGTGGAGCGCGTGGCCAA

GGCTAACCGGTAA
>AY196150_1 2837, 3409 40S ribosomal protein S5B
ATGAGCAGCAAGACTCCCAAGCTCTTCAACAAGTGGTCGTTCGAGGGCCTCGAGACGTCGGAGCTGGCCC
TCCGCGACCACATCTCGACGACGGCCGCGTATGTGCCGCACACGTCCGGCCGCTGGCAGAAGCGCCGCTT
CTACAAGGCCCGCATGCCGATTGTCGAGCGTCTGGCCAACGGCCTGATGTTCAAGGGCCGCGGCAACGGT
CGCAAGCTGCAGGCTGCGCGTCTGCTGAAGCACACACTGGAAATCATCCACCTGCTGACGGACGAGAACC
CCCTGCAGGTTGTTGTGGACGCCGTGTCGAAGGGGGCGCCGCGTGAGGACTCGACCCGCGTCGGCTCCGG
TGGTGTTGTGCGCCGCCAGGCTGTGGATGTGTCACCGATGCGCCGCGTGAATGAGGCGATCTACCAGATG
TGCAAGGGCGCGCGTGAGGCTGCCTTCCGCAACCTGAAGTCGATGCCGGAGTGCCTCGCGGACGAGATCG
TGAACGCGTCCAAGGGTAGCTCGAACTCCTACGCCATCAAGAAGAAGGATGAGGTGGAGCGCGTGGCCAA
GGCTAACCGGTAA
>AY197729 1, 402 ribosomal protein L27
ATGAAGTTCCTCAAGCCTGGCAAGGTGGTGATCGTGACGTCCGGCCGCTACGCCGGCAAGAAGGCGGTGA
TTGTGCAGAACACCGATTCCAAGAGCAAAGAGCGTCCCTACGGCCACTCTCTGATCGCCGGCATGAAGAA
GTACCCGAAGAAGGTTGTCCGCGGCATGAGCAAGCGCACCATCCAGCGCCGTTCTCGCGTGGGCGTGTTC
CTGCGTGTCGTGAATCACAAGCACTTTCTACCGACGCGGTACAACATGGACTTGTCGAGGGAGCTCCGCG
GCCGCATCAACGTCTCGGATGCGTCGAAGAAGGCCAGGTCGAAGCAGCTGGTGAGGAAGTTGTTCCAGGC
TCGCTACAACGCCGGCAGCAACCGCTGGTTCTTCCAGCGTCTGCGCTTCTAG
>AY227807_0 545, 1824 YIP1
ATGCTCAACGAGGTGCACGTGAACGCAGACCCGAACAGCATCTCCACCTTGGATGAGCCGGTGCTTGAGA
CGCTGCTGCGCGATGCCAAGGCGATCGGCCGCAAGCTCGTCGTGGTTGTGTGCCCCTCTCTAGGAGGTGA
TAAGGAGCTTCGCGATTGGGATCTCTGGGGGCCGCTGTTCCTGTGTCTCATTCTGGCCTCCATCCTCACC
ATCAACGCCAGCGACGACCAAGGCGCCGCCGTCTTCTCTGCGGTCTTCATCTTTGTGTGGCTTGGCGGTC
TTGTCGTCACCGTCAATGCCAAGCTCCTTGGCAGCAAGATCATGTTCTTCCAAACGTACTGCGCCATGGG
CTACTGCTTGGCGCCCATCTGTCTCGGCGCTCTCCTGTGCTGCGTCCTTCCGTGGTTTTTATTGAACCTC
ATGCTGTGCTTCATGGCGTGGGCGTGGGCCTGTTGGGCGGCCTTGCGCTTTTTCCGACACACCGTCAGCG
CCGACCGTGAGGTGTTGGTTGTGTACCCTGTCGGGCTCTTCTACGTCTTCTTCACCTGGATGGTTCTGGT
TGGTATTTAG
>AY227807_1 1465, 1824 terbinafine resistance locus ORF3
ATGTGCGTCTGTGTGTGTGTGCTCTCTCCTATCGCTCTGCCGTCCTTTGTTTTTTTTTTGTTTGTGTGTG
CTCGTATCTCTCTCTCTGTGCAATCCCTGATGACGTGGGACACCTCAGTGCGTCGCTTCGCAGGGTGCCG
TGATGCCCCCGCCATCGGCACCCATACTGGTCTACCTATGGTGGTCAGAGGCTCGAGCGCCTACGACGTG
GGGAGGTCAGTGGGATTCATCGCTGCTGATGTCGGTGGTGCTTCGGTGCGACCTGCGACAGCGAGCACAC
GCTTGTGCCATCCATGTGATGGGCGAAGTGTCCGCTTGACTCAAGCGTATCCTACCCCCGGCCCTCACAC
TGCGTGCTGA
>AY230143 264, 2762 SCA1
ATGAGGGGGGACATCACAGCGTCGTTCGGCCTCACAGCAAGGACCTTCGAAACCCACTCATGTGCCGAGG
GGCGAAACCAGACGGGTACGCAAGCGACACGGCTGTCGCACGCACATAGAAGGCCCTTTTTTCCTCGGCG
TCACCACTGCTGTGCCATACTCGTCAGCTGCATCACAATTATGTACTTGCTCAGCACGCTGCACTTCCTC
GTCATGGAACCGGACGTAAGCCGTGAGAATGCCGCCGTTTCAGGCAACGAGACGAGCACCAACATATCCT
CGCCCGGGATAAGGCTCCTGACGAATGAGCTCGAGCTGCCACCCATCCGCGTGATGGCTGCCGATCACAC
ATGCGCCATGTGCACGAACGGTGTGGTGTATACTCAAACTGGCGAAGAGGGCGGTCCCGCTGCGGCGGCG
AGCAGGCCTGAGAACGTGACTGCTATGGTGTGCCGCAAGGTTTCAAAGGAGGAGGTCGATGTGCTGACAT
CCGCCGAGGAGTGCGTGGCGCGGCTGCACCGCCTCGGAATGCTGCATGCGGTCGCTGCTCGCGAGGAGGA
CGTGAACGCGTCGTGCGTGCGTGTGATGGTGCCCGCGATTGTGGGCCGCATGAGCGGTGGCAGCGACTCA

TGCCCCGCGTTCCTGGCTGAGATACGCCCGGGGATCGATAAACATGCGAAGTTGCAGGTGTGGTTTCCGC
GCATGCCGTCGTGGCCGCTGCCGGGGAGCCGCGCGCTGCCGTACGTCGGGACTGCGGCTGTGCGTGACAC
GGACATTGACCGCGACGTTGCGCCGCCCGTGTCGGTAGAGATAACGAGCGACGGCAGCACGATCGTGCTA
CAGTTTGGCCGTGATGCGACGTCGACGGTGCCGGAGGCTTTCGAGCTGCGGTGCCCGACGCACTTCTTCT
CCGTCTTGGTTGGGCGCTGGGGCCGCCAGCACAACCAGCTGCAGGAGCTCCTGCACAGCCTTGCCTTCGC
TCGCGCGTCGAACCGCACGTATGTGCTGCCATCCTTTACGCCGGATACGAATTTGCCGTATGTGCGCTAC
AGTGCGTCGAATCTGTACGGGTTCAACGCGATCCGGCGCGAGGGGCAGTACTGCCTCGTCACGAACGCTG
AGGTGAAGCCGATCTTCGCGCAGCTGCAGGCGATGGGCGTTGTGATGGACATGCGCCGCGTGGACTACAT
GGGCGTCGTGAAACCGCATCTTTCCCCCCAACAGATGGCGCAGGAGATAGTCCGCCGGTGGTGGGTGCTG
CCACCGGTGCGCGACGGCGTGCGCAAGTACAACCTCGAGAGCTGGTTTGCCGACTCGAGGTCATTCTCGC
CCCCGCTGCGCTCCGTGGCGGACTACCGCGCTGACTCGGTGCTGTTCCGCGAGTCTGTGGAGCCGCTGAT
GGGGGATGATTTGTTTCGCACCCCTACTGAGGCTGAGCAGTACAGCAGCTGGGACCGCATCAAGATGTTC
GCTTCGCGCATGCTTGAGGTGCTTGGCGGCGACCACGTGGGCGACGACGCGGCAGGTGGGGCCGCGCAGG
TGCGGATGGCCGTGATATCCGGCAAGGTGGCGTATTTCTTTCACCCGCAACTAGAGGAGGTGACGCGGCT
GTTCGGGCTGCTGCGCCCGTCCGCACATGTGTCTGCGGAGGTGGATCGTATGTACTCGAAGAGTGCGGCA
GCGCTTGGGTGGCCGACGACTGGTTCGACCAAGGACGCTGACGGGCAGCTTGTGATGCGCGACACGCTAC
GGCTTGCAAACTTCAAGGGCGTGATCGGGATTCATGCGCGCCTTCGTGAGCGTACGTGTCGTGCCGAGGC
CATGCGCCTCTGGGACACAAACCTCGCGCTGACGGTCGACAGGTACACCGTCGACAGCACGGACCCGAAC
TGGGCTGGCGTGAAATCGATGGTGTCAAGCATCGAGAGCGACTGCTCGTGGACTGCGGAGTCTGCGGTGG
AGCTGTACCTGCAGTACGCGCACTGGCTTAGGTCAACCATGCCGGGCACGTTTGGCCACGATTACGTGCC
GACGACCTATGTTGCGTACGACGACCAGAGTGGGCCGATGGCAGACGACATGGAGAGGATCATACAGCTG
CTTCCCGAGGAGGTGGTCGTGAAGGACATCGGCAACCTAACCGCGCAGTACCCGCGGCGTTTTGTGGCGT
CGTACGACGGCCGCATCAAAACTAGTCTTAAAGCTGTTTACAATCGTGCGCGGGCGAAGGCGGTAGAACA
GCTGGAGGTGCACGCAAGCTCGCCGAATTACTACGCGCTGCTGCAGAAGGCTTTGCTGAACATGTTATAC
GAGCCTGGTTTGAGGGAGATGCAGGCCATGTCGCTCGACTACATCGTGCTCACGAACACGGAGGTGTTCC
GCGGCAACATCATCTCGTCTGTGGCGACGAACGTGATGCTGCGCCGTTGGGGCCGTGGACTCCCCGCTCA
TGGTTTACTGGCTGGGTACCTCGAGTCGTTTTACACGGGCTTATACTGA
>AY230144 750, 3194 SCG1
ATGCAAGAGGAGAACAATGTGCCAGCGGAGTGGGCTCCCAGGGACACGCACCGCCTCGACCTTCTTCAGC
TGCTTCCCCCCTCTCAGAGGCACTGCGGGCAGAGCAGCGCTGCGGAGAGCGGACAGGGCTGTCACTCGCG
CTCACGCGGCAAGCCGGAGGTGCACTCGACTCGAGCGTCGAGCGGTGGTAAGAGGAGCACCGGCACACTG
TCGAGAAGCGATGACTGCTTGAATGCCATAGCACAGACCCCCCTGTTCAACTCCTGCGGTCATCCTCGAC
GATCCGCGGCACAATGTCTGCGTTTACACACTCGACAGCGTCGTCTGGTTGCGCTAGCCGTCTTACTTGC
TCTCATCGTCATGGTTCACTGGGTCATCAGCCAATTCACGACCAATGTAGGCTCACAACAGAAATCATCG
ATAGAGTTGCTCAATGAAGCGAACCGTGAATATGAGTCTTCATCGCCGTTCTCTGTCCTGGTGCAGCACG
AGAGTGCTGTTGAGCGGTTGACGACCACACAGCGTGGTCTTGCGGCCTCTGGCGCTGGCTCTCTGGCGAG
ACTCGACCGGGATCAGCTGCCGTCGTGGACGTTGCGTGTGATCGACGAGGACTGCACGATGTGCTTGGAC
AATGTCACATACGCTTCTGCGGTCGCTGCGGATGCGGGGAGGTCGACTAACAAGACGGGACATCATAAGC
AAAAAGGGCTTTCCGTGTTCGCGACGACATCTGCACCGCTGGGCCTCATGGGGCCGATGCTGTTTGCGAT
GGCGAGCGTGACGGACGACGTGGACGTTGCAGCGGAACTGCCGCGGTGGCAATGGGTGACCTGGTCGTAC
GCGCAGCAGCGCCGCTTCGTTCACACATCGCAGTCGCGGAGCACAGGTGAGCGACCGCGACACTTGATCG
TGTTGGGCATACCTTCAACGGACCAACCGATGCGCTACCCTCTGCGGGACGCACAGCGGGCGACGTGGCT
GACGTACCGAGAGGTTGCACGCACCGAGAACAACTTTACTGGCGCGCTGCTGCAGCTCTACGTGTTCGCT
GCTGCGGAGCATGATTCTGAGGACTCGACGCATTCCGCCGTGGACATGGTTCAGCTGGCCCCGACAGTGA
GCGAGTACGCCGCAGCAACTGAGCAGCACCTGGCTGTGGAGGGTGGAGACGTCAACAATGCACCCCCCTA

CCTGCAGCGTCGCGTGGTGCTGCGTGATGGGTGGCGCGACATCCCACGAAGCGATGACGCGGTGTGGAGA
TCACCCTGCGCTGGTGTAAGGACCACCGTGGTGACTGCGGCAAGTCTTGTGGCCGGGACCTCGTCCCTCG
CGGCTCTTTCAGCCCAGTTGTCGCTGCCCGTGACACCTGCCTTCACAGCAGCGGCGCAGTACGTGTGCCA
CGCGTCCGCGGCACTGTGGCAGGAGGCGCTGCACCACCGCAACTCGCTATGGCTGGACTTTTTGACGGAC
CGCAAGCCGACCACGAAAAAGAAAATGGGCATGGGTATATCGTGGGGTATACCGACAGAAATAGGCATGT
CTCAAAAGACCGTGTTGTGGCTGAACTACGCGTACACTGCCTTCCCGGACGTGCCGTACATCATGAAGGG
CGACGACGACATGTACCTGAAGGTGCCACAGTACTTGAGCGACCTGCGGCATGTGCGCGGTGGGTGGCAG
AAGCCGCGCAACTTGACGGTGACGATTCCACACAGAGGGGTTATCCCACCGACACTGGCCATCGACGACG
CGGAGGAATGCCTGTATCGAGTGTGGTGGATCTATAGCAATAAAATCGCGTATGGCAACGGCGTGGGCTA
CATTCTGGACCGTCGCCTCATCCAGGCTGCACTGAACCCATTTGATGGCTCCAATGCTCTTCTGATGAAG
CTGCTGACAAATCCCTACGATTACAGGCTGGAAAAGGAATATCTTAGCTTGAACATGCAGCACGAGGATA
AGCATATCGGGAAGCAGATAAGAGATCACCACGACGCCGTGGAGAGGTTCTGCCCCAAGAAGCGCGTGTG
CTACATGGCTGACCGGCGGTTCCGGGCGCACCAGATTCTGCGGCCAAAGCCTGTCAAGCTGACGTGGATC
TCGGTGATGGCGCACTTTGGCATGTCAGCGATTCCATACTATGTCCACTACTTCCACAAAAATGAGTTCA
AGGTGGCCGAGGAGGCGAAGCGGTTGATTGCGCAGGGCATCGACGTGAACACGATTGAGGAGAATGCAAC
GCAGCGGATGCACGAGTGGGTGGCGTCTCAGGTGCCGAGCACACTCGTGGGGCTCGGAGCGTCATTCGAT
CTCGACTGGGTACGTGGAGACCCACGCACTGCGTACACTGTAGCCGAGGAGGACGAAGTCGCGGTATACG
ATGTTCGGTACAAGCTCCGCCGAAGGAACGATATCCCGTGTGTCTTGGAGTCTGATAAGAGGTAG
>AY230145_0 385, 4959 divergent H4 histone
ATGGCCAAGGGCAACCAGAGGCGCCAGAAGAAGGTGCTGCCGACAACATCCGCGGCATCACTCGCGGCGG
CGTGAAGCGCATCTCGAGCGAGGTGTACGAAGAGGTGCGCCGCGTGCTGAAGGCCTACGTGGAGGACATT
GTGCGCTGCAGCACGGCCTACACCGAGTACGCGCGCAAGAAGACCGTGACGGCGTGCGATGTTGTGACCG
CGCTGCGCAAGCAAGGCCACATCCTGTACGGCTACGCGTAA
>AY230145_1 2515, 4959 SCG2
ATGCGAGAGGAGAACAATGTGCCAGCGGAGTGGGCTCCCAGGGACACGCACCGCCTCGACCCTCTTCAGC
TGCTTCCCCCCTCTCAGAGGCACTGCGGGCAGAGCAGCGCTGCGGAGAGCGGAGAGGGCTGTCACTCGCG
CTCACGCGGCAAGCCGGAGGTGCACTCGACTCGAGCGTCGAGCGGTGGTAAGAGGAGCACCGGCACACTG
CCGAGAAGCGATGACTGCTTGAATGCCGTAGCACAGACCCCCCTGTTCAACTCCTGCGGTCATCCTCGAC
GATCCGCGGCACAATGTCTGCGTTTATGCACTCGACAGCGTCGTCTGGCTGCGCTAGCCGTCTTACTTGC
TCTCATCGTCATGGTTCACTGGGTCATCAGCCAATTCACGACCAATGTAGGCTTACAACAGAAATCATCG
ATAGAGTTGCTCAATGAAGCGAACCGTGAATATGAGTCTTCATCGCCGTTCTCTGTCCTGGTGCAGCACG
AGAGTGCTGTTGAGCGGTTGACGACCACACAGCGTGGTCTTGCGGCCTCTGGCGCTGGCTCTCTGGCGAG
ACTCGACCGGGATCAGCTGCCGTCATGGACGTTACGTGTGATCGACGAGGACTGCACGATGTGCTTCGAC
AATGTCACATACGCTTCTGCGGTCGCTGCGGATGCGGGGAGATCGACTAACAAGACGGGACATCATAAGC
AAAAAGGGCTTTCCGTGTTCGCGACGACATCTGCACCGCTGGGCCTCATGGGGCCGATGCTGTTTGCGAT
GGCGAGCGTGACGGACGACGTGGACGTTGCAGCGGAACTGCCGCGGTGGCAATGGGTGACCTGGTCGTAC
GCGCAGCAGCGCCGCTTCGTTCACACATCGCAGTCGCGCAGCACAGGTGAGCGACCGAGACACTTGATCG
TGTTGGGCATACCTTCAACGGACCAACCGATGCGCTACCCTCTGCGGGACGCACAGCGGGCGACGTGGCT
GACGTACCGAGAGGTTGCACGCACCGAGAACAACTTTACTGGCGCGCTGCTGCAGCTCTACGTGTTCGCT
GCTGCACACCGAGATTCTGACTACACAGCGCAGTACCTCGGGGACACGGCGCAGGTGGCCCCGACAGTGA
GCGAGTACGCCGCAGCAACTGAGCAGCACTTGGCTGTGGAGGGTGGAGACGTCAACAATGCACCCCCCTA
CCTGCAGTGTCGCGTGGTGCTGCGTGATGGGTGGCGCGACATCCCACGAAGCGATGACGCGGTGTGGAGA
TCACCCTGCGCTGGTGTAAGGACCACCGTGGTGACTGCGGCAAGTCTTGTGGCCGGGACCTCGTCCCTCA
CGGTCTTTTCAGCCCAGTTGTCGCTGCCCGTGACACCTGCCTTCACAGCAGCGGCGCAGTACGTGTGCCA
CGCGTCCGCGGCACTGTGGCAGGAGGCGCTGCACCATCGCAACTCGCTATGGCTGGACTTTTTGACGGAC

CGCAAGCCGACCACGAAAAAGAAAATGGGCATGGGTATATCGTGGGGTATACCGACGGAAGTAGGCATGT
CTCAAAAGACCGTGTTGTGGCTGAACTACGCGTACACTGCCTTCCCGGACGTGCCGTACATCATGAAGGG
CGACGACGACATGTACCTGAAGGTGCCACAGTACTTGAGCGACCTGCGGCATGTGCGCGGTGGGTGGCAG
AAGCCGCGCAACTTGACGGTGACGATTCCACACAGAGGGGTTATCCCACCGACACTGGCCATCAACGACG
CGGAGGAATGCCTGTATCGAGTGTGGTGGCTCTATAGAAGTAAACTCGCGTATGGCAACGGCGTGGGCTA
CATTCTGGACCGTCGCCTCATCCAGGCTGCACTGAACCCGTTTGATGACTTCAATGCTCTTCTGATGAAG
CTGCTGACAAATCCCTACGATTACAGGCTGGAAAAGGAATATCTTAGCTTGAACATGCAGCACGAGGATA
AGCATATCGGGAAGCAGATAAGAGATCACCACGACGCCGTGGAGAGGTTCTGCCCCAAGAAGCGCGTGTG
CTACATGGCTGACCGGCGGTTCCGGGCGCACCAGATTCTGCGGCCAAAGCCTGTCAAGCTGACGTGGATC
TCGGTGATGGCGCACTTTGGCATGTCAGCGATTCCATACTATGTCCACTACTTCCACAAAAATGAGTTCA
AGGTGGCCGAGGAGGCCAAGCGGTTGATTGCGCAGGGCATCGACGTGAACACGATTGAGGAGAATGCAAC
GCAGCGAATGCACGAGTGGGTGGCGTCTCAGGTGCCGAGCACACTCGTGGGGCTCGGAGCGTCATTCGAT
CTCGACTGGGTACGCGGAGACCCACGCACTGCGTACACTGTAGCCGAGGAGGACGAAGTCGCGGTATACG
ATGTTCGGTACAAGCTCCGCCGAAGGAACGATATCGCGTGTGTCTTGGAGTCTGATAAGAGGTAG
>AY230146 303, 2747 SCG3
ATGCAAGAGGAGAACAATGTGCCAGCGGAGTGGGCTCCCAGGGACACGCACCGCCTCGACCCTCTTCAGC
TGCTTCCCTCCTCTCAGAGGCACTGCGGGCGGAGCAGCGCTGCGGGGAGCGGACAGGGCTGTCACTCGCG
CTCACGCGGCAAGCCGGAGGTGCACTCGACTCGAGCGTCGAGCGGTGGTAAGAGGGGCGCCGGCACACTG
CCGAGAAGCGATGACTGCTTGAATGACGTAGCACGGACCCCCCTGTTCAGGTTTTGCGGTCATCCTTGGC
GACCCGCGGCACACTGTCTGTGTTTACACACTCGACAGCGTCGTCTGGTTGCGCTAGCCGTCTTACTTGC
TCTCATCGTCATGGTTCACTGGGTCATCATCGAATTTACGACCAATATAGACGCACAACAGAAACCATCG
ATAGAGCTGCTCAATGAAGCGGACCGTGAATATGAGTCTTCATCGCCGTTCTCTGTCCTGGTGCAGCACG
AGAGTGCTGTTGAGCGGTTGACGACCACACAGCGTGGTCTTGCGGCCTCTGGCGCTGGCTCTCTGGCGAG
ACTCGAGCAGGATCAGCTGCCGTCGTGGACGTTGCGTGTGATCGACGAAGACTGCACGATGTGCTTCGAC
AATGTCACATACGCTTCTGCGGTCGCTGCGGATGCGGGGAGATCGACTAACAAGACGGGACATCATAAGC
AAAAAGGGCTTTCCGTGTTCGCGACGACATCTGCACCGCTGGGCCTCATGGGGCCGATGCTGTTTGCGAT
GGCGAGCGTGACGGACGACGTGGACGTTGCAGCGGAACTGCCGCGGTGGCAATGGGTGACCTGGTCGTAC
GCGCAGCAGCGCCGCTTCGTTCACGCATCGCAGTCGCGCAGCACAGGTGAGCGACCGCGACACTTGATCG
TGGTGGGCATACCTTCAACGGACCAGCCGATGCGCTACCCTCTGCGGGACGCGCAGCGGGCGACGTGGCT
GACGTACCGAGAGGTTGCACGCACCGAGAACAACTTTACTGGCGCGCTGCTGCAGCTCTACGTGTTCGCT
GCTGCGGAGCATGATTCTGAGGACTCGACGCATTCCACCGTGGACATGGTTCAGCTGGCCCCGACAGTGA
GCGAGTACGCCGCAGCAACTGAGCAGCACCTGGCTGTGGAGGGTGGAGACGTCAACAATGCACCCCCCTA
CCTGCAGTGTCGCGTGGTGCTGCGTGATGGGTGGCGCGACATCCCACGAAGCGATGACGCGGTGTGGAGA
TCACCCTGCGCTGGTGTAAGGACCACCGTGGTGACTGCGGCAAGTCTTGTGGCCGGGACCTCGTCCCTCA
CGGCTCTTTCAGCCCAGTTGTCGCTGCCCGTGACACCTGCCTTCACAGCAGCGGCGCAGTACGTGTGCCA
CGCGTCCGCGGCACTGTGGCAGGAGGCGCTGCACCACCGCAACTTGCTATGGCTGGACTTTTTGACGGAC
CGCAAGCCGACCACCGAAAAGCAAATGGGCATGCGTATGTCGTGGGGTATACCGACGGAAGTAGGCATGT
CTCAAAAGACCGTGTTATGGCTGAACTACGCGTACACTGCCTTCCCGGACGTGCCGTACATCATGAAGGG
CGACGACGACATGTACCTGAAGGTGCCACAGTACTTGAGCGACCTGCGGCATGTGCGCGGTGGGTGGCAG
AAGCCGCGCAACTTGACGGTGACGATTCCACACAGAGGGGTTATCCCACCGACACTGGCCATCGACGACG
CGGAGGAATGCCTGTATCGAGTGTGGTGGCTCTATAGAAGTAAACTCGCGTATGGCAACGGCGCGGGCTA
TGCACTAGACCGTCGTCTCATCCAGGCTGCACTGAACCCGTTTGATGGCTCCAATGCTCTTCTGATGAAG
CTGCTGACAAATCCCTACGATTACAGGCTGGAAAAGGAATATCTTAGCTTGAACATGCAGCACGAGGATA
AGCATATCGGGACGCAGATAAGAGATCACCACGACGCCGTGGAGAGGTTCTGCCCCAAGAAGCGCGTGTG
CTACATGGCTGACCGGCGGTTCCGGGCGCACCAGATTCTGCGGCCAACGCCTGTCAAGCTGACGTGGATC

TCGGTGATGGCGCACTTTGGCATGTCAGCGATTCCATACTATGTCCACTACTTCCACAAAAATGAGTTCA
AGGTGGCCGAGGAGGCCAAGCGGTTGATTGCGCAGGGCATCGACGTGAACACGATTGAGGAGAATGCAAC
GCAGCGGATGCACGAGTGGGTGGCGTCTCAGGTGCCGAGCACACTCGTGGGGCTCGGAGCGTCATTCGAT
CTCGACTGGGTACGCGGAGACCCACGCACTGCGTACACTGTAGCCGAGGGGGACGAAGTCGCGGTATACG
ATGTTCGGTACAAGTTCCGCCGAAGGGACGATGTCGCGTGTGTCTTGGAGTCTGGTAAGAGGTAG
>AY230147 218, 2758 SCG4
ATGCAGCCAAGCGTGTGCTTGTGCCACTTTGCCGGCCACCTGCACACCTTAGGCACTCCTTCCGCCACCC
CCGCACTGCGCGGGAAGTACACCATCATGCGAGAGGAGAACAATGTGCCAGCGGAGTGGGCTCCCAGGGA
CACGCACCGCCTCGACCCTCTTCAGCTGCTTCCCCCCTCTCAGAGGCACTGCGGGCAGAGCAGCGCTGCG
GAGAGCGGAGAGGGCTGTCACTCGCGCTCACGCGGCAAGCCGGAGGTGCACTCGACTCGAGCGTCGAGCG
GTGGTAAGAGGAGCACCGGCACACTGCCGAGAAGCGATGACTGCTTGAATGCCGTAGCACGGACCCCCCT
GTTCAACTCCTGCGGTCATCCTCGACGATCCGCGGCACAATGTCTGCGTTTATGCACTCGACAGCGTCGT
CTGGCTGCGCTAGCCGTCTTACTTGCTCTCATCGTCATGGTTCACTGGGTCATCAGCCAATTCACGACCA
ATGTAGGCTTACAACAGAAATCATCGATAGAGTTGCTCAATGAAGCGAACCGTGAATATGAGTCTTCATC
GCCGTTCTCTGTCCTGGTGCAGCACGAGCGTGCTGTTGAGCGGTTGACGACCGCACAGCGTGGTCTTGCG
GCCTCTGGCGCTGGCTCTCTGGCGAGACTCGACCGGGATCAGCTGCCGTCGTGGACGTTGCGTGTGATCG
ACGAGGACTGCACGATGTGCTTCGACAATGTCACATACGCTTCTGCGGTCGCTGCGGATGCGGGGAGATC
GACTAACAAGACGGGACATCATAAGCAAAAAGGGCTTTCCGTGTTCGCGACGACATCTGCACCGCTGGGC
CTCATGGGGCCGATGCTGTTTGCGATGGCGAGCGTGACGGACGACGTGGACGTTGCAGCGGAACTGCCGC
GGTGGCAATGGGTGACCTGGTCGTACGCGCAGCAGCGCCGCTTCGTTCACGCATCGCAGTCGCGGAGCAC
AGGTGAGCGACCGCGACACTTGATCGTGTTGGGCATACCTTCAACGGACCAACCGATGCGCTACCCTCTG
CGGGACGCGCAGCGGGCGACGTGGCTGACGTACCGAGAGGTTGCACGCACCGAGAACAACTTTACTGGCG
CGCTGCTGCAGCTCTACGTGTTCGCTGCTGCGGAGCATGATTCTGAGGACTCGACGCATTCCGCCGTGGA
CATGGTTCAGCTGGCCCCGACAGTGAGCGAGTACGCCGCAGCAACTGAGCAGCACCTGGCTGTGGAGGCT
GGAGACGTCAACAATGCACCCCCCTACCTGCAGCGTCGCGTGGTGCTGCGTGATGGGTGGCGCGACATCC
CACGAAGTGATGACGCGGTGTGGAGATCACCCTGCGCTGGTGTAAGGACCACCGTGGTGACTGCGGCAAG
TCTTGTGGCCGGGACCTCGTCCCTCGCGGCTCTTTCAGCCCAGTTGTCGCTGCCCGTGACACCTGCCTTC
ACAGCAGCGGCGCAGTACGTGTGCCATGTGTCCGCGGCACTGTGGCAGGAGGCGCTGCACCACCGCAACT
CGCTATGGCTGGACTTTTTGACGGACCGCAAGCCGACCACGAAAAAGAAAATGGGCATGAGTATATCGTG
GGGTATACCGACGGAAGTAGGCATGTCTCAAAAGACCGTGTTATGGCTGAACTACGCGTACACTGCCTTC
CCGGACGTGCCGTACATCATGAAGGGCGACGACGACATGTACCTGAAGGTGCCACAGTACTTGAGCGACC
TGCGGCATGTGCGCGGTGGGTGGCAGAAGCCGCGCAACTTGACGGTGACGATTCCACACAGAGGGGTTAT
CCCACCGACACTGGCCATCGACGACGCGGAGGAATGCCTGTATCGAGTGTGGTGGACCTATAGCAAAAAA
ATCGCGTATGGCAACGGCGTGGGCTACATTCTGGACCGTCGCCTCATCCAGGCTGCACTGAACCCGTTTG
ATGGCTTCAATGCTCTTCTGATGAAGCTGCTGACAAATCCCTACGATTACAGGCTGGAAAAGGAATATCT
TAGCTTGAACATGCAGTACGAGGATATGCATATCGGGAAGCAGATAAGAGATCACCACGACGCCGTGGAG
AGGTTCTGCCCCAAGAAGCGCGTGTGCTACATGGCTGACCGGCGGTTCCGGGCGCACCAGATTCTGCGGC
CAAAGCCTGTCAAGCTGACGTGGATCTCGGTGATGGCGCACTTTGGCATGCCAGCGATTCCATACTATGT
CCACTACTTCCACAAAAATGAGTTCAAGGTGGCCGAGGAGGCGAAGCGGTTGATTGCGCAGGGCATCGAC
GCGAAGACGATTGAGGAGAATGCAACGCAGCGGATGCACGAGTGGGTGGCGTCTCAGGTGCCGAGCACAC
TCGTGGGGCTCGGAGCGTCATTCCATCTCGACTGGGTACGCGGAGACCCACACACGGCGTACACTGTAGC
CGAGGAGGACGAAGTCGCGGTATACGATGTTCGGTACAAGCTCCGCCGAAGGAACGATGTCGCGTGTGTC
TTGGAGTCTGGTAAGAGGTAG
>AY230148 210, 2660 SCG5
ATGCGAGAGGAGAACAATGTGCCAGCGGAGTGGGCTCCCAGGGACACGCACCGCCTCGACCCTCTTCAGC

TGCTTCCCCCCTCTCAGAGGCACTGCGGGCAGAGCAGCGCTGCGGAGAACGGAGAGGGCTGTCACTCGCG
CTCACGCGGCAAGCCGGAGGTGCACTCGACTCGAGCGTCGAGCGGTGGTAAGAGGAGCACCGGCACACTG
CCGAGAAGCGATGACTGCTTGAATGCCGTAGCACAGACCCCCCTGTTCAACTCCTGCGGTCATCCTCGAC
GATCCGCGGCACAATGTCTGTGTTTACACACTCGACAGCGTCGTCTAGTTGTGCTAGCCGTCTTACTTGC
TCTCATCGTCATCGTTCACTGGGTCATCATCGAATTTACGACCAATATAGACGCACAACAGAAATCATCG
ATAGAGTTGCTCAATGAAGCGAACCGTGAATATGAGTCTTCATCGCCGTTCTCTGTCCTGGTGCAGCACG
AGAGTGCTGTTGAGCGGTTGACGACCGCACAGCGTGGTCTTGCGGCCTCTGGCGCTGGCTCTCTGGCGAG
ACTCGACCGGGATCAGCTGCCGTCGTGGACGTTGCGTGTGATCGACGAGGACTGCACGATGTGCTTCGAC
AATGTCACATACGCTTCTGCGGTCGCTGCGGATGCGGGGAGATCGACTAACAAGACGGGACATCATAAGC
AAAAAGGGCTTTCCGTGTTCGCGACGACATCTGCACCGCTGGGCCTCATGGGGCCGATGCTGTTTGCGAT
GGCGAGCGTGACGGACGACGTGGACGTTGCAGCGGAACTGCCGCGGTGGCAATGGGTGACCTGGTCGTAC
GCGCAGCAGCGCCGCTTCGTTCACGCATCGCAGTCGCGCAGCACAGGTGAGCGACCGCGACACTTGATCG
TGGTGGGCATACCTTCAACGGACCAGCCGATGCGCTACCCTCTGCGGGACGCGCAGCGGGCGACGTGGCT
GACGTACCGAGAGGTTGCACGCACCGAGAACAACTTTACTGGCGCGCTGCTGCAGCTCTACGTGTTCGCT
GCTGCGGAGCATGATTCTGAGGACTCGACGCATTCCGCCGTGGACATGGTTCAGCTGGCCCCGACAGTGA
GCGAGTACGCCGCAGCAACTGAGCAGCACCTGGCTGTGGAGGGTGGAGACGTCAACAATGCACCCCCCTA
CCTGCAGCGTCGCGTGGTGCTGCGTGATGGGTGGCGCGACATCCCACGAAGCGATGACGCGGTGTGGAGA
TCACCCTGCGCTGGTGTAAGGACCACCGTGGTGACTGCGGCAAGTCTTGTGGCCGGGACCTCGTCCCTCG
CGGCTCTTTCAGCCCAGTTGTCGCTGCCCGTGACACCTGCCTTCACAGCAGCGGCGCAGTACGTGTGCCA
CGCGTCCGCGGCACTGTGGCAGGAGGCGCTGCACCACCGCAACTCGCTATGGCTGGACTTTTGGACGGAC
CGCAAGCCGACCACGAAAAAGAAAATGGGCATGAGTATATCGTGGGGTATACCGACGGAAGTAGGCATGT
CTCAAAAGACCGTGTTATGGCTGAACTACGCGTACACTGCCTTCCCGGACGTGCCGTACATCATGAAGGG
CGACGACGACACGTACCTGAAGGTGCCACAGTACTTGAGCGACCTGCGGCATGTGCGCGGTGGGTGGCAG
AAGCCGCGCAACTTGACGGCGACCCTTCCACACAGAGGGGTTGTTCCACCGACGCTGGCCATCGACGATG
CGGAGGAATGCCTGTATCGAGTGTGGTGGTGGTACGACAATCACAACGTTGTATTTGGCCACGGTCCTGG
CTATGCACTAGACCGTCGTCTCATCAAGGCTGTACTGAACCCGTTCGATATCACCAACCAACGTTTGCTG
ATGCTATTAACGGTGCCGTACACCCTCGTGTATCACGGTCATTACTTGAGCTTGCTCATGCAGTACGAGG
ACCTTTTCGTGGGACGGCAATTGCAAAGTCTTTCTGCTAGGGTGAAGGAAGTTTGCATGAAGCGCCCACT
GCGCTACGTATCGGACAGAGAGCCGCGTTCGCTGCAGGTTCTCCGACCCGCGCCCTCCAGTCAGACCTGG
ACATGGAGCTCGGTACTGATGCACTATGCTATGCCGGAGATTCCTTATTTCATCCACTACTACTTCAAGC
ACGAGTTCAAGGTAGCCGAGGCGGCTAAAGGTGCGCTTGCGCAGGGCATCAACGCCAGCGCCATCGATGC
GAATGCGACAAAGAAAATGAAGGAATGGGTAGCGTCGCAAGTGCCAACCGTGCTGACGGGTCTGAAAAGG
GTGAAGAATGTACGCTGGGTGCGCGGAGCCCCACGCACCGCGTACGTTGTAGCTGAGGGCGACGGTGTTG
CGGTGTACGATATTGCGTACAGGCGTCGCAAAACGATGCTGATCGAATGTGCCATCGAGTCTGGAAAGTA
G
>AY230149 282, 2726 SCG6
ATGCGAGAGGAGAACAATGTGCCAGCGGAGTGGGCTCCCAGGGACACGCACCGCCTCGACCCTCTTCAGC
TGCTTCCCCCCTCTCAGAGGCACTGCGGGCAGAGCAGCGCTGCGGAGAGCGGACAGGGCTGTCACTCGCC
CTCACGCGGCAAGCCGGAGGTGCACTCGACTCGAGTGTCGAGCGGTGGTAAGAGGAGCACCGGCACACTG
TCGAGAAGCGATGACTGCTTGAATGCCGCAGCACGGACCCCCCTGTTCAACTCCTGCGGTCATCCTCGAC
GATCCGCGGCACACTGTCTGTGTTTACACACTCGACAGCGTCGTCTAGTTGCGCTAGACGTCTTACTTGC
TCTCATCGTCATGGTTCACTGGGTCATCATCGAATTTACGACCAATATAGACGCACAACAGAAATCATCG
ATAGAGTTGCTCAATGAAGCGAACCGTGAATATGAGTCTTCATCGCCGTTCTCTGTCCTGGTGCAGCACG
AGAGTGCTGTTGAGCGGTTGACGACCGCACAGCGTGGTCTTGCGGCCTCTGGCGCTGGCTCTCTGGCGAG
ACTCGACCGGGATCAGCTGCCGTCGTGGACGTTGCGTGTGATCGACGAGGACTGCACGATGTGCTTGGAC

AATGTCACATACGCTTCTGCGGTCGCTGCGGATGCGGGGAGGTCGACTAACAAGACGGGACATCATAAGC
AAAAAGGGCTTTCCGTGTTCGCGACGACATCTGCACCGCTGGGCCTCATGGGGCCGATGCTGTTTGCGAT
GGCGAGCGTGACGGACGACGTGGACGTTGCAGCGGAACTGCCGCGGTGGCAATGGGTGACCTGGTCGTAC
GCGCAGCAGCGCCGCTTCGTTCACACATCGCAGTCGCGGAGCACAGGTGAGCGACCGCGACACTTGATCG
TGTTGGGCATACCTTCAACGGACCAACCGATGCGCTACCCTCTGCGGGACGCACAGCGGGCGACGTGGCT
GACGTACCGAGAGGTTGCACGCACCGAGAACAACTTTACTGGCGCGCTGCTGCAGCTCTACGTGTTCGCT
GCTGCACACCGAGATTCTGACTACACAGCGCAGTACCTCGGGGACACGGCGCAGGTGGCCCCGACAGTGA
TCGAGTACGCCGCAGCAACTGAGCAGCACCTGGCTGTGGAGGCTGGAGACGTCGATAATGCACCCCCCTA
CCTGCAGCGTCGTGTGGTGCTGCGTGATGGGTGGCGCGACATCCCACGAAGCGATGACGCGGTGTGGAGA
TCACCCTGCGCTGGTGTAAGGACCACCGTGGTGACTGCGGCAAGTCTTGTGGCCGGGACCTCGTCCCTCA
CGGCTCTTTCAGCCCAGTTGTCGCTGCCCGTGACACCTGCCTTCACAGCAGCGGCGCAGTACGTGTGCCA
CGCGTCCGCGGCACTGTGGCAGGAGGCACTGCACCACCGCAACTCGCTATGGCTGGACTTTTTGACGGAC
CGCAAGCCGACCACGAAAAAGAAAATGGGCATGAGTATATCGTGGGGTATACCGACGGAAGTAGGCATGT
CTCAAAAGACCGTGTTGTGGCTGAACTACGCGTACACTGCCTTCCCGGACGTGCCGTACATCATGAAGGG
CGACGACGACATGTACCTGAAGGTGCCACAGTACTTGAGCGACCTGCGGCATGTGCGCGGTGGGTGGCAG
AAGCCGCGCAACTTGACGGCGACCCTTCCACACAGAGGGGTTATCCCACCGACACTGGCCATCGACGACG
CGGAGGAATGCCTGTATCGAGTGTGGTGGATCTATAGCAATAAAATCGCGTATGGCAACGGCGTGGGCTA
CATTCTGGACCGTCGCCTCATCCAGGCTGCACTGAACCCGTTTGATGGCTCCAATGCTCTTCTGATGAAG
CTGCTGACAAATCCCTACGATTACAGGCTGGAAAAGGAATATCTTAGCTTGAACATGCAGTACGAGGATA
TGCATATCGGGAAGCAGATAAGAGATCACCACGACGCCGTGGAGAGGTTCTGCCCCAAGAAGCGCGTGTG
CTACATGGCTGACGGGCGGTTCCGGGCGCACCAGATTCTGCGGCCAACGCCTGTCAAGCTGACGTGGATC
TCGGTGATGGCGCACTTTGGCATGTCAGCGATTCCATACTATGTCCACTACTTCCACAAAAATGAGTTCA
AGGTGGCCGAGGAGGCGAAGCGGTTGATTGCGCAGGGCATCGACGTGAACACGATTGAGGAGAATGCAAC
GCAGCGGATGCACGAGTGGGTGGCGTCTCAGGTGCCGAGCACACTCGTGGGGCTCGGAGCGTCATTCCAT
CTCAACTGGGTACGCGGAGGCCCACGCACGGCGTACACTGTAGCCGAGGAGGACGAAGTCGCGGTATACG
ATGTTCGGTACAAGCTCCGCCGAAGGAACGATGTCGCGTGTGTCTTGGAGTCTGGTAAGAGGTAG
>AY235572 742, 2520 LPG1L
ATGAAGGGCAGACTACACCCCGCCCGCCGTGGCCTGCGTTACGAGAAGCGCCATCGACGACCAATTCTTC
TAGGCGGTGCGTGCGTGTTAGGGTACCTTGTGCTCGGCCTCTTCGTGCTGCCTCACGCCTCCTTAAGCGA
CGTAACACAGCCCACGGTTTCGATGGCGCCAGTGCCGCCCTCGTCGCTGTCGCCTCGATCGACTGAGCAT
GCAACATCAGGGGCGCCAAGCTTCACTCTCCCCTTCACCGACGCCCCTCCTGGCACTCTTACTTCTATCA
CTACCGCGGCACCGCTACCGCGTCCCGCTGTAGACGAGAGGAGCAATGCCGCGCATGCCACGGGAGAGGC
CCCAACGGGGTCCACGCACTCCATCTCCGACGCTCCACAACCCACGGAGGTACCACAGCGCGGCGCAGAC
GAGTTGCACAGCCGATACGTATCAGAGCTCGCGCAACTCGACGTCCTCGATGTCTTTCCGCTCCGCCCTC
ACGAGCGCCGAATCTCGTTTATCGAGTTCGCGCGCTGTCTTGGCGCCCGTCTCTCCGTCAGCCCCGAGAC
GGGAGTAGAGGTGGAGCGGGACGACCTCGCCTCCGACCGGTACGCGCCGTTTCTCATCTCCGTCACTCTG
GAGAACACACAGGATCTCAAGGCGCTCATCTGCAACTTGACGATGCCCTACCGCTACATCGTACTCGCCC
AAAATGGCGACACGCCAGAGGTGACGCCGTTTTTCCGCCTGCTGCGCCGCATGTTCGCCTTCACGACGCG
GCTGACGGTTCTCCAGTTCCCCGAAAACATCGGCTTCGCCGGCGCCGTCAATGCCGGTCTGCGTGAGGCC
CTCAGACACCCGTTCAGCGAGGTGCCCTTTCTCCACATTGTGCACAACGACGTGCGCTTCTTGTTCTCGT
CCCTTGAACAGGCTGTGGCAGGCGCCTACAAGACGACGGCGAGGGACACGGATATGATCGAAGCTCTGGA
GAAGGAGGTTGCGACAGAGCCGAACGAGTACACACCGCTGATCCGGCAGCCCGACGGCTTGCGCACGCCG
CTTCTGCCGGGTACCCCGCTGCCCCAGCAACATGACCGCAAGCCGGTTGTCGTCACGTCGGCGCTGTTGC
CGGACCGTGTTCGGTACATGGCCCCGTCCAGGCGCGCGGAGCTGATGGTGGGGCACACTTCCTTCATGTT
CGCCAACAGCCGCGGCGAGTACACGAGCGTCTTTCTTTCTCGCCTGGCTGTGCTGGCGGTTGGCTTCTTT

GACGAGAATTTCTTTCCTATCCTTTACGACGACACGGACTACCGCTGGCGTGCCCACCTGCTCGGCTTCG
TTGAAGACCGCAACGCTGCTATGAACGACCAGGTCATCTCCTTCGACCTCGACTGCGTCAACCAAGCCAT
GAACGACGTCGACGACGATGGCAATGCAGAGGGTCGATTGGGGGGGCTTCGCAAGGCAGCTACGGGGCCG
ACGCTGTCCCTGAAAGGCAAGGCCCTTCAACGTGACTGCCGCAAGGCCTTCTATGCCGGGGTGCAGTACA
CGTATATGCAGCAGAAGTGGGGCGTGGAGTCCATGACGGAACTCATGCAGGCGCATACAAGGCGGGAGCC
CTTTAGCGGCGAGGCGTTCGGCGGTAAACTGCGCCTGCCGCTGGATGCGTGGGTGGTGGATAGGAACCGG
CTGACCAACCTGCGGACGTGGCTGCGCGACGTGGGGCGGCGCATCCTTGATGTGGACAACTATAACACTG
ACGTTATTCTGCAGGCTGTCAGCCCTTAA
>AY235573 562, 1944 LPG1R
ATGAAGCGCGGGCAGAGGTCGCACGCTCGCGGTTTTTCTCCTGCGCGGCGATGCCTGCTGTTCGTAGCCT
GTGTGTTACTCGTAGCCAGCATCACGCTTCTTATCCTGTACTCGCTGCCTGACGCGGCGGTGGGAGCAAT
AGACGGGGGTAAGGAGGTTTGGGTCACAGCACCCTCGGACCAGCCAGTAACAGATGAGGAGCTGTTCGCT
TGCACTCTCAGATGGCTGCAGACAGACGGTGTTCCGGCCGACACCGTCATCCCGTTCGTGATTGTGCCGC
TGACACTGGAGTGGGAAGATTTCCGCATGTTCATGTGCCGCGTGCACGCGCGCGCGCGCTACCTCTACAT
CCTGCAGAATGGCTCAGTGGAGGAGATGACTGCGCTGCTGACGAAGCTGCGCGCTGCGGTGCCGTCGGAG
CGGCTCATCATCGAGCACCGACCGCAAAACATCGGCTACGCTGCGGCTGTCAACGAAGGGTACCGGGTGG
CCCTGCAGAAGCCGCATGCCGAGGTGCCCTTTATCGGCGTGTTCAACTGCGACGTGTACTTTGAGCGCCG
GTATTTTGGCCGGTACATCCCCGTTGTGTACAAAATGCTGGCCCCTGACGCGGCCCGCATTCGTGCGCTG
GAGGAAGTGGTGGAACAGGAGGAGCGGGCGGCGCGCGCGGCAGGGCGGCAGACACTGCGCGCCAGTGTGG
CAACCACACCCGGTCTTTCCATCTCCATGCTCCTGCCGGACCGGATTAGATACAGCTCCTTTGCAGAGCA
GCAGGACGAGTTCAAGGGGCATGTAGGCATGTTTTACTACGAGACCAAGTCCATGTGCGCCTTTCTGGTG
TCGCGCCTCGCGCTGCTGGTCGGCGGCTTCCTCGATGAAAACTTCTACCCCGCCTACTTTGAGGACTACG
ACTGGCAGTTCCGCCTGGCGAACCTTGGCTTCAAGACGTTTACGGGCTCCTCAGCGCAGTTTGGCGGCTT
TTTCCACAGAATGGGTGGCAACATCCGCGTCATCAACAAGGGCCCCGCCGGCAAGTCCATAAGCTACGAC
ATGGCGGCGATACGGGTGGAACGTATGCTTAACGCCAAGCCGGGCATCGACTACGGGGAGACGAAGTGGG
CGTGGCATCGCGAGGCGCTTGTGGCAATGAGCGCGACGCCCTTCCCGCAGCTGGGGTTCATTGTTCCGTT
GGATGTGTGGGTGCTGGATGCGGCGCGGCATCAACTGATCATCGACATCGGCACGGGGCGTCGGCCATCC
GTGGAGATGTCGAACACGTACAACGTCTCTCTCCTTTCGAGTCTGCGGCGATTTGCACTGGCGGACGGCA
AGGACTTCTGGGGAAGCGGCATTATTACAACAGAGCCGGATTGGCGAAAGTAG
>AY235574 1, 1617 SPT2
ATGATTCACGATGCTGTGCAAGCGGCGTCCACGCCAATGAGCGAGGCGGCGCTGAAGCGCGAGATTGCCT
TGCAAGAGGCGTCTGATGTAGTGGACAACAAGTCGCGGGTACTCCCCAAGTCCCCGGCCGACACAAGGGA
GAGAACGCACGAGGTGGTCGCCGCCACGGAGCTGTTCGAACATCCCATCTCTCTGCGCTCCAAGGTGTCG
GCCTACGTCACCCTCGCCCTGCTCTTCTTATTCGCGCGCTTGCGTGAGAGCTATCGCCAGTTCTTCCCGA
ACGATGAGAGCCATGTGCGCCCCGGCTACGCCCCCATCGTGAAGGACTTCGATCACTACTGGAACCGCCG
CTTTTACCGCCGCGTCCGCGACTGCTTCATGCGCCCCATCGACTCTCGCCCTTCGCGTGTGATTGGAGTG
ATGGAGCGCGTGTCGAAGGACAACAACCAAACGTTCGAGTACAGCGGTCGCATCGTGCCCGCGGTGAACA
TGGGCTCGTACAACTACCTTGGGTTCGCCGAGGACACGCAGAGCATCACGCACGAGGTCCTAGATTCGAT
CGACGACTACGGCCTCGCCTCATGTAGCGCGCCGCAGGGGTTGGGCCAGAGCGCCCTCGTGTCTCGCCTC
GAGCACGAGTTCGCCGAGTTCCTCGGAAAGGAGGATGCCATCGTGTGCGGCATGGGCTTCGCCACGAACT
TCCGCGGCCTCCCCACCCTCTTCGGCAAGGAAACGCTGGTGCTCTCTGACAGCCTCAACCACTCCTCGCT
TGTCAACGGCGTGCGTTCGTCTGGCGCAAAGGTCAAAGTCTTCCAGCACAACCACTTTGGTCAGGTGGAG
AAGTGCCTGCGCGAGGGCGTAGTGCTCGGCCAGGACCCATGCGGCGAGTACAAGCCGTACAAGCGCATCG
TCATCATCATTGAGGGCATCTACAGCATGGAGGGCGAGATCGTCAACCTCAAGAAGTTCGTGGAGCTGAA
GAAGAAGTACAAGGCTCTGCTCTTCGTGGACGAGGCGCACTCCATTGGCGCTATTGGCCGCACTGGTCGC

GGTGTGTGCGAGCACACCGGCGTCGACCCCAATGACGTTGACGTGCTCATGGGTACCTTCACGAAGAGCT
TCGGCTCCATCGGCGGCTACATCGCAGCCGACAAGACGCTCGTTCGCTACCTCCGCCAGCATAGTTCTAT
TGCCCTGCACTGCGATCCTCTGGCACCGCCGTGTGCGCAGCAGGTGCTGTCGGTGCTGCACGTGCTGCTC
GGTAAGGACGGTACGGACCTCGGCGAGAAGCGCATCCGTCAGCTCAAAGATAACTCGTCATTTTTTCGTC
GCGGGCTGATGGACCTAGGCCTCGTGGTGCTCGGCGATGACAGCAGCCCGGTGGTGCCGGTGATGTGCTA
CAGTATCGGCAGGCTTAGCGCCCTCTCCCGCCTGTGCCTGGAGCGCGGTGTCGCTATCGTCATCGTCGGC
TACCCGGCGACGCCGCTCCTGCAGGGCCGCGTGCGCTTTTGCGTCTCGGCCTGCCACACGCGCGAGGACC
TGCAGCGCACCCTTGATGTGATAAGGGAGCTCAAGGGCTACGTCTGCATGGAAGACAGCACGAACCCGCT
GCGGTAA
>AY235575 1, 1467 SPT1
ATGTCGTCTCTATCGGAAACGCTGTCAGAGCCGCTGATGAGCCGCCCGTTCCACCGTGCTCTGGAGCTTT
TCGTTGCCGCCGCACTGGCGTATTTTCTGCTGCAGCGCTTCCACCGCCGCGGGAGGGGGAAGCCGCCAAA
TCCGATGAGCAGGCTGTCCGCTGAGGAGCAGGAACGTCGCATTGCCGCCTTTCAGTCGATTCCGTTCCGT
GCCGAGTGCTCGGTGACGTCGAACGTGCCCGTGCCGGAGTACCACGGGCTGACCGAGGTGGTCGGACGCG
AGGGGAGCCACATCACCATTGTGCGGCCCGGCTCATCGGAGGCGGAGGAGTGCCTAGACCTTGCCACCTA
CGACTTTCACTCGTTCTCCACGCTGCCGGAGGTCGTTGAAGTTGCGCGGGCGGCCGTGAACGCCTACGGC
GTGGGCAGTTGCGGGCCTCGCAGCTTTTACGGCACCATCAAGCCGCATCTTGTGGTGGAGCAGGACCTGG
CCAAGTTTCTCAAGACGGATGACGCGGTTGTGTACAGCTTCGCATATGCCACCGTGGCCACCCTCATTTC
CTGCTTTTCCGGCCGCGGCGACTACCTCGTGTACGACGACGGCGTCAGCTCCTCCGTGATGGAGGGCTGC
ATGCTTTCTCGTTCTGACATTCGGCCGTACAAGCACTGCAGCATGACGAGTCTGGAGGAGCGGCTCCAGG
AAGTGGTGGCGAAGGACGGGTACAGCAAGCCTCACCGCCGCTTCGTCGTAACGGAGGGCCTCTTCTCAGA
TACCGGCGACATCTGCCCGCTGCCCCAGATCTTGGGGCTGTGCCACAAGTACCGATTCCGCCTCTTGCTG
GAGGACAGCTACGGCTTTGGCGTGCTGGGCAAGTCAGGGCGCGGAACTCCTGAGCAGTTCGACATCCCGA
CGATGGACGTGGACGTGTACATTGGGAGCCTGAGCACGTCTATGGGTGCGGTTGGCGGTTTCTGCGCTGG
CGCGTCGCGCATGATTGATCATCAGCGCCTCACCTCCACCGCGTACGTGTTCTCGGCGTCGCTGCCCCCC
TACATCACGGCTTCCGTCTCGCAGTCACTCGCGGTGCTGGCGCGTGATGACACGTTTGTGGCCAAGCTGC
GGAAACACACGAAGCGCATGCGCAGACACCTCCGTGAGGCCGGGTTCAATGCTGAGAAGATCAAGCTGGT
GGACAGCATCGACGACGCCTCACCGGTGATTCTTCTTGCGGTGGAGCGGGCGTACGTCGAACGCGAGGGG
CTGGAAAAGGTGGAGAGCCGTCTCCAGCGCGTCATCAATGCCCTGCAGAAGAAGAAAGTGGCGGTCGTGC
GAAACGTCTTCACAACAGATGAGCCGGTGAACAACGTCTCGGGACTGCGTATTGTGGCCAAGAGTCTGGC
GACGGAGGCCGAGGTGACGGCGGCGCTCGAGGCAATCGAGACTGCCGTGAAGGCCGAGTTTTCGTAG
>AY251609 1813, 7236 pentamidine resistance protein
ATGAGCAGCCAGCGACCGGAGATGTCAGAAGGGCCAGCGAGTTATAGCGCCCACTCGTTCGGCGAGGTCA
ACCGACGGCTGTGGCGGTTGCCTACTAAGTCGGTACCGAAGGGCAGCGGTTGTGATGATGCTGTGCTGGA
CTGGGCTGCGGTACGCGAGTTGCTGGAGGAGGCTCCGGAGGAGCGGAGCGGCGTTTTGTCGCGTTACCTG
CTGATGTGGCTGAACCCGTATGTGACGCTTGCGTGGAGGGAGCGGCTGGAGGAGGCTTACATGCCTCCAC
CGCAGCGTGCGCACCGCGCCGTGTGCTGTGGCGCTCTGTTGAGCCGCGCGTTTCGTGACGAGGAGGTTTG
CGGGGGCCGTGACGCGTGGCGTGCGCGCGTTGAAGCTGCGCTGCCAGTGCTGTGTGGCGACTGCGCTGAC
CGCGCTAGTGAATGTGAAGTGGGTGGCAGCGATTGTCACGGGCGATGGGGTGGCGCGCGCGTGGCGGCTG
TGAACGAGCTGTCACGTGTGGGGTTGCGTGGTGAGGAGGGTGTCCACGGACGGCTGCGGTGGGTGGGGTA
CGTTCGATCGAGCGACGCACCGCAGACGCTTTTGTGCGGTGTGGAGTGGGATGCTGACAGTGCGCTGCCG
TTGTATCGTGCGCGTGTTGCTGGTGATGCGGCCGCGGAAGTGGTGCACGATGGGTGCGTGCACGGCGAGC
GTCTGTTTTACCAGGTTCAGGAGGGGCGGGCGCGGTGCACGTGCGAGTACGTGGAGGATCTGGTGCCGGT
ATCGGCGATTCCAACCGCCGATACCGGGTGCCAGCGTACGCCTCACACTCCGTCGCTGCTTTGGGCGCTG
CTCCGCACGTTTCGCTCCGATCTGATGGCGATACTGCCACCGTCGATTGCGGGTATGATGTGCGAAGTGT

TGACACCGTGGTTGCTGCAGCAGTTTGTGCTGTTCCTGCAGTCGAACAAGGATCAGGAGGGAGTGCGAAA
AGGGCTGCTCTTCTTCTTGATGTTTGCGATCGTGAAGTTTGTACAGCCAGCGTTTGTGAACAAGGAGATG
CACCGCAGTCGGCGTGTTTCGAGCCTTTTCCGCACCTCGACGCTTGCCCTGATCTTTGAAAAGTGTCTGA
TGATATCGCCAGATGCGCTGTCGCGGCCGGACATGAGCACAGGTCGCGTGTTAGCGATGGCGAGCTCGGA
TGTTGAGAACATCAAGGAATTTCCCACGCAAGTGACGTTCCTGTGGAAGGCACCGACTATGCTGACGTTT
TACGTTGCCTACCTCTTCGTTCTCGTGGGGCCGAGTGCCGTGGCTGCTGTCCTGGTTTTTGCCGCTTCAC
TGCCTATACAAGGGGGGCTAACGAATGCCATAGGTGGCGCACAAGCGAGCCTGTCGAGCTGCACGGATCA
GCGCCTGCGTCGGACGAATGAGCTGCTTTCCGGCATCCGTATCGTGAAGATGATGGGATGGGATTCGAAG
TTTGTCTCGGCGATCGAAGATAGCGCACGGGCCGATGAGTTGCGGTTCCGACGAAGACTGCAGATGTGCC
AGGTCGGGTTGTGGGCATGCGTGTCATCTACTCCGACGTTCATGATCGCCGCTGTTCTCACTACCTACAC
CCTCAGCGGACACAAGCTTGACGCATCCGTGGTGTTCCCTCTCATTGCAGTTGTCAGTGCTATAACCTTC
CCTGTAATGATGCTCCCGGAGGCCTTTACGTCGCTGGCGAAGTTCATTGTGTCGACGGGGCGCGTCACGC
AGTTCTTGGAGTGCGACGACTCGCACATTATCGTCGAGCACGCGGAGCGCATGTTCAGCAGCACAGGGAG
TGCTGCCGAGTGCCGCTTCGGCCGACCTGGCAAGGGTGCTTGTGTCAGTGCCCGCCGCGCTTCCCGTGTA
CGAGCCGCGACACGTGGGGCTGCGCCGCGTGGCGCAGAGGATGCTGTGTGCGGTGCTGCGCCGCCGCGTG
CCGGTGGAGCTGCAGTGGCGGAGGGCGGCTGCCTCACCTGCCACGGGTGAAGGCGGTAGGAAGCCTGTGC
GCGCTGCTGTGGGGCACGATGATGGCGGTGCGGCTGTTGCGGGTGGTGAGGACGGGCGCGGCAGTGATGG
TGGCGTGGCGCTGTACGCGATGGTGGACAGGACTCTCCTGCACGATGTGCGCGTGTCGTTCCCGCGCGCG
CAGTTGACGGTGGTGGTTGGCGCGACGGGGAGCGGGAAGTCTGCGCTGCTGGCGACGCTTTTGGGTGCGT
TCCGGCTCGAGGGGCATGTGGATGTAGCGAGGTCGGTGGCGTACGTGCCGCAGCAGCCGTGGATTATGCA
GGAAACGTTAGAGGCGAACATCACGTTCTTCGAGGGCGATCGGCGCCGCGCGGCTGGTGGCGTGGCGGAC
GATAGTGATAGCGGCTCTGCGCAGGCGTTCTCACCTCTGAGGTGCGCTGAGCAAGCTGCTGGTGCTGCTG
CTGTGTCACGGTGCTTCGGCTGCGGCTGTGGCACGGTGAGGGAGCGTCTTGCGCGCGCTGTGCGGAGTTG
CCAGCTGGATGCTGATGTGGCGCTGATGGCGCGCGGGCTGGGGACAGAGATCGGCGAGTGTGGCATCAAC
CTGAGCGGTGGGCAGAAGGCGCGCGTGAGCCTTGCGCGCGCCGTGTATGCGGACCGCGACGTGTACCTGC
TGGACGATCCACTGTCGGCGCTGGACGTGCACGTTGGGCGACGCGTGATGGACGAGGTTGTGCTGCGCGC
GCTGGCGGGCAAGACGCGCGTGCTAGCGACGCACCAGCTGCAGGTGCTGCCGCACGCGGACCAGGTGGTG
GTGATGCGCGAGGGCTGCGCCGTGTTTGCGGGAAGCTACGCCGCGTACGCTGCGTCGGAGTGGAAGACGT
ACGTAGAGAGCGATGAGGCCGCCGCCGCAAGCAAGAAGAGCGGGTGTGGCGGTGGTGATGCTGTGGCGGA
GTGCACCGAGGTCAGTTCTGCGTGGTCGTCTGCGGTGAGCGTGGAGTCGTGTGGGGGCACGCGTGAGGAG
TGTGACTTCGTTCGGTGTAGCCGTGCCGATGTCCGTGTCAGGAAAGGCGACGGTGAATGGGAGGAGAACA
ATGGCAGGACTTTGTCGAGAATGCACTGGCAGGGCTCACGGGCCAGCGAGAACTCAGAGGCGGAAGAATG
CAACGACGGGGACTCTACGGTATGTAAAAGTGTTGGCCTCGCCAGGTCTAAGGCCGCGCCGCGCACGGCG
AAGGGCTCATCTGGGCTCGCTACGGATGAGGACGCAGCGGACGGCGGCGACCTGATGATTGCGGAGGAGA
AGGAGACAGGGCACACACCGTGGAGTGTGTACCGCGCCTACGTTGAGGCGGGCGGGGGCGTGCCGGTCGC
AATTCAAGTTGTGCTGCGCTACTTCTTTAGCGAAGTGCTCTCGACAGGCTCCAGCGTTTGGCTGACGCTG
TGGTCAGTGAACTACTTTGGTTCTCTCTCTACCAACGAACAGCTTGGTGTGTATCTTGGACTCGTCTTTG
CGGTTGCTGTCACAGTTTCTGCCAACGACCTGTTTATTTTTCAGTTCGCTCGCCGCGCTGCCTGCCGCTT
GCACGCCATGCTCCTGTACACAGTGAGCTCAGCCACGCTGGCATTTTTTGACCGCACACCGCTGGGTCGC
ATTGTCAACCGGCTTAGCAGGGACGTGCACGTCCTTGACGATGAGCTGCCGGCGAACGTGATTCCTTTTT
TGGGCATTGCGGGATACGTCATGACGTCTTTAGCAGTGACGCTATACACGTCACCGCTTAGTGTGGTCGT
GGTGCTGCTAGCGGCTTACGCTTTTGTGCGCCTGCTGAGGTTCTACGCGACAGTGGTGCGTGAGGTGCGG
CGGCGCAGCAGTGTGGGGCAGTCTCCGCTGCTCTCGCTCCTGGAGGAGGTGCTGCACGGGCGTGCGACGA
TCGCGGCATACGACAAGTCGCACGTGCTGTTCGCGGAGGCGCTGCTGCGGCTGGACCTTGTGTACAGCTG
CACGTACGTGGAGAAGGTTGTGACATTGTGGCTCGCTATCCGAATCGAGTCCATTGCGGCATTGGTCATT

ATTGCTGTGGGTCTGATCGGCGTTGTGGAGAAGCTCGTAGAGGCGTCGCCGGCGATGCCGGAGGCGCGTG
TGGGCCTGATCTCGCTGAGTCTCACCATGTGCCTCGATCTCAGCTGGTCGCTATCTGCACTCCTAGACCT
CGCTGCCGTTGTCGAGGCCAGCATGAACAGTGTGCAGCGCGTTTGCCACTACATTGACTGCGTACCGCAG
GAAGCGGTGCTCCTGGAGCCGCGGGACGCGTATGTGGCGCGCGCTTTGGCAGCGACTGGCGGCAGCGGCA
GAGGAGGAGGCGGGAGCACCAGAAGTGCGAGCGACATCGTTGTGGTTGCCGGCGGTGGTGACGACGAGGC
CGCGCCTGCGTGGCAGGGTGTTGCGGCAAGGAGCGGCGCCGGCGGTGCGTTTGGAGCGCTTGTGCTGGAG
CACGTGGACCTGCGGTACCGGCCTGGGCTGCCGCTGGTTCTGCGCGATGTGTGCTTCGCGATCGCCCCTG
GGCAGAAGGTGGGTGTTGTGGGGCGGACGGGGAGCGGGAAATCGACGCTGATGCTGGCCTTCCTGCGGCT
GGTGGAGGTGAGCGGCGGCCGCATGCTCGTCTGCGGGCGCGACGCGCGGACGTACACACTTCCCGCACTG
CGGCGGCTGTTTTCCATGATCCCGCAGGATCCCGTGCTGTTCGATGGCACGATCCGCAGCAACGTGGATC
CGTTCGGCGACGCGACGGACGAGGAGGTGCGTGCCGCGCTGGTGTCTGTGGGGTTTGTGGGGGTAGGCGG
CAGCTCAGCTACCTTTCCGACAGCTGCTGGATTGCCGAGCGCTGCATCTGCCAGCATGTTTGCACTGGAC
ACTGTGGTGCAGGGGGGGCGGGTCGAACTTCAGCGTTGGGCAGCGCCAGCTGCTGTGCCTAGCGCGCGCGC
TGCTGAAGAAGGGGAGTGCATTCATTTTGATGGACGAGGCGACAGCGAACGTGGACGCACAACTGGACCA
GACAGTGCAGCGTATTGTGGCGGAGCAGTTTGGCGCGTACACGGTGGTGACGATCGCGCACCGTCTGTAC
ACGGTGGCGGCGTACGACGTTGTGCTTGTGATGGCGCGCGGGCGTGTGGTGGAGATGGGGCAGCCGCGGG
CGCTGCTGGAGCGTCGCGACTCCGTGTTCTACGGTATGGTGGCGCAGAGTGCAGCGGTGGCTGGTTCCCG
TGAGGGCTTGTCGCGGGAGGATGCCGAGGGTGCGGCCAGTGGTGCGCGTTCGCGTGGCGTCAGTGACGGA
GGCGCTGAGCTGAGCGCGGCAGGGCGCGAACACGGCGTGGAGGCCGCGGTCGCGTCGCTCATGCGTCAGT
GCAAGTGACTGCGCATCTCTGCAAGTCTTCATT
>AY255810 29, 184 A2
ATGAAGATCTGCAGCGTGCGTCCGCTTGTCGTGTTGCTGGTGTGCGTCGCCGCGATGCTCGCACTCAGCG
CCTCCGCTGAGCCGCACAAGGCAGCCGTTGACGTCGGAACGCTCTCTGTGGGCCTGCAGGCCGTTGACGT
TGGCCCGGTGTCGTAA
>AY273788 2552, 3913 AAAHL
ATGCTGCTGCGCAGTCGTTTGTTCCTAGAGGCTGGCATGGCTGCTGCCGCCGCCACCCTGAAGGCCCAGT
ACGCCTCCCCTGGGAGCAACATCAAGGGCGCCGACAAGAAGACGCGGTGCCGCACGTCCCTGCAGGTGTC
CCTCTCCACTGATAAGCCTGGCGAGCTGTGTCACCTGCTGAGCGTCTTCAAACCGTACAGCATCAACATC
AGCCAGGTCGCCAACCGTCCTCGCGCGTACGAAAACAAGGCGCCGCTGCGCACCATCTTCCTCGACGTGG
ACGCTTACATTGAGGACGAAAGCATGAAGAAGGTCATGGCGGAGCTGCACGCGAAATTTCCGAATGTAGT
CGTAACCGGATCGTGGGTGATTCCGTGGTATCCGACGGAGCCCAAAGACCTCGACGAACTCGATCAGAGC
ACGCTCGCTGCCGGGGAGGAGCTACAAGAAGACCCGGAGAACCCCCACCCCGGCTTCCACGACGAGGTCT
ACCGCGCCCGCCGCCGCGAGATTGTCGGGCTTGCCAAGAACTACAAAACTGGAGATCCGATCCCGATTGT
AAACTACACAGAAGAGGAGAACCGTGTGTGGACGGTGGTCTACGACCACCTGACCCGCCTGTACCCGACG
CACGCGTGCCAGCAGTACAACTACGTCTTCCCGCTGCTCCTCGAAAACGGTGTGCTGAGCCGCACCAAAA
CGCCACAGCTGCGCGATGTGAGCGAGTTCCTGAACGAGGCGACGGGCTTCACGGTGCGCCCGGTGACGGG
GCTGTTGACGTCGCGCGACTTCTTGAACGCACTGGCGTTCCGCGTCTTTTACAGCACCCAGTACATTCGC
CACGCGGCGCAGCCATTGTACACGCCGGAGCCGGACATGGTTCACGACATCATTGGTCACCTGCCCCTGC
TCTCTGATCCCGACTTCGCCAACTTCACCCAGACGATCGGCCTCGCCTCCCTTGGCGCCAGCGATGAGCT
GTTGGACAAGCTGGCAAAGGTCTACTGGTATAGCGTCGAGTTCGGCCTCTGCAGCGAGGGCGGCCGCCGC
AAGGCCTACGGTGCTGGTATTTTATCAAGCTGTGGCGAGCTAGAGTACGCACTGAGCGACAAGCCGGAGT
GCGTCCCGTGGGACCCCACAATGGCGTCCAAAACGCCGTTCCCCATCACCAAGTACCAACCGCTGTACTT
TGTGGCCGAAAGCTTCTCAGATGCGCAGCGTAAGCTGGAGGCGTGGCTCAGCTCGCAGGAGAAGCCGCTG
TACACCGTCTACAACTCGTACTCGCGGCGCGTGCAGTCGTACCCGAAGAATACGTGGAACATGCTCCAGG
AGCAGATGAAGCGAACAAACTTCACCTTCTAA

>AY27807 545, 1114 Leishmania major terbinafine resistance locus, partial sequence
ATGCTCAACGAGGTGCACGTGAACGCAGACCCGAACAGCATCTCCACCTTGGATGAGCCGGTGCTTGAGA
CGCTGCTGCGCGATGCCAAGGCGATCGGCCGCAAGCTCGTCGTGGTTGTGTGCCCCTCTCTAGGAGGTGA
TAAGGAGCTTCGCGATTGGGATCTCTGGGGGCCGCTGTTCCTGTGTCTCATTCTGGCCTCCATCCTCACC
ATCAACGCCAGCGACGACCAAGGCGCCGCCGTCTTCTCTGCGGTCTTCATCTTTGTGTGGCTTGGCGGTC
TTGTCGTCACCGTCAATGCCAAGCTCCTTGGCAGCAAGATCATGTTCTTCCAAACGTACTGCGCCATGGG
CTACTGCTTGGCGCCCATCTGTCTCGGCGCTCTCCTGTGCTGCGTCCTTCCGTGGTTTTTATTGAACCTC
ATGCTGTGCTTCATGGCGTGGGCGTGGGCCTGTTGGGCGGCCTTGCGCTTTTTCCGACACACCGTCAGCG
CCGACCGTGAGGTGTTGGTTGTGTACCCTGTCGGGCTCTTCTACGTCTTCTTCACCTGGATGGTTCTGGT
TGGTATTTAG
>AY328521 1178, 3043 ADS1
ATGCCCGCTGACGAGGCGCAGGCGCCCACCCAGACGGAGGTGTATGATCGCCTCAAGTGGAATGGGTGGG
GTGTCGAGGATGTCCAGATGCAGATCGACGACGAGAACCCGCTCTGGGTACGCCACGTGGATGGCAAGCC
GATCAAAAACTTGCTTGAGTTCCTCTACCAGGAGATCAGCGGCGGAGTAGGTCCAAAGGAGATCCCGCCG
CTGTCGCCGAGCATCCCGCTAGAGAAGGCACTTGCGAAACTTAACAAGGCGAACATCAACGAGGCCTTCA
TGAAGGACATCTCCGCGGTGCTGGAGAAGAGCCAAATTCGCCCTGACGGCAAGAGTCGTCTGTGCCACAT
TGCGGGCAAAAACTACCGCGACCTGTGGCGTGTGCGCAAGGGCATGGTCGAACACGCTCCAGACTGCATT
CTGCTGCCGAATAGCCACAAGGACTGTGTCGAGATTATGAGGCTGGCGCACAAGCACAACGTGGTCGTTA
TTCCTTTTGGTGGCGGCACCAACGTGACGGGTGGCGTCGAGGCAGACCCCTTCGAGCGCGCCCGCATGAT
CATCTCCGTGGACATGCGCCGCATGAACCGCATGATATCGATTGACCGCGAGTCGCGTCTGGCCACCTTT
GAAACCGGTGTGCTTGGCCCTGACCTGGATGCGCAGTTGTTCCGCCACGGCTTCATGTTGGGCCACGACC
CCGACAGCTACATCTACTCAACCCTCGGCGGATGGATTGGTGCACGCGGCAGCGGGGCCATGTCGAACCA
GTACGGCGACATTGAAGACATGGTGGTGGCGGTGAAGGTGGCAACGCCGACAGGCATCATCGAAACCCCA
ACCACGTCCCGCACGTGCGGCGTGGACCTGAACGGGCTCTTCATCGGCTCCGAAGGCTCCTTCGGCATCA
TTACGGAGGCCACCATCAAGCTGGAAACCATCCCGGAGACGAAGCTATACGAAGGGTACCTGTTCCCCAC
CTTCGAGGCTGGTTTCAGCGCCTTCTACACATGCACCGCCAAAGGAATCCATCCGTGCACGATGCGCCTG
TACGACGAGGACGACTTTCGTATGAGTATGGCGATGAGCACCACCAAGCACAGCTTCTTCCAGCGCTTGG
TGAGTATGGGTGTCAAGTCCTTCCTTGAGAGATACCGCGGCTGGAACCTGCGCCGCATAAGCCTAGTGAT
CGTCGGCTTTGAGGGAACGCCGGATCGCGTCAAGTTCCAGCGAAGCGAGACGGCTGCAGTCTTCAAGCAG
TACGGTGGCGTCGGTGTCGGCCGCAGCGCGGGTGCTACGTGGCAGGATAAGAAGTACGACCTGCCGTACA
TACGTGACTTCGCCCTGTCTCTCTCGCACTGGGCGGACGTCTTCGAGACGAGCGTTCTCTACTCGCAGGC
GATCCCGTGCTGGCGTGCAGTGAAGGCCGCTGTGCGGCAGGTGTGGAAGGAACACGGGCACCGCGGCTGG
ATTGGCTGTCACACGGCCCACCAGTACAAGTACGGCTGCTGCCTCTACTTCACCTTCGCAAGCGCGCAAA
AGGACGACATGGACATGAAGATATTCCTCGCCATCAAGAAGCGTGCGACGGAGGCGATGCTGGCGCACAC
TGGAAACCTCACCCACCACCACGGCATCGGCTATGAGCACGTGCCGTGGATGAGCCGCTACATGGGGCCT
GATGCGCTGGACCTCCTCTTTGCTGTGAAGCAGAAAGTGGACCCAAAGAACATCTGTAACCCTGGAAAGT
TGCTGCCGTCGCCTCCACGGGAGAACGAGAGCGCCGCAGCGCTGAAGGCACGCCAGCAGAGGGAGCTGAT
GTTCGACAAGATGGGCGTGCCAGGAGCCGTCGCGTCGCATATTTAA
>AY364408 1, 792 soluble inorganic pyrophosphatase
ATGCTGAGTCGAGCGTTGAGACCCCTTAGCAGCAAGTCGGTAGCCAGCGCCGTGACCCTGCCCGTCTACA
ACACCACGGAGGAGGGCCCCGCTGGCAGCAAGGCGTGGCGCATGTTTTACAAAGTTGGCGCCACAGACAC
CATAGTGTCGGCATGGCACGGCTTGCCCCTGTACGCTGGCGCTTCTGCCGATCCGCTTGTCCTCACCTGT
GTGACTGAGATCCCGAAGGGCACGCGCGCGAAGCTCGAGCTGAGCAAGGAGGAGCCGTACAACCCCATCA
AGCAGGACATTTTCAAAAGCAAGGAGGGGCATCCGCTGCGGTACTTCTCGTACGGCGACATGCCTTTCAA

CTACGGATTCCTGCCCCGCACCTGGGAGGATCCGGTGCACATCGACCCGAACACCAAGTGCAGCGGCGAC
GGCGACCCCGTCGACGTCGTACACATCGGCACGCCGCATCGCGTGGGCACCTACGGACCAGTGCGTATCC
TCGGTGTGCTCGGTCTCATTGATGAGGGCGAGACCGACTGGAAGATCATCGTCGAGTCTGTGTCGGCGAC
GGCGGGCGAGGGCTACGGGACGCTGTCGAAGGTGCCGCAGGAGTTGCAAGCGACCATCATCGACTGGTTC
GAGAACTATAAGGTGCCGGACGGCAAGAAGAGGAACGAGTTTGCCTTTAACAAGGCGATCAAGGATGCCG
AGACGGCGCTTAGCATTGTCGCCCAGTGCGCCTCGCAGTACAACGCCTTGATGGAAGGGAAGTGCGCCAA
CCCGGGCTATTGGCTGCGGTAG
>AY437471 1, 690 MP18
ATGTTTCGGCGCCTTTCCCCTGCTGCAGCGACACGGTCGATGGCGACCATGACGCATCCGCTGACCTCCG
CGACGCAGTCCCAGTCGGTGACAAGCACGCGCGCGGCAGTCTCACGTGTCGCGATGCGCGGGTGCAGCAG
CAGCAGCGGCACCTCATCAGGCGACGTCTCTTCTACAAAGGGTGCCGCCTTTAGCGTGAGTCGATCCGGT
CAGCGCGCGCCGTCGATCAACTGCGTGACACTCGTTGGGGTGGTGCATGACATCCAAACTGGCTACGTCT
TTGAAGACGCTGTCACCCAATTCACGCTCACCACGACAAGCCTGGACGCGGCGAACCAAGCCCAAGAGTG
CGTGGTGGAGAAGGACCATCACACCGTGCGCTGCTATGGCGATGTCTTTTCCCGGGAAGTCCGCGCCAAG
CTGAGCGAGGGCAATGTGGTCTGCGTGAATGGCCGTCTGCGCTTGAATCCGCAAATGGAGCCGTCGTGCA
ACAAGTACTACTACTTCCCTTACATCCAAGTTCAGCCGCCGCACGGCCAGGTGAGCGTCGTGTACGGCGA
CCGTCGCTCCCCTCCGTCGCCGGTGAACGCGGCGACGGGTGAGCTGAACAATGGCAACGGCGACCCCACA
AACAGCGCAGCACCGGCCGAGGATGGCGATCCTAGCGCGACGACGCAGAAGACGCCGTAA
>AY437472 1, 741 MP24
ATGATATCGCTGCGTGAGGCGACACGTCTCGCTAAGATCGCTGGCTGCTTTGACTCTGCTAATGGGTCGT
CTTCGTCTTTGCTGCGTCGCTGCACCGATTCCGCATGTGCGCAGGGTCGAAACGTCACCAGCTCATCATC
CTCCTGGGAGGAAATCAAGCGGCGGCAACATCAGGTGTTCAACGAGCGGCACTTAACCGCCGAGAACACC
ATTGCTGTCACGGTGTCGTGTGTGAACATGGCGCATCTCTCCGTATACGTGCACTCAGTGCAATGCGGGT
ATCCGGCCTCTCACGCGCCAGGACCGTCTGGGGAGGGCGTGCCTGGCAAGCGCACCATCACCACTGCAAC
GTTGACGAATCAATCTTGCGCCGTGCTGCTCCTGTGGGTGTCGATGGGGTCGCCTGAGTTGTTGGCCGTG
CGGTGCGCGCTGTCTCCCGGTGCTGTGGAAACGTGCTGGCGTGCGGCTCCAGCCGCGGCGAGCAGCGACA
CCACTAAGGCTGGCAGCACGATCCAGACTGCTGCGCCATCCTCGACGCTGTCGGAGAAGGACAAGCTGCG
CCGCATAAAGTCCTGGGCCGAGGAGCTGGTCCTCCATCGATGGGTGGTGGTCAGCGGCCAGCTGCGCATG
GAGGACGTGTACGACGGTGATTTGCATAAAGTAGTGGCAGTGCCGGTGCTGGAGGTGGCAAGGGACCACT
TTAAGGGTAGCGTGCTTGCACTGCCGCCAGGGTTTCTATGA
>AY437473 1, 1347 MP42
ATGCGACGGATTGGTGTGGCAACATGTCAGCGACGGTGGCTTGCCTCAGCAATGCTGCCCCGCGCCGGGA
AGTGCGGAACGCCATGGTCAGGGCACAGCTTCATTCTCGATACCGCGGCGTCTCTCACAACCGCCGTCCG
CGAAGTGCGCTCCACGGCGGTCTGCTGCGCCCCGAAGGATGCGCAGTATCAGTGCGGTGAGTGCGGCAAA
ACGTTCCGCCTCGTCAACGCCCTCAATCACCACATTATGACCCGCCACGGCAACAACGCCAAGGCGCTCA
TGAAGAAGGACGGCAAGCTAGTCCCTGTGGAAACGGACCAGCTCAAGGGTGGGGCGCATTGTGTGTCCTC
CTCCTCCTCCTCCACAGCGACGGCGAGCTCGGCAACTCCGGCCGGGGCTGCCGCCGCAGCGTCTTCCTCT
CCACTGTCCGGCGGCACCACCTCACCGTTTCCGGTGCACTTTGCCGCTCCTTTTGGTGCCGCCGCCGCAT
CCATGGGCGGGGTGGCGTCGTCTTCCACTCCACCAGGCACCGCACCAGGGGTGCTGCAGGCCCCTGCGAC
CGCCGCGGCCGGCGCAACCACGGCAGGCACTGACGAGAATGGGAGCGGCGCCGACGGGGCCGCGGAGGAG
AGGGAGAAGCGCATGTTCGTCTGCACGGTGTGCCAGAAGACGTTTCGGCTTGAGGCGGCGCTACAGCACC
ACTATCAGGCGAAGCACAACATGGACATGCCAACCTCCGCCTCGTCTCCGTCTGCTCGCGGCGGCGCGAG
CACGACAGCAGGGGCTGGCGGCGCCTCCGTGTCCACTCCGGGAGAGCCTGGTGCGAATGGTGGTGGTAGT
GCAGGCGCGTTTGGTGGCGTGCCTGGCGTAGCTGCAGACGGTGCCGCAGCTCCTGCGAACGCTTCTAGCT
TCGGTGCTGCGCAGTACGTGCGACAGCAGGAGGGAGCGCTGCCTGACGCCCCGCAGTACCACCTCGATGT

GGCCCCGAACGCCCCGGAGGAGGGCGACATTGCCGCGCACTGGCGTTGCGTGAACATGTGTGTGCTGATG
GGCGACGTGCAGGAGGTGGAGGAGGGGTACGTCTTTGAGGATCATGTGCTGCAGTTCACTGTGGCGACCG
AGTTTGCTACCCCGGCCCCTGGCGACCCTGACATGGACTTCCACACGGTGCGCGTGTACGGGCACGAGTT
TTGGGCCCCACTGAAGGCAGACATGCAGAGTGGCGGTCGCTTTCTCGTCACAGGACGACTGTGTATGGTG
CCGCAGTTTGATACGCAGCTGAAGAAGTACTATCACTATCCAGTGATTCAGGTCTTTCCCGGCACTGGTA
ACGTGGTGCGTGTTTGA
>AY437474 1, 1290 MP44
ATGAAGCGGACATGTCGCTCTCTGCGAAGGTTTTCCGCAGGTCCGCTTGACTTAGCGCATTCGATCTGTA
GAAGCGCGGTGGTTTCTTCCTCTACTCGCGCTGCCTCATCACAAAATTCGGTCGCGCCGCGCACAGGAAG
CTCTTCCTTCGCGCGTCAGGCCGCGCGAATGGCACCGCCGCAGACAACTCTTTCACTGGACGCCAACCTG
CGCAACACCCTCAGAAGTCTGCTCATTGAGCGTCTGTGCAGTCCGACACAGCCCCTGCCGGCAAAGCAGT
TGTATGATGAGCTGACGAACCGCTCGCGTCTCGTGGAAGAGTTTCACTTCTCGAAGACGTCACGCCGCAT
CACCCGCCAGCGGCTGGCACTGCAGTTCTTGCGTCGATACAACGTGCTGTTGCACGGCCTCTTCTTTTAT
TCGGAGGGCACGCGCACGTGGAATACGACGACGGAGTTTCATCGTCTCGCCATCCTTGGAGAGTCTGTGC
TACTAACCGAAGTTCGCACTCGGCTGCTGAAGCTCTTCCCTGTCATGCCGTATGCGGCGTACGTGCAGAG
TTTGCCTCACATGGTCGGCGAGGAAGCGCTGGCAGCAGCTTTTGACCGGTATGAGATGCAGAACATTGTC
GGTGCCAAGCCATCTAACCAGCGCAGCGGCACGCCGCTCACTCGAATGCAGAAAAGCCATATGCTGTGCG
CTATTGTAGCGGAGATGTACTGGTTTACAGCTCGCACGAAGCCCACCGACTTGACGCACAACAACGCGCT
CTTCCCTCCATCTGACGTGCTCATCTTGCACGTGCTGTCCACCCACCTCCTCGAAAATTTGCCTGCAGAG
CTCATTTACAACGTCGTAGAGCCGATCGTGGCGGACATCAAGCGCGTATGGGTGAACGAGCCGATGTCGT
TGCCGTCACAACTGCGCCTAACCCCGCGGACTGTCGGTGCCCTCTCCCTCAATGCTGTCCCCGTCGATCT
CCGCTACACCGCCAAGGAAGACGCTGTCATGTCGCTCCAAAAAAAGCATGAGGAGGCTCATCGCCGACAA
CACGCGCTTACCCGCGAGCCGGACCACAGCTGCGTCAAGTCCTTCATGCGACCGTCGTGCAACTACCGCA
TATTTGACGGTCCACGGTATCAGATACTGAGCTCCGATAATCGCGTCGCACCACTACCGCTTGCACAAGA
GCGGAGCAGTCTTCCCGCTTCGGCTGTGTGCACCGTCGTTGACGATGCGGACAGCGCCGCACGTGCGATG
GAGCTTGCCAAGATGGCGGAGCAGTGGTGA
>AY437475 1, 1257 REL2
ATGCTTCGGCGATGCCTGCTTACACGGCTCGTGCGTCGCTCGCTGGTCCTCTTTTCGGACGAAAAAGAAG
CGCTGTTCGAGCGCTATAGCGAGATTGAAAACGCCCACTCACGACGCATTGAAGCACTGAAGGACGCGGG
TCTCTTCAATGATGAGTGGATTGCCACGGAGAAGGTGCATGGCGCCAACTTCGGCATCTACTCAACAGAG
CATGGGAAGACTATTCAGTACGCGAAGCGGAGCGGCATTATGCCGCCGTACGAGCACTTTTTTGGCTACC
ACATCCTCATACCTGATCTCGCGAGGTATGCCAAGCAAGGGCGAGAACTGCTTACAGAACAGCTCGGCAT
GTCACCCAGCACCGTAATCATCAACGGTGAGCTGTTTGGTGGCAAGTATGACCATCCAAGCGTGCCGAAG
CGGCGGCAGAACGTGATGGTGGCCGGTAGGCCACGCACCATCACGGCTGTGCAGAACAATGCCTTTCCTC
AGTACTGCCCAGACCTGCATTTCTATGCCTTTGACATCAAGTACCGGAAGACCGAGGAGGAGGAGTACTG
CACCATGACGTACGACCAAGCCTTGGCCATCTTCGAGAAAATTTCGGGCCTGCTCTACGCGAAGGCTATC
ATTCGAGGGCCGCTGAGCAAGGTGGCCGCGTTCGACGTGGAAAACTTCGTCACGACAATCCCGCCGCTGG
TGGGCATGGGCGACTACCCGCTCAAGGGGAACTGGGCGGAGGGCCTCGTTGTAAGACACTGCCGTCGCGG
CGAGCCTGACTTCGACGCAAAGGGCCTCACCATCATGAAGTTCAAGTGCACCGCCTTCCAGGAGATCTCG
ACCGACCGCCGCCAGGGACCGCGCGTAGACGCGCTGGAGGAGGTTCGGCGGCAGTCCATCAAAGTCTCCG
GTGTACAGCTGCCGGACATGGAGAGCGTCATCGGCGACCCCGAGGTGCTCCAAGCGGCGCATCGCCTGCT
CGACCACGTGTGTGAGAATCGCCTTCAGAGTGTCTTGTCCAAGATCGGTACAGGCCCGTTCGAGACGCAG
TCCATGACCCCCACAGAGCTCTCCACGCTGCTCGCAAAGGATGCGCTGAAAGACTTCTTGAAGGAGGCGG
ATGCAAAAATCGTCAACACGCCGCTACTCGTGCGCCGCGAGATGGTCCGCTACGTTGTTTTTGAGGCGCG
CAAGTACGTGGCCAGCAGATGGAAGGACGTCGTTGGGCAGCAAAAGGAGGCAGACGGGGCGTCGTGA

>AY437476 1, 1473 REL1
ATGCGTCGACTGGCACTGCGTCGCGCGCCACGCTGCAGTCATGCCACCCTGTGCAGCAGTCGCGGAGGCG
GCAGCGTTCTGAGGCGCCAGGCATCGGCGACCGCCATCTCTCCACCATCCCTCTCCACCACCACCACCAC
CACCACCTCGTCGCAGAGTCTCGCCACGGCTGCGCTCACAACGCCGCACCGCTGCTACATGCCACTTCCC
CGAAACCAAGAGGACTTCTCGGCCTACACGGAGATCGATCTGCCGACGGAGACGCGCATCGACGCGATCC
GGCGCACGGGCATTGCGAGCCAGGAGTGGGTGGCGTGCGAGAAGGTGCACGGCACAAACTTTTCCATCTA
CCTCATCAACGAAAGCGAGGTGCGGTTCGCGAAGCGCAGCGGCATCATGGACCCGAGCGAGAACTTCTTC
GGCTACCACCTCCTCATCGACGACTTCACGGCGCAGGTCCGCGCGCTGTGTGCGCTGCTGAAGCGCAAGT
ACGGCGTGACGGGCCGCATGGGCCGCGTCGTGCTGCACGGGGAGCTGTTCGGTGCCAAGTACAAGCATCC
GCTGGTGCCCAAGAGTACCAAGTGGTGCACGCTGCCGAATAAGAAGCGGATTCCGATCTCGGGGGTGGAG
ATACAGAGCGAGCCGTTCCCGCAGTACAGCCCGGAGCTGCACTACTTCGCATTCGACGTCAAGTACTCCG
TCTCGGGTGCCGAGGAAGATGTGGTGCTGCTGCCCTTCGACGACTTCACGGAGGTGTGCTCGCAGGTGCC
GAATCTCCTGTACGCGAAGCCGCTGGTGCGCGGCACGCTGGACGAGTGCCTCGCCTTCGACGTGGAGAAC
TTCATCACACCGCTGCCCGCGCTGCTCGGGCTGGGCAACTACCCGCTCGAAGGCAACCTCGCCGAGGGTG
TCGTCATCCGTCACGTGCGCCGCGGCGACCCGGCGGTGGAGAGCAGCGGGGTCTCGACAATCATCAAGCT
GCGCTGCTCTTCCTTCATGGAGCTCAAGCACCCCGGCAAGCAGCAGGAGCTGAAGGCGACGTTCCTCGAC
ACCGTGCGTGCCGGCGCGCTGCAGCGCGTGCGCAGAGGCAAGAAGGTGACGGTGCTGGCGGACTCGATGC
TGCCGAAGCTGGAGGCGGCCGCGAACGCGCTGCTGCTGAACAACGTCAGCGAGGGTCGTCTGAGCAACGT
GCTGTCGAAGATCGGCCGCGAGCCGCTGCTGACCGGCGACGTGAAGCAGGAAGATGTCGTGCTGATGCTC
GCGCAGGACGCCCTGAAGGACTTCCTGAAGGAGACGGACCCAGTGGTGCTGAACACGTCGCTGTCGTTTC
GCAAGACGCTGATCCGCAGCGTGTACTTCGCGGCGGAAGAGCTGCTGCAGGGCGAATGGAAGCGCGTCAT
GGACCGGTTGAAGGCGTCGCAGGCGGAGATCGACGCCGCAGCTGCCGCGCAGGAGAAGGCAGAGGCGCAG
TGA
>AY437477 1, 1533 RET2
ATGCACGCTCCTACGCCACTGTACCTCTACGTGGCCGTCACGCGCGGGTTGTGTTCCAGCGCGGTGGCGG
CGTCGAGCTCGGCTGACGCTGCATCTTCGCCGAGCGCGGCGTCCTCGTCGACGTCCGAGGGGGTCAGCTA
TGCCTCGCACCTCGTTGCTTCGAACGATCACTACGTACAGTGGGGTCGTGCACTGCTGGAGGAGAACAGC
AAGCGGATCGGTCCAGAGCAGATGTTTCGTACGGCCATTCGGGCCCAACAGCAGCTGCAGATGCTTGCCG
ACAAGTGGACACCGGATGCGAAAATTTACTGCTGCGGGTCGTTGGTAACGCACGGGCAGATGGAGTGGGG
CAGCGACCTCGACCTTGCCTGTGTCTTCGACGACCCGTACCCCGACCACTCCGTGCAGGCGAAGCGCACG
GAAAAGCTGTGGTCAGTTGTCAAGCGCTACATGCCGCAGTTTCTGCGCAACAATCTCTTGGCCCTCACGG
AGGCTCGCACCCCTGTCGTGAAACTGCGCTACGTCAACGACGACAAGGTCGCGCGGGGGCGGTACACCGT
GCTGACGGAGGAGGAGGACCGCAAGTCGCGCACGACCCTGTGGGACATACGCAACCGCCATCTGGATTCC
AAGGACCTGGAGTACATCGCCACAAAGATGGGGCGAGAGAATATCGAGGGCATCTGGGTGGAGCGGACGA
CGTATGGCTGCCGGTGCGCGGTGCAGTGCACGTCGCGCGAGCAAGCCGTCGGGGCTGTCGGCTTCTTCCC
AGATGGGGTGATCATGACCAAGGGCATGCGCGAGGACTACACGCGCGACATGCTGGACCTCCGATACGTG
CCGGAGATGTTTATGTACAAGTGGGATATTTCTTTTGTGGCTTACGGCGTCAAGAACAGCTACCTGCTCC
GCCACTATCTGCACAACGGCCCCCCAGCTGCCCGCCACGCAGCCATGGCCGCGAAGGCGTGGGGCAAGGC
CTCCGGCGTGGGGCTGGGGACGGCAGCCATGCTCACCTCCTACGCGGTGACGGTCATGTTTGTTCACTAC
CTCTTGGCAACTCGGCAGCTGGCCTGGATCGACCCGTGGTCGTTAGCCCATCCAGTCCACATGCCGCGCT
TCCCCGACTTCTCCCCACTTTTGGACTGCGACCCGGCCGAGCTCGGCAAGCTCCTGCACGGCTTCTTCAT
CTACTACGCGTCTCACTTCAACTATGAGAAGAACATTATCAGCCTCAGCCGCCCACGGCTTTCACTCCGC
TCCGACGTGAACTGGAACTTCCCGCAGAGCAAGCAGGGCACATTCAGCTACCTCCTCGGCATCGAGGACC
CGTACGAAGACGTCGGGGTTGGCGGACTGAACCTCGGTCGCCACCTGCATCCAGCTAAGTTCAACCAGGT
GAAGCAGGAATTCGCGCGTGCGGCCACCACGATGGAGCGCTACGTGCCGTCGAATGCGCCCGAAAAGACA

ATCCTCGGTGTCAGACGCGGGGGTACGTCGAACCACCATCATCACCATAACCGCTTCGATTAG
>AY437478 1, 1764 MP61
ATGAACCGCTGTGGTGTAAAGAGGCGCCTTTCACTGCGGCTGCCAGTGGTGTCAATCGCAGGCCTTCGGC
GGCGAGCGCGAAGAGCAGCAGGCACGCGTAGCGGCAGTGTTGCACTTTGTGTCTCTGCTGCGTCTATTGG
CAGTAGCACCAGCCTCGCTAGCACCCCTGGCCGGAAAGCTCCCGTGTACTACAACTTTGCCACGCCGGTG
AGTGTGCTACGTGACGCGGCGGGTAGTGAAAGTGTGTCTCACCGACGCCCGCGCCGCGAGGCGCCGGCTC
GAGAGGGAGGGCTGCTACTTGTGGCGCCGCGCACGCCGAACCCGAACGACCCGCTTCAACGGCTGAACAT
TTTTCGACGCGAGTTTTCCGATTTCAGCAGCGCCGCCAAGCTCAATCCGAATATCATGGTGAGCGTCGAC
ACCAGCACGCGCCGCTTCCTCGAAATGTTCTGTGACTTCGATGCCAAACGCTGCAAGCTGTGCAATGAGA
CGTTCTTGCAGTGGCACATCCACGCCAACGGCATTCCGCACGCGGGGCGAGAGGGGATGCTGCTGGAGCT
TGTTCGCCCGTATTGTGGAACTCCGGATGAACTCATAGAGATGTGGTGGCAGCGGCTGAACTCGTGTGCT
GCGTTTCACCGGATTCCCGGCTTGAGCCACGACAACCCACACGAGCGCAAGCGGCGTCTGCTATATCTGT
TGAAAGTGCTGAAAGACCGCGGCATCCTTGTAGAGACGTTCAACGTGAGTGACAACCAGTCCACCAACTC
AGCGCGCTCGTGGGAGTTTGAGCGCCTCGAGTTCGTGGGCGACAACGTGGTCAAGTACGTGTTGAACAAC
ATTATTTCCTGTGCATTCCCACCTCACGAGGGCGGCACAAAAGGAAAGATGACGTGCTTTCAGTTCGTGA
TGGACGGCAACGATGGTTTGGCACGCGGCTACGACCACCTCGACCTCCAGCAGCTCGCCTCCAGCCCTAG
GGTAGTCAGCAAATTTAAGAGCGACATTGTCGAGACCCTGTTCGCGGAGCTGCAGATGTACCTGTGGAGC
ACGCAGCACGATATTGGCACCTGTCCTCTTGTGTTTCCATTTACGAAGGATATTTACACCCTGCGGGCGC
TGGTACAGCATGTGCTGTACGAGCTGGGTATTGAACTCTTTCTCTACCACATTCGCCACATTAACGGCAT
GCTACAACGCGTGATGCGAGAGAATCACCTGCAGTTCGTAAAAGCAGATTCCGCGCTGAAGCCCAGGGCG
GGCGCCAGGACCGGGAGCTTGGGCGTGAGCACGCGCCGTGGTGACTGGAGCGATGACGACGATGACTACG
ACTGTCCGCAAAGCTACGGTACGCCGGCGGTGTCCGCCCCCACGGCAGCCCTCATGAAGGTGCGCTCGCA
GCGCACCCACGTGCAAGGCACCGGTGCGGCGCTGTTCTTTGCCTCCACAAACTACGATAACTTCAAGCGC
GTGGTACCGATCGGCGGTCTTTTGCCGCGGCCATTTGCGAGGCAGGATTTGTCAGTCATCCCAAACCACA
TGCCGCACCTCCGGAGCGATGGCGCGATGACGCGGAGGATGAGGAGGGTCGGGAATGCCGGTTGGTCGAC
CCTGCACGCCACAGCAACAGCTCTGACCACGTTGGAAAGCGTTGCCGACGGTCTCGCCCTCAGTGCCGAA
ACAACGCCTCGTGTGGGCAGCGAGCCAGTACGCTTGTCAGTTCCGCGTCTCAAGGACGAGGAGCTCATTC
CAGAGCTGATTTAA
>AY437479 1, 1872 MP63
ATGACGATGAAGAGGCGGATCGGTGTCTTGGCCGCTGCTGCAGCGAGCCGCTGCGCGCTGCGCCGGCTGC
ACCACGTGTCGTCTCGGCCCTTGCACGCGTCGGGTGCTGTCGCCTCCGCGAAAGGCGCCTCAAATGCCGC
TCTGTTGGGGGCTGTGCGGAATCAAAGTGCCGTCCCGGACCGGCGCTTCCACTGCAGCGTTTGCAAAAAG
TCGTTTCGCTTAGAGATGGCGGCAAAGCTGCACCTTCAGCAGGTGCACAGCGGTGAGGGCGCGGTGGAGG
CAGGCGCTGGTCCCGGACAGGGAGAGGATCAGGCGTCGCAGACCCCGGTGGGTGTATTTCGTAATGCGCC
TCCGCAGGCACCAACCGTCGTTACACCGATCGTGCCAGACACTCACGAGCGCGCTGCGCGCCGTGAGAAG
CCGGCACCAAAGCCACTTCACCAAGCCGAACGCGAGGTGCCAGGTATTGTGATGGAAAAGATGCTCTCTG
TATGGGATGACACAGGGGTGAAGCGGATGGGGGATCAGTTTGTTCACAGTAGCATGGTAATGCGCGTGTT
TGCCGCTCGCCCGTCTGACAGCGCCGAGCCGTTGTACGGCGTCATGAATCCAGAGGGTGAGAACCCGTTC
GAGGGTGGCCCGTATGCCCCCGACGTGGCTGGTTCTATCACGAAGGACGAGGTCAAATTTTACAACATCG
GTATAGGTGACGCGTTTGCGCTGGCGTGTGGCGAGTCTTTCGGTCCATTGCGTCCTGCTCGGTGCCCAAA
CCCATTCCTCCGAAAACTAGCAAAGGATGCTGCGAAAACCGCCCCAAGGGTGGGGTTGGCAGAGCAGCAA
ACGGCGCCGGTAACACCGTTCGGACAGCTCCCCATGTTTGGGCAGACTCCGTCTCCTGCTACGGCCCCAG
CTCCCAGCGCCGCCGAGGAACCGTCGACCACGATCAACAGCGTCGTCTCTTCCCCCTTCGCCGCCGGCGC
CAACGACTCGCCTTTTGCTGGGACGACGGACTTTCCGTTTGCTGGGCAAGGGCTCTCTCCGTTCGGATCG
GTGAAGGAGCCAGCAGATCAGGCCGCTGCCGAGCCCGGGATTGCCGCGCGATCGGCGGCCTCGTCGGAGG

CGCCGGCCTCACCGTTCGTGGCTGCATCTAGTGCTCTTCCTCCGTCCCCGTTCGCCGCAGCTCCGAGCCC
TTTCACCGAATCTTCTGGGGCAGCAGCCCCGTATGCTGCGAGGGACGCAGCCGCGGTGGCAAGCGGCGCT
GAGGGTCATGACGCCGCCTTCTTTTCCTCTTCCGCCGCGTCTTCCGTGAGCGCGCCGGCTGAGGTGGCGC
CCGCACCGACGCATGTGTGTACCGTTTGCAAGAAGAACTTTTCGACGTATGACGGCCTTCGAATGCATAG
CAAGGCCAAGCATGGGGAGGAGCTGCCGAAGGGGCGGCGAAACGGGAAGCGCGAGGTTCCGGACTTACCA
GCTTTCATTCCCAGTCCGGTGGACCTCACGATGACGTCACCCTTCGGCTCGTCGGCGCAACGGTCGGCGT
GGACGGAAGTGGAGCTGAAACCGTACGCGCAGTCTGTGAGTAACATTACTGTGGCGGGCCGCGTGCTGGA
TAGCGAAGTTTTGAGTGACGGGGCGTTGCTTTTGTCGCTTTTCGTCCGCGACTCGGCCGAAAGCGAGTCT
GAGGTAATTCCTGTTCGATGCACCTCAGCTGCCTTTAGAACGCTTGGCAGAACGCTCGTGTATGGCGATC
TCGTGTTCGCCTGTGGTTCGCTGCGCGTTTTGCCATTTACGGATAAGAAAACACAAAAGAGATACTCCTC
GGCAGTCGTGCACGTGGGTCTTCCAACTGGCATGATTGCCAAACTGAATTGA
>AY437480 1, 1896 MP67
ATGCGTCTTCGCCGCACTTCTGCGTGGCGAGCGGTTTGCCATATCCCTGATGCCTTGCGCACGCATCGTC
GTGACTTGGTTTCGGTAACATGTTCATCGTCATCGAGGCCACCGCTACGTGAGGTCACGAAGCTTTTTGC
CAGCCCCATGGTGCAGGAGTGGTGGGTGAGCGCTGCTCAAGAGGCGGTCATCTCCCGGAAAGCCACCAGT
CGGGCAGAGCGCATTGTGACGGAGGCCGACGATCCGCGCTCCGGTACGAGTCACTTCTACGATAGTGATG
CCAGACTCTGCCTCGTGTGCAACGCGCGTCTGGAGGGCAGCTACAGCGCGCACAGCCACAGCGTCGACCA
CAAACCGCGCGTTGCACTTTTGAAGCGCACAATCGGTATGATTCTCACATTTCTCGAGCAGGCCGGCTCA
TCACCGTCGCTGCCGCCGCCTCCGGAGTCGGACTCGCCGTACTCGTTGCCTGGCAGCAAACGCTTCTTAG
TGTCAGTGTCAACTCACACAGGAACACCTGCCGCCCCAGCCCCTTCTTGCTTCCACGCCTTGTATGCCGA
GTCACCTCTGTTTGCTGAAGACGGCGTGCCAAGTCGCGCGCTCCTCTATCACCGCCCGTGCACGCTGCGC
GAGTTTGACCTGGTAGACGTGATCATGCGGCGCTGGTGGAACACTTTGCATAATCCACCACCTAGGCGGG
GTAGTATTTCCTTTGACCGCCTCCTCTCGCTTTCCTCGGCTGAGCTGCAGATGCGGCGATGGCGTGTGCG
TTACCTACTCCACTTTCTCAAGTCGAGAGGGGTTCTCCGCTCTTGCCTAACACTGAGGTCCGACCAAGTG
AATGCGGCGCCGTCCCCCAACGAGCGTATTCACCGTGGTGAGGCCTTTGAGCGGCTCGAAATGGTCGGCG
ACTTTTTCTTCAAGAGCCTCACCCCCGACCGAATGCACACCGTCTTCCCGGCTGATGAGGGTGGACTTAC
AGCTGAACTCCAGTGCTTTGAGCGGGCCTTGGATTCGAATCACGGTCTGCTGGCCATCTATGACTATCTC
GGTCTGGATGACATAGTCGGCTGCTTTTTGGCCAACAACAAGGCAAAGGCCGACGTTGTAGAGGCCGTCA
TTGGTGAGCTGAAGGTTCTGTTGTGGTCGACTGAGGTGGTGTGGGGCATGGAGTACTACGCCGTACCTGG
GGAGCGCGCCACGCATGTCTACCTGCGAGCGTTGGTGGCGCACACCATCGCCGAGCTGGGGCACACGGTG
CTGATGTGGCAGCTGGAGAGTACACTACGCAATTGTCGCGACTTCCTTCTCAAGCACACCGTGGAGGAAT
ACTTGGGCGCCGAGAAAGGGAACGAGCGTCGTGCAAGCAGGGGAGGTGACGGGTGCGACGTCCCCGCGGA
CCTTCCAAAGTACTCACCGCTGCCGCCGTTGCTCGCGTGGAATCGGCGCCGCCTGGGCGTGTCGATGCGG
AGGGGTGAGGTGCCGCTCGCAAGCAAACAGTCTCGAAGCAGCATACCGCCGTCTCCTCAAGACCCGCTTC
GCAGACTCGCCCCGACTGCGAGCTGCATTCCGGAGGCTATTCGGGATCACTACGCCGTCGGTCGCAAAAA
GTGGAGCTCATATGCCTGTAAAACAGCCATTCAAGCCCTCTGCGCCTCCCTCACACAAACTGCGCTGCAG
CATGACAGCGGTCACACCGCTGACGCTCTATCGTTGAACTCATCGTCGGCATCCTTGCCCCTCTCTCCAG
AACCGCAGCCGTCCCCAACTACGCCGGCGTATGCGTGCGTCCCTCGCAGTGTGTTACGGGCTCTGGTGAC
TGCGAATTGCGTGGTGATTCGTAATTCGGCCCCGCCCCTCGCTAGAAGCACGTCGACTAGGAGGGTGGCT
GGGTAG
>AY437481 1, 2970 MP81
ATGCACACCGCCGTCCTTCGACAACTCAGCACACCATCGACTCGTCCCTCTGGCACCGCAAACGGCGGCT
TCGGCAGTAGCGGTGTCCGTGACGGCAGTGCACCAGCTCCTCCTAGCAGAGGTGACGGTGCTGCGACAGC
GCCGCAGCAGCGGTCGTCGCACGGCGTGAGCTCCGAGCTCTGCTTCTACGAGACGTTTATCTTGTCCGAT
GTCGACGTGCATCTTGCCCTCCTCGAGGAGGCGCACTACAAACACGGCGTGTGGCTCAACGTGCCACCGC

AGCTCGCGCCCTCCTTCCCGAACGTCGGCCCGCCCGCCGTGCCGGAGCCCATCTACCCATCGGCACAGCG
GGAGCGACTAGCGGCGATGACGGCAGTGCAGAACAAGCGCGGCCGAGGCGCCAGCGGCGGCGGCAGCACG
ACGGATGCGGAGCTCATAGTGGAGCCGTCGTGCCTTGCCTACCGCCCCACCCACGACCCGCCGCCCTTCT
CCACGACCGGCACGCGCTCGCTAAGGATTCCAGAGCAAAGGCGGGCGGCAGCAGAAGCAGCCGCCCCGGG
CGCAAGCGACGCCGTCCCCCGTGTGCCTCCGTCTGCACCGTTTTCCGTGACAGCGTCAACGGGAGAGGCG
AGCTTCGCGGAAGGCACCACCACTACTACAAGCAGCATGCGTGCGGAAGTACAGACAGTTGCCGTACGCA
TGCCGGTGGTCCCGTCAATGCTGTACCACTGCAGTGCGTGCAGTCGCCCGTTTAGGCGACGCGAGGCCGC
AGAGGAGCACGTGCAGCAGCGCCACGAGCCGCGCGGCAACTGCAGCGGCGTCAGCGCCGCTGCAGTTGTG
GTGGAGGGTCCGGGGCCTGGTGAGATTATCGGATACGAGGAGAAGGCCGTCGTCGTGGCGGCCACCACGA
CGGCCACCAGGACGGCCGCTGCTGCCCGCGTGACAAGTTCTTTGCCAATGAAAGCAGCAGCGAAGGCGAA
GGGGGTGGAGGCGAGCGCTGAAGTCGCGACCGCCAAGGGCGTCGCCTGCGCCTCGAGCACTGTCAAGCCC
TCCGCCTTTGATCTCGCTGCTAGCTACCGCGCGACACCACGAGTTGACCGCCCGTCGGACGATCTTATCG
ACGAACTCCTCGATGCCGTCTGGGACGACGTGGCGCTGGCGCGAGATGACATCGTGAAGCCGTCTGACCT
TCCCACAACACGAGCCGACCGTTTGCAACACCCGAAGCGGCGTTACGACAGCTGCCAAGGGCGCTGTTTC
ATCCCCAGTGTGCTCATCGTGGAGGGCGTCGCCGACAACCGCGCTGAGCTGGAGGCGGCGGTGGAGCGGG
CCGCTGTGCGAGCCACGCCAGATGGCGCCGCGCCAGGCATCAAGCGTCGGCCTTCGTCTCTGTTTTCGGT
GTTTTCGCCATCCACGATGGCCCCCCGGCTGCTCCCAACGCAGCGGCGCAACGCCGATGGCACGCTCGGT
GCTGGCGGCGCCGATGCCGCGGCCGCCGGCGGCGGCATCGGCGGTGGGACTCTTGGGGCGGCACCGACGG
CGCAGGAGTTGTCTGTAGCGGAGCTCAGCCGCCACTACCCGAACCCGTTCGGCGACAGCCCAAACGCTGT
GCTGATGGAGTTGGAAAAGGAGCCGATCAACCCCTTCATCGACCTCGAGGGTCAGGCGGCTGAGGTGGCC
GCCGCGGCGCAGTCGGGTGCGGCAGACGGTAGCACCGCTGACGACGCTGTCTCTTCCCCGGCCACGAAGG
AGATGGAGTGGCTGTCGCGGTGGGCAGCGAGGCCGTACGCGTGTCCGCTGTGCCAGCGACGCGCCTTGCC
AGACTTGATGAAAATCATGGCGCCGCTGCTGCCCCCGTCATTGGTAGCAGGCAGCGAGGCAGCCACCTCC
GCGGCGCCGACTGCCGGTGTATACGGTGACGTTCAAGGAGCCAGCCCACATCCGAGGTCTGGTACGAAGG
GGGCCGCTTCTACTGCTGCAGGGGAAACGTCGGCCGCGACTGCAGGCGCCTCCACGTCGAGGCCAGTAGA
GGGTGTGCCCCCCTCAGGCTCCCTCGAGGACGAACCCTTGGTTGCGGATGTTGAGGCGTGGAGCTGGTAC
GCCGAGCGGGTGCCGCGCTTCCGACTACTGGACTCCCTCGAAGACCACTTGCAGTCGAAGCACGCCGGCT
ACTGTGGCGGGGATGACGAGGCAGCGGCTGCACTCGACGATGACGATGGCTGCACTGGCAGAGACGGGGG
CGACACCCTCTCCGAGGCGGACTGGCAGTTCCTCTACCGCGTTGCCAGGCACCAACAGCTGCTAGCGCGA
GCCGAGTTGCTGGCGGTGCAGCAGGCGTACCGCGTCATGTGCCCCAAGCGGCACAGCACCGCTGCGGAGT
CCTTTAGCTCCATTGAGAGCGACGGCGCCGACCTCCCAAATCATGTCGGCGGCGATGCCGGCAAGGACGG
TGCCGCGAACGCGGCGATTCACGGCGCGGTGGGTGGCGCATCGGCGGGTGGGTCGCCCACGTCGGGGTCC
GCCATCGGTGCCACGACTGCCGATGGCACCGATGCCCCTCAGGTGCACGTGCGATCGGCTGTGAACACGG
TGCTGATCGGCACGGTGCGGGATGTGCAGGAGGGCTTCCTTGGCGCGACCCGCATTCTGCAGTACGTTCT
GGCAGTGCGGAACACCTCTGCGGAGTCGTTGGGCTCTGCATCCGCAGACGCGATGGGGAGCCACGTGGAC
GCCGGCAACGGAGAGGACGAAGGCGCAGGCGTGGACGAGGACCTGATCGTTGTGCGCTGTGTCGGCGACC
TCGTCCCTGTCGCGCTGCTGAAGCAGCAGGCCCGTTTGGGCTCCACCATTTTCGTTGCCGGGTCGCTGCG
TCTGAATCGCAACGTCGACACGGTGTCGCGACGGTCGCATGCGTACCCGTATGTGCAGGTGGTGCCGCCC
CTTGGCTGCGTGCGTGTCTTGGAGGCGTAG
>AY437482 1, 3069 MP90
ATGCCCACATCATCGTTACATCTCAGCTACACGCAGGCGATGCATTCCTCCGCAACGCACAGACTCGCAG
CGACAAGGGCACCAACCACCACGCTCCTCTGCACCGCACATGCTGTAAACCGCCTCCCCACAACGTGCAC
GATTCCCAGCCGCGACTGGGGTTCAGGCGGCGGTACGCTCTTCCACCCAACGCGAGCACTGAGGGCTCTC
AAACCAGTCACCGTGGCCCACTCCGACACGTTCGGCTCCTTTCGGCAGGCCAACAGAGCAGCGGCGGGGA
CGTGGTGGCGCAGCTCCACCGAAACGGCCTCCCGTGTGAAGGGCGGAAGGCTTGGCAGGGTGAAGGATCC

GTTTCAGGGCGTCTTTTTGCAACTGCAAGGCTGCACAGAGGTGGAGATGACGCGGCGAAGCCCCACTCCA
CCACCGGACTTCCCGCTGGACAGTCTACCCATCTACGAACCCCATGACCATAACGGGCCACCGCGCACCA
AAATCGGCTTCCACCTTCCCATGCGCTCGCTGCCGGCGTCTCTAGCCCTCGACTTTTTTTTCCCTGGTGC
AGGCTCTAACGATAGCCTGCAAGCAGCCAACGCCTCCAGCAGCAGCAGTAGGACCCATAGCAGTGGGGGG
TCTGCAAACAGTGACCTTGGCGCGCGCTACGAAGAAGCCGACAACGGTGCCGGCAGTGGGCATCAGCAGC
ACACCTCGAGGCTCGACCGCGCGGCGCACGGTACGAAAGGACTCTCCGCTGCGGGCGCTGGGAGTGTCAC
TGGCGACACATCCACGGCGTTCTGCCGTCTGTGTCGCGAAAGCTTCGATAATGATGGAACACTACGGTCA
CACATTACGGTCGCAGACCGGGCGCGGCACGTCTCTCACCCGGTGCGCGAGGTGGCGCTTGAAACGCTGA
CACTGCTGGCCGTGCGCGGATACCCCATCGACAATATAATGACGGTGTGGGCCGACATCCTGTTCAACTG
TCCACTGTTTCCGCGCATTCGCAGCATGACAGACCCGCGGTGGTCGATCGAGAAGCGGGCTGCTCAAATT
GGCATTATCCTGCGGGCTTTAAAGCGGGTTGGGGTGCTTGATGTCGCCCTTGCGACGGCAGAGCCGACGG
ACGTGGCGGGCTCGCTCAACTCGCGCTACCGGCAACGCCGCGTCGCCTTTGAACGACTGGAGTACATTGG
CGACAACTGCTGGGGCAACAATATTTCGAATCGGATCCTGTTGCTGTACCCTGATCAGCAGTGGCTGTAT
AGCGAGCGCTGCAGCACGTTCACGCTGGTGCGTGATGCATGTGAGATGAACGTCAATCTCGACTTTGTCT
TCGACACACTGGAGCTGTGGCACCTCTTGTCGACCACGGCACACTCGCGACTAGGGAGTGGAAAGGTGAA
GGCCGACCTTGTGGAAGGGATCTTTGGGGAGCTGCACCTGCACCTCTACGGTATGGTGCCTAAGCTGCAG
GATGATGTGGAGTACGTGGAGACGAACGGCGCTAGAGAGGTGCAGCTGGTCGCACTCGTGGAGCACTGCC
TGACAGAGCTATACGACCTCATCGTGCTGAAGCACGCACGCGACCTCGTCACATCAGCCATACCTCTCGC
GAAGGAGCTGGCGGCGAAGCGAATATGGGCGCGAACGCGCCCTTTTGTGCTGCCGCAGAAGCGTCCCAAC
ACTGGCCGGGCTCGAGGCAAGACTGCGATGAGCAACGGGGCGCCGCATGCATCAGCCTTTCCATCGGCTG
TTCCGACGGTCGGACGCGTTGCCACCGGCGGGTATACAGCGTCCTTTCCGGCACCACACACGGGAGTGGC
GACCGCAAATGCGTCTGTGGCTACGCTGGAGGACCTCTTCGATGTGTCACCTTCGTCCTTGCAGCAGCAG
CAGCAGCACGATTTGTCCGCATGCGGGGCGGCGAGTGGTACCATCGAGTCGCGCGCTGACGGGCGCACGG
AGGTAGCCGTGGTAAGCAACAGCGGTAAAGGTAGCAGGCATAACACGAAAGAGCGGGCCGAAGAAGCGGC
TGCAGTGGCGAGGGGGCGAGCCGGCGTGATGCCAATCTCTCCTTTATTCCCCACCCCCTTATCCCTCACG
CTGTCGGCCTCCATTGGTCTGGGTAGCACGCGTGTCCTGCCGGCGTTGCCGCGTCTCTTTACCGCTCCAC
ACGTGTACCCCGAACGCGTGCCCAACCCGCTGCGCCTCCTGCCGCTCTCGGCCATCCCAAGCATGACCTA
CTGTCACCACACGTGCTCGGACGTTTTTGCGAGGATGAAGATGAGTTACGAGCGCCTTGGTCTGCTTCAT
GAAGGTTCGAGCCACATGCACTATGTGGTAAGCCCTACTCCGCCGAGGTGGGCGGTGCTGATCGCCACAC
TTGTGCCGCAGCTCGTGGGATGCGCGCCGCTGCCTGTCAAGCCGACCGGTGACACAAAAGGGGCGTCGGC
CGAAGCGCGTCGGCTTTTAGCGTCCATGGATCAGGGCGGCCTGTACTGCCGGGACGCCCTCTACAATCTC
GCAGTGTTGCCGTCCTCGCCTGCTGCCGCCTCGCGACCCCACGCAACGCCGCCGCCGATGAGCACTGCGT
GGGCGCTGCAGAATGCCGGCTTGCCCGCCCTCTCTGCCACGCCGAGCAAGACAAGAGAGGACGACGACGA
CGCTTCCTCGGACAAAAGCGGGCGCAGCGGGTGCACCTTCGCTCCCTCCCGCTACGTCCCGCCCGGTATT
CGAGCGCCGCCGGCTGGCGTGGTGACAGACCGTAACCTCTGCCGCGGCGTCTTTGCCTTCCTGGCTGTCG
GTGTGGCTGACGCCGTGGCAGAGGCTGAGCGACTCACACGCACGGACACGGCGTCGGTCAGTCCCGCTAC
CCCGGCGGGCCCCACCACAGGTGCGCGGTCCTCTGGTGAGTGCCCGGCGCAGCACTCGGCAGAAGACAAC
TCGCCTAAAGCGTCGGCCACTGCGTACGCGCAGCGGATGAAGGCGGCAATGGACTACTGGCGGCGCCGCT
CGCAGACGTCCCTCCAGAGCTTATCGGCCGCATCCGGTGAGGCGTCACTGCCTCGCTAG
>AY437483 1, 2028 MP99
ATGTACGGCGTGACACGGTACTTGCGCTACATCGCCGGCGTAGGCGGCAGCGGTGGGATGGGGGGTATCA
GCAGCACCGGCACCAGCAGCAGTGGTGGTGATCTACGCGGTCCCGCAATCAGCTATCGCAACTCCCTCCA
AATCGCCGATGCCTTTCAGCCGGTGCAGCACCGCAATCAGTGGCCCTTCGATCACGTGCTCATCGACGTC
TCCTTCTTCGCCAACTCCCTCGGGTACCTCACACGCGACCTGGTCGGCCACGAACGCGACTGCGAAACAG
TGAAGAATGTCCACCGACAGCTGCAGACCGTCATCCTGCGCCGCCTCCAGCCGCGCAAGAGCCTCGCATT

CCTGCTAGACGGCAGCGAGCCGCTCTGGATGCTCGAGCATCGACGCCTCTTCCCTGGCCGACGGTACGAC
TCCAGGGTCTATCGTAGCTGTGCCTCGCCGATGCCCTACCTGCTGGAAGAGAAACTCCGCGGCACGGCCA
TGGAGCAGCGCACCCCGCCCTCGGAGGCCGTCATCAGCGGTCCAGCCACCCCCGGACTGGCAGAGGGCAA
GATAAGTGCGTATCTGCTGGACCTTGCAACCCGCACTCTCCGGCCTCCGACGTTCCCGCCGCTCTCCGAC
TCCACGGTCACGGCGAAGGACACAGTCTGCCTGGTCGGCGGGCCCGAGCTCGCTTGGTTGGCTGTTGGTA
TGACGCCGTTTCGCAACATCACGAGCGTGACGCTGCAGCAGGGGGAGCTGAAGAGCTGCTCGCTGCAGGA
ATCGATGGAGTGGATGCGGCTGGCTCACCTGCTGAAGGCGGCGCCAGAGCAGGTGAACGGCGACGGTGGC
GAGGCGTTGGCGCTGCAGCGGCGCCTGGCCGCTGTCCGTACGGATCTAGTTCTACTCGACCTTCTGGCCA
ATGGTCATCCCACTACAGGGCTGCCGCAGGTGCTGGCTACGCCGTTCGCGGACGTGCTGGACGCGTACGT
GGATATGGAGAAGGAACAGCACAGCGCCGCAGCGCCAAGCGCGCCGCAGAACGTCGGGCGCCTTCGTAGC
GTATTATTCGACGAAGAGCCCGTCACAGGCGTGCACCTTGAGCAGCCAAGTCTGCGACTGCGCTTAGTGT
CCCTGCAGCAGCTGCTCGTGCGCATCGTGCGGGCGTTGTCCGGAGCCCACGTTGGTCTGCCAGCCGGCAA
CCGCCCCACCCCCCACGCGGCCACGCTGCTGGAGATGACGCTGCAGACACACGGGCTGCTCTGCGGTGGC
GGCGTGCCGTCTCAAGCGTGGAGCCCCACGCCCGATCCGGCAGATGCCTCGAGTGGCGGCAGCAGCACCG
CCGCCTTGAACGGCACCGTGCTTCTGGACAAGTTCCCGAGGCTGTCGGCGGAGATGCTCCTACAGCACGT
GACTTACCTCCTCAGCAACGAAGCGAAGCAGCGAACGGCGAACGCGGCTGACGGTGCCGGCGAGGGCAGC
GACAGCATCTACCTGAGCCCGCAGCGCACACGGCACTTTGGGCTGACCGGCGTCGAGACGCTCCTCCTCA
GCGCAACGCAGGCAGAGCAGGTGAACCAAGTATTGCCCTTGTACGTTCGCGGCCACACCCTTCCTGACGA
GGTGGCGTCGGACATCACGCAGACACGCAACATCCACGAGGCCGTGCGCAAGACGCAGCGGGTGCTCAGT
CGCGTGCTGCAGCAGGCCAGTCAGGAACTGGCCAGTCAATCTGGCAGTGGCACCGCCGCGGTCGATGATG
CTGGCCACAATGGTCCGCACCCCGCTCTCGCCCATCTTCCGTCGCACTTGTTTGTGCGCACCGCCGGCGC
TCGCGGGCCACCGCCGGGGTGGGAGTACTACGGCGTGCACCTTGGCATCAAGGCAGAGGCCATGAACGTG
CGGTACAACCTCAACGCATCTGACACGGCGACGCTGCACCTCATTGACAGCAGCGGTAGCAGCAACAGCG
GTGGCGTCACCGCGACTGCAGGGAGCGAGGTTGGTGCGGAGTCGACCCGCAGTGGCATGCCCGGCTGA
>AY437484 1, 2982 MP100
ATGCGGGGTGCGCTGGCGCGTAGCGCATGTCGGCTCGCCTCCCTCCAGAAAGGGACGCAGCTAGCTGATG
TCTACCAGCTCCTCATCACCAAGAAGCCGGTCGAGTACGACTACATCGCGATTGACGTGAACGCCTTTGT
CGGCGGCGCCATGCGCATAACAAAGAACATGTCGCCAGAGCAGCGTCGCAGCAAGGAGGCTTCGCGGCAT
GTCCTCAACTCCATCATGCAGATTCTGCGGCGCGTGGTGTGCCGTCACTCTCTTCTTCTCGCCTTTGACG
GTCCGGATACGTTTGCGAAGGCGTACAAGCTTCGCACCACATCGTCGACACGGCGACTCGACATTCGAAC
TCAGCGCCTCCCCGGTGGTGCTTTGATGCGCGCGGTGGAGGATCGCATAGTCAAGGCGATGCCGCTGGGG
CGCGGGCTGATTCCTGGCGAGGTGGTCGTTTCCGGTGTCAACGTGGAGGGCCCGGTGGAGCGAAAGGTGA
CGGCATGGGCTCTCGACTTGGCGTGCCGCGACGGGGCGCAGCACACCTTCAAGTCTATCTGCCTCATCGG
AGCAGGGGAGCTGTGGATGAACGTGCTCGCCCTCACGCCGTACTTTCAAGGCACGAGCATCTTGCACGGA
TCGTCCGACTTGCGCCACATGACGCTCAACGACTCCCTGACATGGCTCCAGCTCGGCGACGACCTGCTGA
CCGGTGATGCACGGGTCATCACTGCAGCGCGGACAGATGCGCTGCTGCTTTATCTGCTGTGCCACGGCTG
CTCCGCAACAGACCTCTCGCCGCTGTCTGGATCAAACTTTACGGTTCTCATGGAAGCATACCACGCTCGA
CGCCGCGAAGCCGCAGCGACCGCCGGTGCGGCCGCCGGGGCACCGACCTTTCAGCTCATCCGCGGGCAGC
CGAACGGTGCCCTGATGCTTGACGTGCAGGCGCTCTACCTACTCTTTGTGGCGGAAAAGGACACTGCAGC
AGCAATCGAGTCGCTGGCGGCGCTTGTCGTGCCGGCGAAGGATGAGAGCCTACACGCGGCACCGCTGAGG
ACCTTCTCCATGCCGCTCTCCACTTCAGGCGGCAGTGCATCAGATGAGAAGATGCGCGACGCTAATATGC
CCGACATGTTGCACGGCAGCTACCTGAGCCACCTGCTTAACTCCCATCACCTCTTCTGCCATGGTGAAAT
GCTCGGTCGGTCCCGTGTGCCGGCCTACATGGAGGCAGAGGTGAAGATGGGGAGCCTTGACCGCAGCGGC
GCCGCGGGTGTCCGCGCCAGCGTGGTCACCGTGGGGGATTGGACCCGCTTTCTGCTCCGCTGTCTCCAAC
GCGGGGGGCGCTACGCTTTCGCCAGTGACGCAGGCGCTGAGCTGAGCTACAGCACCTCGAAGTGCGCACC

ATCCGCTGCGACGACTGCCGCTGCGAGCGCTGCGTCGTCACTCACCGCTCCCGGCACGACAGCCTACTAT
CCTTTCTGGTCCTCCGCGCAGCCGCTGACGGCAGCCGAGTACACCATCCTTAGCACCCCGAATCCAGGCA
TCCTCGATCCGCTACTAGTGGATTACGTGCCAGGGTTTACGCCGGCGCTACGGTCGGAGCTGCCGCGGGC
GCTCACGTCTGTGCAAGCGAACCTCCATGAACTTCGCAGCTCACTGCAGTACTTCTTCGCGTACGCAACA
GCTGATCGGCCGCACCCGTCACTGTGCCTGGCGCCGTCCTACCACTGGTGTCAAAACGAGAAGAGCAAGG
TGTGGTCAATGAGGTACGTGGACTTGGGCGTGGTCGCACGGCAGCAGGGTGTCCGTCACGCGCGCAGCCT
GCTGCCCGGCATGAGCATGGAGCAGGAAGCGCCGACGGAGGGCGCCGCGGTATATGACGCCGAATCACAA
ACGTGGACCTCGCAGCCACGCAGCTTCGCTGTCTTCACAGCGTCGGAGCAGCTACCGGGCCATCAGGAGC
AAGCGCATGCCAACGGGTCATCGTCTTCATCCATGGCGGCCACGGCTGCTGCTTCCGCGGATGCGGCCGA
GGTGTCCACGCTGAGGGTGCTCACGTGGAACGTTATGTTCGATCGGTACAGCAACCAGCCTACCCCACTC
GGTATGCCGGGCATCGACTGGTGCAGCCCGAAGCGGTACCCCGTGCTGGCAAAGCTTATCGACGCCGAGG
ACGCCGATGTGGTGGGGATGCAGGAAGTGGAGCGGCCGTTTGCTGAGTATCTCGCGGCGCAGCCGTGGTG
CCGCGAGCGGTACATCATGTCCTGCAGTCCGCAGAGCCCCATCTTGGATCCGTGGGGCGTGCTGCTACTG
GTGCGCCGCGGCGGGCGGTGGCCGCTGCAGCAGCTGAAACACCTGAATGTGCCGGCGTGGTCAGGCCACG
TCTCCCTCATGCCTGTGGCTACGCTGGATTTGAGTGCTGCCGCCGCCCACAGCGACAACAGTGGCACCGG
CTGTGGCAGCTCAGCGGGAAAGAGCGGCATCAGAAAGCTTGCAGCATCGCCGCGGGTGGTGAACGTCTGC
TCCATGCACTTGCTGGCGCCGTTTGTGAAGGTGAACGAGGTGGCCCGCACGGGTCAGGATCAAGCTCTGC
GTCATGCGCTGACGCGGCAGCTGCAGGGCGACACGCTCGTGATGGGCGACTTCAACGACTGGCCGAGCAA
CGAGTTCCTCATGCCGCCGGAGTCGCGGTACGTCGAGTGCTGGCCGATCATTCACCCCGGTGACTACGGG
AAAACAATGGACGAGTCGAACACCTTCTGCAAGCTGAAGATTGAAGAGATCTTCTTTGGTCGCTCGGACA
AGGTGTTCCTCCGGACAGGTACCGCCACAGCCTCGAGCCCCACCCGAGGCGTGCTGAAGCCAGTGGAGGC
GCATCTAGTAGGAACACGGAGCGTCAATGACGAAAACGGCAACCAAGAAGCACCGGCGTATCTGTTCCCA
TCTGACCACTACGGTGTCAGCATGCAGTTCCAAGTCCTGTGA
>AY437485 1, 1635 Hel61
ATGCGGCGTTTTGCAAGTGCCTTGTTTGGCAGGTGGGGTGCCGCACAGTGCGGGGTGGTGCCTTTTCGGG
CTCGCCCGACTCTTTTGCCCATGATGAGCAGGGTGCGCTGCTTCTCTGTGATTAGTGCTGCTTACGAGGG
ATTGGCCCCCGCAACGAGCTCGAATATCGGCGACCCGCATGCGCCACCGAAGACAAGGGCGTCTGCAGTG
TCAACAGAGCACGATGTCTCTATCACGGACGGCAATGGTGATCGTGTCGACGTCACCCCGCTAAACTCAT
TTGAAGAGTTGCGCGATGCACCGCGATGGCTGGCGGAGGGGTTGAAGACGCTCAAGTACCCTTCCACGAC
TGACATTCAGAAGTTCACAATACCTTTGCTGGCCAACGGCCACGATGTTATCGGACTTGCACCAACAGGC
TCGGGAAAGACGGTGGCGTTCGCGGTTCCAGCCCTGGCGGGGCTCAAGCCCAACCCCGATGGGACGCCGT
CTGTTCTGGTTCTAGCTCCCACGCGGGAGTTGGTGCAACAAACCACCAAGGTGTTTCAGAACCTTGGATG
TGGGCAGGTGCGCGTCTGCGAGGCCTACGGTGGTGCCCCGCGCGATCTACAGGCCCGCCATCTTCGCAAC
GGCTGCGACGCTCTCGTTGCCTGTCCAGGACGACTCAAGGACTTTCTCGACGGCGGCGACGTGTCCATCC
GTAATCTGTCGTTTCTGGTCTTTGACGAAGCAGATCGCCTTCTCGACATGGGGTTCCAGGTTCACTTGGA
TGAGATTATGGCCTACCTCGACTCAGCCTCACATCCGCAGACGATGATGTGGTCGGCGACGTGGCCAGAG
TCGGTGCAAGCGATGGCGCGCAAATATCTATCCGACGATCGTGTTTTGATTCGAGCCGGTACGGCCGGCG
CGGGGCTTCAAGTGAATGAGCGCATCAAGCAAGAGCTTATCTTCTGCCGAACCTTCACAGAGCGCATTGA
GAAGCTTGGGTCGTTGGTCGAGGATGGGACAATCGACGACAACAAGGACAAGCTCATCATCTTTGTGGAG
CGCCAGGCGGATACGGAAAACACAGCCAGGGCTTTCAGCCACCGGCTAGGAATCGATACACGCTACGTAG
GGACAATTCACGGCGGTCTGTCGCAGCGGCAGCGGGACAGGGTCATGAGCATGTTCAAGAGCAACCACAT
TCGCCTTCTCGTCGCGACCGATGTTGCTTCTCGAGGCCTAGACATTCCTGATGTAACGTGCGTGGTGAAC
TTTCAGGCTCCAAAAACCATTGACAGCTACTGCCATCGCATTGGCCGCACCGGCCGCGCCGGTCGCACCG
GCACGGCTTACACATTTCTTGGCGAAGAGGACGGTGGCCTCGCAACAGAACTCGTCAACTACTTGACTCG
TTGTCATGTTACTGCCCCAAAGAAGTTGACGCAGCTCGCCGAGAGCTACCAGCATCGGATGCAGCAGCAG

AGGCAGCGCTTCAGGCGTGTCGATCGAGGAGGTTTCTCCAGAAGTGAGAACAGCAGTGGGTTCGGCCGCC
GCCGCAGCGATCGAGGCGGTTCACGCGAGTTCATGCCTCGCAGGCCCGACTCGCGCAAGCGCGATGACGA
TCTTCCGTCCACGTTGGACTGGTGA
>AY437486 1, 1707 MP46
ATGCAGGCTCAGCTCGGGAGAGGACATGCGGCACTGCTGCGGCTGTACCGCGTCGTCGCCGCCCTGGTGT
CGACGGCGCCGATCTGGCGCCACCCGGCGACCGCTGCCAAAGGACCGAGTTACAGCGCGGATGTGATTGC
ACCACGCTTGAGTTTCGCCGGAAACCGGCGCGCCACCGTCTTCACCTCTTCATGCTGCCGCCTTACTCCT
GCGCGATGCATGACGACAGTGCCGGAGAGCACATGCGCGGCATCGTGTGAGTCGCAGCTTTGTCTGCTGT
GCGACACCCCCTACACCTCCTGGACGGAGCACTCCCGCGAGCATGCGCACGTGGCGCGCTCGGTGGTGTG
CCGTTTCTTTGTGATGCCAGAGAGAAGCGAGAGCATCATGCAGCACCTAGAGCGGCACATCGCTCTGGAC
CTCAAGGAGGTGGATGCCCTCACAGACCGCAAGGTAGGGCGGCGCCGCGAGCGTCTCCTCGCGGGCCTTG
TGCACCTTGTCGAGAAGGGCGTGCTGCGGGATAGCATACCCACGCTGCCGTTCTCAACGCCGGCACGCGG
GGGAACTGACAAGGGAAGGGGTGCTGGCATTAGCGGCGTGACGCTTGGGGTCGACGCTGACCTCTTTCTC
AACCGCGTCCTCGTCGGGGAGGCGTTCCTGCGCCGTGAGGTGCTTGACCGATGCGCGCGGCTCATGCCGT
CCCTCACAGCGATGGAGCTGAACAGCGTAGTGACGTACTTGACGTCGACGCGTCAAATGGCTCGCATCTT
TGACGAGCTGTCGCTGATAGAGCTTCTCGTGACGCGTTCGGGGTTGGAAAGCGCTCCCGCGAACGTGAAC
ACCGCCACCGCTGCTGCCACTGCCGCCGGAAGAGCGGCAGAGTCAGCGAGCGTAGAGCCGCTGAGCGAGA
CAGCTGCGGCACCTTTGCCAGACAGCGACGCGAGCACAGAGTCGCCACCTGCGCATCAGCCCTGCTCTTC
CACACTGGCCGCCAGTCTTCCCTCTCCCCCACGCTGGAGCCTGTCCCGCGAGGACAAGGTGTACATCATG
TGCGCCTGTCTCGGCGAGCTGCACCGCTTTCACGAGCAGGAGCGACCGCGCTCTGTCATCTCCAAGGCAG
CAGCAGATGCTATTGTGCTGAACGTGCTTGCCGGCCACGGGCGCGAGAACCTTGTGTCGGAGTTGGTGCA
TGAAACATTGCAGCGTATCGTGGACGAGGGGACGGTGGTCTGGCGGCAGCATCAGGAGGATGTGAAGCGG
CGCGGTCGTTCATCTGAAGCAAACGTGGCAGGGGCAGCTCGAATCAGCGCGCGGCGGGCACCTCGCACGG
ACAGTACCGTATCCGAGGACTTGTGCTACGTGCGCACTGCACCGCTGCCGTCAGCGCCGTCACCGCGGCC
GGCGTCTCAGGTTACACTGGATAGCGCAGCAGCGGAGCAGCTCGTGTCTTCTTACTTGCCCTTCAATGAT
GTCGATCAACTTAGCGGCGCCGAGACTGACGCTGTGAAAGCGACAGCAGAAGCGCCGTCGAGGTCGAGTC
CGGACCGCACCTGCACTACTTCTCCCGAGTGCATTTCAGTGCTGCACACTTCGGAGAAGGACTGGGGGCC
GCTCACGGCGGAGCTGCGGAAGCGTGCCGAGGATTGGAATCGGCTACCCTTTGTGCCTAGCACACCCGAG
CTAAAAGCGTCGAAGATGCCGCGCTAA
>AY462262 1, 1896 PDEA
ATGCTCGACTTTCTTGAGCAGCTGCAGCAGCTGGCAAGTGTGTACGCCATCTGCGGCAATGCTGTCTCCG
TGATGGCGGGGATGAGCGACACGGCAGAGGAGCTGACGTTCCGTTCCTACGACAGTTTAGAAGGCGCCTC
CTACATCTGCAACTTGAACGAGAAGGCTCTTCACACAGCGAAGGCCTCGCTCTGCGACAATGCCGATTGG
AGCAGCTTCTTTCGCGAGGTTCAGCTGGCATTCAACTCGGGTAGAGTAACCGTGCAGCCTGGAAATGCTC
ATCGAGTCGCCGTTAACGCCGCCGCGCCAGTTTCCGCCGACTCTAAAGGCCTAGCATGCTCGATGGAGGT
GCATTGCCTGTCGGGGTCAGGGGAGAAGTGTGCAACCTTTGTGCTTGAGCGAACCACGGAGGACCAGCAG
AAGTATATTCTCGAGAGCATGCTGCAGGCACATCACATGTATAACCACCCGAAGGAGTACGAGCAGAAGC
TGCTCCACATCATGGAAGCCAAGGAGATAGCGTGCACGAAGCGCGAGGCACTTGATCGTGAGCTGGTCGC
GCTGAACGATAATTTGACACGCAACAAGCACAAGCAGAAGATAAACAACGAGCGCAAAGAGGAGCTTCTA
AAGAAGCTCGGCGGTTACAGCACAGAGAACACGGGAAATCCGTGGCAGGCGATTCAGGAGCAGCAGCAGA
GGACAGCTGGCGAGAACACGAAGTCTCGGCTGCCGAGCCCGCTCGGGAACCGCACCTGCAAGGATTTTGA
TCTTGTCCTGTTTCGCATGATCAAGAGTCGGTGGCTGTCACCGGAGCAGTGCGACGCATCCTCGCCCGCG
AATCGCGTCGTGCAGCCGTACTCCAAGGAGGACCTCGCGATCCAGGTGAGCCAACTCTCAGGTAGCCGAG
CTGCGATATGGAAGGCACTGGATTCCATCGACTCATGGAGCTACCGCGTGTTTGATGTCCAGGCGGCTAT
GAGCGGTGACGACTACCTCTCGCTCTCGACGCAGACGCACGGCGGGTCTCTCCTGATAACCATGTACGCA

CTGCTGTGCATGCACGACTTTCTGCAAAAATTCAAGATTGACGAGCAAATTGCGCTCAACTGGATCAGCG
CAGTGGAGGCGAGTTACCATGGCAACCCGTATCACAACTCGATGCACGCCGCGGATGTGCTGCAGATTAC
GGACTTCATCATCACACAGGGAGGGTTGGCGAAGAGGTGCGACCTAAGCGACATCCAGGTCTTCTCTGCC
TTGCTGGCTGCCTCGATCCATGACTTCGACCACCCTGGCATCAACAATAACTTCCACATCAAGACAGGCA
GCTACCTTGCTACGCTGTACAACGATCGCAGTGTCCTGGAAAATCTGCACGTGAGCAGCGTTTTCGAGCT
CATGAAGAACCCAGCCTTCAACATCCTAGCCAGCTTCAGTGATGAGCAGCATCATGAGGTCCGCGAGACG
ATGATAGAGATGGTGCTGGCGACGGACATGGGCTCTCACGGAAAGTACGTGGCGAGTCTGAAGGGCAAGA
TGCAGGAGCACTCTAGTTTCACTCAGACCGCCGAGCAGAATCTCTGCCTCGCGATTGCTCTGAAAATGGC
CGACATTTCAAACTGCGGGCGCCCGCTTGACATTTACCTGCGCTGGGGAGCGAAGGTGTCGGATGAGTTC
TACCAGCAGGGCGACCGTGAGCGCAACCTGGGCCTCGAATGCAGTCCCTTCATGGATCGCCTTCAGCCGA
GCCTTGCGAAGAGCCAGATTGCCTTCATGAACTACATCATCACTCCCTTCTTTGAGCAGGTGGCCGAGCT
TCTGCCCGATATGCGCTTCGCGGTGGCTTTGGTGGAGGAAAACAAAGCGTACTGGGCCAACCACGACGAC
TCGTAG
>AY462263 1, 2793 PDEB1
ATGGCATATTTCACGGCCAAGAGGGCGTCCTCGATGGGTGCAAGCACCGTTCCGAGCGGCAACGACCATC
TCCTCGAGGCACTCACGCTGTGCGACTGCATTCTGAGCCGTTACAAGCGCTGCGGGGTTCAGCTCAACGA
CGCGGAGGCCGCCGCCTTCGCCAATTTGCAGCAGCGCATATCTAGCATCTCCGGCCACGCAGCAGACGCC
GCATGCCCAGCAGGCGCCACTCGAAAGCAGTCGCAGGAACTGGGACTGCACGAGTACGCTCAACTGGCGC
AGCGGTGCCTGATCTTCCAGAACCCCCTCGCCCAAATTGTCGCCACCATCAATGAAGAGTTCTCCAAACT
TGTGAGCTGTTCAGTGCGCACGCACTACGCCAACACAAACGACGCGGTGCTATGCGACCCAGTGCACGAC
ACTGTTGCAACCATCGATACATCGACCCCCATCGGCAGGTGTGCGAAGACGAAGACGACCGTTACCATTT
CCGACACGGTGTACATCCCCCTATGTTACAACAGCCACGTCGTCGGCTGCCTGGAGGTGGAGAGCACCGC
AATCGACACGAGCACGCCGTTTTTCGGGTACCTGCTTCAAGTGGCGGCACTAACACTGCAGAACGCTACC
TCCATCGATACACTGCGGTGGGAGACTCGGAAGGCAGAGGCCATGGTGGGCATGGCGACACGGCTTGCTC
GAGACACGTTGGAGGAGTCGGTGCTGGTGCAGTCCATCATCAACACGGCAAAGACGCTGACGGAGAGCGA
CCGGTGTAGCATCTTCCTGGTGAAAGCGGACGGCAGCCTGGAGGCGCACTTCGAGGACGGCAACGTTGTG
GTGCTGCCTGCGGGGACGGGCATCGCAGGTCACGTTGCGGAATCTGGCGCCGTGGTGAACATCCCGAACG
CGTACGAGGACGACCGGTTCCACCGGTCCGTGGACAAGGTGACTGGCTACCACACGCGCACGATCTTGTG
TCTGCCGATCGCGTTCGAGGGCACGATCGTTGCCGTTGCGCAGCTGATCAACAAGCTGGACATGGTGACA
CAGAGCGGGCAGCGGCTTCCGCGCGTGTTTGGACGGCGCGACGAGGAGCTGTTCGAGACGTTCTCGATGT
TCGCTGCGGCGTCGCTGCGCAACTGCCGCATCAACGAGACGCTGCTGAAGGAGAAGAAGAAGAGCGACGC
GATCCTGGACGTTGTGGCGCTGCTGTCGAATACGGACATCCGCGATGTGGACAGCATTGTGCGGCACGTG
CTGCACGGCGCGAAGAAGCTGCTGAACGCGGACAGGTCATCGATGTTTCTGCTGGATAAGGAGCGCAATG
AGCTGTACAGTAAGATGGCGGACAGCGCGAACGAGATCCGGTTTCCCTGCGGGCAAGGCATTGCCGGCAC
TGTTGCCGAGTCCGGCGTTGGCGAGAATATCATGGACGCGTACGCTGACTCGCGCTTCAACAGCGCTGTG
GACCGGCAGCTGGGCTACCGCACACAGTCCATCCTGTGCGAGCCGATTACGCTGAATGGCGAGGTGCTTG
CCGTGGTGCAGCTCGTCAACAAGCTCGGCGACGACGGTAGCGTGACCTGCTTTACACCCACTGATCAAGA
GACGTTTAAAGTGTTCTCGCTGTTTGCGGGCATCTCGATCAACAACAGCCATCTGCTGGAGTTCGCGGTG
AACGCAGGTCGTGAGGCGATGACCTTGAACCTGCAGCGTAACAGCATTACAGCGCAGCGTGCTCCGAAGA
GTGTGAAGGTGATCGCGGTGACGCCGGAGGAGCGTGAGGCAGTGATGTCGATCGACTTCGGGGGCGCATA
TGACTTCACTTCACCGGGCTTCAACCTGTTTGAAGTGCGCGAGAAGTACAGCGAGCCGATGGATGCGGCT
GCCGGTGTTGTGTATAACCTGCTATGGAACAGTGGTCTACCCGAGAAGTTTGGCTGCCGTGAGCAGACAC
TGCTGAACTTCATCTTGCAGTGCCGCCGCAGGTACCGCCGAGTGCCGTACCACAACTTCTACCACGTCGT
GGACGTGTGCCAGACGCTGCACACGTACTTGTACACAGGCAAGGCGTCGGAGCTCCTGACAGAGCTGGAG
TGCTACGTGCTGCTCGTGACGGCACTGGTGCACGATCTTGACCACATGGGCGTGAACAACAGCTTCTACC

TGAAGACGGACTCGCCGCTAGGCATCCTCTCCAGCGCGAGCGGGAACAACTCCGTGCTGGAGGTGCACCA
CTGCAGCCTCGCCATCGAGATTCTGTCCGACCCCGCCGCGGACGTGTTCGAGGGGCTGAGCGGGCAGGAC
GTTGCGTATGCGTACCGCGCGCTGATCGATTGCGTGCTGGCCACTGATATGGCTCGCCACGGGGACTTGT
CGAGGGTTTTCGATGATATGGCGAAGGCCGGCTACGACTCTAACGATCAGGAATCTCGTCGCCTGGTGAT
GGAAACGCTGATCAAGGCCGGTGACGTGTCGAATGTGACGAAACCGTTCGAGACGTCGCGCATGTGGGCG
ATGGCTGTGACGGAGGAGTTCTACCGTCAGGGTGACATGGAGAAGGAGAAGGGCGTGGAGGTGCTGCCGA
TGTTTGACCGGTCGAAGAACAACGAGCTGGCGCGTGGGCAGATTGGCTTCATCGACTTCGTAGCTGGCAA
GTTCTTCCGGGATATTGTGGGCAACCTATTTCATGGAATGCAGTGGTGTGTGGACACGGTAAACTCCAAC
CGCGCAAAGTGGCAAGAGATCCTGGATGGCCGCCGCGACTCCATCCGACCCTCGATTGTTTAA
>AY462264 1, 2823 PDEB2
ATGCATTCAGCGGTCTTTTCCCCCGATGCGCCGTATTGCGGCGCAGCCGGCTCCAATCACCTATGTGAGG
CGGTTGCACTCTGCCAGTCGATTCTGGCGCGCTACCGTCGGACTGGTACATCCTTCTCCTCCACAGAGCT
GAAGGCGATTCAAGCCCTGCGCACCGAGTTCCCTGATACCGCGCAAGAGCCGGCTGCGAATAGCGCGGCT
TCACCCGACCAGACCACGAAGGACTTCCTGAGCATTCTTGACGATGCAACCGACGTGCCGCACAACCCAC
AGAACGACATTGTCGCGTTTGTGGAGGAGTGCTGCGACAACACCAAGGAGCCAACAGTATTGTTTGCGGC
GATTAATGAGCGCATTTCAGCCGTGACTTGCTCGCGTAATGTCCGCACGTACATGGTGATCGCAAACGAC
AATCTCCTATGGGACCCTGTCAACGGTGTTGCCGCCCTTATCGACGACGTCACGCCCTTGGGCAAGTGCG
CGCAAGCGCGCAACATGCTGACGATTGCCAACACCCTATACATCCCCCTCTGGTTTCGGTCCGAGCTCGT
TGGCTGCGTGGAGGTGCCGGGTGCCTGCATCCCAAGGGACAAGGCTACCTGTGCTCAGCTTCTGCTCCGG
TGCGTCACCGTTGCCGTTCGAAACAGCATCAACATCTCCATCAGAAAGAGGGAAGCAAATAAGATCGAGG
CCATGGTGGGCATGGCGACACGGCTTGCTCGAGACACGTTGGAGGAGTCGGTGCTGGTGCAGTCCATCAT
CAACACGGCAAAGACGCTGACGGAGAGCGACCGGTGTAGCATCTTCCTGGTGAAAGCGGACGGCAGCCTG
GAGGCGCACTTCGAGGACGGCAACGTTGTGGTGCTGCCTGCGGGGACGGGCATCGCAGGTCACGTTGCGG
AATCTGGCGCCGTGGTGAACATCCCGAACGCGTACGAGGACGACCGGTTCCACCGGTCCGTGGACAAGGT
GACTGGCTACCACACGCGCACGATCTTGTGTCTGCCGATCGCGTTCGAGGGCACGATCGTTGCCGTTGCG
CAGCTGATCAACAAGCTGGACATGGTGACACAGAGCGGGCAGCGGCTTCCGCGCGTGTTTGGACGGCGCG
ACGAGGAGCTGTTCGAGACGTTCTCGATGTTCGCTGCGGCGTCGCTGCGCAACTGCCGCATCAACGAGAC
GCTGCTGAAGGAGAAGAAGAAGAGCGACGCGATCCTGGACGTTGTGGCGCTGCTGTCGAATACGGACATC
CGCGATGTGGACAGCATTGTGCGGCACGTGCTGCACGGCGCGAAGAAGCTGCTGAACGCGGACAGGTCAT
CGATGTTTCTGCTGGATAAGGAGCGCAATGAGCTGTACAGTAAGATGGCGGACAGCGCGAACGAGATCCG
GTTTCCCTGCGGGCAAGGCATTGCCGGCACTGTTGCCGAGTCCGGCGTTGGCGAGAATATCATGGACGCG
TACGCTGACTCGCGCTTCAACAGCGCTGTGGACCGGCAGCTGGGCTACCGCACACAGTCCATCCTGTGCG
AGCCGATTACGCTGAATGGCGAGGTGCTTGCCGTGGTGCAGCTCGTCAACAAGCTCGGCGACGACGGTAG
CGTGACCTGCTTTACACCCATGGACCGGGAAACGTTCCAAGTGTTCTCGCTGTTTGCGGGCATCTCGATC
AACAACAGCCATCTGCTGGAGTTCGCGGTGAACGCAGGTCGTGAGGCGATGACCTTGAGCCTGCAGCGCA
ACAGCATTACAGCGCAGCGTGCTCCGAAGAGTGTGAAGGTGATCGCGGTGACGCCGGAGGAGCGTGAGGC
AGTGATGTCGATCGACTTCGGGGGCGCATATGACTTCACTTCACCGGGCTTCAACCTGTTTGAAGTGCGC
GAGAAGTACAGCGAGCCGATGGATGCGGCTGCCGGTGTTGTGTATAACCTGCTATGGAACAGTGGTCTAC
CCGAGAAGTTTGGCTGCCGTGAGCAGACACTGCTGAACTTCATCTTGCAGTGCCGCCGCAGGTACCGCCG
AGTGCCGTACCACAACTTCTACCACGTCGTGGACGTGTGCCAGACGCTGCACACGTACTTGTACACAGGC
AAGGCGTCGGAGCTCCTGACAGAGCTGGAGTGCTACGTGCTGCTCGTGACGGCACTGGTGCACGATCTTG
ACCACATGGGCGTGAACAACAGCTTCTACCTGAAGACGGACTCGCCGCTAGGCATCCTCTCCAGCGCGAG
CGGGAACAACTCCGTGCTGGAGGTGCACCACTGCAGCCTCGCCATCGAGATTCTGTCCGACCCCGCCGCG
GACGTGTTCGAGGGGCTGAGCGGGCAGGACGTTGCGTATGCGTACCGCGCGCTGATCGATTGCGTGCTGG
CCACTGATATGGCGAAGCACGCTGACGCGCTAAGTCGCTTCACAGAGTTGGCGACAAGCGGGTTTGAGAA

AGACAACGACACCCACCGTCGCCTGGTGATGGAAACGCTGATCAAGGCCGGTGACGTGTCGAATGTGACG
AAACCGTTCGAGACGTCGCGCATGTGGGCGATGGCTGTGACGGAGGAGTTCTACCGTCAGGGTGACATGG
AGAAGGAGAAGGGCGTGGAGGTGCTGCCGATGTTTGACCGGTCGAAGAACAACGAGCTGGCGCGTGGGCA
GATTGGCTTCATCGACTTCGTAGCTGGCAAGTTCTTCCGGGATATTGTGGGCAACCTATTTCATGGAATG
CAGTGGTGTGTGGACACGGTAAACTCCAACCGCGCAAAGTGGCAAGAGATCCTGGATGGCCGCCGCGACT
CCATCCGATCCTCGATTGTTTAA
>AY484446 1, 513 GP63
ATGTCCGTGGACAGCACCAGCACGCACCGGCGTCGCTGCGTCGCCGCGCGCCTGGTGCGCCTCGCGGCTG
CCGGCGCCGCAGTCACCGTTGCTGTCGGCACCGCGGCCGCGTGGGCACACGCCGGTGCGCTGCAGCACCG
CTGCGTCCACGACGCGATGCAGGCACGCGTGCGGCAGTCGGTGGCGGACCACCACAAGGCCCCCGGCGCG
GTGTCCGCGGTGGGTCTGCCGTACGTTACTCTCGACGCCGCGCACACCGCGGCCGCCGCCGATCCCAGGC
CGGGCAGCGCGCGCAGCGTCGTGCGCGACGTGAACTGGGGCGCGCTGCGCATCGCCGTCTCCACCGAGGA
CCTCACCGACCCCGCCTACCACTGCGCTCGCGTCGGGCAGCATGTCAAAGACCACGCCGGCGCCATCGTC
ACCTGCACCGCCGAGGACATCCTCACCAACGAGAAGCGCGACATCCTGGTCAAGCACCTCATCCCGCAGG
CGGTGCAGCTGCACACGGAGCGG
>AY490814 1, 279 kinetoplast membrane protein 11
ATGGCCACCACGTACGAGGAGTTCTCGGCGAAGCTGGACCGCCTGGATGAGGAGTTCAACAGGAAGATGC
AGGAACAGAACGCCAAGTTCTTTGCGGACAAGCCGGATGAGTCGACGCTGTCGCCCGAGATGAAGGAGCA
CTACGAGAAGTTCGAGCGCATGATCAAGGAGCACACAGAGAAGTTCAACAAGAAGATGCACGAGCACTCG
GAGCACTTCAAGCAGAAGTTCGCCGAGCTGCTCGAGCAGCAGAAGGCTGCGCAGTACCCGTCCAAGTAA
>BN000109 1, 630 tag
ATGGATATTCCGGGACTGACGACGGATGAGAATGGCCTGACGCGCTGTACATGGCGCACGGCAGCAGCGG
AATACCCCGATTACCACGACCATGAATGGGGCGTGCCGGTGGCCGATGACATTGCGCTGTACGAGAAGAT
TTGCCTGGAGGGATTTCAGGCGGGCATGGCGTGGATCACCATCCTGCGCAAGCGCGAGGCGTTTCGGCGG
GCGTTCGAGGGCTTTGATTTTCGCCGGGTGGCGCAGTACACCGAGCAGGATATCGAGCGGCTGATGGCCG
ACCCGGCCATCGTGCGTAATCGCGCCAAGATCGTCTCCACCATCAACAACGCCCGGCGGGCCTGTGAGCT
GGTGGATGAAACCGGATCGCTGGCGGCGTGGCTGTGGTCGTTTGAACCGTCCGCCGACGAGCGCCCGGCG
CAGGTCGACCTGGCGTACTGGACGGCCAACCCGACCTCGGCCGCTTCAACACGCTTGTCCAACGCCCTGA
AAAAACGCGGCTGGAGCTACGTGGGGCCGACCACCATGTATGCGTTCATGCAGGCGATGGGCATGGTCAA
CGACCACCTTGAAGGCTGTGCCTGCCGGCCTCGAATCCAACACCTGCGCCACCACTTCAAACGACCCTGA

>J04483 105, 959 P100/11E
ATGGCTGGCGTTGATAAGGCAATGGTCACGCTGAGCAACGGCGTCAAGATGCCGCAGTTCGGTCTCGGCG
TGTGGCAGTCCCCCGCCGGCGAGGTAACCGAGAACGCTGTCAACTGGGCGCTGTGTGCCGGCTACCGCCA
CATCGACACGGCAGCCATCTACAAGAACGAGGAGAGCGTCGGGGCTGGGCTGCGTGCCTCTGGTGTACCG
CGCGAGGACGTGTTCATCACGACGAAGCTCTGGAACACGGAGCAGGGCTACGAGAGCACGCTTGCGGCCT
TCGAGGAGAGTCGACAGAAGCTCGGTGTCGACTACATTGATCTCTACCTCATCCACTGGCCGCGCGGCAA
AGATATCCTGTCGAAAGAGGGCAAGAAGTACCTGGATTCGTGGCGCGCCTTCGAGCAGCTCTACAAGGAG
AAGAAGGTGCGGGCGATTGGAGTGTCGAACTTTCACATCCACCATCTGGAGGACGTGCTTGCAATGTGCA
CTGTGACGCCAATGGTGAACCAGGTGGAGCTTCACCCGTTGAACAATCAGGCCGATCTGCGGGCCTTCTG
CGACGCCAAGCAAATCAAAGTTGAGGCTTGGTCGCCGCTAGGGCAGGGCAAGCTGCTCTCCAACCCGATC
CTCTCCGCAATCGGTGCAAAGTACAATAAGACGGCCGCGCAGGTGATCCTCCGCTGGAACATCCAGAAGA
ATCTCATCACGATCCCCAAGTCAGTCCACAGGGAGCGTATCGAGGAGAACGCGGACATCTTCGACTTTGA
GCTCGGCGCCGAGGACGTGATGAGTATCGACGCTCTCAACACGAACTCGCGCTACGGCCCCGACCCTGAT
GAGGCGCAGTTCTAA

>L01699 562, 1428 PTR1
ATGACTGCTCCGACCGTGCCGGTGGCGTTGGTAACAGGCGCCGCGAAGCGTCTTGGCCGCAGTATCGCTG
AGGGACTCCACGCGGAGGGGTACGCTGTCTGCTTGCACTATCATCGCTCTGCTGCAGAAGCGAACGCACT
ATCCGCGACGCTCAACGCAAGGCGACCGAACAGCGCCATCACGGTGCAGGCGGATCTGAGCAACGTTGCC
ACAGCCCCGGTCAGCGGCGCTGATGGCTCTGCACCTGTTACCCTCTTCACGCGCTGTGCTGAGTTGGTGG
CTGCGTGCTACACCCACTGGGGACGCTGCGACGTGCTAGTGAACAACGCCTCTTCTTTCTACCCCACGCC
GCTGCTGAGGAATGACGAGGATGGACACGAGCCCTGTGTCGGAGATAGAGAGGCAATGGAGACGGCCACC
GCTGACCTCTTCGGCTCCAACGCGATAGCGCCCTACTTCTTGATTAAGGCGTTCGCGCATCGCTTCGCGG
GCACCCCAGCCAAGCATCGCGGCACCAACTACTCCATCATCAACATGGTCGACGCCATGACGAACCAGCC
TCTTCTCGGGTACACCATATATACCATGGCCAAAGGGGCGTTGGAGGGGCTGACACGGTCTGCCGCGCTG
GAGCTTGCGCCGCTGCAGATTCGAGTGAACGGCGTTGGTCCGGGTTTGTCGGTGCTCGTCGATGACATGC
CCCCTGCTGTGTGGGAGGGCCACCGCAGCAAGGTGCCTCTGTACCAGCGCGATTCCTCCGCCGCAGAGGT
GAGCGACGTTGTTATCTTTCTGTGCTCCTCCAAGGCCAAGTACATCACCGGCACCTGTGTCAAAGTGGAT
GGTGGCTACAGCCTTACCCGGGCCTGA
>L12031 75, 677 YPT
ATGACCGCTGAGTACGACTACCTCTTCAAGCTGCTGCTGATCGGCGACACGGGTGTCGGTAAGTCCTGCC
TGCTCCTCCGCTTCGCCGATGACAGCTACACCGATAGCTACATCTCCACCATTGGTGTCGACTTCAAGAT
TCGCACGCTGAACCTGGAAAGCAAGGTAATCAAGCTGCAGATTTGGGATACCGCCGGCCAGGAGCGCTTC
CGCACCATCACGAGTAGCTACTACCGTGGTGCCCACGGCATCATCATAGTGTATGACACGACCGACATGG
AGAGTTTCAACAACGTCAAGACGTGGCTAAGCGAAATCGAAAAGTACGCAAGCGAGAACGTCAACAAGAT
CCTCGTCGGCAACAAGTGCGATTTGGTCACAAAGAAGGCCGTCGACACACAGATGGCGAAAGACTTTGCC
GACTCTCTTGGCATCCCGTTCCTTGAAACGAGCGCCAAGAACTCCACAAACGTCGAGGAAGCCTTCATCC
AAATGGCATCTGGAATCAAGGCGCGCCTGGCCGTGAGCGGTGAGGCCAAATCGGCGGCCCGCCCCAACCT
GCAGAACCCCCCAACGGTGAAGAAGGAAGACAGCTGCTGCTGA
>L14605_1 1545, 3500 heat shock protein 70
ATGACGTTCGACGGCGCCATCGGCATCGACCTGGGCACGACGTACTCGTGCGTGGGCGTGTGGCAGAACG
ACCGCGTGGAAATCATCGCGAACGATCAGGGCAACCGCACGACACCGTCGTACGTTGCGTTCACGGACTC
GGAGCGCTTGATCGGCGATGCCGCAAAGAACCAGGTGGCCATGAACCCGCACAACACGGTGTTCGATGCG
AAGCGCCTGATTGGTCGCAAGTTCAACGACTCGGTTGTGCAGTCGGACATGAAGCACTGGCCGTTCAAGG
TGACGACGAAGGGTGACGACAAGCCCGTGATTTCGGTGCAGTACCGCGGCGAAGAGAAAACCTTCACGCC
GGAGAAGATCAGCTCGATGGTGCTGCTGAAGATGAAGGAGACGGCGGAGGCGTACCTGGGCAAGCAGGTG
AAGAAGGCCGTGGTGACGGTGCCGGCGTACTTCAACGACTCGCAGCGCCAGGCCACGAAGGACGCCGGCA
CGATTTCTGGGCTGGAGGTGTTGCGCATCATCAACGAGCCGACGGCGGCGGCCATCGCGTACGGCCTGGA
CAAGGGCGACGACGGCAAGGAGCGCAACGTGCTGATCTTCGACCTTGGCGGCGGCACGTTCGATGTGACG
CTGCTGACCATCGACGGCGGCATCTTCGAGGTGAAGGCGACGAACGGCGACACGCACCTTGGCGGCGAGG
ACTTCGACAACCGCCTCGTCACGTTCTTCACCGAGGAGTTCAAGCGCAAGAACAAGGGCAAGAACCTGGC
GTCGAGCCACCGCTCGCTGCGCCGTCTGCGCACGGCGTGCGAGCGCGCGAAGCGCACGCTGTCGTCCGCG
ACGCAGGCGACGATCGAGATCGACGCGCTGTTCGATAACGTTGACTTCCAGGCCACCATCAACCGCGCGC
GCTTCGAGGAGCTGTGCGGCGACCTGTTCCGCAGCACGATTCAGCCGGTGGAGCGCGTGCTGCAGGACGC
AAAGATGGACAAGCGCTCCGTGCACGACGTGGTGCTGGTGGGCGGGTCCACGCGCATCCCGAAGGTGCAG
TCCCTCGTGTCGGACTTCTTCGGCGGCAAGGAGCTGAACAAGAGCATCAACCCCGACGAGGCTGTCGCGT
ACGGCGCTGCGGTGCAGGCCTTCATCCTGACGGGCGGCAAGAGCAAGCAGACGGAGGGCCTGCTGCTGCT
GGACGTAACGCCGCTGACGCTGGGCATCGAGACGGCCGGCGGCGTGATGACGGCGCTGATCAAGCGCAAC
ACAACGATCCCGACCAAGAAGAGCCAGATCTTCTCGACGTACGCGGACAACCAGCCCGGTGTGCACATCC
AGGTCTTCGAGGGCGAGCGCGCGATGACGAAGGACTGCCACCTGCTGGGCACGTTCGACCTGTCCGGCAT

CCCGCCGGCGCCGCGCGGCGTGCCGCAGATCGAGGTGACGTTCGACCTGGACGCAAACGGCATCCTGAAC
GTGTCCGCGGAGGAGAAGGGCACCGGCAAGCGCAACCAGATCACCATCACCAACGACAAGGGCCGTCTGA
GCAAGGACGAGATCGAGCGCATGGTGAACGACGCAATGAAGTACGAGGCGGACGACAAGGCGCAGGCCGA
CCGCGTGGAGGCAAAGAACGGTCTGGAGAACTACGCGTACTCGATGAAGAACACGCTCGGCGACTCGAAC
GTGTCCGGCAAGCTGGACGACACCGACAAGTCGACACTGAACAAGGAGATCGAGGCGGCGCTGGAGTGGC
TGAGCAGCAACCAGGAGGCAACGAAGGAGGAGTATGAGCACAAGCAGAAGGAGCTGGAGAACGTATGCAA
CCCGATCATGACCAAGATGTACCAGAGCATGGGCGGTGGTGCGGGCGGCATGGCCGGTATGCCGGACATG
AGCGGCATGAGCGGTGCGCGGCCCGCCGGCGGCGCCTCCTCCGGCCCCAAGGTCGAGGAGGTCGAC
>L16952 679, 930 RPL37
ATGACGAAGGGTACCACCTCGATGGGTCAGCGCCACGGGCGCACGCACATCCTGTGCCGCCGCTGTGGCC
GCAACTCCTACCACGTTCAGTGGGAGCGGTGCGCCGCCTGCGCCTACCCGCGTGCCAGCCGCCGTCGCTA
CAACTGGTCCGTGAAGGCCATCAAGCGCCGCCGCACCGGCACCGGCCGCTGCCGCTACCTGAAGGTGGTC
AACCGCCGCATTGCCAACCACTTCAAGACCCCCAAGGCGTAA
>L16961 71, 1201 actin
ATGGCTGACAACGAGCAGAGCTCCATCGTCTGCGATAACGGTTCCGGCATGGTGAAGGCCGGCTTCTCTG
GCGATGACGCTCCTCGCCACGTTTTCCCTTCGATCGTGGGCCGCCCGAAGAACATGCAGGCCATGATGGG
GAGCGCGAACAAGACGGTTTATGTTGGCGACGAGGCGCAGTCGAAGCGCGGCGTGCTGTCGCTGAAGTAC
CCGATCGAGCACGGAATTGTGACAAACTGGGATGACATGGAGAAGATCTGGCACCATACCTTCTACAACG
ACGTGCGGGTGAACCCCGAGCAGCACAACGTGCTGTTGACGGAAGCGCCGATGAACCCGAAGCAGAACCG
TGAGAAGATGACGCAAATCATGTTCGAGACGTTCAATGTGCCGTCGCTGTACATTGGAATTCAGGCTGTG
CTGTCCCTGTATTCCTCTGGCCGCACCACCGGTATTGTGCTTGATGCGGGCGATGGCGTGACGCACACCG
TTCCAATCTACGAGGGCTACTCCCTGCCGCACGCGGTGCGGCGTGTGGACATGGCTGGCCGCGACCTGAC
GGAGTACCTGATGAAGATCATGATGGAGACGGGTACGACGTTCACGACGACCGCGGAGAAGGAGATTGTG
CGCAACGTGAAGGAGCAGCTGTGCTACGTAGCGCTGGACTTTGAGGAGGAGATGACGAACAGCGCCAAGT
CGGCGAACGAGGAGGCGTTTGAGCTGCCGGACGGCAACGTCATGATGGTGGGCAATCAGCGCTTCCGCTG
CCCAGAGGTGCTGTTCAAGCCGTCGCTCATTGGTCTCGACGAGGCTCCCGGCTTCCCGGAGATGGTGTAC
CAGTCCATCAATAAGTGTGACATTGATGTGCGGCGCGAGCTGTACGGGAACATCGTGCTGTCCGGTGGCT
CGACCATGTTCCTCAACCTTCCGGAGCGCCTGGCGAAGGAGATCTCGAACCTGGCGCCGTCGTCGATAAA
GCCGAAGGTGGTTGCGCCGCCGGAGCGCAAATACAGCGTGTGGATCGGCGGCTCGATTTTGTCATCGCTG
ACGACCTTCCAGACAATGTGGGTGAAGAAGAGCGAGTACGACGAGTCTGGCCCAAGCATCGTGCACAACA
AGTGCTTCTGA
>L25120 166, 1419 cPGK
ATGTCTCTCGTGCTGAAGAAGAGCATCGATGACGCCACCGTCAGGGACAAGAAGGTGCTCATCCGCGTGG
ACTTCAACGTCCCCGTGAAGAACGGTAAGATCACGAATGACTTCCGCATTCGCTCCGCCCTTCCGACGAT
CCAAAAGGTGCTGAAGGAGGGCGGCTCCTGCATCCTGATGAGCCACCTTGGTCGGCCGAAGGGTGCGAGA
ATGAGTGACCCAAGTCCGGAGAAGGGTGTGCGCGGGTACGAGGAGGCCGCCACGCTGCGCCCGGTTGCTG
CACGCATCAGCGAGCTGCTGGGGCAGAAGGTGGAGTTTGCACCGGACTGCCTGGACGCTGCGTCGTACGC
GTCGAAGCTGAAGAACGCGGACGTGCTGTTGCTGGAGAACGTGCGTTTCTACGCGGAGGAGGGCAGCAAG
AAGGAGGAGGAGCGCGACGCGATGGCGAAGGTACTAGCGTCGTACGGCGACGTGTATGTCAGTGACGCGT
TTGGCACTGCGCACCGCGACAGCGCGACGATGACGGGCATCCCCAAGGTGCTGGGTGCGGGCTACGCCGG
GTACCTGATGGAGAAGGAGATCAACTACTTCTCGCGAGTGCTGAACAACCCGCCGCGGCCGCTGGTTGCG
ATTGTCGGCGGTGCGAAGGTCAGCGACAAGATCCAGCTGCTTGACAACATGCTGGGCCGCATCAACTACC
TTGTGATTGGTGGCGCGATGGCGTACACGTTCCAGAAGGCGCAGGGCCGCAAGATCGGCATCTCGATGTG
CGAGGAGGATAAGCTGGACCTTGCCAAGTCGCTGCTGAAGAAGGCGCAAGAGCGGAATGTGCAGGTGTTT
CTGCCAGTTGACCACGTGTGCAACAAGGAATTTAAGGCCGCGGACTCGCCGCTGGTGACGGAGAGCGTGG

ACGTCCCGGACGGCTACATGGCGCTGGACATTGGCCCAAGGACGATCCACATGTACGAGGAAGTGATTGG
CCGCTGCAAGAGCGCGATCTGGAACGGCCCGATGGGCGTGTTCGAGATGCCGTGCTACTCGAAGGGCACA
TTTGCCGTTGCGAAGGCGATGGGGACTGGAACGCAGAAGAACGGGCTGATGAGCATCATCGGCGGTGGTG
ACAGCGCGAGCGCTGCGGAGCTGAGCGGCGAGGCGAAGAACATGTCGCATGTGTCCACCGGCGGCGGTGC
GTCCCTGGAGCTGCTGGAGGGCAAGACGCTGCCTGGCGTCGCCATTCTGACGGACAGAGAATGA
>L25121 166, 1605 gPGK
ATGTCTCTCGTGCTGAAGAAGAGCATCGATGACGCCACCGTCAGGGACAAGAAGGTGCTCATCCGCGTGG
ACTTCAACGTCCCCGTGAAGAACGGTAAGATCACGAATGACTTCCGCATTCGCTCCGCCCTTCCGACGAT
CCAAAAGGTGCTGAAGGAGGGCGGCTCCTGCATCCTGATGAGCCACCTTGGTCGGCCGAAGGGTGCGAGA
ATGAGTGACCCAAGTCCGGAGAAGGGTGTGCGCGGGTACGAGGAGGCCGCCACGCTGCGCCCGGTTGCTG
CACGCATCAGCGAGCTGCTGGGGCAGAAGGTGGAGTTTGCACCGGACTGCCTGGACGCTGCGTCGTACGC
GTCGAAGCTGAAGAACGCGGACGTGCTGTTGCTGGAGAACGTGCGTTTCTACGCGGAGGAGGGCAGCAAG
AAGGAGGAGGAGCGCGACGCGATGGCGAAGGTACTAGCGTCGTACGGCGACGTGTATGTCAGTGACGCGT
TTGGCACTGCGCACCGCGACAGCGCGACGATGACGGGCATCCCCAAGGTGCTGGGTGCGGGCTACGCCGG
GTACCTGATGGAGAAGGAGATCAACTACTTCTCGCGAGTGCTGAACAACCCGCCGCGGCCGCTGGTTGCG
ATTGTCGGCGGTGCGAAGGTCAGCGACAAGATCCAGCTGCTTGACAACATGCTGGGCCGCATCAACTACC
TTGTGATTGGTGGCGCGATGGCGTACACGTTCCAGAAGGCGCAGGGCCGCAAGATCGGCATCTCGATGTG
CGAGGAGGATAAGCTGGACCTTGCCAAGTCGCTGCTGAAGAAGGCGCAAGAGCGGAATGTGCAGGTGTTT
CTGCCAGTTGACCACGTGTGCAACAAGGAATTTAAGGCCGCGGACTCGCCGCTGGTGACGGAGAGCGTGG
ACGTCCCGGACGGCTACATGGCGCTGGACATTGGCCCAAGGACGATCCACATGTACGAGGAAGTGATTGG
CCGCTGCAAGAGCGCGATCTGGAACGGCCCGATGGGCGTGTTCGAGATGCCGTGCTACTCGAAGGGCACA
TTTGCCGTTGCGAAGGCGATGGGGACTGGAACGCAGAAGAACGGGCTGATGAGCATCATCGGCGGTGGTG
ACAGCGCGAGCGCTGCGGAGCTGAGCGGCGAGGCGAAGAACATGTCGCATGTGTCCACCGGCGGCGGTGC
GTCCCTGGAGCTGCTGGAGGGCAAGACGCTGCCTGGCGTCGCCATTCTGACGGACAAGGACGTGAAGGAG
CGGGGAGCTTCGTGCAAGTTCGCTTTCGGGGTCGGGAGCCCGAGCAGGGAGGCTTGTCCGCTCCGTTGTG
GGCATATCTTTGGCGGTGCCTCCATCGTAAGAGAGATAGTTAAGATCGTCGTCGCGCTGCTGATCGGCAT
TTTTATTGGTCGCCGCATGAGCACCAAGCTGATCCGGTAA
>L26252 206, 1099 fibrillarin
ATGCGCGGTGGCTTTGGACGTGGCGGTGGTGGTCGCGGCGGCAGCCGTGGTGGTCGCGGAGGCTTTGGCC
GTGGCGGCGGTCGCGGTGGAGGCCGTGGCGGCGGTCGCGGTGGAGGCCGTGGCGGCGGTCGCGGTGGTGG
CCGTGGCGGCGGTCGCGGTGGTGCGGGTGCCAAGGTTATCGTGGAGCCACACATGCTGCATCCTGGCGTG
TTCATTTCCAAGGCCAAGACCGACTCTCTTTGCACGCTGAACATGGTGCCCGGTATTTCCGTTTATGGCG
AGAAGCGTATCGAGCTGGGTGCAACGCAGGGAGGTGATGATAAGAAGGAGTACCGTCTGTGGAACCCCTA
CCGCTCGAAGCTGGCCTCCGCCATCTACGCTGGTGTGAGTAGCATTCACATGGGTCCCGGCTCGAAGGTA
CTGTATCTCGGCGGTGCAACGGGTACCACCGTCAGCCACGTGAGCGACCTTGTGGGTCCGGAGGGCATGG
TGTATGCTGTGGAGTTCTCGCACCGTGTAGGCCGCGATTTAGTGGAGATGTCGAAGCGCCGCCCGATTGT
GCCCATTGTTGAGGATGCCCGCTACCCCATGAAGTACCGCATGCTTGTGCCGATGGTGGATTGCATTTTC
ATGGATGTCGCCCAGCCAGACCAGGCGCGAATTCTTGCACTCAACGCACAGGCTTTTCTGCAGAACGGTG
GTCACTATGTGATCTCTATCAAGGCTAACTGCATCGACTCTACCCTGGAGGCCCCTGTAGTGATCGCGTC
TGAGCTGAACAAGCTGAAGAAGGACAAGCTGAAGCCACTGGAGCAAGTGTCTCTTGAGCCGTTCGAGCGC
GATCACGCTGTAGTTGTTGGCGTGTACCGCCCTGTCAAGAAGTCGAAGCAATGA
>L26562 206, 1099 Leishmania major fibrillarin homologue, complete cds
ATGCGCGGTGGCTTTGGACGTGGCGGTGGTGGTCGCGGCGGCAGCCGTGGTGGTCGCGGAGGCTTTGGCC
GTGGCGGCGGTCGCGGTGGAGGCCGTGGCGGCGGTCGCGGTGGAGGCCGTGGCGGCGGTCGCGGTGGTGG
CCGTGGCGGCGGTCGCGGTGGTGCGGGTGCCAAGGTTATCGTGGAGCCACACATGCTGCATCCTGGCGTG

TTCATTTCCAAGGCCAAGACCGACTCTCTTTGCACGCTGAACATGGTGCCCGGTATTTCCGTTTATGGCG
AGAAGCGTATCGAGCTGGGTGCAACGCAGGGAGGTGATGATAAGAAGGAGTACCGTCTGTGGAACCCCTA
CCGCTCGAAGCTGGCCTCCGCCATCTACGCTGGTGTGAGTAGCATTCACATGGGTCCCGGCTCGAAGGTA
CTGTATCTCGGCGGTGCAACGGGTACCACCGTCAGCCACGTGAGCGACCTTGTGGGTCCGGAGGGCATGG
TGTATGCTGTGGAGTTCTCGCACCGTGTAGGCCGCGATTTAGTGGAGATGTCGAAGCGCCGCCCGATTGT
GCCCATTGTTGAGGATGCCCGCTACCCCATGAAGTACCGCATGCTTGTGCCGATGGTGGATTGCATTTTC
ATGGATGTCGCCCAGCCAGACCAGGCGCGAATTCTTGCACTCAACGCACAGGCTTTTCTGCAGAACGGTG
GTCACTATGTGATCTCTATCAAGGCTAACTGCATCGACTCTACCCTGGAGGCCCCTGTAGTGATCGCGTC
TGAGCTGAACAAGCTGAAGAAGGACAAGCTGAAGCCACTGGAGCAAGTGTCTCTTGAGCCGTTCGAGCGC
GATCACGCTGTAGTTGTTGGCGTGTACCGCCCTGTCAAGAAGTCGAAGCAATGA
>L28800 201, 734 hydrophilic surface protein
ATGGGAAGCTCTTGCACGAAGGACTCCGCAAAGGAGCCCCAGAAAAGTGCGGATAAGATCAAGAGTACCA
ATGAGACCAACCAGGGAGGGAATGCGAGTGGCTCCCGGAAGAGCGCCGGCGGTCGGGCCAACGAATATGA
CCCGAAGGATGATGGCTTCACGCCGAACAACGAAGACCGTTGCCCGAAGGAGGATGGCCATGCGCCGAAG
AACGATGACCATGCCCCGAAGGAGGATGGCCATGCGCCGAAGAACGATGACCATGCCCCGAAGGAGGATG
GCCATGCGCCGAAGAACGATGACCATGCCCCGAAGGAGGATGGCCATGCGCCGAAGAACGATGACCATGC
CCCGAAGGAGGATGGCCATGCGCCGAAGAACGATGACCATGCCCCGAAGGAGGATGGCCATGCGCCGAAG
AACGACGGGGATGTGCAGAAGAAGAGCGAAGATGGAGACAACGTGGGAGAGGGAGGCAAGGGCAATGAGG
ATGGTAACGATGATCAGCCGAAGGAGCACGCTGCCGGCAACTAG
>L31814 1, 457 beta-tubulin
CGAGGGCGCGGAGCTGATCGACTCCGTGCTTGATGTGTGCCGCAAGGAGGCGGAGTGCTGCGACTGCCTG
CAGGGCTTCCAGCTGTCTCACTCCCTCGGCGGCGGCACGGGCTCCGGCATGGGCACGCTGCTCATTTCCA
AGCTGCGCGAGGAGTACCCGGACCGGATCATGATGACCTTCTCCGTCATCCCGTCCCCCCGCGTGTCGGA
TACCGTTGTGGAGCCGTACAACACGACCCTCTCTGTGCACCAGCTCGTGGAGAACTCCGACGAGTCCATG
TGCATCGACAACGAGGCGCTGTACGATATTTGCTTCCGCACGCTGAAGCTGACGACGCCGACGTTCGGTG
ACCTGAACCACCTCGTCGCCGCTGTGATGTCTGGCGTGACCTGCTGCCTGCGCTTCCCTGGCCAGCTGAA
CTCTGACCTGCGCAAGCTTGCCGTGAACCTCGTGCCG
>L40331 106, 1251 Leishmania major silent information
regulator 2 homolog, complete
ATGACAGGGTCTCCGAGAGCGCCGCATCAGGAACATGCCCTCGGAGAGCCGACTGTGGAAGGGCTTGCGC
GCTACATCAGGGAGAAGGATGTGCGGCGCATTCTCGTGCTCGTCGGAGCAGGCGCCAGCGTAGCTGCCGG
CATCCCAGACTTTCGCTCATCTGACACCGGGATCTACGCCAAGCTCGGCAAGTACAACCTCGACGACCCG
ACCGATGCCTTTTCGCTGACTCTTCTGCGCGAGAAGCCAGAGATATTCTACTCTATCGCACGGGAGCTGA
ACTTGTGGCCTGGGCACTTTCAGCCCACCGCGGTGCATCACTTCATCCGACTGTTGCAAGACGAGGGCCG
TCTTCTGCGCTGCTGCACGCAGAACATTGATGGTCTGGAGAAGGCAGCGGGCGTGTCGCCGGAGCTCCTG
GTCGAGGCGCATGGGTCTTTCGCTGCCGCCGCCTGCATCGAATGCCACACGCCATTCAGCATTGAGCAGA
ACTACCTGGAGGCGATGAGCGGCACGGTGTCCCGCTGCTCTACATGCGGCGGCATTGTGAAGCCAAACGT
CGTTTTCTTTGGTGAAAATTTGCCGGACGCGTTCTTCGACGCGCTGCACCACGACGCCCCGATCGCGGAG
CTGGTCATCATCATCGGGACATCGATGCAGGTGCACCCGTTCGCGTTACTGCCGTGCGTCGTGCCCAAGT
CCATCCCGCGCGTTCTCATGAACCGCGAGCGAGTTGGCGGCCTCCTCTTCCGCTTTCCTGATGACCCGCT
CGACACCATCCACGACGATGCGGTTGCCAAGGAGGGACGCTCGTCCTCTTCGCAGAGCCGTTCCCCGTCC
GCGTCGGCGCGGCGCGAGGAGGGGGGGACGGAGGACGGCTCCTCGTCGCCGAACGAGGAGGTCGAAGACG
CGTCGACGTCCAGTTCGAGTGACGGCTACGGTCAGTACGGTGACTACTACGCCCACCCCGATGTCTGCCG
GGATGTTTTCTTCCGCGGCGACTGCCAGGAGAACGTGCTGAAGCTGGCCGAGTGCCTGGGCCTCAGGGAG
GCGCTGGCCAAGCGATGCGCTTCTCCGGTGCGGCACCAGCTACGGCACGAAAGACGTCGAATGAGACGTG

AGTCTGAATTGCTGCCAACATGGTGACTGAAGAGAGTTCATGAAGGCCGTAGGCAGAGCAGCTGAGCGCA
TCGAAGGCGGTCACACGCACCGCGCGTAGCCTCGCCTCGAATCATTTCAGCGCTGTCGTTTCCTTCTACG
CATG
>L43630 29, 1186 surface antigen 2
ATGGCGCAGTGCGTGCGTCGGTTGGTGCTGGCGGCGACGCTCGCCGCTGCGGTGGCGCTGCTGCTGTGCA
CGAGCAGTGCGCCGGTGGCGCGTGCTGCTGGGACGGGCGACTTCACTGCGGCGCAGCGGACGAACACGCT
GGCGGTGCTGCAGGCGTTTGGGCGTGCGATCCCTAAGCTTGGGGAGAAGTGGGCGGGCAACGACTTCTGC
TCGTGGGAGGCCGTCTTGTGCAATGCGCCGGACGTGTACGTGTCGGGAATCAGTCCGACGTATGCCGGCA
CGCTGCCGGAGATGCCTGTGAACGTCGACTACAGGCACGTCGTGATCAAGCAGCTCGACTTTTCCGAAAT
GGGGCCGGGGCTGAGCGGGACGCTGCCCGCCTCATGGCACTCGATGACATCTTTGGAGTCGTTGTCGATT
GAAAAGTGTGAAAGCATCTCCGGCAGTGTGCCCCCCGAGTGGGGCTCGATGACATCGCTGAGTGTTCTCA
ATCTGCGGGGCACAGGCATCTCCGGCACGCTGCCGCCCCAGTGGAGTGGGATGTCGAAGGCCCGGTCCCT
GCAGCTGCAGGACTGCGACCTGTCCGGAAGTCTGCCCTCTTCGTGGTCTGCGATACCGATGCTGGCTTCC
GTCTCTCTTAAGGGAAACAAGTTCTGCGGGTGTGTGCCGGACTCGTGGGATCAGAAGGCTGGTCTTGTTG
TGGACATCGAGGACAAGCACAAGGGCAGCGACTGCTTGGCTGCTAAGGACTGCACAACGACCACCACTAA
GCCCTCCGCCACGACAGCGACCACCCCGAACCTCACTAACTTTCCCCCTACGCCGAGGACCACGACTGAG
CCGCTTACCACAACCAGCACTGAGGCACCGGCTGAACCCACAACCACCACTGAGGCACCGGCTGAACCCA
CGACCACTGCTACCCCAACAAACACGCCGACTCCTGCACCAGAGACGGAGTGCGAGGTGGATGGGTGTGA
GGTGTGCGAGGGGGACTCCGCTGCGAGGTGCGCGAGGTGCCGTGAGGACTACTTCCTGACGGACGAGAAG
ACGTGCCTGAAGCACAACGATGGCGGTGTTGCTGCTGTGTCGAGCGGAGTGGCAGCAGCAGCTGTTGTGT
GCGTGGCTGTGCTGTTCAGCGTGGGGCTGGCGGCGTGA
>L76742 99, 905 dut
ATGAAGCGCGCTCGCAGCGCCAATATTCCAGGTGCGATTCTGCACTCCTTGGCAGAGCTGCAGGATGGCC
TGAACGCGATGATCGACCCTTCCTGGCGCGCTGTTCGCTCGCTGGACAATTGGGCTCTAGCCATCACGAT
GGAGTCGACGGAGCTGCTGGACTCCTATCCGTGGAAGTGGTGGAAGAACCTCAACGCTACGCCTGATTTG
GCCAACGTTAGGATTGAACTGGTCGACATCTTTCACTTTTCCCTTTCGGGCGCTATGCAAATGCGGTCTA
CCCCGGACGATGAGATTCCCGCCGCTTCGCTCAAGCCTCTGAAGGAGGTCATGACAACTTTTCTGCCGGC
TAAGGAGTGCACGAGCGACCCTTACGGCTTTGTGTTCTTTCCCCTCACCGATACACAGAACGCCATTGCG
AGCTTTCGTAACATCATTCAGCTCGCAAACGCCTATCGCTTCGATGTCATCATAGAGTGCATCATATACG
CCGCTGAGGACCTGGGGTTTAACTTGGTGGCGTACTATATTGCAAAACACACTCTGAACTGCATTCGCCA
GCTGAGTGGCTACAAAGACGGCTCTTACGTGAAGGTGAACAATGGTGTTGAGGACAATTCACTTCTGCAC
AACTGCATCAAAGACGTATCCCTCGATGAAGTTCTCGACGCTGACAAGTATGTCCAGGCGTGGAACAGCA
TCATGGCTAACGTTTACGAAGCTTTCCAGATTAAGGAGTCTGACCGAAAGGATGCGGAGCGCTGGTTTGC
CCTCGCAAAGGAAAATCGGCTGGCGATCAAGGCATAA
>M12734 121, 1683 Leishmania major bifunctional
dihydrofolate reductase-thymidylate
ATGTCCAGGGCAGCTGCGAGGTTTAAGATTCCGATGCCGGAGACGAAGGCAGACTTTGCTTTCCCCTCCC
TGCGCGCCTTCTCCATCGTCGTGGCCCTCGATATGCAGCACGGCATCGGCGACGGCGAGTCGATCCCGTG
GCGGGTGCCGGAGGACATGACGTTTTTCAAGAACCAGACGACGCTGCTGCGCAACAAGAAGCCGCCGACG
GAGAAGAAGCGCAACGCCGTCGTGATGGGCCGCAAGACTTGGGAGAGCGTCCCGGTAAAGTTCCGACCAC
TCAAGGGACGGCTGAACATCGTGTTATCCTCGAAGGCCACCGTCGAGGAGCTTCTGGCGCCGCTGCCGGA
GGGACAGCGCGCGGCGGCGGCGCAGGATGTGGTGGTGGTGAACGGCGGTCTGGCCGAGGCGCTCCGCCTC
CTCGCACGCCCGCTGTACTGCAGCTCCATCGAGACAGCGTATTGCGTCGGTGGTGCGCAGGTTTACGCGG
ACGCCATGCTGTCGCCGTGCATCGAGAAACTGCAGGAAGTGTACCTGACCCGCATCTACGCGACGGCGCC
TGCGTGTACGCGCTTCTTTCCGTTTCCGCCCGAGAACGCGGCCACGGCGTGGGACCTGGCGTCGTCTCAG

GGACGCCGCAAGAGCGAGGCGGAGGGCCTCGAGTTCGAGATCTGCAAGTACGTGCCGCGCAACCACGAGG
AGCGGCAGTACCTTGAGCTGATTGACCGCATCATGAAGACGGGGATCGTGAAGGAGGACCGCACCGGCGT
GGGCACCATCAGCCTCTTCGGCGCCCAGATGCGCTTCTCCCTACGCGACAACCGCCTGCCGCTGCTGACG
ACGAAGCGTGTCTTCTGGCGCGGCGTGTGCGAGGAGCTGCTGTGGTTCCTGCGCGGGGAGACGAGTGCGC
AGCTGCTGGCAGACAAGGACATTCACATCTGGGACGGCAACGGTTCGCGCGAGTTTCTCGACAGCCGCGG
CTTGACAGAGAATAAGGAGATGGACCTCGGCCCTGTCTACGGCTTCCAGTGGCGCCACTTCGGGGCAGAT
TACAAGGGGTTTGAAGCGAACTACGACGGCGAAGGGGTGGACCAGATCAAGCTCATCGTGGAGACCATCA
AGACGAACCCGAACGACCGCCGCCTCCTAGTCACTGCCTGGAACCCGTGCGCGCTGCAAAAGATGGCGCT
GCCGCCGTGCCACTTGCTTGCTCAGTTCTACGTGAACACAGACACGAGCGAGCTATCCTGCATGTTGTAC
CAGCGCTCGTGTGACATGGGTCTTGGCGTCCCCTTCAACATTGCCTCCTACGCGCTGCTCACCATCCTCA
TTGCCAAGGCGACGGGTCTGCGGCCTGGTGAGCTTGTGCACACCCTCGGCGACGCCCACGTCTACCGCAA
CCACGTTGATGCCCTCAAGGCGCAGCTCGAGCGAGTCCCGCACGCGTTCCCGACCCTCATCTTCAAGGAG
GAGCGGCAGTACCTCGAGGACTACGAGTTGACGGACATGGAGGTGATCGACTACGTTCCACACCCGGCGA
TCAAGATGGAGATGGCCGTATAGAGAGAGGGAGGGTGTCATGTGCGTTGTATGCATGCAGCCACCGCCGC
GACGCTGCCACTCACCTGGCTCCCACCTCCTCGTCGCACGACGACCGCCCCTTGCGCAGACTCGTTGGGT
AACCATGAGCGGCGCGGAGGGGTACGCGCCTGCGTTTTCTCGCACATGGCTGCGGCTGATATCCCGCCCG
CACCCGCGCGCGCTCAGGCCGCGTGTCGTCGTGCTCTTTCCATTTTTTTTTTGTGTGTGGTGGTGATTTG
AGCTGCTCTCCGTTGTGTGCTGGGGACCCTCCTTCCCTCGATCTCCTCGTGCGGGGTCTCCGACGCGCAG
ACGCGGTGCGTGCGAGCGTGCACGTGCTGTCGCCGTGGCTGTAGTTGCGAGCGGAGAGAGAGAGAGAGGG
AGGGAGGGAGGGCAGAGGGCAGAGGACATGCGGGTGGGAACGTGCACCGGCCTCGTCTCACGCAGCTGGA
GCCCACGAATGCCACCACCACACCCTTCTTCCCCCCCCCTCCCTCTTCTTCCCACGGCGGCGACGACGAC
GGGGCTCAGCTCACGCTTCTGTAGGGTATTATATTAAAGCACATGTGCGTGCTGTGCTTCGGCTTCTTGT
TGGTGGTCGTTCGCTTTCAAGTCCACGACTCCTGCCGTTGCTCACCGCCGGGCTGTCTTCTTTCGCCCCT
CCCTCGGTGCCTCTTCCGCCGCCGCACGCGTTGCGCTTGATCACGCGTCTTGCGTGATGTGCCTGTGTGT
ATACACACCGTGCACGGAGAGAGCGAGCGAGCGAGAGAGAGAGAGGGCGAGAGGAAGAATGGCCGGTGCC
TCGCGTGGGCAAGCGTGCGCGAGTTTGTCGTGTCGCTGCGCCGCGTGGATGTCGCGCAGCGCAAGACCCC
GTGCGCCTGAAACGTGAAGGGAGGTCGAGAAGCGTGCCGCATGAGGCCTTAAGGCAGGAAAGTGAAAAGA

>M28690 1, 906 ND1
GACATTTTAGTTGTTCTTGTCTTGACCGGCTTTGTAAGTCTTTGTGAAAGAAGAATTTTAGCTTTAGTTC
AAATACGAATAGGTCCGGCCCTTTGTTTTTTTGGAATTTTAACACCAATAACTGACGGAATAAAACTTTT
TATTAAATTTATCATTTTTGTAATTAGTTTTGAAATCATTTACTTAATAGGTGCAATCTTAATTACTACA
TGCTGCATTTTTATTGGTTGGTTTTATTTTCCTATAGGTTTTATACTGTTATTAGACACAGGATTCACTA
TTACAATAATGATGTGCGTACATGTTTTCAGTAATATGTTTAGCACATTTTTTGTAGGTTGCTTTTTATT
CTCTAGTTGCTTTGTATATCTTTCTGCAATGCGCACTATGTTTTTTAGTATTATTTCTGAAAGTGGCTTA
TTTTTATTATACACAACTGTATATTCGTTAGACTACTTTAGCTTTTTTTGCATTAAAGACATTTGTGTTG
GGCAGATTTATATTACAAACTTTTATATTGCCGGCATCTTATTTGTATGTATATTTTGAATAAGTATGCT
TCTAGACGGATTAAAACTACCATTCGACTATATGGAATGTGAAAGTGAATTAGTCGCTGGGTTGATTACA
GAACTATCAGGATTCTTTTTTATATTATATTCCGTTCTTGAAATAAATCATGTGTTACTGACAACATTAT
TATTAGCCAGTCTCTGTTTTGGGGGCTTATTTATATGTTTTAAAGCTATAATTATCTTAATTATTGGGTT
TTTTTATCCAAGGGTAATTGGTTATAGACTAAAAATTACAACAGCGCAAGCATTTATACTTATTTTTTTA
TTCTACATGTGCGTTCTTACTTTTATTTGACTGTTCACTACAAAAATAATTGCTATATTATTTTAA
>M36675 710, 2257 heat shock protein 70
ATGACATTCGACGGCGCCATCGGCATCGACCTGGGCACGACGTACTCGTGCGTAGGCGTGTGGCAGAACG
AACGCCTGGATATCATCGCGAACGACCAGGGTAACCGCACGACACCGTCGTACGTTGCGTTCACGGACTC

GGAGCGGCTGATCGGCGATGCTGCAAAGAACCAGGTGGCAATGAACCCGCACAACACGGTGTTCGACGCG
AAGCGCCTGATTGGCCGCAAGTTCAACGACTCGGTTGTGCAGTCGGACATGAAGCACTGGCCGTTCAAGG
TGACGACGAAGGGTGACGACAAGCCCGTGATTTCGGTGCAGTACCGCGGCGAGGAGAAGACCTTCACGCC
GGAGGAGATCAGCTCGATGGTGCTGCTGAAGATGAAGGAGACGGCGGAGGCGTACCTGGGCAAGCAGGTG
AAGAAGGCCGTGGTGACGGTGCCGGCGTACTTCAACGACTCGCAGCGCCAGGCAACGAAGGACGCCGGCA
CGATTGCTGGCCTGGAGGTCGTGCGCATCATCAACGAGCCGACGGCAGCGGCCATCGCGTACGGTCTGGA
CAAGGGTGACGACGGCAAGGAGCGCAACGTGCTGATCTTCGACCTTGGCGGCGGCACGTTTGATGTGACG
CTGCTGACGATCGACCGCGGCATCTTCGAGGTGAAGGCGACGAACGGCGATACACACCTTGGCGGCGAGG
ACTTCGACAACCGCCTCGTCACGTTCTTCACCGAGGAGTTCAAGCGCAAGAACAAGGGTAAGAACCTGGC
GTCGAGCCACCGCGCGCTGCGCCGTCTGCGCACGGCGTGCGAGCGCGCGAAGCGCACGCTGTCGTCCGCG
ACGCAGGCGACGATCGAGATCGACGCGCTGTTCGAGAACATTGACTTCCAGGCCACCATCACCCGCGCGC
GCTTCGAGGAGCTGTGCGGCGACCTGTTCCGCAGCACGATCCAGCCGGTGGAGCGCGTGCTGCAGGACGC
GAAGATGGACAAGCGCTCCGTGCACGACGTGGTGCTGGTGGGCGGGTCAACGCGCATCCCGAAGGTGCAG
TCCCTCGTGTCGGACTTCTTCGGCGGCAAGGAGCTGAACAAGAGCATCAACCCCGACGAGGCTGTCGCGT
ACGGCGCTGCGGTGCAGGCCTTCATCCTGACGGGCGGCAAGAGCAAGCAGACGGAGGGCCTGCTGCTGCT
GGACGTGACGCCGCTGACGCTGGGCATCGAGACGGCCGGCGGCGTGATGACGGCGCTGATCAAGCGCAAC
ACGACGATCCCGACCAAGAAGAGCCAGATCTTCTCGACGTACGCGGACAACCAGCCCGGCGTGCACATCC
AGGTCTTCGAGGGCGAGCGCGCGATGACGAAGGACTGCCACCTGCTGGGCACGTTCGACCTGTCCGGCAT
CCCGCCGGCGCCGCGCGGCGTACCGCAGATCGAGGTGACGTTCGACCTGGACGCAAACGGCATCCTGAAC
GTGTCCGCGGAGGAGAAGGGCACCGGCAAGCGCAACCAGATCACCATCACCAACGACAAGGGCCGGCTGA
GCAAGGAC
>M63109 1, 704 GP 96-92
AGTGCCAAGCTTGGCTGCAGGTCGACGGATCCTGGAGCCCGTCAGTATCGGCGGGCGAGGAGGAGGAGAA
GCAGCGCATCGAGATGGTGCGCAAGCAGCGTGAGGAGGCTCAAAAGAAGCGCGAGGAGATCCAGAAGCAG
CGCGAGGAGGAGATCAAGCGCCGCAAGGCTGAGATCGAGGCAGAGAGGCAGAAGCTGAAGGAGCTGCAGG
AGGAGCACGAGAGGGAGCAGGAGGAGGCCCGCAGCCGTCGCGTCGCGGAGGAGAAGGAGGCACAGAAGAA
GGCCGAGAAGAAGGCCGAGGAGGCCGAAGACGAGCTTGCTGCGACGCGCCGGCAGAGGAAGGGGGAGTTG
GAGGAGCTGCAGCGCCACGCGGAGAAGGAGGAGAAGCAGCGCATCGAGATGGTGCGCAAGCAGCGTGAGG
AGGCTCAAAAGAAGCGCGAGGAGATCCAGAAGCAGCGCGAGGAGGAGATCAAGCGCCGCAAGGCTGAGAT
CGAGGCAGAGAGGCAGAAGCTGAAGGAGCTGCAGGAGGAGCACGAGAGGGAGCAGGAGGAGGCCCGCCAG
CGTCGCGTCGCGGAGGAGAAGGAGGCACAGAAGAAGGCCGAGAAGAAGGCCGAGGAGGCCGAAGACGAGC
TTGCTGCGACGCGCCGGCAGAGGAAGGGGGAGTTGGAGGAGCTGCAGGCCCAGCGCGAGGAGGAGGCGGA
ATTC
>M64672 201, 620 fl1.1
ATGATCATCCGCTATATGTCGAGGGTTACTTCCCTCCTTTCCCAAGGCCTGCACCATGGGTACGGTGGCG
TCTCGTCTATCCGTAGCGCAGTATTGGAGGAACTCCATCTGCTCTGTATGGAGGGGGCACGCGTGGTGCA
CATGTCTCCATCTTTTGTGCTTGCCTCTGCGCATTTTGTGCGGTGGCGTGTATGTGGGCATACAAACCAC
ACACGCCGTGCCGCCGACCTGCCTGGTGAGAATTTGCCATGCACTGGTCGCCGTTGTACTTTTTTGTTGT
ATTTTGCTCTTCTCTCGTTTTTCGCGCTCTCTCTCGTGCTAGAGATCGCTGTATTGCCTTCTGTGTTTGC
TTTTCTTGATGCCCGTTGTTTGTGCGTCAGTCCCTCCTCTGCCCCACAGTCCCTTCGCCGTTCTCTGTGA

>M94390 592, 1407 HEXBP
ATGTCCGAAACCGAAGACGTCAAGCGTCCGCGCACCGAGAGCAGCACCAGCTGCCGCAATTGCGGTAAGG
AGGGCCACTACGCCCGCGAGTGCCCCGAGGCCGACTCCAAGGGTGACGAGCGCAGCACGACGTGCTTCCG
CTGCGGCGAGGAGGGCCACATGAGCCGCGAGTGCCCGAACGAGGCCAGGTCCGGCGCAGCTGGTGCTATG

ACGTGCTTCCGCTGCGGCGAGGCCGGCCACATGAGCCGTGACTGCCCCAACTCCGCGAAGCCGGGTGCGG
CCAAGGGCTTCGAGTGCTACAAGTGCGGTCAGGAGGGTCATCTCAGCCGTGACTGCCCCAGCAGCCAGGG
TGGAAGCCGCGGTGGATACGGCCAGAAGCGCGGCCGCAGCGGCGCACAGGGCGGCTACAGTGGCGATCGC
ACGTGCTACAAGTGCGGTGACGCCGGCCACATCAGCCGTGACTGCCCCAACGGTCAGGGCGGGTACAGCG
GCGCGGGTGACCGCACATGCTACAAGTGCGGTGACGCCGGCCACATCAGCCGTGACTGCCCCAACGGTCA
GGGCGGGTACAGCGGCGCGGGTGACCGCAAGTGCTACAAGTGCGGCGAGTCGGGCCACATGAGCCGCGAA
TGCCCCAGCGCCGGGAGCACTGGCAGCGGTGACCGCGCATGCTACAAGTGCGGCAAGCCTGGCCACATCA
GCCGCGAGTGCCCGGAGGCCGGTGGCAGCTATGGCGGTTCCCGCGGCGGCGGTGACCGCACGTGCTACAA
GTGCGGCGAGGCCGGCCACATCAGCCGTGACTGCCCCAGCAGCTAA
>U01031 1, 318 histone H-1 like protein
ATGTCCTCTAATTCCGCCGCTGCTGCCGTTTCCGCCGCCACGACCTCGCCGCAGAAGTCTTCTCGCTCGT
CGCCGAAGAGGGCGGCCGTGGGCAAGAAGACCGGCGCGAAGAAGGTTGCGAAGAAGACCTTCGCGAAGAA
GGTTGCGAAGAAGCCAGCGAAGAAGGTTGTGAAGAAGCCAGCGAAGAAGGTTGTGAAGAAGCCAGCGAAG
AAGGTTGTGAAGAAGGCTGTGAAGGCTGTGAAGAAGGCTGTGAAGAAGGTTGTGAAGGCTGTGAAGACCG
CGAAGAAGTCGTCGAAGAAATCCTCGGCGAAGAAGTAA
>U16999 73, 639 L-11
ATGGTCGCCGAGAGCAAGGCTGCGAACCCCATGCGGGAGATCGTCGTGAAGAAGCTCTGCATCAACATTT
GCGTTGGTGAGAGCGGTGATCGCCTGACGCGTGCCTCGAAGGTGCTCGAGCAGCTGTGCGAGCAGACCCC
GGTGCTCTCCCGCGCTCGTCTGACCGTGCGCACGTTCGGCATCCGTCGTAACGAGAAGATCGCCGTGCAC
TGCACCGTCCGCGGCAAGAAGGCCGAGGAGCTGCTGGAGAAGGGCCTCAAGGTGAAGGAGTTCGAGCTGA
AGAGCTACAACTTCGCCGACACCGGCTCCTTCGGCTTCGGCATCGACGAGCACATTGATCTCGGCATCAA
GTACGACCCCTCCACCGGTATCTACGGCATGGACTTCTACGTCGTCCTCGGCCGCCGTGGTGAGCGAGTG
GCGCACCGCAAGCGCAAGTGCAGCCGCGTCGGCCACTCCCACCACGTCACCAAGGAGGAGGCGATGAAGT
GGTTCGAGAAAGTCCACGACGGTATCATCTTCCAGGCCAAGAAGAAGAAGATGATCCGCCGCCGCCG
TCGCTAA
>U27568 72, 1010 LACK
ATGAACTACGAGGGTCACCTGAAGGGTCACCGAGGATGGGTCACCTCCCTGGCCTGCCCGCAGCAGGCGG
GGTCGTACATCAAGGTGGTGTCGACGTCGCGCGATGGTACGGTCATCTCGTGGAAGGCCAACCCCGACCG
CCACAGCGTGGACAGCGACTACGGTCTGCCGAACCACCGCCTCGAGGGCCACACCGGCTTCGTGTCGTGC
GTGTCGCTGGCCCACGCCACCGACTACGCGCTGACCGCGTCCTGGGACCGCTCCATCCGCATGTGGGACC
TGCGCAATGGCCAGTGCCAGCGCAAGTTCCTGAAGCACACCAAGGACGTGCTCGCCGTCGCCTTCTCGCC
GGACGACCGCCTGATCGTGTCCGCGGGCCGCGACAACGTGATCCGCGTGTGGAACGTGGCGGGTGAGTGC
ATGCACGAGTTCCTGCGCGACGGCCACGAGGACTGGGTGAGCAGCATCTGCTTCTCGCCGTCGCTGGAGC
ACCCGATCGTGGTGTCCGGCAGCTGGGACAACACCATCAAAGTATGGAACGTGAACGGGGGCAAGTGTGA
GCGCACGCTCAAGGGCCACAGCAACTACGTGTCCACGGTGACGGTGTCGCCAGACGGGTCTCTGTGCGCA
TCTGGCGGCAAGGACGGCGCGGCGCTGTTGTGGGACCTGAGCACCGGTGAGCAGCTGTTCAAGATCAACG
TGGAGTCGCCCATCAACCAGATCGGCTTCTCGCCCAACCGCTTCTGGATGTGCGTCGCGACGGAGAGGTC
TCTGTCCGTGTACGACCTGGAGAGCAAGGCCGTGATTGCGGAGCTGACGCCGGACGGCGCGAAGCCGTCG
GAGTGCATCTCCATTGCCTGGTCCGCCGACGGCAACACTCTGTACTCCGGCCACAAGGACAACCTGATCC
GCGTGTGGTCCATCTCCGACGCCGAGTAA
>U29952 377, 715 infective insect stage-specific protein
ATGGAGATGAAAAACTTGCTTGGCAAGCACAAGATTGTGTCCGTCAACGGCAAGCCTGCACCGGCGGGTG
TGACGGTGGAGTTCAAGGCCAGCGAGAACAGCGGATCCGTTTACATGCACGCGAAAGTGGCGAACATCAT
GAACGGCCCGTTGAAGCTCGCAAACCGCAAGCTCTCTGGTGCTCTTGTGTCAACGATGATGTTGGGCAGC
GACGACCTGATGAATATCGAGAACGCCCTCAGTCAGGGATTCATGGAGGGCATGACATACACGGTGAAGG

ACGGCGGCAAGTTGACGCTGCAGTCTAAAACACATATCATCATGCTTGTTCCTGCGTAG
>U30455 292, 1536 squalene synthase
ATGGGGTTCTTCTCGGATTCGGTAGCGATGATGCGTGTAAAGTGGCAGATGCGCTCTGTGAAGATTCAGG
TGCCGCCGGAAGAGACGGACTTACGCTTCTGCTACGATATCATGAACGACGTCTCTCGTTCCTTCGCGGT
TGTCGTGGCGCAGCTCGCAGACCAGCAGCTCCGTGATGCCATCTGCATCTTCTACCTTGTCCTCCGTGCG
CTGGATACCTTGGAGGATGACATGAGCGTTCCGGTAGACGTTAAGCTGAAGGAGCTTCCGAAGTTCCACA
CGCACACGAGCGACATGAGCTGGTGCATGAGCGGCGTCGGTGAGGGACGCGAGCGCGAACTGCTGGCGAA
ATACCCGTGCGTCTCGCGCGAATTCAAGAAGCTGAAGAAGGAGTATCAGGACGTCATCGCAAATATCTGC
GAGCGCATGGCAAACGGCATGTGCGAGTTCTTGAAGCGCCCAGTGGTCACAAAGGACGACTACAACCAGT
ACTGCCACTATGTCGCCGGCCTCGTCGGCCACGGCCTCACCCAGCTCTTCGCCCGCTGCGGCTTCGAGGA
CCCGAGCCTGGACGACGATCTCACCAGTTCCAACCACATGGGTCTCTTCCTGCAGAAGACAAACATCATC
CGCGACTACTACGAGGACATCCGAGAGGAGCCGCCGCGGATGTTCTGGCCGAAGGAGATCTGGGGCACGT
ACGTGACGGAGCTGAAGGAGCTGAAAAGTGAGTCGAACAATGCGGCAGCGGTGCAGTGCCTGAACGCCAT
GGTGGCCGACGCTCTCGTGCATGTCCCCTACATCGTGGACTACCTCTCCGCTCTGCGCGACCCGTCCGTG
TTCCGTTTTTGCGCCATTCCTCAGGTGATGGCCATTGCGAGCGTGAAAGAGGTGTACAACAACCCCGACA
CGTTCCAGGTCAAGGTGAAGGTGTCACGGCCGGAGTCGTGCCGTATCATGCTCAAGGCCACGACCCTCTA
CAGCTCCCTCAGCATCGTTCCGCGACTACTGCGTGGAGCTGCAGGAGAAGCTAGACATGCAAGACGCAGC
TCCGTATCGATCGGCAACTCGCTTGCGGCGGCGATTGAGCGGATCGACCTGCAGCTGAAGAAGTGCCAGG
ACGTCTCCTACACCCGCAGCCTCCTCGCCCGCTACCCAGGCCTCGGTGGTCAGTTCCTTCTCACCGTCAT
GGATACCGTCGCTGGTTTCTTTGGTGGCCGCAAGGAGATTGCCGGCCACGCGTAA
>U43705 1, 1023 cathepsin B-like protease
ATGGCCCTCCGCGCCAAGTCTGCGCTGTGCCTGGTGGCCGTGTTCGCCCTGCTGCTGGCCACCACGGTGA
GCGGCCTCTACGCCAAGCCGAGTGACTTTCCGCTTCTCGGCAAGAGTTTTGTGGCGGAGGTCAACTCAAA
GGCGAAGGGTCAGTGGACCGCCTCGGCCAATAATGGCTACCTGGTCACGGGCAAGAGCCTCGGGGAGGTG
CGCAAGCTGATGGGTGTGACCGACATGAGCACCGAGGCTGTCCCTCCCCGCAACTTCTCTGTGGAGGAAC
TGCAGCAAGACCTACCAGAATTTTTTGACGCCGCCGAGCATTGGCCCATGTGCTTGACGATCAGCGAGAT
TCGTGACCAATCGAACTGCGGCTCGTGCTGGGCCATCGCGGCGGTGGAGGCTATTTCGGACCGCTACTGC
ACCTTCGGTGGCGTTCCGGATCGCCGCATGTCGACCAGCAACCTTCTCTCCTGCTGCTTCATATGCGGCC
TTGGCTGCCACGGCGGCATTCCGACGGTAGCGTGGCTATGGTGGGTGTGGGTAGGCATAGCGACTGAGGA
CTGCCAGCCCTACCCCTTTGACCCATGCAGCCATCACGGGAACAGCGAGAAGTACCCGCCCTGCCCGAGC
ACCATCTACGATACCCCTAAATGCAATACCACCTGTGAGAGAAACGAGATGGATCTGGTCAAGTACAAGG
GCAGCACATCTTACTCCGTCAAGGGCGAGAAGGAGCTCATGATTGAGCTCATGACCAACGGCCCCTTGGA
GTTGACCATGCAGGTGTACTCTGACTTCGTCGGCTACAAGAGTGGTGTCTATAAGCACGTCCTTGGTGAT
TTTCTCGGCGGACACGCTGTAAAACTGGTCGGTTGGGGAACCCAAGACGGTGTCCCGTACTGGAAGGTTG
CCAACAGCTGGAACACCGACTGGGGTGACAAAGGCTACTTCTTGATCCAGCGCGGCAACAATGAGTGCAA
AATTGAGAGTGGCGGCGTTGCTGGCATACCCGCGCAGGAGTAG
>U43706 1, 1332 cathepsin L-like protease
ATGGCGACGTCGAGGGCCGCTCTCTGCGCTGTTGCGGTTGTGTGCGTGGTGCTTGCGGTTGCCTGCGCGC
CCGCGCGCGCGATATACGTGGGCACGCCGGCTGCTGCGCTGTTCGAGGAGTTCAAGCGGACGTACCAGCG
CGCGTACGGGACGCTGACCGAGGAGCAGCAGCGGCTGGCGAACTTCGAGCGCAACCTGGAGCTGATGCGC
GAGCATCAGGCGAGGAACCCACACGCGAGGTTCGGGATCACGAAGTTCTTTGACCTGTCGGAGGCGGAGT
TCGCCGCGCGCTACCTGAACGGCGCCGCGTACTTCGCAGCGGCGAAGCAGCACGCCGGCCAGCACTACCG
CAAGGCGCGCGCGGACCTGTCGGCGGTGCCTGATGCGGTGGACTGGCGCGAGAAGGGCGCCGTGACGCCG
GTGAAGAATCAGGGTGCGTGCGGGTCGTGCTGGGCGTTCTCGGCGGTCGGCAACATCGAGTCGCAGTGGG
CCGTTGCCGGCCACAAGCTGGTGAGGCTGTCGGAGCAGCAGCTGGTGAGCTGCGATCACGTGGACAATGG

TTGCGGCGGCGGGCTGATGTTGCAGGCATTCGAGTGGGTGCTGCGAAACATGAACGGGACCGTGTTCACG
GAGAAGAGCTACCCCTACGTCTCCGGCAACGGTGATGTGCCCGAGTGCTCGAACAGCAGTGAACTCGCTC
CCGGTGCGCGAATCGACGGGTACGTGTCGATGGAAAGCAGCGAAAGAGTTATGGCTGCGTGGCTTGCGAA
GAATGGCCCCATCTCGATTGCGGTCGACGCCAGCTCCTTTATGTCTTACCATAGCGGCGTCCTGACCAGC
TGCATTGGTGAGCAGCTGAACCATGGCGTGCTGCTCGTTGGGTACAACATGACTGGTGAGGTTCCGTACT
GGGTGATCAAGAACTCGTGGGGTGAGGACTGGGGCGAGAAGGGCTACGTGCGCGTGACCATGGGGGTGAA
CGCGTGCCTGCTCACTGGGTACCCCGTGTCCGTGCATGTGTCGCAGAGCCCCACCCCTGGCCCAAACACG
ACCACCACGACGCACGCTCCTAAACGGGTGACGGTGAAGCAGATCACCTGCACGGATTATTTCTGCCGAA
AGGGGTGCAAGACGACGGTGATCCCCACGAAAGAGTGCCTGCCGAACGGGGCAGGCGGCTCTTTTCAGAT
GGAGTGCGGTGACCATCAGGTGTTGAAGCTCACCTACACCTCCATGAATTGCACTGGTGAGGCCAAGTAT
ACGGTGACAAGGGAGGGTAAGTGCGGGATATCGTGGTCCGGCTCGAGCAAGAGCATTTGCCAGTACGTGT
AG
>U43906 1222, 2220 pkac1
ATGATCACCAAGGCCGAAGCTTCCCAATGGAGGCTGAGCGACTTGGAGATGCGTGAGACGGTCGGCACCG
GCACTTTCGGCCGAGTACGCTTGGTGAAGCACAAAGGAACAGGCCAGTACGCGGCCCTCAAGATCCTGAA
GAAGCAGGAGATTCTCCGAATGAAGCAGGTCGACCACGTGATGGCGGAGGCAAGTCTTCTCCAGGAGATC
GACCACCCTTTTATTGTGAGCATGCTGCGGGGATACATGGACAAGAACCGGCTGTACATTCTCCTCGAGT
ACGTGGTAGGCGGCGAGCTGTTTTCGCATCTCCGCAAGGCCGGCAAGTTCCCTAATGATGTCTCGAAGTT
CTACTGCGCCGAGGTGATCCTAGCATTTGACTACCTGCACAACAAGACGATCGTTTACCGTGATCTGAAG
CCGGAGAACATTCTCCTCGACCAGGACGGCAACATAAAGATCACGGATTTCGGATTCGCGAAGCGCGTGA
CGGAGCGTACGTTTACGCTTTGTGGCACACCCGAGTACCTAGCACCCGAGATCATTCAGAGCAAGGGGCA
CAACAAGGCTGTCGACTGGTGGGCGCTCGGCATCCTGCTGTACGAAATGCTGGTTGGCTATCCGCCGTTC
TTCGACGACAGCCCAATGAAGATCTACGAGAAGATCCTTGTGGGCAAGGTGCTGTTCCCACGATGGGTAG
ACTCGAAGGCCCGAGACTTCATCAAGGGCCTTCTATCCCTCGATCCCACGAAGCGCCTCGGCAATTTGCC
CAACGGCACGGAGGACATCAAGAACCACAAGTACTTTGCGGAAGTGGACTGGAATGTAGTGCTGTCCAAG
AAGATTCCCGCGCCAATCCCAGTGCGGCAACACAAGGAGGGCGATACCCACTACTTCGACAAGTACCCCG
ACAGCCCACTCAACCCTTTGCGCACTTTGACTCCAGCACAGCAGGACTGCTTCGCCAACTTCTGCAACGG
CCAGTATACGGACGAGTAG
>U51890 1, 502 RNA polymerase II largest subunit
NGACGTCCGACACCGGATACCTGCAGCGCAAGCTCGTGAAGGCGCTGGAGGATGTGCACGCCTCCTACGA
CGGCACGGTGCGGAACGCGAACCAGGAGCTCATCCAGCTGGCGTACGGCGAAGACGGTCTGGACGGTGCT
CGTATTGAGGGCAACCAGGCCTTCCCGATCCCGCACATGACGAACAGTGAGATGGCTGACAAGTACCGCT
ACGAGTACAACGACGAAGGCAGCTTCTCGGAGAACATGGGCGGCCATTACATGGATCCGTTTGTGCGCGA
CTCGCTGCTGCGCGACCCGCAGAGCGTGTTGAAGCTGCAGGAGGAGTTTGAGCAGCTGATGAAGGATCGC
GCGATGTCGCGGCTCGTGATCGACATGGAGGACAAGAACAAGCTCAAGATGAACCTGCCCGTGAACGTCG
CGCGGCTCATCCAGAACGCGCGCACGACGATGGGCAAGCGCAGCCAGGTGTCCAATCTGAACCCGATCAC
TGTGATCAACCG
>U59320 1708, 3477 hsp60
ATGCTCTCCCGTACGGTGCCTCGCTGTGTCAAGTACGGCTCAACGCCGAAGGACATCCGCTATGGCATGG
AGGCCCGCAACGCCCTGCTTGCCGGCGTCGAGAACCTTGTCAAGGCCGTCGGCGTCACGCTGGGCCCCAA
GGGCCGCAACGTGATTCTGGAGATGCCATACGCATGCCCGAAGATCACGAAGGATGGAGTCACGGTGGCC
AAGAGCATCGAGTTCGAGGACAGCTTCGAGAACCTTGGGGCGAACTTGGTCCGTCAGGTGGCAGGGCTGA
CCAACGACAACGCCGGTGACGGCACCACCACCGCTACTGTCCTCTCCGGGGCGATCTTCAAGGAGGGCTT
CCGCAGCGTTGCAAGTGGCACGAACCCGATGGACCTGAAGCGGGGCATCGACCTCGCATGCCGGGAGGTG
CTCATATCGCTGGCCGAGCAGTCTCGCCCAGTCACCTCCAAATCTGAGATCACCCAGGTAGCGATGATTT

CGGCGAACATGGACCAAGAGATCGGCAGCCTGATCGGTGACGCGATGCAGCAGGTCGGCAAGGATGGCGT
AATCACAACGCAGGAGGGTCGCTCGCTTAATACGGAGCTCGAGCTGGTCGAGGGGATGTCCTTTGAGCGT
GGGTACACGTCGCCGTACTTTGTGACGAACACGAAGGCGCAAAGGTGCGAGCTCGAGAACGCCCTCGTCT
ACGTGGCGAACCGCAAACTCACGAGCGTGGCGCACATCTTGCCCGCGCTGAACTACGCCATTCAGCAGAA
GCGTCCGCTTCTGGTGATTGCCGAGGACGTGGAGGGGGAGGCCATGCACACGTTCCTGTACAACAAGATT
CAGGGCCGCATCAGTGGCTGCGCCGTCAAGGCCCCGGGCTTTGGCGACATGCGCATCAACCAGCTGCAGG
ATATCGCCGTCTTCACGGGGTCGCAGATGATCTCAGAAGACCTAGGCCTTTCACTGGACCAGAACGATTT
CTCCGAGCGGTTCCTCGGCACTTGTCGAAAGGTGACCGTCTCCCGTGACGAGTGCATCTTGATGGAGGGC
GGCGGCAGCGCCATCGCGGTGGAGGAGCGAGTGCAGATGATCAAAGACATGATCTCCGCCGAGGACCACG
AATACAATCGCGAGCGTCTGGTCGAGCGTCTCGCGAAGCTCTCGGGTGGGGTTGCGGTGATCAAGGTCGG
TGGCGCCTCGGAGGTGGAGATCAATGAGAAGAAGGACCGCATCATCGACGCGCTCAACGCCACGCGCGCG
GCTGTATCAGAGGGCATCCTCGCAGGTGGTGGCACCGGGTTGCTTATGGCCTCTCTGCGTCTCGAGAGCA
TCTCCAAGGACCGACGACTTCCACCTGACATCCGCACTGGCGTGAACATTGTGAAGAAGGCAATCGGCCT
GCCCGCGCGCTACATCGCGAACAACGCAGGCGTCGAGGGCAGCGTGGTGGCGGGGAAGGTACTCGCGCGC
AAGGATCCGAGCTTCGGCTACAACGCGCAGACCGGCGAGTATGTGAATATGTTCGAGGCTGGCATTATCG
ATCCCATGAAAGTAGTGAAGTCCGCCGTCGTCAACGCATGCTCCGTGGCGGGCATGATGATTACGACGGA
GGCGGCAGTGGTGGAGAAGGACCTGCTAGGGCGAGAGAAACGCATCGAGGATGAGGGGATGGAGGACAAG
GAGAAGAAGCGCAGCGTCGACAAACTGCGCAAGCAGGTAAATGAGGCGGGACGCACCGATGCCCAAGATG
GCGCCGCCGATGAAGTTTGA
>U73845 1, 1640 protein antigen LmSTI1
TGGACGCAACTGAGCTGAAGAACAAGGGGAACGAAGAGTTCTCCGCCGGCCGCTATGTGGAGGCGGTGAA
CTACTTCTCAAAGGCGATCCAGTTGGATGAGCAGAACAGTGTCCTCTACAGCAACCGCTCCGCCTGTTTT
GCAGCCATGCAGAAATACAAGGACGCGCTGGACGACGCCGACAAGTGCATCTCGATCAAGCCGAATTGGG
CCAAGGGCTACGTGCGCCGAGGAGCAGCTCTCCATGGCATGCGCCGCTACGACGATGCCATTGCCGCGTA
TGAAAAGGGGCTCAAGGTGGACCCTTCCAACAGCGGCTGCGCGCAGGGCGTGAAGGACGTGCAGGTAGCC
AAGGCCCGCGAAGCACGTGACCCCATCGCTCGCGTCTTCACCCCGGAGGCGTTCCGCAAGATCCAAGAGA
ATCCCAAGCTGTCTCTACTTATGCTGCAGCCGGACTACGTGAAGATGGTAGACACCGTCATCCGCGACCC
TTCGCAGGGCCGGCTGTACATGGAAGACCAGCGCTTTGCCCTGACGCTCATGTACCTGAGCGGAATGAAG
ATTCCCAACGATGGTGATGGCGAGGAGGAGGAACGTCCGTCTGCGAAGGCGGCAGAGACAGCGAAGCCAA
AAGAGGAGAAGCCTCTCACCGACAACGAGAAGGAGGCCCTGGCGCTCAAGGAGGAGGGCAACAAGCTGTA
CCTCTCGAAGAAGTTTGAGGAGGCGCTGACCAAGTACCAAGAGGCGCAGGTGAAAGACCCCAACAACACT
TTATACATTCTGAACGTGTCGGCCGTGTACTTCGAGCAGGGTGACTACGACAAGTGCATCGCCGAGTGCG
AGCACGGTATCGAGCACGGTCGCGAGAACCACTGCGACTACACAATCATTGCGAAGCTCATGACCCGGAA
CGCCTTGTGCCTCCAGAGGCAGAGGAAGTACGAGGCTGCTATCGACCTTTACAAGCGCGCCCTTGTCGAG
TGGCGTAACCCTGACACCCTCAAGAAGCTGACGGAGTGCGAGAAGGAGCACCAAAAGGCGGTGGAGGAAG
CCTACATCGATCCTGAGATCGCGAAGCAGAAGAAAGACGAAGGTAACCAGTACTTCAAGGAGGATAAGTT
CCCCGAGGCCGTGGCAGCGTACACGGAGGCCATCAAGCGCAACCCTGCCGAGCACACCTCCTACAGCAAT
CGCGCGGCCGCGTACATCAAGCTTGGAGCCTTCAACGACGCCCTCAAGGACGCGGAGAAGTGCATTGAGC
TGAAGCCCGACTTTGTTAAGGGCTACGCGCGCAAGGGTCATGCTTACTTTTGGACCAAGCAGTACAACCG
CGCGCTGCAGGCGTACGATGAGGGCCTCAAGGTGGACCCGAGCAATGCGGACTGCAAGGATGGGCGGTAT
CGCACAATCATGAAGATTCAGGAGATGGCATCTGGCCAATCCGCGGATGGCGACGAGGCGGCGCGCCGGG
CCATGGACGATCCTGAAATCGCGGCAATCATGCAAGATAGCTACATGCAACTAGTGTTGAAGGAGATGCA
GAACGATCCCACGCGCATTCAGGAGTACATGAAGGACTCCGGGATCTCATCGAAGATCAACAAGCTGATT
TCAGCTGGCATCATTCGTTTTGGTCAGTAG
>U91743 1500, 2615 LmPKAC2b

ATGTCGTCTGCTGCCAAGGACAGTTATCCGGCGGAGTCGTCGGGCCACCTCAACAACGAGAAGGCGCAGC
TGTCGAAGATTCCCTCCAGTCATCCAGACCCCTCGAAGGTGACTGTCGCGCCAAACGTCAAGGCCGCTTA
TTACTTCGCAAAGCCAGACACCTCCAGCTGGAAACTCTCAGATTTCGAACTGAAGAACACGCTCGGCACC
GGTTCCTTCGGCCGCGTGCGCATCGCCCACCGCAAGGGCACGGAGGAGTACTACGCGATCAAGTGCCTGA
GAAAGCGCGAGATCATCAAGATGAAGCAGCAGCAGCACGTTGCGCAGGAAAAGGGGATCCTAATGGAGCT
GTGTCACCCGTTCATCGTGAACATGATGTGCTCCTTCCAGGACGAGAAGAAGGTGTACTTTCTGCTGGAG
TTTGTCATGGGCGGCGAGATGTTCACGCACCTGCGCACTGCCGGGCGGTTCCCGAATGACGTTGCGAAGT
TCTACCACGCGGAGCTGGTGCTTGCGTTCGAGTATCTGCACTCGCTGGACGTGATCTACCGCGATCTGAA
GCCGGAGAATCTGCTGCTGGACAACAAGGGGCATGTGAAGATGACGGACTTTGGGTTTGCGAAGAAGGTG
CCGGACCGGACGTTCACGCTGTGCGGGACACCGGAGTACCTTGCGCCGGAGGTGATCCAGAGCAAGGGCC
ACGGGAAGGCGGTGGACTGGTGGACGATGGGCGTGCTGCTGTACGAATTCATTGCCGGATACCCGCCGTT
CTACGACGACACGCCGTTCCGGATTTACGAGAAGATCCTTGCTGGGCGGCTGAAGTTCCCGAACTGGTTT
GACGGCCGCGCGCGCGACCTCGTGAAGGGGCTGCTGCAGACGGACCATACGAAGCGCCTGGGGACGCTGA
AAGGCGGGCCGGCGGACGTGAAGAACCATCCGTACTTCCACGGTGCGAACTGGGACAAGCTGTACGCACG
TTACTACCCCGCGCCGATCCCTGTGCGGGTGAAGAGCCCTGGCGACACGAGCAACTTCGAGAAGTACCCG
GACAGCCCGGTCGACCGCACGCCTGCGCTGACATCGGCGCAGCAGGCCGAATTCAATGGCTTTTAG
>U91744 582, 1487 Lmairk
ATGACGACAGAGGTCGGCCCGGACGACAATGTCGTGAACAACTTCATCATTACCCCGTCGTCCCCAAAGT
CGGAGTGGACGATACACGACTTTGAGCTGCTGCACAAGCTGGGAGGAGGCAATTACGGTGATGTCTACCT
GGCGAGTGTCCGCAAGAGCAACTACGTCGTGGCCATCAAGAAGCTCTCGATCAAGAAGCTGGCAGAGTTT
GACATTGTGAACCAGCTGCGGCGCGAGATCGAGATCGCCTTCAACACGCGACACAAATACCTCCTACGCA
CCTACGCCTACTTCTTCGATGAGCACGACATCTACCTTATCCTGGAGCCGTGCAGCAACGGCATGCTCTA
CAGCGAGCTGAATCGCGTGAAGCTGTTCCCGCCGCCGACGGCAGCCCGCTATGTGGCGCAGCTCGCCGAG
GCACTTCTTTACCTCCACCAGCACCACATTCTGCACCGCGACATCAAGCCGGAGAACATTCTGCTGGATC
ATCATCAGAACATCAAACTCGCCGATTTCGGCTGGTCTGTGCATGACCCGCTGAACCGGCGCAAGACCTC
CTGCGGCACGCCCGAGTACTTCCCCCCCGAGATCGTTAGCCGGCAGATGTATGACATGAGCGCCGATCTC
TGGTGCCTGGGCATCTTCTGCTTTGAACTGCTTGTGGGCCACACACCGTTCGTGAGCAAGGACAACGACC
AAATCTACAAGAAGATCCATGCGATGCAGTACACCATCCCCGACAGCGTCCCACCAGAGGCGAAGGATTT
GATTTCAAATCTGCTCATCCGCGAGGGGTCGAAGCGGCTGGCGCTACATCGCGTGCTGAGCCACCCCTTC
TTGCTCAAATACTACTATGTTCCAAACGGCATTACGCCGCCGACGGGAAAGCGGCCGCGCAGCTAG
>U92487 1, 1344 LmAP
ATGGCCTCGAAGCGATGCCGGCAGTGCAGCGGCGACTCTGCGTCCTCATCGACCTCGTCGTTGTCACCAT
CCGAGCTGCCGCCCTCGAAGAAGGCGGCTGGCGGTCAGCGAGTCACGGCGGAAGTTGAAGTGGCGCCCAT
CACAACGGACGCTACGTCTGCCACTGTCACGGCTGCAGGCGGCGCGAAGAAAAAGGCGACGACGGGGAGC
CCCGCACGTCGCACGAGCAGCGCGGCAAAGATCACGAACGGTGACGCCGGTGAGCTGATCCGCACCGCGG
AGGCCCTCGCCGCTCTCAACGCGAAGAAGTCCGAGAAGGAGATTTGGTCTGACGTGGTGCCGTTCGTGCG
CAGGACCACGGACAGCGACTTCGACCCGTCGCGCATGTACAAGTTCATTACGTGGAACGTAGCCGGCCTA
CGCGGGCTGCTGAAGAAGAACGCCTCGGCGCTGAGAGCGTTCATGGAGGCCGAGAAGCCGGATGTTCTGT
GCCTGCAGGAGACCAAGCTGAACGTCGACGAGGCCGATGCCAATGCGACCCTCGGCGTGGTGGACGGCTA
CTCCTTTGTGGACCACCCGTGCGCCTTCAAGCGGGGCTACTCGGGCACCCGCACCTACATGAAGAACAGC
ACCACTGTGAAAGGGCTGCACGCACGGTGCACTCGTGGCTTTGCATTGCCGTCCGAACCACAGGCAGACG
CGGCCGCCGGCTCACGGGTGCTGGTAGAAGGCGCTGGCGACGAGGAGGGCCGCGTGTTGACTACATTCCT
CTCTCCGGATCCGGACTCTGCATCGTCGTCGTCGCGCATCGCCCTCGTGAACACCTACGTGGCGAACAGC
GGCATGGGACTGACGCGGCTGCCTTACCGCGTCCAATCCTTTGATCCCAGCATGAGGGAGTACCTTCACC
GGCTGGACACCTGGGCCACAGAAAATGCCGCCGTCCCGTCGGCGGCGGCGATGGGCAGCGGCAGCAGCCC

GCACGGCTTTATCTGGGCTGGCGACCTCAACGTGGCGGAGCGGGACTACGACCGTTACTACGCCGGTACG
TTCAAGTCAATGCAGGAGTGCAGCGGCTTTGCACCTGAGGAGCGGATGTCTTTTCGCGAGACGATGCAGC
GGACGAATTCGGTGGACATCTTTCGCCAGCTCTACCCCCAGGCAGGCCCGGTCTACTCCTTCTGGTCTCA
GCGTATCAACGGTCGCCCGAGAAACTTGGGGTGGCGCTTGGACTACTTCGTTGTTTCCTCGCGACTTGCA
TCGTACGTCGTGGACTGCTTCCCGATGCCGACGGTGATGGGCAGCGATCACTGTCCGTTCCAGATGTGGA
TGCGACACCCATGA
>X06555 1, 645 antigen (214 AA)
CAGGAGACGTCCGCGAAGCTCGCTGACACCGAGGAGACGCTGCAGGAGACATCCGCGAAGCTCGCTGACA
CCGAGGAGACGCTGCAGGAGACATCCGCGAAGCTCGCCGACACCGAGGAGACGCTGCAGGAGACGTCCGC
GAAGCTCGCTGACACCGAGGAGACGCTTTCAGCGGAGATCGCGTCGTTGGCGGAGCAGCATGGTGTGGCG
CAAACTTTATGTGAGCAGCAGGGCGCGTACATGAACCAGCTGCTGACGTACGTGAGGGCAGCATACAACG
TGACTCTGGATGGGATGACGGAGGTGCAGGCGACAGCTGTGGCTGATATCCTGAGGCAGTGCGACTATGC
ACTTCGCTCTGTGGCGCAGGCTGCCAGTGACGAGGTGGAGCACGTAAAGGAGATGTACAATATGGTCGAC
GGCAACTACAAGGAGACGGCAGCGCGCTTCGATATACTGCGCCGCATTCTGGGAAAGATCGATCAGGATG
TCAAGGAGGCGAGCGTGGAGATCGACGCACCGGAGCGGCGGCAGACCTGCTCTTTACACCCGACGCCGCG
GCGCTTGGGCGACGTCTCGAACCGCGAGAACAGCATCGAGAACAAGACCCGCTCCGCATCTCGGCTGTCT
GGACGGCTATTTTAG
>X14574 1, 749 Leishmania major hsp70.1 gene for
heat shock protein 70-related
ATGTTTGCTCGTCGTGTGTGCGGAAGCGCTGCGGCGTCGGCTGCGTGCCTGGCGCGCCACGAGTCGCAGA
AGGTGCAGGGCGACGTGATTGGCGTGGACCTGGGCACGACGTACAGCTGCGTGGCGACGATGGACGGCGA
CAAGGCGCGCGTGCTGGAGAACTCGGAGGGCTTCCGGACGACGCCGTCTGTTGTGGCGTTCAAGGGCAGC
GAGAAGCTTGTGGGGCTTGCGGCGAAGCGGCAGGCGATCACGAACCCGCAGTCGACGTTCTATGCTGTGA
AGCGGCTGATCGGGCGCCGGTTCGAGGACGAGCACATCCAGAAGGACATCAAGAACGTGCCGTACAAGAT
CGTGCGCGCGGGGAACGGTGACGCGTGGGTGCAGGACGGGAACGGGAAGCAGTACTCGCCGTCGCAGATC
GGCGCGTTCGTGCTGGAGAAGATGAAGGAGACGGCGGAGAACTTCCTGGGGCACAAGGTGAGCAACGCCG
TCGTGACGTGCCCGGCGTACTTCAACGACGCGCAGCGCCAGGCGACGAAGGACGCGGGGACGATCGCGGG
CCTGAACGTGATCCGCGTGGTCAACGAGCCGACTGCTGCGGCTCTTGCGTACGGCATGGACAAGACGAAG
GACAGCCTGATCGCGGTGTACGACCTCGGTGGCGGCACGTTCGATATCTCCGTGCTGGAGATCGCTGGCG
GCGTGTTCGAGGTGAAGGCGACGAACGGCGACACGCACCTTGGCGGCG
>X14575 1, 685 Leishmania major hsp70.4 gene for heat
shock 70-related protein,
ATGTCGTCTACCAAGCATCGCATCGACCTGGGCACGACGTACTCGTGCGTCGGTGTCTTCAAGAACGAGC
AGGTGGACATCATTGCCAACGACCAGGGTAACCGCACAACGCCGTCTTATGTGGCCTTCACCGAGACAGA
GCGCCTGATCGGCGATGCGCGAAGAACCAGGTGGCATGAACCCCCGGGCAACACCGTGTTTGACGAAGCG
CATGATCGGCCGCAAGTTCGACGACCTGACCTTCAGTCGGACATGAAGCACTGGCCCTTCAAGGTGATCG
TCAAGGACGGCAAGCCGGTCATCAGCGTCGAGTACCAGAACCAGACCAAGACCTTTTCCAGAGGAGATCT
CGGCTATGGTGCTGCAGAAAATGAAGGAGACGGCGGAGGCTACCTCGGCACGACCGTCAAGGACGCCGTC
ATCACCGTCCCGGCGTACTTCAACGACTCGCAGCGCCAGGCAACGAAGGACGCCGGCTCCATCGCTGGCC
TCAACGTGCTGCGCATCATCAACGAGCCCACCGCCGCCGCTATCGCGTACGGCATGGACCGCAAGGGAGA
CAAGGGGGAGAAGAATGTGCTCATCTTCGACCTTGGCGGCGGCACGTTCGATGTGACGCTGCTGACGATC
GAGTCCGGCGTGTTCGAGGTAAAGGCAACCGCGGGTGACACGCACCTTGGCGGC
>X51733 953, 2515 Leishmania major DNA for
dihydrofolate reductase-thymidylate
ATGTCCAGGGCAGCTGCGAGGTTTAAGATTCCGATGCCGGAGACGAAGGCAGACTTTGCTTTCCCCTCCC

TGCGCGCCTTCTCCATCGTCGTGGCCCTCGATATGCAGCACGGCATCGGCGACGGCGAGTCGATCCCGTG
GCGGGTGCCGGAGGACATGACGTTCTTCAAGAACCAGACGACGCTGCTGCGCAACAAGAAGCCGCCGACG
GAGAAGAAGCGCAACGCCGTCGTGATGGGCCGCAAGACTTGGGAGAGCGTCCCGGTAAAGTTCCGACCAC
TCAAGGGACGGCTGAACATCGTGTTATCCTCGAAGGCCACCGTCGAGGAGCTTCTGGCGCCGCTGCCGGA
GGGACAGCGCGCGGCGGCGGCGCAGGATGTGGTGGTGGTGAACGGCGGTCTGGCCGAGGCGCTCCGCCTC
CTCGCACGCCCGCTGTACTGCAGCTCCATCGAGACAGCGTATTGCGTCGGTGGTGCGCAGGTTTACGCGG
ACGCCATGCTGTCGCCGTGCATCGAGAAACTGCAGGAAGTGTACCTGACCCGCATCTACGCGACGGCGCC
TGCGTGTACGCGCTTCTTTCCGTTTCCGCCCGAGAACGCGGCCACGGCGTGGGACCTGGCGTCGTCTCAG
GGACGCCGCAAGAGCGAGGCGGAGGGCCTCGAGTTCGAGATCTGCAAGTACGTGCCGCGCAACCACGAGG
AGCGGCAGTACCTTGAGCTGATTGACCGCATCATGAAGACGGGGATCGTGAAGGAGGACCGCACCGGCGT
GGGCACCATCAGCCTCTTCGGCGCCCAGATGCGCTTCTCCCTACGCGACAACCGCCTGCCGCTGCTGACG
ACGAAGCGTGTCTTCTGGCGCGGCGTGTGCGAGGAGCTGCTGTGGTTCCTGCGCGGGGAGACGAGTGCGC
AGCTGCTGGCAGACAAGGACATTCACATCTGGGACGGCAACGGTTCGCGCGAGTTTCTCGACAGCCGCGG
CTTGACAGAGAATAAGGAGATGGACCTCGGCCCTGTCTACGGCTTCCAGTGGCGCCACTTCGGGGCAGAT
TACAAGGGGTTTGAAGCGAACTACGACGGCGAAGGGGTGGACCAGATCAAGCTCATCGTGGAGACCATCA
AGACGAACCCGAACGACCGCCGCCTCCTAGTCACTGCCTGGAACCCGTGCGCGCTGCAAAAGATGGCGCT
GCCGCCGTGCCACTTGCTTGCTCAGTTCTACGTGAACACAGACACGAGCGAGCTATCCTGCATGTTGTAC
CAGCGCTCGTGTGACATGGGTCTTGGCGTCCCCTTCAACATTGCCTCCTACGCGCTGCTCACCATCCTCA
TTGCCAAGGCGACGGGTCTGCGGCCTGGTGAGCTTGTGCACACCCTCGGCGACGCCCACGTCTACCGCAA
CCACGTTGATGCCCTCAAGGCGCAGCTCGAGCGAGTCCCGCACGCGTTCCCGACCCTCATCTTCAAGGAG
GAGCGGCAGTACCTCGAGGACTACGAGTTGACGGACATGGAGGTGATCGACTACGTTCCACACCCGGCGA
TCAAGATGGAGATGGCCGTATAGAGAGAGGGAGGGTGTCATGTGCGTTGTATGCATGCAGCCACCGCCGC
GACGCTGCCACTCACCTGGCTCCCACCTCCTCGTCGCACGACGACCGCCCCTTGCGCAGACTCGTTGGGT
AACCATGAGCGGCGCGGAGGGGTACGCGCCTGCGTTTTCTCGCACATGGCTGCGGCTGATATCCCGCCCG
CACCCGCGCGCGCTCAGGCCGCGTGTCGTCGTGCTCTTTCCATTTTTTTTTTGTGTGTGGTGGTGATTTG
AGCTGCTCTCCGTTGTGTGCTGGGGACCCTCCTTCCCTCGATCTCCTCGTGCGGGGTCTCCGACGCGCAG
ACGCGGTGCGTGCGAGCGTGCACGTGCTGTCGCCGTGGCTGTAGTTGCGAGCGGAGAGAGAGAGAGAGGG
AGGGAGGGAGGGCAGAGGGCAGAGGACATGCGGGTGGGAACGTGCACCGGCCTCGTCTCACGCAGCTGGA
GCCCACGAATGCCACCACCACACCCTTCTTCCCCCCCCCTCCCTCTTCTTCCCACGGCGGCGACGACGAC
GGGGGCTCAGCTCACGCTTCTGTAGGGTATTATATTAAAGCACATGTGCGTGCTGTGCTTCGGCTTCTTG
TTGGTGGTCGTTCGCTTTCAAGTCCACGACTCCTGCCGTTGCTCACCGCCGGGCTGTCTTCTTTCGCCCC
TCCCTCGGTGCCTCTTCCGCCGCCGCACGCGTGCGCTGATCACGCGTCTTGCGTGATGTGCCTGTGTGTA
TACACACCGTGCACGGAGAGAGCGAGCGAGCGAGAGAGAGAGAGGGCGAGAGGAAGAATGGCCGGTGCCT
CGCGTGGGCAAGCGTGCGCGAGTTTGTCGTGTCGCTGCGCCGCGTGGATGTCGCGCAGCGCAAGACCCCG
TGCGCCTGAAACGTGAAGGGA
>X56810 1, 732 surface antigen P2
NGAATTCCGTGCGCTGCAGTCGCTTGATACACTGCGGCTGTCCGGCAGTAAGGTCTCCGGCACGCTGCCG
CCCCAGTGGAGTGGGATGTCGAAGGCCCGGTCCCTGCAGCTGCAGGACTGCGACCTGTCCGGCAGTCTGC
CCTCTTCGTGGTCTGCGATGCCGATGCTGGCTTCCGTCTCTCTTAAGGGCAACAAGTTCTGCGGGTGTGT
GCCGGACTCGTGGGATCAGAAGGCTGGTCTTGCTGTGGACATCGAGGACAAGCACAAGGGCAGCGACTGC
TTGGCTGGGAGGGAGTGCACAACGACCACCACTAAGCCCCCTACCATGACCACGACCACCACTAAGCCCA
CCGCCACGACAACGACCACTACGAGCCCCACTAACTTTCCCCCTACGCCGACGACCACGACTGAGCCGCT
TACCACAACCAGCACTGAGGCACCAGCTGAACCCACAACCACCACTGAGGCACCGGCTGAACCCACGACC
ACTGCTACCCCAACAAACACGCCGACTCCTGCTCCTGAGACGGAGTGCGAGGTGGATGGGTGTGAGGTGT
GCGAGGGGGACTCCGCTGCGAGGTGCGCCAGGTGCCGTGAGGACTACTTCCTGACGGACGAGAGGACGTG

CCTGATGCACAACGATGGCGGTGTTGCTGCTGTGTCGAGCGGAGTGGCAGCAGCAGCTGTTGTGTGCGTG
GCTGTGCTGTTCAGCGTGGGGCTGGCGGCGTG
>X57009 206, 1360 tr-1
AATGGCGCAGTGCGTGCGTCGGCTGGTGCTCGGCGACGCTCGCCGCTGCGGTGGCGCTGCTGCTGTGCAC
GAGCAGGCTCGGGTGGCGCGTGCTGCTGGGACGGGCGACTTCACTGCGGCGCAGCGGACGAACACGCTGG
CGGTGCTGCAGGCGTTTGGGCGTGCGATCCCTAAGCTTGGGGAGAAGTGGGCGGGCAACGACTTCTGCTC
GTGGGAGGCCGTCTTGTGCAATGCGCCGGACGTGTACGTGTCGGGAATCAGTCCGACGTATGCCGGCACG
CTGCCGGAGATGCCAGAGAACGTCGACTACAGGCACGTCGTGATCAGGCGGCTCGACTTTTCCGAAATGG
GGCCGGGGCTGAGCGGGACCGTGCCCGCCTCATGGCACTCGATGACATCTTTGGAGTCGTTGTCGATTGA
AAAGTGTGAAAGCATCTCCGGCAGTGTGCCCCCCGAGTGGGGCTCGATGACATCGCTGAGTGTTCTCAAT
CTGCGGGGCACAGGCATCTCCGGCACGCTGCCGCCCCAGTGGAGTGGGATGTCGAAGGCCCGGTCCCTGC
AGCTGCAGGACTGCGACCTGTCCGGCAGTCTGCCCTCTTCGTGGTCTGCGATACCGATGCTGGCTTCCGT
CTCTCTTAAGGGCAACAAGTTCTGCGGGTGTGTGCCGGACTCGTGGGATCAGAAGGCTGGTCTTGTTGTG
GACATCGAGGACAAGCACAAGGGCAGCGACTGCTTGGCTGCTAAGGACTGCGCAACGACCACCACTAAGC
CCTCCGCCACGACAGCGACCACCCCGAACCTCACTAACTTTCCCCCTACGCCGAGGACCACGACTGAGCC
GCTTACCACAACCAGCACTGAGGCACCGGCTGAACCCACAACCACCACTGAGGCACCGGCTGAACCCACG
ACCACTGCTACCCCAACAAACACGCCGACTCCTGCACCAGAGACGGAGTGCGAGGTGGATGGGTGTGAGG
TGTGCGAGGGGGACTCCGCTGCGAGGTGCGCGAGGTGCCGTGAGGACTACTTCCTGACGGACGAGAAGAC
GTGCCTGAAGCACAACGATGGCGGTGTTGCTGCTGTGTCGAGCGGAGTGGCAGCAGCAGCTGTTGTGTGC
GTGGCTGTGCTGTTCAGCGTGGGGCTGGCGGCCTG
>X57134 1, 1117 surface antigen P2
CGAGTGGAGCTGGCTGCCTAATTTACAGACTCTGCGGCTAAGGCGACTAAAACTGAGCGGTACGCTGCCT
GCGGAGTGGAGCTCTTGGAAATCACTGTCGAACGTCTTTCTTGACGACACGCCGATCACAGGCTTGTTGC
CCCCGGAGTGGGGCTCGCTGGAGAGAATACAGCAGCTGGTTTTACGGAAATTGAAGATTACCGGCCCTCT
CCCTCCTCAGTGGAGCCTAATGAAGGTATTGCGGGTTCTAGATCTGGATAGCACGAAGGTATGCGGCACG
CTGCCGGCCGAGTGGAGTCGGATGTCGACGGCTGCATACTTCTGGCTGAACAACTACGACCTGTCCGGCA
CGCTGCCGCCCCAGTGGTCGTCGATGCCATACCTGCGCGGCGTCTCACTGAAGGGCAAGCGCTTCTGCGG
GTGTGTGCCGGAGTCGTGGGCCAACAAGGCTGATCTTGCTGTGGAAATCGAGGACAAGCACAAGGGCAGC
GACTGCTTGGCTGGTAAGGACTGCACAACGACCACCACTAAGCTCCCCACCACGACAACGACCACCACAA
AACCCCCCACCACGACAACGACCACCACTAAGCCCCCCACCACGACCACGACCACCACAAAACCCCCCAC
CACGACAACGACCACCACTAAGCCCCCCACCACGACAACAACCACCACAAAACCCCCCACCACGACAACG
ACCACCACAAAACCCCCCACCACGACCACGACCACCACTAAGCCCCTAACGACAGCGACCACCACTAAAC
CCCCTACCACGACCACGACCACCACTAAGCCGCCGACCACCATAACGAGCACTACTAAGCTGCCAACCAC
AACCACCACTGAGGCACCGGCTGAACCCACGACCACTGCTACCCCAACAAACACGCCGACTCCTGCACCA
GAGACGGAGTGCGAGGTGGATGGGTGTGAGGTGTGCGAGGGGGACTCCGCTGCGAGGTGCGCGAGGAGCC
GTGAGGACTACTTCCTGACGGACGAGAGGACGTGCCTGGTGTACTGCGATGGCGGTGTTGCTGCTGTGTC
GAGCGGAGTGGCAGCAGCAGCTGTTGTGTGCGTGGCTGTGCTGTTCAGCGTGGGGCTGGCGGCGTGA
>X57135 1, 984 surface antigen P2
GAATTCCAGCTGGTTCTAGAGAAATCGAAGCTGACCGGCCCTCTCCCTCCTCAGTGGAGCTCGATGAGAT
CGCTGAGCCTTCTGAACCTGAATGGCGCAAAGGTCTCCGGCACGCTGCCGCCCGAGTGGAGTGGGATGTC
GAAGGCCGCATACTTCTGGCTGAACAACTGCGACCTGTCCGGCACGCTGCCGCCCCAGTGGTCGTCGATG
CCAAACCTGCGCGGCGTCTCACTGAAGGGCAACCGCTTTTGCGGGTGTGTGCCGGACTCGTGGGCCAACA
AGGCTGATCTTGCTGTGGAAATCGAGGACAAGCACAAGGGCAGCGACTGCTTGGCTGGTAAGGACTGCAC
AACGACCACCACTAAGCTCCCCACCACGACAACGACCACCACTAAGCCCCCCACCACGACAACGACCACC
ACAAAACCCCCCACCACGACCACGACCACCACAAAACCCCCCACCACGACAACGACCACCACAAAACCCC

CCACCACGACAACGACCACCACTAAGCTCCCCACCACGACAACGACCACCACAAAACCCCCCACCACGAC
AACGACCACCACGACAACGACCACCACGACCACGACCACCACTAAGCCCCCCATCACGACAGCGACCACC
ACTAAACCCCCTACCACGACCACGACCACCACTAAGCCGCCGACCACGATAACGAGCACTACTAAGCTGC
CCACCACAACCACCACTGAGGCACCGGCTGAACCCACGACCACTGCTACCCCAACAAACACGCCGACTCC
TGCACCAGAGACGGAGTGCGAGGTGGATGGGTGTGAGGTGTGCGAGGGGGACTCCGCTGCGAGGTGCGCG
AGGTGCCGTGAGGACTACTTCCGTACGGACGAGAGGACGTGCCTGGTGTACTGCGATGGCGGTGTTGCTG
CTGTGTCGAGCGGAGTGGCGGCAGCAGCTGTTGTGTGCGTGGCTGTGCTGTTCAGCGTGGGGCTGGCGGC
GTGA
>X62942_0 2107, 4329 homologous to rat ribosomal protein S8
CATGGGCATCGTCCGCAGTCGCCTGCATAAGCGCAAGATCACCGGTGGAAAGACCAAGATCCACCGGAAG
CGCATGAAGGCCGAGCTGGGCCGCCTTCCCGCGAACACCCGCCTTGGCGCCCGCCGCGTGAGCCCTGTGC
GTGCTCGCGGTGGCAACTTCAAGATCCGTGCCCTGCGCCTGGACACCGGCAACTTTGCCTGGGCCTCCGA
GGCCATCGCACACCGCGTGCGTCTTCTCGACGTCGTGTACAACGCCACGTCGAACGAGCTGGTGCGTACG
AAGACGCTGGTGAAGAACTGCATCGTCGCCGTGGATGCCGCGCCGTTCAAGCGCTGGTATGCCAAGCACT
ACGGCATCGACTTGGATGCAGACAAGAAGAGCACCAAGGCCGCCGTTGCTGCGGAGAAGAAGGGCAGGAA
GTCCGCCCACGCCGCTGCTGACAAGTACGACGTGAACAAGGCCTCGCCCAAGCTTCAGCGCGAGTGGACG
CGCCGCCGCCGCAACCACAGGGTCGAGAAGGCGATCGCCGATCAGTTGCGCGAGGGTCGCGTGCTTGCGC
GCATAACGAGCCGCCCGGGCCAGTCCGGCCGTGCCGACGGTATCCTGCTGGAGGGCGCCGAGCTCCAGTT
CTACCTGAAGCGCCTGGAGAAGAAGAAGAAGTA
>X62942_1 3667, 4329 homologous to rat ribosomal protein S8
CATGGGCATCGTCCGCAGTCGCCTGCATAAGCGCAAGATCACCGGTGGAAAGACCAAGATCCACCGGAAG
CGCATGAAGGCCGAGCTGGGCCGCCTTCCCGCGAACACCCGCCTTGGCGCCCGCCGCGTGAGCCCTGTGC
GTGCTCGCGGTGGCAACTTCAAGATCCGTGCCCTGCGCCTGGACACCGGCAACTTTGCCTGGGCCTCCGA
GGCCATCGCACACCGCGTGCGTCTTCTCGACGTCGTGTACAACGCCACGTCGAACGAGCTGGTGCGTACG
AAGACGCTGGTGAAGAACTGCATCGTCGCCGTGGATGCCGCGCCGTTCAAGCGCTGGTATGCCAAGCACT
ACGGCATCGACTTGGATGCAGACAAGAAGAGCACCAAGGCCGCCGTTGCTGCGGAGAAGAAGGGCAGGAA
GTCCGCCCACGCCGCTGCTGACAAGTACGACGTGAACAAGGCCTCGCCCAAGCTTCAGCGCGAGTGGACG
CGCCGCCGCCGCAACCACAGGGTCGAGAAGGCGATCGCCGATCAGTTGCGCGAGGGTCGCGTGCTTGCGC
GCATAACGAGCCGCCCGGGCCAGTCCGGCCGTGCCGACGGTATCCTGCTGGAGGGCGCCGAGCTCCAGTT
CTACCTGAAGCGCCTGGAGAAGAAGAAGAAGTA
>X64137 1, 1905 hsp70.1
ATGTTTGCTCGTCGTGTGTGCGGAAGCGCTGCGGCGTCGGCTGCGTGCCTGGCGCGCCACGAGTCGCAGA
AGGTGCAGGGCGACGTGATTGGCGTGGACCTGGGCACGACGTACAGCTGCGTGGCGACGATGGACGGCGA
CAAGGCGCGCGTGCTGGAGAACTCGGAGGGCTTCCGGACGACGCCGTCTGTTGTGGCGTTCAAGGGCAGC
GAGAAGCTTGTGGGGCTTGCGGCGAAGCGGCAGGCGATCACGAACCCGCAGTCGACGTTCTATGCTGTGA
AGCGGCTGATCGGGCGCCGGTTCGAGGACGAGCACATCCAGAAGGACATCAAGAACGTGCCGTACAAGAT
CGTGCGCGCGGGGAACGGTGACGCGTGGGTGCAGGACGGGAACGGGAAGCAGTACTCGCCGTCGCAGATC
GGCGCGTTCGTGCTGGAGAAGATGAAGGAGACGGCGGAGAACTTCCTGGGGCACAAGGTGAGCAACGCCG
TCGTGACGTGCCCGGCGTACTTCAACGACGCGCAGCGCCAGGCGACGAAGGACGCGGGGACGATCGCGGG
CCTGAACGTGATCCGCGTGGTCAACGAGCCGACTGCTGCGGCTCTTGCGTACGGCATGGACAAGACGAAG
GACAGCCTGATCGCGGTGTACGACCTCGGTGGCGGCACGTTCGATATCTCCGTGCTGGAGATCGCTGGCG
GCGTGTTCGAGGTGAAGGCGACGAACGGCGACACGCACCTTGGCGGCGAGGACTTTGACCTGGCGCTGTC
GGACTACATCCTGGAGGAGTTCCGCAAGACGAGCGGGATCGACCTGAGCAAGGAGCGGATGGCGCTGCAG
CGCGTGCGCGAGGCCGCGGAGAAGGCGAAGTGCGAGCTGTCGTCTGCGATGGAGACGGAGGTGAACCTGC
CGTTCATCACTGCGAACGCCGACGGCGCGCAGCACATCCAGATGCGCATCAGCCGTAGCAAGTTCGAGGG

CATCACGCAGCGGCTGATCGAGCGGTCGATTGCGCCGTGCAAGCAGTGCATGAAGGACGCTGGTGTGGAG
CTGAAGGAGATCAACGACGTTGTGCTTGTTGGCGGCATGACGCGCATCCGAAGTGGTGGAGGAGGTGAAG
AAGTTCTTCCAGAAGGACCCGTCCGCGGCGTGAACCCCGACGAGGCTGTGGCGCTTGGTGCCGCGACGCT
GGGCGGCGTGCTGCGGGGTAAGGCGAGTGACTTGATACTGGTGGACGTGACACCGCTTTCGCTGGGCACA
AGTGTCGTCGGCGACGTGTTTGTGCCCATTATCCCGAAGAACACGACGATCCCGTGCATGCGCAGCCATA
TCTTCACAACGGTGGACGATGGTCAGACAGCCATCAAATTCAAGGTGTTCATCCCGCCCGCGCCGCGTGG
GGTGCCGCAGGTTGAGGTGACGTTCGACATCGACGCGAACCAGGGCGAGCGCGAAATCGCCTCCGAAAAC
CAGATAAGGGGTGAGTTCGATCTTAGCGGCGGCATCTGCCACGTGACGGCGAAGGACAAGGCGACGGGCA
AGACGCAGAACATCACGATCACGGCGAACGGCGGGCTGTCGAAGGAGCAGATCGAGCAGATGATCCGCGA
CTCGGAGCAGCACGCGGAGGCCGACCGCGTGAAGCGCGAGCTTGTGGAGGTGCGCAACAACGCGGAGACG
CAGCTGACAACGGCGGAGAGGCAGCTCGGCGAGTGGAAGTACGTGAGCGATGCGGAGAAGGAGAACGTGA
AGACGCTGGTGGCGGAGCTGCGCAAGGCGATGGAGAACCCGAACGTGGCGAAGGATGACCTTGCGGCTGC
GACGGACAAGCTGCAGAAGGCTGTGATGGAGTGCGGCCGCACAGAGTACCAGCAGGCTGCCGCGGCCAAT
TCTGGCCAGTGTTGA
>X69825 1, 1896 hsp70.4
ATGTCGTCTACCAACGCCATCGCGATCGACCTGGGCACGACGTACTCGTGCGTCGGTGTCTTCAAGAACG
AGCAGGTGGACATCATTGCCAACGACCAGGGTAACCGCACAACGCCGTCTTATGTGGCCTTCACCGAGAC
AGAGCGCCTGAATCGGCGATGCGCGAAGAACCAGGTGGCCATGAACCCGAGCAACACCGTGTTTGACGCG
AAGCGCATGATCGGCCGCAAGTTCGACGATCCTGACCTTCAGTCGGACATGAAGCACTGGCCCTTCAAGG
TGATCGTCAAGGACGGCAAGCCGGTCATCAGCGTCGAGTACCAGAACCAGACCAAGACCTTTTTCCCAGA
GGAGATCTCGGCTACGGTGCTGCAGAAAATGAAGGAGACGGCGGAGGCCTACCTCGGCACGACCGTCAAG
GACGCCGTCATCACCGTCCCGGCGTACTTCAACGACTCGCAGCGCCAGGCAACGAAGGACGCCGGCTCCA
TCGCTGGCCTCAACGTGCTGCGCATCATCAACGAGCCCACCGCCGCCGCTATCGCGTACGGCATGGACCG
CAAGGGAGACAAGGGGGAGAAGAATGTGCTCATCTTCGACCTTGGCGGCGGCACGTTCGATGTGACGCTG
CTGACGATCGAGTCCGGCGTGTTCGAGGTAAAGGCAACCGCGGGTGACACGCACCTTGGCGGCGAGGACT
TCGACAACCGTCTCGTCGACTACTCGCCACTGAGCTCAAGATGCGCTGCGGCAAGGACTGCCGTGGCAAC
GCCCGCGCCACGCGCCGGTCTGCGCACGGCGTGCGAGCGCGTGAAGCGCACGCTGTCCAGCTCCACGACG
GCAAACATCGAGATTGACGCGCTGTACGAGGGCAATGACTTCTTCTCGAAGATCACGCGCGCGCGCTTCG
AGGAGATGTGCCGCGATCAGTTTGAGAAGTGCCTCGAGCCGGTGAAAAAGGTGCTGGCAGATGCGGATAT
GAAGCCGCAGGACGTGGACGACGTCGTCCTCGTCGGCGGATCGACCCGAATCCCAAAGATCCAGCAGATT
GTCTCGCAGTTCTTCGGCGGCAAGGAGCTGAACCGCTCCATCAACCCCGATGAGGCCGTTGCGTACGGTG
CGGCGGTGCAGGCGCACATTCTCGCCGGCGGTCATTCCTCTAAGACTGACGGGCTGCTGCTGCTTGACGT
GACGCCGCTCTCGCTCGGTGTGGAGACATCCGGCGGTGTCATGTCCGTGCTCATCCCGCGCAACTCGACG
ATGCCGGTGCAAAAGACGCAGACATACTCGAACAACGCCGATAATCAGCGTAACGTGGTGATCAAGGTGT
ACGAAGGCGAGCGTCCGCTGGTTTCGCAGTGTCAGTGCCTCGGCACCTTCACGCTGACCGACATCCCACC
CATGCCGCGCGGCAAGGCGCGCATCAACGTCACGTTCGACGTGAACACGGACGGCATCCTCATCGTGAGC
GCCGTCGAGGAGTCGGGTGGTCGAAAGGAGGCCATCACGATCCAGAACGACACGGGCCGTCTGAGCAAGG
AGCAGATCGAGAGCATGGTGCGGGAGGCCGAAAAGTTTGCTGAGGAGGACAGGATGAACAGCGAGCGCGT
CGAGGCACGCAACACGCTGGAAAACTACACCTTCTCCATGCGCGCCACCCTAGACGACCCGGATGTGCAG
AATGGAATCACGCAGGGGGACCGCCAGCAAATCCAGGACGCCGTCAACGCTGCCTCGAGCTGGCTTGAGA
AAAACCGGGAGGCCACCAAGGAGGAGTACATGGAGCAGACGAAGCTGATCGAGGGCATCGCGCACCGATT
TTGTCGGAGTTCTACAAAAGCGTGTCATGGAGGCCCCACCAAGCGCTGGGCCGAAGGACGGTGCCCCCTC
AGATGA
>X73119 235, 621 ubiquitin-fusion protein
ATGCAGATCTTCGTGAAGACGCTGACTGGCAAGACGATCGCGCTGGAGGTGGAGCCGAGCGACACGATCG

AAAACGTGAAGGCCAAGATCCAGGACAAGGAGGGCATCCCGCCGGACCAGCAGCGCCTGATCTTCGCTGG
CAAGCAGCTGGAGGAGGGCCGCACTCTCTCGGACTACAACATCCAGAAGGAGTCCACGCTGCACCTGGTG
CTGCGCCTGCGCGGTGGCGTGATGGAGCCGACGCTGGTCGCGCTGGCCAAGAAGTACAACTGGGAGAAGA
AGGTGTGCCGCCGCTGCTACGCCCGCCTGCCGGTGCGCGCCACGAACTGCCGCAAGAAGGCCTGCGGTCA
CTGCTCCAACCTGCGCATGAAGAAGAAGCTGCGCTAG
>X73120 847, 1080 ubiC
ATGCAGATCTTCGTGAAGACGCTGACCGGCAAGACGATCGCGCTGGAGGTGGAGCCGAGCGACACGATCG
AGAACGTGAAGGCCAAGATCCAGGACAAGGAGGGCATCCCGCCGGACCAGCAGCGCCTGATCTTCGCGGG
CAAGCAGCTGGAGGAGGGCCGCACGCTCTCGGACTACAACATCCAGAAGGAGTCCACGCTGCACCTGGTG
CTGCGCCTGCGCGGCGGCCGTTAG
>X93566 120, 1451 beta-tubulin
ATGCGTGAGATCGTTTCCTGCCAGGCCGGCCAGTGCGGCAACCAGATCGGCTCTAAGTTTTGGGAGGTGA
TTGCCGACGAACATGGTGTCGATCCGACTGGCTCCTACCAGGGTGACTCGGATCTGCAGCTGGAGCGCAT
CAACGTGTACTTCGATGAGTCGGCGGGAGGCCGCTACGTGCCGCGCGCCGTGCTGATGGACCTCGAGCCC
GGCACTATGGACTCCGTTCGCGCCGGCCCGTACGGCCAGCTGTTCCGCCCGGACAACTTCATCTTTGGTC
AGTCCGGCGCTGGCAACAACTGGGCCAAGGGCCACTACACTGAGGGTGCGGAGCTGATCGACTCCGTGCT
TGATGTGTGCCGCAAGGAGGCGGAGAGCTGCGACTGCCTGCAGGGCTTCCAGCTGTCTCACTCCCTCGGC
GGCGGCACGGGCTCCGGCATGGGCACGCTGCTCATTTCCAAGCTGCGCGAGGAGTACCCGGACCGGATCA
TGATGACCTTCTCCGTCATCCCGTCCCCCCGCGTGTCGGATACCGTTGTGGAGCCGTACAACACGACCCT
CTCTGTGCACCAGCTCGTGGAGAACTCCGACGAGTCCATGTGCATCGACAACGAGGCGCTGTACGATATT
TGCTTCCGCACGCTGAAGCTGACGACGCCGACGTTCGGTGACCTGAACCACCTCGTCGCCGCTGTGATGT
CTGGCGTGACCTGCTGCCTGCGCTTCCCTGGCCAGCTGAACTCTGACCTGCGCAAGCTTGCCGTGAACCT
CGTGCCGTTCCCGCGCCTGCACTTCTTCATGATGGGCTTCGCGCCGCTGACGAGCCGCGGCTCGCAGGAG
TACCGCGGTCTGTCCGTCGCGGAGCTGACGCAGCAGATGTTCGACGCCAAGAACATGATGCAGGCCGCCG
ACCCGCGCCACGGCCGCTACCTCACCGCGTCCGCGCTGTTCCGCGGCCGCATGTCGACCAAGGAGGTGGA
CGAGCAGATGCTGAACGTGCAGAACAAGAACTCCAGCTACTTCATCGAGTGGATCCCGAACAACATCAAG
TCGTCCATCTGCGATATCCCGCCCAAGGGTCTCAAGATGTCTGTCACCTTCATCGGCAACAACACCTGCA
TCCAGGAGATGTTCCGCCGGGTCGGTGAGCAGCTCACGGGTATGTTCCGCCGCAAGGCCTTCCTCCACTG
GTACACCGGTGAGGGCATGGACGAGATGGAGTTCACGGAGGCCGAGTCCAACATGAACGACCTCGTCTCT
GAGTACCAGCAGTACCAGGACGCCACCGTCGAGGAGGAGGGCGAGTACGACGAGGAGGAGGAGGCCTACT
AG
>X93567 73, 1404 L.major mRNA for beta-tubulin (1404bp)
ATGCGTGAGATCGTTTCCTGCCAGGCCGGCCAGTGCGGCAACCAGATCGGCTCCAAGTTTTGGGAGGTGA
TTTCGGACGAACATGGTGTCGACCCGACTGGCACCTACCAGGGCGACTCGGATCTGCAGCTGGAGCGCAT
CAACGTCTACTTCGATGAGTCGACGGGAGGCCGCTACGTGCCGCGCGCCGTGCTGATGGACCTCGAGCCC
GGCACTATGGACTCCGTTCGCGCCGGCCCGTACGGCCAGCTGTTCCGCCCGGACAACTTCATCTTTGGTC
AGTCCGGCGCTGGCAACAACTGGGCCAAGGGCCACTACACTGAGGGCGCGGAGCTGATCGACTCCGTGCT
TGATGTGTGCCGCAAGGAGGCGGAGAGCTGCGACTGCCTGCAGGGCTTCCAGCTGTCTCACTCCCTCGGC
GGCGGCACGGGCTCCGGCATGGGCACGCTGCTCATTTCCAAGCTGCGCGAGGAGTACCCGGACCGGATCA
TGATGACCTTCTCCGTCATCCCGTCCCCCCGCGTGTCGGATACCGTTGTGGAGCCGTACAACACGACCCT
CTCTGTGCACCAGCTCGTGGAGAACTCCGACGAGTCCATGTGCATCGACAACGAGGCGCTGTACGATATT
TGCTTCCGCACGCTGAAGCTGACGACGCCGACGTTCGGTGACCTGAACCACCTCGTCGCCGCTGTGATGT
CTGGCGTGACCTGCTGCCTGCGCTTCCCTGGCCAGCTGAACTCTGACCTGCGCAAGCTTGCCGTGAACCT
CGTGCCGCTCCCGCGCCTGCACTTCTTCATGATGGGCTTCGCGCCGCTGACGAGCCGCGGCTCGCAGCAG
TACCGCGGTCTGTCCGTCGCGGAGCTGACGCAGCAGATGTTCGACGCCAAGAACATGATGCAGGCCGCCG

ACCCGCGCCACGGCCGCTACCTCACCGCGTCCGCGCTGTTCCGCGGCCGCATGTCGACCAGGGAGGTGGA
CGAGCAGATGCTGAACGTGCAGAACAAGAACTCCAGTTACTTCATCGAGTGGCTCCCGAACAACATCAGG
TCGTCCATCTGCGATATCCCGCCCAAGGGTCTCAAGATGTCCGCCACCTCCATCGGAAACAACACCTGCA
TCCAGGAGATGTTCCGCCGGGTCGGTGAGCAGCGCACGGGTATGTTCCGACGGAAGGCCTTCCTGCACTG
GTACACCGGTGAGGGCATGGACGAGATGGAGTTCACGGAGGCCGAGTCCAACATGAACGACCTCGTCTCT
GAGTACCAGCAGTACCAGGACGCCACCGTCGAGGAGGAGGGCGAGTACGACGAGGAGGAGGAGGCCTACT
AG
>X97072 246, 785 ARL3
ATGGGCCTTCTCACACTCCTTCGAAAGCTCAAAAAGAGCGAAACAGAGCCACGCATGCTGATTCTGGGCC
TCGATAATGCTGGCAAAACTTCCATTCTCCGCAAGCTGTCCGACGAGGACCCTACCACCACCCAGGCGAC
TCAGGGATTCAACATCAAATCCATCAACTGTGAGGGCTTTAAGTTGAACATGTGGGATATTGGTGGCCAG
AAGGCGATCCGCACGTACTGGCCAAACTACTACGATGAAGTGGATTGCCTGATTTACGTTGTCGATGCTG
CCGACCGGCGCCGCCTCGAGGAGACTTCCGCGGAGCTTGACACCCTTCTGCAGGAAGAAAAGCTCCGTCA
GGCCCCTGTACTTATTTTCTCCAACAAGTGCGACCTCGCCACTGCACTGTCCCCGGAAAATGTAAGCACA
GCACTCAATTTGCACAGCTTGCGCGATCGCACGTGGTCTATCCAGAAGTGCAGCGCGAAAACCGGTGAGG
GCCTGCATGAAGGTCTGGAGTGGGCGGTGAAGAACCTTAAGCCCGCGTGA
>Y00647 199, 2007 mature gp63 protein (AA 61-563)
ATGTCCGTGGACAGCAGCAGCACGCACCGGCGCCGCTGCGTCGCCGCGCGCCTGGTGCGCCTCGCGGCTG
CCGGCGCCGCAGTCACCGTTGCTGTCGGCACCGCGGCCGCGTGGGCACACGCCGGTGCGCTGCAGCACCG
CTGCGTCCACGACGCGATGCAGGCACGCGTGCGGCAGTCGGTGGCGGACCACCACAAGGCCCCCGGCGCG
GTGTCCGCGGTGGGTCTGCCGTACGTTACTCTCGACGCCGCGCACACCGCGGCCGCCGCCGATCCCAGGC
CGGGCAGCGCGCGCAGCGTCGTGCGCGACGTGAACTGGGGCGCGCTGCGCATCGCCGTCTCCACCGAGGA
CCTCACCGACCCCGCCTACCACTGCGCTCGCGTCGGGCAGCATGTCAAAGACCACGCCGGCGCCATCGTC
ACCTGCACCGCCGAGGACATCCTCACCAACGAGAAGCGCGACATCCTGGTCAAGCACCTCATCCCGCAGG
CGGTGCAGCTGCACACGGAGCGGCTGAAGGTGCAGCAGGTGCAGGGCAAGTGGAAGGTGACGGACATGGT
CGGCGATATCTGTGGCGACTTCAAGGTGCCGCAGGCGCACATCACCGAGGGCTTCAGCAACACCGACTTC
GTGATGTACGTCGCCTCCGTGCCGAGTGAGGAGGGTGTGCTGGCGTGGGCCACGACCTGCCAGACGTTCT
CTGACGGCCATCCAGCCGTGGGCGTCATCAACATCCCCGCGGCGAACATTGCGTCGCGGTACGACCAGCT
CGTCACGCGTGTCGTCACGCACGAGATGGCGCACGCGCTCGGCTTCAGCGGCCCATTCTTCGAGGACGCC
CGCATCGTGGCGAACGTTCCGAACGTTCGAGGCAAGAACTTCGATGTTCCCGTGATCAACAGCAGCACGG
CAGTGGCGAAGGCGCGCGAGCAGTACGGCTGCGACACCTTGGAGTATCTGGAGGTGGAGGACCAGGGCGG
TGCGGGCTCCGCCGGGTCGCACATCAAGATGCGCAACGCGCAGGACGAGCTCATGGCGCCTGCTGCAGCT
GCCGGGTACTACACCGCCCTGACCATGGCCATCTTCCAGGACCTCGGCTTCTACCAGGCGGACTTCAGCA
AGGCCGAGGTGATGCCGTGGGGCCAGAACGCCGGCTGCGCCTTCCTCACCAACAAGTGCATGGAGCAGAG
CGTCACGCAGTGGCCGGCGATGTTCTGCAATGAGAGCGAGGACGCCATCCGCTGCCCCACCAGTCGTCTC
AGCCTCGGTGCATGCGGTGTTACCCGTCACCCGGGCCTTCCGCCGTACTGGCAGTACTTCACGGACCCGT
CCCTCGCCGGCGTCTCCGCCTTCATGGACTACTGCCCTGTCGTGGTGCCCTACAGTGATGGCAGCTGCAC
GCAGCGTGCCTCTGAGGCACATGCTTCGTTGCTGCCCTTCAACGTCTTCTCTGACGCGGCGCGCTGCATC
GATGGTGCCTTCAGACCGAAGGCAACTGACGGCATAGTCAAGTCGTACGCCGGCCTGTGCGCCAACGTGC
AGTGTGACACGGCCACACGCACGTACAGCGTGCAGGTGCACGGCAGTAACGACTACACCAACTGCACGCC
GGGCCTCAGAGTTGAGCTGAGCACCGTGAGCAACGCCTTCGAGGGGGGCGGCTACATCACGTGCCCGCCG
TACGTGGAGGTGTGCCAGGGCAACGTGCAGGCTGCCAAGGACGGCGGCAACACGGCGGCTGGTCGTCGTG
GTCCGCGCGCCGCGGCGACGGCGCTGCTGGTGGCCGCGCTGCTGGCCGTGGCGCTCTAG
>Y13576 128, 661 cyclophilin
ATGCCTTACACGCCGCACTACCCTGTCGTTGAATCCAACCCCAAGGTTTGGATGGATATCGACATCGGTG

GCAAGCCCGCCGGCCGCGTAACGATGGAGCTCTTCAAGGACGCCGTTCCCCAGACGGCCGAGAACTTCCG
CGCGCTGTGCACGGGCGAGAAGGGCTTCGGCTACGCCAACTCCCCGTTCCACCGTGTGATCCCGGATTTC
ATGTGCCAGGGTGGTGACTTCACCAACGGCAACGGCACTGGCGGCAAGTCCATCTACGGCTCCAAGTTTG
CCGATGAGTCCTTTCTCGGCAAGGCCGGCAAGCACTTCGGCCCAGGCACGCTGTCGATGGCCAATGCCGG
CCCCAACACGAACGGCTCTCAGTTCTTCCTGTGCACAGCGCCCACGAGCTGGCTGGACGGCAAGCACGTC
GTGTTCGGCCAGGTGCTGGAGGGCTATGAAGTGGTCAAGGCTATGGAGGCCGTCGGCAGCCGCAGCGGCA
CCACCTCGAAGCCCGTGCGCGTGTCTGCCTGCGGGCAGCTTTAA
>Z14232_0 511, 897 Ubiquitin
ATGCAGATCTTCGTGAAGACGCTGACCGGCAAGACGATCGCGCTGGAGGTGGAGCCGAGCGACACGATCG
AGAATGTGAAGGCCAAGATCCAGGACAAGGAGGGCATCCCGCCGGACCAGCAGCGCCTGATCTTCGCCGG
CAAGCAGCTGGAGGAGGGCCGCACGCTCTCGGACTACAACATCCAGAAGGAGTCCACGCTGCACCTGGTG
CTGCGCCTGCGCGGCGGC
>Z14232_1 739, 897 ubiquitin tail protein
GTGATGGAGCCGACGCTGGTCGCGCTGGCCAAGAAGTACAACTGGGAGAAGAAGGTGTGCCGCCGCTGCT
ACGCCCGCCTGCCGGTGCGCGCCACGAACTGCCGCAAGAAGGCCTGCGGTCACTGCTCCAACCTGCGCAT
GAAGAAGAAGCTGCGCTAA
>Z28408 71, 385 L30-like ribosomal protein
ATGGCCAAGAAGACGAAGTCTAAGGTGAACACGATCAACGCCAAGCTCCAGCTGGTGATGAAGTCCGGTA
AGTACGTGCTCGGAACGCAGCAGGCGCTGACGACCCTCCGTCAGGGCCGCAGCAAGCTGGTTGTGATCGC
CAACAACTGCCCGCCGATCCGCCGCGCCGAGGTGGAGTACTACTGCACCCTCAGCAAGACCCCCATCCAC
CACTACTCTGGCAACAACCTGGACCTCGGTACGGCGTGCGGCAAGCCATTCCGCACATGCGTCCTGTCCG
TGACAAATGTTGGGGACTCCGACATCGCTACCTAA
>Z38058 2172, 4775 100 kDa heat shock protein (Hsp100)
ATGACGACGCAGCAGCCAGAATGGAAGCAGGCGGCGTCGGACTTGATGGCTCGCATGGCGGCACTGGCAC
GCAAGAAGGCAAACGGCTACCTCGACCCCGTGCATCTCGCCTACGTCATGTTTGAAGATGAAAACAGTCT
TGCTTCCCGTGCCGTGCGCAAACTCGGTGCCGCCTCAGTCAAGGACGGACTGGAGGCGCGTGTCGACGCG
ATTCCCAACCAGATGCCTGCGCCCACGCAGCCGCGGCCCAACTCGGACATGATGCGTGTCATGAACACGG
CGGAGCAGGAGCGTGCTGCCCTCGGTGACACCCTCATGGCCGCTGACCACTTCCTCCTGGCCCTGCACGA
GAGCAAGGAGGTTGGAAGAATCCTGGATGCTGCAGGGGCTGGAAAGAAGGCGATTCGCGCCACGCTGCTC
GAGATGCGCAAGGGGAAAAAGATAACCTCCGACTTCCAAGACGACAACTACGAGTCGCTGAACAAGTACG
CAGTTGATCTCTGCAAGCAGGCGGAGGACGGAAAGCTGGACCCGGTTATTGGTCGCGCAGACGAGATTCT
GCGCACTATCCGCGTGCTGTCACGCCGCACCAAGAACAACCCGGTGCTTATCGGGGAGCCTGGAGTGGGT
AAGACCGCGATTGTGGAGGGCATTGCGCAGCAGGTGGTGCGAGGCGACGTGCCGGACACCCTCTCCGGTA
TTCGCATCTTCTCACTGGACATGGGCGCGCTGATCGCCGGTGCCAAGTACCGCGGCGAGTTCGAGGAGCG
CCTGAAGGCCGTGCTGAATGAGGTGAAGGAAAGCGACAATAAGATCATTCTCTTTATTGTCGAGATCCAC
CTCGTGCTCGGCGCCGGCAAGTCCGACGGCGCGATGGACGCCGCGAATCTGCTGAAGCCGCTGCTGGCCC
GTGGCGACGTGCGCACGATCGGTGCCACAACGCTCGAGGAGTACCGCCAGTACGTGGAGAAGGACGCCGC
CTTCGAGCGCCGCTTCATGCCAGTGCAGGTGCATGAGCCGTCAGTCGAGGAGTGCACGAGCATCCTGCGT
GGTCTGAAAGACCGCTACGAGCAGCACCACGGTGTGCAGATCACGGACAAGGCCGTTGTCGTGGCGGCGC
AGTTGGCTGGCCGCTACATTACGAACCGCTTCCTGCCGGATAAGGCGATTGACCTCATTGATGAAGCGTG
CGCCAACGTGCGCGTGACGCTGTCGTCGCGGCCGGCCGAAATCGACGCCCTCGAGCGCAAGAAGCGCCAG
CTGGAGATCGAGGAGAGGGCACTGCAGCGCGACAAGGACGCATCGGCCAAGGAGCGGCTGAAAGCCGTGA
AGGCGGAGATTCAGAAGGTGGAGGAGAAACTTGGCCCTCTACTTGCCAAGTACGAACAGGAGCGTGGCCG
TATTGACGAGCTGCAGGCAACGCAGGCAAAGCTGGACGAGAAGAAGGTGAAGCTGGAGCGGGCAGAGCGG
ATGCGCGACATGGAGACGGCAGCAGACCTCAAATACCGCGTCATCCCAATCCTCCAGGACAGGATCCGAT

CCCTCAAGGAGGCGATCGAGAAGCAGAAGGCGACGATGCTGCAGGGCACCGTGACCGGGACGGACATCGC
CACCGTTGTGTCGCGCTGGACCAATATCCCGGTGACAAAGCTGAGCCAGACCGAACGCGAGCGCCTGTTG
CACCTGGCCGACCAGCTGCACCTCCGTGTGAAGGGCCAGGATGAGGCGGTGAGCCGTGTCGCGGAGGCCA
TTCTGCGCTCACGTGCCGGCCTGGCCCGTTCGGACCGGCCCACCGGCTCTTTCCTGTTTCTCGGTCCCAC
CGGCGTGGGCAAGACAGAGCTATCCAAGGCCGTTGCCTCAGAGCTCTTCGACGACGCCAAGTACATGGTG
CGACTTGACATGAGCGAGTACATGGAGCAGCACTCTGTGGCGCGGCTGATCGGCACCCCGCCGGGGTACG
TCGGCCACGAAGAAGGTGGCCAGCTGACGGAGCCGGTGCGCCGTCGCCCATACACGGTGGTGCTGCTGGA
TGAAGTGGAAAAGGCGCACCCGAACGTCTTCAACGTACTGCTGCAGGTGCTCGACGACGGGCGGTTGACT
GACTCGCACGGCCGAACCGTGGATTTCTGCAACACGATCATCATCATGACATCCAACTTGGGTGCGCAGT
ATCTGCAGAACATGGACACCTCACCGAAGCCGTACGAAGTGGCACAGGCACAGGTGATGGGCGAGGTAAG
GAAGTTCTTTCGCCCGGAGTTCATCAACCGACTGGACGACATCATCCTCTTCCGCTCTCTGGGCTTAAAG
GAGATGACCGGCATCATCGACCTCATCACAGAGGAGCTCAATGGCCGCCTCAAGGACCAGTCCATCCGGG
TCTCCCTCACCGAAGAGGCCAAGCAGTACGTATTGGAGTCCGCCTTCGACGCGGATATGGGCGCTCGCCC
GCTACGGCGCTGGGTTGAGAAGAACATTACAACAGAGCTCAGCCGCATGATCATCTCGCAAGAGCTGTCG
CCGAACAGCACAGTAAAAGTGACTCTCAGCAGCAATCATAAAAAGCTCTCTTTCTCGGTGAAGCGGACGG
CGGCGCAGACATGA
>Z86100 1, 450 hydrophilic surface protein 1
GAGACCAACCAGGGAGGGAATGCGAGTGGCTCCCGGAAGAGCGCCGGCGGTCGGACCAACGAATATGACC
CGAAGGATGATGGCTTCACGCCGAACAACGAAGACTGTTGCCCGAAGGAGGATGGCCATACAGGGAAGAA
CGATGACGGTGGCCCGAAGGAGGATGGCCATGCGCCGAAGAACGATGACCATGCCCCGAAGGAGGATGGC
CATGCGCCGAAGAACGATGACCATGCCCCGAAGGAGGATGGCCATGCGCCGAAGAACGATGACCATGCCC
CGAAGGAGGATGGCCATGCGCCGAAGAACGATGACCATGCCCCGAAGGAGGATGGCCATGCGCCGAAGAA
CGATGACCATGCCCCGAAGGAGGATGGCCATGCGCCGAAGAACGATGACCATGCCCCGAAGGAGGATGGC
CATGCGCAGAAGAACGACGGGGATGTGCAG
>Z86101 1, 282 hydrophilic surface protein 1
GAGACCAACCAGGGAGGGAATGCGAGTGGCTCCCGGAAGAGCGCCGGCGGTCGGACCAACGAATATGACC
CGAAGGATGATGGCTTCACGCCGAACAACGAAGACCATTGCCCGAAGGAGGATGGCCATACAGGGAAGAA
CGACGACGGTGGCCCGAAGGAGGATGGCCATGCGCCGAAGAACGATGACCATGCCCCGAAGGAGGATGGC
CATGCGGCGAAGAACGATGACCATGCCCCGAAGGAGGATGGCCATGCGCAGAAGAACGACGGGGATGTGC
AG
>Z86102 1, 408 hydrophilic surface protein 1
GAGACCAACCAGGGAGGGAATGCGAGTGGCTCCCGGAAGAGCGCCGGCGGTCGGACCAACGAATATGACC
CGAAGGATGATGGCTTCACGCCGAACAACGAAGACCGTTGCCCGAAGGAGGATGGCCATACAGGGAAGAA
CGACGACGGTGGCCCGAAGGAGGATGGCCATGCGCCGAAGAACGATGACCATGCCCCGAAGGAGGATGGC
CATGCGCCGAAGAACGATGACCATGCCCCGAAGGAGGATGGCCATGCGCCGAAGAACGATGACCATGCCC
CGAAGGAGGATGGCCATGCGCCGAAGAACGATGACCATGCCCCGAAGGAGGATGGCCATGCGCCGAAGAA
CGATGACCATGCCCCGAAGGAGGATGGCCATGCGCCGAAGAACGACGGGGATGTGCAG
>gi|28565603:969-1541 Leishmania major ribosomal protein S5 gene
cluster, complete sequence
ATGAGCAGCAAGACTCCCAAGCTCTTCAACAAGTGGTCGTTCGAGGGCCTCGAGACGTCGGAGCTGGCCC
TCCGCGACCACATCTCGACGACGGCCGCGTATGTGCCGCACACGTCCGGCCGCTGGCAGAAGCGCCGCTT
CTACAAGGCCCGCATGCCGATTGTCGAGCGTCTGGCCAACGGCCTGATGTTCAAGGGCCGCGGCAACGGT
CGCAAGCTGCAGGCTGCGCGTCTGCTGAAGCACACACTGGAAATCATCCACCTGCTGACGGACGAGAACC
CCCTGCAGGTTGTTGTGGACGCCGTGTCGAAGGGGGCGCCGCGTGAGGACTCGACCCGCGTCGGCTCCGG
TGGTGTTGTGCGCCGCCAGGCTGTGGATGTGTCACCGATGCGCCGCGTGAATGAGGCGATCTACCAGATG

TGCAAGGGCGCGCGTGAGGCTGCCTTCCGCAACCTGAAGTCGATGCCGGAGTGCCTCGCGGACGAGATCG
TGAACGCGTCCAAGGGTAGCTCGAACTCCTACGCCATCAAGAAGAAGGATGAGGTGGAGCGCGTGGCCAA
GGCTAACCGGTAA
