# Supplementary material for: A computational investigation of kinetoplastid trans-splicing
Source: Genome Biol. 2005 Oct 17;6(11):R95. doi: 10.1186/gb-2005-6-11-r95 (PMC1297651; doi:10.1186/gb-2005-6-11-r95)
Supplement: Additional data File 4 — PDF file containing the full set of predictions for chromosomes 1 and 3 of Leishmania major [file gb-2005-6-11-r95-S4.pdf]

Complete Predictions for Chromosomes 1 and 3

The complete set of splice site predictions for chromosomes 1 and 3 are included below. For each splice site, we provide the following information:

```
Splice_346 253904 253905 1.797 Predicted splice site
```

Splice_346 is our internal identifier for a given splice site prediction, and the next two values rpresent the chromosomal positions (start and end) of the putative splice site. The fourth column includes the z-score, which as discussed in the main manuscript, is a measure of the likelihood that the predicted splice site is a functional site. The higher the z-score, the more likely the site is functional. We then include a short annotation of the splice site.

For open reading frames, our internal identifier is of the form `lmaj_01_084` where the last three digits indicate that this is the 84th ORF on chromosome 1 (indicated by `lmaj_01`). We include the annotation present in the public version of the genome sequence as well as the publicly reported start and end of the ORF. ORFs do not have a likelihood value assigned to them for obvious reasons.

Predictions for the Forward Strand of Chromosome 1

Splice_1 1403 1404 1.698 Predicted splice site
Splice_2 1957 1958 1.783 Predicted splice site
Splice_3 2159 2160 1.621 Predicted splice site
Splice_4 3020 3021 2.204 Predicted splice site
Splice_5 3715 3716 1.621 Predicted splice site
Splice_6 4448 4449 1.713 Predicted splice site
Splice_7 5301 5302 1.554 Predicted splice site
Splice_8 9263 9264 1.668 Predicted splice site
Splice_9 9410 9411 1.520 Predicted splice site
Splice_10 14061 14062 2.073 Predicted splice site
Splice_11 17610 17611 1.797 Predicted splice site
Splice_12 17703 17704 1.668 Predicted splice site
Splice_13 18051 18052 2.395 Predicted splice site
Splice_14 18644 18645 1.769 Predicted splice site
Splice_15 18933 18934 1.605 Predicted splice site
Splice_16 20163 20164 1.769 Predicted splice site
Splice_17 22934 22935 1.501 Predicted splice site
Splice_18 23855 23856 1.797 Predicted splice site
Splice_19 24187 24188 1.572 Predicted splice site
Splice_20 24284 24285 1.537 Predicted splice site
Splice_21 25975 25976 1.728 Predicted splice site
Splice_22 26122 26123 1.537 Predicted splice site
Splice_23 26405 26406 1.945 Predicted splice site
Splice_24 28010 28011 1.605 Predicted splice site
Splice_25 29756 29757 1.849 Predicted splice site
Splice_26 33534 33535 1.910 Predicted splice site
Splice_27 35595 35596 1.637 Predicted splice site
Splice_28 35699 35700 2.150 Predicted splice site
Splice_29 36898 36899 2.450 Predicted splice site
Splice_30 39673 39674 1.742 Predicted splice site
Splice_31 40398 40399 2.350 Predicted splice site
Splice_32 41054 41055 1.698 Predicted splice site
Splice_33 41114 41115 1.588 Predicted splice site
Splice_34 42925 42926 1.637 Predicted splice site
Splice_35 43300 43301 1.554 Predicted splice site
Splice_36 43801 43802 1.621 Predicted splice site
Splice_37 47567 47568 2.064 Predicted splice site
Splice_38 48633 48634 1.637 Predicted splice site
Splice_39 52578 52579 1.698 Predicted splice site
Splice_40 52846 52847 1.809 Predicted splice site
Splice_41 53242 53243 1.957 Predicted splice site
Splice_42 53481 53482 1.554 Predicted splice site
Splice_43 53553 53554 1.520 Predicted splice site

Splice_44 53981 53982 1.835 Predicted splice site
Splice_45 54060 54061 1.588 Predicted splice site
Splice_46 54407 54408 2.033 Predicted splice site
Splice_47 54585 54586 1.769 Predicted splice site
Splice_48 54735 54736 1.637 Predicted splice site
Splice_49 54839 54840 2.150 Predicted splice site
Splice_50 56185 56186 1.934 Predicted splice site
Splice_51 56276 56277 1.769 Predicted splice site
Splice_52 56963 56964 1.756 Predicted splice site
Splice_53 59873 59874 2.103 Predicted splice site
Splice_54 60173 60174 1.861 Predicted splice site
Splice_55 60854 60855 1.520 Predicted splice site
Splice_56 63980 63981 1.683 Predicted splice site
Splice_57 67225 67226 1.783 Predicted splice site
Splice_58 69108 69109 2.221 Predicted splice site
Splice_59 69620 69621 1.572 Predicted splice site
Splice_60 70266 70267 2.122 Predicted splice site
Splice_61 70991 70992 1.957 Predicted splice site
Splice_62 71226 71227 2.221 Predicted splice site
Splice_63 71551 71552 2.221 Predicted splice site
Splice_64 73859 73860 1.979 Predicted splice site
Splice_65 74412 74413 2.073 Predicted splice site
Splice_66 75627 75628 1.668 Predicted splice site
Splice_67 75894 75895 1.653 Predicted splice site
Splice_68 76162 76163 2.064 Predicted splice site
Splice_69 76629 76630 1.769 Predicted splice site
Splice_70 77599 77600 1.823 Predicted splice site
lmaj_01_032 79144 80067 0 poly(A) export protein,
putative
Splice_71 79316 79317 1.637 Predicted splice site
Splice_72 80301 80302 1.874 Predicted splice site
Splice_73 80469 80470 2.288 Predicted splice site
lmaj_01_033 80534 81163 0 hypothetical protein,
conserved
Splice_74 81253 81254 1.501 Predicted splice site
Splice_75 81312 81313 1.605 Predicted splice site
Splice_76 81545 81546 2.093 Predicted splice site
Splice_77 81635 81636 1.501 Predicted splice site
lmaj_01_034 81748 82041 0 hypothetical protein,
conserved
Splice_78 81967 81968 1.835 Predicted splice site
Splice_79 82099 82100 1.588 Predicted splice site
Splice_80 82498 82499 1.849 Predicted splice site
Splice_81 82804 82805 1.683 Predicted splice site
Splice_82 83140 83141 3.068 Predicted splice site

lmaj_01_035 83340 84116 0 hypothetical protein,
conserved
Splice_83 83644 83645 1.756 Predicted splice site
Splice_84 83968 83969 2.372 Predicted splice site
Splice_85 84098 84099 2.327 Predicted splice site
Splice_86 84232 84233 1.588 Predicted splice site
Splice_87 85072 85073 2.093 Predicted splice site
lmaj_01_036 85900 88104 0 hypothetical protein,
unknown function
Splice_88 86583 86584 1.605 Predicted splice site
Splice_89 88258 88259 1.637 Predicted splice site
Splice_90 88785 88786 2.001 Predicted splice site
Splice_91 88921 88922 2.409 Predicted splice site
lmaj_01_037 88973 89542 0 hypothetical protein,
unknown function
Splice_92 89124 89125 1.537 Predicted splice site
Splice_93 89397 89398 1.728 Predicted splice site
Splice_94 89704 89705 1.898 Predicted splice site
Splice_95 89798 89799 1.742 Predicted splice site
Splice_96 90030 90031 1.653 Predicted splice site
lmaj_01_038 90099 90665 0 hypothetical protein,
conserved
Splice_97 90490 90491 1.756 Predicted splice site
Splice_98 90745 90746 1.637 Predicted splice site
Splice_99 91269 91270 3.013 Predicted splice site
lmaj_01_039 91350 93806 0 hypothetical protein,
conserved
Splice_100 94022 94023 1.683 Predicted splice site
Splice_101 94366 94367 1.501 Predicted splice site
Splice_102 94597 94598 1.668 Predicted splice site
Splice_103 94713 94714 1.989 Predicted splice site
lmaj_01_040 94759 97227 0 hypothetical protein,
conserved
Splice_104 95717 95718 1.756 Predicted splice site
Splice_105 97836 97837 2.113 Predicted splice site
Splice_106 98157 98158 1.849 Predicted splice site
lmaj_01_041 98191 99870 0 hypothetical protein,
conserved
Splice_107 98689 98690 2.168 Predicted splice site
Splice_108 100012 100013 1.910 Predicted splice site
Splice_109 100102 100103 1.861 Predicted splice site
Splice_110 100496 100497 2.450 Predicted splice site
lmaj_01_042 100525 101127 0 ribosomal protein S7,
putative
Splice_111 101480 101481 2.589 Predicted splice site

Splice_112 101702 101703 2.239 Predicted splice site
Splice_113 102413 102414 3.100 Predicted splice site
lmaj_01_043 102441 103043 0 ribosomal protein S7,
putative
Splice_114 103202 103203 2.043 Predicted splice site
Splice_115 103633 103634 2.043 Predicted splice site
Splice_116 103781 103782 2.022 Predicted splice site
lmaj_01_044 103839 106070 0 hypothetical protein,
unknown function
Splice_117 106491 106492 1.823 Predicted splice site
Splice_118 106866 106867 2.288 Predicted splice site
Splice_119 107066 107067 2.746 Predicted splice site
Splice_120 107221 107222 1.537 Predicted splice site
lmaj_01_045 107435 109198 0 hypothetical protein,
unknown function
Splice_121 107735 107736 2.012 Predicted splice site
Splice_122 109569 109570 1.989 Predicted splice site
Splice_123 109843 109844 2.673 Predicted splice site
Splice_124 109951 109952 1.605 Predicted splice site
Splice_125 110414 110415 1.554 Predicted splice site
Splice_126 110971 110972 1.501 Predicted splice site
Splice_127 111836 111837 2.150 Predicted splice site
Splice_128 111980 111981 2.304 Predicted splice site
lmaj_01_046 112136 112792 0 alpha/beta-hydrolase-like
protein
Splice_129 112164 112165 2.358 Predicted splice site
Splice_130 112419 112420 1.756 Predicted splice site
Splice_131 112995 112996 1.520 Predicted splice site
Splice_132 113258 113259 1.742 Predicted splice site
Splice_133 113537 113538 2.612 Predicted splice site
Splice_134 113721 113722 2.204 Predicted splice site
Splice_135 113787 113788 1.713 Predicted splice site
Splice_136 114021 114022 2.715 Predicted splice site
Splice_137 114287 114288 2.509 Predicted splice site
lmaj_01_047 114900 119180 0 hypothetical protein,
unknown function
Splice_138 115087 115088 1.861 Predicted splice site
Splice_139 115348 115349 1.769 Predicted splice site
Splice_140 119520 119521 1.922 Predicted splice site
Splice_141 119628 119629 1.849 Predicted splice site
Splice_142 119790 119791 1.605 Predicted splice site
Splice_143 119911 119912 2.296 Predicted splice site
Splice_144 120267 120268 1.934 Predicted splice site
Splice_145 120486 120487 2.358 Predicted splice site
Splice_146 120566 120567 1.605 Predicted splice site

Splice_147 120650 120651 1.945 Predicted splice site
Splice_148 121414 121415 2.395 Predicted splice site
Splice_149 121575 121576 2.395 Predicted splice site
Splice_150 121725 121726 1.835 Predicted splice site
lmaj_01_048 121756 123846 0 fatty acyl CoA syntetase 1, putative
Splice_151 124285 124286 2.150 Predicted splice site
Splice_152 124466 124467 1.861 Predicted splice site
Splice_153 124538 124539 1.797 Predicted splice site
Splice_154 124603 124604 1.698 Predicted splice site
lmaj_01_049 124708 125787 0 hypothetical protein, conserved
Splice_155 124720 124721 2.187 Predicted splice site
Splice_156 125959 125960 2.230 Predicted splice site
Splice_157 126042 126043 1.588 Predicted splice site
Splice_158 126287 126288 2.204 Predicted splice site
Splice_159 126795 126796 1.797 Predicted splice site
Splice_160 126889 126890 1.537 Predicted splice site
Splice_161 127032 127033 1.501 Predicted splice site
Splice_162 127177 127178 2.437 Predicted splice site
Splice_163 127683 127684 2.033 Predicted splice site
lmaj_01_050 127701 129797 0 long chain fatty acid CoA ligase, putative
Splice_164 129962 129963 2.168 Predicted splice site
Splice_165 130228 130229 1.588 Predicted splice site
Splice_166 130616 130617 1.683 Predicted splice site
Splice_167 130842 130843 2.416 Predicted splice site
lmaj_01_051 130944 133067 0 fatty acyl CoA synthetase 2, putative
Splice_168 133210 133211 2.012 Predicted splice site
Splice_169 133360 133361 2.033 Predicted splice site
Splice_170 133446 133447 1.967 Predicted splice site
Splice_171 133691 133692 1.967 Predicted splice site
Splice_172 134355 134356 2.509 Predicted splice site
Splice_173 134430 134431 1.835 Predicted splice site
Splice_174 134640 134641 1.605 Predicted splice site
Splice_175 135105 135106 1.945 Predicted splice site
Splice_176 135470 135471 2.141 Predicted splice site
Splice_177 136007 136008 2.320 Predicted splice site
Splice_178 136191 136192 2.457 Predicted splice site
lmaj_01_052 136523 137107 0 fatty acyl CoA synthetase 2, putative
Splice_179 136986 136987 2.150 Predicted splice site
Splice_180 137207 137208 2.122 Predicted splice site
Splice_181 137510 137511 2.784 Predicted splice site

Splice_182 137576 137577 1.605 Predicted splice site
lmaj_01_053 137708 139855 0 long chain fatty acid
CoA ligase, putative
Splice_183 140167 140168 2.230 Predicted splice site
Splice_184 140431 140432 1.945 Predicted splice site
Splice_185 140605 140606 2.416 Predicted splice site
Splice_186 140728 140729 2.312 Predicted splice site
Splice_187 140786 140787 1.588 Predicted splice site
Splice_188 140890 140891 2.150 Predicted splice site
Splice_189 141036 141037 2.401 Predicted splice site
Splice_190 141284 141285 1.989 Predicted splice site
Splice_191 141417 141418 2.053 Predicted splice site
Splice_192 141809 141810 2.073 Predicted splice site
Splice_193 141870 141871 1.501 Predicted splice site
Splice_194 142831 142832 1.945 Predicted splice site
Splice_195 142998 142999 1.756 Predicted splice site
Splice_196 143149 143150 2.483 Predicted splice site
Splice_197 143330 143331 1.957 Predicted splice site
Splice_198 143423 143424 1.572 Predicted splice site
Splice_199 143633 143634 1.637 Predicted splice site
lmaj_01_054 143747 148570 0 long chain fatty acid
CoA ligase, putative
Splice_200 147531 147532 1.605 Predicted splice site
Splice_201 148767 148768 2.516 Predicted splice site
Splice_202 149308 149309 2.652 Predicted splice site
lmaj_01_055 149668 151647 0 hypothetical protein,
unknown function
Splice_203 151795 151796 1.797 Predicted splice site
Splice_204 151907 151908 2.221 Predicted splice site
Splice_205 152167 152168 2.033 Predicted splice site
Splice_206 152692 152693 2.740 Predicted splice site
lmaj_01_056 152784 153653 0 hypothetical protein,
unknown function
Splice_207 153215 153216 1.537 Predicted splice site
Splice_208 154841 154842 2.113 Predicted splice site
Splice_209 154918 154919 1.728 Predicted splice site
lmaj_01_057 154973 156835 0 hypothetical protein,
conserved
Splice_210 157070 157071 1.742 Predicted splice site
Splice_211 157326 157327 2.043 Predicted splice site
lmaj_01_058 157433 158413 0 tricarboxylate carrier,
putative
Splice_212 157763 157764 2.001 Predicted splice site
Splice_213 158769 158770 1.698 Predicted splice site
Splice_214 158968 158969 1.898 Predicted splice site

Splice_215 159100 159101 1.898 Predicted splice site
lmaj_01_059 159192 162101 0 hypothetical protein, conserved
Splice_216 159272 159273 1.520 Predicted splice site
Splice_217 161778 161779 1.742 Predicted splice site
Splice_218 162746 162747 1.621 Predicted splice site
Splice_219 162898 162899 2.187 Predicted splice site
Splice_220 163042 163043 2.380 Predicted splice site
Splice_221 163160 163161 1.713 Predicted splice site
lmaj_01_060 163204 164478 0 mitochondrial RNA editing ligase 1
Splice_222 163224 163225 1.653 Predicted splice site
Splice_223 163896 163897 1.537 Predicted splice site
Splice_224 165049 165050 1.849 Predicted splice site
Splice_225 165323 165324 1.885 Predicted splice site
Splice_226 165559 165560 1.683 Predicted splice site
lmaj_01_061 165642 170996 0 hypothetical protein, conserved
Splice_227 166342 166343 1.520 Predicted splice site
Splice_228 168236 168237 1.809 Predicted splice site
Splice_229 171435 171436 2.073 Predicted splice site
lmaj_01_062 171999 172730 0 DNA-damage inducible protein DDI1-like protein
Splice_230 172807 172808 1.713 Predicted splice site
Splice_231 173144 173145 2.893 Predicted splice site
Splice_232 173225 173226 1.910 Predicted splice site
lmaj_01_063 173480 175255 0 hypothetical protein, conserved
Splice_233 176522 176523 2.740 Predicted splice site
Splice_234 176651 176652 1.835 Predicted splice site
lmaj_01_064 177725 179602 0 hypothetical protein, unknown function
Splice_235 177981 177982 1.501 Predicted splice site
Splice_236 179424 179425 1.572 Predicted splice site
Splice_237 180186 180187 1.769 Predicted splice site
Splice_238 180515 180516 1.554 Predicted splice site
Splice_239 180897 180898 1.698 Predicted splice site
Splice_240 181000 181001 1.861 Predicted splice site
Splice_241 181061 181062 1.605 Predicted splice site
Splice_242 181125 181126 1.683 Predicted splice site
Splice_243 181278 181279 2.522 Predicted splice site
lmaj_01_065 181455 184793 0 HSP70-like protein
Splice_244 181674 181675 1.797 Predicted splice site
Splice_245 185061 185062 1.588 Predicted splice site
Splice_246 185595 185596 2.490 Predicted splice site

lmaj_01_066 185782 187266 0 mitochondrial processing peptide beta subunit,
Splice_247 187473 187474 2.483 Predicted splice site
Splice_248 187886 187887 1.967 Predicted splice site
Splice_249 188140 188141 2.471 Predicted splice site
Splice_250 188251 188252 2.103 Predicted splice site
lmaj_01_067 188565 193028 0 hypothetical protein, conserved
Splice_251 190394 190395 1.967 Predicted splice site
Splice_252 193601 193602 2.444 Predicted splice site
Splice_253 193707 193708 2.073 Predicted splice site
Splice_254 193818 193819 2.122 Predicted splice site
Splice_255 193927 193928 2.195 Predicted splice site
Splice_256 194036 194037 2.195 Predicted splice site
lmaj_01_068 194043 194960 0 hypothetical protein, unknown function
Splice_257 195149 195150 2.571 Predicted splice site
Splice_258 195557 195558 3.068 Predicted splice site
Splice_259 195628 195629 1.783 Predicted splice site
Splice_260 195790 195791 2.168 Predicted splice site
Splice_261 196038 196039 2.335 Predicted splice site
Splice_262 196360 196361 2.813 Predicted splice site
Splice_263 197131 197132 2.836 Predicted splice site
Splice_264 197597 197598 2.012 Predicted splice site
Splice_265 197773 197774 1.683 Predicted splice site
Splice_266 197843 197844 1.769 Predicted splice site
Splice_267 198089 198090 1.945 Predicted splice site
Splice_268 198189 198190 1.520 Predicted splice site
Splice_269 198601 198602 1.957 Predicted splice site
Splice_270 198738 198739 2.187 Predicted splice site
lmaj_01_069 198852 201254 0 hypothetical protein, unknown function
Splice_271 201626 201627 1.520 Predicted splice site
Splice_272 201831 201832 2.064 Predicted splice site
Splice_273 202691 202692 2.784 Predicted splice site
Splice_274 202767 202768 1.849 Predicted splice site
lmaj_01_070 202892 203584 0 hypothetical protein, unknown function
Splice_275 203279 203280 1.501 Predicted splice site
Splice_276 203487 203488 1.520 Predicted splice site
Splice_277 203886 203887 2.416 Predicted splice site
Splice_278 204144 204145 2.595 Predicted splice site
Splice_279 204354 204355 1.835 Predicted splice site
Splice_280 204488 204489 1.756 Predicted splice site
Splice_281 204589 204590 1.637 Predicted splice site

Splice_282 204707 204708 1.910 Predicted splice site
Splice_283 204771 204772 1.605 Predicted splice site
Splice_284 204879 204880 1.572 Predicted splice site
Splice_285 205524 205525 1.572 Predicted splice site
Splice_286 205614 205615 1.835 Predicted splice site
lmaj_01_071 206307 207071 0 hypothetical protein,
unknown function
Splice_287 206571 206572 1.713 Predicted splice site
Splice_288 206668 206669 1.668 Predicted splice site
Splice_289 207147 207148 1.537 Predicted splice site
Splice_290 207967 207968 1.698 Predicted splice site
Splice_291 208415 208416 1.728 Predicted splice site
lmaj_01_072 208462 210087 0 ubiquitin activating enzyme,
putative
Splice_292 210277 210278 2.064 Predicted splice site
Splice_293 210510 210511 2.509 Predicted splice site
lmaj_01_073 210682 215415 0 hypothetical protein,
conserved
Splice_294 211524 211525 1.572 Predicted splice site
Splice_295 212913 212914 1.698 Predicted splice site
Splice_296 213794 213795 1.621 Predicted splice site
Splice_297 215120 215121 1.874 Predicted splice site
Splice_298 215508 215509 1.934 Predicted splice site
Splice_299 215768 215769 2.247 Predicted splice site
Splice_300 216094 216095 2.450 Predicted splice site
Splice_301 216230 216231 1.520 Predicted splice site
lmaj_01_074 216577 223302 0 hypothetical protein,
conserved
Splice_302 216749 216750 1.572 Predicted splice site
Splice_303 218626 218627 1.520 Predicted splice site
Splice_304 223535 223536 1.797 Predicted splice site
Splice_305 223835 223836 1.728 Predicted splice site
Splice_306 223979 223980 2.187 Predicted splice site
lmaj_01_075 223996 226251 0 hypothetical protein,
conserved
Splice_307 226949 226950 2.471 Predicted splice site
Splice_308 227012 227013 1.668 Predicted splice site
lmaj_01_076 227137 231810 0 protein kinase, putative
Splice_309 227213 227214 1.967 Predicted splice site
Splice_310 227462 227463 2.720 Predicted splice site
Splice_311 232203 232204 1.668 Predicted splice site
Splice_312 232373 232374 2.304 Predicted splice site
lmaj_01_077 232416 233435 0 hypothetical protein,
unknown function
Splice_313 232618 232619 1.783 Predicted splice site

Splice_314 233987 233988 1.756 Predicted splice site
Splice_315 234279 234280 2.497 Predicted splice site
Splice_316 234366 234367 1.698 Predicted splice site
lmaj_01_078 234389 235600 0 eukaryotic initiation
factor 4a, putative
Splice_317 235684 235685 1.945 Predicted splice site
Splice_318 236093 236094 1.945 Predicted splice site
Splice_319 236322 236323 1.501 Predicted splice site
Splice_320 236555 236556 2.409 Predicted splice site
Splice_321 236623 236624 1.588 Predicted splice site
Splice_322 236704 236705 1.520 Predicted splice site
Splice_323 237276 237277 2.553 Predicted splice site
Splice_324 237363 237364 1.698 Predicted splice site
lmaj_01_079 237386 238597 0 eukaryotic initiation
factor 4a, putative
Splice_325 238681 238682 1.945 Predicted splice site
Splice_326 239252 239253 1.874 Predicted splice site
Splice_327 239448 239449 2.553 Predicted splice site
Splice_328 239817 239818 2.178 Predicted splice site
Splice_329 240488 240489 2.221 Predicted splice site
lmaj_01_080 240764 242350 0 hypothetical protein,
conserved
Splice_330 242537 242538 2.497 Predicted splice site
Splice_331 243077 243078 2.715 Predicted splice site
Splice_332 243244 243245 1.653 Predicted splice site
Splice_333 243661 243662 2.296 Predicted splice site
Splice_334 244498 244499 2.358 Predicted splice site
lmaj_01_081 244528 246057 0 hypothetical protein,
conserved
Splice_335 244645 244646 2.372 Predicted splice site
Splice_336 246882 246883 3.051 Predicted splice site
Splice_337 247078 247079 2.694 Predicted splice site
Splice_338 247345 247346 1.922 Predicted splice site
lmaj_01_082 247548 250601 0 calcium/potassium channel
(CAKC), putative
Splice_339 248052 248053 1.621 Predicted splice site
Splice_340 248167 248168 1.621 Predicted splice site
Splice_341 248341 248342 1.653 Predicted splice site
Splice_342 251556 251557 1.668 Predicted splice site
Splice_343 251614 251615 1.588 Predicted splice site
Splice_344 252112 252113 2.775 Predicted splice site
Splice_345 252369 252370 2.700 Predicted splice site
lmaj_01_083 252409 255888 0 potassium channel
subunit-like protein
Splice_346 253904 253905 1.797 Predicted splice site

Splice_347 255872 255873 1.885 Predicted splice site
Splice_348 256006 256007 2.064 Predicted splice site
Splice_349 256759 256760 2.178 Predicted splice site
Splice_350 256945 256946 1.728 Predicted splice site
Splice_351 257396 257397 2.001 Predicted splice site
Splice_352 258107 258108 1.537 Predicted splice site
Splice_353 258588 258589 1.756 Predicted splice site
Splice_354 258813 258814 1.621 Predicted splice site
lmaj_01_084 258829 260865 0 peptidyl dipeptidase,
putative
Splice_355 260074 260075 1.653 Predicted splice site
Splice_356 261454 261455 2.247 Predicted splice site
Splice_357 262609 262610 1.910 Predicted splice site
Splice_358 262677 262678 1.572 Predicted splice site
Splice_359 263259 263260 1.809 Predicted splice site
Splice_360 263921 263922 1.554 Predicted splice site
Splice_361 264454 264455 1.605 Predicted splice site
Splice_362 264848 264849 2.132 Predicted splice site
Splice_363 264911 264912 1.668 Predicted splice site
Splice_364 265093 265094 1.698 Predicted splice site
Splice_365 265329 265330 1.520 Predicted splice site
Splice_366 265474 265475 1.874 Predicted splice site
Splice_367 267270 267271 2.694 Predicted splice site
Splice_368 267647 267648 2.429 Predicted splice site
Splice_369 267803 267804 1.520 Predicted splice site
Splice_370 268000 268001 1.713 Predicted splice site
Splice_371 268229 268230 1.783 Predicted splice site
Splice_372 268316 268317 1.849 Predicted splice site
Splice_373 268443 268444 1.967 Predicted splice site
Splice_374 268562 268563 1.621 Predicted splice site
Splice_375 268725 268726 2.583 Predicted splice site

Predictions for the Reverse Strand of Chromosome 1

Splice_1 1113 1114 1.783 Predicted splice site
Splice_2 2043 2044 1.653 Predicted splice site
Splice_3 2492 2493 1.769 Predicted splice site
Splice_4 5063 5064 1.537 Predicted splice site
Splice_5 5291 5292 2.343 Predicted splice site
Splice_6 10065 10066 1.572 Predicted splice site
Splice_7 10400 10401 2.214 Predicted splice site
Splice_8 10631 10632 2.312 Predicted splice site
Splice_9 11517 11518 1.554 Predicted splice site
Splice_10 12184 12185 1.742 Predicted splice site
Splice_11 12733 12734 1.668 Predicted splice site
Splice_12 17301 17302 1.501 Predicted splice site
Splice_13 23726 23727 1.537 Predicted splice site
Splice_14 25024 25025 1.823 Predicted splice site
Splice_15 25567 25568 1.756 Predicted splice site
Splice_16 26467 26468 1.910 Predicted splice site
Splice_17 26619 26620 1.605 Predicted splice site
Splice_18 28701 28702 2.187 Predicted splice site
Splice_19 29116 29117 2.366 Predicted splice site
Splice_20 29176 29177 1.621 Predicted splice site
Splice_21 29457 29458 1.885 Predicted splice site
Splice_22 29914 29915 1.885 Predicted splice site
Splice_23 29990 29991 1.849 Predicted splice site
Splice_24 30061 30062 1.783 Predicted splice site
Splice_25 30166 30167 2.141 Predicted splice site
Splice_26 32082 32083 2.033 Predicted splice site
Splice_27 32682 32683 2.132 Predicted splice site
Splice_28 32831 32832 2.497 Predicted splice site
Splice_29 34998 34999 1.501 Predicted splice site
Splice_30 36925 36926 1.683 Predicted splice site
Splice_31 37273 37274 1.823 Predicted splice site
Splice_32 39844 39845 1.537 Predicted splice site
Splice_33 39985 39986 1.588 Predicted splice site
Splice_34 42769 42770 1.783 Predicted splice site
Splice_35 43615 43616 2.813 Predicted splice site
Splice_36 45030 45031 1.572 Predicted splice site
Splice_37 45654 45655 1.874 Predicted splice site
Splice_38 45817 45818 1.501 Predicted splice site
Splice_39 46978 46979 1.637 Predicted splice site
Splice_40 52858 52859 1.537 Predicted splice site
Splice_41 54289 54290 1.572 Predicted splice site
Splice_42 55888 55889 1.849 Predicted splice site
Splice_43 59033 59034 1.756 Predicted splice site

```
Splice_44 59092 59093 1.572 Predicted splice site
Splice_45 61587 61588 1.783 Predicted splice site
Splice_46 62917 62918 1.554 Predicted splice site
Splice_47 63726 63727 1.713 Predicted splice site
Splice_48 64512 64513 1.769 Predicted splice site
Splice_49 66144 66145 1.756 Predicted splice site
Splice_50 66225 66226 1.910 Predicted splice site
Splice_51 68301 68302 1.922 Predicted splice site
Splice_52 68597 68598 1.783 Predicted splice site
Splice_53 70179 70180 1.520 Predicted splice site
Splice_54 73844 73845 2.141 Predicted splice site
Splice_55 75703 75704 1.698 Predicted splice site
Splice_56 75874 75875 2.122 Predicted splice site
Splice_57 76238 76239 2.043 Predicted splice site
Splice_58 79835 79836 1.713 Predicted splice site
Splice_59 80073 80074 2.064 Predicted splice site
Splice_60 80722 80723 1.910 Predicted splice site
Splice_61 81721 81722 2.053 Predicted splice site
Splice_62 83763 83764 2.022 Predicted splice site
Splice_63 84103 84104 1.713 Predicted splice site
Splice_64 84192 84193 1.520 Predicted splice site
Splice_65 84893 84894 2.053 Predicted splice site
Splice_66 86717 86718 1.849 Predicted splice site
Splice_67 88266 88267 1.874 Predicted splice site
Splice_68 88610 88611 1.713 Predicted splice site
Splice_69 89399 89400 1.605 Predicted splice site
Splice_70 91758 91759 1.520 Predicted splice site
Splice_71 92318 92319 2.093 Predicted splice site
Splice_72 92666 92667 1.572 Predicted splice site
Splice_73 93461 93462 1.621 Predicted splice site
Splice_74 93903 93904 1.713 Predicted splice site
Splice_75 98422 98423 1.668 Predicted splice site
Splice_76 102757 102758 1.797 Predicted splice site
Splice_77 102902 102903 1.698 Predicted splice site
Splice_78 104112 104113 1.809 Predicted splice site
Splice_79 104387 104388 1.668 Predicted splice site
Splice_80 104463 104464 1.572 Predicted splice site
Splice_81 106671 106672 1.861 Predicted splice site
Splice_82 107152 107153 1.537 Predicted splice site
Splice_83 107220 107221 1.742 Predicted splice site
Splice_84 110562 110563 2.320 Predicted splice site
Splice_85 112150 112151 1.835 Predicted splice site
Splice_86 113004 113005 2.103 Predicted splice site
Splice_87 114430 114431 1.554 Predicted splice site
Splice_88 114615 114616 2.043 Predicted splice site
```

Splice_89 117565 117566 1.554 Predicted splice site
Splice_90 121919 121920 1.885 Predicted splice site
Splice_91 122864 122865 2.280 Predicted splice site
Splice_92 126444 126445 2.437 Predicted splice site
Splice_93 127500 127501 1.588 Predicted splice site
Splice_94 127964 127965 2.541 Predicted splice site
Splice_95 128760 128761 1.572 Predicted splice site
Splice_96 131992 131993 1.537 Predicted splice site
Splice_97 132193 132194 1.698 Predicted splice site
Splice_98 133502 133503 2.256 Predicted splice site
Splice_99 133684 133685 2.033 Predicted splice site
Splice_100 133757 133758 1.809 Predicted splice site
Splice_101 134545 134546 1.520 Predicted splice site
Splice_102 134900 134901 1.861 Predicted splice site
Splice_103 135793 135794 1.967 Predicted splice site
Splice_104 141273 141274 1.537 Predicted splice site
Splice_105 141587 141588 1.934 Predicted splice site
Splice_106 141709 141710 1.501 Predicted splice site
Splice_107 142507 142508 1.967 Predicted splice site
Splice_108 143822 143823 1.520 Predicted splice site
Splice_109 145132 145133 1.769 Predicted splice site
Splice_110 145423 145424 1.797 Predicted splice site
Splice_111 146496 146497 1.605 Predicted splice site
Splice_112 146999 147000 2.187 Predicted splice site
Splice_113 148124 148125 2.001 Predicted splice site
Splice_114 148736 148737 1.849 Predicted splice site
Splice_115 149312 149313 1.728 Predicted splice site
Splice_116 149677 149678 1.621 Predicted splice site
Splice_117 149875 149876 1.588 Predicted splice site
Splice_118 150304 150305 1.910 Predicted splice site
Splice_119 150888 150889 1.520 Predicted splice site
Splice_120 153760 153761 2.103 Predicted splice site
Splice_121 154521 154522 2.012 Predicted splice site
Splice_122 155460 155461 2.358 Predicted splice site
Splice_123 155730 155731 1.537 Predicted splice site
Splice_124 156216 156217 2.288 Predicted splice site
Splice_125 158014 158015 2.150 Predicted splice site
Splice_126 158168 158169 1.713 Predicted splice site
Splice_127 158226 158227 1.588 Predicted splice site
Splice_128 158740 158741 1.849 Predicted splice site
Splice_129 159372 159373 2.093 Predicted splice site
Splice_130 159762 159763 2.150 Predicted splice site
Splice_131 159897 159898 1.520 Predicted splice site
Splice_132 160750 160751 1.605 Predicted splice site
Splice_133 162725 162726 1.698 Predicted splice site

Splice_134 163046 163047 1.922 Predicted splice site
Splice_135 164706 164707 1.605 Predicted splice site
Splice_136 165482 165483 1.683 Predicted splice site
Splice_137 165671 165672 1.501 Predicted splice site
Splice_138 167118 167119 1.979 Predicted splice site
Splice_139 167315 167316 2.033 Predicted splice site
Splice_140 167488 167489 2.141 Predicted splice site
Splice_141 167878 167879 2.053 Predicted splice site
Splice_142 170987 170988 1.637 Predicted splice site
Splice_143 172543 172544 1.554 Predicted splice site
Splice_144 172660 172661 1.605 Predicted splice site
Splice_145 174637 174638 1.823 Predicted splice site
Splice_146 174790 174791 1.922 Predicted splice site
Splice_147 174916 174917 1.698 Predicted splice site
Splice_148 178063 178064 2.150 Predicted splice site
Splice_149 178429 178430 1.698 Predicted splice site
Splice_150 179162 179163 1.898 Predicted splice site
Splice_151 179448 179449 1.783 Predicted splice site
Splice_152 179509 179510 1.637 Predicted splice site
Splice_153 179697 179698 1.501 Predicted splice site
Splice_154 179803 179804 1.713 Predicted splice site
Splice_155 180429 180430 2.043 Predicted splice site
Splice_156 180499 180500 1.769 Predicted splice site
Splice_157 183403 183404 2.033 Predicted splice site
Splice_158 183758 183759 1.683 Predicted splice site
Splice_159 184252 184253 1.835 Predicted splice site
Splice_160 184390 184391 1.989 Predicted splice site
Splice_161 184493 184494 1.537 Predicted splice site
Splice_162 187295 187296 2.043 Predicted splice site
Splice_163 187640 187641 1.683 Predicted splice site
Splice_164 187952 187953 1.588 Predicted splice site
Splice_165 188061 188062 1.835 Predicted splice site
Splice_166 188145 188146 1.823 Predicted splice site
Splice_167 188415 188416 1.797 Predicted splice site
Splice_168 190046 190047 1.683 Predicted splice site
Splice_169 190167 190168 1.520 Predicted splice site
Splice_170 190338 190339 1.742 Predicted splice site
Splice_171 190760 190761 1.945 Predicted splice site
lmaj_01_031 191601 192767 0 acidocalcisomal exopolyphosphatase,
putative
Splice_172 191953 191954 1.713 Predicted splice site
Splice_173 193241 193242 1.922 Predicted splice site
Splice_174 193325 193326 1.501 Predicted splice site
lmaj_01_030 193331 193510 0 hypothetical protein,
conserved

lmaj_01_029 194432 194950 0 hypothetical protein,
conserved
Splice_175 195263 195264 1.637 Predicted splice site
Splice_176 195367 195368 2.150 Predicted splice site
Splice_177 195922 195923 1.637 Predicted splice site
Splice_178 196136 196137 1.621 Predicted splice site
lmaj_01_028 196197 197438 0 pseudouridylate synthase-like
protein
Splice_179 196228 196229 1.520 Predicted splice site
Splice_180 198343 198344 2.214 Predicted splice site
Splice_181 198506 198507 2.304 Predicted splice site
Splice_182 198903 198904 1.572 Predicted splice site
lmaj_01_027 199077 199400 0 thioredoxin,
putative
Splice_183 199671 199672 1.861 Predicted splice site
Splice_184 200088 200089 2.230 Predicted splice site
Splice_185 200210 200211 1.572 Predicted splice site
Splice_186 200266 200267 1.554 Predicted splice site
Splice_187 200477 200478 2.577 Predicted splice site
Splice_188 200612 200613 1.742 Predicted splice site
lmaj_01_026 200696 201541 0 hypothetical protein,
conserved
Splice_189 201895 201896 1.967 Predicted splice site
Splice_190 202132 202133 2.033 Predicted splice site
Splice_191 202192 202193 1.537 Predicted splice site
Splice_192 202373 202374 1.756 Predicted splice site
Splice_193 202736 202737 2.601 Predicted splice site
lmaj_01_025 202999 205365 0 hypothetical protein,
conserved
Splice_194 204182 204183 1.756 Predicted splice site
Splice_195 204868 204869 1.637 Predicted splice site
Splice_196 206074 206075 2.914 Predicted splice site
Splice_197 206135 206136 1.588 Predicted splice site
lmaj_01_024 206151 208928 0 DNA excision/repair protein SNF2,
putative
Splice_198 207478 207479 1.501 Predicted splice site
Splice_199 209011 209012 1.898 Predicted splice site
Splice_200 209184 209185 2.640 Predicted splice site
lmaj_01_023 209731 210147 0 hypothetical protein,
conserved
Splice_201 210185 210186 1.572 Predicted splice site
Splice_202 210507 210508 1.783 Predicted splice site
Splice_203 210808 210809 2.429 Predicted splice site
lmaj_01_022 210951 211958 0 cyclophilin type
peptidyl-prolyl cis-trans

Splice_204 212425 212426 2.327 Predicted splice site
Splice_205 212564 212565 2.053 Predicted splice site
Splice_206 212814 212815 1.588 Predicted splice site
Splice_207 213074 213075 1.874 Predicted splice site
Splice_208 213209 213210 2.343 Predicted splice site
Splice_209 215223 215224 1.756 Predicted splice site
Splice_210 215379 215380 2.280 Predicted splice site
lmaj_01_021 215614 218307 0 hypothetical protein,
conserved
Splice_211 218657 218658 1.537 Predicted splice site
Splice_212 218717 218718 1.621 Predicted splice site
Splice_213 218948 218949 1.910 Predicted splice site
Splice_214 219078 219079 2.350 Predicted splice site
lmaj_01_020 219232 220134 0 hypothetical protein,
conserved
Splice_215 219283 219284 2.343 Predicted splice site
Splice_216 220282 220283 2.280 Predicted splice site
Splice_217 220469 220470 1.783 Predicted splice site
Splice_218 220715 220716 1.742 Predicted splice site
lmaj_01_019 220847 221437 0 hypothetical protein,
conserved
Splice_219 221747 221748 1.783 Predicted splice site
Splice_220 221839 221840 2.033 Predicted splice site
Splice_221 222105 222106 2.195 Predicted splice site
Splice_222 222256 222257 1.653 Predicted splice site
Splice_223 222428 222429 1.728 Predicted splice site
lmaj_01_018 222520 224838 0 CLC-type chloride channel,
putative
Splice_224 222545 222546 1.713 Predicted splice site
Splice_225 224986 224987 1.979 Predicted splice site
Splice_226 225189 225190 2.710 Predicted splice site
Splice_227 225386 225387 2.230 Predicted splice site
Splice_228 225512 225513 2.335 Predicted splice site
Splice_229 226005 226006 1.898 Predicted splice site
Splice_230 226172 226173 1.572 Predicted splice site
lmaj_01_017 226337 227713 0 hypothetical protein,
unknown function
Splice_231 227484 227485 1.835 Predicted splice site
Splice_232 228422 228423 1.621 Predicted splice site
Splice_233 229545 229546 1.637 Predicted splice site
Splice_234 229728 229729 2.296 Predicted splice site
lmaj_01_016 229803 230045 0 hypothetical protein,
unknown function
Splice_235 229819 229820 1.835 Predicted splice site
Splice_236 230361 230362 2.571 Predicted splice site

Splice_237 230592 230593 2.141 Predicted splice site
Splice_238 230736 230737 2.429 Predicted splice site
lmaj_01_015 230921 231271 0 hypothetical protein,
conserved
Splice_239 231327 231328 1.572 Predicted splice site
Splice_240 231577 231578 2.221 Predicted splice site
lmaj_01_014 231691 232107 0 hypothetical protein,
conserved
Splice_241 232390 232391 2.387 Predicted splice site
Splice_242 232478 232479 1.989 Predicted splice site
Splice_243 232728 232729 2.914 Predicted splice site
Splice_244 232838 232839 2.204 Predicted splice site
Splice_245 233791 233792 1.572 Predicted splice site
Splice_246 234041 234042 1.934 Predicted splice site
lmaj_01_013 234261 235355 0 hypothetical protein,
conserved
Splice_247 235630 235631 2.471 Predicted splice site
Splice_248 236068 236069 1.797 Predicted splice site
Splice_249 236360 236361 2.652 Predicted splice site
Splice_250 236525 236526 1.554 Predicted splice site
Splice_251 236768 236769 2.947 Predicted splice site
lmaj_01_012 237409 238707 0 hypothetical protein,
conserved
Splice_252 238997 238998 1.554 Predicted splice site
Splice_253 239116 239117 1.668 Predicted splice site
Splice_254 239209 239210 1.797 Predicted splice site
Splice_255 239609 239610 2.132 Predicted splice site
Splice_256 239866 239867 2.001 Predicted splice site
lmaj_01_011 239877 240464 0 glutaredoxin-like protein
Splice_257 241194 241195 2.064 Predicted splice site
lmaj_01_010 241624 242580 0 hypothetical protein,
conserved
Splice_258 242778 242779 1.653 Predicted splice site
Splice_259 242984 242985 1.621 Predicted splice site
Splice_260 243248 243249 1.653 Predicted splice site
lmaj_01_009 243302 244126 0 hypothetical protein,
conserved
Splice_261 244360 244361 1.637 Predicted splice site
Splice_262 244464 244465 2.150 Predicted splice site
Splice_263 245001 245002 1.501 Predicted splice site
Splice_264 245517 245518 2.160 Predicted splice site
lmaj_01_008 245659 246372 0 hypothetical protein,
conserved
Splice_265 245706 245707 2.490 Predicted splice site
Splice_266 246461 246462 1.874 Predicted splice site

Splice_267 246881 246882 2.093 Predicted splice site
Splice_268 246979 246980 1.979 Predicted splice site
lmaj_01_007 247238 249034 0 hypothetical protein,
conserved
Splice_269 247251 247252 2.122 Predicted splice site
Splice_270 247908 247909 1.835 Predicted splice site
Splice_271 248500 248501 1.769 Predicted splice site
Splice_272 249230 249231 1.885 Predicted splice site
Splice_273 249322 249323 2.033 Predicted splice site
Splice_274 249481 249482 1.957 Predicted splice site
Splice_275 249544 249545 1.668 Predicted splice site
Splice_276 249703 249704 1.713 Predicted splice site
Splice_277 249894 249895 2.735 Predicted splice site
Splice_278 250057 250058 1.520 Predicted splice site
lmaj_01_006 250099 250848 0 hypothetical protein,
conserved
Splice_279 251189 251190 2.204 Predicted splice site
Splice_280 251598 251599 2.380 Predicted splice site
Splice_281 251783 251784 2.288 Predicted splice site
Splice_282 251942 251943 2.471 Predicted splice site
lmaj_01_005 251963 253960 0 carboxylase,
putative
Splice_283 254148 254149 2.239 Predicted splice site
Splice_284 254464 254465 2.720 Predicted splice site
Splice_285 254544 254545 1.898 Predicted splice site
Splice_286 254609 254610 1.653 Predicted splice site
Splice_287 254686 254687 1.861 Predicted splice site
Splice_288 254766 254767 1.898 Predicted splice site
Splice_289 255356 255357 3.220 Predicted splice site
Splice_290 255466 255467 1.823 Predicted splice site
Splice_291 255550 255551 1.728 Predicted splice site
Splice_292 256017 256018 2.150 Predicted splice site
Splice_293 256110 256111 1.572 Predicted splice site
Splice_294 256238 256239 2.350 Predicted splice site
Splice_295 256339 256340 2.122 Predicted splice site
lmaj_01_004 256342 256911 0 hypothetical protein,
unknown function
Splice_296 257327 257328 2.662 Predicted splice site
Splice_297 257539 257540 1.742 Predicted splice site
Splice_298 257680 257681 1.910 Predicted splice site
Splice_299 257773 257774 1.520 Predicted splice site
lmaj_01_003 257917 259923 0 MCAK-like kinesin,
putative
Splice_300 259431 259432 1.653 Predicted splice site
Splice_301 260061 260062 2.272 Predicted splice site

Splice_302 260329 260330 2.409 Predicted splice site
Splice_303 260547 260548 2.547 Predicted splice site
Splice_304 260758 260759 1.653 Predicted splice site
Splice_305 260875 260876 2.264 Predicted splice site
Splice_306 260953 260954 1.874 Predicted splice site
Splice_307 261223 261224 1.769 Predicted splice site
Splice_308 261351 261352 2.350 Predicted splice site
Splice_309 261411 261412 1.621 Predicted splice site
lmaj_01_002 261545 263194 0 hypothetical protein,
conserved
Splice_310 263188 263189 1.922 Predicted splice site
Splice_311 263267 263268 1.572 Predicted splice site
Splice_312 263879 263880 1.728 Predicted splice site
Splice_313 264033 264034 2.103 Predicted splice site
lmaj_01_001 264282 265280 0 hypothetical protein,
unknown function
Splice_314 265074 265075 1.898 Predicted splice site
Splice_315 265513 265514 2.679 Predicted splice site
Splice_316 265575 265576 1.572 Predicted splice site
Splice_317 265640 265641 1.698 Predicted splice site
Splice_318 265739 265740 2.103 Predicted splice site
Splice_319 265973 265974 2.559 Predicted splice site
Splice_320 266238 266239 2.652 Predicted splice site
Splice_321 266417 266418 1.698 Predicted splice site
Splice_322 266482 266483 1.621 Predicted splice site
Splice_323 266730 266731 2.751 Predicted splice site
Splice_324 266803 266804 1.501 Predicted splice site
Splice_325 267296 267297 2.001 Predicted splice site
Splice_326 267446 267447 1.885 Predicted splice site
Splice_327 267511 267512 1.698 Predicted splice site
Splice_328 268016 268017 2.541 Predicted splice site
Splice_329 268141 268142 1.554 Predicted splice site
Splice_330 268288 268289 2.073 Predicted splice site
Splice_331 268398 268399 2.132 Predicted splice site

Predictions for the Forward Strand of Chromosome 3

Splice_1 5437 5438 1.683 Predicted splice site
lmaj_03_002 5471 6277 0 hypothetical protein,
unknown function
lmaj_03_003 6699 8234 0 D-3-phosphoglycerate
dehydrogenase-like protein
Splice_2 6832 6833 1.653 Predicted splice site
Splice_3 6925 6926 1.605 Predicted splice site
Splice_4 7017 7018 1.588 Predicted splice site
Splice_5 8539 8540 1.967 Predicted splice site
lmaj_03_004 8999 10306 0 2-aminoethylphosphonate:pyruvate
Splice_6 9058 9059 1.934 Predicted splice site
Splice_7 9145 9146 1.979 Predicted splice site
Splice_8 9596 9597 1.756 Predicted splice site
Splice_9 10460 10461 1.934 Predicted splice site
Splice_10 10532 10533 1.797 Predicted splice site
Splice_11 10712 10713 2.012 Predicted splice site
Splice_12 11243 11244 1.742 Predicted splice site
Splice_13 11377 11378 2.395 Predicted splice site
lmaj_03_005 11551 11754 0 hypothetical protein,
unknown function
Splice_14 12042 12043 1.756 Predicted splice site
Splice_15 12508 12509 1.572 Predicted splice site
Splice_16 12721 12722 2.221 Predicted splice site
Splice_17 12899 12900 1.742 Predicted splice site
Splice_18 13075 13076 2.150 Predicted splice site
Splice_19 13333 13334 1.683 Predicted splice site
Splice_20 13466 13467 1.783 Predicted splice site
lmaj_03_006 13513 14685 0 hypothetical protein,
unknown function
Splice_21 15258 15259 2.103 Predicted splice site
Splice_22 15381 15382 2.312 Predicted splice site
Splice_23 15460 15461 1.588 Predicted splice site
lmaj_03_007 15549 18050 0 hypothetical protein,
unknown function
Splice_24 16324 16325 1.520 Predicted splice site
Splice_25 17002 17003 1.554 Predicted splice site
Splice_26 17500 17501 2.064 Predicted splice site
Splice_27 18179 18180 2.012 Predicted splice site
Splice_28 18395 18396 2.372 Predicted splice site
lmaj_03_008 18824 20710 0 Transporter Protein,
putative
Splice_29 20381 20382 1.823 Predicted splice site
Splice_30 20449 20450 1.742 Predicted splice site

Splice_31 21142 21143 1.637 Predicted splice site
Splice_32 21295 21296 2.264 Predicted splice site
Splice_33 21555 21556 1.698 Predicted splice site
Splice_34 21632 21633 1.554 Predicted splice site
Splice_35 21695 21696 1.668 Predicted splice site
Splice_36 21957 21958 2.264 Predicted splice site
lmaj_03_009 21972 23675 0 DNA primase large subunit p58-like protein
Splice_37 22852 22853 1.756 Predicted splice site
Splice_38 23122 23123 1.572 Predicted splice site
Splice_39 24074 24075 1.922 Predicted splice site
Splice_40 24279 24280 1.588 Predicted splice site
Splice_41 24576 24577 1.537 Predicted splice site
Splice_42 24839 24840 2.132 Predicted splice site
lmaj_03_010 25140 25634 0 Cytochrome c oxidase assembly protein, putative
Splice_43 26028 26029 2.187 Predicted splice site
Splice_44 26151 26152 2.103 Predicted splice site
Splice_45 26416 26417 2.629 Predicted splice site
Splice_46 26487 26488 1.537 Predicted splice site
lmaj_03_011 26692 29559 0 hypothetical protein, unknown function
Splice_47 26753 26754 1.554 Predicted splice site
Splice_48 26834 26835 1.910 Predicted splice site
Splice_49 27633 27634 1.554 Predicted splice site
Splice_50 29994 29995 1.520 Predicted splice site
Splice_51 30084 30085 1.957 Predicted splice site
lmaj_03_012 30321 33098 0 hypothetical protein, unknown function
Splice_52 30437 30438 1.668 Predicted splice site
Splice_53 32864 32865 1.520 Predicted splice site
Splice_54 33391 33392 1.728 Predicted splice site
Splice_55 33992 33993 1.910 Predicted splice site
Splice_56 34076 34077 1.653 Predicted splice site
lmaj_03_013 34115 34663 0 hypothetical protein, unknown function
Splice_57 35036 35037 1.922 Predicted splice site
Splice_58 35459 35460 2.770 Predicted splice site
lmaj_03_014 35547 35966 0 hypothetical protein, unknown function
Splice_59 35569 35570 1.885 Predicted splice site
Splice_60 36042 36043 1.698 Predicted splice site
Splice_61 36148 36149 1.797 Predicted splice site
Splice_62 36409 36410 1.910 Predicted splice site
Splice_63 36495 36496 1.967 Predicted splice site

Splice_64 36983 36984 1.756 Predicted splice site
Splice_65 37114 37115 2.033 Predicted splice site
lmaj_03_015 37224 38159 0 hypothetical protein,
unknown function
Splice_66 38573 38574 1.769 Predicted splice site
Splice_67 38770 38771 1.967 Predicted splice site
Splice_68 38973 38974 2.689 Predicted splice site
lmaj_03_016 39082 41256 0 ABC transporter protein,
putative
Splice_69 40322 40323 1.520 Predicted splice site
Splice_70 40376 40377 1.520 Predicted splice site
Splice_71 41821 41822 1.537 Predicted splice site
Splice_72 42028 42029 2.001 Predicted splice site
lmaj_03_017 42231 42809 0 hypothetical protein,
unknown function
Splice_73 43013 43014 2.132 Predicted splice site
lmaj_03_018 43600 45291 0 hypothetical protein,
unknown function
Splice_74 43871 43872 2.195 Predicted splice site
Splice_75 45658 45659 1.922 Predicted splice site
Splice_76 45924 45925 2.765 Predicted splice site
lmaj_03_019 46204 46836 0 U2AF23/25 spliceosome component,
putative
Splice_77 46349 46350 1.797 Predicted splice site
Splice_78 47588 47589 2.366 Predicted splice site
Splice_79 47700 47701 1.520 Predicted splice site
Splice_80 48151 48152 1.728 Predicted splice site
Splice_81 48240 48241 1.537 Predicted splice site
Splice_82 48379 48380 1.874 Predicted splice site
lmaj_03_020 48406 50088 0 delta-1-pyrroline-5-carboxylate
dehydrogenase,
Splice_83 50560 50561 2.483 Predicted splice site
Splice_84 50702 50703 2.312 Predicted splice site
Splice_85 50837 50838 1.989 Predicted splice site
Splice_86 51593 51594 2.296 Predicted splice site
Splice_87 51749 51750 2.168 Predicted splice site
Splice_88 51876 51877 1.835 Predicted splice site
lmaj_03_021 51987 53126 0 Protein kinase,
putative
Splice_89 53656 53657 1.934 Predicted splice site
Splice_90 53885 53886 1.683 Predicted splice site
lmaj_03_022 54144 55085 0 hypothetical protein,
unknown function
Splice_91 55254 55255 1.683 Predicted splice site
Splice_92 55621 55622 2.033 Predicted splice site

lmaj_03_023 56016 58130 0 Long Chain Fatty Acyl
CoA Synthetase, putative
Splice_93 57850 57851 1.874 Predicted splice site
Splice_94 58428 58429 1.783 Predicted splice site
Splice_95 58654 58655 1.849 Predicted splice site
Splice_96 58730 58731 1.849 Predicted splice site
Splice_97 58860 58861 2.122 Predicted splice site
Splice_98 59151 59152 2.012 Predicted splice site
Splice_99 59759 59760 1.728 Predicted splice site
Splice_100 59903 59904 2.160 Predicted splice site
Splice_101 59995 59996 1.934 Predicted splice site
Splice_102 60355 60356 2.673 Predicted splice site
Splice_103 60598 60599 2.221 Predicted splice site
Splice_104 60778 60779 2.247 Predicted splice site
Splice_105 60871 60872 2.043 Predicted splice site
lmaj_03_024 61005 61469 0 hypothetical protein,
unknown function
Splice_106 61597 61598 1.537 Predicted splice site
Splice_107 61678 61679 1.910 Predicted splice site
Splice_108 62043 62044 2.700 Predicted splice site
lmaj_03_025 62112 62363 0 ribosomal protein L38,
putative
Splice_109 62715 62716 1.501 Predicted splice site
Splice_110 63348 63349 1.989 Predicted splice site
Splice_111 63435 63436 1.783 Predicted splice site
Splice_112 63546 63547 2.214 Predicted splice site
lmaj_03_026 63660 64568 0 hypothetical protein,
unknown function
Splice_113 64403 64404 1.809 Predicted splice site
Splice_114 64851 64852 1.588 Predicted splice site
Splice_115 65324 65325 2.103 Predicted splice site
lmaj_03_027 66061 73125 0 hypothetical protein,
unknown function
Splice_116 73267 73268 1.653 Predicted splice site
Splice_117 73783 73784 2.239 Predicted splice site
lmaj_03_028 74031 76958 0 hypothetical protein,
unknown function
Splice_118 75437 75438 1.637 Predicted splice site
Splice_119 75735 75736 1.605 Predicted splice site
Splice_120 75883 75884 2.187 Predicted splice site
Splice_121 77255 77256 1.783 Predicted splice site
Splice_122 77336 77337 1.910 Predicted splice site
Splice_123 77451 77452 1.653 Predicted splice site
lmaj_03_029 77815 78075 0 hypothetical protein,
unknown function

Splice_124 78650 78651 1.605 Predicted splice site
Splice_125 78799 78800 1.823 Predicted splice site
lmaj_03_030 78812 79147 0 hypothetical protein,
unknown function
Splice_126 79491 79492 1.756 Predicted splice site
Splice_127 79912 79913 2.450 Predicted splice site
Splice_128 80109 80110 1.520 Predicted splice site
Splice_129 80202 80203 1.861 Predicted splice site
Splice_130 80293 80294 1.874 Predicted splice site
Splice_131 80351 80352 1.537 Predicted splice site
Splice_132 80432 80433 1.910 Predicted splice site
Splice_133 80800 80801 1.554 Predicted splice site
Splice_134 80933 80934 2.160 Predicted splice site
Splice_135 81163 81164 2.350 Predicted splice site
Splice_136 81325 81326 2.477 Predicted splice site
Splice_137 81403 81404 1.874 Predicted splice site
Splice_138 81554 81555 2.444 Predicted splice site
Splice_139 81649 81650 2.064 Predicted splice site
Splice_140 81728 81729 1.885 Predicted splice site
Splice_141 81960 81961 2.612 Predicted splice site
Splice_142 82233 82234 2.528 Predicted splice site
Splice_143 82417 82418 2.700 Predicted splice site
Splice_144 82633 82634 1.934 Predicted splice site
Splice_145 82750 82751 2.264 Predicted splice site
lmaj_03_031 82959 89222 0 hypothetical protein,
unknown function
Splice_146 83016 83017 2.876 Predicted splice site
Splice_147 89450 89451 1.668 Predicted splice site
Splice_148 89950 89951 2.141 Predicted splice site
Splice_149 90146 90147 2.256 Predicted splice site
lmaj_03_032 90199 93297 0 hypothetical protein,
unknown function
Splice_150 93490 93491 1.967 Predicted splice site
Splice_151 93604 93605 1.572 Predicted splice site
Splice_152 94173 94174 1.809 Predicted splice site
Splice_153 94232 94233 1.605 Predicted splice site
lmaj_03_033 94562 96454 0 hypothetical protein,
unknown function
Splice_154 96682 96683 1.945 Predicted splice site
Splice_155 96863 96864 1.849 Predicted splice site
lmaj_03_034 97234 98367 0 hypothetical protein,
unknown function
Splice_156 97270 97271 2.589 Predicted splice site
Splice_157 98679 98680 1.668 Predicted splice site
Splice_158 98770 98771 2.022 Predicted splice site

Splice_159 99198 99199 2.565 Predicted splice site
Splice_160 99251 99252 1.501 Predicted splice site
Splice_161 99393 99394 2.450 Predicted splice site
Splice_162 99529 99530 1.945 Predicted splice site
lmaj_03_035 99584 104167 0 hypothetical protein,
unknown function
Splice_163 101633 101634 1.572 Predicted splice site
Splice_164 102367 102368 1.698 Predicted splice site
Splice_165 104539 104540 1.979 Predicted splice site
Splice_166 104783 104784 1.957 Predicted splice site
Splice_167 104868 104869 1.683 Predicted splice site
Splice_168 105389 105390 1.520 Predicted splice site
Splice_169 105492 105493 1.668 Predicted splice site
Splice_170 105581 105582 1.957 Predicted splice site
Splice_171 106529 106530 2.064 Predicted splice site
Splice_172 106647 106648 2.012 Predicted splice site
Splice_173 106873 106874 2.705 Predicted splice site
Splice_174 107193 107194 3.190 Predicted splice site
Splice_175 107366 107367 2.084 Predicted splice site
Splice_176 107448 107449 1.922 Predicted splice site
Splice_177 107551 107552 1.898 Predicted splice site
Splice_178 107720 107721 2.150 Predicted splice site
Splice_179 107821 107822 1.989 Predicted splice site
lmaj_03_036 108060 113042 0 hypothetical protein,
unknown function
Splice_180 109092 109093 1.537 Predicted splice site
Splice_181 109200 109201 1.698 Predicted splice site
Splice_182 110027 110028 1.713 Predicted splice site
Splice_183 110131 110132 2.012 Predicted splice site
Splice_184 110327 110328 2.256 Predicted splice site
Splice_185 110541 110542 2.113 Predicted splice site
Splice_186 111717 111718 1.797 Predicted splice site
Splice_187 113260 113261 1.874 Predicted splice site
Splice_188 113376 113377 1.742 Predicted splice site
Splice_189 113433 113434 1.572 Predicted splice site
Splice_190 113558 113559 1.605 Predicted splice site
Splice_191 113958 113959 2.401 Predicted splice site
Splice_192 114160 114161 2.726 Predicted splice site
Splice_193 114339 114340 2.113 Predicted splice site
Splice_194 114620 114621 3.087 Predicted splice site
Splice_195 114760 114761 2.437 Predicted splice site
Splice_196 114828 114829 1.742 Predicted splice site
Splice_197 115011 115012 1.957 Predicted splice site
Splice_198 115204 115205 2.256 Predicted splice site
Splice_199 115346 115347 1.605 Predicted splice site

Splice_200 115501 115502 1.637 Predicted splice site
Splice_201 115619 115620 1.713 Predicted splice site
Splice_202 115945 115946 2.012 Predicted splice site
Splice_203 116021 116022 1.849 Predicted splice site
Splice_204 116297 116298 2.296 Predicted splice site
Splice_205 116417 116418 1.501 Predicted splice site
Splice_206 116553 116554 2.387 Predicted splice site
lmaj_03_037 116651 117583 0 conserved hypothetical protein, unknown
Splice_207 117326 117327 1.520 Predicted splice site
Splice_208 117408 117409 1.922 Predicted splice site
Splice_209 117968 117969 2.423 Predicted splice site
Splice_210 118326 118327 2.327 Predicted splice site
Splice_211 118467 118468 2.113 Predicted splice site
Splice_212 118800 118801 2.132 Predicted splice site
Splice_213 119000 119001 1.835 Predicted splice site
Splice_214 119113 119114 1.572 Predicted splice site
Splice_215 119415 119416 2.679 Predicted splice site
Splice_216 119623 119624 1.605 Predicted splice site
Splice_217 120163 120164 1.885 Predicted splice site
Splice_218 120468 120469 2.740 Predicted splice site
lmaj_03_038 120632 121744 0 conserved hypothetical protein, unknown
Splice_219 121487 121488 2.320 Predicted splice site
Splice_220 121891 121892 2.093 Predicted splice site
Splice_221 122238 122239 2.656 Predicted splice site
Splice_222 122500 122501 1.588 Predicted splice site
Splice_223 122687 122688 2.715 Predicted splice site
Splice_224 122975 122976 1.861 Predicted splice site
Splice_225 123086 123087 2.012 Predicted splice site
Splice_226 123422 123423 2.775 Predicted splice site
Splice_227 123540 123541 1.756 Predicted splice site
Splice_228 123742 123743 2.595 Predicted splice site
Splice_229 124219 124220 3.104 Predicted splice site
Splice_230 124427 124428 2.589 Predicted splice site
Splice_231 124611 124612 2.673 Predicted splice site
Splice_232 124714 124715 1.572 Predicted splice site
Splice_233 124842 124843 1.572 Predicted splice site
Splice_234 125042 125043 2.595 Predicted splice site
Splice_235 125140 125141 2.093 Predicted splice site
Splice_236 125253 125254 2.230 Predicted splice site
lmaj_03_039 125348 126784 0 conserved hypothetical protein, unknown
Splice_237 125913 125914 1.653 Predicted splice site
Splice_238 126001 126002 1.554 Predicted splice site

Splice_239 126200 126201 1.769 Predicted splice site
Splice_240 126739 126740 2.990 Predicted splice site
Splice_241 126856 126857 1.783 Predicted splice site
Splice_242 127066 127067 1.756 Predicted splice site
Splice_243 127152 127153 1.945 Predicted splice site
lmaj_03_040 127156 128067 0 conserved hypothetical protein,
unknown
Splice_244 127834 127835 2.320 Predicted splice site
Splice_245 128275 128276 1.537 Predicted splice site
Splice_246 128416 128417 1.501 Predicted splice site
Splice_247 128648 128649 2.477 Predicted splice site
Splice_248 128937 128938 2.429 Predicted splice site
Splice_249 129041 129042 1.637 Predicted splice site
Splice_250 129400 129401 1.957 Predicted splice site
Splice_251 129840 129841 2.093 Predicted splice site
Splice_252 130118 130119 1.910 Predicted splice site
Splice_253 130345 130346 2.214 Predicted splice site
Splice_254 130969 130970 1.501 Predicted splice site
Splice_255 131382 131383 1.898 Predicted splice site
Splice_256 131470 131471 1.637 Predicted splice site
Splice_257 131692 131693 1.572 Predicted splice site
lmaj_03_041 131775 133826 0 hypothetical protein,
unknown function
Splice_258 132088 132089 1.874 Predicted splice site
Splice_259 132207 132208 1.537 Predicted splice site
Splice_260 132603 132604 1.898 Predicted splice site
Splice_261 133207 133208 2.103 Predicted splice site
Splice_262 133295 133296 1.989 Predicted splice site
Splice_263 133490 133491 1.783 Predicted splice site
Splice_264 133600 133601 1.910 Predicted splice site
Splice_265 133920 133921 1.861 Predicted splice site
Splice_266 134347 134348 1.934 Predicted splice site
Splice_267 134581 134582 2.335 Predicted splice site
Splice_268 134642 134643 1.637 Predicted splice site
Splice_269 134868 134869 2.601 Predicted splice site
Splice_270 135007 135008 1.783 Predicted splice site
Splice_271 135547 135548 1.605 Predicted splice site
Splice_272 136125 136126 1.501 Predicted splice site
Splice_273 136216 136217 2.022 Predicted splice site
lmaj_03_042 136735 141885 0 hypothetical protein,
unknown function
Splice_274 138276 138277 1.668 Predicted splice site
Splice_275 138528 138529 1.554 Predicted splice site
Splice_276 139867 139868 1.698 Predicted splice site
Splice_277 141766 141767 1.554 Predicted splice site

Splice_278 142160 142161 1.698 Predicted splice site
Splice_279 142289 142290 2.358 Predicted splice site
Splice_280 142536 142537 1.683 Predicted splice site
Splice_281 142849 142850 2.746 Predicted splice site
lmaj_03_043 142929 143264 0 Ribosomal Protein P1, putative
Splice_282 142939 142940 1.885 Predicted splice site
Splice_283 143547 143548 2.043 Predicted splice site
Splice_284 143832 143833 2.247 Predicted splice site
lmaj_03_044 143880 144215 0 Ribosomal Protein P1, putative
Splice_285 144625 144626 1.728 Predicted splice site
Splice_286 144740 144741 2.122 Predicted splice site
lmaj_03_045 145028 150139 0 hypothetical protein, unknown function
Splice_287 146768 146769 1.501 Predicted splice site
Splice_288 150441 150442 1.849 Predicted splice site
Splice_289 150610 150611 2.618 Predicted splice site
Splice_290 150690 150691 1.605 Predicted splice site
Splice_291 150778 150779 1.989 Predicted splice site
Splice_292 151284 151285 2.756 Predicted splice site
Splice_293 151607 151608 1.621 Predicted splice site
Splice_294 151862 151863 2.522 Predicted splice site
Splice_295 152259 152260 2.918 Predicted splice site
Splice_296 152477 152478 2.457 Predicted splice site
Splice_297 152616 152617 1.957 Predicted splice site
Splice_298 152823 152824 1.823 Predicted splice site
Splice_299 153444 153445 2.673 Predicted splice site
Splice_300 153597 153598 1.501 Predicted splice site
Splice_301 153745 153746 2.490 Predicted splice site
Splice_302 153826 153827 1.769 Predicted splice site
Splice_303 153913 153914 1.979 Predicted splice site
Splice_304 153984 153985 1.728 Predicted splice site
Splice_305 154049 154050 1.668 Predicted splice site
Splice_306 154370 154371 2.033 Predicted splice site
Splice_307 154796 154797 1.572 Predicted splice site
Splice_308 155066 155067 2.343 Predicted splice site
Splice_309 155167 155168 1.910 Predicted splice site
Splice_310 155228 155229 1.637 Predicted splice site
Splice_311 155364 155365 1.554 Predicted splice site
lmaj_03_046 155374 158562 0 hypothetical protein, unknown function
Splice_312 156397 156398 1.501 Predicted splice site
Splice_313 156867 156868 1.957 Predicted splice site
Splice_314 159017 159018 1.783 Predicted splice site

Splice_315 159134 159135 2.103 Predicted splice site
Splice_316 159230 159231 1.979 Predicted splice site
Splice_317 159316 159317 1.967 Predicted splice site
Splice_318 159492 159493 2.589 Predicted splice site
Splice_319 159939 159940 2.808 Predicted splice site
Splice_320 160434 160435 1.554 Predicted splice site
Splice_321 160523 160524 2.001 Predicted splice site
Splice_322 162293 162294 1.797 Predicted splice site
Splice_323 162468 162469 2.064 Predicted splice site
Splice_324 162649 162650 1.823 Predicted splice site
Splice_325 162828 162829 2.559 Predicted splice site
Splice_326 163012 163013 1.835 Predicted splice site
Splice_327 163091 163092 1.572 Predicted splice site
Splice_328 163269 163270 2.168 Predicted splice site
lmaj_03_047 163294 166311 0 hypothetical protein,
unknown function
Splice_329 163819 163820 1.554 Predicted splice site
Splice_330 165500 165501 1.537 Predicted splice site
Splice_331 166462 166463 2.320 Predicted splice site
Splice_332 166531 166532 1.756 Predicted splice site
Splice_333 166954 166955 1.698 Predicted splice site
Splice_334 167072 167073 1.605 Predicted splice site
Splice_335 167223 167224 1.989 Predicted splice site
Splice_336 167449 167450 2.296 Predicted splice site
Splice_337 167524 167525 1.835 Predicted splice site
Splice_338 167599 167600 1.713 Predicted splice site
lmaj_03_048 167882 170911 0 hypothetical protein,
unknown function
Splice_339 169182 169183 1.742 Predicted splice site
Splice_340 169625 169626 1.554 Predicted splice site
Splice_341 169776 169777 1.797 Predicted splice site
Splice_342 170510 170511 1.769 Predicted splice site
Splice_343 171084 171085 1.621 Predicted splice site
Splice_344 171173 171174 1.554 Predicted splice site
Splice_345 172245 172246 2.668 Predicted splice site
lmaj_03_049 172571 175639 0 hypothetical protein,
unknown function
Splice_346 174735 174736 1.668 Predicted splice site
Splice_347 175106 175107 2.335 Predicted splice site
Splice_348 175225 175226 2.053 Predicted splice site
Splice_349 175805 175806 1.797 Predicted splice site
Splice_350 175981 175982 2.656 Predicted splice site
Splice_351 176215 176216 1.967 Predicted splice site
Splice_352 176628 176629 2.789 Predicted splice site
Splice_353 176849 176850 2.122 Predicted splice site

Splice_354 176968 176969 2.033 Predicted splice site
lmaj_03_050 176975 178693 0 Phosphate-Repressible
Phosphate Permease-like
Splice_355 177488 177489 2.033 Predicted splice site
Splice_356 177582 177583 1.756 Predicted splice site
Splice_357 178802 178803 1.945 Predicted splice site
Splice_358 178958 178959 1.769 Predicted splice site
Splice_359 179084 179085 2.335 Predicted splice site
Splice_360 179210 179211 2.335 Predicted splice site
Splice_361 179484 179485 2.818 Predicted splice site
Splice_362 179857 179858 1.554 Predicted splice site
Splice_363 180074 180075 1.874 Predicted splice site
Splice_364 180398 180399 2.571 Predicted splice site
Splice_365 180936 180937 2.084 Predicted splice site
Splice_366 181140 181141 1.797 Predicted splice site
lmaj_03_051 181367 188830 0 hypothetical protein,
unknown function
Splice_367 183275 183276 1.572 Predicted splice site
Splice_368 186504 186505 1.501 Predicted splice site
Splice_369 188431 188432 1.537 Predicted splice site
Splice_370 189339 189340 1.605 Predicted splice site
lmaj_03_052 189467 191743 0 hypothetical protein,
unknown function
Splice_371 191322 191323 2.012 Predicted splice site
Splice_372 192306 192307 1.572 Predicted splice site
Splice_373 192759 192760 1.698 Predicted splice site
lmaj_03_053 194007 200258 0 hypothetical protein,
unknown function
Splice_374 198761 198762 1.554 Predicted splice site
Splice_375 200795 200796 1.979 Predicted splice site
Splice_376 200980 200981 1.835 Predicted splice site
Splice_377 201344 201345 2.689 Predicted splice site
Splice_378 201457 201458 2.022 Predicted splice site
lmaj_03_054 201966 203156 0 26S Protease Regulatory Subunit,
putative
Splice_379 201970 201971 1.874 Predicted splice site
Splice_380 203287 203288 1.501 Predicted splice site
Splice_381 203397 203398 2.053 Predicted splice site
Splice_382 203612 203613 2.230 Predicted splice site
Splice_383 204044 204045 2.187 Predicted splice site
Splice_384 204157 204158 1.910 Predicted splice site
Splice_385 204246 204247 1.605 Predicted splice site
Splice_386 204315 204316 1.605 Predicted splice site
Splice_387 204628 204629 1.742 Predicted splice site
lmaj_03_055 204891 208841 0 hypothetical protein,

unknown function
Splice_388 205000 205001 1.698 Predicted splice site
Splice_389 205067 205068 1.520 Predicted splice site
Splice_390 206469 206470 1.742 Predicted splice site
Splice_391 206721 206722 2.001 Predicted splice site
Splice_392 208158 208159 2.204 Predicted splice site
Splice_393 208330 208331 1.621 Predicted splice site
Splice_394 208841 208842 1.653 Predicted splice site
Splice_395 209026 209027 2.103 Predicted splice site
Splice_396 209211 209212 1.554 Predicted splice site
Splice_397 209905 209906 2.662 Predicted splice site
Splice_398 210022 210023 2.264 Predicted splice site
Splice_399 210216 210217 2.387 Predicted splice site
lmaj_03_056 210310 212466 0 hypothetical protein,
unknown function
Splice_400 210704 210705 1.520 Predicted splice site
Splice_401 211581 211582 1.885 Predicted splice site
Splice_402 211707 211708 1.698 Predicted splice site
Splice_403 212031 212032 1.898 Predicted splice site
Splice_404 212780 212781 2.320 Predicted splice site
Splice_405 212873 212874 1.910 Predicted splice site
Splice_406 213037 213038 2.312 Predicted splice site
Splice_407 213298 213299 1.605 Predicted splice site
Splice_408 214979 214980 2.160 Predicted splice site
lmaj_03_057 215070 216068 0 Quinone Oxidoreductase,
putative
Splice_409 215220 215221 1.554 Predicted splice site
Splice_410 216174 216175 1.989 Predicted splice site
Splice_411 216453 216454 2.662 Predicted splice site
lmaj_03_058 216530 217066 0 hypothetical protein,
unknown function
Splice_412 217172 217173 1.605 Predicted splice site
Splice_413 217261 217262 1.728 Predicted splice site
Splice_414 217376 217377 2.247 Predicted splice site
Splice_415 217551 217552 1.588 Predicted splice site
Splice_416 218104 218105 1.861 Predicted splice site
Splice_417 218354 218355 2.230 Predicted splice site
Splice_418 218498 218499 2.239 Predicted splice site
Splice_419 218782 218783 1.885 Predicted splice site
lmaj_03_059 219039 223118 0 hypothetical protein,
unknown function
Splice_420 223432 223433 1.572 Predicted splice site
Splice_421 223573 223574 2.122 Predicted splice site
Splice_422 223667 223668 1.922 Predicted splice site
lmaj_03_060 223728 224945 0 Protein Arginine

MethylTransferase-like protein
Splice_423 225622 225623 2.084 Predicted splice site
Splice_424 226134 226135 1.979 Predicted splice site
Splice_425 226228 226229 1.520 Predicted splice site
Splice_426 226396 226397 1.554 Predicted splice site
lmaj_03_061 226471 227520 0 Kinase-like protein
Splice_427 227908 227909 1.554 Predicted splice site
Splice_428 227969 227970 1.637 Predicted splice site
Splice_429 228126 228127 1.898 Predicted splice site
Splice_430 228385 228386 1.520 Predicted splice site
Splice_431 228520 228521 2.221 Predicted splice site
lmaj_03_062 228710 230737 0 conserved hypothetical protein,
unknown
Splice_432 232123 232124 2.423 Predicted splice site
Splice_433 232225 232226 2.132 Predicted splice site
lmaj_03_063 232372 233349 0 hypothetical protein,
unknown function
Splice_434 232669 232670 1.537 Predicted splice site
Splice_435 233105 233106 1.713 Predicted splice site
Splice_436 233735 233736 2.280 Predicted splice site
Splice_437 233810 233811 1.835 Predicted splice site
lmaj_03_064 234148 235989 0 hypothetical protein,
unknown function
Splice_438 234193 234194 2.497 Predicted splice site
Splice_439 235618 235619 1.554 Predicted splice site
Splice_440 236295 236296 1.537 Predicted splice site
Splice_441 236783 236784 1.823 Predicted splice site
Splice_442 236961 236962 1.537 Predicted splice site
Splice_443 237504 237505 2.372 Predicted splice site
Splice_444 237593 237594 1.967 Predicted splice site
Splice_445 237704 237705 2.168 Predicted splice site
Splice_446 237818 237819 2.239 Predicted splice site
Splice_447 238157 238158 2.893 Predicted splice site
Splice_448 238370 238371 2.122 Predicted splice site
lmaj_03_065 238454 241135 0 hypothetical protein,
unknown function
Splice_449 238491 238492 2.280 Predicted splice site
Splice_450 238578 238579 1.572 Predicted splice site
Splice_451 239430 239431 1.520 Predicted splice site
Splice_452 239676 239677 2.168 Predicted splice site
Splice_453 240617 240618 1.537 Predicted splice site
Splice_454 241221 241222 1.849 Predicted splice site
Splice_455 241535 241536 1.934 Predicted splice site
Splice_456 241653 241654 1.653 Predicted splice site
Splice_457 242073 242074 1.769 Predicted splice site

Splice_458 242153 242154 1.898 Predicted splice site
Splice_459 242229 242230 1.823 Predicted splice site
lmaj_03_066 242317 244599 0 hypothetical protein,
unknown function
Splice_460 243106 243107 2.553 Predicted splice site
Splice_461 243446 243447 1.621 Predicted splice site
Splice_462 245125 245126 1.957 Predicted splice site
lmaj_03_067 245162 246871 0 hypothetical protein,
unknown function
Splice_463 247084 247085 1.835 Predicted splice site
Splice_464 247250 247251 1.989 Predicted splice site
Splice_465 248518 248519 1.898 Predicted splice site
Splice_466 248809 248810 1.554 Predicted splice site
Splice_467 249756 249757 1.668 Predicted splice site
Splice_468 250713 250714 1.572 Predicted splice site
Splice_469 250828 250829 1.823 Predicted splice site
Splice_470 251226 251227 1.898 Predicted splice site
Splice_471 251310 251311 1.945 Predicted splice site
Splice_472 252226 252227 2.827 Predicted splice site
Splice_473 253609 253610 1.621 Predicted splice site
Splice_474 253837 253838 2.033 Predicted splice site
Splice_475 253932 253933 1.898 Predicted splice site
Splice_476 254774 254775 1.621 Predicted splice site
Splice_477 255035 255036 1.501 Predicted splice site
Splice_478 255104 255105 1.756 Predicted splice site
Splice_479 255697 255698 1.713 Predicted splice site
Splice_480 256902 256903 1.945 Predicted splice site
lmaj_03_068 257045 257905 0 hypothetical protein,
unknown function
Splice_481 257079 257080 2.001 Predicted splice site
Splice_482 257809 257810 1.537 Predicted splice site
Splice_483 258204 258205 1.520 Predicted splice site
Splice_484 258831 258832 2.141 Predicted splice site
Splice_485 259901 259902 1.668 Predicted splice site
Splice_486 261914 261915 1.683 Predicted splice site
Splice_487 263077 263078 1.742 Predicted splice site
Splice_488 264683 264684 2.522 Predicted splice site
Splice_489 267128 267129 1.520 Predicted splice site
Splice_490 269880 269881 1.572 Predicted splice site
Splice_491 271117 271118 1.945 Predicted splice site
Splice_492 272195 272196 1.922 Predicted splice site
Splice_493 272606 272607 1.797 Predicted splice site
Splice_494 272975 272976 1.756 Predicted splice site
Splice_495 274434 274435 1.520 Predicted splice site
Splice_496 275624 275625 1.910 Predicted splice site

Splice_497 276860 276861 1.554 Predicted splice site
Splice_498 277376 277377 2.064 Predicted splice site
Splice_499 277521 277522 2.230 Predicted splice site
Splice_500 278073 278074 1.756 Predicted splice site
Splice_501 278484 278485 1.621 Predicted splice site
Splice_502 281929 281930 1.957 Predicted splice site
Splice_503 283079 283080 2.001 Predicted splice site
Splice_504 284060 284061 1.554 Predicted splice site
Splice_505 284953 284954 2.132 Predicted splice site
Splice_506 285244 285245 1.520 Predicted splice site
Splice_507 286142 286143 2.187 Predicted splice site
Splice_508 286400 286401 3.025 Predicted splice site
Splice_509 286680 286681 1.653 Predicted splice site
Splice_510 286930 286931 2.280 Predicted splice site
Splice_511 288880 288881 1.554 Predicted splice site
Splice_512 289439 289440 1.537 Predicted splice site
Splice_513 290698 290699 1.861 Predicted splice site
Splice_514 290787 290788 1.967 Predicted splice site
Splice_515 292618 292619 1.809 Predicted splice site
Splice_516 293242 293243 1.728 Predicted splice site
Splice_517 293889 293890 1.756 Predicted splice site
Splice_518 295287 295288 1.713 Predicted splice site
Splice_519 297951 297952 1.588 Predicted splice site
Splice_520 299094 299095 1.588 Predicted splice site
Splice_521 299655 299656 1.979 Predicted splice site
Splice_522 300182 300183 1.797 Predicted splice site
Splice_523 300561 300562 1.769 Predicted splice site
Splice_524 301577 301578 1.501 Predicted splice site
Splice_525 301669 301670 1.809 Predicted splice site
Splice_526 302584 302585 1.605 Predicted splice site
Splice_527 304538 304539 2.195 Predicted splice site
Splice_528 304689 304690 1.934 Predicted splice site
Splice_529 304964 304965 1.588 Predicted splice site
Splice_530 305147 305148 2.084 Predicted splice site
Splice_531 305779 305780 2.012 Predicted splice site
Splice_532 306777 306778 1.637 Predicted splice site
Splice_533 309985 309986 1.668 Predicted splice site
Splice_534 311695 311696 1.713 Predicted splice site
Splice_535 313762 313763 1.797 Predicted splice site
Splice_536 314829 314830 1.621 Predicted splice site
Splice_537 315127 315128 2.064 Predicted splice site
Splice_538 316522 316523 1.605 Predicted splice site
Splice_539 318001 318002 1.728 Predicted splice site
Splice_540 321971 321972 1.783 Predicted splice site
Splice_541 323498 323499 2.221 Predicted splice site

```
Splice_542 324475 324476 1.554 Predicted splice site
Splice_543 325253 325254 2.280 Predicted splice site
Splice_544 326252 326253 1.668 Predicted splice site
Splice_545 328148 328149 1.742 Predicted splice site
Splice_546 328458 328459 1.698 Predicted splice site
Splice_547 329001 329002 1.572 Predicted splice site
Splice_548 329367 329368 1.501 Predicted splice site
Splice_549 331884 331885 1.823 Predicted splice site
Splice_550 334590 334591 1.835 Predicted splice site
Splice_551 335458 335459 1.756 Predicted splice site
Splice_552 338023 338024 1.588 Predicted splice site
Splice_553 338239 338240 1.520 Predicted splice site
Splice_554 338741 338742 1.769 Predicted splice site
Splice_555 339092 339093 1.572 Predicted splice site
Splice_556 339984 339985 2.195 Predicted splice site
Splice_557 340375 340376 1.501 Predicted splice site
Splice_558 344129 344130 1.520 Predicted splice site
Splice_559 347039 347040 1.520 Predicted splice site
Splice_560 348419 348420 1.637 Predicted splice site
Splice_561 350937 350938 1.637 Predicted splice site
Splice_562 352345 352346 1.605 Predicted splice site
Splice_563 358195 358196 1.957 Predicted splice site
Splice_564 362920 362921 1.967 Predicted splice site
Splice_565 364683 364684 1.637 Predicted splice site
Splice_566 364979 364980 2.012 Predicted splice site
Splice_567 366585 366586 1.934 Predicted splice site
Splice_568 367412 367413 2.033 Predicted splice site
Splice_569 368250 368251 1.713 Predicted splice site
Splice_570 368408 368409 1.769 Predicted splice site
Splice_571 369190 369191 1.898 Predicted splice site
Splice_572 373588 373589 1.605 Predicted splice site
Splice_573 373795 373796 1.797 Predicted splice site
Splice_574 375446 375447 2.064 Predicted splice site
Splice_575 375550 375551 1.783 Predicted splice site
Splice_576 378466 378467 2.012 Predicted splice site
Splice_577 380226 380227 2.204 Predicted splice site
Splice_578 381138 381139 1.520 Predicted splice site
Splice_579 381214 381215 1.849 Predicted splice site
Splice_580 381499 381500 2.366 Predicted splice site
Splice_581 382291 382292 1.728 Predicted splice site
Splice_582 382884 382885 1.668 Predicted splice site
```

Predictions for the Reverse Strand of Chromosome 3

Splice_1 688 689 1.605 Predicted splice site
Splice_2 742 743 1.520 Predicted splice site
Splice_3 799 800 1.520 Predicted splice site
lmaj_03_098 1944 3242 0 Elongation Initiation Factor 2,
alpha subunit,
Splice_4 1974 1975 2.522 Predicted splice site
Splice_5 3422 3423 1.797 Predicted splice site
Splice_6 3529 3530 2.053 Predicted splice site
Splice_7 3830 3831 2.113 Predicted splice site
lmaj_03_097 3925 4989 0 conserved hypothetical protein,
unknown
Splice_8 3955 3956 2.327 Predicted splice site
Splice_9 5112 5113 1.910 Predicted splice site
Splice_10 5463 5464 2.320 Predicted splice site
lmaj_03_096 5531 5845 0 hypothetical protein,
unknown function
Splice_11 6447 6448 2.387 Predicted splice site
Splice_12 6556 6557 1.572 Predicted splice site
lmaj_03_095 6740 8410 0 oxidase-like protein
Splice_13 8713 8714 2.350 Predicted splice site
Splice_14 8801 8802 1.989 Predicted splice site
Splice_15 8933 8934 2.239 Predicted splice site
Splice_16 9389 9390 2.022 Predicted splice site
Splice_17 9661 9662 1.637 Predicted splice site
lmaj_03_094 9896 10882 0 hypothetical protein,
unknown function
Splice_18 10966 10967 1.742 Predicted splice site
Splice_19 11063 11064 1.885 Predicted splice site
Splice_20 11417 11418 2.652 Predicted splice site
Splice_21 11494 11495 1.861 Predicted splice site
lmaj_03_093 11508 13601 0 hypothetical protein,
unknown function
Splice_22 11857 11858 1.605 Predicted splice site
Splice_23 12348 12349 1.898 Predicted splice site
Splice_24 13656 13657 1.823 Predicted splice site
Splice_25 13720 13721 1.683 Predicted splice site
Splice_26 13857 13858 2.416 Predicted splice site
Splice_27 14159 14160 2.646 Predicted splice site
Splice_28 14360 14361 2.256 Predicted splice site
Splice_29 14579 14580 2.103 Predicted splice site
lmaj_03_092 14665 15321 0 hypothetical conserved protein,
unknown
Splice_30 14934 14935 1.537 Predicted splice site

Splice_31 15035 15036 1.728 Predicted splice site
Splice_32 15679 15680 2.380 Predicted splice site
Splice_33 15947 15948 1.698 Predicted splice site
lmaj_03_091 16128 16808 0 Inorganic Pyrophosphatase-like protein
Splice_34 16234 16235 1.823 Predicted splice site
Splice_35 17121 17122 1.809 Predicted splice site
Splice_36 17331 17332 1.885 Predicted splice site
Splice_37 17423 17424 1.797 Predicted splice site
Splice_38 17655 17656 2.618 Predicted splice site
Splice_39 17953 17954 2.022 Predicted splice site
lmaj_03_090 17972 19096 0 second-step splicing factor-like protein
Splice_40 19262 19263 2.053 Predicted splice site
Splice_41 19390 19391 2.350 Predicted splice site
Splice_42 20236 20237 2.380 Predicted splice site
lmaj_03_089 21327 23543 0 hypothetical protein, unknown function
Splice_43 23807 23808 1.945 Predicted splice site
Splice_44 23890 23891 1.934 Predicted splice site
lmaj_03_088 24461 30262 0 hypothetical protein, unknown function
Splice_45 30390 30391 2.103 Predicted splice site
Splice_46 30469 30470 1.797 Predicted splice site
Splice_47 30806 30807 1.520 Predicted splice site
Splice_48 31021 31022 1.554 Predicted splice site
lmaj_03_087 31242 35414 0 Leucine Rich Repeat containing protein,
Splice_49 31247 31248 1.668 Predicted splice site
Splice_50 33196 33197 1.572 Predicted splice site
Splice_51 35718 35719 2.103 Predicted splice site
Splice_52 35986 35987 1.668 Predicted splice site
lmaj_03_086 36369 40760 0 hypothetical protein, unknown function
Splice_53 41286 41287 1.967 Predicted splice site
Splice_54 41533 41534 2.471 Predicted splice site
Splice_55 41694 41695 1.756 Predicted splice site
lmaj_03_085 41698 48846 0 hypothetical protein, unknown function
Splice_56 42662 42663 1.979 Predicted splice site
Splice_57 44547 44548 1.520 Predicted splice site
Splice_58 46820 46821 2.423 Predicted splice site
Splice_59 48447 48448 1.537 Predicted splice site
Splice_60 48987 48988 2.012 Predicted splice site
Splice_61 49053 49054 1.713 Predicted splice site

Splice_62 49586 49587 2.132 Predicted splice site
Splice_63 49686 49687 1.713 Predicted splice site
lmaj_03_084 49706 52630 0 hypothetical protein,
unknown function
Splice_64 50100 50101 1.621 Predicted splice site
Splice_65 52887 52888 1.835 Predicted splice site
Splice_66 53144 53145 2.335 Predicted splice site
Splice_67 53591 53592 2.967 Predicted splice site
lmaj_03_083 53730 54941 0 hypothetical protein,
unknown function
Splice_68 53737 53738 1.756 Predicted splice site
Splice_69 55109 55110 2.053 Predicted splice site
Splice_70 55432 55433 1.809 Predicted splice site
Splice_71 55517 55518 1.861 Predicted splice site
Splice_72 55667 55668 1.898 Predicted splice site
Splice_73 56007 56008 2.840 Predicted splice site
Splice_74 56336 56337 2.577 Predicted splice site
lmaj_03_082 56454 65264 0 hypothetical protein,
unknown function
Splice_75 56689 56690 1.501 Predicted splice site
Splice_76 56927 56928 1.588 Predicted splice site
Splice_77 60599 60600 1.885 Predicted splice site
Splice_78 65329 65330 1.520 Predicted splice site
Splice_79 65574 65575 2.534 Predicted splice site
Splice_80 65776 65777 2.022 Predicted splice site
Splice_81 65854 65855 1.874 Predicted splice site
lmaj_03_081 66053 69289 0 hypothetical protein,
unknown function
Splice_82 67418 67419 2.064 Predicted splice site
Splice_83 69354 69355 1.554 Predicted splice site
Splice_84 69672 69673 2.350 Predicted splice site
Splice_85 70087 70088 2.012 Predicted splice site
Splice_86 70219 70220 2.012 Predicted splice site
Splice_87 70317 70318 1.823 Predicted splice site
Splice_88 70448 70449 2.187 Predicted splice site
lmaj_03_080 70538 77806 0 6-PhosphoFructo-2-Kinase-like
protein
Splice_89 70541 70542 1.653 Predicted splice site
Splice_90 72115 72116 2.132 Predicted splice site
Splice_91 75186 75187 1.934 Predicted splice site
Splice_92 76497 76498 1.769 Predicted splice site
Splice_93 78103 78104 2.204 Predicted splice site
Splice_94 78188 78189 1.957 Predicted splice site
Splice_95 78507 78508 2.141 Predicted splice site
lmaj_03_079 78532 79839 0 hypothetical protein,

unknown function
Splice_96 80315 80316 2.187 Predicted splice site
Splice_97 80502 80503 1.989 Predicted splice site
Splice_98 81170 81171 2.931 Predicted splice site
Splice_99 81332 81333 1.653 Predicted splice site
Splice_100 81450 81451 2.272 Predicted splice site
Splice_101 81528 81529 1.683 Predicted splice site
Splice_102 81607 81608 1.885 Predicted splice site
lmaj_03_078 81616 90954 0 hypothetical protein,
unknown function
Splice_103 81846 81847 1.520 Predicted splice site
Splice_104 82751 82752 1.588 Predicted splice site
Splice_105 83638 83639 1.668 Predicted splice site
Splice_106 91228 91229 1.849 Predicted splice site
Splice_107 91612 91613 2.668 Predicted splice site
Splice_108 91758 91759 2.043 Predicted splice site
lmaj_03_077 91788 93428 0 hypothetical protein,
unknown function
Splice_109 93746 93747 2.141 Predicted splice site
Splice_110 93861 93862 1.809 Predicted splice site
Splice_111 94073 94074 2.073 Predicted splice site
Splice_112 94194 94195 1.945 Predicted splice site
lmaj_03_076 94273 95223 0 hypothetical protein,
unknown function
Splice_113 95642 95643 2.053 Predicted splice site
Splice_114 95915 95916 2.372 Predicted splice site
lmaj_03_075 96084 98351 0 hypothetical protein,
unknown function
Splice_115 98890 98891 1.683 Predicted splice site
Splice_116 98969 98970 1.621 Predicted splice site
lmaj_03_074 99044 99298 0 copper chaperone-like protein
Splice_117 99377 99378 1.653 Predicted splice site
Splice_118 99522 99523 2.122 Predicted splice site
Splice_119 99861 99862 1.809 Predicted splice site
Splice_120 100034 100035 1.605 Predicted splice site
Splice_121 100161 100162 2.103 Predicted splice site
lmaj_03_073 100183 101388 0 hypothetical protein,
unknown function
Splice_122 100383 100384 1.756 Predicted splice site
Splice_123 102158 102159 1.605 Predicted splice site
Splice_124 102845 102846 2.683 Predicted splice site
lmaj_03_072 103631 106252 0 hypothetical protein,
unknown function
Splice_125 104064 104065 1.756 Predicted splice site
Splice_126 106453 106454 1.797 Predicted splice site

Splice_127 106625 106626 1.501 Predicted splice site
Splice_128 106799 106800 1.637 Predicted splice site
lmaj_03_071 106842 107603 0 hypothetical protein,
unknown function
Splice_129 107931 107932 1.989 Predicted splice site
Splice_130 108239 108240 1.797 Predicted splice site
Splice_131 108323 108324 1.698 Predicted splice site
Splice_132 108389 108390 1.713 Predicted splice site
lmaj_03_070 108444 116918 0 hypothetical protein,
unknown function
Splice_133 111328 111329 1.537 Predicted splice site
Splice_134 116219 116220 1.572 Predicted splice site
Splice_135 117126 117127 1.797 Predicted splice site
Splice_136 117537 117538 1.605 Predicted splice site
Splice_137 117604 117605 1.728 Predicted splice site
lmaj_03_069 117704 124339 0 hypothetical protein,
unknown function
Splice_138 121428 121429 1.572 Predicted splice site
Splice_139 125028 125029 2.150 Predicted splice site
Splice_140 128189 128190 1.698 Predicted splice site
Splice_141 128544 128545 1.783 Predicted splice site
Splice_142 129975 129976 1.742 Predicted splice site
Splice_143 130866 130867 1.537 Predicted splice site
Splice_144 131100 131101 1.572 Predicted splice site
Splice_145 131159 131160 1.605 Predicted splice site
Splice_146 131647 131648 2.012 Predicted splice site
Splice_147 133537 133538 1.809 Predicted splice site
Splice_148 134130 134131 2.022 Predicted splice site
Splice_149 134577 134578 1.588 Predicted splice site
Splice_150 134799 134800 1.588 Predicted splice site
Splice_151 134913 134914 1.605 Predicted splice site
Splice_152 135080 135081 1.934 Predicted splice site
Splice_153 135150 135151 1.520 Predicted splice site
Splice_154 135227 135228 1.861 Predicted splice site
Splice_155 135528 135529 1.898 Predicted splice site
Splice_156 135815 135816 1.605 Predicted splice site
Splice_157 136285 136286 2.416 Predicted splice site
Splice_158 136369 136370 1.945 Predicted splice site
Splice_159 137477 137478 2.022 Predicted splice site
Splice_160 139811 139812 1.898 Predicted splice site
Splice_161 140043 140044 1.537 Predicted splice site
Splice_162 143092 143093 2.187 Predicted splice site
Splice_163 143145 143146 1.501 Predicted splice site
Splice_164 144604 144605 1.835 Predicted splice site
Splice_165 145011 145012 1.554 Predicted splice site

```
Splice_166 145423 145424 1.572 Predicted splice site
Splice_167 145519 145520 1.501 Predicted splice site
Splice_168 147429 147430 1.910 Predicted splice site
Splice_169 147562 147563 1.637 Predicted splice site
Splice_170 147657 147658 1.605 Predicted splice site
Splice_171 148486 148487 1.967 Predicted splice site
Splice_172 150566 150567 1.588 Predicted splice site
Splice_173 151798 151799 1.588 Predicted splice site
Splice_174 153149 153150 2.132 Predicted splice site
Splice_175 156737 156738 1.898 Predicted splice site
Splice_176 156871 156872 2.395 Predicted splice site
Splice_177 157044 157045 2.490 Predicted splice site
Splice_178 157475 157476 1.874 Predicted splice site
Splice_179 159574 159575 2.256 Predicted splice site
Splice_180 159685 159686 1.934 Predicted splice site
Splice_181 161244 161245 1.885 Predicted splice site
Splice_182 161388 161389 2.168 Predicted splice site
Splice_183 163906 163907 2.256 Predicted splice site
Splice_184 165440 165441 1.621 Predicted splice site
Splice_185 166420 166421 2.053 Predicted splice site
Splice_186 166626 166627 1.520 Predicted splice site
Splice_187 166882 166883 1.742 Predicted splice site
Splice_188 167063 167064 1.756 Predicted splice site
Splice_189 167131 167132 1.742 Predicted splice site
Splice_190 167529 167530 1.728 Predicted splice site
Splice_191 169119 169120 1.621 Predicted splice site
Splice_192 169481 169482 1.713 Predicted splice site
Splice_193 169846 169847 1.769 Predicted splice site
Splice_194 170470 170471 1.922 Predicted splice site
Splice_195 170763 170764 2.053 Predicted splice site
Splice_196 174728 174729 1.861 Predicted splice site
Splice_197 175486 175487 1.809 Predicted splice site
Splice_198 175628 175629 1.823 Predicted splice site
Splice_199 176124 176125 2.150 Predicted splice site
Splice_200 177339 177340 2.256 Predicted splice site
Splice_201 177653 177654 1.520 Predicted splice site
Splice_202 177746 177747 1.874 Predicted splice site
Splice_203 177816 177817 1.769 Predicted splice site
Splice_204 179215 179216 1.605 Predicted splice site
Splice_205 180214 180215 2.043 Predicted splice site
Splice_206 180744 180745 2.168 Predicted splice site
Splice_207 183509 183510 1.605 Predicted splice site
Splice_208 183729 183730 2.195 Predicted splice site
Splice_209 183914 183915 1.967 Predicted splice site
Splice_210 184157 184158 1.520 Predicted splice site
```

Splice_211 184220 184221 1.668 Predicted splice site
Splice_212 188061 188062 1.501 Predicted splice site
Splice_213 190682 190683 1.572 Predicted splice site
Splice_214 192192 192193 2.141 Predicted splice site
Splice_215 192688 192689 2.073 Predicted splice site
Splice_216 192752 192753 1.683 Predicted splice site
Splice_217 192859 192860 1.653 Predicted splice site
Splice_218 192995 192996 1.713 Predicted splice site
Splice_219 194579 194580 1.572 Predicted splice site
Splice_220 195547 195548 1.967 Predicted splice site
Splice_221 198699 198700 1.572 Predicted splice site
Splice_222 199092 199093 2.022 Predicted splice site
Splice_223 200328 200329 1.588 Predicted splice site
Splice_224 201247 201248 1.683 Predicted splice site
Splice_225 202462 202463 2.247 Predicted splice site
Splice_226 203294 203295 1.698 Predicted splice site
Splice_227 203427 203428 1.520 Predicted splice site
Splice_228 203503 203504 1.653 Predicted splice site
Splice_229 203810 203811 1.957 Predicted splice site
Splice_230 203908 203909 1.728 Predicted splice site
Splice_231 204033 204034 2.103 Predicted splice site
Splice_232 204588 204589 1.554 Predicted splice site
Splice_233 204869 204870 1.979 Predicted splice site
Splice_234 204957 204958 1.989 Predicted splice site
Splice_235 206608 206609 1.713 Predicted splice site
Splice_236 208838 208839 1.713 Predicted splice site
Splice_237 210616 210617 1.621 Predicted splice site
Splice_238 211046 211047 2.395 Predicted splice site
Splice_239 211716 211717 1.501 Predicted splice site
Splice_240 211947 211948 1.668 Predicted splice site
Splice_241 212312 212313 2.477 Predicted splice site
Splice_242 212561 212562 1.683 Predicted splice site
Splice_243 212973 212974 2.132 Predicted splice site
Splice_244 213065 213066 2.033 Predicted splice site
Splice_245 213367 213368 1.537 Predicted splice site
Splice_246 213588 213589 1.572 Predicted splice site
Splice_247 214370 214371 1.885 Predicted splice site
Splice_248 216700 216701 1.572 Predicted splice site
Splice_249 217815 217816 1.637 Predicted splice site
Splice_250 218001 218002 1.588 Predicted splice site
Splice_251 218260 218261 1.756 Predicted splice site
Splice_252 219389 219390 1.756 Predicted splice site
Splice_253 220042 220043 1.668 Predicted splice site
Splice_254 222005 222006 1.910 Predicted splice site
Splice_255 223201 223202 1.554 Predicted splice site

Splice_256 224350 224351 2.380 Predicted splice site
Splice_257 224529 224530 1.683 Predicted splice site
Splice_258 224880 224881 1.967 Predicted splice site
Splice_259 225063 225064 2.387 Predicted splice site
Splice_260 225890 225891 2.064 Predicted splice site
Splice_261 230387 230388 2.141 Predicted splice site
Splice_262 233087 233088 1.501 Predicted splice site
Splice_263 233669 233670 1.668 Predicted splice site
Splice_264 237661 237662 1.683 Predicted splice site
Splice_265 239618 239619 1.698 Predicted splice site
Splice_266 240392 240393 1.809 Predicted splice site
Splice_267 241211 241212 1.520 Predicted splice site
Splice_268 241343 241344 1.809 Predicted splice site
Splice_269 242769 242770 1.809 Predicted splice site
Splice_270 242854 242855 1.653 Predicted splice site
Splice_271 245261 245262 2.022 Predicted splice site
Splice_272 247661 247662 1.835 Predicted splice site
Splice_273 248763 248764 1.957 Predicted splice site
Splice_274 248883 248884 1.769 Predicted splice site
Splice_275 249161 249162 1.637 Predicted splice site
Splice_276 249880 249881 1.874 Predicted splice site
Splice_277 250183 250184 1.967 Predicted splice site
Splice_278 250493 250494 2.583 Predicted splice site
Splice_279 251686 251687 2.380 Predicted splice site
Splice_280 253666 253667 1.861 Predicted splice site
Splice_281 253725 253726 1.605 Predicted splice site
Splice_282 254017 254018 2.335 Predicted splice site
Splice_283 254692 254693 1.967 Predicted splice site
Splice_284 255121 255122 2.387 Predicted splice site
Splice_285 255303 255304 1.756 Predicted splice site
Splice_286 256428 256429 1.520 Predicted splice site
Splice_287 258245 258246 1.554 Predicted splice site
Splice_288 260566 260567 3.329 Predicted splice site
Splice_289 260987 260988 1.501 Predicted splice site
Splice_290 262803 262804 1.769 Predicted splice site
Splice_291 264462 264463 1.756 Predicted splice site
Splice_292 265212 265213 2.239 Predicted splice site
Splice_293 265548 265549 2.022 Predicted splice site
Splice_294 270793 270794 1.989 Predicted splice site
Splice_295 270974 270975 1.835 Predicted splice site
Splice_296 271217 271218 1.668 Predicted splice site
Splice_297 271497 271498 1.537 Predicted splice site
Splice_298 272241 272242 1.797 Predicted splice site
Splice_299 273589 273590 1.683 Predicted splice site
Splice_300 273669 273670 1.572 Predicted splice site

Splice_301 276690 276691 2.178 Predicted splice site
Splice_302 278228 278229 2.093 Predicted splice site
Splice_303 278371 278372 1.728 Predicted splice site
Splice_304 278506 278507 1.588 Predicted splice site
Splice_305 279486 279487 2.064 Predicted splice site
Splice_306 279875 279876 1.683 Predicted splice site
Splice_307 280238 280239 1.809 Predicted splice site
Splice_308 280974 280975 1.823 Predicted splice site
Splice_309 281750 281751 2.746 Predicted splice site
Splice_310 283071 283072 1.621 Predicted splice site
Splice_311 284709 284710 2.656 Predicted splice site
Splice_312 285655 285656 2.073 Predicted splice site
Splice_313 286141 286142 1.934 Predicted splice site
Splice_314 287470 287471 1.653 Predicted splice site
Splice_315 290698 290699 1.698 Predicted splice site
Splice_316 291226 291227 1.637 Predicted splice site
Splice_317 292763 292764 1.668 Predicted splice site
Splice_318 293111 293112 2.195 Predicted splice site
Splice_319 293519 293520 2.160 Predicted splice site
Splice_320 295021 295022 1.683 Predicted splice site
Splice_321 295181 295182 1.605 Predicted splice site
Splice_322 297587 297588 1.934 Predicted splice site
Splice_323 299383 299384 1.537 Predicted splice site
Splice_324 299639 299640 1.698 Predicted splice site
Splice_325 300602 300603 1.713 Predicted splice site
Splice_326 300743 300744 1.572 Predicted splice site
Splice_327 302104 302105 1.769 Predicted splice site
Splice_328 304493 304494 1.637 Predicted splice site
Splice_329 304801 304802 1.653 Predicted splice site
Splice_330 305419 305420 2.380 Predicted splice site
Splice_331 308575 308576 1.945 Predicted splice site
Splice_332 310994 310995 1.742 Predicted splice site
Splice_333 311123 311124 2.168 Predicted splice site
Splice_334 312863 312864 1.668 Predicted splice site
Splice_335 313049 313050 1.835 Predicted splice site
Splice_336 313124 313125 1.520 Predicted splice site
Splice_337 319572 319573 1.537 Predicted splice site
Splice_338 319807 319808 2.012 Predicted splice site
Splice_339 321703 321704 1.653 Predicted splice site
Splice_340 321906 321907 1.742 Predicted splice site
Splice_341 322099 322100 1.554 Predicted splice site
Splice_342 322360 322361 1.713 Predicted splice site
Splice_343 324529 324530 2.073 Predicted splice site
Splice_344 324919 324920 2.113 Predicted splice site
Splice_345 325546 325547 1.957 Predicted splice site

Splice_346 325645 325646 1.823 Predicted splice site
Splice_347 326276 326277 1.520 Predicted splice site
Splice_348 326401 326402 2.073 Predicted splice site
Splice_349 327018 327019 1.572 Predicted splice site
Splice_350 328224 328225 1.520 Predicted splice site
Splice_351 328713 328714 1.605 Predicted splice site
Splice_352 329131 329132 1.989 Predicted splice site
Splice_353 331109 331110 1.537 Predicted splice site
Splice_354 331178 331179 1.756 Predicted splice site
Splice_355 331278 331279 2.113 Predicted splice site
Splice_356 331561 331562 1.922 Predicted splice site
Splice_357 333293 333294 2.132 Predicted splice site
Splice_358 333460 333461 1.957 Predicted splice site
Splice_359 333587 333588 1.797 Predicted splice site
Splice_360 334383 334384 1.683 Predicted splice site
Splice_361 336818 336819 2.084 Predicted splice site
Splice_362 337321 337322 1.668 Predicted splice site
Splice_363 337767 337768 1.742 Predicted splice site
Splice_364 341915 341916 1.621 Predicted splice site
Splice_365 343002 343003 2.679 Predicted splice site
Splice_366 343302 343303 1.989 Predicted splice site
Splice_367 344148 344149 2.043 Predicted splice site
Splice_368 345009 345010 1.849 Predicted splice site
Splice_369 346088 346089 1.742 Predicted splice site
Splice_370 346220 346221 2.141 Predicted splice site
Splice_371 346417 346418 1.637 Predicted splice site
Splice_372 349789 349790 1.797 Predicted splice site
Splice_373 351132 351133 2.288 Predicted splice site
Splice_374 351301 351302 2.239 Predicted splice site
Splice_375 352378 352379 1.713 Predicted splice site
Splice_376 354231 354232 1.698 Predicted splice site
Splice_377 355238 355239 1.621 Predicted splice site
Splice_378 355412 355413 1.683 Predicted splice site
Splice_379 355616 355617 1.621 Predicted splice site
Splice_380 358328 358329 1.849 Predicted splice site
Splice_381 358701 358702 1.683 Predicted splice site
Splice_382 360733 360734 1.621 Predicted splice site
Splice_383 360848 360849 2.230 Predicted splice site
Splice_384 363760 363761 1.501 Predicted splice site
Splice_385 364549 364550 1.742 Predicted splice site
Splice_386 370518 370519 1.683 Predicted splice site
Splice_387 373642 373643 1.572 Predicted splice site
Splice_388 374233 374234 1.728 Predicted splice site
Splice_389 374906 374907 1.874 Predicted splice site
Splice_390 376674 376675 1.588 Predicted splice site

Splice_391 377152 377153 1.797 Predicted splice site
Splice_392 379003 379004 1.910 Predicted splice site
Splice_393 379751 379752 1.537 Predicted splice site
Splice_394 380037 380038 1.653 Predicted splice site
Splice_395 380201 380202 2.053 Predicted splice site
lmaj_03_001 380342 383353 0 hypothetical protein,
unknown function
Splice_396 380347 380348 1.885 Predicted splice site
Splice_397 380444 380445 1.945 Predicted splice site
Splice_398 380582 380583 1.637 Predicted splice site
Splice_399 380780 380781 1.537 Predicted splice site
Splice_400 382086 382087 1.668 Predicted splice site
Splice_401 382224 382225 1.668 Predicted splice site
Splice_402 382500 382501 1.520 Predicted splice site
Splice_403 383494 383495 2.053 Predicted splice site
Splice_404 383569 383570 1.588 Predicted splice site
Splice_405 383652 383653 1.898 Predicted splice site
Splice_406 383836 383837 2.001 Predicted splice site
Splice_407 383943 383944 1.572 Predicted splice site
Splice_408 384018 384019 1.835 Predicted splice site
Splice_409 384251 384252 1.922 Predicted splice site
Splice_410 384395 384396 1.554 Predicted splice site
